# Discovery of a Highly Selective Cell‐Active Inhibitor of the Histone Lysine Demethylases KDM2/7

**DOI:** 10.1002/anie.201706788

**Published:** 2017-11-07

**Authors:** Philip A. Gerken, Jamie R. Wolstenhulme, Anthony Tumber, Stephanie B. Hatch, Yijia Zhang, Susanne Müller, Shane A. Chandler, Barbara Mair, Fengling Li, Sebastian M. B. Nijman, Rebecca Konietzny, Tamas Szommer, Clarence Yapp, Oleg Fedorov, Justin L. P. Benesch, Masoud Vedadi, Benedikt M. Kessler, Akane Kawamura, Paul E. Brennan, Martin D. Smith

**Affiliations:** ^1^ Chemistry Research Laboratory University of Oxford 12 Mansfield Road Oxford OX1 3TA UK; ^2^ Structural Genomics Consortium and Target Discovery Institute Nuffield Department of Medicine University of Oxford Roosevelt Drive Oxford OX3 7DQ UK; ^3^ Structural Genomics Consortium University of Toronto Toronto Ontario M5G 1L7 Canada

**Keywords:** asymmetric catalysis, epigenetics, inhibitors, lysine demethylases

## Abstract

Histone lysine demethylases (KDMs) are of critical importance in the epigenetic regulation of gene expression, yet there are few selective, cell‐permeable inhibitors or suitable tool compounds for these enzymes. We describe the discovery of a new class of inhibitor that is highly potent towards the histone lysine demethylases KDM2A/7A. A modular synthetic approach was used to explore the chemical space and accelerate the investigation of key structure–activity relationships, leading to the development of a small molecule with around 75‐fold selectivity towards KDM2A/7A versus other KDMs, as well as cellular activity at low micromolar concentrations.

Chemical modifications of DNA and its associated histones regulate gene expression across the entire genome, and therefore have a profound impact on a number of fundamental biological processes.^1^ As a result, targeting the epigenetic pathways responsible for these chemical modifications may represent a pivotal approach to addressing disease at the transcription level.^2^ In order to realize the potential of epigenetics in drug discovery, a toolkit of chemical probes that selectively target individual epigenetic proteins and allow researchers to clearly identify their downstream effects is invaluable.^3^ Significant progress has been made towards the development of a library of chemical probes that target the proteins involved in histone acetylation, in particular the bromodomain family of epigenetic readers.^4^ In contrast, proteins involved in the dynamic methylation of histone lysine residues have proven to be more challenging targets, especially the histone lysine demethylases (KDMs).^5^

The majority of KDMs belong to the Jumonji C (JmjC) family of enzymes, which contain a catalytically‐active Fe^II^ ion in the active site and require a 2‐oxoglutarate (2‐OG) cofactor for demethylation in the catalytic JmjC domain.^6^ The JmjC KDMs may be divided into six sub‐families (KDM2–KDM7) based on substrate specificity, with KDM2 and KDM7 being closely related.^7^ A major challenge in generating chemical probes for KDMs is achieving selectivity between these structurally similar sub‐families. Currently, most inhibitors of the JmjC KDMs are iron‐chelating 2‐OG competitors (Figure 1).^8^, ^9^, ^10^, ^11^, ^12^, ^13^, ^14^ Although many of these molecules achieve high levels of in vitro potency, they are frequently limited by a lack of selectivity and activity in cells. Peptide inhibitors that either mimic the histone substrate or bind KDMs allosterically have also been reported,^15^ however peptides are often limited by their low cellular permeability. Herein, we describe the discovery of a first‐in‐class, cell‐permeable KDM2A/7A inhibitor that exhibits more than or equal to 75‐fold selectivity relative to other JmjC KDM sub‐families.

**Figure 1:**
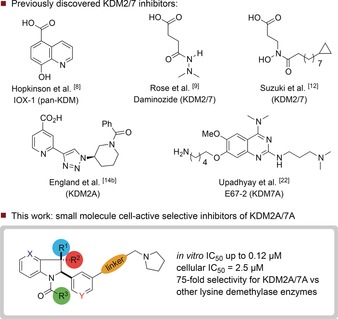
Previously discovered KDM2/7 inhibitors, and this work.

KDM2A catalyses the demethylation of mono‐ and dimethylated lysine 36 on histone H3 (H3K36).^16^ The enzyme has been reported to be involved in the regulation of NF‐κB signalling^17^ and the control of stem‐cell differentiation and proliferation.^18^ Its overexpression in gastric and small‐cell lung cancer cells suggests that inhibiting KDM2A may represent a strategy for targeting certain cancers at the transcription level.^19^, ^20^ All KDM2A inhibitors described to date are 2‐OG competitors, and none are truly selective. In addition, many 2‐OG competitors show reduced activity in cells, mainly due to poor cellular permeability and the high intracellular concentration of 2‐OG.^21^ We therefore envisioned an inhibitor that would mimic the structure of the histone substrate rather than the 2‐OG co‐substrate, as postulated for the KDM7A inhibitor E67‐2.^22^

To identify a starting point, a library of known binders to methyllysine reader domains and histone methyl transferases was screened for inhibitory activity against a panel of KDMs, since we reasoned that such a specialized library would be more likely to contain molecules that also interact with demethylases. Compound **1**, which was prepared as a putative methyllysine binding domain inhibitor,^23^ was identified as a promising candidate for further optimization against KDM2A. However, attempts to significantly improve potency by functionalizing at the indole NH position and varying the aromatic substituent on the indole C‐2 position were unsuccessful. The pyrrolidine moiety in compound **1** was shown to be critical for potency, which we postulated was due to its role as a H3K36me2 mimic. Preliminary docking studies of **1** with KDM2A (PDB ID: 2YU1; Figure S11 and Section S8 in the Supporting Information) indicated a potential for occupancy of the peptide binding site on the enzyme. Based on this initial model, we subsequently hypothesized that inhibitory activity towards KDM2A might be improved by replacing the original indole scaffold with a saturated indoline ring system. We envisioned an exploration of three‐dimensional chemical space around this core, with the aim of augmenting selectivity through increasing complexity.^24^ To achieve this, a modular synthetic approach was employed to generate a library of indoline‐containing compounds and identify key structure–activity relationships (Scheme 1 A).

**Scheme 1:**
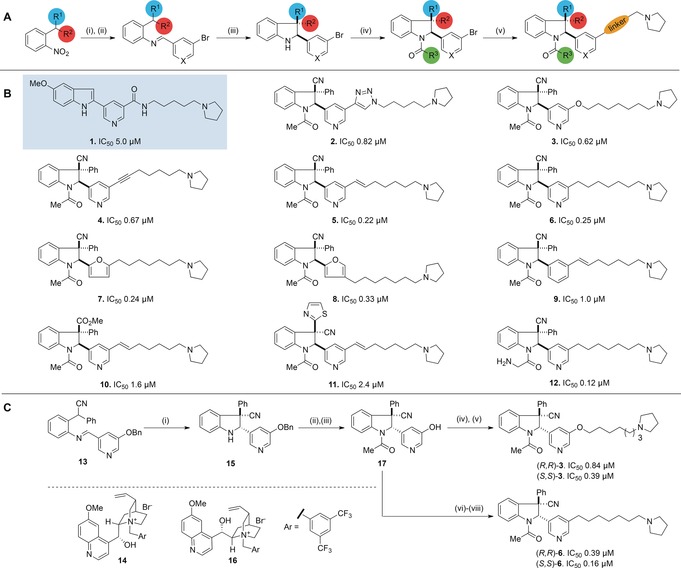
A) General synthetic strategy for racemic synthesis of KDM2A inhibitors. Reagents and conditions: i) Zn powder (10 equiv), NH_4_Cl (15 equiv), 5:1 acetone/H_2_O or H_2_, Pd/C (10 % w/w), EtOAc; ii) ArCHO (1.3 equiv), MgSO_4_ (5 equiv), PhMe or ArCHO (1.3 equiv), pyrrolidine (0.1 equiv), 3 Å sieves, CH_2_Cl_2_; iii) KO^*t*^Bu (1.1 equiv), PhMe, 0 °C; iv) R^3^COCl (2–5 equiv), pyridine (2–5 equiv), CH_2_Cl_2_; v) metal‐catalysed cross‐coupling. B) Key structure–activity relationships. IC_50_ values were determined by RapidFire MS and confirmed by AlphaScreen. All compounds are racemic. C) Catalytic enantioselective synthesis. Reagents and conditions: i) CsOH⋅H_2_O (2.0 equiv), catalyst **14**/**16** (10 mol %), PhMe, −30 °C. Catalyst **14**: 89 %, d.r. 10:1, e.r. 88:12; catalyst **16**: 84 %, d.r. 7:1, e.r. 17:83; ii) CH_3_COCl (2.0 equiv), pyridine (2.0 equiv), CH_2_Cl_2_; iii) H_2_, Pd/C (10 % w/w), 88 % (2 steps); iv) K_2_CO_3_ (5 equiv), Br(CH_2_)_7_Br (4 equiv), acetone, reflux, 38 %; v) K_2_CO_3_ (5 equiv) pyrrolidine (4 equiv), CH_3_CN, 65 %; vi) PhN(SO_2_CF_3_)_2_ (1.1 equiv), DIPEA (2.0 equiv), CH_2_Cl_2_, 0 °C, 85 %; vii) 7‐pyrrolidine‐hept‐1‐yne (1.5 equiv), PdCl_2_(PPh_3_)_2_ (5 mol %), CuI (5 mol %), HN(^*i*^Pr)_2_, 75 °C, 79 %; viii) H_2_, Pd/C (10 % w/w), MeOH, 74 %. DIPEA=(^*i*^Pr)_2_NEt.

A base‐mediated 5‐*endo*‐*trig* cyclization of a C‐2‐substituted aromatic imine afforded the racemic indoline core with two adjacent stereocenters. Subsequent acylation of the indoline ring system conferred stability towards oxidation and provided a handle for modulating polarity. Finally, metal‐catalysed cross‐coupling of the aryl bromide provided access to a variety of linkers between the indoline core and pyrrolidine capping group. In total, 45 racemic compounds were synthesized, and IC_50_ values for inhibition of KDM2A were determined using two orthogonal enzyme activity assays: AlphaScreen^25^ and RapidFire MS^26^ (see Section S3.1 in the Supporting Information for complete inhibition data). Key structure–activity relationships are summarized below (Scheme 1 B, compounds **2**–**12**). We examined different linkers and found that triazole (**2**), ether (**3**), and alkyne (**4**) linkers were well tolerated, with significantly lower IC_50_ values than the original hit. Reduction of the alkyne functional group in **4** to an alkene (**5**) or an alkane (**6**) also improved potency. Molecules containing a pyridine ring at the indoline C‐2 position were marginally more active than analogues bearing other aromatic groups such as furan (**7** or **8**) and significantly more active than a substituted benzene (**9**). In addition, pyridine‐containing compounds displayed the highest selectivity towards KDM2A (Section S3.1). Exploration of substituents at the all‐carbon quaternary stereocenter as in **10** and **11** demonstrated that a Ph,CN combination gave rise to the most potent series of compounds. Unfortunately, **12**, the most potent inhibitor identified, was found to be reactive in aqueous solution due to the susceptibility of the α‐aminoacetyl group to hydrolysis. However, the N‐acetyl group present in compounds **2**–**10** proved inert to hydrolytic cleavage. The optimal length of the linker connecting the indoline core to the pyrrolidine capping group was found to be 7–8 atoms, and replacing pyrrolidine with other secondary amines or a cyclopentyl ring led to a significant drop in potency (Section S3.1).

Having succeeded in augmenting the potency of our initial hit compound, we focused on the development of enantioselective syntheses of **3** and **6** using a counterion‐mediated strategy (Scheme 1 C).^27^ Cyclization of imine **13** with CsOH⋅H_2_O in the presence of quinine‐derived salt **14** afforded (*S*,*S*)‐**15** as the major product (10:1 d.r.) with 88:12 e.r. The OBn substituent on the pyridine ring was found to be crucial for attaining good levels of stereoselectivity. Pseudoenantiomeric ammonium salt **16** afforded (*R*,*R*)‐**15** as the major product (7:1 d.r.) with acceptable e.r. (17:83). Enantiopurity was subsequently augmented (to >99:1 e.r.) by preparative HPLC. N‐Acetylation and hydrogenolysis of the benzyl group afforded common intermediate **17**, which could be converted into (*S*,*S*)‐ and (*R*,*R*)‐**3** through alkylation with 1,7‐dibromoheptane and subsequent treatment with pyrrolidine. Alternatively, (*S*,*S*)‐ and (*R*,*R*)‐**6** could be synthesized through O‐triflation, Sonogashira coupling (with 1‐(6‐heptyn)‐pyrrolidine), and reduction of the resulting alkyne.

The (*S*,*S*) enantiomers of **3** and **6** were found to be slightly more potent than their respective (*R*,*R*) analogues, and (*S*,*S*)‐**6** (IC_50_: 0.16 μm) was assessed further in a variety of biological assays. In immunofluorescence assays using HeLa cells ectopically expressing catalytically active KDM2A, a dose‐dependent increase in H3K36me2 staining was observed upon incubation with (*S*,*S*)‐**6**, reflecting augmented cellular H3K36me2 levels (Figure 2 A and Section S3.3). No significant change in H3K36me2 fluorescence was observed for cells containing constitutively inactive KDM2A (Section S3.3).^28^ Cytotoxicity towards HeLa and HAP1 cells was observed at higher concentrations (EC_50_ 22 μm and 7.1 μm respectively), and (*S*,*S*)‐**6** showed a similar effect on the viability of human fibroblasts (HDFa; EC_50_: 10 μm) to GSK‐J4, a well‐characterized chemical probe for KDM5/6^9^ (Section S3.4). This suggests a potential activity window for investigating the effects of KDM2A inhibition within cells. To profile its selectivity, (*S*,*S*)‐**6** was tested for inhibitory activity against a panel of KDMs, methyllysine binding motifs, and epigenetic enzymes. It was found to be remarkably selective towards KDM2A (≥100‐fold) relative to representatives of the other KDM sub‐families, except closely related KDM7A (H3K9/K27me2/me1 demethylase),^29^ where it was similarly potent (Figure 2 B).^30^ (*S*,*S*)‐6 was inactive towards a representative panel of methyllysine binding domains, methyl transferases, and histone acetyl transferases (Section S3.2). To our knowledge, this is the first time a KDM2A/7A‐selective small molecule has been shown to inhibit demethylation in cells, with a significant reduction in demethylation achievable at low μm concentrations. To explore its cellular activity further, the effect of (*S*,*S*)‐**6** on gene expression in HAP1 cells was monitored using a highly multiplexed 3′ mRNA sequencing method.^31^ Within a diverse panel of in‐house compounds, our series of indoline‐containing inhibitors was represented by (*S*,*S*)‐**6** and the less active close analogue **18** (IC_50_: 17 μm, Figure 2 C).

**Figure 2:**
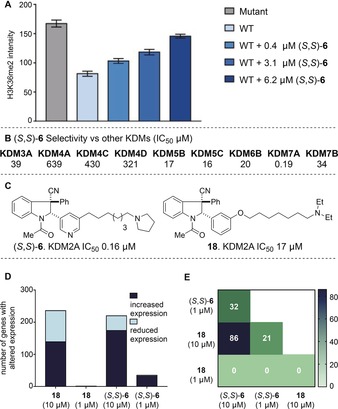
A) (*S*,*S*)‐**6** inhibits KDM2A catalysed demethylation of H3K36me2 in HeLa cells at μm concentrations. Mutant: cells contain constitutively inactive KDM2A. WT: cells contain active KDM2A. B) AlphaScreen IC_50_ values (μm) of (*S*,*S*)‐**6** against other KDMs. C) **18** is a less active close analogue of (*S*,*S*)‐**6**. D) Both (*S*,*S*)‐**6** and **18** affect gene expression in HAP1 cells at high concentrations, but only (*S*,*S*)‐**6** has an effect at low concentrations. E) Overlap of gene expression changes for (*S*,*S*)‐**6** and **18**.

When dosed at a concentration of 10 μm, both molecules influenced the expression levels of more than 200 genes. However, at a concentration of 1 μm, only the active analogue (*S*,*S*)‐**6** had a significant effect on expression levels (Figure 2 D and Section S4). We postulate that this concentration dependence is a consequence of predominantly off‐target effects at high concentrations, as opposed to a more specific effect resulting from KDM2A/7A inhibition at low concentrations. The overlap of the gene expression signatures of (*S*,*S*)‐**6** and **18** is depicted in Figure 2 E.

Obtaining a co‐crystal structure of (*S*,*S*)‐**6** bound to KDM2A proved challenging, and hence, non‐denaturing mass spectrometry (MS) experiments were performed to determine the binding stoichiometry of (*S*,*S*)‐**6** to KDM2A. KDM2A was incubated with (*S*,*S*)‐**6** and subsequently introduced into a mass spectrometer under conditions optimized for the preservation of noncovalent interactions.^32^ The native mass spectrum (Figure 3) shows 1:1 binding of (*S*,*S*)‐**6** to KDM2A. To verify the identity of bound (*S*,*S*)‐**6**, we performed tandem‐MS on the 14+ charge state, resulting in the removal of (*S*,*S*)‐**6** as a singly charged species (Figure 3 C, see inset).

**Figure 3:**
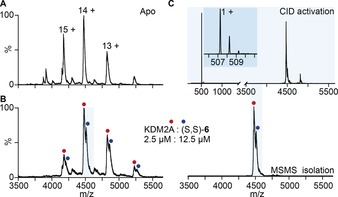
Non‐denaturing MS indicates 1:1 binding of (*S*,*S*)‐**6** to KDM2A. A) Non‐denaturing mass spectrum of apo KDM2A. B) Non‐denaturing mass spectrum of KDM2A (2.5 μm) and the 1:1 complex with (*S*,*S*)‐**6** (12.5 μm). C) The 14^+^ charge state of the complex was selected (lower) and subjected to collisional activation (upper) to release bound (*S*,*S*)‐**6** (inset). The spectrum intensity has been magnified 1.5‐fold above 3500 *m*/*z* (CID=collision‐induced dissociation).

Kinetic analyses subsequently revealed that (*S*,*S*)‐**6** does not display competitive inhibition kinetics with respect to either 2‐OG or the peptide substrate (Section S6), thus suggesting a different mode of inhibition to the majority of previously discovered KDM inhibitors.^33^ Consistent with this observation, (*S*,*S*)‐**6** did not displace fluorescent methylstat (a “bivalent” substrate‐cofactor tracer for KDM2A) in fluorescence polarisation assays. To probe the (*S*,*S*)‐**6** binding site further, KDM2A was subjected to a photoaffinity labelling profile with a diazirine‐containing analogue of (*S*,*S*)‐**6**, and LC‐MS/MS experiments were conducted (Section S7). The majority of covalently modified residues were found to be either aspartic or glutamic acids, thus suggesting the formation of a relatively long‐lived electrophilic intermediate following photo‐induced isomerization of the diazirine to a diazo compound.^34^ While this precludes the unambiguous determination of the inhibitor binding site, the observed lack of labelling within the JmjC domain active site (Section S7) is consistent with the observed lack of competitive inhibition with respect to either 2‐OG or the peptide substrate. This may indicate the presence of an alternative (allosteric) binding site specific to KDM2A/7A, although further investigation is necessary to demonstrate this clearly.

In conclusion, we have developed a potent and selective first‐in‐class inhibitor of the histone lysine demethylases KDM2A/7A. Compound (*S*,*S*)‐**6** displays more than 75 fold selectivity towards KDM2A/7A versus other JmjC lysine demethylases and is, to our knowledge, the first reported selective KDM2A/7A inhibitor that has been demonstrated to reduce H3K36me2 demethylation within cells. This study demonstrates how the generation of three‐dimensional scaffolds bearing significant saturation and multiple chiral centres can lead to the discovery of selective compounds that may be useful in the study of a challenging epigenetic target.

## Conflict of interest

The authors declare no conflict of interest.

## Supporting information

As a service to our authors and readers, this journal provides supporting information supplied by the authors. Such materials are peer reviewed and may be re‐organized for online delivery, but are not copy‐edited or typeset. Technical support issues arising from supporting information (other than missing files) should be addressed to the authors.

Supplementary
