# Supplementary material for: Discovery of a Highly Selective Cell‐Active Inhibitor of the Histone Lysine Demethylases KDM2/7
Source: Angew Chem Int Ed Engl. 2017 Nov 7;56(49):15555–9. doi: 10.1002/anie.201706788 (PMC5725665; doi:10.1002/anie.201706788)
Supplement: Supplementary file 1 — Supplementary [file ANIE-56-15555-s001.pdf]

Angewandte Chemie
Eine Zeitschrift der Gesellschaft Deutscher Chemiker

Supporting Information

Discovery of a Highly Selective Cell-Active Inhibitor of the Histone Lysine Demethylases KDM2/7

Philip A. Gerken, Jamie R. Wolstenhulme, Anthony Tumber, Stephanie B. Hatch, Yijia Zhang, Susanne Müller, Shane A. Chandler, Barbara Mair, Fengling Li, Sebastian M. B. Nijman, Rebecca Konietzny, Tamas Szommer, Clarence Yapp, Oleg Fedorov, Justin L. P. Benesch, Masoud Vedadi, Benedikt M. Kessler, Akane Kawamura, Paul E. Brennan, and Martin D. Smith**

anie_201706788_sm_miscellaneous_information.pdf

Supporting Information

Table of Contents

I) Synthesis – General Information 2

II) Synthesis – Experimental Procedures 4

2.1 General Experimental Procedures 4

2.2 Experimental Procedures 7

III) Biological Activity Assays 99

3.1 AlphaScreen and Rapidfire assays 99

3.2 Selectivity-screening against other epigenetic writer and reader proteins 106

3.3 Cellular immunofluorescence activity assay and HeLa cytotoxicity 109

3.4 Additional viability assays 110

IV) Transcriptomics 111

V) Native Mass-Spectrometry 123

VI) Mode of Action Studies 124

VII) Photoaffinity Labelling 126

VIII) Computational Docking 128

IX) NMR Spectra 129

X) HPLC Traces 260

XI) X-ray Crystallography Data 267

XII) References 269

I) Synthesis – General Information

Reaction Conditions

Reactions were carried out in flame-dried glassware under an atmosphere of argon unless stated otherwise. Room temperature (rt) refers to 20-25 °C. Temperatures of 0 °C were obtained using an ice/water bath. Temperatures of -78 °C were obtained using a dry ice/acetone bath. Reflux conditions were obtained using an oil bath or a Drysyn® heating block equipped with a contact thermometer. Temperatures of 0 °C or below which had to be maintained for extended periods of time were obtained using a Julabo FT902 immersion cooler.

Solvents

Acetonitrile, dichloromethane, diethyl ether, methanol and tetrahydrofuran were purified by filtration through activated alumina columns employing the method of Grubbs *et al.*[1] Dimethylsulfoxide, dimethylformamide, and *N*-methylpyrrolidinone were purchased as an anhydrous solvent in a Sure/Seal™ bottle from Sigma-Aldrich. All other solvents were used as supplied without prior purification.

Reagents and Catalysts

All reagents were used directly as supplied by major chemical suppliers or following purification procedures described by Perrin and Armarego.[2]

Chromatography

Thin layer chromatography was performed on Merck Kieselgel 60 F_{254} 0.25 mm precoated aluminium plates. Product spots were visualized under UV light (λ = 254 nm) and/or by staining with potassium permanganate solution, vanillin solution, or ninhydrin solution. Flash pressure column chromatography was performed using VWR silica gel 60 (40-63 µm particle size) using head pressure by means of a nitrogen line.

Nuclear Magnetic Resonance Spectrometry

NMR spectroscopy was carried out using Bruker Avance spectrometers in the deuterated solvent stated, using the residual non-deuterated solvent signal as an internal reference. ^{19}F NMR spectra were referenced externally to $CFCl_3$ in $CDCl_3$. Magnetic field strengths are quoted in MHz and refer to the resonance frequency

of the relevant nucleus. Chemical shifts are quoted in ppm with signal splittings recorded as singlet (s), doublet (d), triplet (t), quartet (q), quintet (quin), sextet (sex), septet (sept), octet (oct), nonet (non) and multiplet (m). The abbreviation br. is to denote broad, app. to denote apparent, Ph to denote phenyl, and C=O to denote carbonyl groups. Coupling constants, *J*, are measured to the nearest 0.1 Hz for ^{1}H NMR spectra and to the nearest 1 Hz for ^{13}C NMR spectra and are presented as observed. For rotameric molecules, spectra were obtained at 348 K in deuterated benzene or deuterated dimethylsulfoxide.

Mass Spectrometry

Low resolution mass spectra were recorded on a Micromass LCT Premier spectrometer under conditions of electrospray ionization (ESI). High resolution mass spectra were carried out using Bruker MicroTOF and Micromass GCT spectrometers under conditions of electrospray ionization (ESI), field ionization (FI) and chemical ionization (CI). Values are reported as a ratio of mass to charge in Daltons.

Polarimetry

Optical rotations were recorded on a Perkin-Elmer 241 polarimeter with a path length of 1 dm (using the sodium D line, 589 nm). Concentrations are reported in g/100 mL. Temperatures are reported in °C.

Melting Points

Melting points were determined using a Reichert melting point app.aratus and are uncorrected.

HPLC

Analytical chiral HPLC was performed on a Dionex UltiMate 3000 system comprising a Dionex LPG-3400A pump, WPS-3000SL autosampler, TCC-3000SD column compartment, DAD-3000 diode-array detector, fitted with the app.ropriate Daicel Chiralpak column (dimensions: 0.46 cm ø x 25 cm) and corresponding guard column (0.4 cm ø x 1 cm). Wavelengths (λ) are reported in nm, retention times (τ_R) are reported in minutes and solvent flow rates are reported in mL min^{-1}. Semi-preparatory HPLC was performed on the same system, fitted with a YMC Chiral amylose-SA S-5µm column (dimensions: 250 x 10.0 mml.D).

II) Synthesis – Experimental Procedures

2.1 General Experimental Procedures

General Procedures for Synthesis of Class 1 Racemic Indolines:

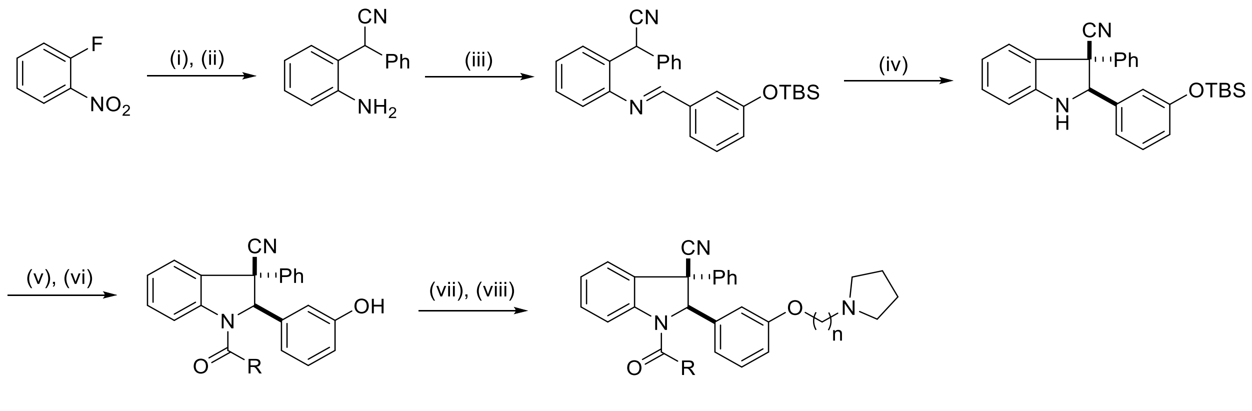

Class 1

Scheme 1: Reagents and conditions: (i) $PhCH_2CN$ (1 eq.), NaOH (50% aq., 13 eq.), NBu_4HSO_4 (1 eq.), toluene, 0 °C, 47%; (ii) Zn powder (10 eq.), NH_4Cl (15 eq.), 5:1 acetone/H_2O, rt, 82%; (iii) **21** (1.1 eq.), 3 Å mol. sieves, toluene, 100 °C, 46%; (iv) ^{t}BuOK (2 eq.), toluene, 0 °C, 81%; (v) RCOCl (1.2 eq.), pyridine (1.25 equiv), CH_2Cl_2, 0 °C, 59%-97%; (vi) TBAF (1.2 eq.), THF, rt, 62%-99%; (vii) Br-$(CH_2)_n$-Br (3 eq.), K_2CO_3 (5 eq.), acetone, reflux, 31%-94%; (viii) pyrrolidine (2 eq.), K_2CO_3 (5 eq.), CH_3CN, 60 °C, 65%-99%.

General Procedures for Synthesis of Class 2 Racemic Indolines:

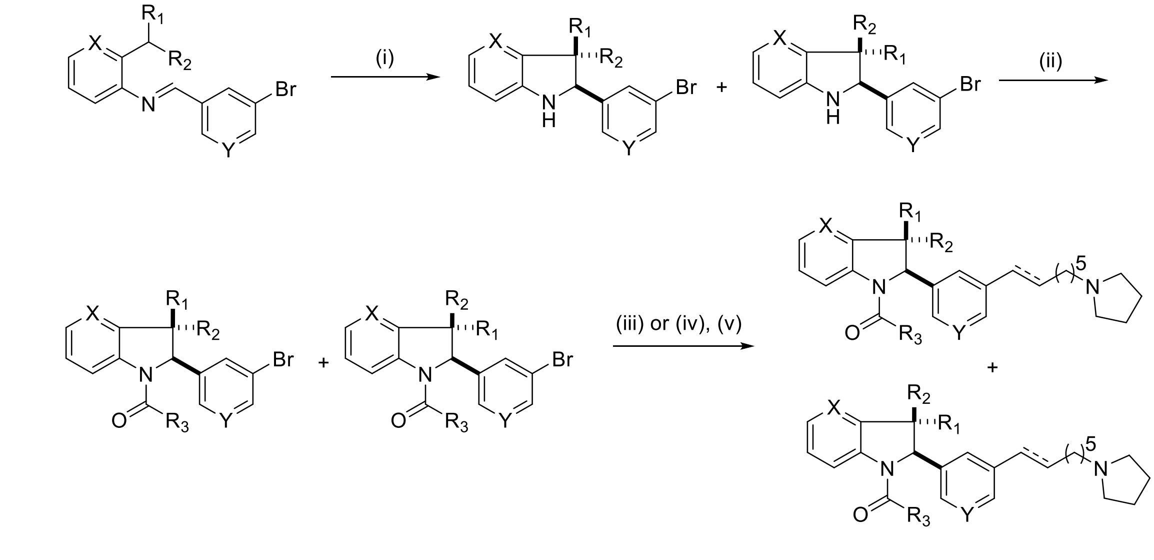

Class 2

Scheme 2: Reagents and conditions: (i) KO^{t}Bu (1.1-1.8 eq.), toluene, 0 °C. (ii) R_3COCl (2-5 eq.), pyridine (2-5 eq.), CH_2Cl_2, rt; (iii) $Bu_3SnC_7H_{12}N(C_4H_8)$ (1.2 eq.) $Pd(PPh_3)_4$ (0.1 eq.) NMP, heating; (iv) $C_7H_{11}N(C_4H_8)$ (1.2 eq.), $PdCl_2(PPh_3)_2$ (0.05 eq.), CuI (0.05 eq.), NH(^{i}Pr)$_2$, heating; (v) H_2 , Pd/C, MeOH, rt.

General Procedure 1: Racemic cyclization of imines to form indolines

KO^{t}Bu (1.1-1.8 eq.) was added to a solution of imine (1 eq.) in toluene and stirred at 0 °C until completion. NH_4Cl (saturated aq., 10 mL/mmol imine) was added, and the mixture was extracted with EtOAc. The combined organic extracts were washed with brine, dried over Na_2SO_4, filtered, and concentrated.

General Procedure 2: Acylation of indolines with acyl chlorides and chloroformates

Acyl chloride (2-5 eq.) was added portionwise/dropwise to a solution of indoline (1 eq.) and pyridine (2-5 eq.) in CH_2Cl_2. The reaction mixture was stirred at rt until completion. $NaHCO_3$ (saturated aq., 10 mL/mmol indoline) was added, and the mixture was extracted with CH_2Cl_2. The combined organic extracts were washed with brine, dried over Na_2SO_4, filtered, and concentrated.

General Procedure 3: Deprotection of TBS-protected phenols

To a solution of the appropriate TBS protected phenol (300 mg) in THF (5 mL) at room temperature was added TBAF (1.0 M in THF, 1.2 eq.) and the reaction was stirred for 10 minutes before acidifying with 1 M HCl and extracting with EtOAc. The organic layer was dried over $MgSO_4$ and evaporated under reduced pressure. Purification by column chromatography afforded the respective product.

General Procedure 4: Alkylation of phenols with alkyl dibromide

To a solution of the appropriate phenol (200 mg) in acetone (5 mL, pre-dried over 3 ÅMS) was added K_2CO_3 (5.0 eq.) and the required alkyl dibromide (3.0 eq.). The reaction was stirred at reflux overnight before cooling to room temperature and concentrating *in vacuo*. The residue was dissolved in EtOAc, washed with water and brine, dried over $MgSO_4$ and evaporated under reduced pressure. Purification by column chromatography afforded the respective product.

General Procedure 5: Alkylation of pyrrolidine

To a solution of the appropriate bromide (200 mg) in MeCN (5 mL) was added K_2CO_3 (5.0 eq.) and pyrrolidine (2.0 eq.). The reaction was heated to 60 °C overnight before diluting with EtOAc and washing with water and brine. The organic phase was dried over $MgSO_4$ and evaporated under reduced pressure. Purification by column chromatography (SiO_2, DCM/MeOH/Et_3N 95:5:0.5) afforded the respective product.

General Procedure 6: Stille-Migita cross-coupling

A solution of aryl bromide/triflate (1 eq.) and vinyl stannane (1.2 eq.) in NMP was degassed with Ar. $Pd(Ph_3)_4$ (0.1 eq.) was added, and the mixture was stirred at the specified temperature until completion. The reaction mixture was allowed to cool to rt, diluted with CH_2Cl_2, and washed 3 times with H_2O, then brine. The organic layer was dried over Na_2SO_4, filtered, and concentrated.

General Procedure 7: Sonogashira cross-coupling in *N*,*N*-diisopropylamine

Indoline (1 eq.), $PdCl_2(PPh_3)_2$ (0.05 eq.), and CuI (0.05 eq.) were degassed 3 times in a Schlenk tube with Ar. A degassed solution of alkyne (1.2 eq.) in *N*,*N*-diisopropylamine was added, and the reaction mixture was stirred at the specified temperature until completion. The reaction mixture was allowed to cool to rt, filtered through Celite™ and eluted with EtOAc. The filtrate was washed 3 times with H_2O, then brine, dried over Na_2SO_4, filtered, and concentrated.

General Procedure 8: Hydrogenation and hydrogenolysis over Pd/C

Pd/C (wet degussa type, 10% w/w substrate) was added to a solution of the substrate in the specified solvent. The suspension was first degassed 3 times with N_2 using a pump-flood procedure and then flushed 3 times with H_2 using a pump-flood procedure. The reaction mixture was stirred under a H_2 atmosphere until completion and flushed with N_2. The suspension was filtered through Celite™, eluted with EtOAc, and concentrated.

2.2 Experimental Procedures

2-Phenylnitrile nitrobenzene **19**

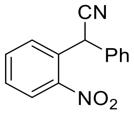

Nitrile **19** was prepared according to a modified literature procedure.[3] NaOH (aq., 50% w/w, 12 mL, 150 mmol) was added to a suspension of 1-fluoro-2-nitrobenzene (3.2 mL, 30 mmol), benzyl cyanide (3.5 mL, 30 mmol), and tetrabutylammonium bisulfate (10.2 g, 30.0 mmol) in toluene (80 mL). The mixture was stirred at 0 °C for 60 min. HCl (aq., 1 M, 150 mL) was added, and the mixture was extracted with CH_2Cl_2. The combined organic extracts were washed with brine, dried over $MgSO_4$, filtered, and concentrated. Purification by flash pressure column chromatography (10% EtOAc/petroleum ether) afforded nitrile **19** as a yellow solid (3.34 g, 14.0 mmol, 47%).

^{1}H NMR (400 MHz, $CDCl_3$): δ 6.18 (s, 1H), 7.30-7.41 (m, 5H), 7.56 (ddd, *J* = 8.3 Hz, *J* = 7.4 Hz, *J* = 1.3 Hz, 1H), 7.68-7.77 (m, 2H), 8.08 (dd, *J* = 8.3 Hz, *J* = 1.3 Hz, 1H).

^{13}C NMR (100.6 MHz, $CDCl_3$): δ 38.3, 118.6, 125.8, 127.9, 128.7, 129.3, 129.7, 130.5, 130.9, 134.1, 134.1, 147.6.

HRMS (ES^+): $C_{14}H_{10}N_2NaO_2^+$ requires 261.0634; found 261.0640.

MP: 47-48 °C.

2-Phenylnitrile aniline **20**

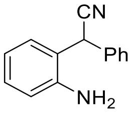

Aniline **20** was prepared according to a modified literature procedure.[4] Zinc powder (8.24 g, 126 mmol) and NH_4Cl (10.1 g, 189 mmol) were added to a solution of nitrile **19** (3.00 g, 12.6 mmol) in 5:1 acetone/H_2O (240 mL). The mixture was stirred at rt for 20 min, then filtered through Celite™, eluted with EtOAc, and washed with water, then brine. The organic layer was dried over $MgSO_4$, filtered, and concentrated. Purification by flash pressure column chromatography (13% EtOAc/petroleum ether) afforded aniline **20** as an orange solid (2.16 g, 10.4 mmol, 82%).

^{1}H NMR (400 MHz, $CDCl_3$): δ 3.54 (br. S, 2H), 5.19 (s, 1H), 6.74 (dd, *J* = 7.8 Hz, 1.2 Hz, 1H), 6.89 (td, *J* = 7.8 Hz, 1.2 Hz, 1H), 7.21 (td, *J* = 7.8 Hz, 1.2 Hz, 1H), 7.30-7.44 (m, 6H).

^{13}C NMR (100.6 MHz, $CDCl_3$): δ 39.0, 117.5, 118.9, 119.5, 120.0, 127.7, 128.5, 129.4, 129.5, 129.6, 133.9, 143.8.

HRMS (ES^+): $C_{14}H_{12}N_2Na^+$ requires 231.0893; found 231.0901.

MP: 72-73 °C.

3-((Tert-butyldimethylsilyl)oxy)benzaldehyde **21**

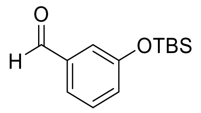

Aldehyde **21** was prepared according to a literature procedure.[5] To a solution of 3-hydroxybenzaldehyde (5.00 g, 40.9 mmol) in acetonitrile (10 mL) was added K_2CO_3 (11.3 g, 81.9 mmol) followed by TBSCl (7.40 g, 49.1 mmol). The reaction was stirred at room temperature for 24 hours before EtOAc was added and the organic layer washed with water and brine. After drying over $MgSO_4$ and evaporation under reduced pressure, purification by column chromatography (SiO_2, EtOAc/PE 1:19) afforded the title compound as a colourless liquid (6.86 g, 29.2 mmol, 71% yield). The spectral data match those reported in the literature.

^{1}H NMR (400 MHz, $CDCl_3$): δ 0.23 (s, 6H), 1.00 (s, 9H), 7.11 (ddd, *J* = 8.0 Hz, *J* = 2.5 Hz, *J* = 1.2 Hz, 1H), 7.33 (dd, *J* = 2.4 Hz, *J* = 1.6 Hz, 1H), 7.40 (t, *J* = 7.8 Hz, 1H), 7.48 (dt, *J* = 7.6 Hz, *J* = 1.3 Hz, 1H), 9.96 (s, 1H).

^{13}C NMR (100.6 MHz, $CDCl_3$): δ -4.4, 18.2, 25.6, 119.9, 123.6, 126.6, 130.1, 137.9, 156.4, 192.2.

(*E*)-2-(2-((3-((Tert-butyldimethylsilyl)oxy)benzylidene)amino)phenyl)-2-phenylacetonitrile **22**

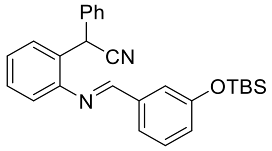

To a solution of 3-((tert-butyldimethylsilyl)oxy)benzaldehyde **21** (2.62 g, 11.0 mmol) in toluene (50 mL) was added 2-(2-aminophenyl)-2-phenylacetonitrile **20** (2.07 g, 10.0 mmol) and 3 Å MS (10 g). The reaction was stirred at 100 °C for 72 hours before filtering through celite (EtOAc) and evaporating under reduced pressure.

Purification by column chromatography (SiO_2, EtOAc/PE 1:19) afforded the title compound as a colourless oil (1.95 g, 4.58 mmol, 46% yield).

^{1}H NMR (400 MHz, $CDCl_3$): δ 0.25 (s, 6H), 1.02 (s, 9H), 5.95 (s, 1H), 7.01 (ddd, *J* = 8.0 Hz, *J* = 2.5 Hz, *J* = 1.0 Hz, 1H), 7.07 (dd, *J* = 7.9 Hz, *J* = 1.2 Hz, 1H), 7.22-7.31 (m, 1H), 7.32-7.43 (m, 5H), 7.44-7.48 (m, 1H), 7.52 (dd, *J* = 7.7 Hz, *J* = 1.4 Hz, 1H), 8.33 (s, 1H).

^{13}C NMR (100.6 MHz, $CDCl_3$): δ -4.3, 18.3, 25.7, 37.4, 118.1, 119.6, 120.3, 122.7, 123.9, 126.9, 127.8, 127.8, 128.4, 128.9, 129.5, 129.9, 131.0, 136.3, 137.4, 148.7, 156.2, 160.7.

HRMS (ES$^+$): $[C_{27}H_{31}ON_2Si]^+$ $(M+H)^+$ requires 427.2211; found 427.2194.

(2*RS*,3*RS*)-2-(3-((Tert-butyldimethylsilyl)oxy)phenyl)-3-phenylindoline-3-carbonitrile **23** and (2*RS*,3*SR*)-2-(3-((tert-butyldimethylsilyl)oxy)phenyl)-3-phenylindoline-3-carbonitrile **24**

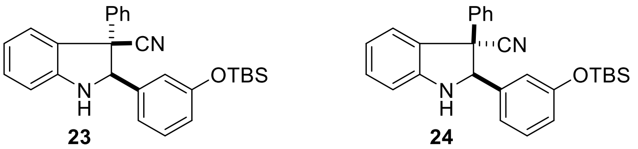

23 **24**

To a solution of 2-(2-((3-((tert-butyldimethylsilyl)oxy)benzylidene)amino)phenyl)-2-phenylacetonitrile **22** (1.30 g, 3.05 mmol) in PhMe (100 mL) at 0 °C was added ^{t}BuOK (674 mg, 6.10 mmol). The reaction was stirred for 30 minutes before quenching with NH_4Cl and extracting with EtOAc. The combined organic layers were washed with brine, dried over $MgSO_4$ and evaporated under reduced pressure. Purification by column chromatography (SiO_2, EtOAc/PE 1:19) afforded the title compounds as a colourless liquid (1.03 g, 2.42 mmol, 81% yield, 9:1 d.r.).

N.B. Stereochemistry assigned by analogy to previously reported similar indolines.[6]

(2*RS*,3*RS*)-2-(3-((Tert-butyldimethylsilyl)oxy)phenyl)-3-phenylindoline-3-carbonitrile **23**:

^{1}H NMR (400 MHz, $CDCl_3$): δ 0.13 (s, 3H), 0.15 (s, 3H), 0.95 (s, 9H), 4.29 (br. s, N*H*, 1H), 4.99 (s, 1H), 6.74 (t, *J* = 1.9 Hz, 1H), 6.83-6.90 (m, 4H), 7.02-7.05 (m, 1H), 7.21 (t, *J* = 7.9 Hz, 1H), 7.26 (td, *J* = 7.8 Hz, *J* = 1.2 Hz, 1H), 7.38-7.47 (m, 5H).

^{13}C NMR (100.6 MHz, $CDCl_3$): δ -4.4, -4.4, 18.2, 25.7, 58.3, 77.4, 110.4, 118.8, 119.1, 120.5, 120.5, 120.9, 125.8, 127.4, 127.7, 128.4, 128.8, 129.5, 130.2, 137.8, 138.0, 150.3, 155.7

HRMS (ES+): $[C_{27}H_{30}N_2NaOSi]^+$ $(M+Na)^+$ requires 449.2020; found 449.2025.

(2*RS*,3*SR*)-2-(3-((Tert-butyldimethylsilyl)oxy)phenyl)-3-phenylindoline-3-carbonitrile **24:**

^{1}H NMR (400 MHz, $CDCl_3$): δ 0.08 (s, 3H), 0.10 (s, 3H), 0.94 (s, 9H), 4.19 (d, *J* = 2.7 Hz, 1H), 5.51 (d, *J* = 2.8 Hz, 1H), 6.61-6.66 (m, 2H), 6.75-6.80 (m, 3H), 6.87-6.96 (m, 2H), 6.96-7.02 (m, 1H), 7.03-7.10 (m, 3H), 7.27-7.34 (m, 2H).

^{13}C NMR (100.6 MHz, $CDCl_3$): δ -4.4, -4.4, 18.1, 25.7, 55.2, 74.3, 110.4, 119.1, 120.1, 120.5, 120.7, 121.8, 125.6, 127.8, 127.9, 128.0, 128.3, 129.0, 130.2, 134.3, 137.2, 150.1, 155.5.

HRMS (ES+): $[C_{27}H_{30}N_2NaOSi]^+$ $(M+Na)^+$ requires 449.2020; found 449.2027.

(2*RS*,3*RS*)-1-Acetyl-2-(3-((tert-butyldimethylsilyl)oxy)phenyl)-3-phenylindoline-3-carbonitrile **25**

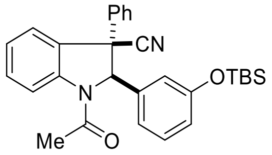

To a solution of 2-(3-((tert-butyldimethylsilyl)oxy)phenyl)-3-phenylindoline-3-carbonitrile **23** (1.00 g, 2.35 mmol) in CH_2Cl_2 (40 mL) at 0 °C was added pyridine (240 µL, 2.94 mmol), and AcCl (200 mL, 2.82 mmol). The reaction was stirred for 15 minutes before quenching with $NaHCO_3$ and extracting into DCM. The combined DCM layers were washed with brine, dried over $MgSO_4$ and evaporated under reduced pressure. Purification by column chromatography (SiO_2, EtOAc/PE 1:9) afforded the title compound as a colourless liquid which slowly crystallized into a white solid (1.03 g, 2.19 mmol, 93% yield).

^{1}H NMR (400 MHz, $CDCl_3$): δ 0.01 (s, 3H), 0.03 (s, 3H), 0.84 (s, 9H), 1.88 (s, 3H), 5.13 (s, 1H), 6.55 (t, *J* = 1.8 Hz, 1H), 6.75-6.82 (m, 2H), 7.11-7.22 (m, 4H), 7.23-7.27 (m, 1H), 7.27-7.34 (m, 3H), 7.41 (ddd, *J* = 8.8 Hz, *J* = 7.5 Hz, *J* = 1.4 Hz, 1H), 8.33 (br. s, 1H).

^{13}C NMR (125.75 MHz, $CDCl_3$): δ -4.5, -4.5, 18.2, 24.1, 25.7, 57.6, 76.3, 117.2, 117.6, 118.4, 119.4, 121.7, 125.5, 125.6, 125.7, 128.2, 128.9, 129.6, 130.6, 130.7, 138.7, 140.3, 143.1, 156.6, 169.0.

HRMS (ESI+, m/z): $[C_{29}H_{32}N_2NaO_2Si]^+$ $(M+Na)^+$ calc. 491.2125, found 491.2131.

(2*RS*,3*RS*)-1-acetyl-2-(3-hydroxyphenyl)-3-phenylindoline-3-carbonitrile **26**

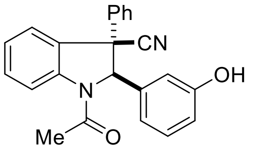

To a solution of 2-(3-((tert-butyldimethylsilyl)oxy)phenyl)-3-phenylindoline-3-carbonitrile **25** (1.00 g, 2.14 mmol) in THF (20 mL) at room temperature was added TBAF (1 M in THF, 4.3 mL, 4.3 mmol). The reaction was stirred for 10 minutes before being diluted with EtOAc (50 mL) and acidified with 1 M HCl. The aqueous layer was discarded and the organic layer dried over $MgSO_4$ and evaporated under reduced pressure. Purification by column chromatography (SiO_2, EtOAc/PE 1:3) afforded the title compound as a colourless liquid which slowly crystallized into a white solid (562 mg, 1.71 mmol, 80% yield).

^{1}H NMR (500 MHz, $CDCl_3$): δ 1.98 (br. s, 3H), 5.27 (br. s, 1H), 5.69 (br. s, 1H), 6.70 (t, *J* = 1.9 Hz, 1H), 6.84 (d, *J* = 7.7 Hz, 1H), 6.88 (dd, *J* = 8.0 Hz, *J* = 1.9 Hz, 1H), 7.24-7.29 (m, 3H), 7.31 (t, *J* = 7.9 Hz, 1H), 7.33-7.38 (m, 1H), 7.39-7.45 (m, 3H), 7.48-7.53 (m, 1H), 8.42 (br. s, 1H).

^{13}C NMR (125.75 MHz, $CDCl_3$): δ 24.1, 57.6, 76.3, 112.9, 116.9, 117.3, 118.5, 118.7, 125.5, 125.6, 125.8, 128.7, 129.0, 129.6, 130.8, 130.8, 139.0, 140.3, 142.9, 156.7, 169.4.

HRMS (ESI$^+$, m/z): $[C_{23}H_{18}N_2NaO_2]^+$ $(M+Na)^+$ calc. 377.1260, found 377.1261.

Methyl 6-hydroxyhexanoate **27**

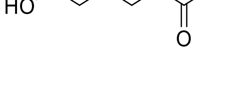

Ester **27** was prepared according to a literature procedure.[7] To a solution of ε-caprolactone (10.0 g, 87.6 mmol) in dry MeOH (100 mL) was added H_2SO_4 (0.8 mL) and the reaction was stirred at room temperature for 48 hours. The reaction was concentrated under reduced pressure and diluted with ether before washing with water, $NaHCO_3$ and brine. The ether layer was dried over $MgSO_4$ and evaporated under reduced pressure to

afford the title compound which did not require further purification. The spectral data match those reported in the literature.

^{1}H NMR (400 MHz, CDCl$_3$): δ 1.36-1.46 (2H, m), 1.55-1.64 (2H, m), 1.64-1.72 (2H, m), 1.67 (1H br. s, O*H*), 2.34 (2H, t, *J* = 7.5 Hz), 3.66 (2H, t, *J* = 6.6 Hz), 3.68 (3H, s).

^{13}C NMR (100.6 MHz, CDCl$_3$): δ 24.6, 25.3, 32.3, 34.0, 51.5, 62.6, 174.2.

Methyl 6-oxohexanoate **28**

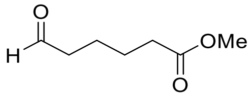

Aldehyde **28** was prepared according to a literature procedure.[8] To a solution of PCC (8.20 g, 38.0 mmol) in DCM (70 mL) was added methyl 6-hydroxyhexanoate **27** (5.11 g, 35.0 mmol) in DCM (10 mL). The reaction was stirred at room temperature for 2 hours before ether was added. The solution was decanted and the black residue washed several times with ether. Purification by column chromatography (SiO_2, Et_2O/PE 1:3) afforded the title compound as a colourless liquid (2.99 g, 20.7 mmol, 59% yield). The spectral data match those reported in the literature.

^{1}H NMR (400 MHz, CDCl$_3$): δ 1.62-1.68 (m, 4H), 2.29-2.36 (m, 2H), 2.43-2.49 (m, 2H), 3.65 (s, 3H), 9.75 (t, *J* = 1.6 Hz, 1H).

^{13}C NMR (100.6 MHz, CDCl$_3$): δ 21.5, 24.3, 33.7, 43.5, 51.6, 173.7, 202.0.

Methyl 6-cyclopentylidenehexanoate **29**

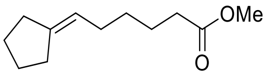

To a solution of cyclopentyltriphenylphosphonium bromide (10.0 g, 24.3 mmol) in THF (100 mL) at 0 °C was slowly added ^{n}BuLi (1.6 M sln hexanes, 15.2 mL, 24.3 mmol). The reaction was stirred at room temperature for 1 hour before cooling to 0 °C. A solution of methyl 6-oxohexanoate **28** (2.70 g, 18.7 mmol) in THF (5 mL) was added and the reaction was stirred for 24 hours before quenching with NH_4Cl and extracting into EtOAc. The

organic phase was washed with brine, dried over $MgSO_4$ and evaporated under reduced pressure. Purification by column chromatography (SiO_2, Et_2O/PE 1:49) afforded the title compound as a colourless liquid (2.92 g, 14.9 mmol, 80% yield).

^{1}H NMR (400 MHz, CDCl$_3$): δ 1.33-1.42 (m, 2H), 1.54-1.70 (m, 6H), 1.94-2.02 (m, 2H), 2.12-2.18 (m, 2H), 2.18-2.25 (m, 2H), 2.31 (t, *J* = 7.6 Hz, 2H), 3.67 (s, 3H), 5.18-5.25 (m, 1H).

^{13}C NMR (100.6 MHz, CDCl$_3$): δ = 24.7, 26.3, 26.4, 28.6, 29.2, 29.2, 33.6, 34.1, 51.4, 119.6, 143.5, 174.3.

HRMS (ESI$^+$, m/z): $[C_{12}H_{20}NaO_2]^+$ $(M+Na)^+$ calc. 219.1356, found 219.1359.

Methyl 6-cyclopentylhexanoate **30**

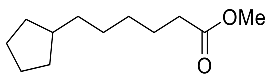

To a solution of methyl 6-cyclopentylidenehexanoate **29** (2.90 g, 14.8 mmol) in EtOAc (100 mL) at room temperature was added 10% Pd/C (314 mg). The reaction was stirred under an atmosphere of H_2 for 20 hours before filtering through Celite™ (eluting with EtOAc) and evaporating under reduced pressure to afford the title compound as a colourless liquid (2.91 g, 99% yield).

^{1}H NMR (400 MHz, CDCl$_3$): δ 1.01-1.13 (m, 2H), 1.26-1.37 (m, 6H), 1.45-1.69 (m, 6H), 1.69-1.81 (m, 3H), 2.32 (t, *J* = 7.5 Hz, 2H), 3.69 (s, 3H).

^{13}C NMR (100.6 MHz, CDCl$_3$): δ 25.0, 25.2, 28.4, 29.4, 32.7, 34.1, 36.0, 40.1, 51.4, 174.4.

HRMS (ESI$^+$, m/z): $[C_{12}H_{22}NaO_2]^+$ $(M+Na)^+$ calc. 221.1512, found 221.1522.

(2*RS*,3*RS*)-1-Acetyl-2-(3-((6-cyclopentylhexyl)oxy)phenyl)-3-phenylindoline-3-carbonitrile **32**

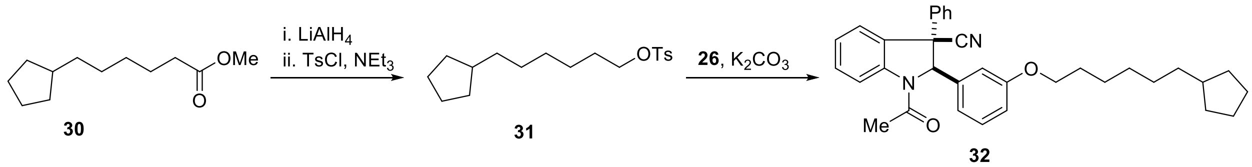

To a solution of methyl 6-cyclopentylhexanoate **30** (2.90 g, 14.8 mmol) in THF (100 mL) at 0 °C was added $LiAlH_4$ (1.69 g, 44.4 mmol) and the reaction was warmed to room temperature after 10 minutes and stirred for 2 hours before cooling to 0 °C and quenching with $Na_2SO_4.10H_2O$ and filtering through Celite™ and then silica

(eluting with EtOAc). After concentrating *in vacuo* the residue was dissolved in DCM (50 mL), and Et_3N (2.80 mL, 20.0 mmol) and TsCl (2.86 g, 15.0 mmol) were added. The reaction was stirred for 24 hours before quenching with $NaHCO_3$. The organic layer was further washed with brine, dried over $MgSO_4$ and evaporated under reduced pressure. Purification by column chromatography (SiO_2, PhMe/PE 4:1) afforded 6-cyclopentylhexyl 4-methylbenzenesulfonate **31** as a colourless liquid (182 mg, 0.56 mmol, 4% yield), which was used directly in the subsequent step.

To a solution of *syn*-1-acetyl-2-(3-hydroxyphenyl)-3-phenylindoline-3-carbonitrile **26** (125 mg, 0.38 mmol) in acetone (5 mL, pre-dried over 3 ÅMS) was added K_2CO_3 (266 mg, 1.91 mmol) and 6-cyclopentylhexyl 4-methylbenzenesulfonate **31** (135 mg, 0.42 mmol). The reaction was stirred at reflux overnight before cooling to room temperature and concentrating *in vacuo*. The residue was dissolved in EtOAc, washed with water and brine, dried over $MgSO_4$ and evaporated under reduced pressure. Purification by column chromatography (SiO_2, EtOAc/PE 1:9) afforded the title compound as a colourless oil (59 mg, 0.12 mmol, 31% yield).

^{1}H NMR (400 MHz, $CDCl_3$): δ 1.01-1.14 (m, 2H), 1.28-1.38 (m, 6H), 1.38-1.67 (m, 6H), 1.69-1.81 (m, 5H), 1.97 (br. s, 3H), 3.91 (t, *J* = 6.6 Hz, 2H), 5.27 (br. s, 1H), 6.75 (t, *J* = 1.6 Hz, 1H), 6.80 (d, *J* = 7.6 Hz, 1H), 6.92 (dd, *J* = 8.2 Hz, *J* = 2.1 Hz, 1H), 7.23 (td, *J* = 7.6 Hz, *J* = 0.9 Hz, 1H), 7.25-7.29 (m, 2H), 7.29-7.35 (m, 2H), 7.37-7.43 (m, 3H), 7.48-7.53 (m, 1H), 8.42 (br. s, 1H).

^{13}C NMR (125.75 MHz, $CDCl_3$): δ 24.1, 25.2, 26.1, 28.7, 29.2, 29.7, 32.8, 36.2, 40.2, 57.6, 68.1, 76.5, 113.0, 115.0, 117.3, 118.2, 118.5, 125.4, 125.6, 125.7, 125.8, 128.9, 129.6, 130.6, 130.7, 138.8, 140.5, 143.1, 159.8, 169.2.

HRMS (ESI$^+$, m/z): $[C_{34}H_{39}O_2N_2]^+$ $(M+Na)^+$ calc. 507.3006, found 507.3000.

(2*RS*,3*RS*)-1-Benzoyl-2-(3-((tert-butyldimethylsilyl)oxy)phenyl)-3-phenylindoline-3-carbonitrile **34**

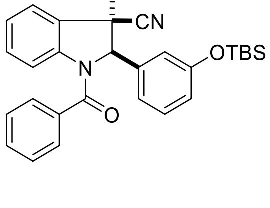

Prepared according to general procedure 2 using indoline **23** (1 eq.), benzoyl chloride (2 eq.), and pyridine (2 eq.) in CH_2Cl_2 (0.2 M). Purification by flash pressure column chromatography (SiO_2, EtOAc/PE 1:19) afforded the title compound as a white solid (59% yield).

^{1}H NMR (500 MHz, CDCl$_3$): δ 0.06 (s, 3H), 0.07 (s, 3H), 0.91 (s, 9H), 5.30 (s, 1H), 6.50 (s, 1H), 6.66 (br. s, 1H), 6.83 (ddd, *J* = 8.2 Hz, *J* = 2.4 Hz, *J* = 0.9 Hz, 1H), 7.05-7.21 (m, 3H), 7.21-7.32 (m, 5H), 7.35-7.50 (m, 6H), 7.89 (br. s, 1H).

^{13}C NMR (125.75 MHz, CDCl$_3$): δ -4.5, -4.5, 18.2, 25.7, 57.1, 117.4, 117.6, 118.5, 119.7, 121.3, 125.7, 125.8, 125.9, 126.9, 128.5, 128.9, 129.5, 129.5, 130.0, 130.1, 130.4, 130.7, 135.6, 138.8, 139.5, 143.1, 156.2, 169.6.

HRMS (ESI$^+$, m/z): $[C_{32}H_{39}O_2N_2Si]^+$ $(M+H)^+$ calc. 511.2786, found 511.2769.

(2*RS*,3*RS*)-2-(3-((Tert-butyldimethylsilyl)oxy)phenyl)-1-(3-methylbutanoyl)-3-phenylindoline-3-carbonitrile **35**

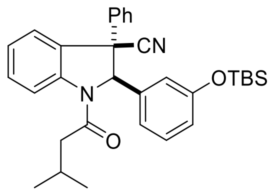

Prepared according to general procedure 2 using indoline **23** (1 eq.), isovaleryl chloride (2 eq.), and pyridine (2 eq.) in CH_2Cl_2 (0.2 M). Purification by flash pressure column chromatography (SiO_2, EtOAc/PE 1:19) afforded the title compound as a white solid (88% yield).

^{1}H NMR (400 MHz, CDCl$_3$): δ 0.09 (s, 3H), 0.10 (s, 3H), 0.70 (d, *J* = 6.4 Hz, 3H), 0.79 (d, *J* = 6.4 Hz, 3H), 0.93 (s, 9H), 1.83-2.21 (m, 3H), 5.27 (br. s, 1H), 6.64 (t, *J* = 1.9 Hz, 1H), 6.84-6.90 (m, 2H), 7.20-7.27 (m, 3H), 7.29 (d, *J* = 8.0 Hz, 1H), 7.32-7.43 (m, 4H), 7.47-7.53 (m, 1H), 8.44 (br. s, 1H).

^{13}C NMR (125.75 MHz, CDCl$_3$): δ -4.5, 18.2, 22.2, 22.4, 25.2, 25.7, 27.8, 44.3, 57.5, 75.7, 117.3, 117.7, 118.5, 119.5, 121.7, 125.4, 125.5, 125.7, 128.8, 128.9, 129.5, 130.5, 130.7, 138.9, 140.2, 143.3, 156.5, 171.3.

HRMS (ESI$^+$, m/z): $[C_{32}H_{39}O_2N_2Si]^+$ $(M+H)^+$ calc. 511.2786, found 511.2769.

(2*RS*,3*RS*)-2-(3-((Tert-butyldimethylsilyl)oxy)phenyl)-1-(furan-2-carbonyl)-3-phenylindoline-3-carbonitrile **36**

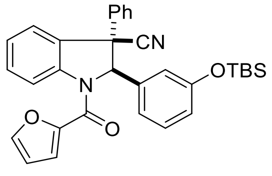

Prepared according to general procedure 2 using indoline **23** (1 eq.), 2-furoyl chloride (2 eq.), and pyridine (2 eq.) in CH_2Cl_2 (0.2 M). Purification by flash pressure column chromatography (SiO_2, EtOAc/PE 1:9) afforded the title compound as a white solid (76% yield).

^{1}H NMR (400 MHz, $CDCl_3$): δ 0.06 (s, 3H), 0.08 (s, 3H), 0.91 (s, 9H), 6.01 (s, 1H), 6.36 (dd, *J* = 3.6 Hz, *J* = 1.8 Hz, 1H), 6.65 (t, *J* = 2.0 Hz, 1H), 6.81 (ddd, *J* = 8.2 Hz, *J* = 2.4 Hz, *J* = 0.9 Hz, 1H), 6.87 (d, *J* = 7.7 Hz, 1H), 7.01 (dd, *J* = 3.5 Hz, *J* = 0.8 Hz, 1H), 7.22 (t, *J* = 7.9 Hz, 1H), 7.27 (td, *J* = 7.7 Hz, *J* = 0.9 Hz, 1H), 7.31-7.35 (m, 2H), 7.35-7.43 (m, 5H), 7.50-7.56 (m, 1H), 8.37 (d, *J* = 7.6 Hz, 1H).

^{13}C NMR (100.6 MHz, $CDCl_3$): δ -4.5, 18.2, 25.7, 58.1, 75.6, 111.9, 117.6, 118.0, 118.4, 118.5, 119.5, 121.3, 125.7, 125.7, 126.0, 128.8, 129.4, 129.5, 130.2, 130.6, 139.5, 140.0, 143.6, 144.7, 147.1, 156.2, 157.8.

HRMS (ESI$^+$, m/z): $[C_{32}H_{33}O_3N_2Si]^+$ $(M+H)^+$ calc. 521.2250, found 521.2250.

(2*RS*,3*RS*)-2-(3-((Tert-butyldimethylsilyl)oxy)phenyl)-3-phenyl-1-(4-(trifluoromethyl)benzoyl)indoline-3-carbonitrile **37**

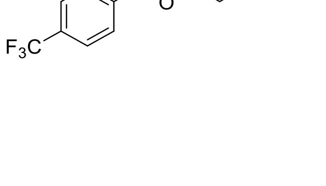

Prepared according to general procedure 2 using indoline **23** (1 eq.), 4-trifluoromethyl benzoyl chloride (2 eq.), and pyridine (2 eq.) in CH_2Cl_2 (0.2 M). Purification by flash pressure column chromatography (SiO_2, EtOAc/PE 1:19) afforded the title compound as a white solid (73% yield).

^{1}H NMR (500 MHz, CDCl$_3$): δ 0.06 (s, 3H), 0.07 (s, 3H), 0.91 (s, 9H), 5.15 (br. s, 1H), 6.46 (s, 1H), 6.62 (br. s, 1H), 6.84 (ddd, *J* = 8.1 Hz, *J* = 2.3 Hz, *J* = 0.7 Hz, 1H), 7.08-7.24 (m, 3H), 7.26-7.33 (m, 3H), 7.39-7.45 (m, 4H), 7.46-7.57 (m, 3H), 8.24 (br. s, 1H).

^{13}C NMR (125.75 MHz, CDCl$_3$): δ -4.6, -4.5, 18.2, 25.6, 57.2, 77.1, 117.6, 118.3, 119.5, 121.6, 123.6 (q, *J* = 272.1 Hz), 125.5, 125.6, 126.1, 126.3, 127.2, 128.3 (q, *J* = 6.3 Hz), 129.1, 129.6, 129.9, 130.4, 130.6, 132.4 (q, *J* = 32.6 Hz), 138.5, 139.0, 139.4, 142.7, 156.3, 168.1.

HRMS (ESI$^+$, m/z): $[C_{35}H_{34}O_2N_2Si]^+$ $(M+H)^+$ calc. 599.2347, found 599.2329.

Ethyl (2*RS*,3*RS*)-2-(3-((tert-butyldimethylsilyl)oxy)phenyl)-3-cyano-3-phenylindoline-1-carboxylate **38**

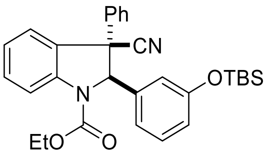

Prepared according to general procedure 2 using indoline **23** (1 eq.), ethyl chloroformate (2 eq.), and pyridine (2 eq.) in CH_2Cl_2 (0.2 M). Purification by flash pressure column chromatography (SiO_2, EtOAc/PE 1:19) afforded the title compound as a white solid (97% yield).

^{1}H NMR (400 MHz, CDCl$_3$): δ 0.09 (s, 3H), 0.11 (s, 3H), 0.93 (s, 9H), 1.08 (br. s, 3H), 4.01-4.19 (m, 2H), 5.33 (s, 1H), 6.63 (t, *J* = 2.0 Hz, 1H), 6.82-6.87 (m, 2H), 7.17 (td, *J* = 7.6 Hz, *J* = 1.0 Hz, 1H), 7.21-7.29 (m, 3H), 7.30-7.34 (m, 1H), 7.35-7.43 (m, 3H), 7.48 (ddd, *J* = 8.8 Hz, *J* = 7.5 Hz, *J* = 1.3 Hz, 1H), 8.02 (br. s, 1H).

^{13}C NMR (100.6 MHz, CDCl$_3$): δ -4.5, 14.1, 18.2, 25.7, 56.8, 62.1, 75.0, 115.1, 117.5, 118.7, 119.4, 121.1, 124.4, 125.8, 125.9, 128.7, 128.8, 129.4, 130.0, 130.7, 139.5, 140.6, 142.6, 152.5, 156.1.

HRMS (ESI$^+$, m/z): $[C_{30}H_{35}O_3N_2Si]^+$ $(M+H)^+$ calc. 499.2412, found 499.2399.

(2*RS*,3*RS*)-1-Benzoyl-2-(3-hydroxyphenyl)-3-phenylindoline-3-carbonitrile **39**

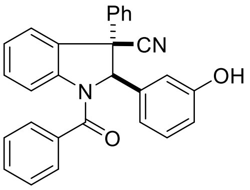

Prepared according to general procedure 3 to afford the title compound after column chromatography (SiO_2, EtOAc/PE 1:3) as a white solid (81% yield).

^{1}H NMR (700 MHz, $CDCl_3$): δ 5.30 (s, 1H), 5.33 (br. s, 1H), 6.52 (br. s, 1H), 6.61 (br. s, 1H), 6.76 (dd, *J* = 8.0 Hz, *J* = 2.4 Hz, 1H), 7.10-7.19 (m, 3H), 7.19-7.32 (m, 6H), 7.35-7.45 (m, 6H).

^{13}C NMR (125.75 MHz, $CDCl_3$): δ 57.0, 60.5, 113.3, 116.5, 117.2, 118.6, 118.9, 125.7, 125.8, 126.0, 126.9, 128.5, 129.0, 129.5, 129.9, 130.3, 130.4, 130.8, 135.5, 139.1, 139.5, 142.9, 156.1, 169.6.

HRMS (ESI$^+$, m/z): $[C_{28}H_{20}N_2NaO_2]^+$ $(M+Na)^+$ calc. 439.1417, found 439.1425.

(2*RS*,3*RS*)-2-(3-Hydroxyphenyl)-1-(3-methylbutanoyl)-3-phenylindoline-3-carbonitrile **40**

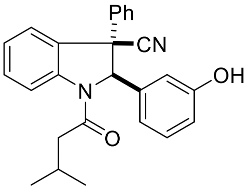

Prepared according to general procedure 3 to afford the title compound after column chromatography (SiO_2, EtOAc/PE 1:3) as a white solid (62% yield).

^{1}H NMR (500 MHz, $CDCl_3$): δ 0.73 (d, *J* = 5.7 Hz, 3H), 0.80 (d, *J* = 5.7 Hz, 3H), 1.82-2.22 (m, 3H), 5.32 (br. s, 1H), 5.71 (br. s, 1H), 6.70 (t, *J* = 1.9 Hz, 1H), 6.84 (d, *J* = 7.7 Hz, 1H), 6.87 (dd, *J* = 8.2 Hz, *J* = 2.1 Hz, 1H), 7.24 (td, *J* = 7.5 Hz, *J* = 1.0 Hz, 1H), 7.25-7.28 (m, 2H), 7.29-7.32 (m, 1H), 7.35 (d, *J* = 7.5 Hz, 1H), 7.38-7.44 (m, 3H), 7.49 (ddd, *J* = 8.2 Hz, *J* = 7.5 Hz, *J* = 1.3 Hz, 1H), 8.44 (br. s, 1H).

^{13}C NMR (125.75 MHz, $CDCl_3$): δ 22.2, 22.4, 25.3, 44.3, 75.7, 113.0, 116.8, 117.4, 118.6, 118.7, 125.5, 125.6, 125.7, 125.8, 128.9, 129.6, 130.7, 130.8, 139.1, 140.2, 143.0, 156.6, 171.6.[ǂ]

ǂ 1 peak obscured.

HRMS (ESI$^+$, m/z): $[C_{26}H_{25}O_2N_2]^+$ $(M+H)^+$ calc. 397.1911, found 397.1902.

(2*RS*,3*RS*)-1-(Furan-2-carbonyl)-2-(3-hydroxyphenyl)-3-phenylindoline-3-carbonitrile **41**

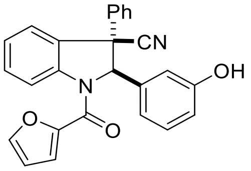

Prepared according to general procedure 3 to afford the title compound after column chromatography (SiO_2, EtOAc/PE 1:3) as a white solid (71% yield).

^{1}H NMR (500 MHz, $CDCl_3$): δ 5.43 (s, 1H), 6.05 (s, 1H), 6.38 (dd, *J* = 3.5 Hz, *J* = 1.7 Hz, 1H), 6.71 (t, *J* = 2.0 Hz, 1H), 6.79 (ddd, *J* = 8.1 Hz, *J* = 2.4 Hz, *J* = 0.7 Hz, 1H), 6.84 (d, *J* = 7.7 Hz, 1H), 7.03 (dd, *J* = 3.5 Hz, *J* = 0.7 Hz, 1H), 7.23 (t, *J* = 7.9 Hz, 1H), 7.28 (td, *J* = 7.6 Hz, *J* = 0.9 Hz, 1H), 7.32-7.44 (m, 7H), 7.51 (ddd, *J* = 8.8 Hz, *J* = 7.5 Hz, *J* = 1.3 Hz, 1H), 8.35 (br. s, 1H).

^{13}C NMR (125.75 MHz, $CDCl_3$): δ 58.0, 75.6, 112.0, 116.4, 118.0, 118.6, 118.6, 118.8, 125.7, 125.8, 126.1, 128.9, 129.2, 129.6, 130.4, 130.7, 139.8, 140.0, 143.3, 144.8, 146.9, 156.2, 157.9.ƚ

ƚ 1 peak obscured.

HRMS (ESI$^+$, m/z): $[C_{26}H_{18}N_2NaO_3]^+$ $(M+Na)^+$ calc. 429.1210, found 429.1219.

(2*RS*,3*RS*)-2-(3-Hydroxyphenyl)-3-phenyl-1-(4-(trifluoromethyl)benzoyl)indoline-3-carbonitrile **42**

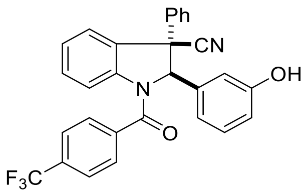

Prepared according to general procedure 3 to afford the title compound after column chromatography (SiO_2, EtOAc/PE 1:4) as a white solid (99% yield).

^{1}H NMR (400 MHz, $CDCl_3$): δ 5.24 (s, 1H), 5.32 (s, 1H), 6.53 (s, 1H), 6.59 (br. s, 1H), 6.80 (dd, *J* = 7.7 Hz, *J* = 2.0 Hz, 1H), 7.12-7.61 (m, 13H), 8.29 (br. s, 1H).

^{13}C NMR (100.6 MHz, $CDCl_3$): δ = 57.1, 77.1, 113.1, 116.7, 118.4, 123.5 (q, *J* = 273.3), 125.6, 126.1, 126.3, 127.2, 128.3, 129.1, 129.7, 129.7, 129.8, 130.5, 130.7, 132.5 (q, *J* = 33.5), 138.8, 138.9, 139.4, 142.5, 149.9, 156.3, 168.2.

HRMS (ESI$^+$, m/z): $[C_{29}H_{19}F_3N_2NaO_2]^+$ $(M+Na)^+$ calc. 507.1291, found 507.1301.

Ethyl (2*RS*,3*RS*)-3-cyano-2-(3-hydroxyphenyl)-3-phenylindoline-1-carboxylate **43**

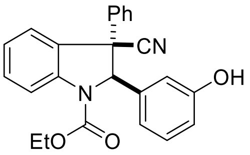

Prepared according to general procedure 3 to afford the title compound after column chromatography (SiO_2, EtOAc/PE 1:4) as a white solid (81% yield).

^{1}H NMR (400 MHz, $CDCl_3$): δ 1.12 (br. s, 3H), 4.04-4.26 (m, 2H), 5.38 (s, 1H), 5.51 (br. s, 1H), 6.69 (t, *J* = 2.2 Hz, 1H), 6.77-6.83 (m, 2H), 7.19 (td, *J* = 7.6 Hz, *J* = 1.0 Hz, 1H), 7.23 (t, *J* = 7.9 Hz, 1H), 7.26-7.32 (m, 2H), 7.32-7.35 (m, 1H), 7.38-7.45 (m, 3H), 7.46-7.51 (m, 1H), 8.02 (br. s, 1H).

^{13}C NMR (100.6 MHz, $CDCl_3$): δ 14.1, 56.8, 62.3, 75.1, 112.9, 115.2, 116.3, 118.5, 118.8, 124.5, 125.7, 126.0, 128.6, 128.7, 129.5, 130.3, 130.8, 139.8, 140.5, 142.4, 152.6, 156.2.

HRMS (ESI$^+$, m/z): $[C_{24}H_{21}O_3N_2]^+$ $(M+H)^+$ calc. 385.1558, found 385.1542.

(2*RS*,3*RS*)-1-Acetyl-2-(3-(4-bromobutoxy)phenyl)-3-phenylindoline-3-carbonitrile **44**

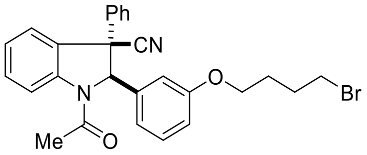

Prepared according to general procedure 4 to afford the title compound after column chromatography (SiO_2, EtOAc/PE 3:17) as a white solid (89% yield, contains minor impurities).

^{1}H NMR (700 MHz, $CDCl_3$): δ 1.91-2.10 (m, 7H), 3.50 (t, *J* = 6.5 Hz, 2H), 3.94-4.00 (m, 2H), 5.27 (br. s, 1H), 6.75 (s, 1H), 6.83 (d, *J* = 7.6 Hz, 1H), 6.93 (d, *J* = 7.4 Hz, 1H), 7.25 (t, *J* = 7.7 Hz, 1H), 7.27-7.29 (m, 2H), 7.32-7.37 (m, 2H), 7.38-7.45 (m, 3H), 7.52 (t, *J* = 7.8 Hz, 1H), 8.45 (br. s, 1H).

^{13}C NMR (176.05 MHz, $CDCl_3$): δ 24.2, 27.8, 29.4, 33.4, 57.6, 66.9, 76.4, 112.9, 115.0, 117.3, 118.5, 118.6, 125.5, 125.6, 125.7, 128.9, 129.6, 130.7, 130.8, 138.9, 140.4, 143.0, 143.0, 159.5, 169.1.

HRMS (ESI$^+$, m/z): $[C_{27}H_{25}{}^{79}BrN_2NaO_2]^+$ $(M+Na)^+$ calc. 511.0992, found 511.0999.

(2*RS*,3*RS*)-1-Acetyl-2-(3-((5-bromopentyl)oxy)phenyl)-3-phenylindoline-3-carbonitrile **45**

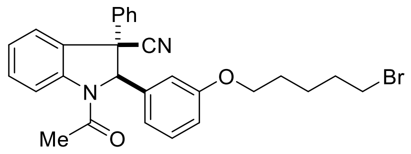

Prepared according to general procedure 4 to afford the title compound after column chromatography (SiO_2, EtOAc/PE 3:17) as a white solid (94% yield).

^{1}H NMR (500 MHz, $CDCl_3$): δ 1.59-1.67 (m, 2H), 1.78-1.84 (m, 2H), 1.95 (tt, *J* = 7.6 Hz, *J* = 6.9 Hz, 2H), 1.99 (br. s, 3H, *Ac*), 3.46 (t, *J* = 6.8 Hz, 2H), 3.95 (td, *J* = 6.2 Hz, *J* = 2.3 Hz, 2H), 5.28 (br. s, 1H), 6.75 (s, 1H), 6.83 (d, *J* = 7.6 Hz, 1H), 6.93 (dd, *J* = 8.2 Hz, *J* = 2.1 Hz, 1H), 7.25 (td, *J* = 7.6 Hz, *J* = 0.9 Hz, 1H), 7.27-7.28 (m, 1H), 7.29-7.30 (m, 1H), 7.32-7.37 (m, 2H), 7.39-7.45 (m, 3H), 7.52 (ddd, *J* = 8.2 Hz, *J* = 7.5 Hz, *J* = 1.3 Hz, 1H), 8.44 (br. s, 1H).

^{13}C NMR (125.75 MHz, $CDCl_3$): δ 24.2, 24.8, 28.3, 29.3, 32.5, 33.6, 57.6, 67.6, 112.9, 115.1, 117.3, 118.5, 118.5, 125.5, 125.6, 125.8, 125.8, 125.9, 129.6, 130.7, 130.7, 138.8, 140.4, 143.0, 159.6, 169.1.

HRMS (ESI$^+$, m/z): $[C_{28}H_{28}O_2N_2{}^{79}Br]^+$ $(M+H)^+$ calc. 503.1329, found 503.1324.

(2*RS*,3*RS*)-1-Acetyl-2-(3-((6-bromohexyl)oxy)phenyl)-3-phenylindoline-3-carbonitrile **46**

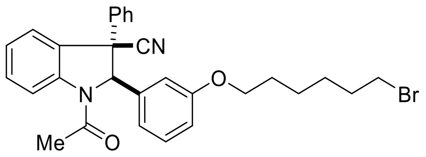

Prepared according to general procedure 4 to afford the title compound after column chromatography (SiO_2, EtOAc/PE 3:17) as a white solid (85% yield, contains minor impurities).

^{1}H NMR (500 MHz, $CDCl_3$): δ 1.45-1.54 (m, 4H), 1.74-1.82 (m, 2H), 1.86-1.93 (m, 2H), 1.97 (br. s, 3H), 3.43 (t, J = 6.8 Hz, 2H), 3.88-3.96 (m, 2H), 5.26 (br. s, 1H), 6.74 (br. s, 1H), 6.81 (d, J = 7.7 Hz, 1H), 6.92 (dd, J = 8.4 Hz, J = 2.3 Hz, 1H), 7.23 (td, J = 7.5 Hz, J = 0.8 Hz, 1H), 7.25-7.28 (m, 2H), 7.30-7.35 (m, 2H), 7.37-7.43 (m, 3H), 7.51 (ddd, J = 8.2 Hz, J = 7.7 Hz, J = 1.4 Hz, 1H), 8.42 (br. s, 1H).

^{13}C NMR (125.75 MHz, $CDCl_3$): δ 24.2, 25.3, 27.9, 29.0, 32.7, 33.8, 57.6, 67.8, 76.5, 112.9, 115.0, 117.3, 118.4, 118.5, 125.5, 125.6, 125.8, 128.2, 128.9, 129.6, 130.6, 130.7, 138.8, 140.4, 143.0, 159.7, 169.1.

HRMS (ESI$^+$, m/z): $[C_{29}H_{29}{}^{79}BrN_2NaO_2]^+$ $(M+Na)^+$ calc. 539.1305, found 539.1311.

(2*RS*,3*RS*)-1-Acetyl-2-(3-((7-bromoheptyl)oxy)phenyl)-3-phenylindoline-3-carbonitrile **47**

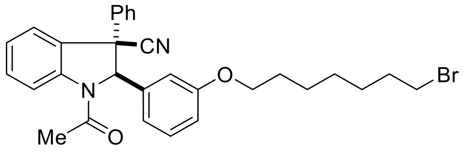

Prepared according to general procedure 4 to afford the title compound after column chromatography (SiO_2, EtOAc/PE 3:17) as a white solid (89% yield).

^{1}H NMR (500 MHz, $CDCl_3$): δ 1.34-1.42 (2H, m), 1.42-1.52 (4H, m), 1.76 (2H, quin, J = 6.9 Hz), 1.88 (2H, quin, J = 6.9 Hz), 1.97 (3H, br. s), 3.43 (2H, t, J = 6.8 Hz), 3.91 (2H, m), 5.26 (1H, br. s), 6.74 (1H, s), 6.81 (1H, d, J = 7.7 Hz), 6.92 (1H, dd, J = 8.2, 2.0 Hz), 7.20-7.29 (3H, m), 7.30-7.35 (2H, m), 7.37-7.44 (3H, m), 7.51 (1H, m), 8.43 (1H, br. s).

^{13}C NMR (125.75 MHz, $CDCl_3$): δ 24.2, 25.9, 28.1, 28.5, 29.1, 32.7, 34.0, 57.6, 67.9, 76.5, 112.9, 115.0, 117.3, 118.3, 118.5, 125.5, 125.6, 125.8, 128.7, 128.9, 129.6, 130.6, 130.7, 138.8, 140.4, 143.0, 159.7, 169.1.

HRMS (ESI$^+$, m/z): $[C_{30}H_{31}{}^{79}BrN_2NaO_2]^+$ $(M+Na)^+$ calc. 553.1461, found 553.1469.

(2*RS*,3*RS*)-1-Acetyl-2-(3-((8-bromooctyl)oxy)phenyl)-3-phenylindoline-3-carbonitrile **48**

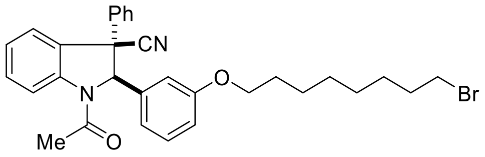

Prepared according to general procedure 4 to afford the title compound after column chromatography (SiO_2, EtOAc/PE 3:17) as a white solid (86% yield).

^{1}H NMR (500 MHz, $CDCl_3$): δ 1.33-1.42 (m, 4H), 1.42-1.52 (m, 4H), 1.77 (tt, *J* = 7.4 Hz, *J* = 6.9 Hz, 2H), 1.89 (tt, *J* = 7.4 Hz, *J* = 6.9 Hz, 2H), 1.99 (br. s, 3H, *Ac*), 3.44 (t, *J* = 6.9 Hz, 2H), 3.93 (td, *J* = 6.5 Hz, *J* = 2.1 Hz, 2H), 5.27 (br. s, 1H), 6.76 (s, 1H), 6.82 (d, *J* = 7.7 Hz, 1H), 6.94 (dd, *J* = 8.2 Hz, *J* = 2.1 Hz, 1H), 7.25 (td, *J* = 7.6 Hz, *J* = 0.7 Hz, 1H), 7.27-7.30 (m, 2H), 7.31-7.37 (m, 2H), 7.38-7.45 (m, 3H), 7.52 (td, *J* = 7.9 Hz, *J* = 1.2 Hz, 1H), 8.44 (br. s, 1H).

^{13}C NMR (125.75 MHz, $CDCl_3$): δ 24.2, 25.9, 28.1, 28.7, 29.1, 29.2, 29.7, 32.8, 34.0, 57.6, 68.0, 112.9, 115.0, 117.3, 118.3, 118.5, 125.5, 125.6, 125.8, 128.9, 129.6, 129.6, 130.6, 130.7, 138.8, 140.4, 143.0, 159.8, 169.1.

HRMS (ESI$^+$, m/z): $[C_{31}H_{34}O_2N_2{}^{79}Br]^+$ $(M+H)^+$ calc. 545.1798, found 545.1789.

(2*RS*,3*RS*)-1-acetyl-2-(3-((9-bromononyl)oxy)phenyl)-3-phenylindoline-3-carbonitrile **49**

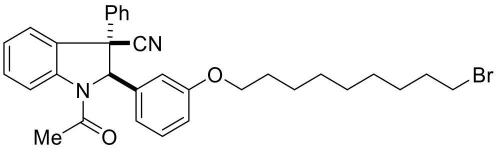

Prepared according to general procedure 4 to afford the title compound after column chromatography (SiO_2, EtOAc/PE 3:17) as a white solid (71% yield).

^{1}H NMR (700 MHz, $CDCl_3$): δ 1.30-1.38 (m, 6H), 1.40-1.48 (m, 4H), 1.72-1.79 (m, 2H), 1.84-1.90 (m, 2H), 1.97 (s, 3H), 3.42 (t, *J* = 6.8 Hz, 2H), 3.88-3.94 (m, 2H), 5.26 (s, 1H), 6.74 (s, 1H), 6.80 (d, *J* = 7.8 Hz, 1H), 6.92 (d, *J* = 8.5 Hz, 1H), 7.23 (t, *J* = 7.5 Hz, 1H), 7.25-7.28 (m, 2H), 7.29-7.35 (m, 2H), 7.36-7.43 (m, 3H), 7.50 (t, *J* = 7.9 Hz, 1H), 8.43 (br. s, 1H).

^{13}**C NMR (176.05 MHz, CDCl$_3$):** δ 24.1, 26.0, 28.1, 28.7, 29.2, 29.3, 29.3, 32.8, 34.0, 57.6, 68.0, 76.5, 112.9, 115.0, 117.3, 118.3, 118.5, 125.5, 125.6, 125.7, 128.7, 128.9, 129.6, 130.6, 130.7, 138.8, 140.4, 143.1, 159.8, 169.1.

HRMS (ESI$^+$, m/z): [$C_{32}H_{35}{}^{79}BrN_2NaO_2$]$^+$ (M+Na)$^+$ calc. 581.1774, found 581.1777.

(2*RS*,3*RS*)-1-Benzoyl-2-(3-((7-bromoheptyl)oxy)phenyl)-3-phenylindoline-3-carbonitrile **50**

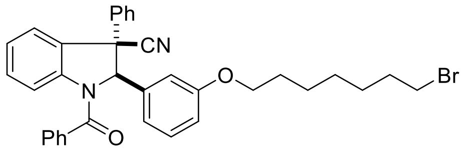

Prepared according to general procedure 4 to afford the title compound after column chromatography (SiO$_2$, EtOAc/PE 3:17) as a white solid (74% yield, contains minor impurities).

^{1}**H NMR (700 MHz, CDCl$_3$):** δ 1.36-1.42 (m, 2H), 1.42-1.52 (m, 4H), 1.71-1.78 (m, 2H), 1.86-1.92 (m, 2H), 3.44 (t, *J* = 6.8 Hz, 2H), 3.83-3.91 (m, 2H), 5.36 (s, 1H), 6.57 (s, 1H), 6.65 (s, 1H), 6.87 (dd, *J* = 8.2 Hz, *J* = 2.0 Hz, 1H), 7.01-7.51 (m, 15H).

^{13}**C NMR (176.05 MHz, CDCl$_3$):** δ 25.9, 28.1, 28.5, 29.0, 32.7, 34.0, 57.0, 67.8, 77.2, 112.9, 115.1, 117.3, 118.5, 118.6, 125.7, 126.0, 126.9, 128.4, 128.5, 128.9, 129.5, 130.0, 130.1, 130.4, 130.7, 135.6, 138.9, 139.6, 143.0, 159.3, 169.5.

HRMS (ESI$^+$, m/z): [$C_{35}H_{34}O_2N_2{}^{79}Br$]$^+$ (M+H)$^+$ calc. 593.1798, found 593.1792.

(2*RS*,3*RS*)-2-(3-((7-Bromoheptyl)oxy)phenyl)-1-(3-methylbutanoyl)-3-phenylindoline-3-carbonitrile **52**

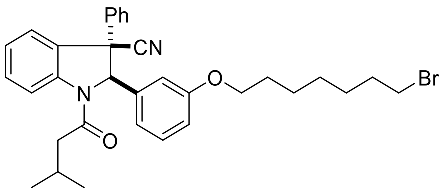

Prepared according to general procedure 4 to afford the title compound after column chromatography (SiO$_2$, EtOAc/PE 1:9) as a white solid (73% yield).

^{1}H NMR (700 MHz, CDCl$_3$): δ 0.69-0.88 (m, 6H), 1.36-1.42 (m, 2H), 1.44-1.52 (m, 4H), 1.74-1.80 (m, 2H), 1.85-2.00 (m, 3H), 2.01-2.21 (m, 2H), 3.44 (t, *J* = 6.9 Hz, 2H), 3.90-3.96 (m, 2H), 5.32 (s, 1H), 6.74 (s, 1H), 6.81 (d, *J* = 7.6 Hz, 1H), 6.92 (dd, *J* = 8.1 Hz, *J* = 1.6 Hz, 1H), 7.24 (t, *J* = 7.5 Hz, 1H), 7.26-7.29 (m, 2H), 7.32 (t, *J* = 7.9 Hz, 1H), 7.35 (d, *J* = 7.7 Hz, 1H), 7.36-7.43 (m, 3H), 7.52 (t, *J* = 7.9 Hz, 1H), 8.47 (br. s, 1H).

^{13}C NMR (176.05 MHz, CDCl$_3$): δ 22.2, 22.4, 25.3, 25.8, 28.1, 28.5, 29.0, 32.7, 33.9, 44.3, 57.5, 67.9, 75.8, 112.9, 115.1, 117.4, 118.5, 118.6, 125.4, 125.5, 125.7, 128.1, 128.9, 129.5, 130.5, 130.7, 139.0, 140.4, 143.2, 159.7, 171.3.

HRMS (ESI$^+$, m/z): $[C_{33}H_{38}O_2N_2{}^{79}Br]^+$ $(M+H)^+$ calc. 573.2111, found 573.2104.

(2*RS*,3*RS*)-2-(3-((7-Bromoheptyl)oxy)phenyl)-1-(furan-2-carbonyl)-3-phenylindoline-3-carbonitrile **53**

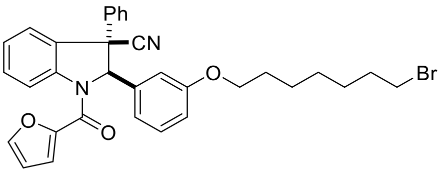

Prepared according to general procedure 4 to afford the title compound after column chromatography (SiO_2, EtOAc/PE 1:4) as a white solid (66% yield).

^{1}H NMR (700 MHz, CDCl$_3$): δ 1.36-1.41 (m, 2H), 1.42-1.51 (m, 4H), 1.72-1.78 (m, 2H), 1.86-1.92 (m, 2H), 3.43 (t, *J* = 6.9 Hz, 2H), 3.85-3.92 (m, 2H), 6.05 (s, 1H), 6.38 (dd, *J* = 3.5 Hz, *J* = 1.7 Hz, 1H), 6.74 (t, *J* = 1.8 Hz, 1H), 6.83 (d, *J* = 7.7 Hz, 1H), 6.85 (dd, *J* = 8.2 Hz, *J* = 2.0 Hz, 1H), 7.02 (d, *J* = 3.5 Hz, 1H), 7.26 (t, *J* = 7.9 Hz, 1H), 7.27-7.30 (m, 1H), 7.33-7.36 (m, 2H), 7.36-7.40 (m, 3H), 7.40-7.44 (m, 2H), 7.54 (t, *J* = 7.9 Hz, 1H), 8.36 (br. s, 1H).

^{13}C NMR (176.05 MHz, CDCl$_3$): δ 25.8, 28.1, 28.5, 29.0, 32.7, 34.0, 58.0, 67.9, 75.8, 111.9, 112.7, 114.9, 118.0, 118.3, 118.4, 118.6, 125.7, 125.7, 126.0, 128.8, 129.3, 129.5, 130.1, 130.6, 139.5, 140.1, 143.5, 144.7, 147.1, 157.8, 159.4.

HRMS (ESI$^+$, m/z): $[C_{33}H_{32}O_3N_2{}^{79}Br]^+$ $(M+H)^+$ calc. 583.1591, found 583.1585.

(2*RS*,3*RS*)-2-(3-((7-Bromoheptyl)oxy)phenyl)-3-phenyl-1-(4-(trifluoromethyl)benzoyl)indoline-3-carbonitrile **54**

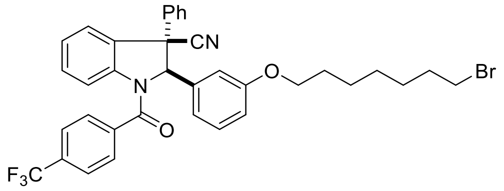

Prepared according to general procedure 4 to afford the title compound after column chromatography (SiO_2, EtOAc/PE 1:9) as a white solid (86% yield).

^{1}H NMR (500 MHz, $CDCl_3$): δ 1.35-1.53 (m, 6H), 1.71-1.79 (m, 2H), 1.90 (tt, *J* = 7.3 Hz, *J* = 6.8 Hz, 2H), 3.44 (t, *J* = 6.8 Hz, 2H), 3.79-3.90 (m, 2H), 5.21 (br. s, 1H), 6.49 (br. s, 1H), 6.62 (br. s, 1H), 6.89 (dd, *J* = 8.2 Hz, *J* = 2.0 Hz, 1H), 7.14-7.35 (m, 6H), 7.40-7.47 (m, 4H), 7.47-7.57 (m, 3H), 8.22 (br. s, 1H).

^{13}C NMR (125.75 MHz, $CDCl_3$): δ 25.8, 28.1, 28.5, 29.0, 32.7, 34.0, 57.1, 60.4, 67.9, 112.9, 115.3, 117.5, 118.4, 123.6 (q, *J* = 274.0 Hz), 125.5, 125.6, 126.1, 126.3, 127.2, 129.1, 129.6, 129.9, 130.3, 130.7, 132.3 (q, *J* = 29.7 Hz), 138.6, 139.0, 139.6, 142.6, 159.5, 168.1.

HRMS (ESI$^+$, m/z): $[C_{36}H_{33}O_2N_2{}^{79}BrF_3]^+$ $(M+H)^+$ calc. 661.1672, found 661.1664.

Ethyl (2*RS*,3*RS*)-2-(3-((7-bromoheptyl)oxy)phenyl)-3-cyano-3-phenylindoline-1-carboxylate **55**

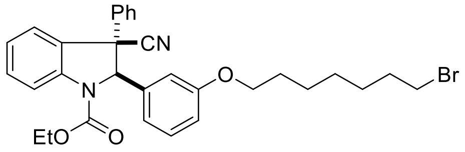

Prepared according to general procedure 4 to afford the title compound after column chromatography (SiO_2, EtOAc/PE 1:8) as a white solid (56% yield).

^{1}H NMR (700 MHz, $CDCl_3$): δ 1.11 (br. s, 3H), 1.36-1.42 (m, 2H), 1.44-1.53 (m, 4H), 1.74-1.81 (m, 2H), 1.86-1.93 (m, 2H), 3.44 (t, *J* = 6.8 Hz, 2H), 3.91-3.96 (m, 2H), 4.05-4.23 (m, 2H), 5.38 (s, 1H), 6.76 (s, 1H), 6.81 (d, *J* = 7.6 Hz 1H), 6.90 (dd, *J* = 8.2 Hz, *J* = 2.1 Hz, 1H), 7.19 (t, *J* = 7.5 Hz, 1H), 7.27-7.31 (m, 3H), 7.33 (d, *J* = 7.5 Hz, 1H), 7.36-7.44 (m, 3H), 7.50 (t, *J* = 7.8 Hz, 1H), 8.04 (br. s, 1H).

^{13}C NMR (176.05 MHz, $CDCl_3$): δ 14.2, 25.9, 28.1, 28.5, 29.1, 32.7, 34.0, 56.8, 62.1, 67.8, 75.3, 112.8, 114.7, 115.2, 118.3, 118.8, 124.4, 125.8, 125.9, 128.7, 128.7, 129.4, 130.0, 130.7, 139.6, 140.7, 142.5, 152.5, 159.4.

HRMS (ESI$^+$, m/z): $[C_{31}H_{34}O_3N_2{}^{79}Br]^+$ $(M+H)^+$ calc. 561.1747, found 561.1740.

(2*RS*,3*RS*)-1-Acetyl-3-phenyl-2-(3-(4-(pyrrolidin-1-yl)butoxy)phenyl)indoline-3-carbonitrile **56**

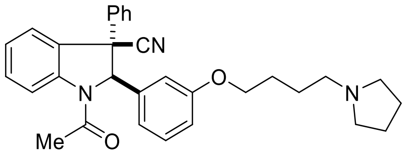

Prepared according to general procedure 5 to afford the title compound as a highly hydroscopic colourless solid (99% yield).

^{1}H NMR (400 MHz, CDCl$_3$): δ 1.72-2.06 (m, 11H), 2.58-2.73 (m, 6H), 3.89-3.99 (m, 2H), 5.26 (s, 1H), 6.73 (t, *J* = 1.8 Hz, 1H), 6.80 (d, *J* = 7.8 Hz, 1H), 6.90 (dd, *J* = 8.3 Hz, *J* = 2.1 Hz, 1H), 7.22 (td, *J* = 7.5 Hz, *J* = 0.9 Hz, 1H), 7.24-7.28 (m, 2H), 7.28-7.34 (m, 2H), 7.36-7.43 (m, 3H), 7.50 (ddd, *J* = 8.9 Hz, *J* = 7.6 Hz, *J* = 1.3 Hz, 1H), 8.42 (br. s, 1H).

^{13}C NMR (100.6 MHz, CDCl$_3$): δ 23.4, 24.2, 24.9, 27.1, 54.1, 55.9, 57.6, 67.6, 76.4, 112.9, 115.0, 117.2, 118.4, 118.6, 125.5, 125.6, 125.7, 128.9, 129.6, 130.6, 130.7, 130.7, 138.8, 140.4, 143.0, 159.6, 169.1.

HRMS (ESI$^+$, m/z): $[C_{31}H_{34}O_2N_3]^+$ $(M+H)^+$ calc. 480.2646, found 480.2632.

(2*RS*,3*RS*)-1-Acetyl-3-phenyl-2-(3-((5-(pyrrolidin-1-yl)pentyl)oxy)phenyl)indoline-3-carbonitrile **57**

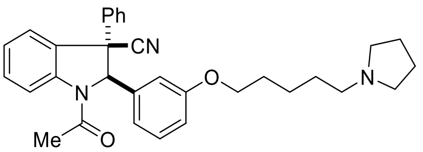

Prepared according to general procedure 5 to afford the title compound as a highly hydroscopic colourless solid (87% yield).

^{1}H NMR (500 MHz, CDCl$_3$): δ 1.43-1.54 (m, 2H), 1.64-1.73 (m, 2H), 1.73-1.82 (m, 6H), 1.83-2.10 (br. s, 3H), 2.60-2.66 (m, 2H), 2.70-2.78 (m, 4H), 3.86-3.95 (m, 2H), 5.26 (s, 1H), 6.72 (t, *J* = 1.7 Hz, 1H), 6.78 (d, *J* = 7.9 Hz, 1H), 6.89 (dd, *J* = 8.4 Hz, *J* = 2.0 Hz, 1H), 7.21 (td, *J* = 7.6 Hz, *J* = 0.9 Hz, 1H), 7.22-7.26 (m, 2H), 7.27-7.33 (m, 2H), 7.34-7.42 (m, 3H), 7.46-7.52 (m, 1H), 8.41 (br. s, 1H).

^{13}C NMR (125.75 MHz, $CDCl_3$): δ 23.4, 23.9, 24.2, 27.6, 28.9, 54.0, 56.1, 57.7, 67.6, 76.5, 112.9, 115.0, 117.2, 118.4, 118.6, 125.4, 125.5, 125.7, 125.7, 128.9, 130.6, 130.7, 138.8, 140.4, 143.0, 159.6, 169.1.ǂ

ǂ 1 peak obscured.

HRMS (ESI$^+$, m/z): $[C_{32}H_{36}O_2N_3]^+$ $(M+H)^+$ calc. 494.2802, found 494.2785.

(2*RS*,3*RS*)-1-Acetyl-3-phenyl-2-(3-((6-(pyrrolidin-1-yl)hexyl)oxy)phenyl)indoline-3-carbonitrile **58**

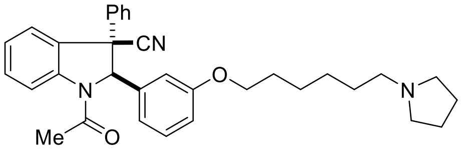

Prepared according to general procedure 5 to afford the title compound as a highly hydroscopic colourless solid (80% yield).

^{1}H NMR (400 MHz, $CDCl_3$): δ 1.36-1.54 (m, 4H), 1.62-1.72 (m, 2H), 1.73-1.82 (m, 2H), 1.85-2.06 (m, 7H), 2.57-2.64 (m, 2H), 2.65-2.78 (m, 4H), 3.92 (t, *J* = 6.4 Hz, 2H), 5.27 (s, 1H), 6.75 (s, 1H), 6.81 (d, *J* = 7.7 Hz, 1H), 6.92 (dd, *J* = 8.2 Hz, *J* = 1.9 Hz, 1H), 7.21-7.30 (m, 3H), 7.32 (t, *J* = 8.0 Hz, 2H), 7.37-7.45 (m, 3H), 7.51 (td, *J* = 8.4 Hz, *J* = 1.3 Hz, 1H), 8.43 (br. s, 1H).

^{13}C NMR (100.6 MHz, $CDCl_3$): δ 23.4, 24.2, 25.9, 27.2, 28.1, 29.0, 54.1, 57.6, 56.3, 67.8, 76.4, 112.9, 115.0, 118.3, 118.5, 125.4, 125.6, 125.7, 128.9, 129.6, 130.6, 130.7, 138.8, 140.4, 143.0, 159.7, 169.1.ǂ

ǂ 2 peaks obscured.

HRMS (ESI$^+$, m/z): $[C_{33}H_{38}O_2N_3]^+$ $(M+H)^+$ calc. 508.2959, found 508.2945.

(2*RS*,3*RS*)-1-Acetyl-3-phenyl-2-(3-((7-(pyrrolidin-1-yl)heptyl)oxy)phenyl)indoline-3-carbonitrile **59**

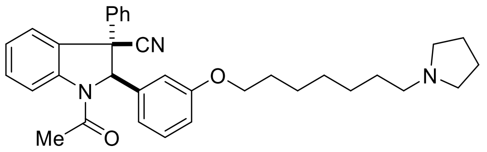

Prepared according to general procedure 5 to afford the title compound as a highly hydroscopic colourless solid (90% yield).

^{1}H NMR (400 MHz, $CDCl_3$): δ 1.32-1.50 (m, 6H), 1.58-1.69 (m, 2H), 1.70-1.79 (m, 2H), 1.84-2.06 (m, 7H), 2.55-2.62 (m, 2H), 2.64-2.79 (m, 4H), 3.85-3.95 (m, 2H), 5.25 (s, 1H), 6.73 (t, *J* = 1.7 Hz, 1H), 6.79 (d, *J* = 7.9 Hz, 1H), 6.91 (dd, *J* = 8.0 Hz, *J* = 2.0 Hz, 1H), 7.22 (td, *J* = 7.5 Hz, *J* = 1.0 Hz, 1H), 7.24-7.27 (m, 2H), 7.28-7.34 (m, 2H), 7.36-7.43 (m, 3H), 7.47-7.53 (m, 1H), 8.41 (br. s, 1H).

^{13}C NMR (100.6 MHz, $CDCl_3$): δ 23.4, 24.2, 25.9, 27.4, 28.0, 29.1, 29.2, 54.1, 56.4, 57.5, 67.9, 76.5, 112.9, 115.0, 117.2, 118.3, 118.5, 125.4, 125.6, 125.8, 128.9, 129.6, 130.6, 130.6, 130.7, 138.8, 140.4, 143.1, 159.7, 169.2.

HRMS (ESI$^+$, m/z): $[C_{34}H_{40}O_2N_3]^+$ $(M+H)^+$ calc. 522.3126, found 522.3104.

(2*RS*,3*RS*)-1-Acetyl-3-phenyl-2-(3-((8-(pyrrolidin-1-yl)octyl)oxy)phenyl)indoline-3-carbonitrile **60**

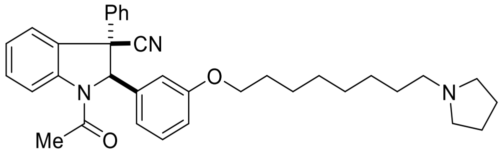

Prepared according to general procedure 5 to afford the title compound as a highly hydroscopic colourless solid (98% yield).

^{1}H NMR (400 MHz, $CDCl_3$): δ 1.29-1.48 (m, 8H), 1.57-1.68 (m, 2H), 1.69-1.78 (m, 2H), 1.83-2.06 (m, 7H), 2.55-2.63 (m, 2H), 2.66-2.78 (m, 4H), 3.85-3.93 (m, 2H), 5.26 (s, 1H), 6.73 (t, *J* = 1.7 Hz, 1H), 6.78 (d, *J* = 8.1 Hz, 1H), 6.90 (dd, *J* = 8.3 Hz, *J* = 2.2 Hz, 1H), 7.21 (td, *J* = 7.5 Hz, *J* = 0.7 Hz, 1H), 7.23-7.34 (m, 4H), 7.35-7.42 (m, 3H), 7.46-7.51 (m, 1H), 8.41 (br. s, 1H).

^{13}C NMR (100.6 MHz, $CDCl_3$): δ 23.4, 24.1, 25.9, 27.4, 28.0, 29.1, 29.2, 29.3, 54.0, 56.4, 57.6, 68.0, 76.5, 112.9, 115.0, 117.2, 118.3, 118.5, 125.4, 125.7, 125.8, 128.9, 129.6, 130.6, 130.6, 130.7, 138.8, 140.4, 143.0, 159.8, 169.2.

HRMS (ESI$^+$, m/z): $[C_{35}H_{42}O_2N_3]^+$ $(M+H)^+$ calc. 536.3283, found 536.3258.

(2*RS*,3*RS*)-1-Acetyl-3-phenyl-2-(3-((9-(pyrrolidin-1-yl)nonyl)oxy)phenyl)indoline-3-carbonitrile **62**

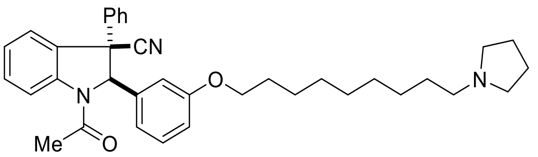

Prepared according to general procedure 5 to afford the title compound as a highly hydroscopic colourless solid (99% yield).

^{1}H NMR (400 MHz, CDCl$_3$): δ = 1.26-1.45 (m, 10H), 1.60-1.79 (m, 4H), 1.86-2.04 (m, 7H), 2.61-2.70 (m, 2H), 2.76-2.88 (m, 4H), 3.84-3.93 (m, 2H), 5.24 (s, 1H), 6.72 (t, *J* = 1.7 Hz, 1H), 6.78 (d, *J* = 7.8 Hz, 1H), 6.90 (dd, *J* = 8.0 Hz, *J* = 1.9 Hz, 1H), 7.21 (td, *J* = 7.6 Hz, *J* = 0.8 Hz, 1H), 7.22-7.26 (m, 2H), 7.27-7.33 (m, 2H), 7.34-7.42 (m, 3H), 7.48 (ddd, *J* = 8.9 Hz, *J* = 7.6 Hz, *J* = 1.3 Hz, 1H), 8.40 (br. s, 1H).

^{13}C NMR (100.6 MHz, CDCl$_3$): δ 23.4, 24.2, 26.0, 27.3, 27.6, 29.1, 29.2, 29.3, 29.4, 53.9, 56.2, 57.6, 68.0, 76.4, 112.9, 115.0, 117.2, 118.2, 118.5, 125.4, 125.5, 125.7, 125.7, 128.9, 129.6, 130.6, 130.7, 138.8, 140.4, 143.0, 159.8, 169.1.

HRMS (ESI$^+$, m/z): $[C_{36}H_{44}O_2N_3]^+$ $(M+H)^+$ calc. 550.3428, found 550.3411.

(2*RS*,3*RS*)-1-Benzoyl-3-phenyl-2-(3-((7-(pyrrolidin-1-yl)heptyl)oxy)phenyl)indoline-3-carbonitrile **63**

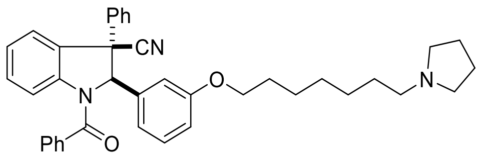

Prepared according to general procedure 5 to afford the title compound as a highly hydroscopic colourless solid (99% yield).

^{1}H NMR (500 MHz, CDCl$_3$): δ 1.34-1.49 (m, 6H), 1.69-1.78 (m, 4H), 1.93-2.04 (m, 4H), 2.67-2.77 (m, 2H), 2.78-3.00 (m, 4H), 3.82-3.91 (m, 2H), 5.36 (s, 1H), 6.57 (br. s, 1H), 6.64 (d, *J* = 6.8 Hz, 1H), 6.87 (dd, *J* = 8.4 Hz, *J* = 2.2 Hz, 1H), 7.08-7.51 (m, 15H).

^{13}C NMR (125.75 MHz, CDCl$_3$): δ 23.4, 25.8, 27.2, 27.2, 29.0, 29.0, 53.9, 56.1, 57.0, 67.8, 77.2, 112.8, 115.2, 117.2, 118.5, 118.7, 125.7, 125.7, 126.0, 126.9, 128.5, 128.9, 129.5, 129.9, 130.1, 130.4, 130.7, 135.6, 138.9, 139.6, 143.0, 159.3, 169.5.

HRMS (ESI$^+$, m/z): $[C_{39}H_{42}N_3O_2]^+$ $(M+H)^+$ calc. 584.3272, found 584.3274.

(2*RS*,3*RS*)-1-(Furan-2-carbonyl)-3-phenyl-2-(3-((7-(pyrrolidin-1-yl)heptyl)oxy)phenyl)indoline-3-carbonitrile **64**

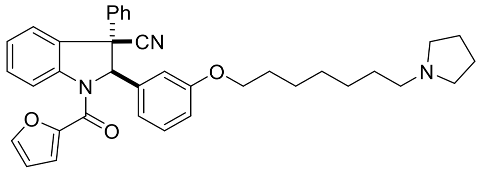

Prepared according to general procedure 5 to afford the title compound as a highly hydroscopic colourless solid (99% yield).

^{1}H NMR (500 MHz, CDCl$_3$): δ 1.28-1.47 (m, 6H), 1.62-1.76 (m, 4H), 1.84-1.95 (m, 4H), 2.57-2.67 (m, 2H), 2.69-2.85 (m, 4H), 3.81-3.92 (m, 2H), 6.03 (s, 1H), 6.35-6.38 (m, 1H), 6.73 (s, 1H), 6.80 (d, *J* = 7.8 Hz, 1H), 6.83 (d, *J* = 8.2 Hz, 1H), 6.99 (d, *J* = 2.9 Hz, 1H), 7.20-7.29 (m, 2H), 7.30-7.43 (m, 7H), 7.52 (t, *J* = 7.8 Hz, 1H), 8.35 (br. s, 1H).

^{13}C NMR (125.75 MHz, CDCl$_3$): δ 23.8, 26.3, 27.7, 28.2, 29.5, 29.5, 54.4, 56.7, 58.5, 68.3, 76.2, 112.3, 113.1, 115.3, 118.4, 118.8, 119.0, 126.1, 126.2, 126.4, 129.3, 129.7, 130.0, 130.5, 131.0, 139.9, 140.5, 143.9, 145.1, 147.5, 158.2, 159.8.ƚ

ƚ 1 peak obscured.

HRMS (ESI$^+$, m/z): $[C_{37}H_{40}O_3N_3]^+$ $(M+H)^+$ calc. 574.3064, found 574.3050.

(2*RS*,3*RS*)-1-(3-Methylbutanoyl)-3-phenyl-2-(3-((7-(pyrrolidin-1-yl)heptyl)oxy)phenyl)indoline-3-carbonitrile **65**

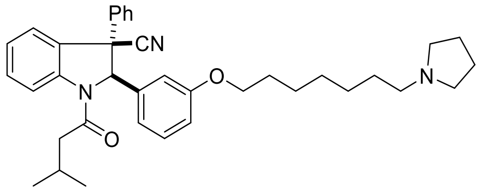

Prepared according to general procedure 5 to afford the title compound as a highly hydroscopic colourless solid (99% yield).

^{1}H NMR (500 MHz, CDCl$_3$): δ 0.67-0.86 (m, 6H), 1.34-1.51 (m, 6H), 1.63-1.82 (m, 4H), 1.87-2.21 (m, 7H), 2.61-2.70 (m, 2H), 2.71-2.89 (m, 4H), 3.88-3.95 (m, 2H), 5.32 (s, 1H), 6.75 (t, *J* = 1.7 Hz, 1H), 6.80 (d, *J* = 7.6 Hz, 1H), 6.92 (dd, *J* = 8.3 Hz, *J* = 2.1 Hz, 1H), 7.24 (td, *J* = 7.6 Hz, *J* = 0.9 Hz, 1H), 7.26-7.29 (m, 2H), 7.29-7.37 (m, 2H), 7.37-7.44 (m, 3H), 7.52 (ddd, *J* = 8.8 Hz, *J* = 7.4 Hz, *J* = 1.3 Hz, 1H), 8.47 (br. s, 1H).

^{13}C NMR (125.75 MHz, CDCl$_3$): δ 22.2, 22.4, 23.4, 23.4, 25.3, 25.9, 27.3, 27.7, 29.0, 29.1, 44.3, 54.0, 56.2, 67.9, 75.8, 112.9, 115.1, 117.4, 118.4, 118.6, 125.4, 125.5, 125.7, 128.1, 128.9, 129.5, 130.5, 139.0, 140.3, 143.2, 159.7, 171.3.

HRMS (ESI$^+$, m/z): $[C_{37}H_{46}N_3O_2]^+$ $(M+H)^+$ calc. 564.3585, found 564.3591.

(2*RS*,3*RS*)-3-Phenyl-2-(3-((7-(pyrrolidin-1-yl)heptyl)oxy)phenyl)-1-(4-(trifluoromethyl)benzoyl)indoline-3-carbonitrile **66**

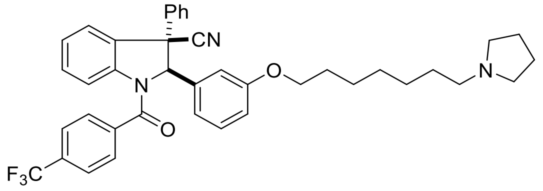

Prepared according to general procedure 5 to afford the title compound as a highly hydroscopic colourless solid (78% yield).

^{1}H NMR (500 MHz, CDCl$_3$): δ 1.32-1.50 (m, 6H), 1.63-1.78 (m, 4H), 1.87-1.99 (m, 4H), 2.60-2.69 (m, 2H), 2.69-2.88 (m, 4H), 3.77-3.91 (m, 2H), 5.21 (br. s, 1H), 6.47 (br. s, 1H), 6.61 (d, *J* = 5.9 Hz, 1H), 6.88 (dd, *J* = 8.3 Hz, *J* = 2.4 Hz, 1H), 7.12-7.35 (m, 7H), 7.37-7.63 (m, 7H).

^{13}C NMR (125.75 MHz, CDCl$_3$): δ 23.4, 25.8, 27.3, 27.8, 29.0, 29.1, 54.0, 56.3, 57.1, 67.9, 77.3, 112.9, 115.3, 117.5, 118.4, 123.5 (q, *J* = 273.1 Hz), 125.5, 125.6, 126.1, 126.3, 127.2, 128.3 (q, *J* = 18.4 Hz), 129.1, 129.6, 129.9, 130.3, 130.7, 132.3 (q, *J* = 33.6 Hz), 138.6, 139.0, 139.6, 142.6, 159.5, 168.2.

HRMS (ESI$^+$, m/z): $[C_{40}H_{41}O_2N_3F_3]^+$ $(M+H)^+$ calc. 652.3156, found 652.3128.

Ethyl (2*RS*,3*RS*)-3-cyano-3-phenyl-2-(3-((7-(pyrrolidin-1-yl)heptyl)oxy)phenyl)indoline-1-carboxylate **67**

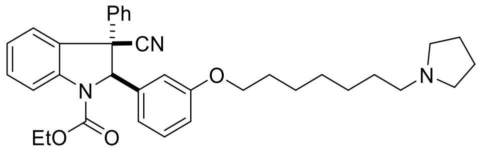

Prepared according to general procedure 5 to afford the title compound as a highly hydroscopic colourless solid (86% yield).

^{1}H NMR (700 MHz, CDCl$_3$): δ 1.10 (br. s, 3H), 1.35-1.41 (m, 4H), 1.43-1.49 (m, 2H), 1.62-1.70 (m, 2H), 1.73-1.79 (m, 2H), 1.87-1.95 (m, 4H), 2.61 (t, *J* = 7.5 Hz, 2H), 2.66-2.82 (m, 4H), 3.92 (t, *J* = 6.4 Hz, 2H), 4.04-4.23 (m, 2H), 5.38 (s, 1H), 6.75 (s, 1H), 6.80 (d, *J* = 7.6 Hz, 1H), 6.89 (dd, *J* = 8.0 Hz, *J* = 2.2 Hz, 1H), 7.18 (t, *J* = 7.6 Hz, 1H), 7.27-7.30 (m, 3H), 7.32 (d, *J* = 7.7 Hz, 1H), 7.36-7.39 (m, 1H), 7.39-7.43 (m, 2H), 7.49 (t, *J* = 8.0 Hz, 1H), 8.03 (br. s, 1H).

^{13}C NMR (176.05 MHz, CDCl$_3$): δ 14.2, 23.4, 25.9, 27.4, 27.9, 29.1, 29.2, 54.0, 56.3, 56.8, 62.1, 67.9, 75.3, 112.8, 114.7, 115.2, 118.2, 118.8, 124.4, 125.8, 125.9, 128.7, 128.7, 129.4, 130.0, 130.7, 139.6, 140.6, 142.4, 152.5, 159.4.

HRMS (ESI$^+$, m/z): $[C_{35}H_{42}N_3O_3]^+$ $(M+H)^+$ calc. 552.3221, found 552.3223.

(2*RS*,3*RS*)-1-Acetyl-2-(3-((7-(diethylamino)heptyl)oxy)phenyl)-3-phenylindoline-3-carbonitrile **18**

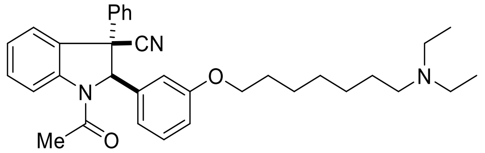

To a solution of *syn*-1-acetyl-2-(3-((7-bromoheptyl)oxy)phenyl)-3-phenylindoline-3-carbonitrile **47** (300 mg) in MeCN (5 mL) was added K_2CO_3 (5.0 eq.) and diethylamine (4.0 eq.). The reaction was heated to 60 °C overnight before diluting with EtOAc and washing with water and brine. The organic phase was dried over $MgSO_4$ and evaporated under reduced pressure. Purification by column chromatography (SiO_2, DCM/MeOH/Et_3N 95:5:0.5) afforded the title compound as a highly hydroscopic colourless solid (75% yield).

^{1}H NMR (400 MHz, CDCl$_3$): δ 1.23 (t, *J* = 7.2 Hz, 6H), 1.31-1.52 (m, 6H), 1.60-1.71 (m, 2H), 1.71-1.81 (m, 2H), 1.97 (br. s, 3H), 2.66-2.73 (m, 2H), 2.83 (q, *J* = 7.2 Hz, 4H), 3.87-3.96 (m, 2H), 5.27 (br. s, 1H), 6.75 (t, *J* = 1.7 Hz, 1H), 6.81 (d, *J* = 7.7 Hz, 1H), 6.92 (dd, *J* = 8.2 Hz, *J* = 2.1 Hz, 1H), 7.24 (dd, *J* = 7.6 Hz, *J* = 0.9 Hz, 1H), 7.25-7.28 (m, 2H), 7.30-7.36 (m, 2H), 7.37-7.45 (m, 3H), 7.49-7.55 (m, 1H), 8.43 (br. s, 1H).

^{13}C NMR (100.6 MHz, CDCl$_3$): δ 10.1, 24.2, 25.0, 25.9, 27.2, 29.0, 29.1, 46.0, 46.6, 52.0, 67.9, 76.4, 112.9, 115.0, 117.3, 118.3, 118.5, 125.4, 125.5, 125.7, 128.9, 129.6, 130.6, 130.6, 130.7, 138.8, 140.4, 143.2, 159.7.ǂ

ǂ 1 peak obscured.

HRMS (ESI$^+$, m/z): $[C_{34}H_{42}O_2N_3]^+$ $(M+H)^+$ calc. 524.3272, found 524.3257.

(2*RS*,3*RS*)-1-Acetyl-3-phenyl-2-(3-((7-(piperidin-1-yl)heptyl)oxy)phenyl)indoline-3-carbonitrile **69**

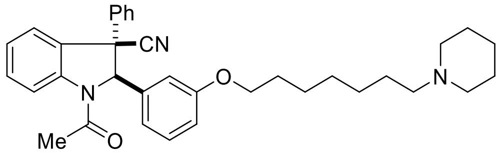

To a solution of *syn*-1-acetyl-2-(3-((7-bromoheptyl)oxy)phenyl)-3-phenylindoline-3-carbonitrile **47** (300 mg) in MeCN (5 mL) was added K_2CO_3 (5.0 eq.) and piperidine (4.0 eq.). The reaction was heated to 60 °C overnight before diluting with EtOAc and washing with water and brine. The organic phase was dried over $MgSO_4$ and evaporated under reduced pressure. Purification by column chromatography (SiO_2, DCM/MeOH/Et_3N 95:5:0.5) afforded the title compound as a highly hydroscopic colourless solid (65% yield).

^{1}H NMR (500 MHz, CDCl$_3$): δ 1.29-1.56 (m, 9H), 1.59-1.68 (m, 2H), 1.69-1.80 (m, 6H), 1.98 (br. s, 3H, *Ac*), 2.43-2.50 (m, 2H), 2.50-2.67 (m, 3H), 3.92 (td, *J* = 6.5 Hz, *J* = 1.8 Hz, 2H), 5.27 (br. s, 1H), 6.75 (br. s, 1H), 6.81 (d, *J* = 7.8 Hz, 1H), 6.92 (dd, *J* = 8.3 Hz, *J* = 2.1 Hz, 1H), 7.24 (td, *J* = 7.6 Hz, *J* = 1.0 Hz, 1H), 7.26-7.29 (m, 2H), 7.30-7.36 (m, 2H), 7.37-7.44 (m, 3H), 7.51 (ddd, *J* = 8.1 Hz, *J* = 7.6 Hz, *J* = 1.3 Hz, 1H), 8.43 (br. s, 1H).

^{13}C NMR (125.75 MHz, CDCl$_3$): δ 23.9, 24.2, 25.0, 25.9, 25.9, 27.4, 29.1, 29.2, 54.2, 57.6, 59.0, 67.9, 76.5, 112.9, 115.0, 117.3, 118.3, 118.5, 125.5, 125.6, 125.7, 128.7, 128.9, 129.6, 130.6, 130.7, 138.8, 140.4, 143.0, 159.7, 169.1.

HRMS (ESI$^+$, m/z): $[C_{35}H_{42}O_2N_3]^+$ $(M+H)^+$ calc. 536.3272, found 536.3258.

(2*RS*,3*RS*)-1-Acetyl-2-(3-((7-morpholinoheptyl)oxy)phenyl)-3-phenylindoline-3-carbonitrile **70**

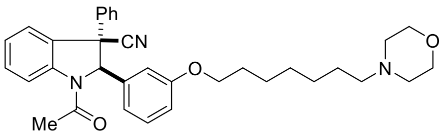

To a solution of *syn*-1-acetyl-2-(3-((7-bromoheptyl)oxy)phenyl)-3-phenylindoline-3-carbonitrile **47** (300 mg) in MeCN (5 mL) was added K_2CO_3 (5.0 eq.) and morpholine (4.0 eq.). The reaction was heated to 60 °C overnight before diluting with EtOAc and washing with water and brine. The organic phase was dried over $MgSO_4$ and evaporated under reduced pressure. Purification by column chromatography (SiO_2, DCM/MeOH/Et_3N 95:5:0.5) afforded the title compound as a highly hydroscopic colourless solid (88% yield).

^{1}H NMR (400 MHz, CDCl$_3$): δ 1.33-1.59 (m, 8H), 1.73-1.82 (m, 2H), 1.98 (br. s, 3H), 2.32-2.39 (m, 2H), 2.42-2.52 (m, 4H), 3.75 (t, *J* = 4.6 Hz, 4H), 3.88-3.97 (m, 2H), 5.28 (br. s, 1H), 6.75 (t, *J* = 1.9 Hz, 1H), 6.82 (d, *J* = 7.7 Hz, 1H), 6.93 (dd, *J* = 8.2 Hz, *J* = 2.1 Hz, 1H), 7.25 (td, *J* = 7.5 Hz, *J* = 1.0 Hz, 1H), 7.26-7.28 (m, 2H), 7.31-7.37 (m, 2H), 7.38-7.45 (m, 3H), 7.52 (ddd, *J* = 8.8 Hz, *J* = 7.5 Hz, *J* = 1.3 Hz, 1H), 8.44 (br. s, 1H).

^{13}C NMR (100.6 MHz, CDCl$_3$): δ 24.2, 26.0, 26.5, 27.4, 29.1, 29.3, 53.8, 56.1, 59.2, 67.0, 68.0, 76.5, 112.9, 115.0, 117.2, 118.3, 118.5, 125.5, 125.6, 125.8, 128.9, 129.6, 130.6, 130.6, 130.7, 138.8, 140.4, 143.1, 159.8.[ł]

ł 1 peak obscured.

HRMS (ESI$^+$, m/z): $[C_{34}H_{40}O_3N_3]^+$ $(M+H)^+$ calc. 538.3064, found 538.3044.

2-(3-Nitropyridin-2-yl)-2-phenylacetonitrile **72**

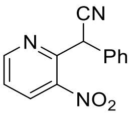

Nitrile **72** was prepared according to a modified literature procedure.[19] Benzyl cyanide (1.20 mL, 10.4 mmol) was added to a suspension of tris[2-(2-methoxyethoxy)ethyl]amine (3.0 mL, 9.4 mmol) and KOH (2.33 g, 41.6 mmol) in THF (25 mL). The mixture was stirred at 50 °C for 10 min, and a solution of 2-chloro-3-nitropyridine (1.48 g, 9.36 mmol) in THF (15 mL) was added over 1 h by syringe pump. The mixture was stirred at 50 °C for a further 30 min, then allowed to cool to rt and quenched with NH_4Cl (saturated aq., 50 mL). The mixture was extracted with EtOAc, and the combined organic extracts were washed with pH 6 phosphate buffer, then brine, dried over $MgSO_4$, filtered, and concentrated. Purification by flash pressure column chromatography (22% EtOAc/petroleum ether) afforded nitrile **72** as a yellow solid (1.02 g, 4.26 mmol, 46%).

^{1}H NMR (400 MHz, CDCl$_3$): δ 8.89 (1H, dd, *J* = 4.6 Hz, 1.5 Hz), 8.32 (1H, dd, *J* = 8.3 Hz, 1.7 Hz), 7.48 (1H, dd, *J* = 8.3 Hz , 4.6 Hz), 7.38-7.44 (2H, m), 7.24-7.32 (3H, m), 6.25 (1H, s).

^{13}C NMR(100.6 MHz, CDCl$_3$): δ 153.7, 149.5, 143.9, 133.8, 133.2, 129.2, 128.9, 128.3, 124.2, 118.0, 42.0.

HRMS (ES$^+$): $[C_{13}H_9O_2N_3Na]^+$ ($[M+Na]^+$) requires 262.0587; found 262.0588.

MP: 96-98 °C.

Ethyl 2-cyano-2-(nitrophenyl)acetate **73**

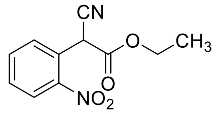

Ethyl cyanoacetate (4.3 mL, 40 mmol) was added dropwise to a suspension of KO^{t}Bu (4.9 g, 44 mmol) in THF (50 mL) and stirred at 0 °C for 10 min. 1-Fluoro-2-nitrobenzene (2.1 mL, 20 mmol) was added dropwise and stirred at 60 °C for 16 h. The mixture was diluted with EtOAc, and HCl (aq., 1 M, 30 mL) was added. The solution with extracted with EtOAc. The combined organic extracts were washed with brine, dried over $MgSO_4$, filtered, and concentrated. Purification by flash pressure column chromatography (45% Et_2O/hexane) afforded nitrile **73** as a yellow oil (1.9 g, 8.0 mmol, 40%).

^{1}H NMR (400 MHz, CDCl$_3$): δ 8.23 (1H, d, *J* = 7.8 Hz), 7.74-7.81 (2H, m), 7.65 (1H, m), 5.66 (1H, s), 4.30 (2H, q, *J* = 7.1 Hz), 1.32 (3H, t, *J* = 7.1 Hz).

^{13}C (100.6 MHz, CDCl$_3$): δ 163.5, 147.3, 134.5, 131.5, 130.6, 126.0, 125.2, 114.4, 63.8, 41.2, 13.8.

HRMS (ES^+): $C_{11}H_{10}NaN_2O_4^+$ ($[M+Na]^+$) requires 257.0538; found 257.0541.

Methyl 2-(nitrophenyl)-2-phenylacetate **74**

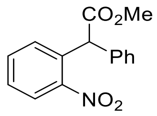

A solution of methyl phenylacetate (0.56 mL, 4.0 mmol) in THF (20 mL) was added to a solution of KHMDS (0.5 M in toluene, 16.8 mL, 8.40 mmol) in THF (20 mL) and stirred at -78 °C for 10 min. 1-Fluoro-2-nitrobenzene (0.42 mL, 4.0 mmol) was added dropwise, and the mixture was stirred at -60 °C for 30 min. The solution was allowed to warm to 0 °C, and H_2O (10 mL) was added. The mixture was extracted with EtOAc. Purification by flash pressure column chromatography (10% Et_2O/petroleum ether) afforded ester **74** as a pale yellow oil (335 mg, 1.24 mmol, 31%).

^{1}H NMR (400 MHz, $CDCl_3$): δ 7.56 (1H, dd, *J* = 7.9 Hz, 1.3 Hz), 6.95-7.14 (6H, m), 6.72 (1H, dt, *J* = 7.9 Hz, 1.3 Hz), 6.58 (1H, dt, *J* = 7.9 Hz, 1.2 Hz), 5.75 (1H, app. s), 3.31 (3H, s).

^{13}C (100.6 MHz, $CDCl_3$): δ 172.1, 149.9, 137.5, 134.6, 133.0, 132.0, 130.0, 129.6, 128.3, 128.2, 125.2, 54.0, 52.4.

HRMS (ES^+): $C_{15}H_{13}NaNO_4^+$ ($[M+Na]^+$) requires 294.0742; found 294.0745.

2-(2-Nitrophenyl)-2-(thiazol-2-yl)acetonitrile **75**

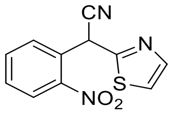

Thiazole **75** was prepared according to a modified literature procedure.[9] A solution of 2-nitrophenylacetonitrile (800 mg, 4.93 mmol) in DMSO (8 mL) was added dropwise to a suspension of 2-chlorothiazole (0.42 mL, 4.9 mmol) and Cs_2CO_3 (3.54 g, 10.9 mmol) in DMSO (8 mL) and stirred at 80 °C for 16 h. The mixture was allowed to cool to rt, and NH_4Cl (saturated aq.) was added. The mixture was extracted with CH_2Cl_2. The combined organic extracts were washed three times with H_2O, then brine, dried over Na_2SO_4, filtered, and concentrated. Purification by flash pressure column chromatography (20% EtOAc/petroleum ether) afforded nitrile **75** as an orange solid (344 mg, 1.40 mmol, 28%).

^{1}H NMR (400 MHz, CDCl$_3$): δ 8.18 (1H, dd, *J* = 7.8 Hz, 1.2 Hz), 7.97 (1H, dd, *J* = 7.8 Hz, 1.3 Hz), 7.74-7.83 (2H, m), 7.63 (1H, td, *J* = 7.8 Hz, 1.3 Hz), 7.39 (1H, d, *J* = 3.4 Hz), 6.61 (1H, s).

^{13}C NMR (100.6 MHz, CDCl$_3$): δ 161.3, 147.1, 143.6, 134.6, 131.2, 130.5, 128.2, 125.9, 121.2, 116.7, 37.2.

HRMS (ES$^+$): $[C_{11}H_7O_2Na^{32}S]^+$ ([M+Na]$^+$) requires 268.0151; found 268.0151.

MP: 40-41 °C.

(*E*)-(((5'-Bromopyridin-3'-yl)methylene)amino)phenyl-2-phenylacetonitrile **76**

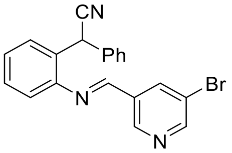

5-Bromo-3-pyridinecarboxaldehyde (800 mg, 4.30 mmol) was added to a suspension of aniline **20** (597 mg, 2.87 mmol) and $MgSO_4$ (1.73 g, 14.3 mmol) in dry toluene (25 mL). The mixture was stirred at rt for 2 h, then filtered and concentrated. The crude product was triturated with ice-cooled Et_2O to afford imine **76** as a yellow solid (1.02 g, 2.71 mmol, 94%).

^{1}H NMR (400 MHz, CDCl$_3$): δ 8.85 (1H, d, *J* = 1.7 Hz), 8.74 (1H, d, *J* = 2.2 Hz), 8.33-8.39 (2H, m), 7.45 (1H, dd, *J* = 7.6 Hz, 1.5 Hz), 7.35 (1H, td, *J* = 7.7 Hz, 1.5 Hz), 7.19-7.33 (6H, m, *Ph*), 7.05 (1H, dd, *J* = 7.7 Hz, 1.2 Hz), 5.83 (1H, s).

^{13}C NMR (100.6 MHz, CDCl$_3$): δ 156.1, 153.4, 148.9, 147.8, 137.2, 135.8, 132.9, 131.2, 129.7, 129.0, 128.9, 128.0, 127.9, 127.7, 121.4, 119.8, 117.8, 37.7.

HRMS (ES$^+$): $C_{20}H_{14}{}^{79}BrN_3Na^+$ ([M+Na]$^+$) requires 398.0263; found 398.0265. $C_{20}H_{14}{}^{81}BrN_3Na^+$ ([M+Na]$^+$) requires 400.0244; found 400.0244.

MP: 115-118 °C.

(*E*)-2-(2-((3-Bromobenzylidene)amino)phenyl)-2-phenylacetonitrile **77**

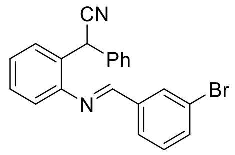

3-Bromobenzaldehyde (0.25 mL, 2.1 mmol) was added to a suspension of aniline **20** (370 mg, 1.78 mmol) and $MgSO_4$ (1.07 g, 8.89 mmol) in dry toluene (12 mL). The mixture was stirred at rt for 16 h, then filtered and concentrated. Purification by flash pressure column chromatography (8-15% Et_2O/petroleum ether) afforded imine **77** as a yellow solid (497 mg, 1.32 mmol, 74%).

^{1}H NMR (400 MHz, C_6D_6): δ 7.78 (1H, t, *J* = 1.4 Hz), 7.55 (1H, s), 7.48 (1H, dd, *J* = 7.6 Hz, 1.4 Hz), 7.41 (1H, dt, *J* = 7.8 Hz, 1.4 Hz), 7.21-7.27 (3H, m), 6.85-7.01 (5H, m), 6.74 (1H, t, *J* = 7.8 Hz), 6.53 (1H, dd, *J* = 7.6 Hz, 1.4 Hz), 5.66 (1H, s).

^{13}C NMR (100.6 MHz, C_6D_6): δ 159.3, 149.0, 138.6, 137.2, 134.9, 132.6, 132.2, 130.8, 129.8, 129.4, 129.2, 128.5, 128.1, 127.8, 127.8, 123.6, 120.4, 118.5, 38.5.

HRMS (ES$^+$): $C_{21}H_{16}{}^{79}BrN_2{}^+$ ($[M+H]^+$) requires 375.0491; found 375.0488. $C_{21}H_{16}{}^{81}BrN_2{}^+$ ($[M+H]^+$) requires 377.0471; found 377.0467.

MP: 123-126 °C.

(*E*)-2-(2-(((5-Bromofuran-2-yl)methylene)amino)phenyl)-2-phenylacetonitrile **78**

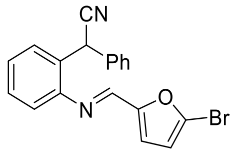

5-Bromo-2-furaldehyde (333 mg, 1.90 mmol) was added to a suspension of aniline **20** (330 mg, 1.58 mmol) and $MgSO_4$ (954 mg, 7.92 mmol) in CH_2Cl_2 (12 mL). The mixture was stirred at rt for 16 h, then filtered and concentrated. Purification by flash pressure column chromatography (20% Et_2O/petroleum ether) afforded imine **78** as a yellow oil (398 mg, 1.09 mmol, 69%).

^{1}H NMR (400 MHz, C_6D_6): δ 7.41-7.48 (2H, m), 7.29-7.35 (2H, m), 6.86-6.99 (5H, m), 6.47 (1H, dd, *J* = 7.3 Hz, 1.9 Hz), 6.28 (1H, d, *J* = 3.4 Hz), 5.84-5.91 (2H, m).

^{13}C NMR (100.6 MHz, C_6D_6): δ 154.9, 148.7, 147.1, 137.4, 132.7, 129.7, 129.4, 129.2, 128.6, 127.8, 127.4, 120.6,

118.0, 117.8, 114.7, 38.1.ǂ

ǂ 1 peak obscured.

HRMS (ES$^+$): $C_{19}H_{13}{}^{79}BrNaN_2O^+$ ([M+Na]$^+$) requires 387.0104; found 387.0096. $C_{19}H_{13}{}^{81}BrNaN_2O^+$ ([M+Na]$^+$) requires 389.0083; found 389.0074.

(*E*)-2-(2-(((4-Bromofuran-2-yl)methylene)amino)phenyl)-2-phenylacetonitrile **79**

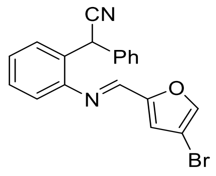

Imine **79** was prepared according to a modified literature procedure.[10] Pyrrolidine (14 µL, 0.17 mmol) was added to a stirred slurry of aniline **20** (350 mg, 1.68 mmol), 4-bromo-2-furaldehyde (382 mg, 2.18 mmol), and 3 Å molecular sieves (1.7 g) in CH_2Cl_2 (10 mL). The mixture was stirred at 50 °C for 18 h, then filtered through Celite™, eluted with CH_2Cl_2, and concentrated. Purification by flash pressure column chromatography (10-45% Et_2O/petroleum ether) afforded imine **79** as an orange oil (359 mg, 0.98 mmol, 59%).

^{1}H NMR (400 MHz, C_6D_6): δ 7.41-7.47 (2H, m), 7.29-7.34 (2H, m), 6.86-6.98 (6H, m), 6.54 (1H, s), 6.43 (1H, m), 5.89 (1H, s).

^{13}C NMR (100.6 MHz, C_6D_6): δ 153.6, 148.4, 147.5, 144.3, 137.3, 132.8, 129.7, 129.4, 129.2, 128.5, 128.0, 120.5, 118.0, 117.8, 102.7, 38.0.ǂ

ǂ 1 peak obscured.

HRMS (ES$^+$): $C_{19}H_{13}{}^{79}BrNaN_2O^+$ ([M+Na]$^+$) requires 387.0104; found 387.0095. $C_{19}H_{13}{}^{81}BrNaN_2O^+$ ([M+Na]$^+$) requires 389.0083; found 389.0074.

(*E*)-2-(2-(((5-Bromopyridin-3-yl)methylene)amino)phenyl)-2-phenylacetonitrile **80**

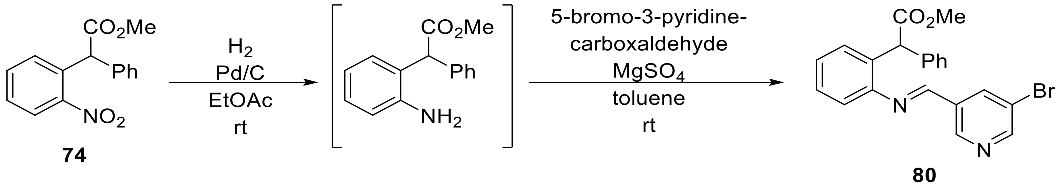

Pd/C (wet degussa type, 16 mg) was added to a solution of ester **74** (160 mg, 0.59 mmol) in EtOAc (6 mL). The suspension was degassed 3 times with hydrogen using a pump-flood procedure and placed under hydrogen for 2.5 h. The mixture was filtered through Celite™, eluted with EtOAc, and concentrated at 26 °C. The resulting residue was dissolved in toluene (6 mL), and $MgSO_4$ (355 mg, 2.95 mmol) and 5-Bromo-3-pyridinecarboxaldehyde (121 mg, 0.65 mmol) were added. The mixture was stirred at rt for 16 h, then filtered and concentrated. Purification by flash pressure column chromatography (17% EtOAc/petroleum ether) afforded imine **80** as a yellow oil (220 mg, 0.54 mmol, 91%).

^{1}H NMR (400 MHz, C_6D_6): δ 8.63 (1H, d, *J* = 2.0 Hz), 8.59 (1H, d, *J* = 2.0 Hz), 8.12 (1H, t, *J* = 2.0 Hz), 7.59 (1H, s), 7.34-7.44 (3H, m), 6.98-7.14 (5H, m), 6.67 (1H, dd, *J* = 7.6 Hz, 1.5 Hz), 5.74 (1H, s), 3.32 (3H, s).

^{13}C NMR (100.6 MHz, C_6D_6): δ 173.3, 155.6, 153.5, 149.5, 149.4, 138.7, 137.4, 135.6, 133.9, 130.0, 129.8, 129.3, 128.6, 127.9, 127.8, 121.9, 117.7, 53.6, 52.1.

HRMS (ES$^+$): $C_{21}H_{17}{}^{79}BrNaN_2O_2{}^+$ ($[M+Na]^+$) requires 431.0371; found 431.0364.

(E)-2-(2-(((5-(benzyloxy)pyridin-3-yl)methylene)amino)phenyl)-2-phenylacetonitrile **13**

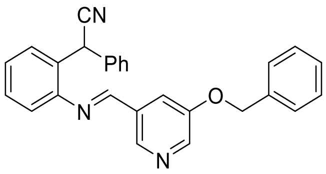

A solution of 5-(benzyloxy)nicotinaldehyde (4.86 g, 22.8 mmol) in toluene (25 mL) was added to a slurry of aniline **20** (4 g, 19 mmol) and $MgSO_4$ (11.4 g, 95 mmol) in toluene (75 mL). The mixture was stirred at rt for 16 h, filtered, and concentrated. Purification by flash pressure column chromatography (30% EtOAc/petroleum ether) afforded imine **13** as a pale yellow solid (6.05 g, 15 mmol, 79%).

^{1}H NMR (500 MHz, C_6D_6): δ 8.59 (1H, d, *J* = 2.9 Hz), 8.41 (1H, d, *J* = 1.6 Hz), 7.74 (1H, dd, *J* = 2.9 Hz, 1.6 Hz), 7.63

(1H, s), 7.43 (1H, dd, *J* = 7.5 Hz, 1.7 Hz), 7.18-7.24 (4H, m), 7.13 (2H, app. t, *J* = 7.6 Hz), 7.05 (1H, t, *J* = 7.4 Hz), 6.84-7.00 (5H, m), 6.50 (1H, dd, *J* = 7.8 Hz, 1.4 Hz), 5.65 (1H, s), 4.72 (1H, d, *J* = 12.0 Hz), 4.67 (1H, d, *J* = 12.0 Hz).

^{13}C NMR (126 MHz, C_6D_6): δ 157.6, 155.4, 148.5, 144.7, 142.9, 136.8, 136.5, 132.4, 131.8, 129.5, 129.1, 128.9, 128.6, 128.5, 127.7, 127.5, 120.0, 118.2, 117.9, 70.3, 38.3.‡

‡ 2 peaks obscured.

HRMS (ES$^+$): $[C_{27}H_{22}ON_3]^+$ ($[M+H]^+$) requires 404.1757; found 404.1759.

MP: 78-80 °C.

(E)-2-(3-(((5-bromopyridin-3-yl)methylene)amino)pyridin-2-yl)-2-phenylacetonitrile **82**

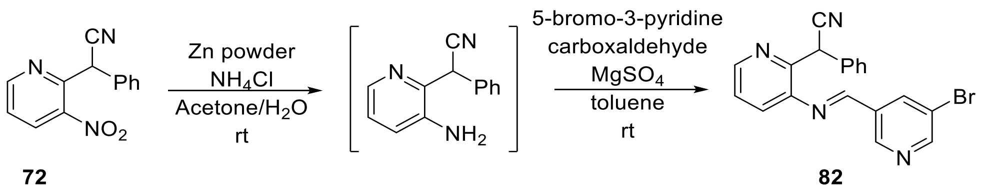

72 **82**

Zn powder (2.73 g, 41.8 mmol) and NH_4Cl (3.35 g, 62.7 mmol) were added to a solution of nitrile **72** (1.0 g, 4.2 mmol) in 5:1 acetone/H_2O (78 mL) and stirred at rt for 4.5 h. The mixture was filtered through Celite, and the filtrate was partitioned between EtOAc and H_2O. The organic layer was washed with brine, dried over Na_2SO_4, filtered, and concentrated. The remaining residue (250 mg) was dissolved in toluene (10 mL). 5-Bromopyridinecarboxaldehyde (289 mg, 1.55 mmol) and $MgSO_4$ (719 mg, 5.97 mmol) were added, and the suspension was stirred at rt for 24 h. The mixture was filtered and concentrated. The crude product was triturated three times with ice-cooled Et_2O to afford imine **82** as a yellow solid (265 mg, 0.70 mmol, 59%, contains minor impurities).

^{1}H NMR (400 MHz, $CDCl_3$): δ 8.69 (1H, d, *J* = 2.0 Hz), 8.61 (1H, d, *J* = 2.0 Hz), 8.36 (1H, dd, *J* = 4.8 Hz, 1.6 Hz), 8.21 (1H, t, *J* = 2.0 Hz), 8.16 (1H, s), 7.17-7.25 (3H, m), 7.11-7.16 (1H, dd, *J* = 7.8 Hz, 4.8 Hz), 6.98-7.10 (3H, m), 5.74 (1H, s).

^{13}C NMR (101 MHz, $CDCl_3$): δ 158.0, 153.8, 150.1, 149.0, 148.4, 143.4, 137.4, 134.7, 132.4, 128.9, 128.1, 127.8, 125.5, 124.4, 121.5, 118.9, 41.1.

HRMS (ES$^+$): $[C_{19}H_{14}N_4{}^{79}Br]^+$ ($[M+H]^+$) requires 377.0396; found 377.0400. $[C_{19}H_{14}N_4{}^{81}Br]^+$ ($[M+H]^+$) requires 379.0376; found 379.0379.

MP: 116-118 °C.

(2*RS*, 3*SR*)-2-(5'-Bromopyridin-3'-yl)-3-phenylindoline-3-carbonitrile **83** and (2*RS*, 3*RS*)-2-(5'-bromopyridin-3'-yl)-3-phenylindoline-3-carbonitrile **84**

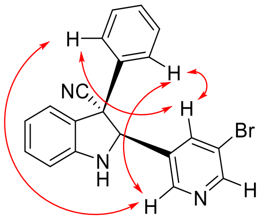

83

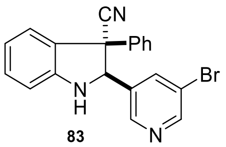

83

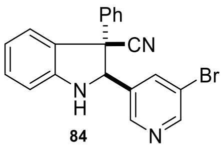

84

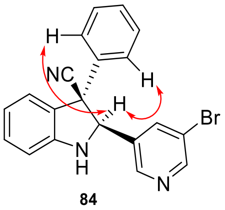

84

Indolines **83** and **84** were prepared according to general procedure 1, using KO^{t}Bu (656 mg, 5.85 mmol) and imine **76** (1.10 g, 2.92 mmol) in toluene (50 mL). The reaction mixture was stirred at 0 °C for 20 min. Purification by flash pressure column chromatography (18% EtOAc/petroleum ether) afforded separately indoline **83** as a colorless solid (338 mg, 0.90 mmol, 31%) and indoline **84** as a colorless solid (580 mg, 1.54 mmol, 53%).

N.B. Stereochemistry assigned by nOe analysis, with red arrows indicating through-space interactions.

(2*RS*,3*SR*)-2-(5-Bromopyridin-3-yl)-3-phenylindoline-3-carbonitrile **83**

^{1}H NMR (500 MHz, C_6D_6): δ 8.46 (1H, d, *J* = 2.0 Hz), 8.36 (1H, d, *J* = 2.0 Hz), 7.16 (1H, app. s), 7.05 (1H, td, *J* = 7.7 Hz, 1.2 Hz), 6.99 (1H, app. d, *J* = 7.7 Hz), 6.69-6.75 (3H, m), 6.60-6.68 (3H, m), 6.41 (1H, d, *J* = 7.7 Hz), 4.69 (1H, d, *J* = 3.4 Hz), 2.72 (1H, d, *J* = 3.4 Hz, N*H*).

^{13}C NMR (126 MHz, C_6D_6): δ 151.4, 150.4, 147.8, 137.8, 134.7, 134.4, 130.7, 128.9, 128.9, 128.7, 128.2, 126.1, 121.8, 121.5, 120.7, 111.0, 71.9, 56.0.

HRMS (ES$^+$): $[C_{20}H_{14}{}^{79}BrN_3Na]^+$ ($[M+Na]^+$) calc. 398.0263; found 398.0270. $[C_{20}H_{14}{}^{81}BrN_3Na]^+$ ($[M+Na]^+$) calc. 400.0244; found 400.0250.

MP: 133-136 °C

(2*RS*,3*RS*)-2-(5-Bromopyridin-3-yl)-3-phenylindoline-3-carbonitrile **84**

^{1}H NMR (500 MHz, C_6D_6): δ 8.65 (1H, app. s), 8.08 (1H, app. s), 7.91 (1H, t, *J* = 1.9 Hz), 7.17-7.20 (2H, m), 6.95-7.04 (4H, m), 6.67 (1H, app. d, *J* = 7.7 Hz), 6.60 (1H, td, *J* = 7.7 Hz, 0.9 Hz), 6.43 (1H, app. d, *J* = 7.7 Hz), 4.21 (1H, d, *J* = 3.2 Hz), 2.93 (1 H, d, *J* = 3.2 Hz, N*H*).

^{13}C NMR (126 MHz, C_6D_6): δ 152.3, 150.6, 148.1, 138.5, 137.4, 134.6, 130.7, 129.5, 129.2, 128.7, 127.5, 126.2, 121.5, 121.4, 118.8, 111.2, 75.4, 58.9.

HRMS (ES$^+$): $[C_{20}H_{14}{}^{79}BrN_3Na]^+$ ($[M+Na]^+$) calc. 398.0263; found 398.0270. $[C_{20}H_{14}{}^{81}BrN_3Na]^+$ ($[M+Na]^+$) calc. 400.0244; found 400.0252.

MP: 69-70 °C.

Ethyl (2*RS*, 3*RS*)-2-(5-bromopyridin-3-yl)-3-cyanoindoline-3-carboxylate **85**

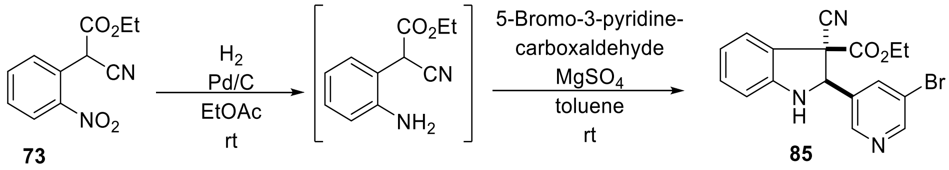

Pd/C (wet degussa type, 10% palladium by weight, 80 mg) was added to a solution of nitrile **73** (800 mg, 3.42 mmol) in EtOAc (20 mL). The suspension was degassed 3 times with hydrogen using a pump-flood procedure and placed under hydrogen for 45 min. The mixture was filtered through Celite™, eluted with EtOAc, and concentrated at 26 °C. The resulting residue was dissolved in toluene (24 mL), and $MgSO_4$ (2.06 g, 17.1 mmol) and 5-Bromo-3-pyridinecarboxaldehyde (699 mg, 3.76 mmol) were added. The mixture was stirred at rt for 16 h, then filtered and concentrated. Purification by flash pressure column chromatography (28% EtOAc/petroleum ether) afforded imine **85** as a pale yellow solid (889 mg, 2.38 mmol, 68%, contains minor impurities).

N.B. Stereochemistry assigned by X-ray crystallography. Major diastereomer (as drawn):

^{1}H NMR (400 MHz, C_6D_6): δ 8.69 (1H, d, *J* = 1.9 Hz), 8.60 (1H, d, *J* = 2.2 Hz), 7.85 (1H, app. t, *J* = 2.0 Hz), 7.09 (1H, d, *J* = 7.9 Hz), 6.98 (1H, td, *J* = 7.7 Hz, 1.2 Hz), 6.62 (1H, td, *J* = 7.7 Hz, 1.0 Hz), 6.34 (1H, d, *J* = 7.9 Hz), 4.66 (1H, d, *J* = 3.4 Hz), 3.35 (2H, q, *J* = 7.1 Hz), 2.72 (1H, d, *J* = 3.4 Hz, N*H*), 0.42 (3H, t, *J* = 7.1 Hz).

^{13}C NMR (101 MHz, C_6D_6): δ 164.7, 152.0, 150.8, 148.1, 138.0, 134.4, 131.2, 125.5, 124.6, 121.4, 121.1, 118.4, 111.5, 70.2, 63.2, 57.5, 13.7.

HRMS (ES$^+$): $[C_{17}H_{14}{}^{79}BrNaN_3O_2]^+$ ($[M+Na]^+$) calc. 394.0167; found 394.0169. $[C_{17}H_{14}{}^{81}BrNaN_3O_2]^+$ ($[M+Na]^+$) calc. 396.0142; found 396.0148.

MP: 100-102 °C.

Methyl (2*RS*,3*SR*)-2-(5-bromopyridin-3-yl)-3-phenylindoline-3-carboxylate **86** and methyl (2*RS*,3*RS*)-2-(5-bromopyridin-3-yl)-3-phenylindoline-3-carboxylate **87**

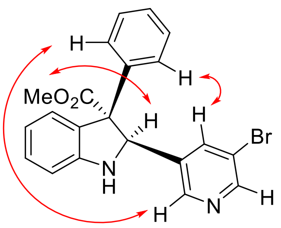

86

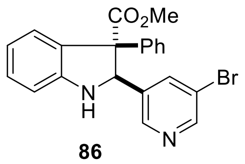

86

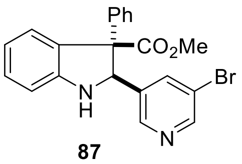

87

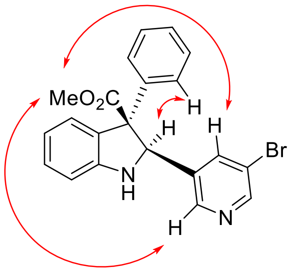

87

Indolines **86** and **87** were prepared according to general procedure 1, using KO^{t}Bu (72 mg, 0.65 mmol) and imine **80** (240 mg, 0.59 mmol) in toluene (11 mL). The reaction mixture was stirred at 0 °C for 20 min. Purification by flash pressure column chromatography (18% EtOAc/petroleum ether) afforded separately indoline **86** as a colorless solid (76 mg, 0.19 mmol, 31%) and indoline **87** as a colorless solid (66 mg, 0.16 mmol, 27%).

N.B. Stereochemistry assigned by nOe analysis, with red arrows indicating through-space interactions.

Methyl (2*RS*,3*SR*)-2-(5-bromopyridin-3-yl)-3-phenylindoline-3-carboxylate **86**

^{1}H NMR (500 MHz, C_6D_6): δ 8.68 (1H, d, *J* = 1.7 Hz), 8.52 (1H, d, *J* = 2.4 Hz), 7.51 (1H, d, *J* = 7.6 Hz), 7.40 (1H, t, *J* = 1.9 Hz), 7.16 (1H, obscured), 6.83 (1H, td, *J* = 7.6 Hz, 1.0 Hz), 6.69-6.79 (3H, m), 6.50 (1H, d, *J* = 7.7 Hz), 6.44 (2H, m), 5.55 (1H, d, *J* = 3.6 Hz), 3.26 (3H, s), 2.91 (1H, d, *J* = 3.3 Hz, N*H*).

^{13}C NMR (126 MHz, C_6D_6): δ 173.7, 151.2, 150.4, 149.1, 138.8, 138.8, 137.4, 130.0, 128.6, 128.4, 127.9, 127.4, 120.5, 120.5, 110.5, 67.8, 67.2, 52.6.‡

‡ 1 peak obscured.

HRMS (ES$^+$): $[C_{21}H_{18}{}^{79}BrN_2O_2]^+$ ($[M+H]^+$) calc. 409.0546; found 409.0538. $[C_{21}H_{18}{}^{81}BrN_2O_2]^+$ ($[M+H]^+$) calc. 411.0526; found 411.0516.

MP: 118-121 °C.

Methyl (2*RS*,3*RS*)-2-(5-bromopyridin-3-yl)-3-phenylindoline-3-carboxylate **87**

^{1}H NMR (500 MHz, C_6D_6): δ 8.64 (1H, d, *J* = 2.2 Hz), 8.34 (1H, d, *J* = 1.7 Hz), 7.79 (1H, t, *J* = 2.1 Hz), 7.37 (1H, d, *J* = 7.4 Hz), 7.32 (2H, m), 7.01-7.11 (4H, m), 6.77 (1H, td, *J* = 7.5 Hz, 1.0 Hz), 6.46 (1H, d, *J* = 7.7 Hz), 4.57 (1H, d, *J* = 2.4 Hz), 3.06 (1H, app. s, N*H*), 2.89 (3H, s).

^{13}C NMR (126 MHz, C_6D_6): δ 171.0, 151.0, 150.9, 148.4, 142.8, 138.7, 137.7, 129.7, 129.5, 129.1, 128.5, 128.4, 128.4, 120.7, 120.5, 110.6, 73.0, 67.4, 51.8.

HRMS (ES$^+$): $[C_{21}H_{18}{}^{79}BrN_2O_2]^+$ ($[M+H]^+$) calc. 409.0546; found 409.0536. $[C_{21}H_{18}{}^{81}BrN_2O_2]^+$ ($[M+H]^+$) calc. 411.0526; found 411.0515.

MP: 72-76 °C.

(2*RS*,3*RS*)-2-(5-Bromopyridin-3-yl)-3-(thiazol-2-yl)indoline-3-carbonitrile **88**

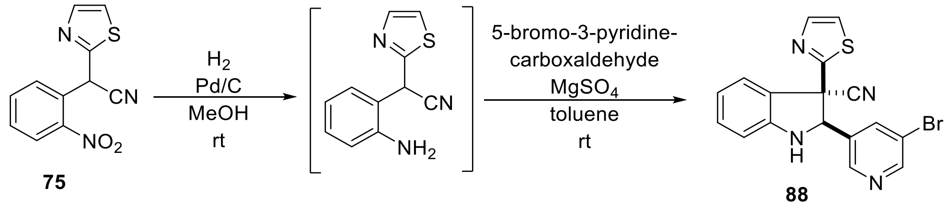

Pd/C (wet Degussa type, 30 mg) was added to a solution of nitrile **75** (300 mg, 1.22 mmol) in MeOH (12 mL). The suspension was first degassed 3 times with N_2 and then 3 times with H_2 using a pump-flood procedure. The mixture was stirred under a H_2 atmosphere for 18 h, then flushed with N_2 before being filtered through Celite^{TM}, eluted with EtOAc, and concentrated. Purification by flash pressure column chromatography (25% EtOAc/petroleum ether) afforded an orange solid (150 mg, impure), which was re-dissolved in toluene (6 mL). $MgSO_4$ (414 mg, 3.44 mmol) and 5-bromo-3-pyridinecarboxaldehyde (166 mg, 0.89 mmol) were added, and the mixture was stirred at rt for 48 h. The mixture was filtered and concentrated. Purification by flash pressure column chromatography (40% EtOAc/petroleum ether) afforded indoline **88** as a yellow solid (62 mg, 0.16 mmol, 13%).

N.B. Stereochemistry assigned by analogy to indolines **83** and **84**.

^{1}H NMR (500 MHz, C_6D_6): δ 8.54 (1H, d, *J* = 2.4 Hz), 8.46 (1H, d, *J* = 1.9 Hz), 7.42 (1H, t, *J* = 2.1 Hz), 7.07 (1H, d, *J*

= 3.3 Hz), 6.98-7.05 (2H, m), 6.62 (1H, td, *J* = 7.6 Hz, 0.9 Hz), 6.38 (1H, d, *J* = 7.9 Hz), 6.16 (1H, d, *J* = 3.3 Hz), 4.72 (1H, d, *J* = 3.5 Hz), 2.79 (1H, d, *J* = 3.3 Hz, N*H*).

^{13}C NMR (126 MHz, C_6D_6): δ 164.5, 151.7, 150.3, 147.9, 143.8, 137.6, 133.8, 131.5, 127.4, 126.2, 121.6, 121.3, 120.8, 119.4, 111.5, 72.0, 54.7.

HRMS (ES$^+$): $[C_{17}H_{12}N_4{}^{79}Br^{32}S]^+$ ($[M+H]^+$) requires 382.9961; found 382.9963. $[C_{17}H_{12}N_4{}^{81}Br^{32}S]^+$ ($[M+H]^+$) requires 384.9940; found 384.9941.

MP: 152-155 °C.

(2*RS*,3*SR*)-2-(3-Bromophenyl)-3-phenylindoline-3-carbonitrile **89** and (2*RS*,3*RS*)-2-(3-bromophenyl)-3-phenylindoline-3-carbonitrile **90**

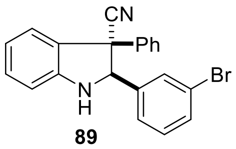

89

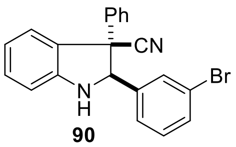

90

Indolines **89** and **90** were prepared according to general procedure 1, using KO^{t}Bu (278 mg, 2.48 mmol) and imine **77** (465 mg, 1.24 mmol) in toluene (20 mL). The reaction mixture was stirred at 0 °C for 15 min. Purification by flash pressure column chromatography (13% Et_2O/petroleum ether) afforded separately indoline **89** as a colorless solid (118 mg, 0.31 mmol, 25%) and indoline **90** as a colorless solid (194 mg, 0.52 mmol, 42%).

N.B. Stereochemistry assigned by analogy to previously reported similar indolines.[6]

(2*RS*,3*SR*)-2-(3-Bromophenyl)-3-phenylindoline-3-carbonitrile **89**

^{1}H NMR (500 MHz, C_6D_6): δ 2.78 (1H, d, *J* = 3.3 Hz), 4.84 (1H, d, *J* = 3.3 Hz), 6.41 (1H, app. d, *J* = 7.6 Hz), 6.57 (1H, t, *J* = 7.8 Hz), 6.65 (1H, td, *J* = 7.5 Hz, 1.0 Hz), 6.68-6.82 (5H, m), 6.90 (1H, app. d, *J* = 7.8 Hz), 7.02-7.09 (3H, m), 7.14 (1H, t, *J* = 1.8 Hz).

^{13}C NMR (125.75 MHz, C_6D_6): δ 55.9, 74.4, 110.8, 121.5, 122.0, 122.8, 126.1, 126.8, 128.4, 128.7, 128.7, 129.0, 129.9, 130.5, 131.3, 131.8, 135.1, 139.1, 150.7.

HRMS (ESI$^+$, m/z): $[C_{21}H_{16}{}^{79}BrN_2]^+$ ($[M+H]^+$) calc. 375.0491; found 375.0485. $[C_{21}H_{16}{}^{81}BrN_2]^+$ ($[M+H]^+$) calc. 377.0471; found 377.0465.

MP: 140-143 °C.

(2*RS*,3*RS*)-2-(3-bromophenyl)-3-phenylindoline-3-carbonitrile **90**

^{1}H NMR (500 MHz, C_6D_6): δ 3.01 (1H, d, *J* = 3.0 Hz), 4.35 (1H, d, *J* = 3.0 Hz), 6.42 (1H, app. d, *J* = 7.9 Hz), 6.60 (1H, dt, *J* = 7.5 Hz, 0.9 Hz), 6.69-6.78 (2H, m), 6.97-7.06 (5H, m), 7.22-7.30 (3H, m), 7.40 (1H, t, *J* = 1.8 Hz).

^{13}C NMR (125.75 MHz, C_6D_6): δ 58.9, 77.8, 111.0, 118.9, 121.2, 123.0, 126.2, 127.0, 128.0, 128.4, 129.0, 129.4, 130.5, 130.6, 131.7, 132.6, 138.3, 139.6, 150.3.

HRMS (ESI$^+$, m/z): $[C_{21}H_{16}{}^{79}BrN_2]^+$ ($[M+H]^+$) calc. 375.0491; found 375.0484. $[C_{21}H_{16}{}^{81}BrN_2]^+$ ($[M+H]^+$) calc. 377.0471; found 377.0463.

MP: 120-122 °C.

(2*SR*,3*RS*)-2-(4-Bromofuran-2-yl)-3-phenylindoline-3-carbonitrile **92**

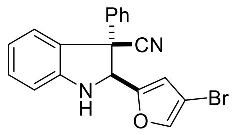

Indoline **92** was prepared according to general procedure 1, using KO^{t}Bu (109 mg, 0.98 mmol) and imine **79** (325 mg, 0.89 mmol) in toluene (12 mL). The reaction mixture was stirred at 15 °C for 15 min. Purification by flash pressure column chromatography (15% Et_2O/petroleum ether) afforded indoline **92** as a colorless solid (240 mg, 0.65 mmol, 73%).

N.B. Stereochemistry assigned by analogy to previously reported similar indolines.[6]

^{1}H NMR (400 MHz, C_6D_6): δ 3.10 (1H, d, *J* = 2.5 Hz, N*H*), 4.47 (1H, d, *J* = 2.5 Hz), 6.33 (1H, app. s), 6.38 (1H, app. d, *J* = 7.8 Hz), 6.57 (1H, td, *J* = 7.5 Hz, 0.8 Hz), 6.80 (1H, d, *J* = 7.5 Hz), 6.87 (1H, d, *J* = 0.7 Hz), 6.94-7.06 (4H, m), 7.32-7.41 (2H, m).

^{13}C NMR (100.6 MHz, C_6D_6): δ 57.7, 71.1, 101.1, 110.9, 112.9, 119.0, 121.3, 126.1, 127.7, 127.8, 129.0, 129.5, 130.6, 139.2, 141.9, 150.3, 153.4.

HRMS (ESI$^+$, m/z): $[C_{19}H_{13}{}^{79}BrNaN_2O]^+$ ($[M+Na]^+$) calc. 387.0104; found 387.0097. $[C_{19}H_{13}{}^{81}BrNaN_2O]^+$ ($[M+Na]^+$) calc. 389.0083; found 389.0076.

MP: 137-140 °C.

(2*R*,3*R*)-2-(5-(Benzyloxy)pyridin-3-yl)-3-phenylindoline-3-carbonitrile **(*R,R*)-15** and (2*S*,3*S*)-2-(5-(benzyloxy)pyridin-3-yl)-3-phenylindoline-3-carbonitrile **(*S,S*)-15**

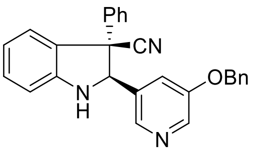

(*R,R*)-15

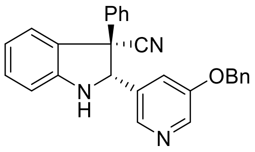

(*S,S*)-15

(2*R*,3*R*)-2-(5-(Benzyloxy)pyridin-3-yl)-3-phenylindoline-3-carbonitrile **(*R,R*)-15**

Catalyst **16** (70 mg, 0.11 mmol) was added to a solution of imine **13** (880 mg, 2.18 mmol) in toluene (33 mL), and the suspension was stirred at -30 °C for 30 min. $CsOH{\cdot}H_2O$ (732 mg, 4.36 mmol) was added and stirred at -30 °C for 16 h. The mixture was diluted with EtOAc (40 mL), and NH_4Cl (sat. aq., 40 mL) was added. The mixture was allowed to warm to rt, the organic and aqueous layers were separated, and the aqueous layer was extracted with EtOAc. The combined organic layers were washed with brine, dried over Na_2SO_4, filtered, and concentrated. Purification by flash pressure column chromatography (35% EtOAc/petroleum ether) afforded indoline **(*R,R*)-15** as a colorless solid (787 mg, 1.95 mmol, d.r. 7:1, e.r. 17:83, 89%). Resolution by semi-preparatory chiral HPLC (YMC chiral amylose SA, 5% ^{i}PrOH, 95% hexane, 5.0 mL/min) afforded indoline **(*R,R*)-15** as a colorless solid (d.r. > 20:1, e.r. > 99:1).

(2*S*,3*S*)-2-(5-(Benzyloxy)pyridin-3-yl)-3-phenylindoline-3-carbonitrile **(*S,S*)-15**

Indoline **(*S,S*)-15** was prepared in an analogous manner to indoline **(*R,R*)-15** using catalyst **14** (47 mg, 0.074 mmol), imine **13** (600 mg, 1.49 mmol), and $CsOH{\cdot}H_2O$ (500 mg, 2.97 mmol) in toluene (19 mL). The reaction mixture was stirred at -30 °C for 16 h. Purification by flash pressure column chromatography (35% EtOAc/petroleum ether) afforded indoline **(*S,S*)-15** as a colorless solid (538 mg, 1.33 mmol, d.r. 10:1, e.r. 88:12, 90%). Resolution by semi-preparatory chiral HPLC (YMC chiral amylose SA, 5% ^{i}PrOH, 95% hexane, 5.0 mL/min) afforded indoline **(*S,S*)-15** as a colorless solid (d.r. > 20:1, e.r. > 99:1).

N.B. Stereochemistry assigned by X-ray crystallography of **17**.

^{1}H (400 MHz, C_6D_6): δ 8.58 (1H, d, *J* = 2.9 Hz), 7.91 (1H, d, *J* = 1.4 Hz), 7.63 (1H, t, *J* = 2.0 Hz), 7.27 (2H, m), 6.93-7.14 (7H, m), 6.74 (1H, d, *J* = 7.6 Hz), 6.61 (1H, t, *J* = 7.6 Hz), 6.44 (1H, d, *J* = 8.0 Hz), 4.73 (1H, d, *J* = 11.8 Hz), 4.69 (1H, d, *J* = 11.8 Hz), 4.40 (1H, d, *J* = 3.2 Hz), 3.06 (1H, d, *J* = 3.0 Hz, N*H*).ǂ

^{13}C (101 MHz, C_6D_6): δ 155.3, 150.6, 142.2, 140.1, 137.5, 136.8, 133.0, 130.3, 129.1, 128.7, 128.6, 127.5, 125.9, 121.0, 120.5, 119.0, 110.8, 75.7, 70.4, 58.6.‡

ǂ 2 peaks obscured.

‡ 3 peaks obscured.

HRMS (ES$^+$): $[C_{27}H_{22}ON_3]^+$ ($[M+H]^+$) requires 404.1757; found 404.1763.

MP: 48-50 °C.

$[\alpha]_D^{25.0}$ (*R*,*R*) = +26 (*c* = 0.1, $CHCl_3$); $[\alpha]_D^{25.0}$ (*S*,*S*) = -19 (*c* = 0.1, $CHCl_3$).

Chiral HPLC: (Chiralpak IA, 10% ^{i}PrOH, 90% hexane, 1.0 mL/min, λ = 254 nm, 20 μL injection): τ_R (*R*,*R*) = 33.2 min, τ_R (*S*,*S*) = 27.5 min.

(2*RS*, 3*SR*)-1-Acetyl-2-(5'-bromopyridin-3'-yl)-3-phenylindoline-3-carbonitrile **93**

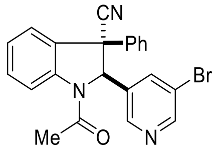

Indoline **93** was prepared according to general procedure 2, using indoline **83** (250 mg, 0.66 mmol), acetyl chloride (57 μL, 0.80 mmol), and pyridine (64 μL, 0.80 mmol) in CH_2Cl_2 (13 mL). The reaction mixture was stirred at rt for 15 min. Purification by flash pressure column chromatography (30% EtOAc/petroleum ether) afforded indoline **93** as a colorless solid (270 mg, 0.65 mmol, 98%).

^{1}H NMR (500 MHz, C_6D_6, 348 K): δ 1.64 (3H, s), 5.62 (1H, s), 6.71-6.84 (6H, m), 6.88 (2H, m), 7.08 (1H, t, *J* = 7.8 Hz), 7.87 (1H, s), 8.10 (1H, br. s), 8.23 (1H, d, *J* = 2.2 Hz).

^{13}C NMR (125.75 MHz, C_6D_6, 348 K): δ 24.0, 55.8, 72.9, 117.6, 120.8, 121.4, 125.7, 126.8, 129.1, 129.1, 129.5, 131.6, 133.7, 134.3, 137.4, 144.6, 146.9, 151.3, 168.2.ǂ

ł 1 peak obscured.

HRMS (ESI$^+$, m/z): $[C_{22}H_{16}{}^{79}BrN_3NaO]^+$ ($[M+Na]^+$) calc. 440.0369; found 440.0371. $[C_{22}H_{16}{}^{81}BrN_3NaO]^+$ ($[M+Na]^+$) calc. 442.0349; found 442.0353.

MP: 126-128 °C.

(2*RS*, 3*RS*)-1-Acetyl-2-(5'-bromopyridin-3'-yl)-3-phenylindoline-3-carbonitrile **94**

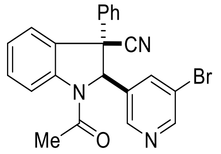

Indoline **94** was prepared according to general procedure 2, using indoline **84** (400 mg, 1.06 mmol), acetyl chloride (91 µL, 1.3 mmol), and pyridine (103 µL, 1.28 mmol) in CH_2Cl_2 (20 mL). The reaction mixture was stirred at rt for 15 min. Purification by flash pressure column chromatography (30% EtOAc/petroleum ether) afforded indoline **94** as a colorless solid (379 mg, 0.91 mmol, 85%).

^{1}H NMR (500 MHz, C_6D_6, 348 K): δ 1.49 (3H, s), 5.18 (1H, br. s), 6.74 (1H, t, *J* = 7.8 Hz), 6.96-7.03 (4H, m), 7.06 (1H, t, *J* = 7.8 Hz), 7.17 (2H, m), 7.55 (1H, app. t, *J* = 1.8 Hz), 8.04 (1H, br. s), 8.41 (1H, d, *J* = 1.6 Hz), 8.56 (1H, d, *J* = 2.2 Hz).

^{13}C NMR (125.75 MHz, C_6D_6, 348 K): δ 23.7, 57.9, 74.0, 117.5, 118.9, 121.8, 126.1, 126.2, 126.5, 129.5, 129.8, 130.2, 131.4, 136.0, 136.8, 140.8, 143.4, 147.1, 152.9, 167.9.

HRMS (ESI$^+$, m/z): $[C_{22}H_{16}{}^{79}BrN_3NaO]^+$ ($[M+Na]^+$) calc. 440.0369; found 440.0370. $[C_{22}H_{16}{}^{81}BrN_3NaO]^+$ ($[M+Na]^+$) calc. 442.0349; found 442.0350.

MP: 90-94 °C.

(2*RS*,3*SR*)-1-Acetyl-3-phenyl-2-(5'-((trimethylsilyl)ethynyl)pyridin-3'-yl)indoline-3-carbonitrile **95**

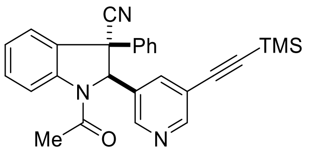

A suspension of indoline **93** (120 mg, 0.29 mmol), $PdCl_2(PPh_3)_2$ (10 mg, 0.014 mmol), and CuI (3.0 mg, 0.014 mmol) in diisopropylamine (4 mL) was degassed and put under an Ar atmosphere before ethynyltrimethylsilane (50 µL, 0.34 mmol) was added. The mixture was stirred at 65 °C for 5.5 h, then filtered through Celite™. The filtrate was washed with water, then brine, dried over $MgSO_4$, filtered, and concentrated. Purification by flash pressure column chromatography (30% EtOAc/petroleum ether) afforded alkyne **95** as a colorless solid (121 mg, 0.28 mmol, 96%).

^{1}H NMR (500 MHz, C_6D_6, 348 K): δ 0.12 (9H, s), 1.60 (3H, s), 5.62 (1H, s), 6.67-6.83 (5H, m), 6.85-6.94 (3H, m), 7.06 (1H, td, *J* = 7.8 Hz, 1.4 Hz), 7.85 (1H, s), 8.15 (1H, br. s), 8.43 (1H, s).

^{13}C NMR (125.75 MHz, C_6D_6, 348 K): δ 0.02, 24.0, 55.9, 73.3, 99.5, 102.0, 117.7, 120.5, 121.5, 125.6, 126.7, 129.1, 129.2, 129.4, 131.5, 132.2, 133.9, 137.1, 144.8, 148.2, 153.0, 168.2.‡

‡ 1 peak obscured.

HRMS (ESI$^+$, m/z): $[C_{27}H_{25}N_3NaOSi]^+$ ($[M+Na]^+$) calc. 458.1659; found 458.1655.

MP: 125-127 °C.

(2*RS*,3*RS*)-1-Acetyl-3-phenyl-2-(5-((trimethylsilyl)ethynyl)pyridin-3-yl)indoline-3-carbonitrile **96**

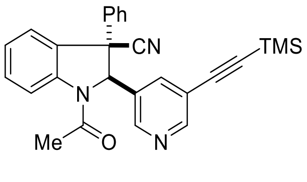

A suspension of indoline **94** (150 mg, 0.36 mmol), $PdCl_2(PPh_3)_2$ (13 mg, 0.018 mmol), and CuI (3.5 mg, 0.018 mmol) in diisopropylamine (4 mL) was degassed and put under an Ar atmosphere before ethynyltrimethylsilane (61 µL, 0.43 mmol) was added. The mixture was stirred at 65 °C for 5.5 h, then filtered through Celite™. The filtrate was washed three times with water, then with brine, dried over $MgSO_4$, filtered, and concentrated. Purification by flash pressure column chromatography (30% EtOAc/petroleum ether) afforded alkyne **96** as a colorless solid (141 mg, 0.32 mmol, 89%).

^{1}H NMR (500 MHz, C_6D_6, 348 K): δ 0.14 (9H, s), 1.47 (3H, s), 5.16 (1H, s), 6.71 (1H, t, *J* = 7.8 Hz), 6.95-7.01 (4H, m), 7.03 (1H, t, *J* = 7.8 Hz), 7.13-7.18 (2H, obscured), 7.59 (1H, s), 8.10 (1H, br. s), 8.47 (1H, app. s), 8.78 (1H, app. s).

^{13}**C NMR (125.75 MHz, C_6D_6, 348 K):** δ 0.04, 23.6, 58.0, 74.5, 99.9, 102.2, 117.6, 118.9, 126.0, 126.2, 126.4, 129.4, 129.7, 130.2, 131.3, 134.0, 136.6, 141.1, 143.6, 148.0, 154.6, 167.9.ƚ

ƚ 1 peak obscured.

HRMS (ESI$^+$, m/z): $[C_{27}H_{26}N_3OSi]^+$ ($[M+H]^+$) calc. 436.1840; found 436.1850.

MP: 155-157 °C.

(2*RS*,3*SR*)-1-Acetyl-2-(5-ethynylpyridin-3-yl)-3-phenylindoline-3-carbonitrile **97**

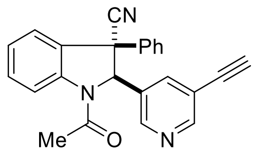

TBAF (1.0 M in THF, 0.3 mL, 0.3 mmol) was added dropwise to a solution of alkyne **95** (110 mg, 0.25 mmol) in THF (5 mL) at 0 °C and stirred at rt for 15 min. The mixture was diluted with EtOAc (10 mL) and washed three times with water, then with brine, dried over $MgSO_4$, filtered, and concentrated. Purification by flash pressure column chromatography (36% EtOAc/petroleum ether) afforded alkyne **97** as a colorless oil (90 mg, 0.25 mmol, 99%).

^{1}**H NMR (500 MHz, C_6D_6, 348 K):** δ 1.60 (3H, s), 2.58 (1H, s), 5.61 (1H, s), 6.70-6.81 (5H, m), 6.83 (1H, app. s), 6.89 (2H, m), 7.07 (1H, t, *J* = 7.8 Hz), 7.86 (1H, app. s), 8.17 (1H, br. s), 8.36 (1H, app. s).

^{13}**C NMR (125.75 MHz, C_6D_6, 348 K):** δ 24.0, 55.9, 73.2, 80.3, 81.6, 117.7, 119.5, 121.4, 125.6, 126.7, 129.1, 129.1, 129.4, 131.5, 132.3, 133.8, 137.5, 144.8, 148.5, 153.0, 168.2.ƚ

ƚ 1 peak obscured.

HRMS (ESI$^+$, m/z): $[C_{24}H_{17}N_3NaO]^+$ ($[M+Na]^+$) calc. 386.1264; found 386.1268.

(2*RS*,3*RS*)-1-acetyl-2-(5-ethynylpyridin-3-yl)-3-phenylindoline-3-carbonitrile **98**

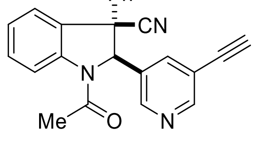

TBAF (1.0 M in THF, 0.35 mL, 0.35 mmol) was added dropwise to a solution of alkyne **96** (120 mg, 0.28 mmol) in THF (5 mL) at 0 °C and stirred at rt for 15 min. The mixture was diluted with EtOAc (10 mL) and washed three times with water, then with brine, dried over $MgSO_4$, filtered, and concentrated. Purification by flash pressure column chromatography (36% EtOAc/etroleum ether) afforded alkyne **98** as a colorless oil (94 mg, 0.26 mmol, 92%).

^{1}H NMR (500 MHz, C_6D_6, 348 K): δ 1.45 (3H, s), 2.66 (1H, s), 5.16 (1H, s), 6.73 (1H, t, *J* = 7.6 Hz), 6.95-7.03 (4H, m), 7.05 (1H, t, *J* = 7.9 Hz), 7.14-7.20 (2H, obscured), 7.53 (1H, app. s), 8.12 (1H, br. s), 8.46 (1H, app. s), 8.71 (1H, app. s).

^{13}C NMR (125.75 MHz, C_6D_6, 348 K): δ 23.6, 58.0, 74.4, 80.5, 82.1, 117.5, 118.9, 120.5, 126.0, 126.2, 126.4, 129.4, 129.7, 130.2, 131.3, 133.9, 137.0, 141.0, 143.6, 148.3, 154.5, 167.9.

HRMS (ESI$^+$, m/z): $[C_{24}H_{17}N_3NaO]^+$ ($[M+Na]^+$) calc. 386.1264; found 386.1265.

Ethyl (2*RS*,3*RS*)-1-acetyl-2-(5-bromopyridin-3-yl)-3-cyanoindoline-3-carboxylate **99**

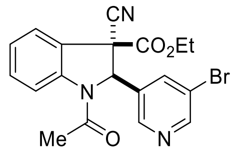

Indoline **99** was prepared according to general procedure 2, using indoline **85** (840 mg, 2.26 mmol), acetyl chloride (0.20 mL, 2.7 mmol), and pyridine (0.20 mL, 2.7 mmol) in CH_2Cl_2 (35 mL). The reaction mixture was stirred at rt for 15 min. Purification by flash pressure column chromatography (36% EtOAc/petroleum ether) afforded indoline **99** as a colorless solid (819 mg, 1.97 mmol, 87%).

^{1}H NMR (500 MHz, C_6D_6, 348 K): δ 0.63 (3H, t, *J* = 7.1 Hz), 1.55 (3H, s), 3.48 (2H, q, *J* = 7.1 Hz), 5.73 (1H, s), 6.77 (1H, t, *J* = 7.6 Hz), 7.00 (1H, t, *J* = 7.8 Hz), 7.36 (1H, t, *J* = 1.8 Hz), 7.72 (1H, br. s), 8.25 (1H, d, *J* = 1.6 Hz), 8.44 (1H, d, *J* = 1.8 Hz).ł

^{13}C NMR (125.75 MHz, C_6D_6, 348 K): δ 13.3, 13.4, 55.6, 63.6, 69.5, 116.7, 117.5, 120.8, 125.0, 125.2, 127.1, 131.4, 133.6, 137.2, 143.5, 147.2, 152.1, 163.9, 167.3.

ł 1 peak obscured.

HRMS (ESI$^+$, m/z): $[C_{19}H_{16}{}^{79}BrNaN_3O_3]^+$ ($[M+Na]^+$) calc. 436.0273; found 436.0276. $[C_{19}H_{16}{}^{81}BrNaN_3O_3]^+$ ($[M+Na]^+$) calc. 438.0248; found 438.0257.

MP: 91-93 °C.

Methyl (2*RS*,3*SR*)-1-acetyl-2-(5-bromopyridin-3-yl)-3-phenylindoline-3-carboxylate **100**

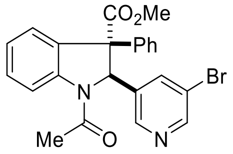

Indoline **100** was prepared according to general procedure 2, using indoline **86** (65 mg, 0.16 mmol), acetyl chloride (14 µL, 0.19 mmol), and pyridine (15 µL, 0.19 mmol) in CH_2Cl_2 (5 mL). The reaction mixture was stirred at rt for 15 min. Purification by flash pressure column chromatography (30% EtOAc/petroleum ether) afforded indoline **100** as a colorless paste (72 mg, 0.16 mmol, 99%).

^{1}H NMR (500 MHz, C_6D_6, 348 K): δ 1.83 (3H, s), 3.19 (3H, s), 6.31 (1H, s), 6.72-6.77 (2H, m), 6.77-6.81 (3H, m), 6.90 (1H, td, *J* = 7.7 Hz, 1.0 Hz), 7.00 (1H, app. s), 7.15 (1H, obscured), 7.30 (1H, d, *J* = 7.7 Hz), 8.02 (1H, d, *J* = 1.4 Hz), 8.24 (1H, d, *J* = 2.1 Hz), 8.41 (1H, br. s).

^{13}C NMR (125.75 MHz, C_6D_6, 348 K): δ 23.7, 52.8, 67.9, 69.6, 117.5, 120.4, 124.4, 127.9, 128.1, 128.6, 129.5, 130.4, 136.2, 136.9, 137.1, 144.7, 147.1, 150.3, 167.9, 172.6.[ⱡ]

ⱡ 1 peak obscured.

HRMS (ES$^+$): $[C_{23}H_{20}{}^{79}BrO_3N_2]^+$ ($[M+H]^+$) calc. 451.0652; found 451.0651. $[C_{23}H_{20}{}^{81}BrO_3N_2]^+$ ($[M+H]^+$) calc. 453.0631; found 453.0627.

(2*RS*,3*RS*)-1-acetyl-2-(5-bromopyridin-3-yl)-3-(thiazol-2-yl)indoline-3-carbonitrile **102**

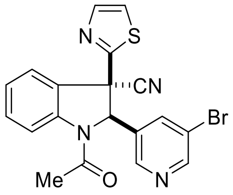

Indoline **102** was prepared according to general procedure 2, using indoline **88** (52 mg, 0.14 mmol), acetyl chloride (24 µL, 0.33 mmol), and pyridine (26 µL, 0.33 mmol) in CH_2Cl_2 (3 mL). The reaction mixture was stirred

at rt for 1 h. $NaHCO_3$ (saturated aq., 3 mL) was added, and the mixture was stirred at rt for a further 10 min. Purification by flash pressure column chromatography (42% EtOAc/petroleum ether) afforded indoline **102** as a pale yellow solid (55 mg, 0.13 mmol, 92%).

^{1}H NMR (400 MHz, $CDCl_3$): δ 2.24 (3H, br. s), 6.08 (1H, br. s), 7.24 (1H, d, *J* = 3.3 Hz), 7.28-7.35 (2H, m), 7.49 (1H, d, *J* = 7.6 Hz), 7.61 (1H, td, *J* = 7.9 Hz, 1.2 Hz), 7.80 (1H, d, *J* = 3.3 Hz), 8.07 (1H, d, *J* = 1.9 Hz), 8.36 (1H, br. s), 8.43 (1H, d, *J* = 1.3 Hz).

^{13}C NMR (100.6 MHz, $CDCl_3$): δ 24.2, 53.2, 71.5, 117.7, 118.6, 120.5, 121.9, 125.8, 126.4, 132.2, 132.4, 136.9, 143.3, 143.9, 146.3, 151.3, 162.2, 168.0.[ǂ]

ǂ 1 peak obscured.

HRMS (ES$^+$): $[C_{19}H_{14}{}^{79}BrN_4O^{32}S]^+$ ($[M+H]^+$) calc. 425.0066; found 425.0066. $[C_{19}H_{14}{}^{81}BrN_4O^{32}S]^+$ ($[M+H]^+$) calc. 427.0046; found 427.0045.

MP: 188-190 °C.

(2*RS*,3*SR*)-1-Acetyl-2-(3-bromophenyl)-3-phenylindoline-3-carbonitrile **103**

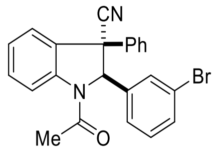

Indoline **103** was prepared according to general procedure 2, using indoline **89** (100 mg, 0.27 mmol), acetyl chloride (23 µL, 0.32 mmol), and pyridine (26 µL, 0.32 mmol) in CH_2Cl_2 (6 mL). The reaction mixture was stirred at rt for 25 min. Purification by flash pressure column chromatography (15% EtOAc/petroleum ether) afforded indoline **103** as a colorless solid (97 mg, 0.23 mmol, 87%).

^{1}H NMR (500 MHz, C_6D_6, 348 K): δ 1.62 (3H, s), 5.52 (1H, s), 6.33 (1H, t, *J* = 7.8 Hz), 6.40 (1H, d, *J* = 7.8 Hz), 6.61 (1H, br. s), 6.73-6.88 (6H, m), 6.93 (2H, app. d, *J* = 7.3 Hz), 7.13 (1H, td, *J* = 7.7 Hz, 1.4 Hz), 8.48 (1H, br. s).

^{13}C (125.75 MHz, C_6D_6, 348 K): δ 24.1, 56.2, 75.1, 117.9, 121.7, 123.1, 125.5, 125.7, 126.6, 128.8, 128.8, 129.2, 130.3, 131.3, 131.4, 131.8, 134.2, 139.2, 145.2, 168.6.[‡]

‡ 1 peak obscured.

HRMS (ESI^+, m/z): [$C_{23}H_{17}{}^{79}BrNaN_2O$]$^+$ ([M+Na]$^+$) calc. 439.0422; found 439.0415.

MP: 125-128 °C.

(2*RS*,3*RS*)-1-Acetyl-2-(3-bromophenyl)-3-phenylindoline-3-carbonitrile **104**

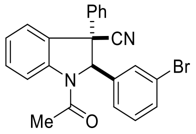

Indoline **104** was prepared according to general procedure 2, using indoline **90** (170 mg, 0.45 mmol), acetyl chloride (39 μL, 0.54 mmol), and pyridine (44 μL, 0.54 mmol) in CH_2Cl_2 (10 mL). The reaction mixture was stirred at rt for 25 min. Purification by flash pressure column chromatography (16% EtOAc/petroleum ether) afforded indoline **104** as a colorless solid (179 mg, 0.43 mmol, 95%).

^{1}H NMR (500 MHz, C_6D_6, 348 K): δ 1.48 (3H, s), 5.08 (1H, s), 6.68 (1H, t, *J* = 7.9 Hz), 6.77 (1H, t, *J* = 7.6 Hz), 6.90 (1H, d, *J* = 7.9 Hz), 6.96-7.04 (3H, m), 7.06 (1H, d, *J* = 7.6 Hz), 7.13 (1H, t, *J* = 7.9 Hz), 7.19 (1H, d, *J* = 8.2 Hz), 7.20-7.25 (2H, m), 7.37 (1H, s), 8.42 (1H, br. s).

^{13}C NMR (125.75 MHz, C_6D_6, 348 K): δ 23.8, 58.4, 76.4, 117.7, 118.8, 124.0, 125.3, 125.9, 126.2, 126.4, 129.3, 130.0, 130.2, 130.5, 131.2, 131.6, 133.2, 141.0, 141.6, 144.1, 168.3.

HRMS (ESI^+, m/z): $C_{23}H_{17}{}^{79}BrNaN_2O^+$ ([M+Na]$^+$) calc. 439.0422; found 439.0414.

MP: 149-152 °C.

(2*RS*,3*RS*)-1-Acetyl-2-(5-bromofuran-2-yl)-3-phenylindoline-3-carbonitrile **105** and (2*RS*,3*SR*)-1-acetyl-2-(5-bromofuran-2-yl)-3-phenylindoline-3-carbonitrile **106**

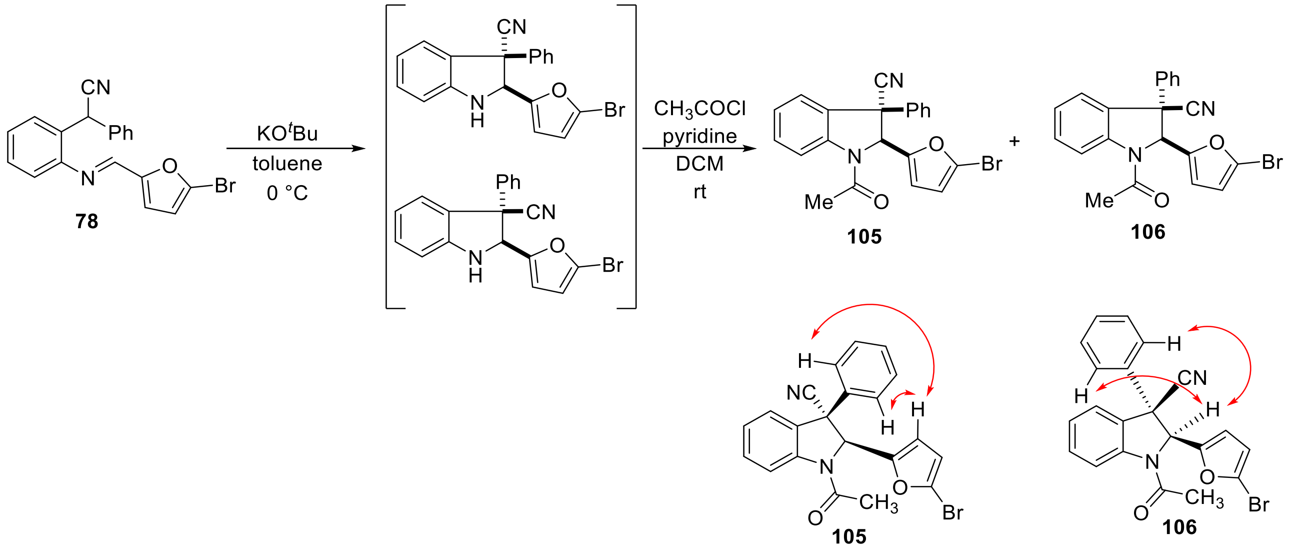

KO^{t}Bu (68 mg, 0.60 mmol) was added to a solution of imine **78** (200 mg, 0.55 mmol) in toluene (8 mL) and stirred at 0 °C for 10 min. NH_4Cl (saturated aq., 6 mL) was added, and the mixture was extracted with EtOAc. The combined organic extracts were washed with brine, dried over $MgSO_4$, filtered. The resulting residue (200 mg) was dissolved in CH_2Cl_2 (10 mL) with pyridine (53 µL, 0.66 mmol). Acetyl chloride (47 µL, 0.66 mmol) was added dropwise, and the solution was stirred at rt for 10 min. $NaHCO_3$ (saturated aq., 5 mL) was added, and the mixture was extracted with CH_2Cl_2. The combined organic extracts were washed with brine, dried over $MgSO_4$, filtered, and concentrated. Purification by flash pressure column chromatography (3.5% Et_2O/toluene) afforded separately indoline **106** as a colorless oil (115 mg, 0.28 mmol, 51%) and indoline **105** as a colorless oil (71 mg, 0.17 mmol, 32%).

N.B. Stereochemistry assigned by nOe analysis, with red arrows indicating through-space interactions.

(2*RS*,3*RS*)-1-Acetyl-2-(5-bromofuran-2-yl)-3-phenylindoline-3-carbonitrile **106**

^{1}H NMR (500 MHz, C_6D_6, 348 K): δ 1.53 (3H, s), 5.27 (1H, br. s), 5.80 (1H, d, *J* = 3.4 Hz), 5.85 (1H, d, *J* = 3.4 Hz), 6.77 (1H, td, *J* = 7.6 Hz, 0.8 Hz), 6.93-7.01 (3H, m), 7.06-7.13 (2H, m), 7.18-7.23 (2H, m), 8.30 (1H, br. s).

^{13}C NMR (125.75 MHz, C_6D_6, 125.75): δ 23.2, 56.9, 70.9, 111.9, 113.4, 118.0, 118.7, 123.9, 125.7, 126.2, 126.3, 129.4, 130.0, 130.1, 131.0, 140.5, 143.4, 153.2, 168.0.

HRMS (ESI$^+$, m/z): $[C_{21}H_{16}{}^{79}BrN_2O_2]^+$ ($[M+H]^+$) calc. 407.0390; found 407.0382. $[C_{21}H_{16}{}^{81}BrN_2O_2]^+$ ($[M+H]^+$) calc. 409.0369; found 409.0361.

(2*RS*,3*SR*)-1-Acetyl-2-(5-bromofuran-2-yl)-3-phenylindoline-3-carbonitrile **105**

^{1}H NMR (500 MHz, C_6D_6, 348 K): δ 1.63 (3H, s), 5.43 (1H, d, *J* = 3.2 Hz), 5.48 (1H, d, *J* = 3.2 Hz), 5.60 (1H, s), 6.74 (1H, td, *J* = 7.7 Hz, 0.9 Hz), 6.82 (1H, app. d, *J* = 7.7 Hz), 6.91-7.02 (3H, m), 7.10 (1H, td, *J* = 7.7 Hz, 1.2 Hz), 7.13-7.14 (2H, m), 8.35 (1H, br. s).

^{13}C NMR (125.75 MHz, C_6D_6, 125.75): δ 23.5, 55.4, 70.1, 112.5, 112.7, 118.1, 121.0, 123.2, 125.3, 126.4, 128.9, 128.9, 129.4, 130.1, 131.2, 134.0, 144.7, 151.5, 168.2.

HRMS (ESI$^+$, m/z): $[C_{21}H_{16}{}^{79}BrN_2O_2]^+$ ($[M+H]^+$) calc. 407.0390; found 407.0382. $[C_{21}H_{16}{}^{81}BrN_2O_2]^+$ ($[M+H]^+$) calc. 409.0369; found 409.0362.

(2*SR*,3*RS*/*SR*)-1-Acetyl-2-(4-bromofuran-2-yl)-3-phenylindoline-3-carbonitrile **107**

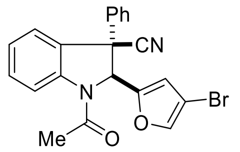

Indoline **107** was prepared according to general procedure 2, using indoline **92** (133 mg, 0.36 mmol), acetyl chloride (31 μL, 0.44 mmol), and pyridine (35 μL, 0.44 mmol) in CH_2Cl_2 (7 mL). The reaction mixture was stirred at rt for 10 min. Purification by flash pressure column chromatography (15% EtOAc/petroleum ether) afforded indoline **107** as a yellow oil (144 mg, 0.38 mmol, 97%).

^{1}H NMR (500 MHz, C_6D_6, 348 K): δ 1.52 (3H, s), 5.25 (1H, br. s), 6.08 (1H, s), 6.78 (1H, t, *J* = 7.6 Hz), 6.92 (1H, s), 6.94-7.02 (3H, m), 7.07-7.13 (2H, m), 7.20-7.25 (2H, m), 8.26 (1H, br. s).

^{13}C NMR (125.75 MHz, C_6D_6, 348 K): δ 23.2, 56.7, 70.8, 101.3, 112.9, 118.0, 118.6, 125.8, 126.1, 126.3, 128.9, 129.4, 130.2, 131.0, 140.5, 142.6, 143.3, 152.2, 167.8.

HRMS (ESI$^+$, m/z): $[C_{21}H_{16}{}^{79}BrN_2O_2]^+$ ($[M+H]^+$) calc. 407.0390; found 407.0383. $[C_{21}H_{16}{}^{81}BrN_2O_2]^+$ ($[M+H]^+$) calc. 409.0369; found 409.0362.

(2*RS*,3*RS*)-1-acetyl-2-(5-(benzyloxy)pyridin-3-yl)-3-phenylindoline-3-carbonitrile **108** and (2*RS*,3*SR*)-1-acetyl-2-(5-(benzyloxy)pyridin-3-yl)-3-phenylindoline-3-carbonitrile **109**

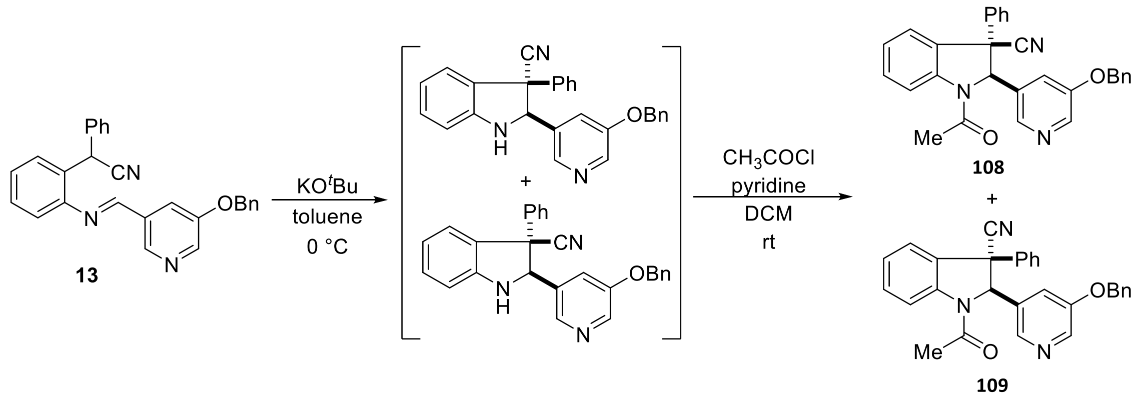

Racemic:

^{t}BuOK (706 mg, 6.29 mmol) was added to a solution of imine **13** (1.27 g, 3.15 mmol) in toluene (60 mL) and stirred at 0 °C for 25 min. NH_4Cl (saturated aq., 60 mL) was added, and the mixture was extracted 3 times with EtOAc. The combined organic extracts were washed with brine, dried over Na_2SO_4, filtered, and concentrated. Purification by flash pressure column chromatography (30% EtOAc/petroleum ether) afforded an inseparable mixture of diastereomers (853 mg, 2.11 mmol, 67%). This mixture (755 mg, 1.87 mmol) was dissolved in CH_2Cl_2 (40 mL) with pyridine (212 µL, 2.63 mmol). Acetyl chloride (181 µL, 2.53 mmol) was added dropwise, and the solution was stirred at rt for 30 min. $NaHCO_3$ (saturated aq., 40 mL) was added, and the mixture was extracted with CH_2Cl_2. The combined organic extracts were washed with brine, dried over $MgSO_4$, filtered, and concentrated. Purification by flash pressure column chromatography (15% EtOAc/petroleum ether) afforded separately diastereomers **108** as a colorless solid (376 mg, 0.84 mmol, 45%) and **109** as a colorless solid (167 mg, 0.38 mmol, 20%).

Asymmetric:

Indolines ***(R,R)*-108** and ***(S,S)*-108** were prepared according to general procedure 2 using indoline ***(R,R)*-15** (157 mg, 0.39 mmol), acetyl chloride (33 µL, 0.47 mmol), and pyridine (38 µL, 0.47 mmol) in CH_2Cl_2 (8.5 mL) and indoline ***(S,S)*-15** (216 mg, 0.54 mmol), acetyl chloride (46 µL, 0.64 mmol), and pyridine (52 µL, 0.64 mmol) in CH_2Cl_2 (10 mL). Purification by flash pressure column chromatography (50% EtOAc/petroleum ether) afforded indolines ***(R,R)*-108** (165 mg, 0.37 mmol, 95%) and ***(S,S)*-108** (230 mg, 0.52 mmol, 96%) respectively.

(2*RS*,3*RS*)-1-Acetyl-2-(5-(benzyloxy)pyridin-3-yl)-3-phenylindoline-3-carbonitrile **108**

^{1}H (500 MHz, C_6D_6, 348 K): δ 8.45 (1H, s), 8.33 (1H, br. s), 8.26 (1H, s), 7.22 (2H, app. d, *J* = 7.6 Hz), 6.97-7.13 (11H, m), 6.77 (1H, t, *J* = 7.6 Hz), 5.20 (1H, br. s), 4.50-4.61 (2H, d, *J* = 12.5 Hz), 1.48 (3H, s).

^{13}C (126 MHz, C_6D_6, 348 K): δ 168.2, 156.1, 144.0, 141.7, 141.3, 140.9, 136.8, 134.8, 131.2, 130.2, 129.9, 129.4, 129.1, 126.4, 126.3, 125.9, 119.7, 119.0, 117.7, 74.7, 71.2, 58.3, 23.7.‡

‡ 2 peaks obscured.

HRMS (ES$^+$): $[C_{29}H_{24}N_3O_2]^+$ ($[M+H]^+$) calc. 446.1863; found 446.1854.

MP: 61-64 °C.

$[\alpha]_D^{25.0}$ (*R*,*R*) = +76 (*c* = 0.1, $CHCl_3$); $[\alpha]_D^{25.0}$ (*S*,*S*) = -84 (*c* = 0.1, $CHCl_3$).

(2*RS*,3*SR*)-1-Acetyl-2-(5-(benzyloxy)pyridin-3-yl)-3-phenylindoline-3-carbonitrile **109**

^{1}H (500 MHz, C_6D_6): δ 8.61 (1H, br. s), 8.45 (1H, d, *J* = 2.7 Hz), 8.28 (1H, app. s), 7.19-7.24 (2H, m), 6.87-7.12 (11H, m), 6.70 (1H, td, *J* = 7.6 Hz, 1.0 Hz), 5.09 (1H, br. s), 4.42 (1H, d, *J* = 11.7 Hz), 4.38 (1H, d, *J* = 11.7 Hz), 1.36 (3H, br. s).

^{13}C (126 MHz, C_6D_6): δ 168.0, 155.5, 143.5, 141.1, 140.7, 140.0, 136.1, 134.4, 130.9, 129.8, 129.0, 128.7, 127.9, 126.0, 125.9, 125.7, 118.8, 118.7, 117.3, 73.9, 70.3, 57.8, 23.3.‡

‡ 2 peaks obscured.

HRMS (ES$^+$): $[C_{29}H_{24}N_3O_2]^+$ ($[M+H]^+$) calc. 446.1863; found 446.1860.

MP: 63-68 °C.

(2*RS*,3*SR*)-1-acetyl-2-(5-hydroxypyridin-3-yl)-3-phenylindoline-3-carbonitrile **110**

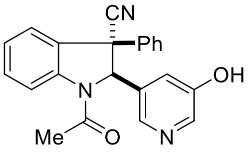

Indoline **110** was prepared according to general procedure 8, using indoline **109** (150 mg, 0.34 mmol) and Pd/C (wet degussa type, 15 mg). The reaction mixture was stirred under a H_2 atmosphere for 5.5 h. The crude residue was triturated with toluene to afford indoline **110** as a colorless solid (75 mg, 0.21 mmol, 63%).

^{1}H NMR (500 MHz, CD_3OD): δ 8.28 (1H, br. s), 7.77 (1H, d, *J* = 2.4 Hz), 7.59 (1H, td, *J* = 7.8 Hz, 1.1 Hz), 7.38 (1H, d, *J* = 1.3 Hz), 7.31 (1H, td, *J* = 7.8 Hz, 1.1 Hz), 7.19-7.28 (4H, m), 7.15 (2H, m), 6.61 (1H, app. t, *J* = 1.9 Hz), 6.30 (1H, s), 2.16 (3H, br. s).ǂ

^{13}C NMR (126 MHz, CD_3OD): δ 171.5, 155.7, 145.3, 140.0, 138.5, 134.5, 133.7, 132.4, 130.4, 129.8, 129.8, 127.6, 127.0, 122.5, 122.3, 118.8, 72.9, 56.6, 24.2.‡

ǂ O*H* peak not observed due to exchange with MeOD.

‡ 1 peak obscured.

HRMS (ES$^+$): $[C_{22}H_{18}N_3O_2]^+$ ($[M+H]^+$) calc. 356.1394; found 356.1390.

MP: 132-136 °C.

(2*RS*,3*RS*)-1-acetyl-2-(5-hydroxypyridin-3-yl)-3-phenylindoline-3-carbonitrile **17**

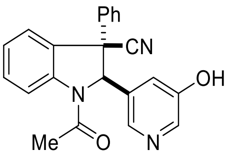

Indoline **17** was prepared according to general procedure 8, using indoline **108** (390 mg, 0.88 mmol) and Pd/C (wet degussa type, 36 mg). The reaction mixture was stirred under a H_2 atmosphere for 5 h. The crude residue was triturated with toluene to afford indoline **17** as a colorless solid (308 mg, 0.87 mmol, 98%). The non-racemic forms of indoline **17** were prepared from **(*R*,*R*)-108** and **(*S*,*S*)-108**, following an identical protocol.

^{1}H NMR (500 MHz, CD_3OD): δ 8.33 (1H, br. s), 8.15 (1H, br. s), 8.06 (1H, br. s), 7.57 (1H, t, *J* = 7.7 Hz), 7.36-7.46 (4H, m), 7.32 (1H, t, *J* = 7.7 Hz), 7.28 (2H, m), 7.05 (1H, t, *J* = 2.0 Hz), 5.75 (1H, s), 1.99 (3H, br. s).ǂ

^{13}C NMR (126 MHz, CD_3OD): δ 171.2, 156.3, 144.2, 141.0, 140.0, 139.6, 136.6, 132.2, 130.9, 130.4, 127.2, 127.2, 126.9, 121.7, 119.8, 118.5, 74.1, 58.8, 24.0.‡

ǂ O*H* peak not observed.

‡ 1 peak obscured.

HRMS (ES$^+$): $[C_{22}H_{18}N_3O_2]^+$ ($[M+H]^+$) calc. 356.1394; found 356.1389.

MP: 120-125 °C.

$[\alpha]_D^{25.0}$ (*R*,*R*) = +72 (*c* = 0.1, $CHCl_3$); $[\alpha]_D^{25.0}$ (*S*,*S*) = -73 (*c* = 0.1, $CHCl_3$).

5-((2*R*,3*R*)-1-Acetyl-3-cyano-3-phenylindolin-2-yl)pyridin-3-yl trifluoromethanesulfonate ***(R,R)*-111** and 5-((2*S*,3*S*)-1-acetyl-3-cyano-3-phenylindolin-2-yl)pyridin-3-yl trifluoromethanesulfonate ***(S,S)*-111**

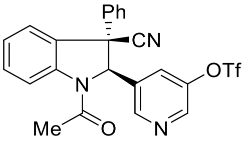

(*R*,*R*)-111

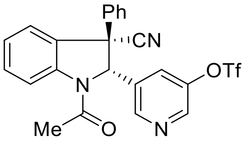

(*S*,*S*)-111

5-((2*R*,3*R*)-1-Acetyl-3-cyano-3-phenylindolin-2-yl)pyridin-3-yl trifluoromethanesulfonate ***(R,R)*-111**

Diisopropylethylamine (49 µL, 0.28 mmol) was added to a solution of indoline ***(R,R)*-17** (50 mg, 0.14 mmol) and *N*-phenyl-bis(trifluoromethylsulfonimide) (100 mg, 0.28 mmol) in CH_2Cl_2 (4 mL) at 0 °C. The reaction mixture was allowed to warm to rt and stirred for 3 h. H_2O (10 mL) was added, and the mixture was extracted with CH_2Cl_2. The combined organic extracts were washed with brine, dried over Na_2SO_4, filtered, and concentrated. Purification by flash pressure column chromatography (30% EtOAc/petroleum ether) afforded indoline ***(R,R)*-111** as a colorless solid (53 mg, 0.11 mmol, 79%).

5-((2*S*,3*S*)-1-Acetyl-3-cyano-3-phenylindolin-2-yl)pyridin-3-yl trifluoromethanesulfonate ***(S,S)*-111**

Indoline ***(S,S)*-111** was prepared in an analogous manner to indoline ***(R,R)*-111**, using indoline ***(S,S)*-17** (148 mg, 0.42 mmol), diisopropylethylamine (0.15 mL, 0.83 mmol), and *N*-phenyl-bis(trifluoromethylsulfonimide) (298 mg, 0.83 mmol) in CH_2Cl_2 (12 mL). The reaction mixture was stirred at rt for 3 h. Purification by flash

pressure column chromatography (30% EtOAc/petroleum ether) afforded indoline**(*S,S*)-111** as a colorless solid (189 mg, 0.39 mmol, 93%).

^{1}H (500 MHz, C_6D_6, 348 K): δ 8.44 (1H, app. s), 8.34 (1H, d, *J* = 2.1 Hz), 7.96 (1H, br. s), 7.24 (1H, app. s), 7.11-7.21 (2H, obscured), 7.06 (1H, t, *J* = 7.9 Hz), 6.94-7.03 (4H, m), 6.74 (1H, t, *J* = 7.6 Hz), 5.23 (1H, s), 1.50 (3H, s).

^{13}C (126 MHz, C_6D_6, 348 K): δ 167.7, 148.4, 147.4, 144.2, 143.2, 140.4, 136.2, 131.4, 130.3, 129.7, 129.6, 127.1, 126.6, 126.2, 126.2, 118.6, 117.2, 73.6, 57.7, 23.5.ǂ

ǂ CF_3 signal obscured due to insufficient signal-to-noise ratio.

^{19}F (377 MHz, C_6D_6): δ -72.7.

HRMS (ES$^+$): $[C_{23}H_{17}O_4N_3F_3{}^{32}S]^+$ ($[M+H]^+$) requires 488.0886; found 488.0887.

$[\alpha]_D^{25.0}$ (*R,R*) = +51 (*c* = 0.1, $CHCl_3$), $[\alpha]_D^{25.0}$ (*S,S*) = -60 (*c* = 0.1, $CHCl_3$).

(2*RS*,3*SR*)-1-acetyl-2-(5-bromopyridin-3-yl)-3-phenyl-2,3-dihydro-1H-pyrrolo[3,2-b]pyridine-3-carbonitrile **112**

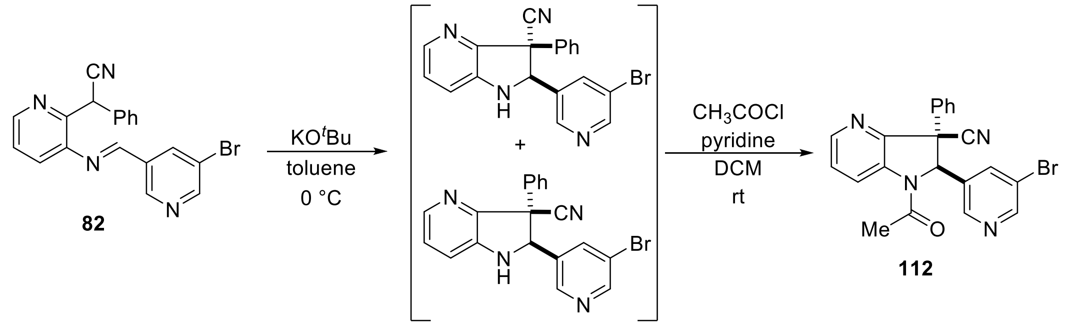

KO^{t}Bu (65 mg, 0.58 mmol) was added to a solution of imine **82** (200 mg, 0.53 mmol) in toluene and stirred at 0 °C for 20 min. NH_4Cl (saturated aq., 20 mL) was added, and the mixture was extracted with EtOAc. The combined organic extracts were washed with brine, dried over Na_2SO_4, filtered, and concentrated. Purification by flash pressure column chromatography (50% EtOAc/petroleum ether) afforded a 1:1.23 inseparable mixture of diastereoisomers (87 mg, 0.23 mmol, 44%). 60 mg (0.16 mmol) of the residue was dissolved in CH_2Cl_2 (6 mL). Pyridine (19 µL, 0.24 mmol) was added, followed by acetyl chloride (17 µL, 0.24 mmol). The mixture was stirred at rt for 2 h. $NaHCO_3$ (saturated aq., 6 mL) was added, and the mixture was extracted with CH_2Cl_2. The combined organic extracts were washed with brine, dried over Na_2SO_4, filtered and concentrated. Purification by flash pressure column chromatography afforded indoline **112** as a colorless solid (38 mg, 0.09 mmol, 57%).

N.B. Stereochemistry assigned by analogy to indolines **93** and **94**.

^{1}H NMR (500 MHz, C_6D_6, 348 K): δ 1.41 (3H, s), 5.17 (1H, s), 6.63 (1H, dd, *J* = 8.2 Hz, 4.7 Hz), 6.93-7.03 (3H, m), 7.23 (2H, app. d, *J* = 8.0 Hz), 7.48 (1H, t, *J* = 1.9 Hz), 7.98 (1H, dd, *J* = 4.7 Hz, 1.3 Hz), 8.19 (1H, br. s), 8.33 (1H, d, *J* = 1.9 Hz), 8.57 (1H, d, *J* = 1.9 Hz).

^{13}C NMR (125.75 MHz, C_6D_6, 348 K): δ 23.2, 59.4, 72.3, 117.5, 121.9, 124.3, 125.0, 126.2, 129.5, 130.3, 135.5, 136.7, 137.5, 139.8, 146.9, 147.4, 149.8, 153.1, 168.6.

HRMS (ES$^+$): $[C_{21}H_{16}{}^{79}BrN_4O]^+$ ($[M+H]^+$) calc. 419.0502; found 419.0504. $[C_{21}H_{16}{}^{81}BrN_4O]^+$ ($[M+H]^+$) calc. 421.0482; found 421.0482.

(2*RS*,3*RS*)-2-(5-bromopyridin-3-yl)-1-(2-cyanoacetyl)-3-phenylindoline-3-carbonitrile **113**

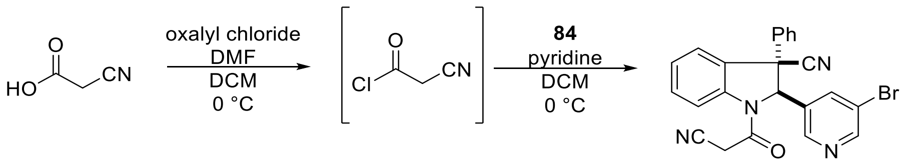

1 drop of DMF was added to a solution of cyanoacetic acid (200 mg, 2.35 mmol) in CH_2Cl_2 (8 mL), and the mixture was cooled to 0 °C. Oxalyl chloride (0.18 mL, 2.1 mmol) was added dropwise and the mixture was stirred at 0 °C for 1 h. 4 mL of the reaction mixture was transferred to a solution of indoline **84** (60 mg, 0.16 mmol) in CH_2Cl_2 (4 mL) at 0 °C, and the mixture was stirred at 0 °C for 25 min. $NaHCO_3$ (sat aq., 10 mL) was added, and the mixture was extracted with DCM. The combined organic extracts were washed with brine, dried over Na_2SO_4, filtered, and concentrated. Purification by flash pressure column chromatography (30% EtOAc/Petroleum ether) afforded indoline **113** as a colorless oil (56 mg, 0.13 mmol, 79%).

^{1}H NMR (500 MHz, C_6D_6, 348 K): δ 2.24 (1H, d, *J* = 18.5 Hz), 2.30 (1H, d, *J* = 18.5 Hz), 5.01 (1H, s), 6.70 (1H, t, *J* = 7.6 Hz), 6.93 (1H, d, *J* = 7.5 Hz), 6.95-7.04 (4H, m), 7.08 (2H, m), 7.42 (1H, app. s), 7.84 (1H, br. s), 8.28 (1H, d, *J* = 1.6 Hz), 8.55 (1H, d, *J* = 1.9 Hz).

^{13}C NMR (125.75 MHz, C_6D_6, 348 K): δ 26.6, 58.2, 73.6, 112.5, 117.5, 118.4, 122.0, 126.1, 126.7, 127.3, 129.9, 130.4, 131.5, 134.5, 136.6, 139.9, 142.2, 146.7, 153.5, 160.0.†

ǂ 1 peak obscured.

HRMS (ES$^+$): $[C_{23}H_{16}{}^{79}BrN_4O]^+$ ($[M+H]^+$) calc. 443.0502; found 443.0502. $[C_{23}H_{16}{}^{81}BrN_4O]^+$ ($[M+H]^+$) calc. 445.0482; found 445.0480.

MP: 93-95 °C.

2-(1,3-Dioxoisoindolin-2-yl)acetyl chloride **114**

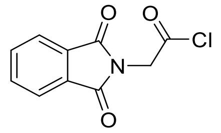

Acyl chloride **114** was prepared according to a literature procedure.[11] DMF (1 drop) was added to a suspension of *N*-phthaloylglycine (1.00 g, 4.87 mmol) in CH_2Cl_2 (35 mL) and cooled to 0 °C. Oxalyl chloride (0.82 mL, 9.8 mmol) was added dropwise, and the mixture was stirred at 0 °C for 1.5 h. The reaction mixture was concentrated at rt to afford acyl chloride **114** as a yellow solid (1.12 g, 5.01 mmol, quant). The spectral data matched those reported in the literature.

^{1}H NMR (400 MHz, $CDCl_3$): δ 4.79 (2H, s), 7.75 (2H, m), 7.88 (2H, m).

^{13}C NMR (100.6 MHz, $CDCl_3$): δ 47.7, 124.1, 131.7, 134.8, 166.7, 169.2.

(2*RS*,3*RS*)-2-(5-Bromopyridin-3-yl)-1-(2-(1,3-dioxoisoindolin-2-yl)acetyl)-3-phenylindoline-3-carbonitrile **115**

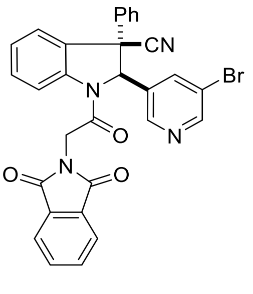

Indoline **115** was prepared according to general procedure 2, using indoline **84** (80 mg, 0.21 mmol), acyl chloride **114** (119 mg, 0.53 mmol), and pyridine (43 µL, 0.53 mmol) in CH_2Cl_2 (6.5 mL). The reaction mixture was stirred at rt for 1 h. Purification by flash pressure column chromatography (30-35% EtOAc/petroleum ether) afforded indoline **115** as a colorless solid (75 mg, 0.13 mmol, 63%).

^{1}H NMR (400 MHz, dmso-D$_6$): δ 4.14 (1H, d, *J* = 17.2 Hz), 4.92 (1H, d, *J* = 17.2 Hz), 6.22 (1H, s), 7.32-7.42 (3H, m), 7.43-7.50 (2H, m), 7.50-7.57 (2H, m), 7.60 (1H, t, *J* = 7.8 Hz), 7.82-7.93 (4H, m), 8.00 (1H, app. s), 8.11 (1H, d, *J* = 7.8 Hz), 8.57 (1H, d, *J* = 1.6 Hz), 8.79 (1H, d, *J* = 1.9 Hz).

^{13}C NMR (100.6 MHz, dmso-D$_6$): δ 56.4, 69.5, 116.4, 117.8, 119.8, 122.8, 125.1, 125.3, 125.8, 127.7, 128.4, 128.6, 129.1, 130.6, 131.1, 134.2, 134.4, 136.4, 138.1, 140.9, 145.8, 150.9, 164.3, 166.5.

HRMS (ES$^+$): $[C_{30}H_{20}O_3N_4{}^{79}Br]^+$ ($[M+H]^+$) requires 563.0713; found 563.0714. $[C_{30}H_{20}O_3N_4{}^{81}Br]^+$ ($[M+H]^+$) requires 565.0693; found 565.0692.

MP: 250-252 °C.

(2*RS*,3*RS*)-2-(5-Bromopyridin-3-yl)-1-(3-(3-methyl-3H-diazirin-3-yl)propanoyl)-3-phenylindoline-3-carbonitrile **116**

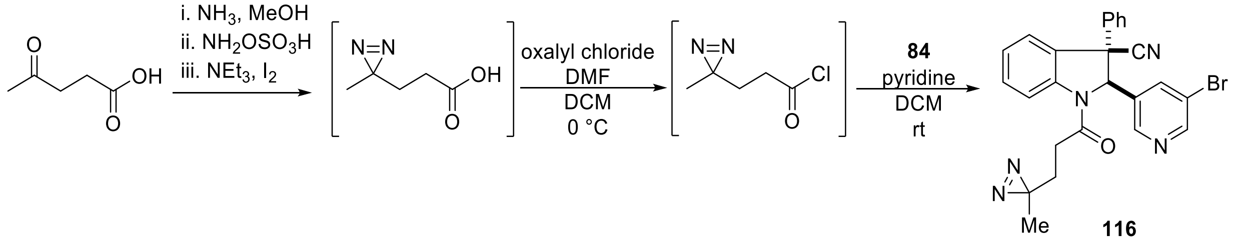

Diazirine **116** was prepared according to a modified literature procedure.[12] A solution of NH_3 in MeOH (7 N, 5.0 mL, 35 mmol) was added dropwise to a solution of levulinic acid (0.44 mL, 4.3 mmol) in MeOH (1 mL) at 0 °C and stirred for 3 h. A solution of hydroxylamine *O*-sulfonic acid (510 mg, 4.95 mmol) in MeOH (1.5 mL) was added and stirred at rt for 16 h. The excess NH_3 was removed by blowing the mixture down with N_2. The white precipitate was filtered off and the filtrate concentrated. The remaining residue was dissolved in MeOH (0.5 mL) and cooled to 0 °C. NEt_3 (0.90 mL, 6.5 mmol) was added and stirred at 0 °C for 5 min. I_2 was added portionwise until the yellow colour of the solution remained (app.rox. 1 g). The solution was diluted with EtOAc, washed with HCl (aq., 1 M), $Na_2S_2O_3$ (aq., 10% w/w), and brine. The organic layer was dried over Na_2SO_4, filtered, and concentrated.

The crude residue (210 mg) was dissolved in CH_2Cl_2 (12 mL) and 1 drop DMF was added. The solution was cooled to 0 °C, and oxalyl chloride (0.30 mL, 3.3 mmol) was added dropwise. The reaction mixture was stirred at 0 °C for 1.5 h and concentrated.

The crude residue (78 mg) was dissolved in CH_2Cl_2 (1.5 mL) and added dropwise to a solution of indoline **84** (50 mg, 0.13 mmol) and pyridine (43 µL, 0.53 mmol) in CH_2Cl_2 (2 mL) and stirred at rt for 30 min. $NaHCO_3$ (saturated aq., 5 mL) was added, and the mixture was extracted with CH_2Cl_2. The combined organic extracts were washed with brine, dried over Na_2SO_4, filtered, and concentrated. Purification by flash pressure column chromatography (25% EtOAc/petroleum ether) afforded indoline **116** as a colourless gum (40 mg, 0.082 mmol, 62%).

^{1}H NMR (500 MHz, C_6D_6, 348 K): δ 0.56 (3H, s), 1.42 (2H, t, *J* = 6.7 Hz), 1.57 (2H, app. m), 5.25 (1H, s), 6.73 (1H, td, *J* = 7.6 Hz, 0.8 Hz), 6.94-7.10 (5H, m), 7.20 (2H, m), 7.56 (1H, t, *J* = 2.1 Hz), 8.06 (1H, br. s), 8.42 (1H, d, *J* = 1.9 Hz), 8.56 (1H, d, *J* =2.1 Hz).

^{13}C NMR (126 MHz, C_6D_6, 348 K): δ 19.7, 24.7, 29.0, 29.7, 57.5, 73.2, 117.2, 118.3, 121.4, 125.8, 125.9, 126.1, 129.1, 129.8, 130.9, 135.4, 136.4, 140.3, 142.7, 146.6, 152.6, 169.0.ǂ

ǂ 1 peak obscured.

HRMS (ES$^+$): $[C_{25}H_{21}ON_5{}^{79}Br]^+$ ($[M+H]^+$) requires 486.0924; found 486.0924. $[C_{25}H_{21}ON_5{}^{81}Br]^+$ ($[M+H]^+$) requires 488.0904; found 488.0902.

1-(5′-Azidopentyl)pyrrolidine **118**

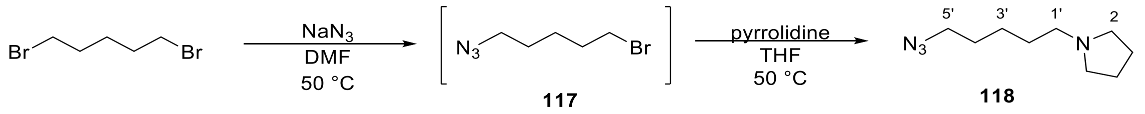

Azide **118** was prepared according to a literature procedure.[13] NaN_3 (286 mg, 4.42 mmol) was added to a solution of 1,5-dibromopentane (1.2 mL, 8.8 mL) in DMF (30 mL) and stirred at 50 °C for 3 h. The mixture was allowed to cool to rt, diluted with EtOAc, and washed with H_2O, then brine. The organic layer was dried over Na_2SO_4, filtered, and concentrated. Purification by flash pressure column chromatography (3.5% Et_2O/hexane) afforded **117** as a colorless liquid (557 mg, 2.90 mmol, 66%). Azide **117** was not fully characterized due to concerns about its toxicity and stability.

A solution of azide **117** (200 mg, 1.04 mmol) in dry THF (2 mL) was added dropwise to a solution of pyrrolidine (0.96 mL, 12 mmol) in dry THF (3 mL) and stirred at 50 °C for 1 h. Water (5 mL) was added, and the mixture was extracted with Et_2O. The combined organic extracts were washed with brine, dried over $MgSO_4$, filtered, and

concentrated to afford azide **118** as a yellow liquid (171 mg, 0.94 mmol, 90%).

^{1}H NMR (400 MHz, $CDCl_3$): δ 1.41 (2H, m), 1.49-1.68 (4H, m), 1.81 (4H, m), 2.37-2.55 (6H, m), 3.27 (2H, t, *J* = 7.0 Hz).

^{13}C NMR (100.6 MHz, $CDCl_3$): δ 23.5, 24.9, 28.5, 28.9, 51.5, 54.3, 56.4.

HRMS (ES$^+$): $[C_9H_{19}N_4]^+$ ($[M+H]^+$) requires 183.1604; found 183.1605.

(2*RS*,3*SR*)-1-Acetyl-3-phenyl-2-(5-(1-(5-(pyrrolidin-1-yl)pentyl)-1H-1,2,3-triazol-4-yl)pyridin-3-yl)indoline-3-carbonitrile **119**

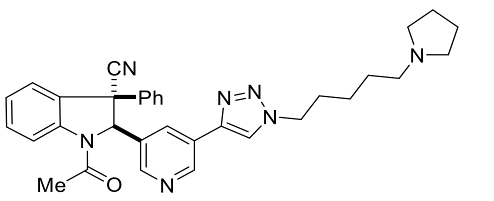

Indoline **119** was prepared according to a modified literature procedure.[14] $CuSO_4$ (aq., 2.5 mM, 0.55 mL, 0.0014 mmol) and sodium-L-ascorbate (aq., 12.5 mM, 0.55 mL, 0.0069 mmol) were added to a solution of alkyne **97** (25 mg, 0.070 mmol) and azide **118** (15 mg, 0.083 mmol) in ^{t}BuOH (1.1 mL). The mixture was stirred at rt for 16 h. H_2O (4 mL) was added, and the mixture was extracted with EtOAc. The combined organic extracts were washed with brine, dried over Na_2SO_4, filtered, and concentrated. Purification by preparatory TLC (6% MeOH/CH_2Cl_2 + 0.5% aq. NH_4OH) afforded indoline **119** as a colorless solid (9.3 mg, 0.017 mmol, 26%).

^{1}H NMR (500 MHz, C_6D_6, 348 K): δ 1.10 (2H, quin, *J* = 7.2 Hz), 1.31 (2H, quin, *J* = 7.2 Hz), 1.47 (2H, quin, *J* = 7.2 Hz), 1.61 (4H, m), 1.70 (3H, s), 2.26 (2H, t, *J* = 7.0 Hz), 2.36 (4H, m), 3.69 (2H, t, *J* = 7.1 Hz), 5.82 (1H, s), 6.72-6.82 (5H, m), 6.84 (1H, dd, *J* = 7.7 Hz, 0.9 Hz), 7.04-7.09 (2H, m), 7.12 (1H, td, *J* = 7.8 Hz, 1.4 Hz), 7.65 (1H, t, *J* = 2.0 Hz), 8.04 (1H, app. s), 8.32 (1H, br. s), 8.75 (1H, d, *J* = 2.0 Hz).

^{13}C NMR (125.75 MHz, C_6D_6, 348 K): δ 24.1, 24.4, 24.9, 28.9, 30.5, 50.4, 54.6, 56.1, 56.3, 73.7, 117.9, 120.0, 121.7, 125.6, 126.7, 127.6, 128.9, 129.1, 129.3, 129.3, 131.5, 131.7, 132.9, 134.2, 144.2, 145.1, 147.6, 148.4, 168.5.

HRMS (ESI$^+$, m/z): $[C_{33}H_{36}N_7O]^+$ ($[M+H]^+$) calc. 546.2976; found 546.2983.

MP: 116-123 °C.

(2*RS*,3*RS*)-1-Acetyl-3-phenyl-2-(5-(1-(5-(pyrrolidin-1-yl)pentyl)-1H-1,2,3-triazol-4-yl)pyridin-3-yl)indoline-3-carbonitrile **2**

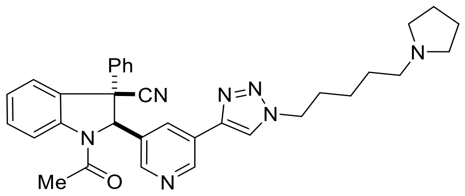

Indoline **2** was prepared according to a modified literature procedure.[14] $CuSO_4$ (aq., 10 mM, 0.33 mL, 0.0033 mmol) and sodium-L-ascorbate (aq., 50 mM, 0.13 mL, 0.0065 mmol) were added to a solution of alkyne **98** (24 mg, 0.070 mmol) and azide **118** (12 mg, 0.066 mmol) in 3:1 ^{t}BuOH/H_2O (1 mL). The mixture was stirred at rt for 16 h. H_2O (4 mL) was added, and the mixture was extracted with EtOAc. The combined organic extracts were washed with brine, dried over Na_2SO_4, filtered, and concentrated. Purification by preparatory TLC (6% MeOH/CH_2Cl_2 + 0.5% aq. NH_4OH) afforded indoline **2** as a colorless solid (9.5 mg, 0.017 mmol, 26%).

^{1}H NMR (500 MHz, C_6D_6, 348 K): δ 1.13 (2H, quin, *J* = 7.3 Hz), 1.33 (2H, quin, *J* = 7.3 Hz), 1.50 (2H, quin, *J* = 7.5 Hz), 1.55 (3H, s), 1.62 (4H, m), 2.27 (2H, t, *J* = 7.3 Hz), 2.37 (4H, m), 3.72 (2H, t, *J* = 7.3 Hz), 5.35 (1H, s), 6.76 (1H, t, *J* = 7.5 Hz), 6.88 (1H, s), 6.95-7.05 (3H, m), 7.08 (1H, d, *J* = 7.5 Hz), 7.11 (1H, t, *J* = 7.6 Hz), 7.25 (2H, m), 8.32 (1H, br. s), 8.36 (1H, t, *J* = 2.0 Hz), 8.61 (1H, d, *J* = 2.0 Hz), 9.06 (1H, d, *J* = 2.0 Hz).

^{13}C NMR (125.75 MHz, C_6D_6, 348 K): δ 23.7, 24.4, 25.0, 28.9. 30.5, 50.5, 54.6, 56.3, 58.3, 75.0, 117.8, 119.2, 120.4, 125.9, 126.3, 126.4, 129.3, 129.8, 130.2, 131.2, 131.3, 134.7, 141.2, 144.0, 144.4, 148.0, 149.1, 168.2.[ƚ]

ƚ 1 peak obscured.

HRMS (ES$^+$): $[C_{33}H_{36}N_7O]^+$ ($[M+H]^+$) calc. 546.2976; found 546.2980.

MP: 109-114 °C.

(*E*)-7-(Tributylstannyl)hept-6-en-1-ol **120**

Vinylstannane **120** was prepared according to a literature procedure.[15] Pd_2dba_3 (16.3 mg, 0.018 mmol), cyc_3PHBF_4 (26 mg, 0.071 mmol), and DIPEA (25 µL, 0.14 mmol) were added to a solution of CH_2Cl_2 (15 mL) and stirred at rt for 10 min. 6-Heptyn-1-ol (400 mg, 3.57 mmol) was added, and the mixture was cooled to 0 °C. A solution of Bu_3SnH (1.15 mL, 4.28 mmol) in CH_2Cl_2 (5 mL) was added dropwise over 5 min and stirred at 0 °C for 2.5 h. The mixture was concentrated and the residue purified by flash pressure column chromatography (10% EtOAc/petroleum ether) to afford vinyl stannane **120** as a colorless liquid (1.20 g, 2.98 mmol, 84%).

^{1}H NMR (400 MHz, CDCl$_3$): δ 0.74-0.98 (15H, m), 1.28-1.63 (19H, m), 2.15 (2H, m), 3.65 (2H, td, *J* = 6.6 Hz, 5.4 Hz), 5.86 (1H, app. d, *J* = 19.0 Hz), 5.93 (1H, app. dt, *J* = 19.0 Hz, 5.6 Hz).

^{13}C NMR (100.6 MHz, CDCl$_3$): δ 9.4, 13.7, 25.2, 27.3, 28.7, 29.1, 32.7, 37.8, 63.0, 127.3, 149.4.

(*E*)-1-(7-(Tributylstannyl)hept-6-en-1-yl)pyrrolidine **122**

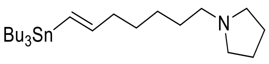

PPh_3 (972 mg, 3.71 mmol) was added portionwise to a solution of vinyl stannane **120** (1.15 g, 2.85 mmol) and CBr_4 (1.23 g, 3.71 mmol) in CH_2Cl_2 (20 mL), and the mixture was stirred at 0 °C for 1 h. Ice-cooled pentane (50 mL) was added. The white precipitate was removed by filtration, and the filtrate was concentrated. The resulting residue was dissolved in THF (10 mL) and added to a solution of pyrrolidine (1.20 mL, 13.9 mmol) in THF (10 mL). The mixture was stirred at 50 °C for 4 h. H_2O (20 mL) was added and the mixture was extracted with EtOAc. The combined organic extracts were washed with brine, dried over Na_2SO_4, filtered, and concentrated. Purification by flash pressure column chromatography (5% MeOH/CH_2Cl_2 + 0.5% aq. NH_4OH) afforded vinyl stannane **122** as a yellow oil (413 mg, 0.90 mmol, 32%).

^{1}H NMR (400 MHz, CDCl$_3$): δ 0.82-0.93 (15H, m), 1.23-1.37 (8H, m), 1.39-1.55 (10H, m), 1.78 (4H, m), 2.14 (2H, app. q, *J* = 6.7 Hz), 2.42 (2H, m), 2.49 (4H, m), 5.86 (1H, app. d, *J* = 18.9 Hz), 5.96 (1H, app. dt, *J* = 18.9 Hz, 5.8 Hz).

^{13}C NMR (100.6 MHz, CDCl$_3$): δ 9.4, 13.7, 23.4, 27.2, 27.3, 28.9, 29.0, 29.1, 37.8, 54.2, 56.7, 127.1, 149.6.

HRMS (ES$^+$): $[C_{23}H_{48}N^{120}Sn]^+$ ($[M+H]^+$) requires 458.2807; found 458.2809.

(2*RS*,3*SR*)-1-Acetyl-3-phenyl-2-(5-((E)-7-(pyrrolidin-1-yl)hept-1-en-1-yl)pyridin-3-yl)indoline-3-carbonitrile **123**

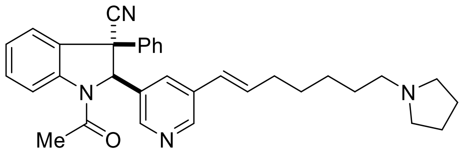

Indoline **123** was prepared according to general procedure 6, using indoline **93** (60 mg, 0.14 mmol), vinyl stannane **122** (78 mg, 0.17 mmol), and $Pd(PPh_3)_4$ (17 mg, 0.014 mmol) in NMP (1.5 mL). The reaction mixture was stirred at 85 °C for 1.5 h. Purification by flash pressure column chromatography (4.5% $MeOH/CH_2Cl_2$ + 0.5% aq. NH_4OH) afforded indoline **123** as a pale yellow oil (38 mg, 0.075 mmol, 54%).

^{1}H NMR (500 MHz, C_6D_6, 348 K): δ 1.23-1.35 (4H, m), 1.49 (2H, quin, *J* = 7.1 Hz), 1.63 (4H, m), 1.67 (3H, s), 1.93 (2H, m), 2.37 (2H, t, *J* = 7.2 Hz), 2.42 (4H, m), 5.71 (1H, s), 5.89 (1H, dt, *J* = 16.1 Hz, 6.2 Hz), 5.86 (1H, d, *J* = 16.1 Hz), 6.68-6.88 (6H, m), 7.00 (2H, m), 7.13 (1H, obscured), 7.91 (1H, app. s), 8.29 (1H, d, *J* = 2.0 Hz), 8.41 (1H, br. s).

^{13}C NMR (125.75 MHz, C_6D_6, 348 K): δ 24.0, 24.3, 27.6, 29.5, 29.5, 33.4, 54.5, 56.0, 56.6, 73.6, 117.8, 121.6, 125.5, 126.5, 126.5, 128.9, 129.2, 131.3, 131.4, 132.1, 133.4, 134.1, 134.5, 145.1, 147.2, 148.4, 168.4.‡

‡ 2 peaks obscured.

HRMS (ESI$^+$, m/z): $[C_{33}H_{37}N_4O]^+$ ($[M+H]^+$) calc. 505.2962; found 505.2962.

(2*RS*,3*RS*)-1-Acetyl-3-phenyl-2-(5-((E)-7-(pyrrolidin-1-yl)hept-1-en-1-yl)pyridin-3-yl)indoline-3-carbonitrile **5**

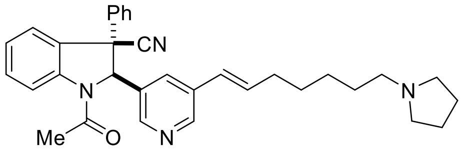

Indoline **5** was prepared according to general procedure 6, using indoline **94** (40 mg, 0.095 mmol), vinyl stannane **122** (48 mg, 0.11 mmol), and $Pd(PPh_3)_4$ (11 mg, 0.0095 mmol) in NMP (1.2 mL). The reaction mixture was stirred at 85 °C for 2 h. Purification by flash pressure column chromatography (5% $MeOH/CH_2Cl_2$ + 0.5% aq. NH_4OH) afforded indoline **5** as a pale yellow oil (19 mg, 0.038 mmol, 40%).

^{1}H NMR (500 MHz, C_6D_6, 348 K): 1.24-1.35 (4H, m), 1.42-1.56 (5H, m), 1.64 (4H, m), 1.93 (2H, app. q, *J* = 6.9 Hz), 2.38 (2H, t, *J* = 7.1 Hz), 2.43 (4H, m), 5.24 (1H, s), 5.94-6.10 (2H, m),† 6.77 (1H, t, *J* = 7.6 Hz), 6.95-7.04 (3H, m),

7.07 (1H, d, *J* = 7.6 Hz), 7.12 (1H, obscured), 7.21-7.30 (2H, m), 7.42 (1H, t, *J* = 2.0 Hz), 8.39 (1H, br. s), 8.45 (1H, d, *J* = 2.0 Hz), 8.66 (1H, d, *J* = 2.0 Hz).

ŧ ^{1}**H NMR (500 MHz, C_6D_6):** δ 5.96 (1H, dt, *J* = 16.1 Hz, 6.7 Hz), 5.86 (1H, d, *J* = 16.1 Hz).

^{13}**C NMR (125.75 MHz, C_6D_6, 348 K):** δ 23.7, 24.5, 27.9, 29.6, 29.6, 33.7, 54.7, 56.8, 58.3, 74.9, 117.7, 119.1, 126.0, 126.3, 126.5, 126.8, 129.4, 130.2, 130.6, 131.3, 134.1, 134.8, 135.1, 141.4, 144.1, 147.3, 149.9, 168.3.‡

‡ 1 peak obscured.

HRMS (ESI$^+$, m/z): $[C_{33}H_{37}N_4O]^+$ ($[M+H]^+$) calc. 505.2962; found 505.2967.

Ethyl (2*RS*,3*RS*)-1-acetyl-3-cyano-2-(5-((E)-7-(pyrrolidin-1-yl)hept-1-en-1-yl)pyridin-3-yl)indoline-3-carboxylate **124**

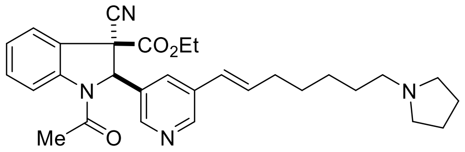

Indoline **124** was prepared according to general procedure 6, using indoline **99** (70 mg, 0.17 mmol), vinyl stannane **122** (108 mg, 0.24 mmol), and $Pd(PPh_3)_4$ (20 mg, 0.017 mmol) in NMP (2.0 mL). The reaction mixture was stirred at 85 °C for 1 h. Purification by flash pressure column chromatography (5% $MeOH/CH_2Cl_2$ + 0.5% aq. NH_4OH) afforded indoline **124** as a pale yellow oil (30 mg, 0.060 mmol, 36%, contains minor impurities).

^{1}**H NMR (500 MHz, C_6D_6, 348 K):** δ 0.65 (3H, t, *J* = 7.0 Hz), 1.20-1.32 (4H, m), 1.46 (2H, quin, *J* = 7.1 Hz), 1.59 (3H, s), 1.64 (4H, m), 1.90 (2H, app. q, *J* = 6.4 Hz), 2.37 (2H, t, *J* = 7.1 Hz), 2.41 (4H, m), 3.51 (2H, q, *J* = 7.0 Hz), 5.81 (1H, br. s), 5.91 (1H, d, *J* = 16.1 Hz), 5.97 (1H, dt, *J* = 16.1 Hz, 6.4 Hz), 6.82 (1H, td, *J* = 7.7 Hz, 0.9 Hz), 7.08 (1H, td, *J* = 7.7 Hz, 1.3 Hz), 7.22 (1H, app. d, *J* = 7.5 Hz), 7.26 (1H, m), 8.12 (1H, br. s), 8.31 (1H, d, *J* = 2.2 Hz), 8.51 (1H, d, *J* = 2.2 Hz).

^{13}**C NMR (125.75 MHz, C_6D_6, 348 K):** δ 13.7, 23.9, 24.5, 27.8, 29.5, 29.6, 33.6, 54.7, 56.4, 56.8, 63.8, 70.6, 117.4, 118.3, 125.4, 125.4, 126.4, 127.4, 131.4, 131.7, 132.0, 134.2, 135.3, 144.5, 147.8, 149.5, 164.4 , 168.0.

HRMS (ESI$^+$, m/z): $[C_{30}H_{37}N_4O_3]^+$ ($[M+H]^+$) calc. 501.2860; found 501.2847.

Methyl (2*RS*,3*SR*)-1-acetyl-3-phenyl-2-(5-((E)-7-(pyrrolidin-1-yl)hept-1-en-1-yl)pyridin-3-yl)indoline-3-carboxylate **125**

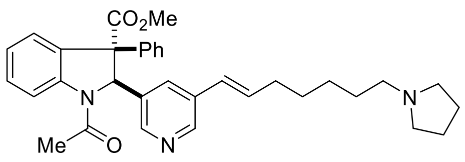

Indoline **125** was prepared according to general procedure 6, using indoline **100** (50 mg, 0.11 mmol), vinyl stannane **122** (71 mg, 0.16 mmol), and $Pd(PPh_3)_4$ (13 mg, 0.011 mmol) in NMP (1.5 mL). The reaction mixture was stirred at 85 °C for 1.2 h. Purification by flash pressure column chromatography (6.5% $MeOH/CH_2Cl_2$ + 0.5% aq. NH_4OH) afforded indoline **125** as a pale yellow oil (40 mg, 0.074 mmol, 67%).

^{1}H NMR (500 MHz, C_6D_6, 348 K): δ 1.23-1.39 (4H, m), 1.49 (2H, quin, *J* = 6.8 Hz), 1.64 (4H, m), 1.88-1.99 (5H, m), 2.39 (2H, t, *J* = 7.2 Hz), 2.42 (4H, m), 3.23 (3H, s), 5.84 (1H, dt, *J* = 16.0 Hz, 6.1 Hz), 5.90 (1H, d, *J* = 16.0 Hz), 6.45 (1H, s), 6.77-6.95 (7H, m), 7.21 (1H, td, *J* = 7.7 Hz, 1.3 Hz), 7.36 (1H, d, *J* = 7.7 Hz), 8.07 (1H, app. s), 8.29 (1H, s), 8.64 (1H, br. s).

^{13}C NMR (125.75 MHz, C_6D_6, 348 K): δ 24.1, 24.5, 27.8, 29.7, 29.7, 33.6, 53.1, 54.7, 56.8, 68.4, 70.6, 118.0, 124.6, 127.0, 128.8, 129.0, 130.2, 130.7, 131.4, 133.4, 134.1, 134.3, 137.7, 145.5, 147.9, 147.9, 168.6 , 173.2.‡

‡ Remaining 2 peaks obscured.

HRMS (ES$^+$): $[C_{34}H_{40}N_3O_3]^+$ ($[M+H]^+$) calc. 538.3064; found 538.3058.

Methyl (2*RS*,3*RS*)-1-acetyl-3-phenyl-2-(5-((E)-7-(pyrrolidin-1-yl)hept-1-en-1-yl)pyridin-3-yl)indoline-3-carboxylate **10**

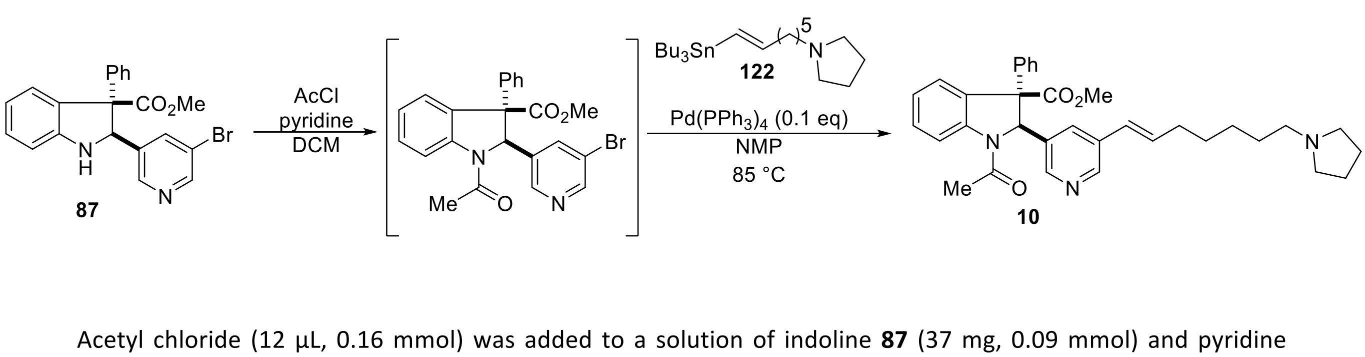

Acetyl chloride (12 µL, 0.16 mmol) was added to a solution of indoline **87** (37 mg, 0.09 mmol) and pyridine (13 µL, 0.16 mmol) in CH_2Cl_2 (5 mL). The reaction mixture was stirred at rt for 20 min. $NaHCO_3$ (saturated aq.)

was added, and the mixture was extracted with CH_2Cl_2. The combined organic extracts were washed with brine, dried over Na_2SO_4, filtered, and concentrated. The remaining residue (45 mg) was dissolved together with vinyl stannane **122** (57 mg, 0.12 mmol) in NMP (2.0 mL) and degassed with Ar. $Pd(PPh_3)_4$ (10 mg, 0.0087 mmol) was added, and the mixture was stirred at 85 °C for 4.5 h. The mixture was diluted with CH_2Cl_2 and washed with H_2O, then brine. The organic layer was dried over Na_2SO_4, filtered, and concentrated. Purification by flash pressure column chromatography (5% MeOH/CH_2Cl_2 + 0.5% aq. NH_4OH) afforded indoline **10** as a pale yellow oil (20 mg, 0.037 mmol, 41%).

^{1}H NMR (500 MHz, $(CD_3)_2SO$, 363 K): δ 1.33-1.39 (2H, quin, *J* = 6.9 Hz), 1.40-1.51 (4H, m), 1.67 (4H, m), 2.15 (3H, s), 2.19 (2H, app. q, *J* = 7.2 Hz), 2.38 (2H, t, *J* = 7.3 Hz), 2.42 (4H, m), 3.23 (3H, s), 6.18 (1H, s), 6.26 (1H, dt, *J* = 16.0 Hz, 6.7 Hz), 6.35 (1H, d, *J* = 16.0 Hz), 7.19 (1H, t, *J* = 7.6 Hz), 7.30 (1H, t, *J* = 7.3 Hz), 7.37 (3H, m), 7.44 (2H, app. d, *J* = 7.6 Hz), 7.53 (1H, s), 7.63 (1H, d, *J* = 7.6 Hz), 7.99 (1H, br. s), 8.19 (1H, d, *J* = 1.9 Hz), 8.49 (1H, d, *J* = 1.9 Hz).

^{13}C NMR (125.75 MHz, $(CD_3)_2SO$, 363 K): δ 22.8, 23.2, 26.1, 27.7, 27.9, 31.7, 51.2, 53.0, 55.1, 69.1, 115.5, 123.5, 125.3, 127.1, 127.3, 128.3, 128.4, 130.6, 132.1, 133.3, 133.5, 133.5, 141.3, 141.6, 146.0, 146.9, 167.4, 169.3.‡

‡ 1 peak obscured.

HRMS (ES$^+$): $[C_{34}H_{40}N_3O_3]^+$ ($[M+H]^+$) calc. 538.3064; found 538.3066.

(2*RS*,3*SR*)-1-Acetyl-3-phenyl-2-(3-((E)-7-(pyrrolidin-1-yl)hept-1-en-1-yl)phenyl)indoline-3-carbonitrile **126**

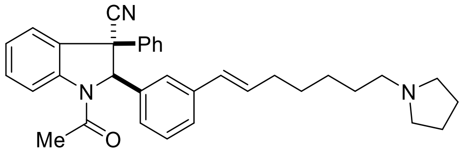

Indoline **126** was prepared according to general procedure 6, using indoline **103** (38 mg, 0.091 mmol), vinyl stannane **122** (66 mg, 0.15 mmol), and $Pd(PPh_3)_4$ (11 mg, 0.0095 mmol) in NMP (1.6 mL). The reaction mixture was stirred at 90 °C for 2.5 h. Purification by flash pressure column chromatography (5% MeOH/CH_2Cl_2 + 0.5% aq. NH_4OH) afforded indoline **126** as a pale yellow oil (27 mg, 0.054 mmol, 59%).

^{1}H NMR (500 MHz, C_6D_6, 348 K): δ 1.35 (4H, m), 1.50 (2H, quin, *J* = 6.8 Hz), 1.59-1.67 (4H, m), 1.69 (3H, s), 2.00 (2H, app. q, *J* = 6.8 Hz), 2.35-2.49 (6H, m), 5.64 (1H, s), 5.84 (1H, dt, *J* = 15.8 Hz, 6.8 Hz), 6.00 (1H, d, *J* = 15.8 Hz),

6.43 (1H, d, J = 7.5 Hz), 6.55 (1H, br. s), 6.64 (1H, t, J = 7.5 Hz), 6.74-6.88 (6H, m), 7.03 (2H, m), 7.12-7.20 (1H, obscured), 8.69 (1H, d, J = 8.2 Hz).

^{13}C NMR (125.75 MHz, C_6D_6): δ 24.3, 24.3, 27.8, 29.7, 29.9, 33.6, 54.7, 56.1, 56.8, 75.4, 117.9, 122.1, 125.5, 126.2, 126.5, 127.9, 128.9, 129.1, 129.2, 129.8, 131.4, 132.3, 134.3, 136.8, 138.8, 145.4, 169.2.[‡]

‡ 3 peaks obscured

HRMS (ESI$^+$, m/z): $[C_{34}H_{38}N_3O]^+$ ($[M+H]^+$) calc. 504.3009; found 504.2998.

(2*RS*,3*RS*)-1-Acetyl-3-phenyl-2-(3-((E)-7-(pyrrolidin-1-yl)hept-1-en-1-yl)phenyl)indoline-3-carbonitrile **9**

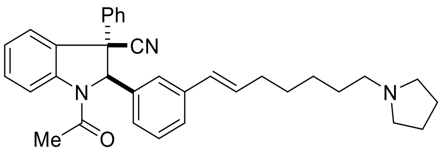

Indoline **9** was prepared according to general procedure 6, using indoline **104** (50 mg, 0.12 mmol), vinyl stannane **122** (77 mg, 0.17 mmol), and $Pd(PPh_3)_4$ (14 mg, 0.012 mmol) in NMP (2.0 mL). The reaction mixture was stirred at 85 °C for 1.5 h. Purification by flash pressure column chromatography (4.5% $MeOH/CH_2Cl_2$ + 0.5% aq. NH_4OH) afforded indoline **9** as a pale yellow oil (35 mg, 0.070 mmol, 58%).

^{1}H NMR (500 MHz, C_6D_6, 348 K): δ 1.37 (4H, m), 1.51 (2H, quin, J = 6.9 Hz), 1.57 (3H, s), 1.60-1.68 (4H, m), 2.05 (2H, app. q, J = 6.7 Hz), 2.35-2.47 (6H, m), 5.22 (1H, s), 6.11 (1H, dt, J = 15.6 Hz, 6.8 Hz), 6.25 (1H, d, J = 15.6 Hz), 6.80 (1H, t, J = 7.6 Hz), 6.95 (1H, d, J = 7.3 Hz), 6.98-7.07 (4H, m), 7.11 (1H, d, J = 7.6 Hz), 7.17-7.22 (2H, m), 7.24 (1H, s), 7.33 (2H, m), 8.62 (1H, d, J = 7.8 Hz).

^{13}C NMR (125.75 MHz, C_6D_6, 348 K): δ 24.0, 24.3, 27.9, 29.7, 29.8, 33.7, 54.7, 56.8, 58.5, 77.1, 117.7, 119.1, 125.0, 125.2, 125.8, 126.3, 126.4, 127.3, 128.9, 129.2, 130.1, 130.1, 130.2, 131.2, 132.7, 138.9, 139.9, 141.9, 144.4, 168.9.

HRMS (ESI$^+$, m/z): $[C_{34}H_{38}N_3O]^+$ ($[M+H]^+$) calc. 504.3009; found 504.2993.

(2*RS*,3*RS*)-1-(3-(3-Methyl-3H-diazirin-3-yl)propanoyl)-3-phenyl-2-(5-((E)-7-(pyrrolidin-1-yl)hept-1-en-1-yl)pyridin-3-yl)indoline-3-carbonitrile **127**

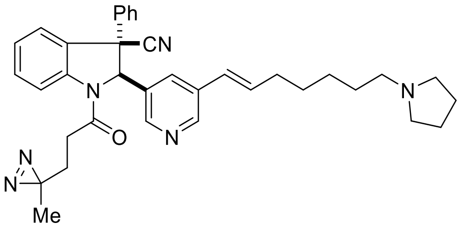

Indoline **127** was prepared according to general procedure 6 using indoline **116** (35 mg, 0.072 mmol), vinyl stannane **122** (39 mg, 0.086 mmol), and $Pd(PPh_3)_4$ (8 mg, 0.007 mmol) in NMP (1.5 mL). The reaction mixture was stirred at 60 °C for 3.5 h. Purification by flash pressure column chromatography (6.5% $MeOH/CH_2Cl_2$ + 0.5% aq. NH_4OH) afforded indoline **127** as a pale yellow paste (14 mg, 0.024 mmol, 34%).

^{1}H NMR (500 MHz, C_6D_6, 348 K): δ 0.53 (3H, s), 1.22-1.34 (4H, m), 1.34-1.51 (4H, m), 1.56-1.73 (6H, m), 1.93 (2H, app. q, *J* = 6.4 Hz), 2.36 (2H, t, *J* = 7.3 Hz), 2.41 (4H, m), 5.35 (1H, s), 5.98-6.10 (2H, m),[ƚ] 6.96-7.06 (3H, m), 7.08 (1H, d, *J* = 7.8 Hz), 7.12 (1H, t, *J* = 7.9 Hz), 7.28 (2H, app. d, *J* = 7.5 Hz), 7.45 (1H, app. s), 8.36 (1H, br. s), 8.48 (1H, app. s), 8.65 (1H, app. s).

ƚ **^{1}H NMR (500 MHz, C_6D_6):** δ 6.00 (1H, dt, *J* = 15.9 Hz, 6.7 Hz), 5.94 (1H, d, *J* = 15.9 Hz).

^{13}C NMR (126 MHz, C_6D_6, 348 K): δ 20.0, 24.5, 25.1, 27.8, 29.5, 29.6, 29.6, 30.3, 33.6, 54.6, 56.7, 58.3, 74.5, 117.8, 118.9, 126.1, 126.3, 126.5, 126.8, 129.4, 130.2, 130.8, 131.2, 133.9, 134.8, 135.2, 141.2, 143.8, 147.2, 150.0, 169.8.[‡]

‡ 1 peak obscured.

HRMS (ES$^+$): $[C_{36}H_{41}ON_6]^+$ ($[M+H]^+$) requires 573.3336; found 573.3339.

7-bromohept-1-yne **128**

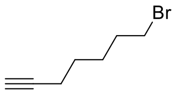

Alkyne **128** was prepared according to a literature procedure.[16] PPh_3 (1.22 mg, 4.64 mmol) was added portionwise to a solution of 6-heptyn-1-ol (400 g, 3.57 mmol) and CBr_4 (1.54 g, 4.64 mmol) in CH_2Cl_2 (8 mL), and

the mixture was stirred at 0 °C for 1.5 h. Ice-cooled hexane (30 mL) was added. The white precipitate was removed by filtration, and the filtrate was concentrated. Purification by flash pressure column chromatography (100% petroleum ether) afforded alkyne **128** as a colorless liquid (537 mg, 3.07 mmol, 86%).

^{1}H (400 MHz, CDCl$_3$): δ 3.42 (2H, t, *J* = 6.8 Hz), 2.22 (2H, app. m), 1.96 (1H, t, *J* = 2.7 Hz), 1.89 (2H, app. m), 1.48-1.64 (4H, m).

^{13}C (101 MHz, CDCl$_3$): δ 84.1, 68.5, 33.5, 32.3, 27.6, 27.3, 18.3.

7-Pyrrolidine-hept-1-yne **129**

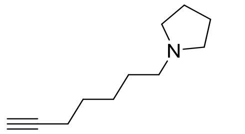

Alkyne **128** (280 mg, 1.60 mmol) in THF (4 mL) was added to a solution of pyrrolidine (1.3 mL, 16 mmol) in THF (4 mL). The mixture was stirred at 50 °C for 1.5 h. H_2O was added, and the mixture was extracted with Et_2O. The combined organic extracts were washed with brine, dried over Na_2SO_4, filtered, and concentrated. Purification by flash pressure column chromatography (5% MeOH/CH_2Cl_2 + 0.5% aq. NH_4OH) afforded alkyne **129** as a yellow liquid (245 mg, 1.48 mmol, 41%).

^{1}H NMR (400 MHz, CDCl$_3$): δ 1.43 (2H, quin, *J* = 7.1 Hz), 1.47-1.61 (4H, m), 1.76 (4H, m), 1.93 (1H, t, *J* = 2.7 Hz), 2.18 (2H, td, *J* = 7.0 Hz, 2.7 Hz), 2.37-2.53 (6H, m).

^{13}C NMR (100.6 MHz, CDCl$_3$): δ 18.3, 23.4, 26.8, 28.4, 28.6, 54.2, 56.5, 68.1, 84.6.

HRMS (ES$^+$): $[C_{11}H_{20}N]^+$ ($[M+H]^+$) requires 166.1590; found 166.1592.

(2*RS*,3*SR*)-1-Acetyl-3-phenyl-2-(5-(7-(pyrrolidin-1-yl)hept-1-yn-1-yl)pyridin-3-yl)indoline-3-carbonitrile **130**

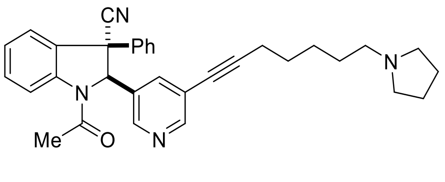

Indoline **130** was prepared according to general procedure 7, using indoline **93** (70 mg, 0.17 mmol), alkyne **129** (41 mg, 0.25 mmol), $PdCl_2(PPh_3)_2$ (5.9 mg, 0.0084 mmol), and CuI (1.6 mg, 0.0084 mmol) in *N*,*N*-

diisopropylamine (2.5 mL). The reaction mixture was stirred at 70 °C for 1 h. Purification by flash pressure column chromatography (5% MeOH/CH_2Cl_2 + 0.5% aq. NH_4OH) afforded indoline **130** as a pale yellow oil (55 mg, 0.11 mmol, 66%).

^{1}H NMR (500 MHz, C_6D_6, 348 K): δ 1.32-1.44 (6H, m), 1.57-1.67 (7H, m), 2.09 (2H, t, *J* = 6.6 Hz), 2.33 (2H, t, *J* = 6.9 Hz), 2.39 (4H, m), 5.63 (1H, s), 6.73 (1H, t, *J* = 7.7 Hz), 6.76-6.86 (4H, m), 6.89-6.99 (3H, m), 7.08 (1H, td, *J* = 7.7 Hz, 1.3 Hz), 7.84 (1H, br. s), 8.26 (1H, br. s), 8.45 (1H, d, *J* = 1.9 Hz).

^{13}C NMR (125.75 MHz, C_6D_6): δ 19.9, 24.1, 24.3, 27.4, 28.9, 29.1, 54.6, 55.7, 56.6, 72.8, 77.7, 95.4, 117.8, 121.4, 121.6, 125.7, 126.6, 128.9, 129.1, 129.4, 131.5, 132.0, 133.5, 136.8, 137.9, 144.7, 147.5, 152.7, 168.4.

HRMS (ESI$^+$, m/z): $[C_{33}H_{35}N_4O]^+$ ($[M+H]^+$) calc. 503.2805; found 503.2792.

(2*RS*,3*RS*)-1-Acetyl-3-phenyl-2-(5-(7-(pyrrolidin-1-yl)hept-1-yn-1-yl)pyridin-3-yl)indoline-3-carbonitrile **4**

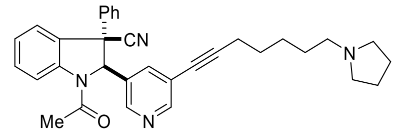

Indoline **4** was prepared according to general procedure 7, using indoline **94** (70 mg, 0.17 mmol), alkyne **129** (41 mg, 0.25 mmol), $PdCl_2(PPh_3)_2$ (5.9 mg, 0.0084 mmol), and CuI (1.6 mg, 0.0084 mmol) in *N*,*N*-diisopropylamine (2.5 mL). The reaction mixture was stirred at 70 °C for 1.25 h. Purification by flash pressure column chromatography (5% MeOH/CH_2Cl_2 + 0.5% aq. NH_4OH) afforded indoline **4** as a pale yellow oil (66 mg, 0.092 mmol, 79%). The non-racemic forms of indoline **4** were prepared from indolines **(*R*,*R*)-110** and **(*S*,*S*)-110**, following the same procedure.

^{1}H NMR (500 MHz, C_6D_6, 348 K): δ 1.34-1.45 (6H, m), 1.47 (3H, s), 1.62 (4H, m), 2.13 (2H, t, *J* = 6.9 Hz), 2.33 (2H, t, *J* = 6.9 Hz), 2.40 (4H, m), 5.18 (1H, br. s), 6.73 (1H, t, *J* = 7.7 Hz), 6.95-7.04 (4H, m), 7.06 (1H, app. t, *J* = 7.7 Hz), 7.19 (2H, m), 7.60 (1H, s), 8.21 (1H, br. s), 8.46 (1H, d, *J* = 2.2 Hz), 8.81 (1H, d, *J* = 1.9 Hz).

^{13}C NMR (125.75 MHz, C_6D_6): δ 20.0, 23.7, 24.3, 27.4, 28.9, 29.1, 54.6, 56.5, 58.0, 74.2, 77.9, 96.0, 117.7, 118.3, 119.0, 126.1, 126.3, 126.4, 129.4, 130.2, 130.6, 131.3, 133.8, 136.1, 140.9, 143.6, 147.2, 154.4, 168.1.

HRMS (ESI$^+$, m/z): $[C_{33}H_{35}N_4O]^+$ ($[M+H]^+$) calc. 503.2805; found 503.2790.

$[\alpha]_D^{25.0}$ (*S*,*S*) = -55 (*c* = 0.1, $CHCl_3$).

(2*SR*,3*RS*)-1-acetyl-3-phenyl-2-(5-(7-(pyrrolidin-1-yl)hept-1-yn-1-yl)furan-2-yl)indoline-3-carbonitrile **132**

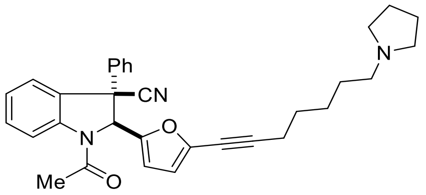

Indoline **132** was prepared according to general procedure 7, using indoline **106** (50 mg, 0.12 mmol), alkyne **129** (26 mg, 0.16 mmol), $PdCl_2(PPh_3)_2$ (4 mg, 0.006 mmol), and CuI (1 mg, 0.006 mmol) in *N*,*N*-diisopropylamine (5 mL). The reaction mixture was stirred at 70 °C for 2.5 h. Purification by flash pressure column chromatography (4.5 % MeOH/CH_2Cl_2 + 0.5% aq. NH_4OH) afforded indoline **132** as a pale yellow oil (51 mg, 0.10 mmol, 83%).

^{1}H NMR (500 MHz, C_6D_6, 348 K): δ 1.33-1.42 (6H, m), 1.55 (3H, s), 1.62 (4H, m), 2.13 (2H, t, *J* = 6.8 Hz), 2.31 (2H, t, *J* = 6.9 Hz), 2.38 (4H, m), 5.31 (1H, br. s), 5.90 (1H, d, *J* = 3.4 Hz), 6.22 (1H, d, *J* = 3.4 Hz), 6.77 (1H, td, *J* = 7.6 Hz, 0.9 Hz), 6.92-7.01 (3H, m), 7.08-7.14 (2H, m), 7.20-7.27 (2H, m), 8.39 (1H, br. s).

^{13}C NMR (125.75 MHz, C_6D_6): δ 20.0, 23.3, 24.3, 27.4, 28.8, 29.2, 54.6, 56.6, 57.0, 71.0, 71.8, 96.8, 110.2, 115.6, 118.1, 118.8, 125.7, 126.2, 126.4, 129.3, 130.1, 131.0, 139.7, 140.5, 143.4, 150.9, 168.3.‡

‡ 1 peak obscured.

HRMS (ESI$^+$, m/z): $[C_{32}H_{34}N_3O_2]^+$ ($[M+H]^+$) calc. 492.2645; found 492.2639.

(2*SR*,3*SR*)-1-acetyl-3-phenyl-2-(5-(7-(pyrrolidin-1-yl)hept-1-yn-1-yl)furan-2-yl)indoline-3-carbonitrile **133**

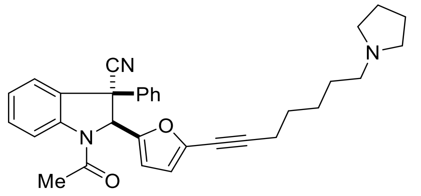

Indoline **133** was prepared according to general procedure 7, using indoline **105** (50 mg, 0.12 mmol), alkyne **129** (26 mg, 0.16 mmol), $PdCl_2(PPh_3)_2$ (4 mg, 0.006 mmol), and CuI (1 mg, 0.006 mmol) in *N*,*N*-diisopropylamine (5 mL). The reaction mixture was stirred at 70 °C for 1.5 h. Purification by flash pressure column

chromatography (4% $MeOH/CH_2Cl_2$ + 0.5% aq. NH_4OH) afforded indoline **133** as a pale yellow oil (50 mg, 0.10 mmol, 82%).

^{1}H NMR (500 MHz, C_6D_6): δ 1.21-1.30 (4H, m), 1.34 (2H, quin, *J* = 6.7 Hz), 1.54 (3H, br. s), 1.62 (4H, m), 1.98 (2H, t, *J* = 6.5 Hz), 2.27 (2H, t, *J* = 7.1 Hz), 2.35 (4H, m), 5.39 (1H, d, *J* = 3.4 Hz), 5.55 (1H, br. s), 5.84 (1H, d, *J* = 3.4 Hz), 6.69 (1H, td, *J* = 7.6 Hz, 1.0 Hz), 6.74 (1H, dd, *J* = 7.6 Hz, 1.2 Hz), 6.94-7.03 (3H, m), 7.07 (1H, t, *J* = 7.5 Hz), 7.23 (2H, m), 8.75 (1H, br. s).

^{13}C NMR (125.75 MHz, C_6D_6): δ 19.8, 23.6, 24.2, 27.3, 28.7, 29.0, 54.6, 55.3, 56.5, 69.9, 71.3, 96.0, 111.1, 114.7, 118.2, 121.2, 125.3, 126.3, 128.7, 128.9, 129.3, 131.2, 133.9, 139.0, 144.6, 149.2, 168.4.‡

‡ 1 peak obscured.

HRMS (ESI$^+$, m/z): $[C_{32}H_{34}N_3O_2]^+$ ($[M+H]^+$) calc. 492.2645; found 492.2634.

(2*SR*,3*RS*)-1-acetyl-3-phenyl-2-(4-(7-(pyrrolidin-1-yl)hept-1-yn-1-yl)furan-2-yl)indoline-3-carbonitrile **134**

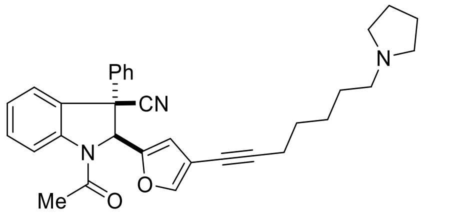

Indoline **134** was prepared according to general procedure 7, using indoline **107** (50 mg, 0.12 mmol), alkyne **129** (28 mg, 0.17 mmol), $PdCl_2(PPh_3)_2$ (4 mg, 0.006 mmol), and CuI (1 mg, 0.006 mmol) in *N*,*N*-diisopropylamine (5 mL). The reaction mixture was stirred at 70 °C for 16 h. Purification by flash pressure column chromatography (4.5% $MeOH/CH_2Cl_2$ + 0.5% aq. NH_4OH) afforded indoline **134** as a pale yellow oil (15 mg, 0.031 mmol, 24%).

^{1}H NMR (500 MHz, C_6D_6, 348 K): δ 1.33-1.49 (9H, m), 1.61 (4H, m), 2.19 (2H, t, *J* = 6.7 Hz), 2.31 (2H, t, *J* = 7.0 Hz), 2.37 (4H, m), 5.08 (1H, br. s), 6.21 (1H, br. s), 6.72 (1H, td, *J* = 7.5 Hz, 1.1 Hz), 6.90-6.97 (3H, m), 7.00-7.13 (2H, m), 7.20 (1H, s), 7.24 (2H, m), 8.68 (1H, br. s).

^{13}C NMR (125.75 MHz, C_6D_6): δ 20.0, 23.3, 24.2, 27.4, 29.1, 29.2, 54.6, 56.6, 56.7, 70.6, 93.4, 110.4, 112.6, 118.2, 118.8, 125.7, 125.9, 126.3, 129.4, 130.1, 131.0, 140.4, 143.4, 146.6, 150.8, 168.2.‡

‡ 2 peaks obscured.

HRMS (ES$^+$): $[C_{32}H_{34}O_2N_3]^+$ ($[M+H]^+$) requires 492.26455; found 492.26437.

(2*RS*,3*RS*)-1-(2-cyanoacetyl)-3-phenyl-2-(5-(7-(pyrrolidin-1-yl)hept-1-yn-1-yl)pyridin-3-yl)indoline-3-carbonitrile **135**

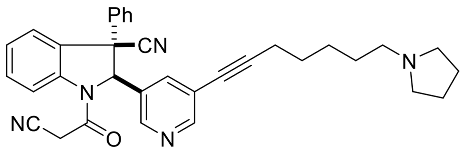

Indoline **135** was prepared according to general procedure 7 using indoline **113** (40 mg, 0.090 mmol), alkyne **129** (16 mg, 0.099 mmol), $PdCl_2(PPh_3)_2$ (3.2 mg, 0.0045 mmol), and CuI (< 1 mg) in diisopropylamine (2 mL). The reaction mixture was stirred at 70 °C for 4.5 h. Purification by flash pressure column chromatography (4.5% MeOH/CH_2Cl_2 + 0.5% aq. NH_4OH) afforded indoline **135** as a colorless oil (19 mg, 0.036 mmol, 40%, contains minor impurities).

^{1}H NMR (400 MHz, C_6D_6, 348 K): δ 1.34-1.50 (6H, m), 1.64 (4H, m), 2.14 (2H, t, *J* = 6.6 Hz), 2.28-2.37 (4H, m), 2.40 (4H, m), 5.06 (1H, br. s), 6.72 (1H, t, *J* = 7.5 Hz), 6.93-7.08 (5H, m), 7.12 (2H, m), 7.47 (1H, app. s), 8.01 (1H, br. s), 8.34 (1H, d, *J* = 1.8 Hz), 8.80 (1H, d, *J* = 1.5 Hz).

^{13}C NMR (100.6 MHz, C_6D_6, 348 K): δ 20.1, 24.5, 26.7, 27.6, 29.0, 29.2, 54.7, 56.6, 58.4, 74.1, 77.8, 96.6, 112.7, 117.7, 118.5, 122.9, 126.2, 126.6, 127.2, 129.7, 130.4, 131.5, 132.5, 136.0, 140.3, 142.6, 146.8, 154.9, 160.2.ǂ

ǂ 1 peak obscured.

HRMS (ES$^+$): $[C_{34}H_{34}N_5O]^+$ ($[M+H]^+$) requires 528.2763; found 528.2762.

(2*RS*,3*RS*)-1-(2-(1,3-dioxoisoindolin-2-yl)acetyl)-3-phenyl-2-(5-(7-(pyrrolidin-1-yl)hept-1-yn-1-yl)pyridin-3-yl)indoline-3-carbonitrile **136**

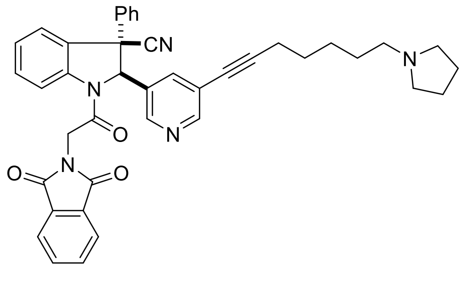

Indoline **136** was prepared according to general procedure 7 using indoline **115** (62 mg, 0.11 mmol), alkyne **129** (23 mg, 0.14 mmol), $Pd(Ph_3)_2Cl_2$ (4 mg, 0.006 mmol), and CuI (app.rox. 1 mg) in *N*,*N*-diisopropylamine (2 mL). The reaction mixture was stirred at 70 °C for 3 h. Purification by flash pressure column chromatography (4.5% $MeOH/CH_2Cl_2$ + 0.5% aq. NH_4OH) afforded indoline **136** as a pale yellow oil (34 mg, 0.053 mmol, 48%).

^{1}H NMR (400 MHz, C_6D_6, 348 K): δ 1.32-1.51 (6H, m), 1.62 (4H, m), 2.13 (2H, t, *J* = 6.7 Hz), 2.33 (2H, t, *J* = 7.0 Hz), 2.40 (4H, m), 4.00 (1H, d, *J* = 16.4 Hz), 4.19 (1H, d, *J* = 16.4 Hz), 5.50 (1H, s), 6.70 (1H, t, *J* = 7.6 Hz), 6.87-6.95 (2H, m), 6.95-7.03 (3H, m), 7.03-7.12 (2H, m), 7.29 (2H, app. d, *J* = 7.4 Hz), 7.37-7.50 (2H, m), 7.68 (1H, s), 7.99 (1H, br. s), 8.52 (1H, s), 8.81 (1H, s).

^{13}C NMR (100.6 MHz, C_6D_6, 348 K): δ 20.1, 24.4, 27.5, 29.0, 29.0, 41.1, 54.6, 56.6, 58.6, 73.7, 78.2, 95.9, 117.9, 118.7, 122.7, 123.7, 126.1, 126.3, 126.6, 129.5, 130.3, 131.2, 133.1, 134.0, 136.4, 140.7, 142.9, 147.2, 154.6, 164.9, 167.5.[†]

ⱡ 2 peaks obscured.

HRMS (ES$^+$): $[C_{41}H_{38}N_5O_3]^+$ ($[M+H]^+$) requires 648.2969; found 648.2963.

(2*RS*,3*RS*)-3-phenyl-2-(5-(7-(pyrrolidin-1-yl)hept-1-yn-1-yl)pyridin-3-yl)indoline-3-carbonitrile **137**

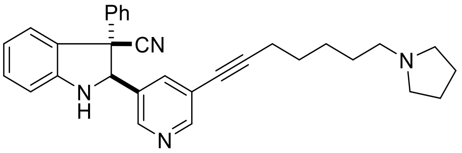

Indoline **137** was prepared according to general procedure 7, using indoline **84** (215 mg, 0.57 mmol), alkyne **129** (104 mg, 0.63 mmol), $Pd(PPh_3)_2Cl_2$ (20 mg, 0.03 mmol), and CuI (5 mg, 0.03 mmol) in *N*,*N*-diisopropylamine (5 mL). The reaction mixture was stirred at 60 °C for 3 h. Purification by flash pressure column chromatography (4.5% $MeOH/CH_2Cl_2$ + 0.5% aq. NH_4OH) afforded indoline **137** as a pale yellow oil (80 mg, 0.17 mmol, 31%).

^{1}H NMR (500 MHz, C_6D_6): δ 1.33-1.50 (6H, m), 1.63 (4H, m), 2.15 (2H, t, *J* = 6.6 Hz), 2.33 (2H, t, *J* = 6.9 Hz), 2.38 (4H, m), 2.99 (1H, d, *J* = 3.2 Hz), 4.29 (1H, d, *J* = 3.2 Hz), 6.42 (1H, d, *J* = 7.6 Hz), 6.59 (1H, td, *J* = 7.6 Hz, 0.9 Hz), 6.73 (1H, d, *J* = 7.6 Hz), 6.93-7.06 (4H, m), 7.22 (2H, m), 8.00 (1H, t, *J* = 2.0 Hz), 8.18 (1H, d, *J* = 2.0 Hz), 8.96 (1H, d, *J* = 2.0 Hz).

^{13}C NMR (125.75 MHz, C_6D_6): δ 20.0, 24.3, 27.5, 29.1, 29.3, 54.7, 56.7, 58.9, 75.8, 78.4, 95.1, 111.1, 118.9, 121.3, 121.8, 126.2, 127.7, 129.1, 129.5, 130.6, 132.3, 137.8, 138.3, 148.4, 150.8, 153.ǂ

ǂ 1 peak obscured.

HRMS (ES$^+$): $[C_{31}H_{33}N_4]^+$ ($[M+H]^+$) requires 461.2700; found 461.2695.

(2*RS*,3*RS*)-1-Acetyl-3-phenyl-2-(5-(7-(pyrrolidin-1-yl)heptyl)pyridin-3-yl)indoline-3-carbonitrile **6**

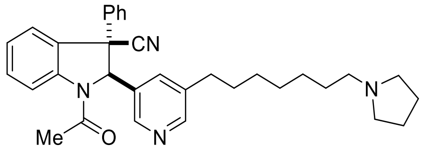

Indoline **6** was prepared according to general procedure 8, using indoline **4** (50 mg, 0.099 mmol) and Pd/C (wet degussa type, 5 mg). The reaction mixture was stirred under a H_2 atmosphere for 4.5 h. Purification by flash pressure column chromatography (5% MeOH/CH_2Cl_2 + 0.5% aq. NH_4OH) afforded indoline **6** as a colorless oil (37 mg, 0.073 mmol, 74%). The non-racemic forms of indoline **6** (e.r. > 99:1) were prepared from indolines **(*R*,*R*)-4** and **(*S*,*S*)-4**, following the same procedure.

^{1}H NMR (400 MHz, C_6D_6): δ 0.98-1.17 (4H, m), 1.22-1.33 (4H, m), 1.38-1.55 (5H, m), 1.65 (4H, m), 2.09 (2H, t, *J* = 7.3 Hz), 2.36-2.48 (6H, m), 5.13 (1H, br. s), 6.74 (1H, td, *J* = 7.7 Hz, 0.9 Hz), 6.94-7.01 (3H, m), 7.03 (1H, d, *J* = 7.7 Hz), 7.10 (1H, t, *J* = 7.7 Hz), 7.20-7.28 (3H, m), 8.46 (1 H,d, *J* = 1.7 Hz), 8.50 (1H, d, *J* = 2.0 Hz), 8.69 (1H, br. s).

^{13}C NMR (100.6 MHz, C_6D_6): δ 23.8, 24.3, 28.2, 29.6, 29.9, 29.9, 31.4, 33.1, 54.8, 57.0, 58.2, 74.5, 117.7, 119.1, 126.0, 126.3, 126.4, 129.4, 130.2, 131.3, 133.3, 133.7, 139.2, 141.1, 144.0, 146.6, 152.3, 168.5.‡

‡ 1 peak obscured.

HRMS (ESI$^+$, m/z): $C_{33}H_{39}N_4O^+$ ($[M+H]^+$) calc. 507.3118; found 507.3106.

$[\alpha]_D^{25.0}$ (*S*,*S*) = -87 (*c* = 0.1, $CHCl_3$).

Chiral HPLC: (Chiralpak ODH, 15% ^{i}PrOH, 85% hexane, 1.3 mL/min, λ = 254 nm, 20 µL injection):

τ_R (*R*,*R*) = 8.7 min, τ_R (*S*,*S*) = 11.4 min.

(2*SR*,3*RS*)-1-acetyl-3-phenyl-2-(5-(7-(pyrrolidin-1-yl)heptyl)furan-2-yl)indoline-3-carbonitrile **7**

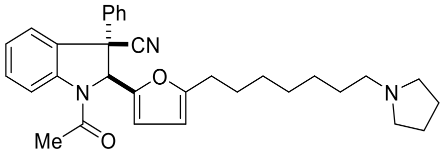

Indoline **7** was prepared according to general procedure 8, using indoline **132** (41 mg, 0.083 mmol) and Pd/C (wet degussa type, 4 mg) in MeOH (1.5 mL). The reaction mixture was stirred under a H_2 atmosphere for 4.5 h. Purification by flash pressure column chromatography (4.5% MeOH/CH_2Cl_2 + 0.5% aq. NH_4OH) afforded indoline **7** as a colorless oil (23 mg, 0.046 mmol, 56%).

^{1}H NMR (500 MHz, C_6D_6, 348 K): δ 1.17-1.28 (4H, m), 1.33 (2H, quin, *J* = 7.4 Hz), 1.47-1.60 (7H, m), 1.64 (4H, m), 2.35-2.48 (8H, m), 5.23 (1H, br. s), 5.73 (1H, d, *J* = 3.3 Hz), 5.93 (1H, br. s), 6.75 (1H, td, *J* = 7.6 Hz, 0.9 Hz), 6.0-7.0 (3H, m), 7.04-7.15 (2H, m), 7.31 (2H, m), 8.77 (1H, br. s).

^{13}C NMR (125.75 MHz, C_6D_6): δ 23.4, 24.3, 28.2, 28.5, 28.6, 29.7, 29.9, 30.0, 54.7, 57.0, 57.0, 71.2, 106.7, 110.2, 118.1, 119.0, 125.6, 126.0, 126.3, 129.2, 130.0, 130.9, 140.8, 143.6, 148.8, 158.7, 168.4.‡

‡ 1 peak obscured.

HRMS (ESI$^+$, m/z): $[C_{32}H_{38}N_3O_2]^+$ ($[M+H]^+$) calc. 496.2959; found 496.2940.

(2*SR*,3*SR*)-1-Acetyl-3-phenyl-2-(5-(7-(pyrrolidin-1-yl)heptyl)furan-2-yl)indoline-3-carbonitrile **138**

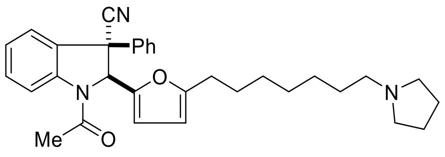

Indoline **138** was prepared according to general procedure 8, using indoline **133** (40 mg, 0.081 mmol) and Pd/C (wet degussa type, 4 mg) in MeOH (1.5 mL). The reaction mixture was stirred under a H_2 atmosphere for 1 h. Purification by flash pressure column chromatography (4% MeOH/CH_2Cl_2 + 0.5% aq. NH_4OH) afforded indoline **138** as a colorless oil (26 mg, 0.052 mmol, 64%).

^{1}H NMR (500 MHz, C_6D_6, 348 K): δ 1.00 (2H, quin, *J* = 7.1 Hz), 1.04-1.22 (4H, m), 1.32 (2H, quin, *J* = 7.4 Hz), 1.50 (2H, quin, *J* = 7.4 Hz), 1.65 (4H, m), 1.74 (3H, s), 2.04 (2H, t, *J* = 7.4 Hz), 2.38-2.48 (6H, m), 5.38 (1H, d, *J* = 3.0 Hz), 5.60 (1H, d, *J* = 3.0 Hz), 5.70 (1H, s), 6.78 (1H, t, *J* = 7.7 Hz), 6.86 (1H, d, *J* = 7.7 Hz), 6.95-7.02 (3H, m), 7.14 (1H, t, *J* = 7.7 Hz), 7.24 (2H, app. d, *J* = 7.5 Hz), 8.54 (1H, br. s).

^{13}C NMR (125.75 MHz, C_6D_6): δ 23.6, 24.5, 28.2, 28.2, 28.4, 29.7, 29.9, 29.9, 54.7, 55.6, 56.9, 70.6, 106.0, 110.9, 118.2, 121.4, 125.1, 126.3, 129.0, 129.0, 129.0, 131.1, 134.7, 145.1, 147.4, 157.9, 168.6.ǂ

ǂ 1 peak obscured.

HRMS (ESI$^+$, m/z): $[C_{32}H_{38}N_3O_2]^+$ ($[M+H]^+$) calc. 496.2959; found 496.2938.

(2*SR*,3*RS*)-1-Acetyl-3-phenyl-2-(4-(7-(pyrrolidin-1-yl)heptyl)furan-2-yl)indoline-3-carbonitrile **8**

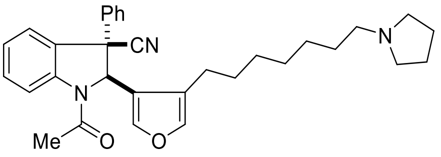

Indoline **8** was prepared according to general procedure 8, using indoline **134** (10 mg, 0.020 mmol) and Pd/C (wet degussa type, 1.5 mg) in MeOH (1.0 mL). The reaction mixture was stirred under a H_2 atmosphere for 16 h. Purification by flash pressure column chromatography (4% MeOH/CH_2Cl_2 + 0.5% aq. NH_4OH) afforded indoline **8** as a colorless oil (6.0 mg, 0.012 mmol, 60%).

^{1}H NMR (500 MHz, C_6D_6): δ 1.14-1.25 (4H, m), 1.26-1.38 (4H, m), 1.48-1.59 (5H, m), 1.65 (4H, m), 2.06 (2H, t, *J* = 7.6 Hz), 2.39-2.46 (6H, m), 5.22 (1H, br. s), 6.01 (1H, br. s), 6.74 (1H, td, *J* = 7.6 Hz, 0.9 Hz), 6.91-6.98 (4H, m), 7.06-7.14 (2H, m), 7.31 (2H), 8.80 (1H, br. s).

^{13}C NMR (125.75 MHz, C_6D_6): δ 23.4, 24.3, 25.2, 28.3, 29.9, 30.0, 30.0, 30.2, 54.8, 57.0, 57.0, 71.2, 110.8, 118.2, 118.9, 125.7, 126.1, 126.3, 127.3, 129.3, 130.1, 131.0, 140.4, 140.8, 143.7, 150.8, 168.4.‡

‡ 1 peak obscured.

HRMS (ESI$^+$, m/z): $[C_{32}H_{34}N_3O_2]^+$ ($[M+H]^+$) calc. 492.2645; found 492.2638.

(2*RS*,3*RS*)-1-(2-cyanoacetyl)-3-phenyl-2-(5-(7-(pyrrolidin-1-yl)heptyl)pyridin-3-yl)indoline-3-carbonitrile **139**

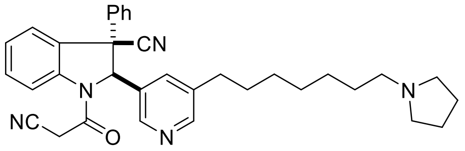

Indoline **139** was prepared according to general procedure 8 using indoline **135** (16 mg, 0.030 mmol) and Pd/C (wet degussa type, 2 mg) in MeOH (2 mL). The reaction mixture was stirred at rt under a H_2 atmosphere for 4 h. Purification by flash pressure column chromatography (4.5% MeOH/CH_2Cl_2 + 0.5% aq. NH_4OH) afforded indoline **139** as a colorless oil (14 mg, 0.026 mmol, 87%, contains minor impurities).

^{1}H NMR (400 MHz, C_6D_6, 348 K): δ 1.12 (2H, quin, *J* = 7.5 Hz), 1.19 (2H, quin, *J* = 7.5 Hz), 1.27-1.41 (4H, m), 1.50 (2H, quin, *J* = 7.4 Hz), 1.65 (4H, m), 2.21 (2H, t, *J* = 7.6 Hz), 2.37-2.49 (8H, m), 5.12 (1H, br. s), 6.78 (1H, t, *J* = 7.6 Hz), 6.97-7.06 (4H, m), 7.09 (1H, t, *J* = 7.9 Hz), 8.23 (1H, br. s), 8.35 (1H, s), 8.49 (1H, s).[ƚ]

^{13}C NMR (100.6 MHz, C_6D_6, 348 K): δ 24.5, 26.8, 28.2, 29.5, 29.8, 29.9, 31.1, 33.2, 54.7, 56.9, 58.7, 74.5, 112.8, 117.8, 118.6, 126.3, 126.6, 127.1, 129.7, 130.4, 131.5, 132.5, 133.3, 139.5, 140.5, 143.0, 146.3, 153.0, 160.4.[‡]

ƚ 3 peaks obscured.

‡ 1 peak obscured.

HRMS (ES$^+$): $[C_{34}H_{38}N_5O]^+$ ($[M+H]^+$) calc. 532.3071; found 532.3073.

(2*RS*,3*RS*)-1-(2-(1,3-dioxoisoindolin-2-yl)acetyl)-3-phenyl-2-(5-(7-(pyrrolidin-1-yl)heptyl)pyridin-3-yl)indoline-3-carbonitrile **140**

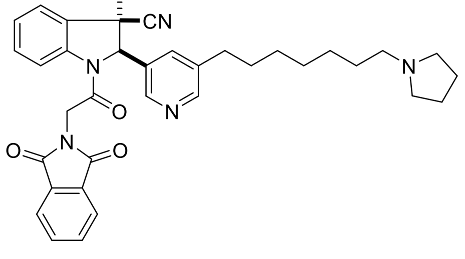

Indoline **140** was prepared according to general procedure 8, using indoline **136** (27 mg, 0.042 mmol) and Pd/C (wet degussa type, 3 mg) in MeOH (2.5 mL). The reaction mixture was stirred at rt under a H_2 atmosphere for 24 h. Purification by flash pressure column chromatography (4.5% MeOH/CH_2Cl_2 + 0.5% aq. NH_4OH) afforded indoline **140** as a colorless oil (17 mg, 0.026 mmol, 62%).

^{1}H NMR (400 MHz, C_6D_6, 348 K): δ 1.13-1.28 (4H, m), 1.28-1.39 (2H, quin, *J* = 7.3 Hz), 1.40-1.56 (4H, m), 1.64 (4H, m), 2.28 (2H, t, *J* = 6.9 Hz), 2.36-2.49 (6H, m), 3.99 (1H, d, *J* = 16.4 Hz), 4.19 (1H, d, *J* = 16.4 Hz), 5.51 (1H, s), 6.76 (1H, t, *J* = 7.6 Hz), 6.88-6.96 (2H, m), 6.98-7.12 (5H, m), 7.32 (2H, app. d, *J* = 7.6 Hz), 7.36-7.45 (3H, m), 8.27 (1H, br. s), 8.47-8.58 (2H, m).

^{13}C NMR (100.6 MHz, C_6D_6, 348 K): δ 24.4, 28.2, 29.6, 29.8, 30.0, 31.2, 33.3, 41.2, 54.7, 56.9, 59.0, 73.9, 117.9, 118.7, 123.6, 126.2, 126.3, 126.5, 129.4, 130.3, 130.4, 131.2, 132.9, 133.1, 133.5, 133.9, 139.4, 140.8, 143.4, 146.8, 152.6, 165.1, 167.5.

HRMS (ES$^+$): $[C_{41}H_{42}N_5O_3]^+$ ($[M+H]^+$) requires 652.3288; found 652.3281.

(2*RS*,3*RS*)-1-Glycyl-3-phenyl-2-(5-(7-(pyrrolidin-1-yl)heptyl)pyridin-3-yl)indoline-3-carbonitrile **12**

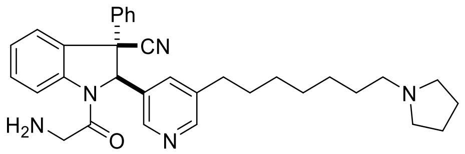

$N_2H_4{\cdot}H_2O$ (7.0 µL, 0.13 mmol) was added dropwise to a solution of indoline **140** (17 mg, 0.026 mmol) in EtOH (2 mL) and stirred at 60 °C for 1 h. The mixture was filtered and concentrated. CH_2Cl_2 was added to the remaining residue, the resulting precipitate was removed by filtration, and the filtrate was concentrated. This process was repeated 4 times. Purification by flash pressure column chromatography (5.5% MeOH/CH_2Cl_2 + 0.5% aq. NH_4OH) to afford indoline **12** as a pale yellow oil (9.4 mg, 0.018 mmol, 69%, contains minor impurities).

^{1}H NMR (400 MHz, C_6D_6): δ 0.77 (3H, br. s, includes H_2O), 1.05 (2H, quin, *J* = 7.2 Hz), 1.12 (2H, quin, *J* = 7.2 Hz), 1.28 (4H, m), 1.50 (2H, quin, *J* = 7.6 Hz), 1.65 (4H, m), 2.10 (2H, t, *J* = 7.5 Hz), 2.36-2.48 (6H, m), 2.72 (2H, br. s), 5.20 (1H, br. s), 6.73 (1H, td, *J* = 7.7 Hz, 1.0 Hz), 6.92-6.99 (3H, m), 7.03 (1H, dd, *J* = 7.7 Hz, 0.8 Hz), 7.10 (1H, t, *J* = 7.7 Hz), 7.19-7.24 (3H, m), 8.46 (1H, d, *J* = 1.8 Hz), 8.50 (1H, d, *J* = 2.0 Hz), 8.53 (1H, br. s).

^{13}C NMR (100.6 MHz, C_6D_6): δ 24.3, 28.2, 29.5, 29.8, 30.0, 31.3, 33.1, 46.2, 54.8, 57.0, 58.4, 73.1, 117.6, 119.1, 126.2, 126.3, 126.5, 129.4, 130.2, 131.4, 133.3, 133.5, 139.2, 141.0, 143.9, 146.5, 152.4, 172.1.ⱡ

ⱡ 1 peak obscured.

HRMS (ES$^+$): $[C_{33}H_{40}N_5O]^+$ ($[M+H]^+$) requires 522.3227; found 522.3227.

(2*RS*,3*RS*)-3-Phenyl-2-(5-(7-(pyrrolidin-1-yl)heptyl)pyridin-3-yl)indoline-3-carbonitrile **142**

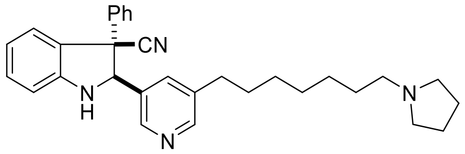

Indoline **142** was prepared according to general procedure 8 using indoline **137** (20 mg, 0.04 mmol) and Pd/C (degussa type, 2 mg) in MeOH (1.5 mL). The reaction mixture was stirred at rt under a H_2 atmosphere for 16 h. Purification by flash pressure column chromatography (5% MeOH/CH_2Cl_2 + 0.5% aq. NH_4OH) afforded indoline **142** as a colorless oil (14 mg, 0.029 mmol, 72%).

^{1}H NMR (500 MHz, C_6D_6): δ 1.17-1.29 (4H, m), 1.35 (2H, quin, *J* = 7.5 Hz), 1.46-1.57 (4H, m), 1.63 (4H, m), 2.31-2.49 (8H, m), 3.31 (1H, d, *J* = 3.0 Hz), 4.45 (1H, d, *J* = 3.0 Hz), 6.47 (1H, d, *J* = 8.1 Hz), 6.61 (1H, td, *J* = 7.6 Hz, 0.9 Hz), 6.75 (1H, d, *J* = 7.6 Hz), 6.97-7.06 (4H, m), 7.28 (2H, m), 7.83 (1H, t, *J* = 2.0 Hz), 8.11 (1H, d, *J* = 2.0 Hz), 8.57 (1H, d, *J* = 2.0 Hz).

^{13}C NMR (125.75 MHz, C_6D_6): δ 24.3, 28.1, 29.7, 29.8, 30.0, 31.7, 33.5, 54.8, 57.0, 59.1, 76.4, 111.1, 119.3, 121.3, 126.3, 127.9, 129.0, 129.4, 130.6, 132.1, 135.7, 138.0, 138.3, 147.8, 151.2, 151.8.ⱡ

ⱡ 1 peak obscured.

HRMS (ES$^+$): $[C_{31}H_{37}N_4]^+$ ($[M+H]^+$]) calc. 465.3013; found 465.3013.

(2*RS*,3*SR*)-1-Acetyl-2-(5-((7-bromoheptyl)oxy)pyridin-3-yl)-3-phenylindoline-3-carbonitrile **143**

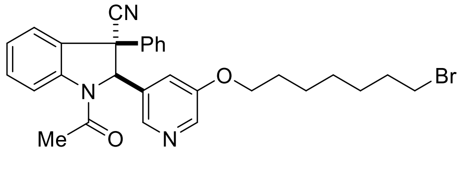

K_2CO_3 (146 mg, 1.06 mmol) was added to a solution of indoline **110** (75 mg, 0.21 mmol) in acetone (15 mL), followed by 1,7-dibromoheptane (140 µL, 0.84 mmol). The mixture was stirred under reflux for 16 h, allowed to cool to rt, and concentrated. The remaining residue was dissolved in EtOAc, washed three times with H_2O, then brine. The organic layer was dried over Na_2SO_4, filtered, and concentrated. Purification by flash pressure column chromatography (40% EtOAc/petroleum ether) afforded indoline **143** as a pale yellow oil (36 mg, 0.068 mmol, 32%, contains minor impurities).

^{1}H NMR (500 MHz, C_6D_6, 348 K): δ 0.99 (2H, quin, *J* = 7.0 Hz), 1.07 (2H, quin, *J* = 7.0 Hz), 1.15 (2H, quin, *J* = 7.4 Hz), 1.33 (2H, quin, *J* = 7.4 Hz), 1.69 (3H, s), 3.00 (2H, t, *J* = 7.0 Hz), 3.33 (2H, app. m), 5.71 (1H, s), 6.36 (1H, s), 6.76 (1H, t, *J* = 7.7 Hz), 6.78-6.87 (4H, m), 7.02 (2H, app. d, *J* = 7.8 Hz), 7.11 (1H, td, *J* = 7.8 Hz, 1.3 Hz), 7.72 (1H, s), 8.08 (1H, d, *J* = 2.4 Hz), 8.32 (1H, br. s).

^{13}C NMR (125.75 MHz, C_6D_6, 348 K): δ 23.7, 25.9, 28.2, 28.5, 29.1, 32.9, 33.1, 55.8, 68.5, 73.2, 117.4, 119.6, 121.3, 125.2, 126.3, 128.6, 128.9, 129.0, 131.1, 132.8, 133.9, 138.6, 141.3, 144.8, 155.2, 168.2.[ǂ]

ǂ 1 peak obscured.

HRMS (ES$^+$): $[C_{29}H_{31}{}^{79}BrN_3O_3]^+$ ($[M+H]^+$) calc. 532.1594; found 532.1592. $[C_{29}H_{31}{}^{81}BrN_3O_3]^+$ ($[M+H]^+$) calc. 534.1574; found 534.1571.

(2*RS*,3*RS*)-1-Acetyl-2-(5-((7-bromoheptyl)oxy)pyridin-3-yl)-3-phenylindoline-3-carbonitrile **144**

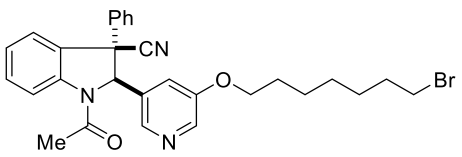

K_2CO_3 (214 mg, 1.55 mmol) was added to a solution of indoline **17** (110 mg, 0.31 mmol) in acetone (20 mL), followed by 1,7-dibromoheptane (0.20 mL, 1.2 mmol). The mixture was stirred under reflux for 8 h, allowed to cool to rt, and concentrated. The remaining residue was dissolved in EtOAc, washed three times with H_2O, then brine. The organic layer was dried over Na_2SO_4, filtered, and concentrated. Purification by flash pressure column chromatography (40% EtOAc/petroleum ether) afforded indoline **144** as a pale yellow oil (63 mg, 0.12 mmol, 38%, contains minor impurities). The non-racemic forms of indoline **144** were prepared from **(*R*,*R*)-17** and **(*S*,*S*)-17**, following an identical protocol.

^{1}H NMR (500 MHz, C_6D_6, 348 K): δ 0.96 (2H, quin, *J* =7.2 Hz), 1.04 (2H, quin, *J* = 7.5 Hz), 1.10 (2H, quin, *J* = 7.5 Hz), 1.35 (2H, quin, *J* = 7.4 Hz), 1.44-1.57 (5H, m), 2.99 (2H, t, *J* = 6.8 Hz), 3.47 (2H, app. td, *J* = 6.7 Hz, 1.6 Hz), 5.23 (1H, s), 6.76 (1H, td, *J* = 7.7 Hz, 0.9 Hz), 6.96-7.03 (3H, m), 7.04 (1H, t, *J* = 2.2 Hz), 7.06 (1H, d, *J* = 7.6 Hz), 7.10 (1H, td, *J* = 7.9 Hz, 1.2 Hz), 7.24 (2H, m), 8.28 (1H, d, *J* = 1.9 Hz), 8.38 (1H, br. s), 8.43 (1H, d, *J* = 2.5 Hz).

^{13}C NMR (125.75 MHz, C_6D_6, 348 K): δ 23.7, 26.0, 28.4, 28.8, 29.3, 33.1, 34.0, 58.1, 68.5, 74.2, 117.6, 118.6, 119.1, 126.0, 126.2, 126.4, 129.4, 130.2, 131.3, 134.8, 139.8, 140.9, 141.2, 143.8, 156.2, 168.4.[ǂ]

ǂ 1 peak obscured.

HRMS (ES$^+$): $[C_{29}H_{31}{}^{79}BrN_3O_2]^+$ ($[M+H]^+$) calc. 532.1594; found 532.1580. $[C_{29}H_{31}{}^{81}BrN_3O_2]^+$ ($[M+H]^+$) calc. 534.1574; found 534.1562.

$[\boldsymbol{\alpha}]_{\boldsymbol{D}}^{\mathbf{25.0}}$ (*R*,*R*) = +26 (*c* = 0.1, $CHCl_3$); $[\boldsymbol{\alpha}]_{\boldsymbol{D}}^{\mathbf{25.0}}$ (*S*,*S*) = -34 (*c* = 0.1, $CHCl_3$).

(2*RS*,3*SR*)-1-Acetyl-3-phenyl-2-(5-((7-(pyrrolidin-1-yl)heptyl)oxy)pyridin-3-yl)indoline-3-carbonitrile **145**

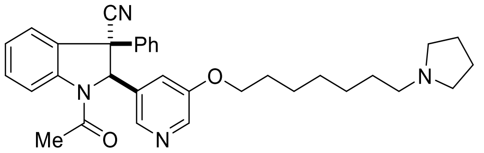

K_2CO_3 (29 mg, 0.21 mmol) was added to a solution of indoline **143** (22 mg, 0.04 mmol) in acetonitrile (3.0 mL), followed by pyrrolidine (14 µL, 0.17 mmol). The mixture was stirred at 60 °C for 2.5 h. H_2O was added, and the mixture was extracted with EtOAc. The combined organic extracts were washed with brine, dried over Na_2SO_4, filtered, and concentrated. Purification by flash pressure column chromatography (4.5-5% MeOH/CH_2Cl_2 + 0.5% aq. NH_4OH) afforded indoline **145** as a colorless oil (14 mg, 0.027 mmol, 65%).

^{1}H NMR (500 MHz, C_6D_6, 348 K): δ 1.15-1.23 (4H, m), 1.32 (2H, quin, *J* = 6.9 Hz), 1.39 (2H, quin, *J* = 6.8 Hz), 1.50 (2H, quin, *J* = 7.3 Hz), 1.61-1.69 (7H, m), 2.39-2.46 (6H, m), 3.32 (2H, app. m), 5.67 (1H, br. s), 6.32 (1H, br. s), 6.74 (1H, td, *J* = 7.7 Hz, 1.0 Hz), 6.77-6.86 (4H, m, *H*4), 7.01 (2H, app. d, *J* = 7.3 Hz), 7.10 (1H, td, *J* = 7.7 Hz, 1.2 Hz), 7.72 (1H, br. s), 8.09 (1H, d, *J* = 2.8 Hz), 8.36 (1H, br. s).

^{13}C NMR (125.75 MHz, C_6D_6, 348 K): δ 24.1, 24.3, 26.5, 28.1, 29.5, 29.8, 29.9, 54.8, 55.8, 56.9, 68.5, 73.0, 117.7, 119.6, 121.7, 125.6, 126.6, 129.0, 129.2, 129.2, 131.5, 132.9, 133.9, 138.7, 141.1, 144.9, 155.4, 168.6.[‡]

‡ 1 peak obscured.

HRMS (ES$^+$): $[C_{33}H_{39}N_4O_2]^+$ ($[M+H]^+$) calc. 523.3068; found 523.3068.

(2*RS*,3*SR*)-1-Acetyl-3-phenyl-2-(5-((7-(pyrrolidin-1-yl)heptyl)oxy)pyridin-3-yl)indoline-3-carbonitrile **3**

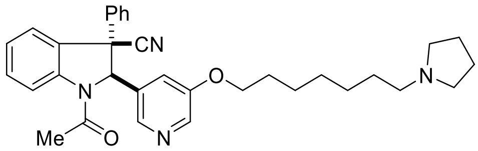

K_2CO_3 (58 mg, 0.42 mmol) was added to a solution of indoline **144** (45 mg, 0.085 mmol) in acetonitrile (6 mL), followed by pyrrolidine (28 µL, 0.34 mmol). The mixture was stirred at 60 °C for 3 h. H_2O was added, and the mixture was extracted with EtOAc. The combined organic extracts were washed with brine, dried over Na_2SO_4, filtered, and concentrated. Purification by flash pressure column chromatography (5% MeOH/CH_2Cl_2 + 0.5% aq. NH_4OH) afforded indoline **3** as a colorless oil (24 mg, 0.046 mmol, 54%). The non-racemic forms of indoline **3** were prepared from **(*R*,*R*)-3** and **(*S*,*S*)-3**, following an identical protocol (e.r. > 99:1).

^{1}H NMR (500 MHz, C_6D_6, 348 K): δ 1.18 (4H, m), 1.29 (2H, quin, *J* = 7.1 Hz), 1.43 (2H, quin, *J* = 6.8 Hz), 1.46-1.54 (5H, m), 1.65 (4H, m), 2.37-2.48 (6H, m), 3.50 (2H, app. td, *J* = 6.4 Hz, 1.7 Hz), 5.22 (1H, br. s), 6.76 (1H, td, *J* = 7.6 Hz, 0.9 Hz), 6.96-7.04 (4H, m), 7.05-7.13 (2H, m), 7.24 (2H, m), 8.28 (1H, d, *J* = 1.2 Hz), 8.39 (1H, br. s), 8.42 (1H, d, *J* = 2.5 Hz).

^{13}C NMR (125.75 MHz, C_6D_6): δ 23.8, 24.3, 26.4, 28.1, 29.6, 29.8, 30.0, 54.8, 56.9, 58.2, 68.7, 74.3, 117.6, 118.7, 119.2, 126.0, 126.3, 126.4, 128.7, 129.4, 130.2, 131.3, 134.8, 139.9, 141.0, 141.1, 143.9, 156.3, 168.5.

HRMS (ES$^+$): $[C_{33}H_{39}N_4O_2]^+$ ($[M+H]^+$) calc. 523.3068; found 523.3063.

$[\boldsymbol{\alpha}]_{\boldsymbol{D}}^{\mathbf{25.0}}$ (*R*,*R*) = +57 (*c* = 0.1, $CHCl_3$); $[\boldsymbol{\alpha}]_{\boldsymbol{D}}^{\mathbf{25.0}}$ (*S*,*S*) = -48 (*c* = 0.1, $CHCl_3$).

Chiral HPLC: (Chiralpak ODH, 15% ^{i}PrOH, 85% hexane, 1.3 mL/min, λ = 254 nm, 20 µL)

τ_R (*R*,*R*) = 11.8 min, τ_R (*S*,*S*) = 16.7 min.

(2*RS*,3*RS*)-1-Acetyl-2-(5-((7-(dimethylamino)heptyl)oxy)pyridin-3-yl)-3-phenylindoline-3-carbonitrile **146**

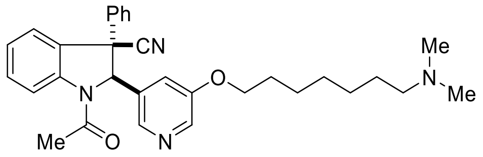

Dimethylamine hydrochloride (20 mg, 0.23 mmol) was added to a solution of indoline **144** (30 mg, 0.056 mmol) in acetonitrile, followed by K_2CO_3 (40 mg, 0.28 mmol). The mixture was stirred 50 °C for 4.5 h. H_2O was added, and the mixture was extracted with EtOAc. The combined organic extracts were washed with brine, dried over Na_2SO_4, filtered, and concentrated. Purification by flash pressure column chromatography (5% MeOH/CH_2Cl_2 + 0.5% aq. NH_4OH) afforded indoline **146** as a colorless oil (19 mg, 0.038 mmol, 68%).

^{1}H NMR (500 MHz, C_6D_6, 348 K): δ 1.11-1.21 (4H, m), 1.25 (2H, quin, *J* = 7.5 Hz), 1.40 (2H, quin, *J* = 7.2 Hz), 1.46 (2H, quin, *J* = 7.3 Hz), 1.54 (3H, s), 2.13 (6H, s), 2.18 (2H, t, *J* = 7.1 Hz), 3.54 (2H, t, *J* = 6.5 Hz), 5.24 (1H, s), 6.77 (1H, t, *J* = 7.6 Hz), 6.95-7.05 (4H, m), 7.06-7.13 (2H, m), 7.24 (2H, app. d, *J* = 7.6 Hz), 8.27 (1H, s), 8.35 (1H, br. s), 8.42 (1H, d, *J* = 2.2 Hz).

^{13}C NMR (125.75 MHz, C_6D_6): δ 23.7, 26.5, 28.0, 28.4, 29.6, 29.9, 45.9, 58.3, 60.4, 69.2, 74.8, 117.7, 119.1, 119.2, 125.9, 126.3, 126.4, 129.4, 130.0, 130.2, 131.3, 134.8, 140.4, 141.3, 141.3, 144.1, 156.5, 168.3.

HRMS (ES$^+$): $[C_{31}H_{37}N_4O_2]^+$ ($[M+H]^+$) calc. 497.2911; found 497.2909.

(2*RS*,3*RS*)-1-Acetyl-2-(5-(2-(2-(2-bromoethoxy)ethoxy)ethoxy)pyridin-3-yl)-3-phenylindoline-3-carbonitrile **147**

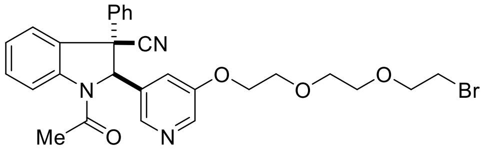

K_2CO_3 (272 mg, 1.97 mmol) was added to a solution of indoline **17** (140 mg, 0.39 mmol), followed by 1,2-bis(2-bromoethoxy)ethane (410 mg, 1.58 mmol). The mixture was stirred at 50 °C for 2 h, allowed to cool to rt, and concentrated. The remaining residue was dissolved in EtOAc, washed three times with H_2O, then brine. The organic layer was dried over Na_2SO_4, filtered, and concentrated. Purification by flash pressure column

chromatography (3.5% MeOH/CH_2Cl_2) afforded indoline **147** as an orange paste (80 mg, 0.15 mmol, 37%, contains minor impurities).

^{1}H NMR (500 MHz, C_6D_6, 348 K): δ 1.53 (3H, s), 3.06 (2H, t, *J* = 6.3 Hz), 3.24-3.33 (4H, m), 3.35 (2H, app. s), 3.41 (2H, t, *J* = 6.3 Hz), 3.65 (2H, app. m), 5.22 (1H, s), 6.77 (1H, t, *J* = 7.6 Hz), 6.95-7.04 (3H, m), 7.04-7.08 (2H, m), 7.11 (1H, t, *J* = 7.8 Hz), 7.23 (2H, app. d, *J* = 7.7 Hz), 8.26 (1H, app. s), 8.33 (1H, br. s), 8.41 (1H, d, *J* = 2.3 Hz).

^{13}C NMR (125.75 MHz, C_6D_6): δ 23.7, 31.0, 58.1, 68.2, 69.9, 70.9, 71.2, 71.6, 74.2, 117.6, 119.1, 119.2, 125.9, 126.2, 126.2, 129.3, 130.1, 131.2, 134.7, 139.8, 141.0, 141.3, 143.8, 156.1, 168.4.

HRMS (ES$^+$): $[C_{28}H_{29}{}^{79}BrN_3O_4]^+$ ($[M+H]^+$) calc. 550.1336; found 550.1326. $[C_{28}H_{29}{}^{81}BrN_3O_4]^+$ ($[M+H]^+$) calc. 552.1316; found 552.1308.

(2*RS*,3*RS*)-1-Acetyl-3-phenyl-2-(5-(2-(2-(2-(pyrrolidin-1-yl)ethoxy)ethoxy)ethoxy)pyridin-3-yl)indoline-3-carbonitrile **148**

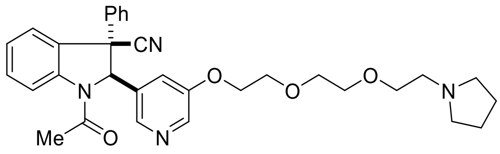

K_2CO_3 (63 mg, 0.45 mmol) was added to a solution of indoline **147** (50 mg, 0.091 mmol) in acetonitrile (6 mL), followed by pyrrolidine (30 µL, 0.36 mmol). The mixture was stirred at 60 °C for 3 h. H_2O was added, and the mixture was extracted with EtOAc. The combined organic extracts were washed with brine, dried over Na_2SO_4, filtered, and concentrated. Purification by flash pressure column chromatography (5% MeOH/CH_2Cl_2 + 0.5% aq. NH_4OH) afforded indoline **148** as a pale yellow oil (38 mg, 0.070 mmol, 77%).

^{1}H NMR (500 MHz, C_6D_6, 348 K): δ 1.55 (3H, s), 1.56-1.64 (4H, m), 2.46 (4H, m), 2.63 (2H, t, *J* = 6.0 Hz), 3.36-3.47 (6H, m), 3.51 (2H, t, *J* = 6.0 Hz), 3.69 (2H, app. m), 5.24 (1H, s), 6.78 (1H, t, *J* = 7.4 Hz), 6.96-7.09 (5H, m), 7.12 (1H, t, *J* = 8.0 Hz), 7.24 (2H, m), 8.26 (1H, app. s), 8.33 (1H, br. s), 8.41 (1H, d, *J* = 2.8 Hz).

^{13}C NMR (125.75 MHz, C_6D_6, 348 K): δ 23.7, 24.5, 55.1, 56.4, 58.3, 68.9, 70.1, 71.2, 71.5, 71.6, 74.7, 117.7, 119.1, 119.7, 125.9, 126.3, 126.4, 129.4, 129.9, 130.2, 131.3, 134.8, 140.5, 141.3, 141.5, 144.0, 156.3, 168.3.

HRMS (ESI$^+$, m/z): $[C_{32}H_{37}N_4O_4]^+$ ($[M+H]^+$) calc. 541.2809; found 541.2788.

(2*RS*,3*RS*)-1-Acetyl-2-(5-((Z)-7-hydroxyhept-1-en-1-yl)pyridin-3-yl)-3-phenylindoline-3-carbonitrile **149**

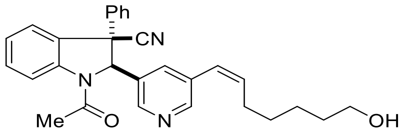

Indoline **149** was prepared according to a modified literature procedure.[17] $InCl_3$ (66 mg, 0.30 mmol) was dissolved in THF (0.9 mL) and cooled to -78 °C. DIBAL-H (1.0 M in hexanes, 0.28 mL, 0.28 mmol) was added and stirred at -78 °C for 45 min. A solution of 6-heptyn-1-ol (22 mg, 0.2 mmol) in THF (0.6 mL) was added, followed by Et_3B (1.0 M in hexanes, 0.19 mL, 0.19 mmol), and the mixture was stirred at -78 °C or 4 h. The reaction was allowed to warm to rt, and a solution of indoline **94** (110 mg, 0.26 mmol) in DMF (0.9 mL) was added, followed by a solution of $Pd(PPh_3)_4$ in THF (4.6 mM, 0.35 mL, 0.0016 mmol). The reaction mixture was stirred at 60 °C for 7 h, then diluted with EtOAc, washed with $NaHCO_3$ (saturated aq.), and extracted with EtOAc. The combined organic extracts were washed with brine, dried over Na_2SO_4, filtered, and concentrated. Purification by flash pressure column chromatography (3.5% MeOH/CH_2Cl_2) afforded indoline **149** as a colorless oil (42 mg, 0.093 mmol, 58%, contains minor impurities).

^{1}H NMR (500 MHz, C_6D_6, 348 K): δ 0.94 (1H, t, *J* = 5.5 Hz, O*H*), 1.13-1.24 (4H, m), 1.33 (2H, quin, *J* = 6.7 Hz), 1.52 (3H, s), 2.02 (2H, app. q, *J* = 7.4 Hz), 3.38 (2H, app. q, *J* = 6.3 Hz), 5.24 (1H, s), 5.53 (1H, dt, *J* = 11.7 Hz, 7.6 Hz), 6.07 (1H, d, *J* = 11.7 Hz), 6.80 (1H, t, *J* = 7.6 Hz), 6.96-7.05 (3H, m), 7.08 (1H, d, *J* = 7.6 Hz), 7.14 (1H, m), 7.24 (2H, m), 7.42 (1H, s), 8.34 (1H, br. s), 8.46 (1H, s), 8.59 (1H, s).

^{13}C NMR (125.75 MHz, C_6D_6, 348 K): δ 23.7, 26.1, 29.0, 30.0, 33.3, 58.3, 63.0, 74.8, 117.7, 119.1, 125.6, 126.0, 126.3, 126.5, 129.4, 129.9, 130.2, 131.3, 133.2, 133.7, 134.7, 136.9, 141.2, 144.0, 147.1, 152.2, 168.3.

HRMS (ES$^+$): $[C_{29}H_{30}N_3O_2]^+$ ($[M+H]^+$) calc. 452.2333; found 452.2326.

(*Z*)-7-(5-((2*RS*,3*RS*)-1-acetyl-3-cyano-3-phenylindolin-2-yl)pyridin-3-yl)hept-6-en-1-yl 4-methylbenzenesulfonate **150**

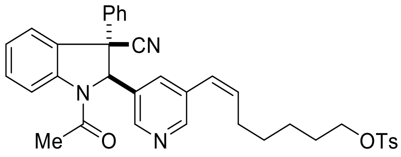

p-Toluenesulfonyl chloride (38 mg, 0.20 mmol) was added to a solution of indoline **149** (30 mg, 0.066 mmol) and pyridine (32 µL, 0.41 mmol) in CH_2Cl_2 (5 mL) at 0 °C. The reaction mixture was stirred at 0 °C for 10 min, allowed to warm to rt, and stirred for a further 20 h. H_2O (5 ml) was added, and the mixture was extracted 3 times with CH_2Cl_2. The combined organic extracts were washed with H_2O, then brine, dried over Na_2SO_4, filtered, and concentrated. Purification by flash pressure column chromatography (55% EtOAc/petroleum ether) afforded indoline **150** as a colorless oil (41 mg, 0.068 mmol, 95%).

^{1}H NMR (500 MHz, C_6D_6, 348 K): δ 0.93-1.11 (4H, m), 1.32 (2H, quin, *J* = 6.9 Hz), 1.53 (3H, s), 1.86-1.95 (5H, m), 3.88 (2H, t, *J* = 6.5 Hz), 5.25 (1H, s), 5.42 (1H, dt, *J* = 11.5 Hz, 7.4 Hz), 6.04 (1H, d, *J* = 11.5 Hz), 6.76-6.88 (3H, m), 6.97-7.06 (3H, m), 7.10 (1H, d, *J* = 7.6 Hz), 7.13-7.18 (1H, obscured), 7.24 (2H, app. d, *J* = 8.2 Hz), 7.38 (1H, app. s), 7.77 (2H, app. d, *J* = 8.2 Hz), 8.32 (1H, br. s), 8.46 (1H, d, *J* = 1.9 Hz), 8.56 (1H, d, *J* = 1.6 Hz).

^{13}C NMR (125.75 MHz, C_6D_6, 348 K): δ 21.4, 23.7, 25.6, 28.8, 29.4, 29.5, 58.3, 70.5, 74.7, 117.6, 119.1, 125.8, 126.0, 126.3, 126.5, 129.4, 130.2, 130.2, 131.3, 133.2, 133.7, 134.5, 135.7, 136.4, 141.2, 144.0, 144.4, 147.2, 152.1, 168.2.‡

‡ 2 peaks obscured.

HRMS (ES$^+$): $[C_{36}H_{36}N_3O_4{}^{32}S]^+$ ($[M+H]^+$) calc. 606.2421; found 606.2414.

(2*RS*,3*RS*)-1-Acetyl-3-phenyl-2-(5-((*Z*)-7-(pyrrolidin-1-yl)hept-1-en-1-yl)pyridin-3-yl)indoline-3-carbonitrile **152**

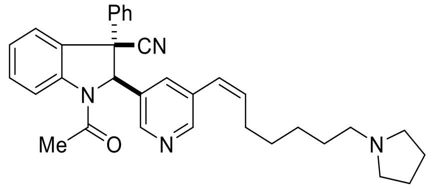

Pyrrolidine (23 µL, 0.3 mmol) was added to a solution of indoline **150** (17 mg, 0.028 mmol) in CH_3CN (2 mL). The reaction mixture was stirred at 50 °C for 1 h, then allowed to cool to rt and diluted with EtOAc. The solution was washed twice with H_2O, then with brine. The organic layer was dried over Na_2SO_4, filtered, and concentrated. Purification by flash pressure column chromatography (5% $MeOH/CH_2Cl_2$ + 0.5% aq. NH_4OH) to afford indoline **152** as a colorless oil (9.2 mg, 0.018 mmol, 64%).

^{1}H NMR (500 MHz, C_6D_6, 348 K): δ 1.22-1.33 (4H, m), 1.45 (2H, quin, *J* = 7.1 Hz), 1.52 (3H, s), 1.64 (4H, m), 2.07 (2H, app. q, *J* = 7.0 Hz), 2.38 (2H, t, *J* = 7.3 Hz), 2.43 (4H, m), 5.23 (1H, s), 5.57 (1H, dt, *J* = 11.7 Hz, 7.4 Hz), 6.07 (1H, d, *J* = 11.7 Hz), 6.81 (1H, t, *J* = 7.6 Hz), 6.94-7.06 (3H, m), 7.10 (1H, d, *J* = 7.6 Hz), 7.13-7.18 (1H, obscured), 7.25 (2H, m), 7.43 (1H, app. s), 8.36 (1H, br. s), 8.46 (1H, d, *J* = 1.9 Hz), 8.61 (1H, d, *J* = 1.5 Hz).

^{13}C NMR (125.75 MHz, C_6D_6, 348 K): δ 23.7, 24.5, 27.9, 29.3, 29.7, 30.4, 54.7, 56.9, 58.3, 74.8, 117.7, 119.1, 125.5, 125.9, 126.3, 126.4, 129.4, 129.9, 130.2, 131.3, 133.2, 133.7, 134.7, 137.0, 141.3, 144.1, 147.1, 152.2, 168.2.

HRMS (ES$^+$): $[C_{33}H_{37}ON_4]^+$ ($[M+H]^+$) requires 505.2962; found 505.2961.

(2*RS*,3*SR*)-1-Acetyl-3-phenyl-2-(5-((E)-7-(pyrrolidin-1-yl)hept-1-en-1-yl)pyridin-3-yl)-2,3-dihydro-1H-pyrrolo[3,2-b]pyridine-3-carbonitrile **153**

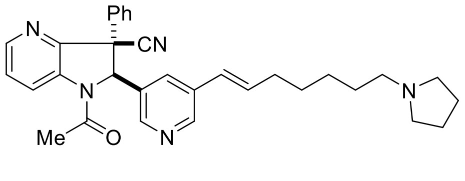

Indoline **153** was prepared according to general procedure 6 using indoline **112** (30 mg, 0.072 mmol), vinyl stannane **129** (42 mg, 0.093 mmol), and $Pd(PPh_3)_4$ (8 mg, 0.007 mmol) in NMP (1.5 mL). The reaction mixture was stirred at 85 °C for 1.5 h. Purification by flash pressure column chromatography (4.5% $MeOH/CH_2Cl_2$ + 0.5% aq. NH_4OH) afforded indoline **153** as a pale yellow oil (20 mg, 0.040 mmol, 55%).

^{1}H NMR (400 MHz, C_6D_6, 348 K): δ 1.28-1.36 (4H, m), 1.44-1.53 (5H, m), 1.64 (4H, m), 1.96 (2H, app. br. s), 2.39 (2H, t, *J* = 7.2 Hz), 2.43 (4H, m), 5.29 (1H, s), 5.98-6.08 (2H, m),ⱡ 6.70 (1H, dd, *J* = 8.2 Hz, 4.8 Hz), 6.95-7.05 (3H, m), 7.31 (2H, m), 7.35 (1H, app. s), 8.03 (1H, dd, *J* = 4.8 Hz, 1.3 Hz), 8.38 (1H, d, *J* = 2.0 Hz), 8.44 (1H, br. s), 8.66 (1H, d, *J* = 1.8 Hz).

ⱡ **^{1}H NMR (400 MHz, C_6D_6):** δ 5.98 (1H, dt, *J* = 16.0 Hz, 6.5 Hz), 5.88 (1H, d, *J* = 16.0 Hz).

^{13}C NMR (100.6 MHz, C_6D_6, 348 K): δ 23.3, 24.5, 27.9, 29.5, 29.6, 33.6, 54.7, 56.7, 59.6, 73.2, 118.0, 124.3, 124.9, 126.3, 126.7, 129.4, 130.2, 130.6, 133.6, 134.8, 135.4, 138.0, 140.2, 147.0, 147.2, 150.0, 169.0.‡

‡ 1 peak obscured.

HRMS (ES$^+$): $[C_{23}H_{36}N_5O]^+$ ($[M+H]^+$) calc. 506.2914; found 506.2914.

(2*RS*,3*RS*)-1-Acetyl-2-(5-((E)-7-(pyrrolidin-1-yl)hept-1-en-1-yl)pyridin-3-yl)-3-(thiazol-2-yl)indoline-3-carbonitrile **11**

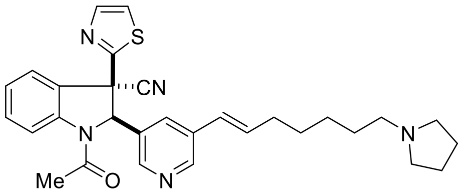

Indoline **11** was prepared according to general procedure 6 using indoline **102** (40 mg, 0.094 mmol), vinyl stannane **129** (55 mg, 0.12 mmol), and $Pd(PPh_3)_4$ (14 mg, 0.012 mmol) in NMP (1.5 mL). The reaction mixture was stirred at 85 °C for 2 h. Purification by flash pressure column chromatography (4.5% $MeOH/CH_2Cl_2$ + 0.5% aq. NH_4OH) afforded indoline **11** as a pale yellow oil (29 mg, 0.057 mmol, 60%).

^{1}H NMR (500 MHz, C_6D_6): 1.27 (4H, m), 1.38-1.55 (5H, m), 1.64 (4H, m), 1.88 (2H, app. q, *J* = 6.8 Hz), 2.32-2.44 (6H, m), 5.78 (1H, d, *J* = 16.0 Hz), 5.83 (1H, br. s), 5.88 (1H, dt, *J* = 16.0 Hz, 6.5 Hz), 6.20 (1H, d, *J* = 3.3 Hz), 6.71 (1H, td, *J* = 7.6 Hz, 0.9 Hz), 6.93 (1H, d, *J* = 7.9 Hz), 6.97 (1H, br. s), 7.07 (1H, t, *J* = 7.7 Hz), 7.33 (1H, d, *J* = 3.3 Hz), 8.09 (1H, s), 8.37 (1H, d, *J* = 2.0 Hz), 8.68 (1H, br. s).

^{13}C NMR (125.75 MHz, C_6D_6): 24.0, 24.3, 27.8, 29.6, 29.6, 33.7, 54.3, 54.7, 56.8, 72.7, 118.0, 119.8, 121.9, 125.6, 126.2, 126.7, 131.1, 131.5, 132.4, 133.5, 134.8, 143.8, 144.8, 147.3, 148.7, 163.6, 168.5.ⱡ

ⱡ 1 peak obscured.

HRMS (ES$^+$): $[C_{30}H_{34}N_5O^{32}S]^+$ ($[M+H]^+$) calc. 512.2479; found 512.2478.

III) Biological Activity Assays

3.1 AlphaScreen and Rapidfire assays

Recombinant KDM2A (1-517) containing the JmjC domain only was expressed and purified from *E. coli* [22], and used for all *in vitro* assays. AlphaScreen demethylation assays for KDM2A, KDM3A, KDM4A/C, KDM5A/B/C, KDM6B, KDM7B were carried out as described, using 2OG concentrations at approximately at K_M values of each enzymes [23,24]. KDM7A protein production and AlphaScreen assay optimisation will be described elsewhere. RapidFire IC_{50} assay for KDM2A was carried out as described in [25].

Summary of tested indolines:

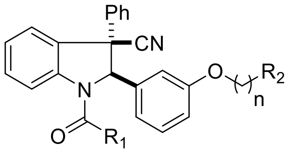

Compound	n	R_1	R_2
**56**	4	CH_3	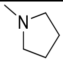
**57**	5	CH_3	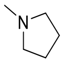
**58**	6	CH_3	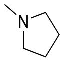
**59**	7	CH_3	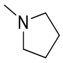
**60**	8	CH_3	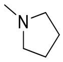
**62**	9	CH_3	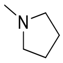
**65**	7	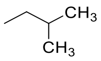	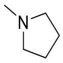
**66**	7	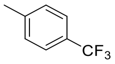	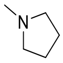
**67**	7	OEt	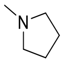
**63**	7	Ph	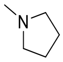
**64**	7	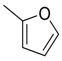	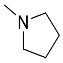
**69**	7	CH_3	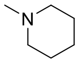

**70**	7	CH_3	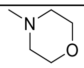
**18**	7	CH_3	NEt_2
**32**	7	CH_3	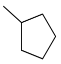

Table S-1: Summary of class 1 indolines tested.

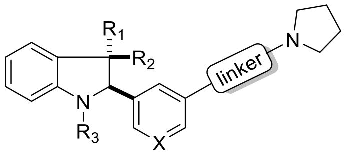

Compound	R_1	R_2	R_3	Aryl	linker
**5**	Ph	CN	$COCH_3$	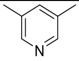	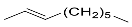
**123**	CN	Ph	$COCH_3$	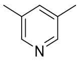	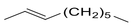
**124**	CN	CO_2Et	$COCH_3$	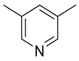	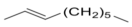
**10**	Ph	CO_2Me	$COCH_3$	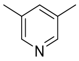	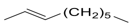
**125**	CO_2Me	Ph	$COCH_3$	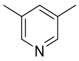	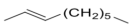
**11**	CN	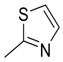	$COCH_3$	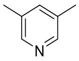	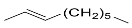
**142**	Ph	CN	H	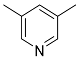	—$(CH_2)_7$—
**137**	Ph	CN	H	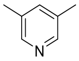	
**6**	Ph	CN	$COCH_3$	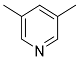	—$(CH_2)_7$—
**139**	Ph	CN	$COCH_2CN$	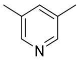	—$(CH_2)_7$—
**12**	Ph	CN	$COCH_2NH_2$	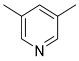	—$(CH_2)_7$—
**127**	Ph	CN	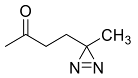	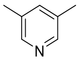	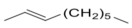
**9**	Ph	CN	$COCH_3$	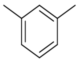	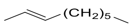
**126**	CN	Ph	$COCH_3$	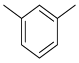	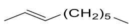
**132**	Ph	CN	$COCH_3$	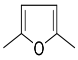	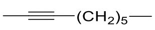
**7**	Ph	CN	$COCH_3$	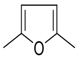	—$(CH_2)_7$—

**133**	CN	Ph	$COCH_3$	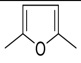	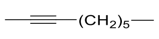
**138**	CN	Ph	$COCH_3$	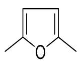	
**134**	Ph	CN	$COCH_3$	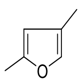	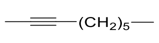
**8**	Ph	CN	$COCH_3$	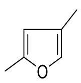	
**4**	Ph	CN	$COCH_3$	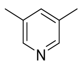	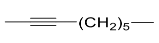
**130**	CN	Ph	$COCH_3$	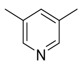	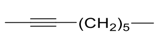
**2**	Ph	CN	$COCH_3$	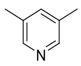	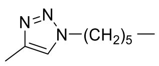
**119**	CN	Ph	$COCH_3$	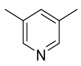	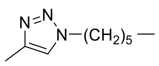
**3**	Ph	CN	$COCH_3$	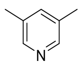	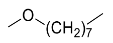
**145**	CN	Ph	$COCH_3$	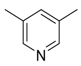	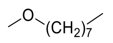
**152**	Ph	CN	$COCH_3$	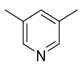	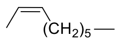
**148**	Ph	CN	$COCH_3$	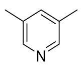	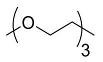
**153**[a]	Ph	CN	$COCH_3$	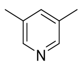	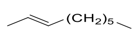
**146**[b]	Ph	CN	$COCH_3$	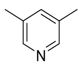	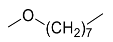

Table S-2: Summary of class 2 indolines tested. [a] 4-azaindoline scaffold. [b] NMe_2 capping group instead of pyrrolidine.

AlphaScreen Data:

pIC50 – average pIC50 value when n (biological replicate) > 1; SD – standard deviation.

KDM2A

ID	SGC ID	pIC50	SD	n
**119**	KDOOA011841a	5.753	0.249	2
**2**	KDOOA011842a	6.088	0.217	2
**123**	KDOOA011843a	6.408	0.359	4
**5**	KDOOA011844a	6.605	0.134	4

JARID1A (KDM5A)

ID	SGC ID	n	pIC50	% Inhibition at 50 µM
**4**	KDOIN000004a	1	< 4.3	39.0
**130**	KDOIN000005a	1	< 4.3	30.9
**6**	KDOIN000003a	1	< 4.3	25.5
**124**	KDOIN000010a	1	< 4.3	20.0
**(*R*,*R*)-6**	KDOIN000014a	1	< 4.3	40.0
**(*S*,*S*)-6**	KDOIN000015a	1	< 4.3	-20.3
**138**	KDOIN000033a	1	< 4.3	46.6
**132**	KDOIN000034a	1	< 4.3	49.1
**7**	KDOIN000035a	1	< 4.3	47.5
**8**	KDOIN000040a	1	< 4.3	23.0
**145**	KDOIN000041a	1	< 4.3	36.7
**3**	KDOIN000042a	1	< 4.3	24.0
**119**	KDOOA011841a	1	< 4.3	39.5
**5**	KDOOA011844a	1	< 4.3	39.1
**123**	KDOOA011843a	1	< 4.3	35.3

JARID1B (KDM5B)

ID	SGC ID	pIC50	SD	n
**(*S*,*S*)-6**	KDOIN000015a	4.882	0.555	12
**138**	KDOIN000033a	5.019	0.548	12

JARID1C (KDM5C)

ID	SGC ID	pIC50	SD	n	% Inhibition at 100 µM
**123**	KDOOA011843a	< 4.0		1	29.3
**5**	KDOOA011844a	< 4.0		1	32.3
**119**	KDOOA011841a	< 4.0		1	46.7
**2**	KDOOA011842a	4.439	0.709	2	
**(*S*,*S*)-6**	KDOIN000015a	4.803	0.207	10	
**138**	KDOIN000033a	5.006	0.185	10	

JMJD1A (KDM3A)

ID	SGC ID	pIC50	SD	n	Mean % Inhibition at 100 µM (n)
**(*S*,*S*)-6**	KDOIN000015a	< 4.0		1	35.4
**119**	KDOOA011841a	< 4.0		1	21.1
**2**	KDOOA011842a	< 4.0		1	32.5
**5**	KDOOA011844a	< 4.0		2	15.1±9.5
**123**	KDOOA011843a	< 4.0		2	18.1±12
**138**	KDOIN000033a	5.00	0.457	2	

JMJD2A (KDM4A)

ID	SGC ID	pIC50	SD	n	% Inhib. (µM)
**4**	KDOIN000004a	< 4.7		1	10.2 (18.75)
**130**	KDOIN000005a	< 4.7		1	-1.3 (18.75)
**6**	KDOIN000003a	< 4.7		1	-1.1 (18.75)
**124**	KDOIN000010a	< 4.7		1	-12.7 (18.75)
**(*R*,*R*)-6**	KDOIN000014a	< 4.7		1	7.8 (18.75)
**(*S*,*S*)-6**	KDOIN000015a	< 4.7		3	27.0±4.0
**9**	KDOIN000021a	< 4.7		1	3.4 (18.75)
**126**	KDOIN000022a	< 4.7		1	27.8 (18.75)
**133**	KDOIN000032a	< 4.7		1	19.8 (18.75)
**138**	KDOIN000033a	< 4.7		3	24.4±9.1 (10)
**132**	KDOIN000034a	< 4.7		1	18.3 (18.75)
**7**	KDOIN000035a	< 4.7		1	8.5 (18.75)
**134**	KDOIN000036a	< 4.7		1	19.4 (18.75)
**8**	KDOIN000040a	< 4.7		1	21.8 (18.75)
**145**	KDOIN000041a	< 4.7		1	-9.59 (18.75)
**3**	KDOIN000042a	< 4.7		1	-5.4 (18.75)
**148**	KDOIN000044a	< 4.7		1	-5.9 (18.75)
**5**	KDOOA011844a	< 4.7		1	18.8 (100)
**123**	KDOOA011843a	< 4.7		1	14.7 (100)
**119**	KDOOA011841a	4.140	0.350	2	
**2**	KDOOA011842a	4.413	0.220	2	

JMJD2C (KDM4C)

ID	SGC ID	pIC50	SD	n	% Inhib. (conc, µM)
**123**	KDOOA011843a	< 4.0		1	14.7 (100)
**5**	KDOOA011844a	< 4.0		1	18.8 (100)
**(*S*,*S*)-6**	KDOIN000015a	< 4.0		1	30.5 (10)
**138**	KDOIN000033a	< 4.0		1	32.3 (10)
**119**	KDOOA011841a	4.140	0.350	2	
**2**	KDOOA011842a	4.413	0.220	2	

JMJD2D (KDM4D)

ID	SGC ID	pIC50	n	% Inhibition at 10 µM
**(*S*,*S*)-6**	KDOIN000015a	< 4.0	1	22.6
**138**	KDOIN000033a	< 4.0	1	14.4

JMJD3A (KDM6B)

ID	SGC ID	pIC50	SD	n	% Inhib. (conc, μM)
**5**	KDOOA011844a	< 4.0		1	2.4
**119**	KDOOA011841a	< 4.0		1	24.6
**2**	KDOOA011842a	< 4.0		1	42.5
**123**	KDOOA011843a	< 4.0		1	3.3
**(*S*,*S*)-6**	KDOIN000015a	4.704	0.394	2	
**138**	KDOIN000033a	4.868	0.556	2	

PHF8 (KDM7B)

ID	SGC ID	pIC50	SD	n
**(*S*,*S*)-6**	KDOIN000015a	4.642	0.2956	2

KIAA1718 (KDM7A)

ID	SGC ID	pIC50	SD	n
**(*S*,*S*)-6**	KDOIN000015a	6.725	0.1400	3

RapidFire Data for inhibition of KDM2A:

ID	SGC ID	pIC50	SD	n
**148**	KDOIN000044a	3.159	1.653	4
**32**	KDOIN000017a	4.533	0.312	2
**18**	KDOIN000029a	4.771	0.089	2
**146**	KDOIN000060a	5.199	0.199	4
**70**	KDOIN000031a	5.487	0.078	2
**124**	KDOIN000010a	5.518	0.044	2
**119**	KDOOA011841a	5.533	0.062	2
**134**	KDOIN000036a	5.621	0.085	2
**125**	KDOIN000057a	5.639	0.149	2
**11**	KDOIN000061a	5.624	0.465	2
**56**	KDOIN000006a	5.695	0.176	2
**63**	KDOIN000023a	5.695	0.176	2
**130**	KDOIN000005a	5.761	0.104	4
**10**	KDOIN000056a	5.799	0.162	2
**62**	KDOIN000009a	5.822	0.234	2
**60**	KDOIN000008a	5.861	0.103	2
**65**	KDOIN000025a	5.869	0.035	2
**66**	KDOIN000026a	5.881	0.055	2
**7**	KDOIN000035a	5.917	0.084	2
**137**	KDOIN000074a	5.884	0.162	2

**57**	KDOIN000018a	5.903	0.180	2
**67**	KDOIN000027a	5.907	0.060	2
**153**	KDOIN000062a	5.881	0.383	2
**2**	KDOOA011842a	5.991	0.057	2
**69**	KDOIN000030a	5.971	0.063	2
**58**	KDOIN000019a	5.988	0.084	2
**9**	KDOIN000021a	6.016	0.066	2
**59**	KDOIN000007a	6.049	0.076	2
**134**	KDOIN000034a	5.990	0.070	2
**133**	KDOIN000032a	5.988	0.084	2
**126**	KDOIN000022a	5.983	0.114	2
**145**	KDOIN000041a	5.996	0.206	2
**64**	KDOIN000024a	6.054	0.046	2
**(*R*,*R*)-3**	KDOIN000042b	6.074	0.093	2
**152**	KDOIN000058a	6.077	0.092	2
**4**	KDOIN000004a	6.177	0.102	4
**123**	KDOOA011843a	6.340	0.192	2
**(*R*,*R*)-6**	KDOIN000014a	6.416	0.051	2
**(*S*,*S*)-3**	KDOIN000059a	6.401	0.087	2
**139**	KDOIN000073a	6.368	0.097	2
**3**	KDOIN000042a	6.537	0.122	2
**8**	KDOIN000040a	6.485	0.180	2
**138**	KDOIN000033a	6.617	0.096	2
**6**	KDOIN000003a	6.606	0.174	4
**142**	KDOIN000075a	6.708	0.067	2
**5**	KDOOA011844a	6.662	0.186	2
**(*S*,*S*)-6**	KDOIN000015a	6.799	0.041	2
**12**	KDOIN000072a	6.910	0.064	2

Indole-based inhibitors:

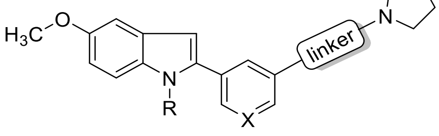

**R**	**linker**	**IC$_{50}$ (µM)**
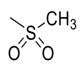	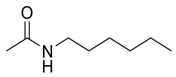	6.29
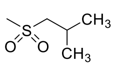	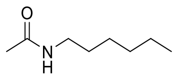	2.13

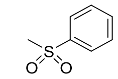	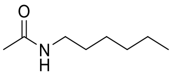	1.76
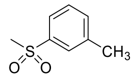	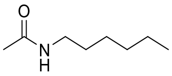	1.63
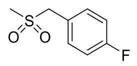	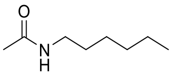	2.01
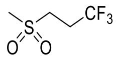	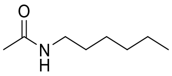	133
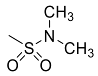	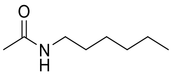	2.78
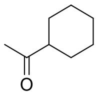	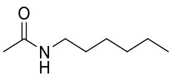	6.13
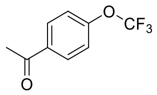	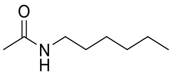	6.23
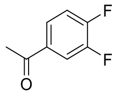	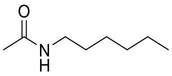	3.55
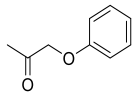	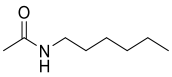	2.63
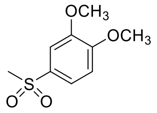	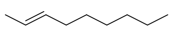	1.75
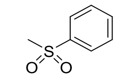	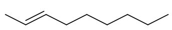	1.31

Table S-3: Summary of indoles tested. IC_{50} values correspond to KDM2A inhibition by AlphaScreen.

3.2 Selectivity-screening against other epigenetic writer and reader proteins

Methyl-Lysine Reader domain assays.

Fluorescence polarisation assays for methyl-lysine reader domains were carried out as described [26]. AlphaScreen peptide displacement assays were performed according to manufacturer's protocol (PerkinElmer, USA) with minor modifications. All reagents were diluted in 25 mM HEPES, 100 mM NaCl, 0.1 % BSA, pH 7.4 and 0.05 % CHAPS and allowed to equilibrate to room temperature prior to addition to plates. An 11-point 1:2.0

serial dilution of the ligands was prepared on low-volume 384-well plates (ProxiPlateTM-384 Plus, PerkinElmer, USA), using LabCyte Eco liquid handler. Plates filled with 5 uL of the assay buffer followed by 7 uL of biotinylated peptide ARTK(me3)QTARKSTGGKAPRK-biotin and His-tagged protein to achieve final assay concentrations of 25 nM. Plates were sealed and incubated for a further 60 minutes, before the addition of 8 µl of the mixture of streptavidin-coated donor beads (12.5 µg/ml) and nickel chelate acceptor beads (12.5 µg/ml) under low light conditions. Plates were foil-sealed to protect from light, incubated at room temperature for 60 minutes and read on a PHERAstar FS plate reader (BMG Labtech, Germany) using an AlphaScreen 680 excitation/570 emission filter set. IC_{50} values were calculated in Prism 6 (GraphPad Software, USA) after normalization against corresponding DMSO controls and are given as the final concentration of compound in the 20 µl reaction volume.

Chromodomains	WD40 domains	Tudor domains		
**CBX8**	**WDR5**	**UHRF1**	**53BP1**	**TDRD3**
> 100 µM^{+}	> 100 µM^{+}	> 100 µM^{+}	> 100 µM^{+}	> 100 µM^{+}
**CHD1**	**PHD-finger domain**	**SGF29**	**KDM4A**	**SPIN1**
>20 µM*	**KDM7B**	>20 µM*	>20 µM*	>20 µM*
	>20 µM*			

Table S-4: IC_{50} values for inhibition of methyl-lysine domains by **(*S*,*S*)-6**. Data generated using * AlphaScreen $^{+}$Fluorescence Polarisation assay.

Methyltransferase and Acetyltransferase assays.

(S,S)-6 at two different concentrations was screened for inhibition against a panel of 35 methyltransferases (MTs) and 5 histone acetyltransferases (HATs). Assays were carried out in 20mM Tris pH8.0, 5mM DTT, 0.01% Triton X-100 buffer, with S-adenosinyl L-methionine (SAM) or acetyl-coenzyme A (Acetyl-CoA) and substrate concentrations close to K_M values for each enzyme.

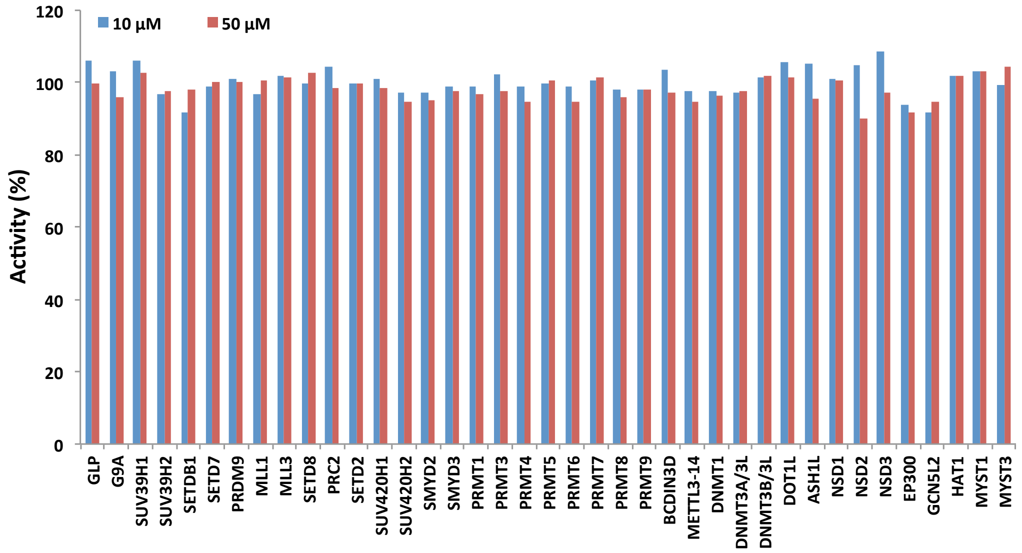

Figure S-1. Enzyme activity screening of methyltransferases and histone acetyltransferases in the presence of (S,S)-6. Percentage activity of the enzyme remaining when incubated with 10 µM or 50 µM (S,S)-6. Average (N = 2) is shown.

3.3 Cellular immunofluorescence activity assay and HeLa cytotoxicity

Cellular immunofluorescence (IF) assays were carried out as described [27]. In brief, HeLa cells were acclimatised to lower serum concentration (0.5% foetal calf serum (FCS)) for at least a week. Acclimatised cells were transiently transfected with a plasmid encoding either a FLAG-tagged wild-type (WT) or catalytically inactive mutant (H212A/D214A, MUT) KDM2A using Lipofectamine 2000 (Life Technologies). Four hours after transfection the cells were treated with serial dilutions of compound for 24 hours in OPTIMEM supplemented with 0.5% FCS. The cells were then fixed and stained with an anti-FLAG antibody (Sigma F7425) and an anti-H3K36me2 antibody (Active Motif 61019) overnight at four degrees. The secondary antibodies were Alexa 488 anti-mouse and Alexa 568 anti-rabbit (Life Technologies). After staining the nuclei with DAPI, the cells were imaged on the Operetta High Content Imaging System (PerkinElmer) and image analysis was performed with the Columbus software (PerkinElmer). H3K36me2 levels were only analysed in cells highly expressing the demethylase. HeLa cellular toxicity was determined by counting the total number of DAPI-stained nuclei imaged in 12 fields (x20 objective) during the KDM IF assay shown (representative of 3 experiments).

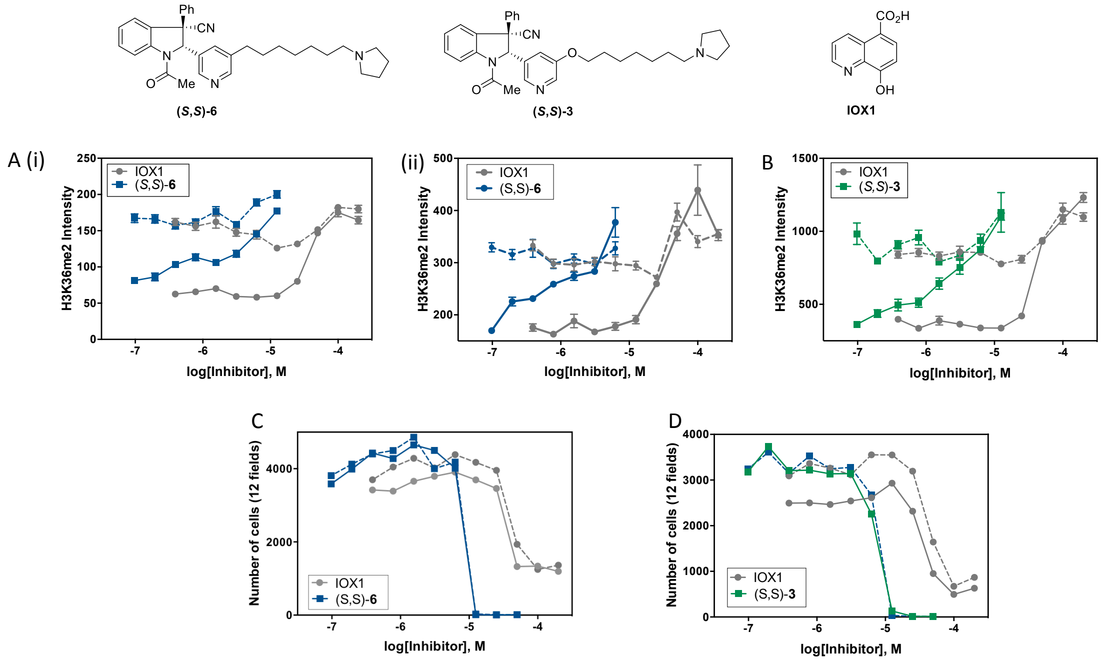

Figure S-2. Cellular effects of inhibitor dosing on HeLa cells overexpressing KDM2A. (A,B) Immunofluorescence assay measuring the global nuclear H3K36me2 staining levels (average ± SEM (N >100 transfected cells)) of HeLa cells overexpressing FLAG-tagged wild-type or catalytically mutant KDM2A (full-length). IOX1 is used as a positive control [23,26]. Datasets for N <100 cells are omitted. Examples of two independent experiments are shown for (S,S)-6 (A i, ii) with (i) data presented in Figure 2A. **(C,D)** Number of cells in 12 fields (all cells) based on DAPI staining. corresponding to the IF data (A.ii and C, B and D). EC_{50} for cell viability calculated using DAPI staining are: (S,S)-6 (7.6 µM), (S,S)-3 (7.2 µM) and IOX1 (appx. 50 µM); Solid lines – wild type KDM2A transfected wells; Dashed lines – catalytic mutant KDM2A transfected wells.

3.4 Additional viability assays

Viability assays in HAP1 cells

HAP1 cells (4000 cells / well) were treated with compounds or DMSO for 48h in triplicates. Cell viability was measured by luminescent ATP read-out (CellTiterGlo, Promega) and normalised to the DMSO control (plotted as lowest concentration point to allow log-calculation). Data analysis including EC_{50} calculations was performed in GraphPad Prism. Dose response curves are representative of multiple experiments (2-3 biological replicates each).

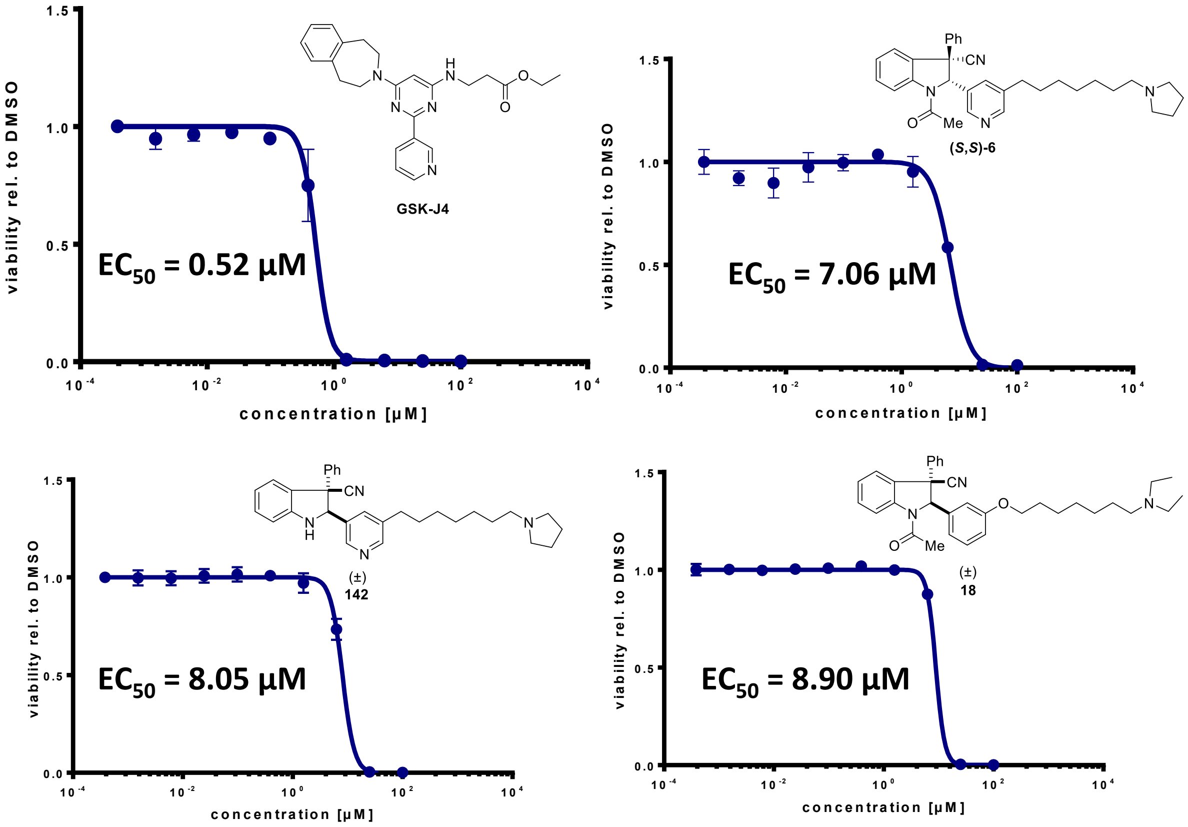

Figure S-3. KDM Inhibitor Toxicity on HAP1 Cells. HAP1 wild-type cells were treated with the indicated concentrations of various KDM inhibitors for 2 days. Cell viability was measured by luminescent ATP read-out and normalised to a DMSO control. The graphs show the mean of triplicate measurements and are representative of 2-3 independent experiments. Error bars indicate SEM.

Cell proliferation assay

Human fibroblasts (HDFa) from ThermoFisher were cultured in Medium 106 supplemented with Low Serum Growth Supplement Kit (ThermoFisher). HDFa cells were trypsinized and plated 24 hrs before addition of compound in a standard 96-well tissue culture plate so that they were subconfluent a day later. The next day, the medium was replaced with fresh medium containing compound at various dilutions starting at 100uM with 1:2 serial dilutions. The solvent concentration was maintained at 0.2% DMSO for each dilution and each

compound was tested in triplicate. After 24hrs, the medium in each well was replaced with fresh medium containing 10% WST-1 (Roche) and incubated for 1 hr. The plate was read on a Spectramax spectrophotometer (Molecular Devices) and blanked against wells containing medium only. The dose response curves were then plotted in GraphPad Prism 6.0.

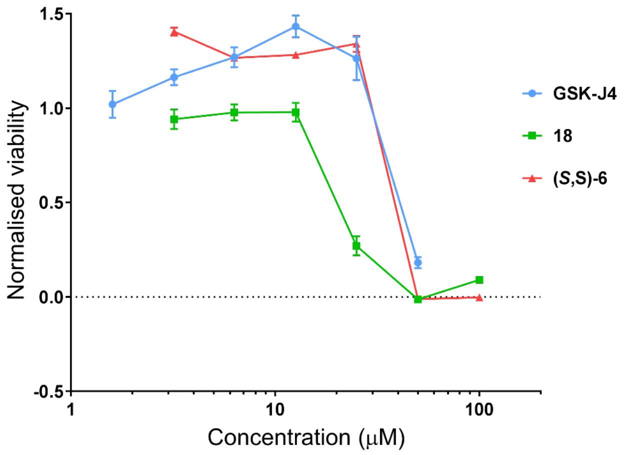

Figure S-4. KDM inhibitor toxicity on Human Dermal Fibroblasts using WST1 assay.

IV) Transcriptomics

RNA sequencing protocol:

HAP1 cells were plated in 12-well plates. KDM inhibitors were added the next day (2 concentrations each, HI = 10 μM, LO = 1 μM; duplicates for each condition) and cells were treated for 24 h. Afterwards, cells were washed twice with cold PBS and frozen at -80°C. Total RNA was isolated using the RNeasy Mini kit (Qiagen) and 500ng were used as input for library preparation with the QuantSeq 3' mRNA-Seq Library Prep Kit (Lexogen) according to manufacturer's instructions (13 instead of 12 PCR cycles were used for library amplification). cDNA concentrations were measured using Qubit dsDNA HS assay on a Qubit 2.0 Fluorometric Quantitation System (Life Technologies). Amplified libraries (7 ng) were pooled (48 samples per sequencing lane) and analysed for size distribution on an Agilent Tapestation 2200 D1000. Libraries were quantified using a Library Quant Kit (Kapa Biosystems) and sequenced on a HiSeq 2500 (Illumina) machine (50bp single-end) following the manufacturer's guidelines. Illumina's sequencing primer was exchanged with Lexogen's CSP primer in order to pinpoint the 3' end of each mRNA.

Raw sequencing data was cleaned to discard low-quality reads and then aligned onto the human reference genome using GSNAP [28]. Gene expression was evaluated on GENCODE genes using Exp3p (github.com/tkonopka/Exp3p). Subsequent gene expression analysis was performed using package ExpCube (github.com/tkonopka/ExpCube) following a previously described procedure [29] and default package settings. In brief, variability in gene expression among DMSO-treated samples was used to empirically adjust gene expression uncertainty intervals provided by Exp3p. Each gene in each sample was then scored for differential expression against the DMSO-treated samples. A score of +1 was assigned if the fold change was above 1.75 and a z-score was above 1.75. Lower scores were given for weaker signals, and similar thresholds were applied for down-regulated genes. Scores among replicates were averaged to produce group-wise scores, and genes were finally called as differentially expressed if the group score was above 0.8. Raw sequencing data will become available at the European Nucleotide Archive.

Table of gene expression changes:

Gene	Compound (conc.*)	Fold change
MT1E	GSKJ1J4_HI	2.0907
MT1F	GSKJ1J4_HI	1.9188
MT1X	GSKJ1J4_HI	2.4833
MT2A	GSKJ1J4_HI	3.2407
RAB40C	GSKJ1J4_HI	2.6167
SLC16A3	GSKJ1J4_HI	2.6995
TMEM63B	GSKJ1J4_HI	2.2001
ZNF550	GSKJ1J4_LO	0.5206
MT1E	GSKJ1J4_LO	2.2194
MT1F	GSKJ1J4_LO	1.9364
MT2A	GSKJ1J4_LO	3.2511
ALDH1B1	18_HI	0.3503
ALDH5A1	18_HI	0.3545
ANXA1	18_HI	0.2586
AP1B1	18_HI	0.5191
ARHGAP18	18_HI	0.4115
ATP5G1	18_HI	0.4941
ATP5G3	18_HI	0.4962
BAG2	18_HI	0.4839
BOP1	18_HI	0.4516
C12orf43	18_HI	0.4081
C16orf59	18_HI	0.4446
CAMKV	18_HI	0.3676
CCDC71	18_HI	0.3555

CCDC86	18_HI	0.4756
CD3EAP	18_HI	0.4155
CDKN3	18_HI	0.4345
CHCHD10	18_HI	0.457
CHCHD7	18_HI	0.409
CIRH1A	18_HI	0.4887
CNN2	18_HI	0.5043
COQ3	18_HI	0.4803
DDX23	18_HI	0.5221
DPH2	18_HI	0.4277
ELAC2	18_HI	0.4768
EMG1	18_HI	0.426
ERCC6L	18_HI	0.4259
FABP5	18_HI	0.4899
FARSB	18_HI	0.5197
FASTKD1	18_HI	0.4552
GAS2L1	18_HI	0.4253
GDAP1	18_HI	0.3718
GEMIN6	18_HI	0.4869
GFM1	18_HI	0.3642
GNG2	18_HI	0.3686
IPO4	18_HI	0.4243
IVNS1ABP	18_HI	0.4996
JMJD4	18_HI	0.3511
KM-PA-2	18_HI	0.449
LGSN	18_HI	0.1996
MCM4	18_HI	0.4963
MIPEP	18_HI	0.3272
MRPL12	18_HI	0.3879
MRPS33	18_HI	0.5073
MT-ND6	18_HI	0.3952
MTRNR2L1	18_HI	0.3885
MTRNR2L2	18_HI	0.361
MT-TC	18_HI	0.3485
MT-TM	18_HI	0.1557
MT-TN	18_HI	0.3898
MT-TS2	18_HI	0.3194
MYBBP1A	18_HI	0.5322
MYCBP	18_HI	0.29
NAV3	18_HI	0.33
NDUFAF3	18_HI	0.425
NLE1	18_HI	0.4413
NOP9	18_HI	0.4534
NRGN	18_HI	0.3732
PAK1IP1	18_HI	0.4861

PDK3	18_HI	0.4215
PIGW	18_HI	0.4651
PKMYT1	18_HI	0.5094
POLA2	18_HI	0.3758
POLR2L	18_HI	0.5005
PRMT1	18_HI	0.4966
PSMD3	18_HI	0.4887
PTGES2	18_HI	0.4368
RFC3	18_HI	0.4401
RP11-145M9.4	18_HI	0.4377
RP5-886K2.3	18_HI	0.3184
RTN4IP1	18_HI	0.3488
RUVBL1	18_HI	0.5094
SCFD2	18_HI	0.4413
SDF2L1	18_HI	0.4852
SEMA3A	18_HI	0.3525
SEPW1	18_HI	0.4863
SLC43A2	18_HI	0.4959
SRM	18_HI	0.4571
SUV39H1	18_HI	0.4456
SVIP	18_HI	0.4669
TIMM8A	18_HI	0.2646
TIPIN	18_HI	0.3974
TMEM177	18_HI	0.388
TPD52L1	18_HI	0.3055
TUBA1B	18_HI	0.4485
TUBB	18_HI	0.487
TUBB4B	18_HI	0.4041
TUBG1	18_HI	0.4803
UCP2	18_HI	0.3485
UHRF1	18_HI	0.4563
UMPS	18_HI	0.471
UNG	18_HI	0.4104
VPS9D1-AS1	18_HI	0.3836
WDR4	18_HI	0.4567
WDR46	18_HI	0.4258
XRCC5	18_HI	0.5372
ZNF17	18_HI	0.2465
ZWINT	18_HI	0.4735
ABL1	18_HI	5.66
AC159540.1	18_HI	3.7418
ACAT2	18_HI	2.4699
ACSS2	18_HI	3.6116
ADM2	18_HI	4.19
ADRA2C	18_HI	3.1276

AGO3	18_HI	2.2012
AKNA	18_HI	4.1566
ALDOC	18_HI	2.9672
ARID5B	18_HI	2.4555
ASNS	18_HI	2.431
ASS1	18_HI	7.1909
ATF3	18_HI	5.1861
ATXN2L	18_HI	3.2466
BHLHE22	18_HI	2.7722
CACNA2D2	18_HI	3.0487
CAMK2N1	18_HI	2.3366
CCL18	18_HI	4.793
CEBPB	18_HI	2.8466
CEBPG	18_HI	2.2092
CHAC1	18_HI	6.0447
CLCN6	18_HI	2.4903
CNR1	18_HI	13.9931
COLGALT2	18_HI	2.6122
CREB3L1	18_HI	4.237
CTC-444N24.11	18_HI	2.7375
CTH	18_HI	2.5996
CYP51A1	18_HI	3.0066
DAAM1	18_HI	2.3059
DDIT3	18_HI	3.919
DDIT4	18_HI	6.867
DDX26B	18_HI	2.3394
DHCR7	18_HI	2.2907
DLK1	18_HI	3.6025
DLL1	18_HI	3.9184
DST	18_HI	2.6356
EGR1	18_HI	5.7539
FA2H	18_HI	3.1
FADS1	18_HI	2.353
FAM129A	18_HI	4.8189
FDPS	18_HI	2.4722
FNIP2	18_HI	2.559
FTL	18_HI	2.0875
FYN	18_HI	3.2712
FZD7	18_HI	2.8274
GADD45A	18_HI	2.5823
GADD45G	18_HI	2.2124
GDF15	18_HI	13.232
GOLGB1	18_HI	2.6135
GPM6B	18_HI	3.2646
HERPUD1	18_HI	2.9467

HMGCR	18_HI	2.7638
HMGCS1	18_HI	5.1171
HRK	18_HI	5.1215
HSD17B7	18_HI	2.5229
IDI1	18_HI	2.7214
IGF2R	18_HI	3.4711
INHBE	18_HI	8.2963
INSIG1	18_HI	5.6273
INSM1	18_HI	3.4284
JUN	18_HI	8.7752
JUND	18_HI	3.1845
KLHL24	18_HI	3.5878
LIMCH1	18_HI	2.4689
LINC00086	18_HI	2.7542
LIPG	18_HI	4.5117
LRP8	18_HI	2.3141
LRRD1	18_HI	2.9128
LRRN1	18_HI	2.7177
LSS	18_HI	2.6824
LUZP2	18_HI	3.7098
MAF	18_HI	4.7708
MALAT1	18_HI	3.4188
MAP1B	18_HI	2.3707
MMAB	18_HI	2.2522
MSMO1	18_HI	3.1185
MVD	18_HI	4.3176
MVK	18_HI	2.3755
NEU1	18_HI	3.1338
NEUROG2	18_HI	10.6425
NPTXR	18_HI	2.427
NUPR1	18_HI	8.8782
OPTN	18_HI	3.2986
PARD3B	18_HI	4.2091
PGPEP1	18_HI	2.3484
PLCB1	18_HI	2.6651
PLXNA2	18_HI	3.9174
PLXNC1	18_HI	2.1336
PMAIP1	18_HI	2.0498
PNPLA3	18_HI	4.0521
PNRC1	18_HI	2.5567
PPP1R15A	18_HI	4.5274
RENBP	18_HI	9.3875
RFX4	18_HI	3.3044
RN7SK	18_HI	6.5757
RN7SL1	18_HI	3.707

RN7SL2	18_HI	4.5714
RN7SL5P	18_HI	4.2667
RP11-265D19.6	18_HI	3.802
RP11-390P2.4	18_HI	5.9028
RP11-54O7.1	18_HI	3.908
RP11-54O7.2	18_HI	3.5638
RP11-73M18.9	18_HI	3.4741
RP5-952N6.1	18_HI	4.076
RPS6KA2	18_HI	2.2401
SARS	18_HI	1.9547
SC5D	18_HI	1.9931
SERPINF1	18_HI	4.21
SESN2	18_HI	2.4825
SHC2	18_HI	3.609
SLC1A4	18_HI	2.3455
SLC38A7	18_HI	2.6138
SLC3A2	18_HI	2.055
SLC6A9	18_HI	3.1666
SLC7A11	18_HI	3.2405
SLFN5	18_HI	4.4807
SLIT2	18_HI	3.385
SOX21	18_HI	2.3046
SQLE	18_HI	2.2821
SRGAP1	18_HI	4.2997
STARD4	18_HI	3.3651
STAT2	18_HI	2.5389
STC2	18_HI	2.9119
STX3	18_HI	2.2503
TBC1D9	18_HI	2.4082
THSD4	18_HI	2.5675
TMEM2	18_HI	2.2466
TMSB4X	18_HI	2.8999
TNRC6B	18_HI	2.3131
TRIB3	18_HI	2.6484
TSC22D3	18_HI	4.411
TTC28	18_HI	2.1222
UAP1L1	18_HI	2.2236
VCAM1	18_HI	4.729
VEGFA	18_HI	4.0584
VLDLR	18_HI	3.0539
WIPI1	18_HI	5.7027
ZBED3	18_HI	2.1499
ZSCAN18	18_HI	2.82
ADAMTS1	18_LO	0.3645
ALDH1B1	(S,S)-6_HI	0.3893

ATP10D	(S,S)-6_HI	0.4706
C17orf104	(S,S)-6_HI	0.3713
CCDC86	(S,S)-6_HI	0.4357
CD3EAP	(S,S)-6_HI	0.3461
CHCHD10	(S,S)-6_HI	0.4677
CHCHD7	(S,S)-6_HI	0.4271
CHN1	(S,S)-6_HI	0.4565
CNN2	(S,S)-6_HI	0.4226
DCLK1	(S,S)-6_HI	0.472
ENOSF1	(S,S)-6_HI	0.4464
ERCC6L	(S,S)-6_HI	0.4293
FABP5	(S,S)-6_HI	0.4735
FANCF	(S,S)-6_HI	0.3613
FGF13	(S,S)-6_HI	0.4284
FKBP4	(S,S)-6_HI	0.4631
GALNT12	(S,S)-6_HI	0.2182
GCSH	(S,S)-6_HI	0.5103
GINS2	(S,S)-6_HI	0.4722
GPD2	(S,S)-6_HI	0.4302
GTF3C6	(S,S)-6_HI	0.5225
HIST1H1D	(S,S)-6_HI	0.231
IVNS1ABP	(S,S)-6_HI	0.4897
KCND2	(S,S)-6_HI	0.2938
KISS1R	(S,S)-6_HI	0.3999
LIN28B	(S,S)-6_HI	0.4238
MCCC2	(S,S)-6_HI	0.3173
MFI2-AS1	(S,S)-6_HI	0.2878
MME	(S,S)-6_HI	0.4238
MT1E	(S,S)-6_HI	0.4267
MT1F	(S,S)-6_HI	0.4161
MYBBP1A	(S,S)-6_HI	0.4852
MYCBP	(S,S)-6_HI	0.3961
NAV3	(S,S)-6_HI	0.2758
POLR3H	(S,S)-6_HI	0.475
RP11-31F19.1	(S,S)-6_HI	0.3148
RPP25	(S,S)-6_HI	0.3562
SCFD2	(S,S)-6_HI	0.2721
SEMA3A	(S,S)-6_HI	0.1927
SLC2A4RG	(S,S)-6_HI	0.4778
TAF9B	(S,S)-6_HI	0.4942
TIMM8A	(S,S)-6_HI	0.3904
TMEM261	(S,S)-6_HI	0.4919
TNS3	(S,S)-6_HI	0.4216
TXNDC17	(S,S)-6_HI	0.4379
VPS9D1-AS1	(S,S)-6_HI	0.3669

ACAT2	(S,S)-6_HI	4.6371
ACSL4	(S,S)-6_HI	1.9564
ACSS2	(S,S)-6_HI	3.6303
ADM	(S,S)-6_HI	2.1079
ALDOC	(S,S)-6_HI	5.5455
AMDHD2	(S,S)-6_HI	2.4027
ASNS	(S,S)-6_HI	2.0167
ATF3	(S,S)-6_HI	6.4519
ATP6V0A1	(S,S)-6_HI	2.9255
BBC3	(S,S)-6_HI	1.9576
BHLHE40	(S,S)-6_HI	3.7678
BICD1	(S,S)-6_HI	2.1192
BLOC1S2	(S,S)-6_HI	2.117
BLVRB	(S,S)-6_HI	2.7103
BNIP3P1	(S,S)-6_HI	1.9652
BRF2	(S,S)-6_HI	2.3323
C10orf35	(S,S)-6_HI	2.2849
C14orf1	(S,S)-6_HI	2.5026
C6orf1	(S,S)-6_HI	2.2739
CACNA2D2	(S,S)-6_HI	3.1812
CCL18	(S,S)-6_HI	7.2115
CD70	(S,S)-6_HI	2.1
CDKN1A	(S,S)-6_HI	4.6469
CEBPB	(S,S)-6_HI	2.356
CHAC1	(S,S)-6_HI	3.963
CHPF2	(S,S)-6_HI	2.4365
CLCN6	(S,S)-6_HI	2.9713
CLCN7	(S,S)-6_HI	2.0449
CLU	(S,S)-6_HI	2.3983
CRYAA	(S,S)-6_HI	8.3321
CTH	(S,S)-6_HI	2.1147
CTSD	(S,S)-6_HI	3.1788
CYLD	(S,S)-6_HI	2.3486
CYP51A1	(S,S)-6_HI	3.3282
DDIT3	(S,S)-6_HI	3.8741
DDIT4	(S,S)-6_HI	7.5524
DHCR24	(S,S)-6_HI	4.4157
DHCR7	(S,S)-6_HI	3.262
DYNC1H1	(S,S)-6_HI	2.2429
EBP	(S,S)-6_HI	2.1981
EIF2AK3	(S,S)-6_HI	3.1385
ELOVL6	(S,S)-6_HI	2.7144
ENO2	(S,S)-6_HI	2.4436
FA2H	(S,S)-6_HI	3.4973
FADS1	(S,S)-6_HI	2.3944

FADS2	(S,S)-6_HI	2.0969
FAM129A	(S,S)-6_HI	3.4036
FAM219A	(S,S)-6_HI	2.6558
FASN	(S,S)-6_HI	2.2437
FDFT1	(S,S)-6_HI	2.5978
FDPS	(S,S)-6_HI	3.8535
FLCN	(S,S)-6_HI	2.5249
FNIP1	(S,S)-6_HI	1.9599
FNIP2	(S,S)-6_HI	2.2589
FOS	(S,S)-6_HI	6.0247
FTL	(S,S)-6_HI	2.6565
GABARAPL1	(S,S)-6_HI	2.9028
GADD45A	(S,S)-6_HI	2.7988
GOLGA2	(S,S)-6_HI	2.2351
GSTP1	(S,S)-6_HI	2.4334
HERPUD1	(S,S)-6_HI	2.0488
HMGCR	(S,S)-6_HI	3.8679
HMGCS1	(S,S)-6_HI	8.2428
HMOX1	(S,S)-6_HI	6.8056
HRK	(S,S)-6_HI	4.6729
HSD17B7	(S,S)-6_HI	3.8005
HSD17B7P2	(S,S)-6_HI	3.7994
HSPA1A	(S,S)-6_HI	2.4003
HSPA1B	(S,S)-6_HI	2.6978
IDI1	(S,S)-6_HI	2.9232
IGF2R	(S,S)-6_HI	3.1292
INSIG1	(S,S)-6_HI	7.1722
JDP2	(S,S)-6_HI	2.4164
JUN	(S,S)-6_HI	14.5392
JUND	(S,S)-6_HI	3.0464
KDM6B	(S,S)-6_HI	2.6529
KLF11	(S,S)-6_HI	3.3754
KLF2	(S,S)-6_HI	2.8813
KLF6	(S,S)-6_HI	2.159
KLHL21	(S,S)-6_HI	2.5235
KLHL24	(S,S)-6_HI	2.3626
LBH	(S,S)-6_HI	2.3929
LINC00086	(S,S)-6_HI	2.1979
LINC00685	(S,S)-6_HI	2.0497
LINC00963	(S,S)-6_HI	3.6953
LIPG	(S,S)-6_HI	5.096
LPIN1	(S,S)-6_HI	2.8343
LRP8	(S,S)-6_HI	2.7475
LRRD1	(S,S)-6_HI	3.2604
LSS	(S,S)-6_HI	3.928

MAF	(S,S)-6_HI	5.5669
MAFG	(S,S)-6_HI	2.7324
MALAT1	(S,S)-6_HI	3.3044
MAP1A	(S,S)-6_HI	5.9987
MAP1B	(S,S)-6_HI	2.3865
MLLT11	(S,S)-6_HI	2.3172
MMAB	(S,S)-6_HI	3.0765
MSMO1	(S,S)-6_HI	3.8137
MVD	(S,S)-6_HI	8.8991
MVK	(S,S)-6_HI	3.6993
MXD1	(S,S)-6_HI	1.9931
NDRG4	(S,S)-6_HI	3.4375
NEU1	(S,S)-6_HI	3.9699
NEUROG2	(S,S)-6_HI	5.5463
NFKBIA	(S,S)-6_HI	2.0794
NPC2	(S,S)-6_HI	2.3008
NPDC1	(S,S)-6_HI	2.1615
NSDHL	(S,S)-6_HI	3.1224
NXPE3	(S,S)-6_HI	2.1096
OPTN	(S,S)-6_HI	3.8478
OSGIN1	(S,S)-6_HI	6.112
PANX2	(S,S)-6_HI	3.5853
PHF1	(S,S)-6_HI	3.081
PLA2G16	(S,S)-6_HI	2.5933
PNPLA3	(S,S)-6_HI	4.754
POMT2	(S,S)-6_HI	2.1067
PPP1R15A	(S,S)-6_HI	6.212
PPP2R5B	(S,S)-6_HI	2.6898
PROX1	(S,S)-6_HI	2.0861
PSAP	(S,S)-6_HI	2.2535
QPRT	(S,S)-6_HI	2.3306
RAB15	(S,S)-6_HI	3.0855
RELB	(S,S)-6_HI	17.5232
RENBP	(S,S)-6_HI	5.22
RND1	(S,S)-6_HI	3.6006
RP11-265D19.6	(S,S)-6_HI	4.6137
RP11-284F21.10	(S,S)-6_HI	4.9095
RP11-390P2.4	(S,S)-6_HI	3.6155
RP11-395B7.7	(S,S)-6_HI	2.9018
RP11-443P15.2	(S,S)-6_HI	5.2555
RP5-1024G6.8	(S,S)-6_HI	2.9508
RP5-850E9.3	(S,S)-6_HI	2.167
RRAGC	(S,S)-6_HI	2.0283
S100A6	(S,S)-6_HI	2.0705
SC5D	(S,S)-6_HI	2.7321

SCARNA22	(S,S)-6_HI	2.7001
SCD	(S,S)-6_HI	2.9975
SDCBP	(S,S)-6_HI	2.1132
SEL1L3	(S,S)-6_HI	2.4861
SERPINF1	(S,S)-6_HI	2.056
SESN2	(S,S)-6_HI	2.5016
SIPA1L2	(S,S)-6_HI	2.1281
SLC16A3	(S,S)-6_HI	5.5172
SLC25A16	(S,S)-6_HI	2.5041
SLC2A3	(S,S)-6_HI	3.1954
SLC2A6	(S,S)-6_HI	2.6181
SLC36A1	(S,S)-6_HI	2.2807
SLC38A2	(S,S)-6_HI	2.0693
SLC38A6	(S,S)-6_HI	2.2647
SLC7A11	(S,S)-6_HI	4.1788
SNAI3-AS1	(S,S)-6_HI	6.8746
SQLE	(S,S)-6_HI	2.6462
SQSTM1	(S,S)-6_HI	5.3332
SRXN1	(S,S)-6_HI	2.2349
STARD4	(S,S)-6_HI	4.0455
STAT2	(S,S)-6_HI	2.5237
STX3	(S,S)-6_HI	3.1633
SYNE2	(S,S)-6_HI	2.352
SYT2	(S,S)-6_HI	5.2602
TBC1D9	(S,S)-6_HI	1.9919
TES	(S,S)-6_HI	2.8091
TMEM2	(S,S)-6_HI	2.3277
TMEM55B	(S,S)-6_HI	2.8658
TMEM63B	(S,S)-6_HI	2.1617
TP53INP2	(S,S)-6_HI	2.0959
TRIB3	(S,S)-6_HI	2.6126
TSC22D3	(S,S)-6_HI	4.1307
TUBA1A	(S,S)-6_HI	2.5922
UAP1L1	(S,S)-6_HI	2.2814
UBC	(S,S)-6_HI	2.3261
VEGFA	(S,S)-6_HI	3.0189
VWA5A	(S,S)-6_HI	4.0982
WNT9A	(S,S)-6_HI	2.6243
YPEL5	(S,S)-6_HI	1.9396
FAM195A	(S,S)-6_LO	0.4053
FUK	(S,S)-6_LO	0.3545
ACAT2	(S,S)-6_LO	3.7492
ARHGEF9	(S,S)-6_LO	2.0189
C14orf1	(S,S)-6_LO	1.9715
CYP51A1	(S,S)-6_LO	2.4049

DHCR24	(S,S)-6_LO	2.0807
DHCR7	(S,S)-6_LO	2.1388
EBP	(S,S)-6_LO	1.8331
ELOVL6	(S,S)-6_LO	2.0769
FADS1	(S,S)-6_LO	1.8837
FADS2	(S,S)-6_LO	1.8222
FDFT1	(S,S)-6_LO	2.4838
FDPS	(S,S)-6_LO	2.5827
HMGCR	(S,S)-6_LO	2.5977
HMGCS1	(S,S)-6_LO	5.0219
HSD17B7	(S,S)-6_LO	3.7338
HSD17B7P2	(S,S)-6_LO	3.495
IDI1	(S,S)-6_LO	2.6648
INSIG1	(S,S)-6_LO	3.7241
LRRD1	(S,S)-6_LO	2.3418
LSS	(S,S)-6_LO	3.0803
MMAB	(S,S)-6_LO	2.1291
MSMO1	(S,S)-6_LO	3.3877
MVD	(S,S)-6_LO	3.0871
MVK	(S,S)-6_LO	2.2178
NEUROG2	(S,S)-6_LO	2.8444
NSDHL	(S,S)-6_LO	2.2227
PNPLA3	(S,S)-6_LO	2.3546
SC5D	(S,S)-6_LO	2.1587
SCD	(S,S)-6_LO	2.2016
SQLE	(S,S)-6_LO	2.0534
STARD4	(S,S)-6_LO	2.9088
TUBA1A	(S,S)-6_LO	2.064

Table S-5: Gene expression changes in HAP1 cells upon incubation with **18** and **(*S,S*)-6** at 10 µM (HI) and 1 µM (LO). Differentially expressed genes ("gene signatures") compared to the DMSO control were determined.

V) Non-denaturing Mass-Spectrometry

A sample of KDM2A was buffer-exchanged into 200 mM ammonium acetate and diluted to a concentration of 2.5 µM for analysis. (S,S)-**6** was dissolved in DMSO to a concentration of 10 mM, further diluted with 200 mM ammonium acetate and mixed at a 1:1 v/v ratio with KDM2A to give final concentrations of 2.5 µM and 12.5 µM for KDM2A and (S,S)-**6** respectively. The sample was then equilibrated at room temperature for 30 minutes before MS analysis. Data were acquired on a quadrupole time-of-flight (TOF) mass spectrometer (Synapt G1 HDMS, Waters Corp., Wilmslow, UK) using nanoelectrospray ionisation, and with instrument settings optimised for the transmission of intact noncovalent protein complexes.[18] Experiments were

conducted in positive polarity and TOF mode, with the following instrument settings: capillary voltage 1.4 kV, sample cone 10 V, "trap" collision voltage 10 V (40 V for tandem MS dissociation spectrum), "transfer" collision voltage 5 V, source pressure 4.0 mbar, and the collision cell pressures 10 µbar.

VI) Mode of Action Studies

2-OG Competition

Solutions containing H3K36me2 peptide (20 µM), $Fe^{II}(NH_4)SO_4$ (20 µM), sodium-L-ascorbate (200 µM), and 2-OG (0.4 µM, 0.8 µM, 1.2 µM, 2.0 µM, 4.0 µM, 6.0 µM) in MES buffer (pH 7.0) were prepared in a deep 96-well plate (total volume per well = 600 µL). A solution of KDM2A in MES buffer (50 mM MES, 100 mM NaCl [pH 7.0]) was prepared (total volume = 12 mL) and divided equally into 3 falcon tubes, to which was added **(*S*,*S*)-6** (tube A (solution A): 0 nM, tube B (solution B): 500 nM, tube C (solution C): 1000 nM). After incubation at 0 °C for 45 min, 600 µL of solution A was added to each well, and the progress of demethylation was monitored *via* automated sampling using the RapidFire mass spectrometry platform (Agilent Technologies, Wakefield MA). The relative concentrations of H3K36me0 (where observed) and H3K36me1 were determined by integration of the extracted ion chromatogram corresponding to the H3K36me0/1 peptides using MassHunter software (Agilent Technologies). This procedure was repeated with solutions B and C using freshly-prepared 2-OG wells. Initial rates were determined by calculating the slope of the linear region of the reaction. Kinetic parameters are apparent values due to non-saturating conditions used.

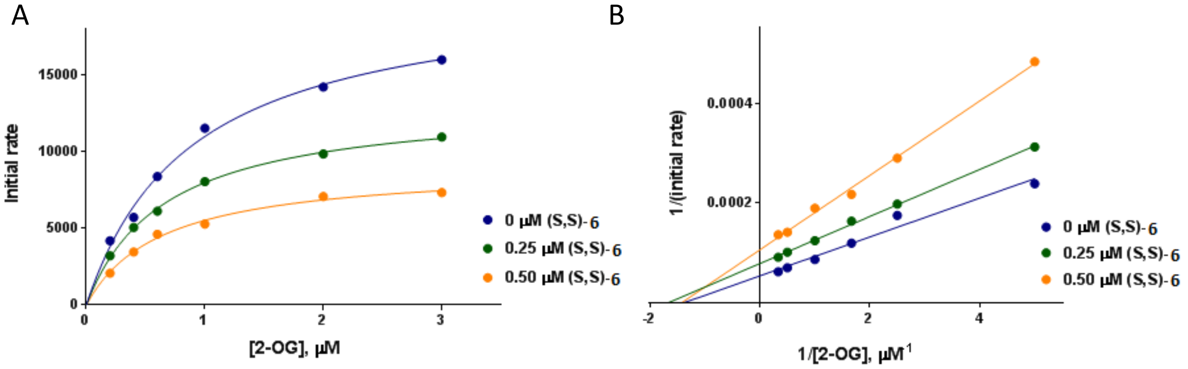

Figure S-5 – A) Michaelis-Menten plot of [2-OG] vs. initial rate of peptide demethylation. The concentration of the peptide substrate is 10 µM. Mixed-mode inhibition fit gives K_M^{app} (2OG) = 0.88 ± 0.08 µM, Ki^{app} = 0.9 ± 0.3 µM; α = 0.45 ± 0.21, R^2 = 0.9908 (B) Lineweaver-Burke plot

Peptide Competition

Solutions containing 2-OG (20 µM), $Fe^{II}(NH_4)SO_4$ (20 µM), sodium-L-ascorbate (200 µM), and H3K36me2 peptide (2 µM, 4 µM, 10 µM, 20 µM, 32 µM, 80 µM) in MES buffer (pH 7.0) were prepared in a deep 96-well plate (total volume per well = 600 µL). A solution of KDM2A in MES buffer (50 mM MES, 100 mM NaCl [pH 7.0]) was prepared (total volume = 12 mL) and divided equally into 3 falcon tubes, to which was added **(*S*,*S*)-6** (tube A (solution A): 0 nM, tube B (solution B): 500 nM, tube C (solution C): 1000 nM). After incubation at 0 °C for 45 min, 600 µL of solution A was added to each well, and the progress of demethylation was monitored *via* automated sampling using the RapidFire mass spectrometry platform (Agilent Technologies, Wakefield MA). The relative concentration of H3K36me1 was determined by integration of the extracted ion chromatogram corresponding to the H3K36me1 peptide using MassHunter software (Agilent Technologies). This procedure was repeated with solutions B and C using freshly-prepared peptide wells. Initial rates were determined by calculating the slope of the linear region of the reaction. Kinetic parameters are apparent values due to non-saturating conditions used in the assay.

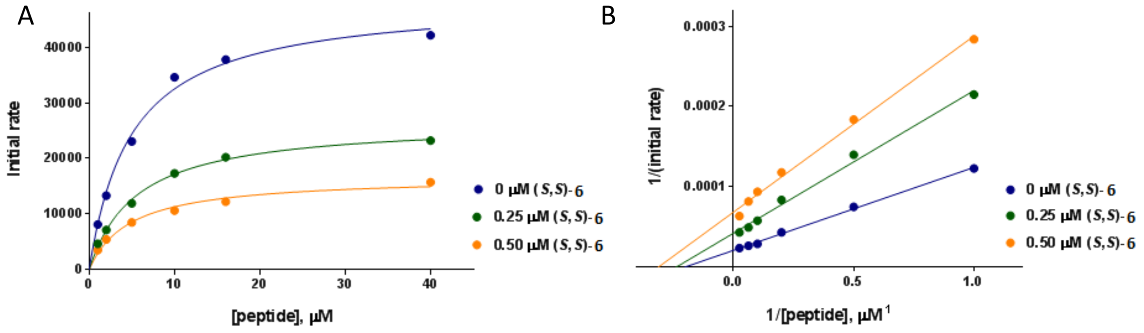

Figure S-6 – A) Michaelis-Menten plot of [peptide] vs. initial rate of peptide demethylation. The concentration of the 2-OG cosubstrate is 10 µM. Mixed-mode inhibition fit gives K_M^{app} (H3K36me2)= 5.0 ± 0.39 µM, Ki^{app} = 0.26 ± 0.05 µM; α = 1.1 ± 0.3, R^2 = 0.9914. B) Lineweaver-Burke plot.

Fluorescence Polarisation assay with Methylstat^{Fluoro}

Fluorescence polarization competition assay with methylstat^{fluoro} for KDM2A was carried out as described [25]. Detailed KDM7A and KDM7B FP assay optimization and methods will be reported elsewhere. In brief, assays were carried out in 384-well, black, non-binding microplate (Greiner Bio-One) at 22 °C. KDM7s (150 nM

KDM7A, 600 nM KDM7B or 50 nM KDM7C) were incubated with 12.5 nM methylstat[fluoro] or 50 nM $H3_{(1-15)}$K4Me3K9Me2-FITC, 100 µM $NiSO_4$, and compounds at various concentrations in 50 mM Hepes buffer (pH7.0), containing 50 mM NaCl and 0.1% Tween 20. Enzyme was not pre-incubated with compounds to avoid stabilising effect. The plate was incubated in the dark for 30 minutes and analysed by PheraStar (BMG).

A

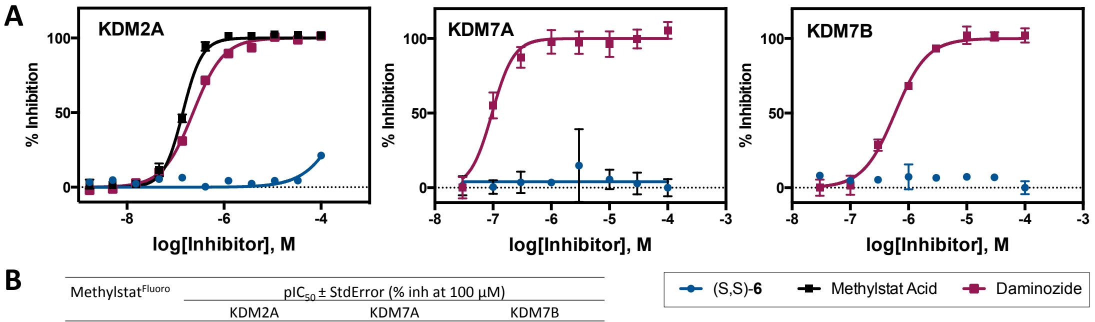

B

Methylstat^{Fluoro}	pIC_{50} ± StdError (% inh at 100 µM)		
	KDM2A	KDM7A	KDM7B
(S,S)-6	< 4.0 (21%)	< 4.0	< 4.0
Daminozide	6.64 ± 0.02	7.02 ± 0.03	6.23 ± 0.03
Methylstat acid	6.85 ± 0.01	-	-

C

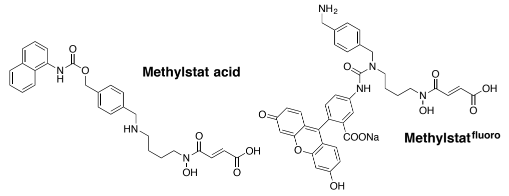

Figure S-7 – Fluorescence Polarisation competition assay with Methystat[Fluoro]. (A) FP competition assay with methylstat[fluro] for KDM2A, KDM7A and KDM7B with (S,S)-6 and control inhibitors. **(B)** pIC_{50} values calculated for methylstat[Fluoro] competition assay. **(C)** Structures of methylstat and methylstat[Fluoro].

VII) Photoaffinity Labelling

Photoaffinity Labelling of KDM2A with 127

A solution containing KDM2A (1 µM), 127 (4 µM), $NiCl_2$ (50 µM), and 2-OG (10 µM) was prepared using MES buffer (50 mM MES, 100 mM NaCl [pH 7.0]) and incubated at 0 °C for 45 min. The solution was irradiated in 60 µL aliquots with 350 nm uv-light at 1-4 °C using a CaproBox™ (Caprotec, Berlin). Irradiation times longer than 5 min were carried out in 5 min pulses, separated by 2 min intervals to prevent sample warming. Samples were analyzed by liquid-chromatography/mass-spectrometry.

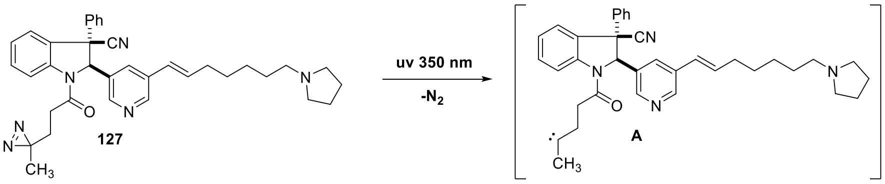

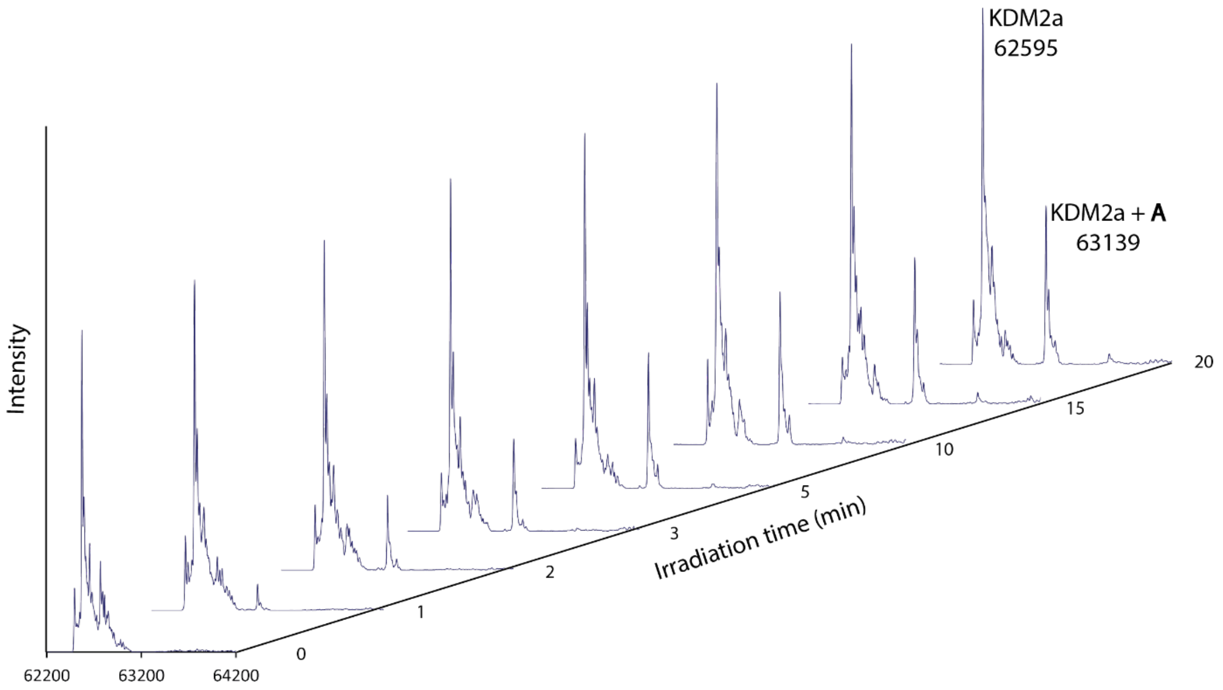

Figure S-8 – Progress of KDM2A photo-crosslinking by **127** as a function of irradiation time assessed by mass spectometry.

Distribution of photo-crosslinked residues

To map photo-crosslinked amino acide residues by **127** within the KDM2A protein, samples were digested in-solution with elastase, followed by analysis by liquid chromatography tandem mass spectrometry (LC-MS/MS) as described previously.[31] MS data analysis and identification of uv cross-linking sites was performed using PEAKS Studio software (v7.5). Complete coverage of the KDM2A sequence was achieved.

MEPEEERIRYSQRLRGTMRRRYEDDGISDDEIEGKRTFDLEEKLHTNKYNANFVTFMEGKDFNVEYIQRGGLRDPLIFKNSDGLGIKMPDPDFTVNDVKMCVG

SRRMVDVMDVNTQKGIEMTMAQWTRYYETPEEEREKLYNVISLEFSHTRLENMVQRPSTVDFIDWVDNMWPRHLKESQTESTNAILEMQYPKVQKYCLMSVRG

CYTDFHVDFGGTSVWYHIHQGGKVFWLIPPTAHNLELYENWLLSGKQGDIFLGDRVSDCQRIELKQGYTFVIPSGWIHAVYTPTDTLVFGGNFLHSFNIPMQL

KIYNIEDRTRVPNKFRYPFYYEMCWYVLERYVYCITNRSHLTKEFQKESLDLELNGLESGNGDEEAVDREPRRLSSRRSVLTSPVANGVNLDYDGLGKTCRSL

PSLKKTLAGDSSSDCSRGSHNGQVWDPQCAPRKDRQVHLTHFELEGLRCLVDKLESLPLHKKCVPTGIEDEDALIADVKILLEELANSDPKLALTGVPIVQWP

Figure S-9 – Sequence of KDM2A construct used for all experiments, including photoaffinity labelling. Residues highlighted in red were covalently modified by **127** upon uv irradiation. Residues highlighted in yellow are exposed within the enzyme

active site (i.e. in close proximity to the peptide substrate or 2-OG). The residue highlighted in green is both exposed within the enzyme active site and undergoes photolabelling by **127**.

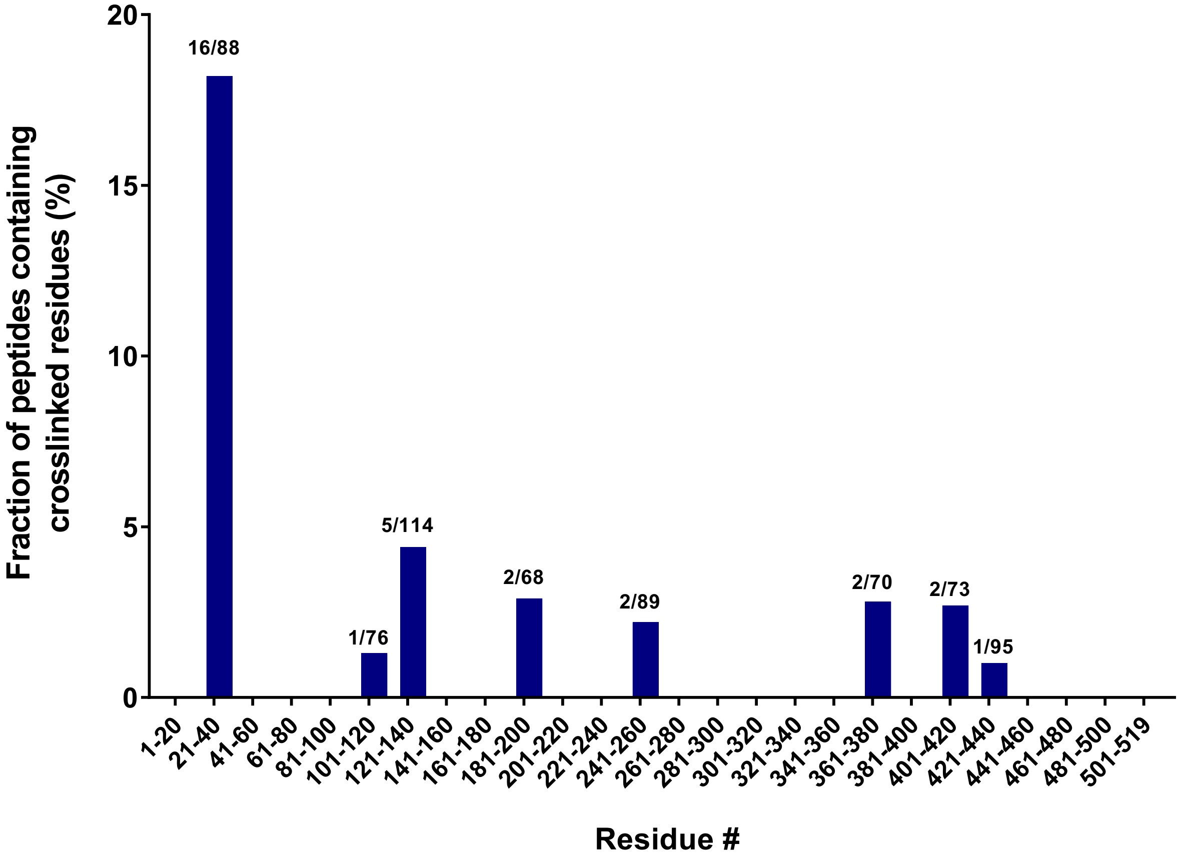

Figure S-10 – Distribution of labelled peptides, showing apparent preference for photo-crosslinking of residues near the protein *N*-terminus. Observed ratios of labelled to unlabelled peptides as analysed using PEAKS are displayed above each bar.

VIII) Computational Docking

Compounds were docked into the published KDM2A structure (PDB ID 2YU1) using the MolSoft ICM [30]. The Dock Table function in the Advanced mode of the ligedit menu was used. Three poses were kept from a docking run with thoroughness of 20. Representatives poses for compounds **1** and ***(S,S)*-6** are shown overlayed with the structure of mouse KDM2A, H3.2 and *N*-oxalyl glycine [31] in Figure S-11. Although the pyrido-indole

and -indoline tails fill different parts of the peptide binding pocket, docking places the alkyl pyrrolidine moiety in the same region as the quarternary trimethyl lysine substrate.

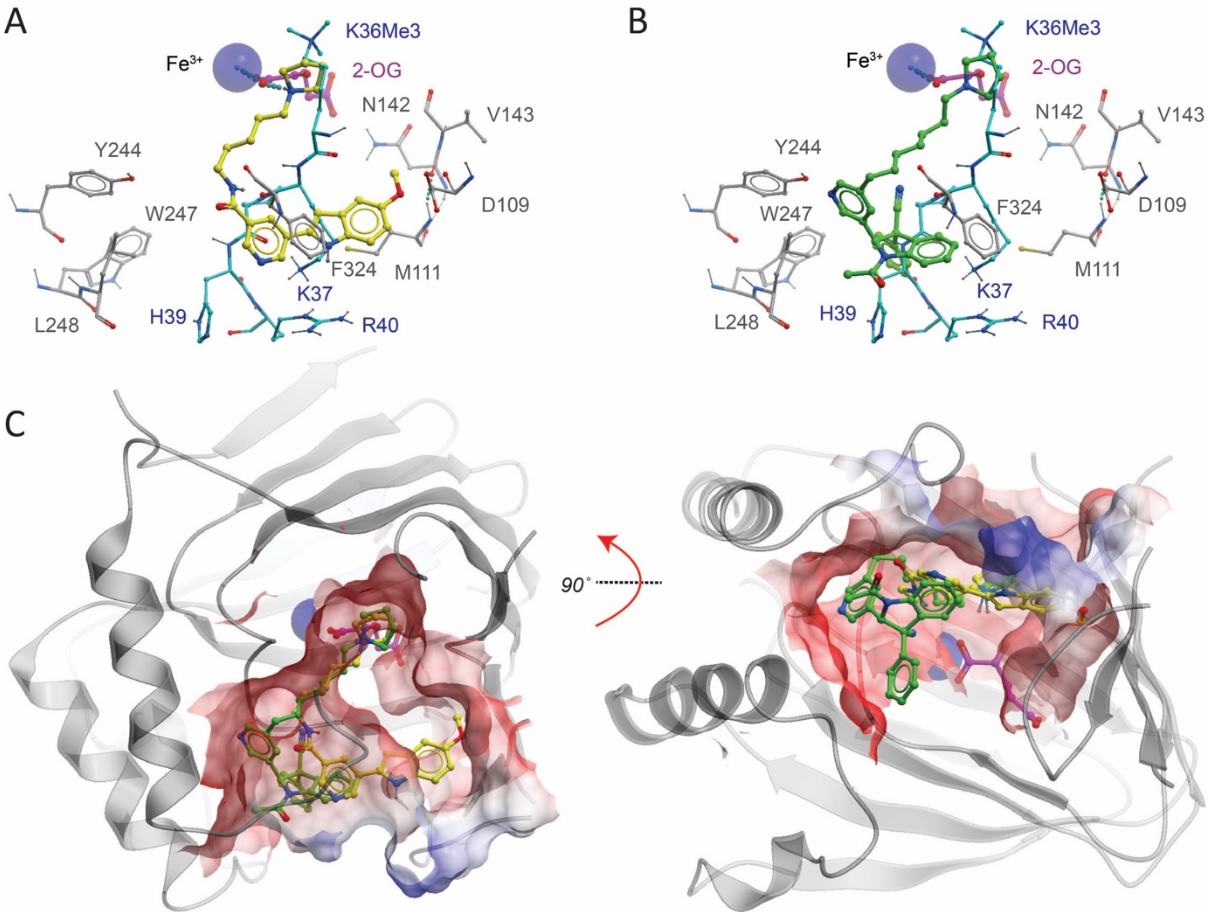

Figure S-11: Computational docking of compounds in the KDM2A peptide binding pocket using MolSoft ICM. A. Overlay of compound **1** (yellow ball and stick) and H3.2 (cyan sticks from PDB ID 4QXB) in 2-OG (magenta ball and stick from PDB ID 2YU1) bound KDM2A (grey stick from PDB ID 2YU1). B. Overlay of compound **(*S,S*)-6** (green ball and stick) as in A. C. Electrostatic surface view of compounds **1** and **(*S,S*)-6** binding to the histone substrate pocket of KDM2A (grey ribbon from PDB ID 2YU1).

IX) NMR Spectra

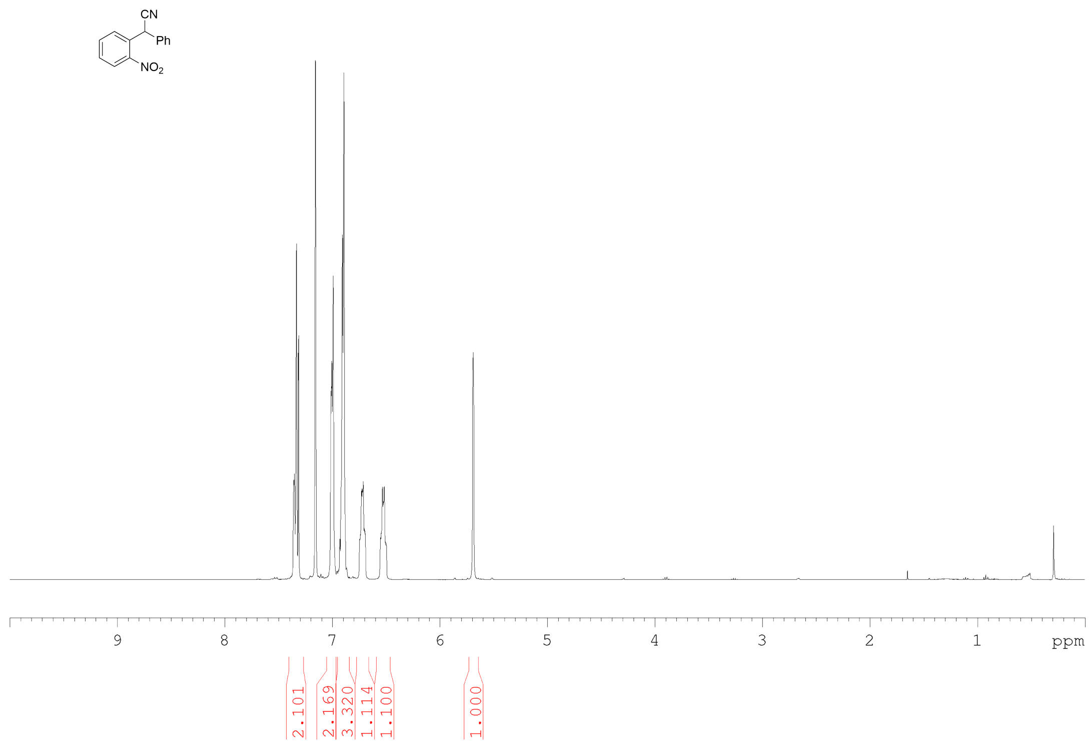
CN
Ph
NO2
9 8 7 6 5 4 3 2 1 ppm
2.101
2.169
3.320
1.114
1.100
1.000

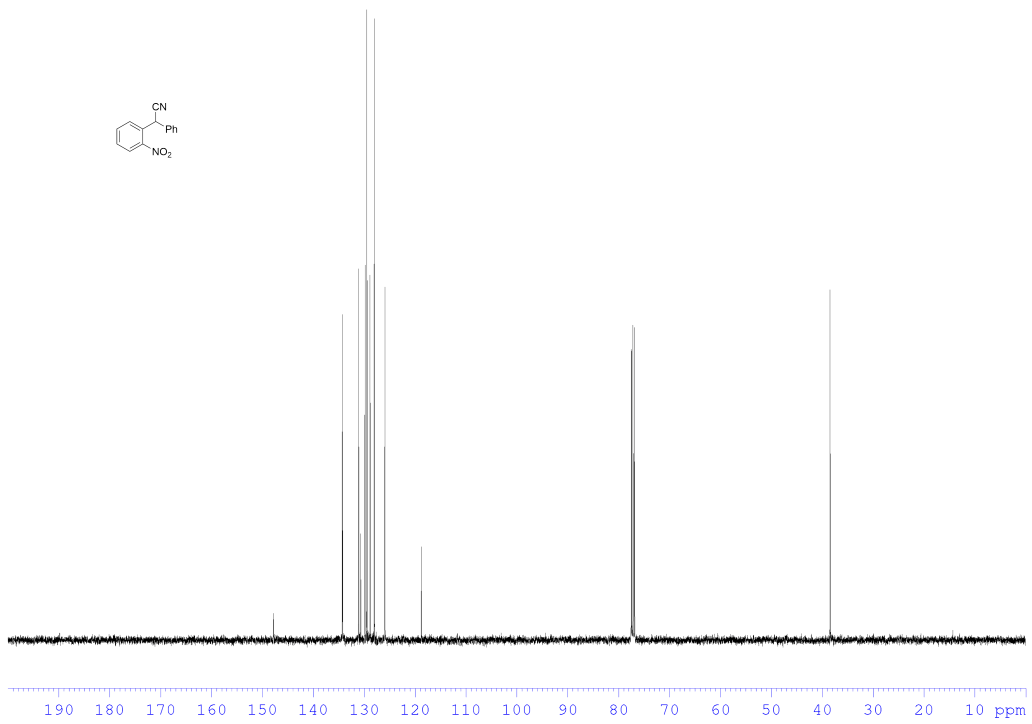
CN
Ph
NO2
190 180 170 160 150 140 130 120 110 100 90 80 70 60 50 40 30 20 10 ppm

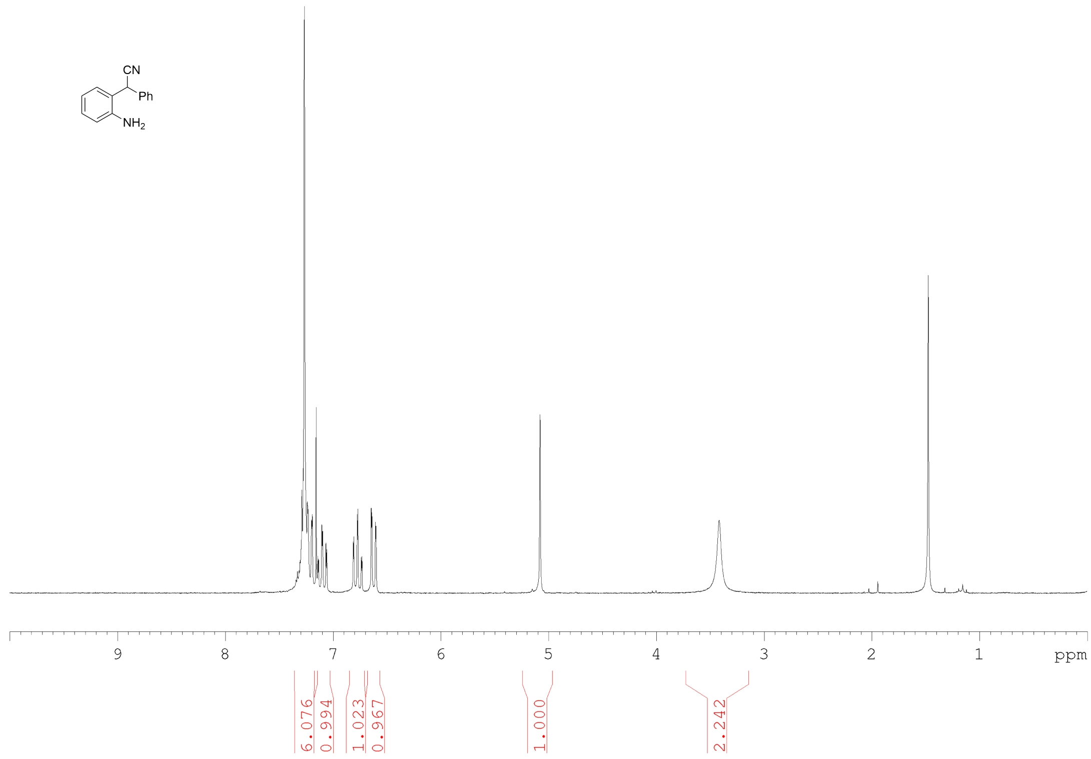
CN
Ph
NH2
9 8 7 6 5 4 3 2 1 ppm
6.076 0.994 1.023 0.967 1.000 2.242

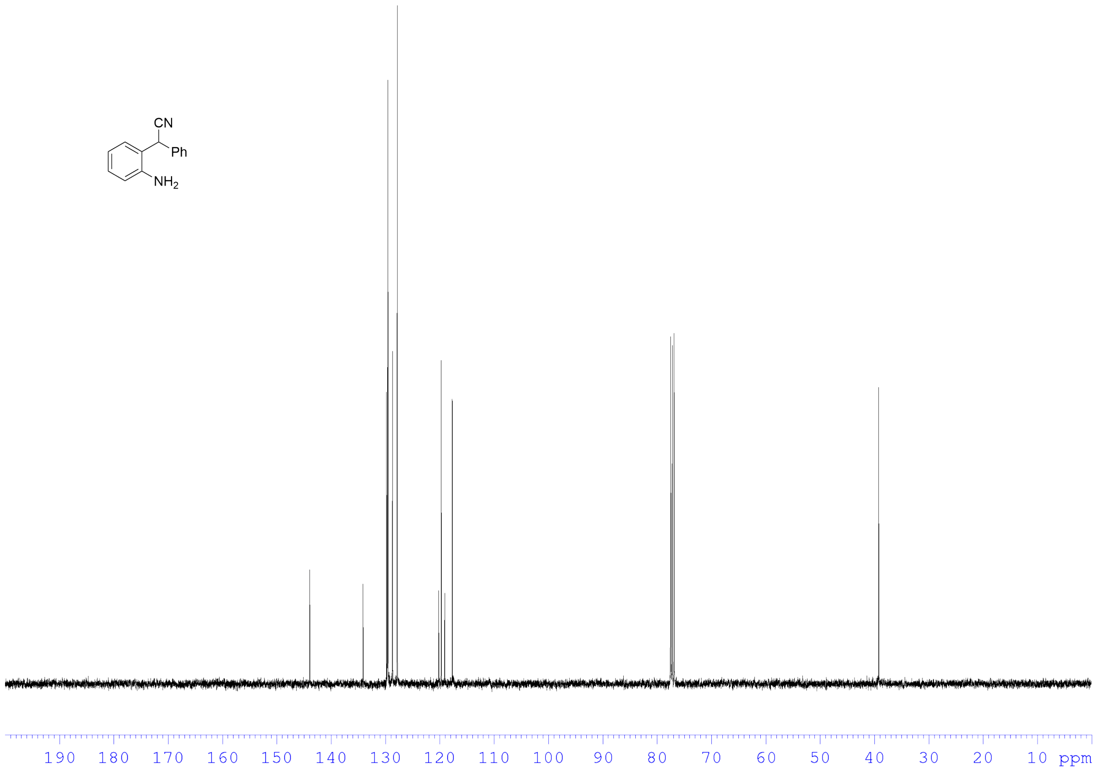
CN
Ph
NH2
190 180 170 160 150 140 130 120 110 100 90 80 70 60 50 40 30 20 10 ppm

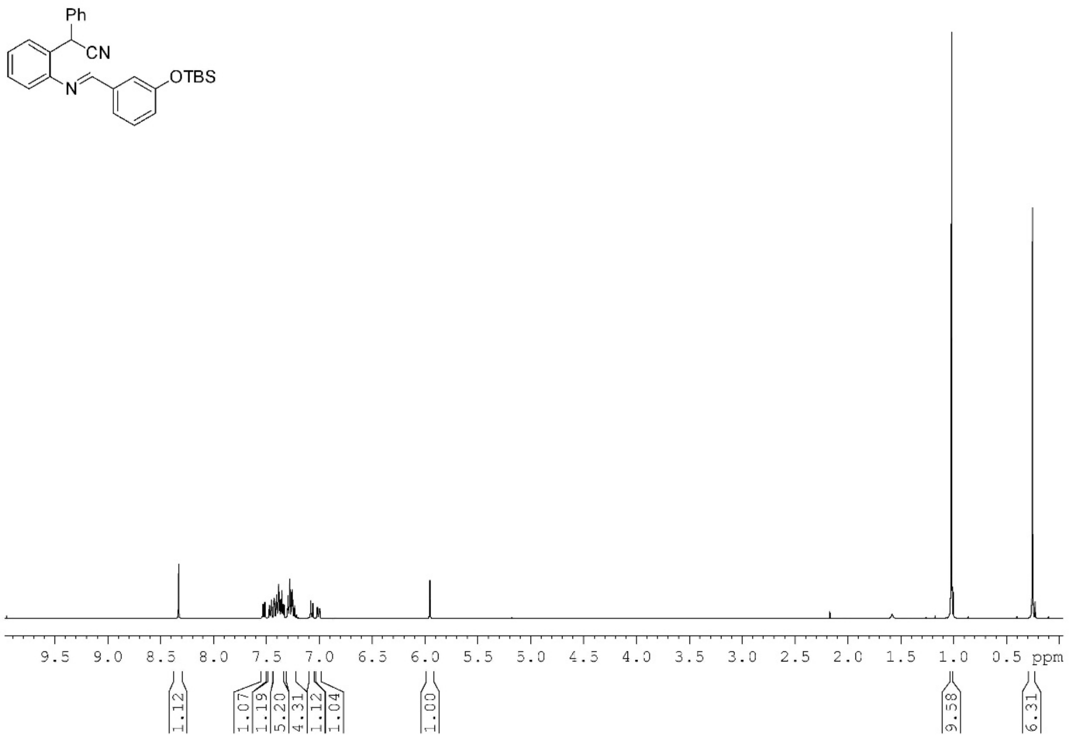
Ph
CN
N
OTBS
9.5 9.0 8.5 8.0 7.5 7.0 6.5 6.0 5.5 5.0 4.5 4.0 3.5 3.0 2.5 2.0 1.5 1.0 0.5 ppm
1.12
1.07
1.19
5.20
4.31
1.12
1.04
1.00
9.58
6.31

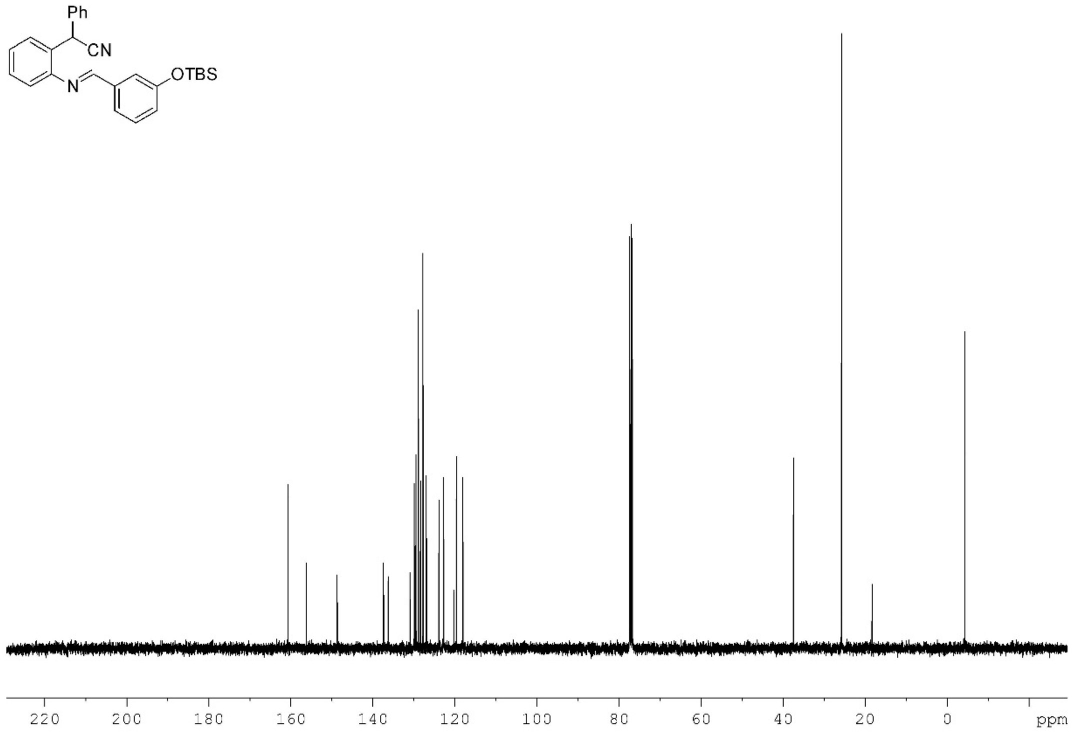
Ph
CN
N
OTBS
220 200 180 160 140 120 100 80 60 40 20 0 ppm

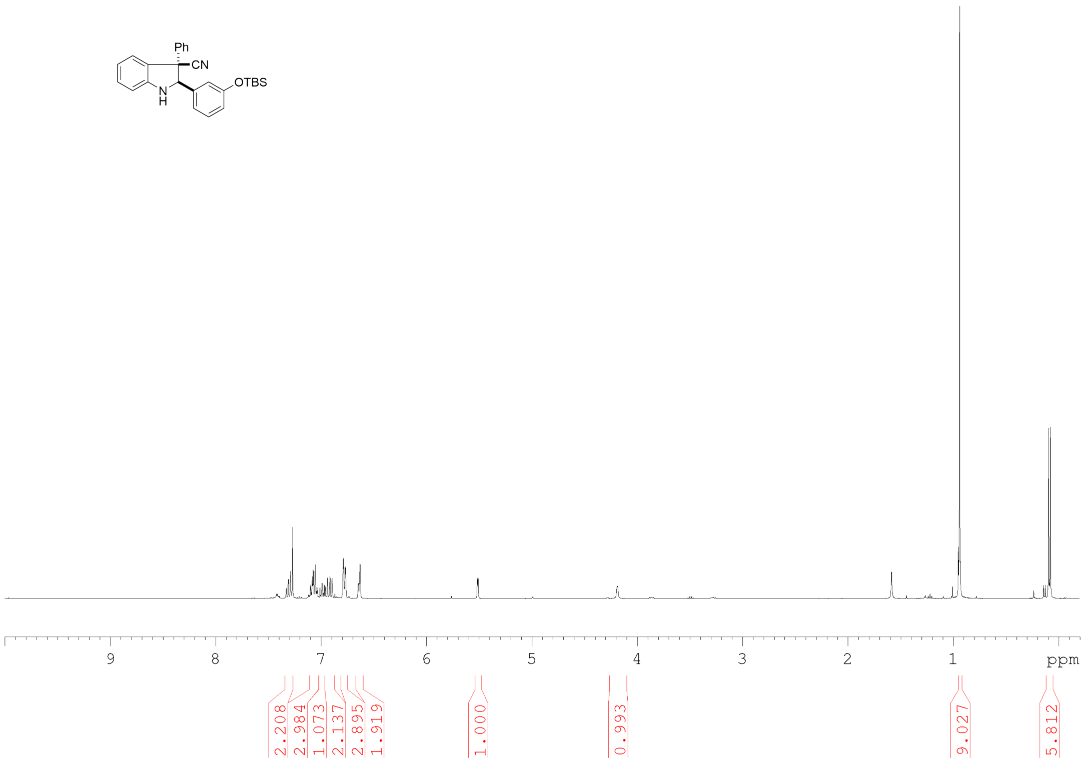
Ph
CN
OTBS
N
H
9
8
7
6
5
4
3
2
1
ppm
2.208
2.984
1.073
2.137
2.895
1.919
1.000
0.993
9.027
5.812

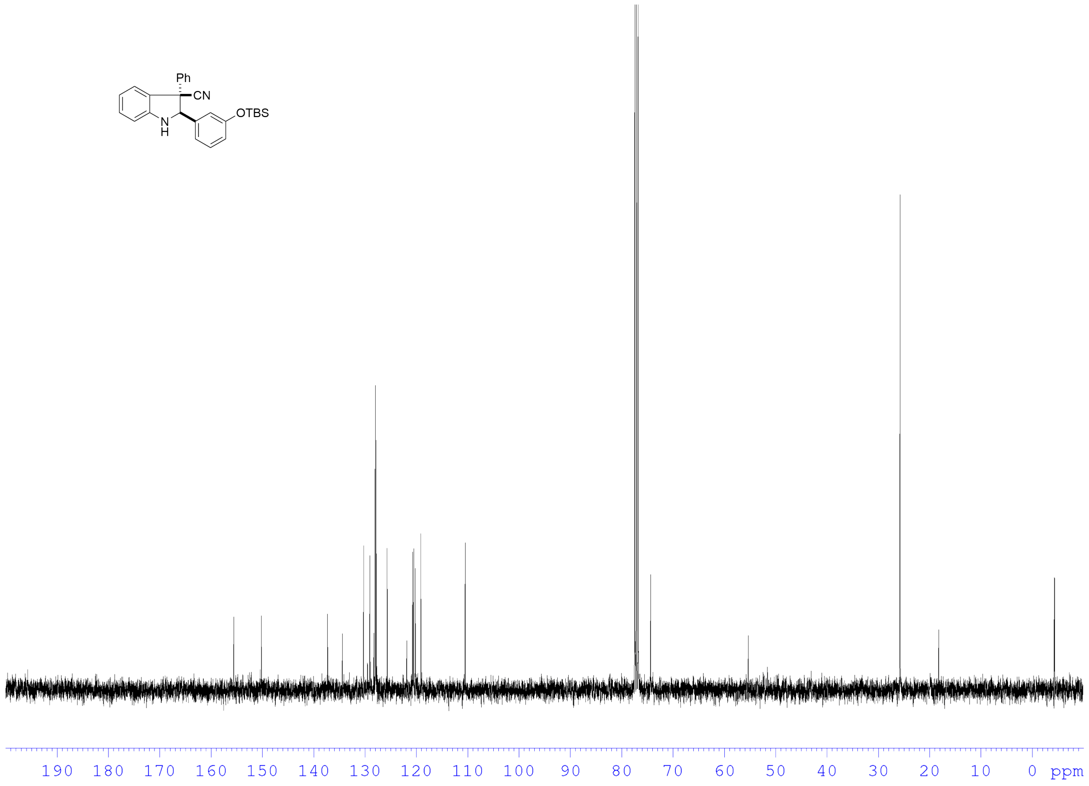
Ph
CN
OTBS
N
H
190 180 170 160 150 140 130 120 110 100 90 80 70 60 50 40 30 20 10 0 ppm

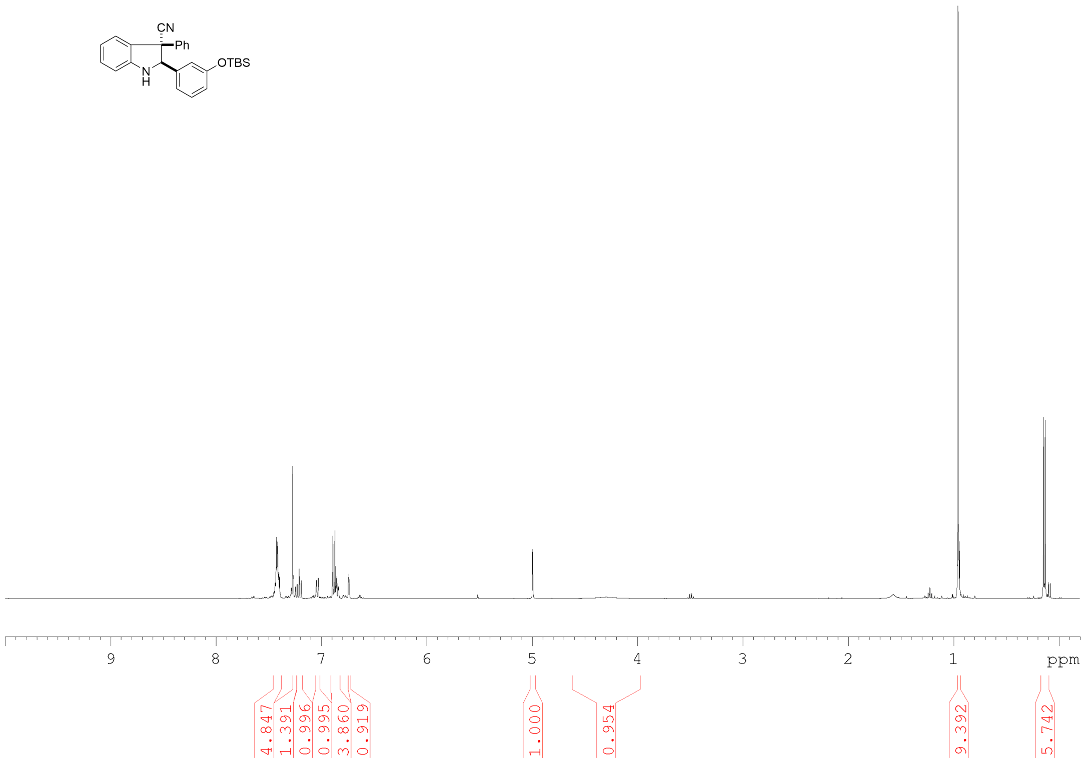
CN
Ph
OTBS
N
H
9
8
7
6
5
4
3
2
1
ppm
4.847
1.391
0.996
0.995
3.860
0.919
1.000
0.954
9.392
5.742

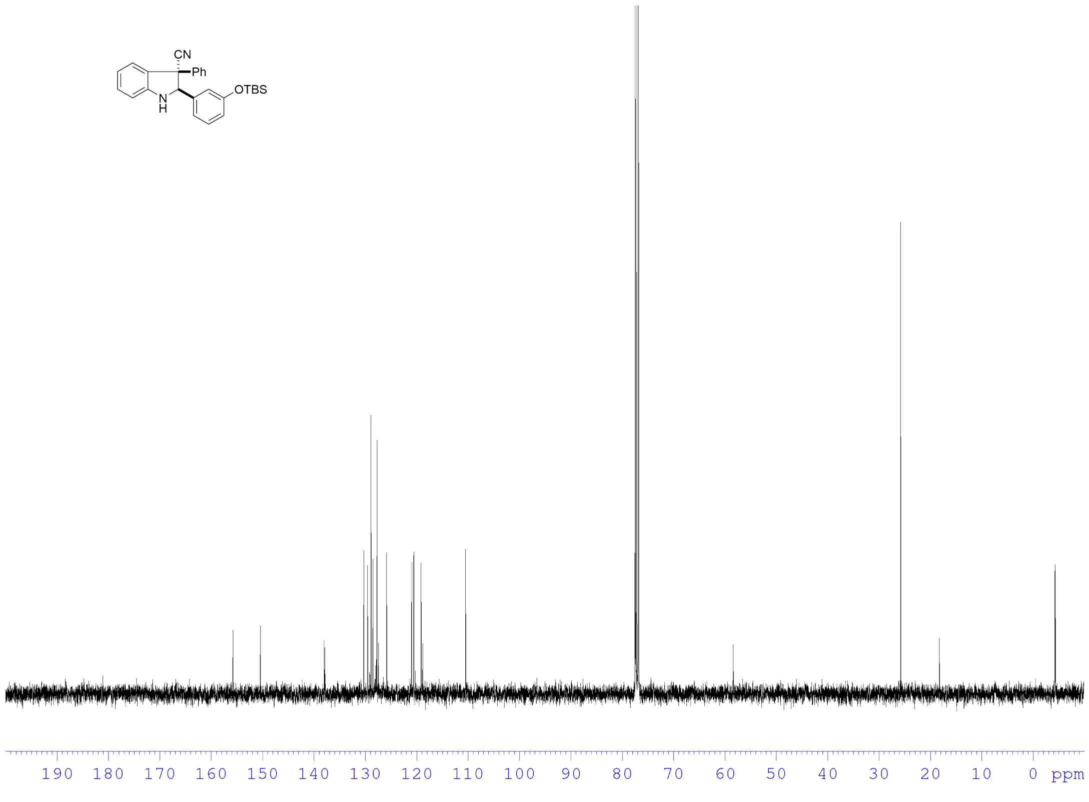
CN
Ph
OTBS
N
H
190 180 170 160 150 140 130 120 110 100 90 80 70 60 50 40 30 20 10 0 ppm

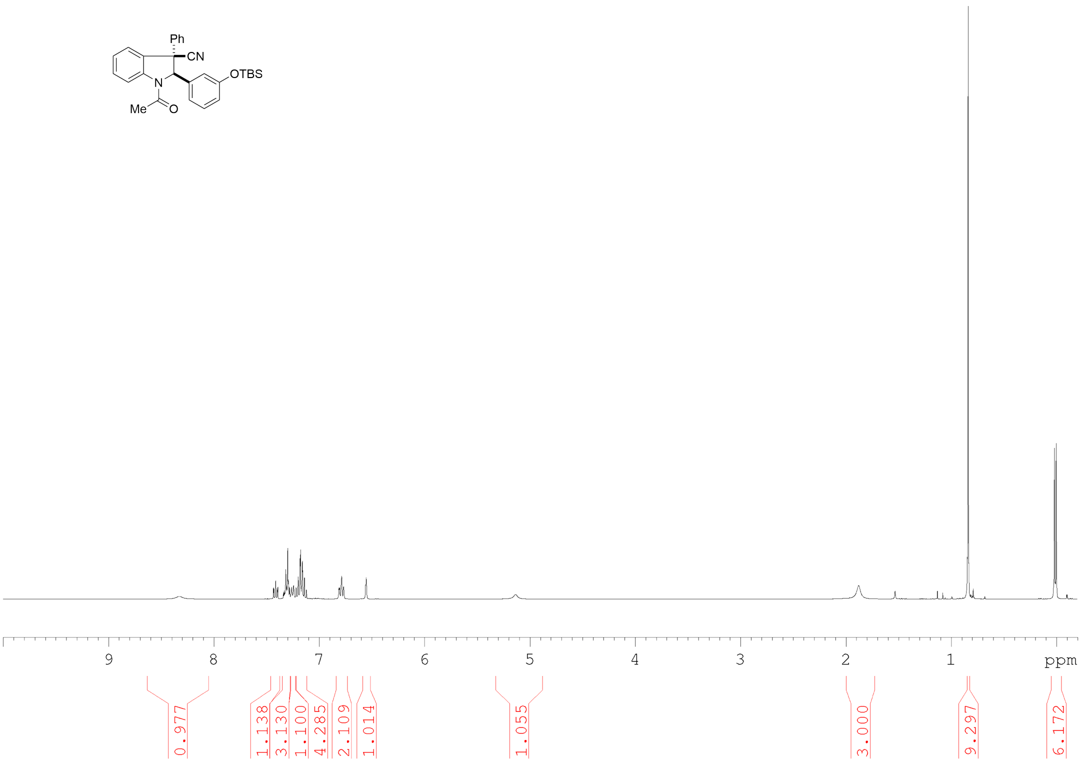
Ph
CN
OTBS
N
Me
O
9 8 7 6 5 4 3 2 1 ppm
0.977
1.138
3.130
1.100
4.285
2.109
1.014
1.055
3.000
9.297
6.172

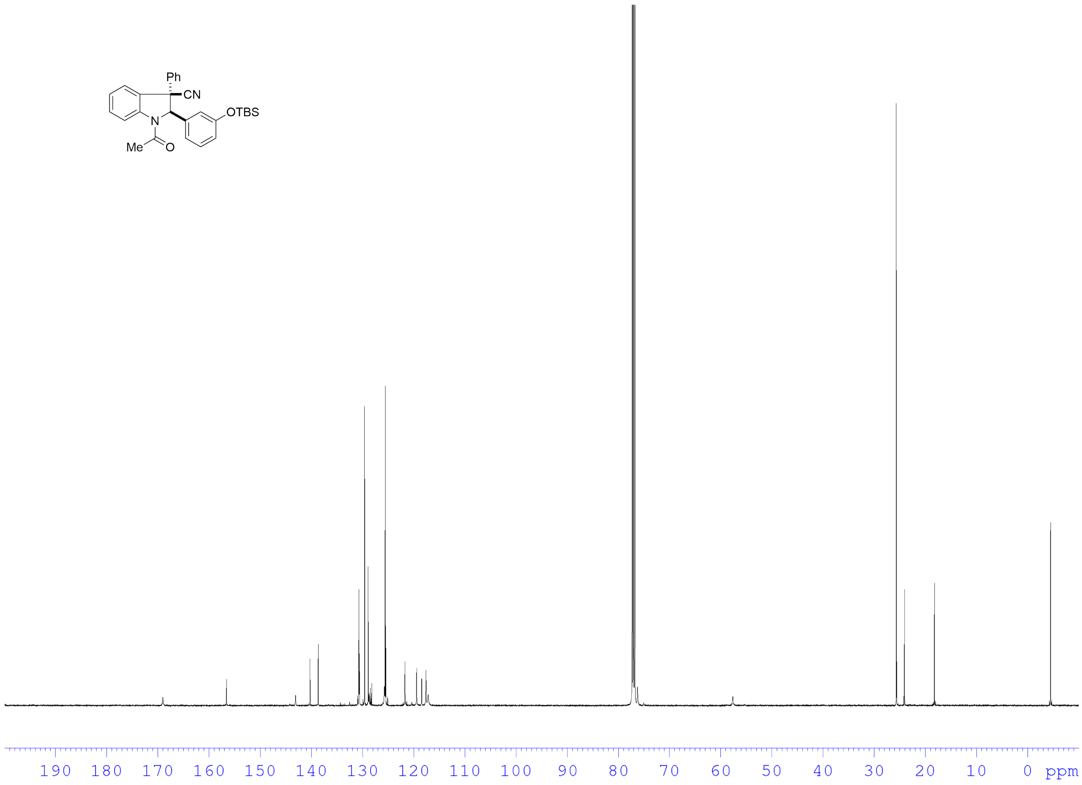
Ph
CN
OTBS
N
Me
O
190 180 170 160 150 140 130 120 110 100 90 80 70 60 50 40 30 20 10 0 ppm

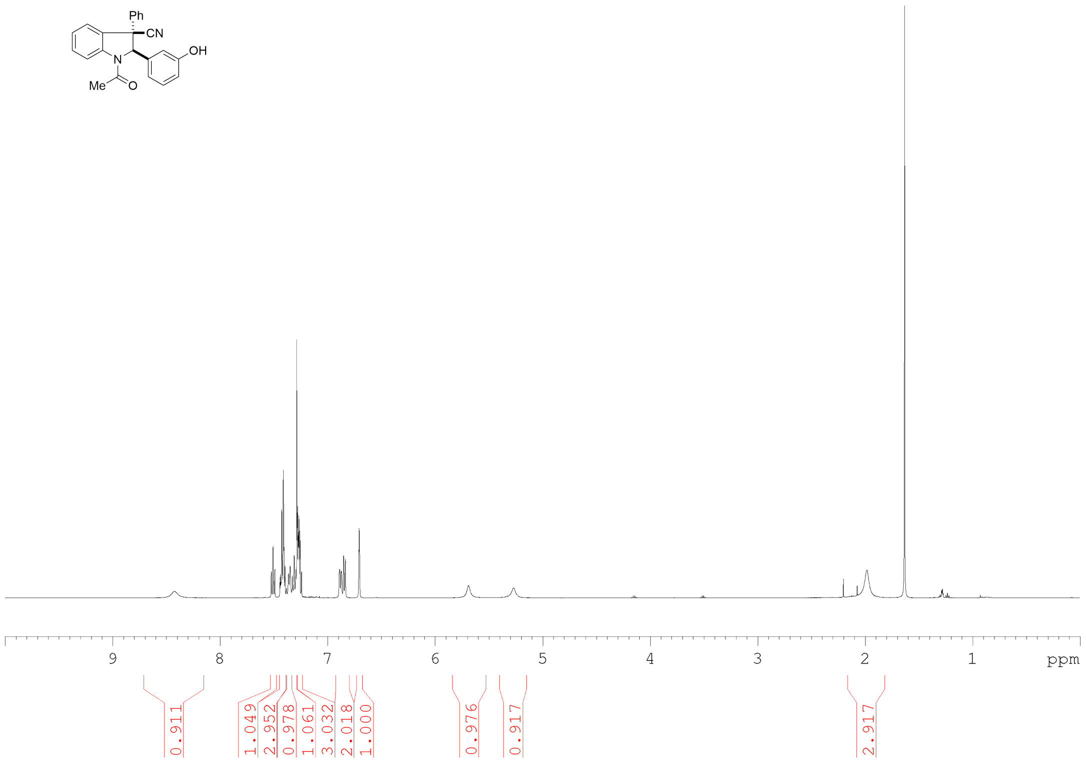
Ph
CN
N
OH
Me
O
9
8
7
6
5
4
3
2
1
ppm
0.911
1.049
2.952
0.978
1.061
3.032
2.018
1.000
0.976
0.917
2.917

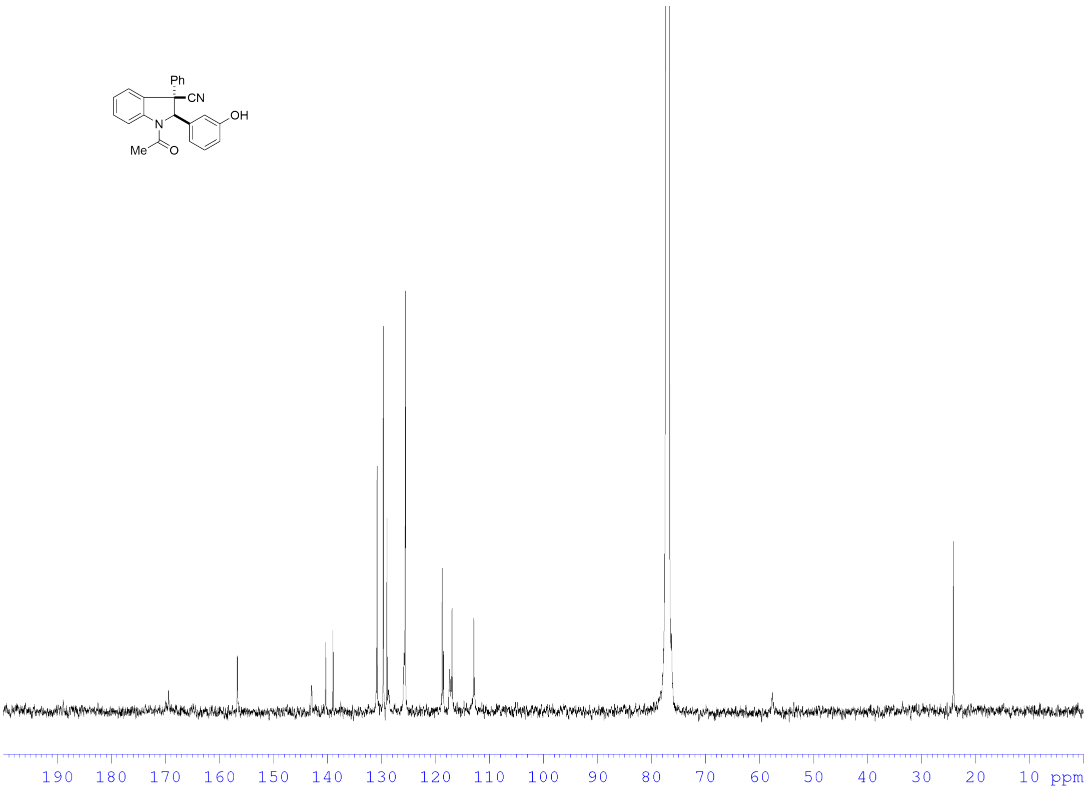
Ph
CN
N
OH
Me
O
190
180
170
160
150
140
130
120
110
100
90
80
70
60
50
40
30
20
10
ppm

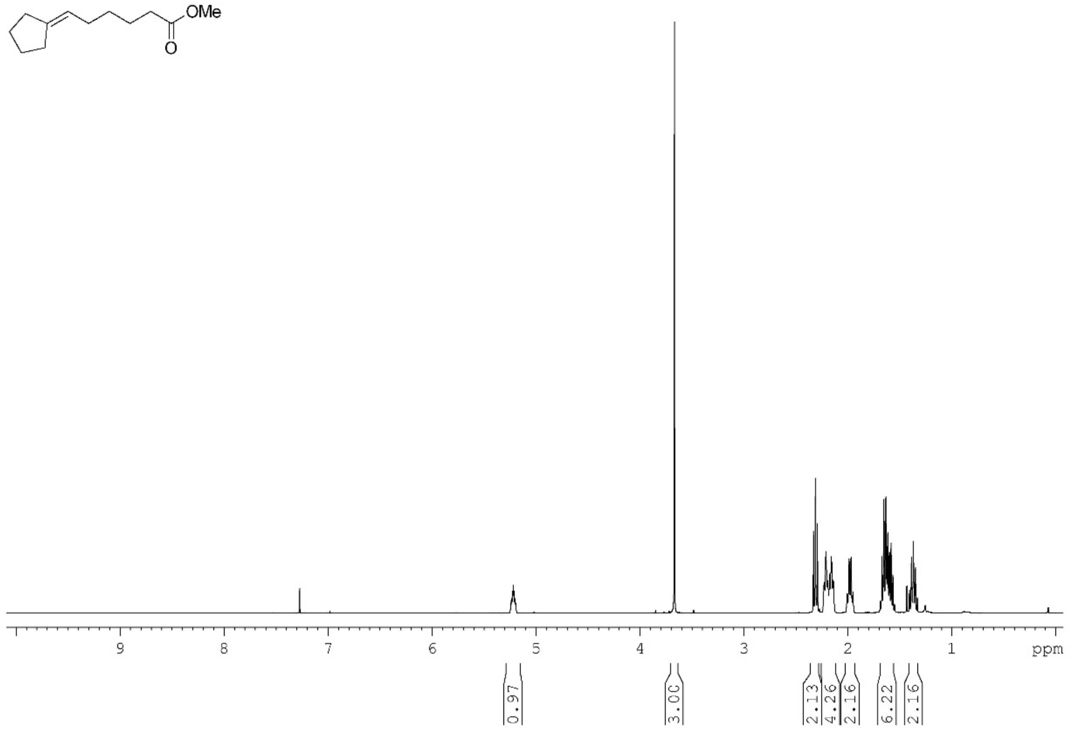
OMe
O
9 8 7 6 5 4 3 2 1 ppm
0.97
3.00
2.13 4.26 2.16 6.22 2.16

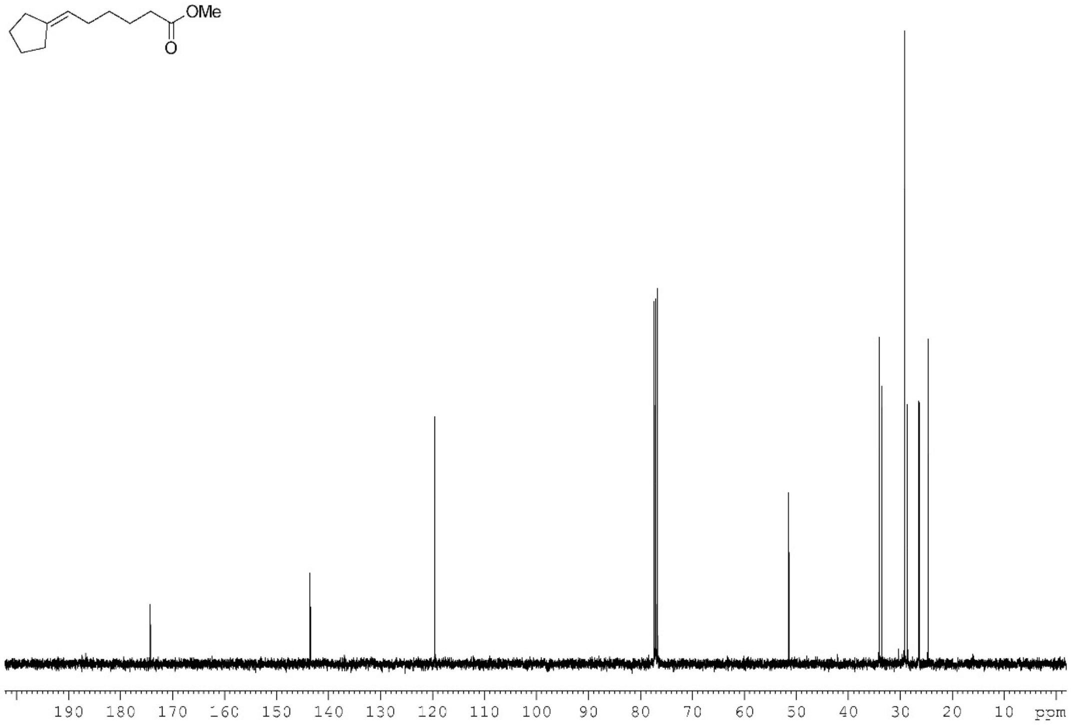
OMe
O
190 180 170 160 150 140 130 120 110 100 90 80 70 60 50 40 30 20 10 ppm

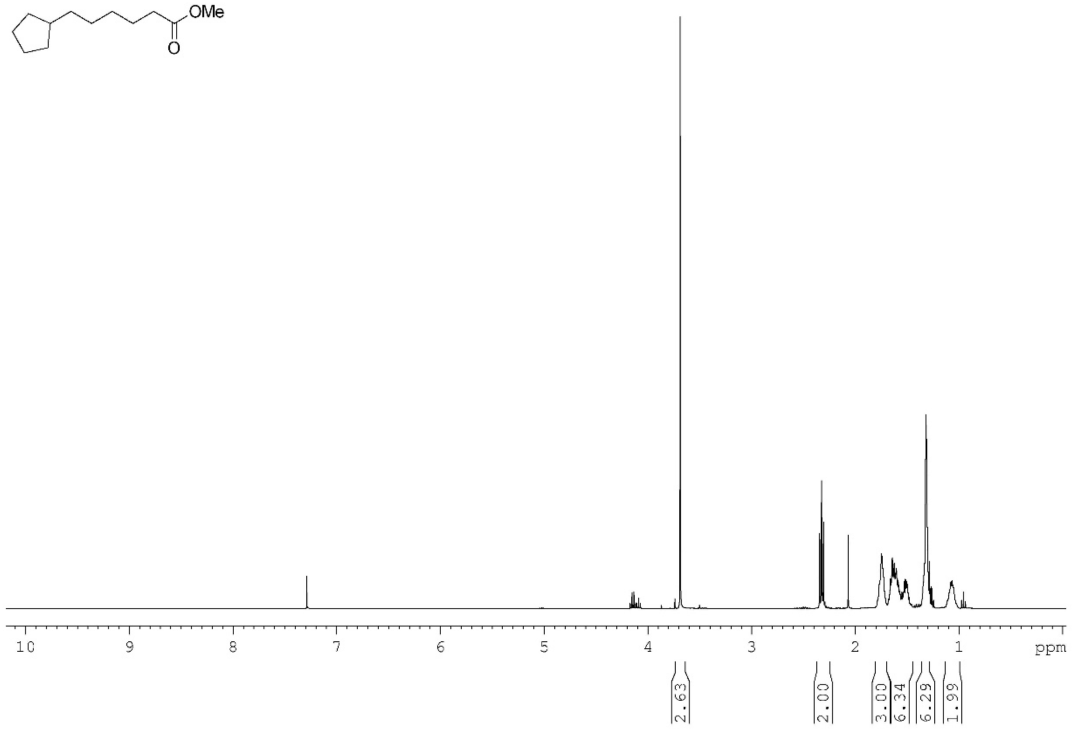
OMe
O
10
9
8
7
6
5
4
3
2
1
ppm
2.63
2.00
3.00
6.34
6.29
1.99

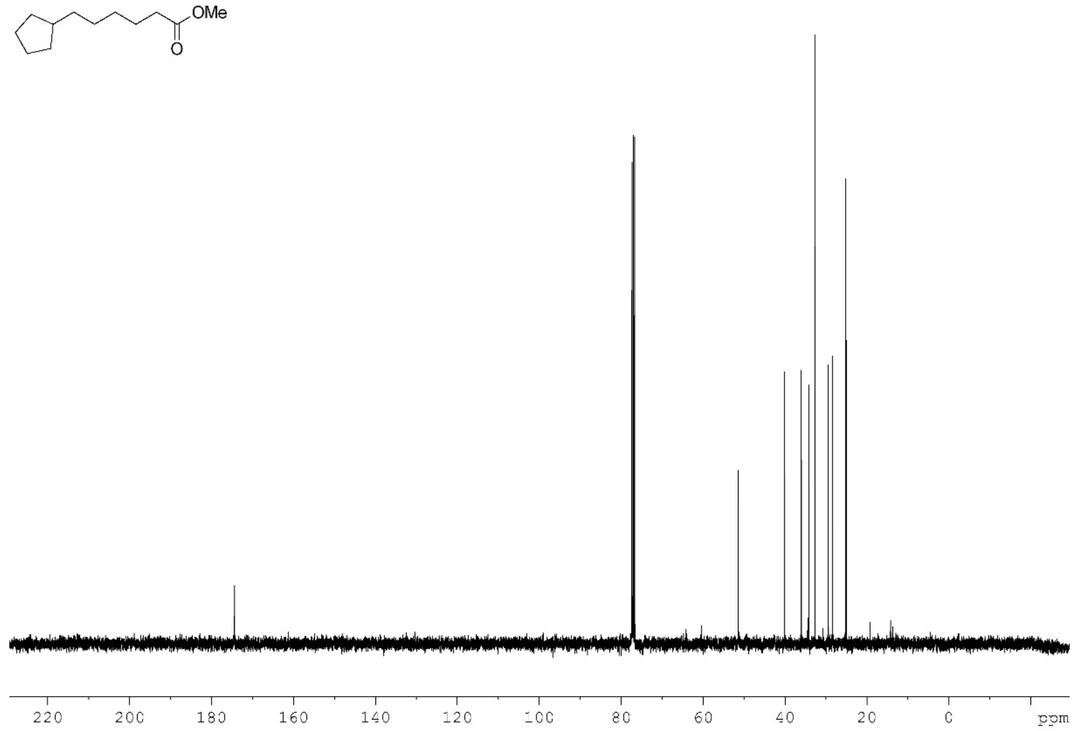
OMe
O
220
200
180
160
140
120
100
80
60
40
20
0
ppm

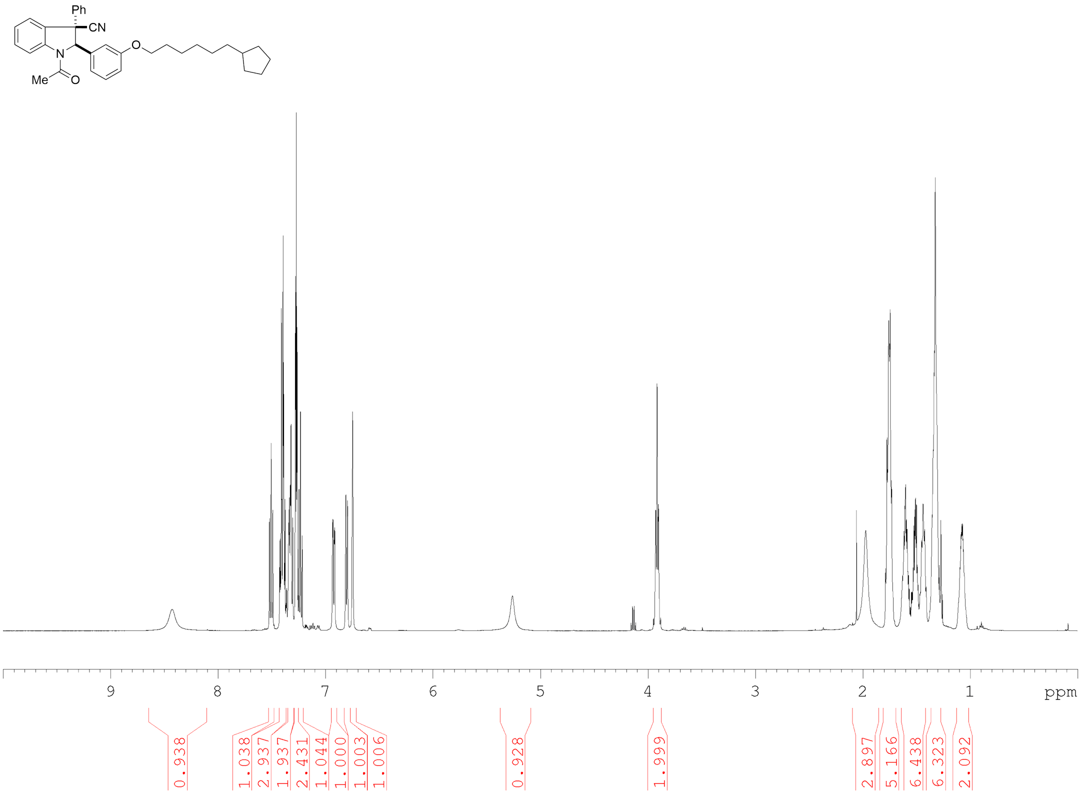
Ph
CN
N
O
Me
O
9 8 7 6 5 4 3 2 1 ppm
0.938
1.038
2.937
1.937
2.431
1.044
1.000
1.003
1.006
0.928
1.999
2.897
5.166
6.438
6.323
2.092

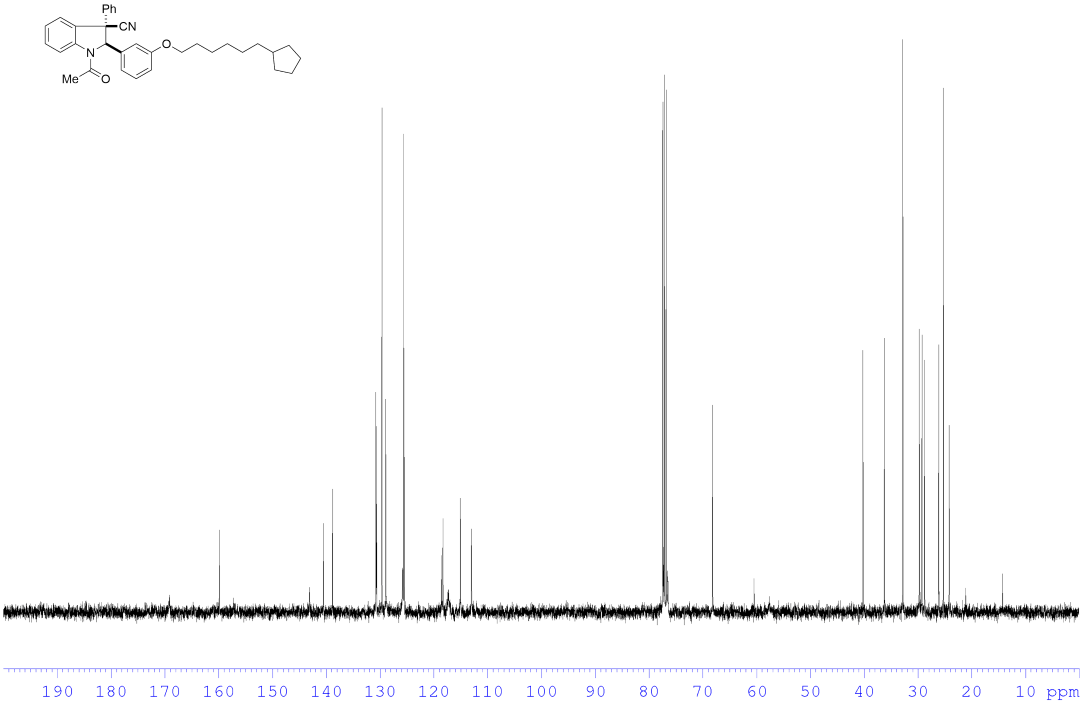
Ph
CN
N
O
Me
O
190 180 170 160 150 140 130 120 110 100 90 80 70 60 50 40 30 20 10 ppm

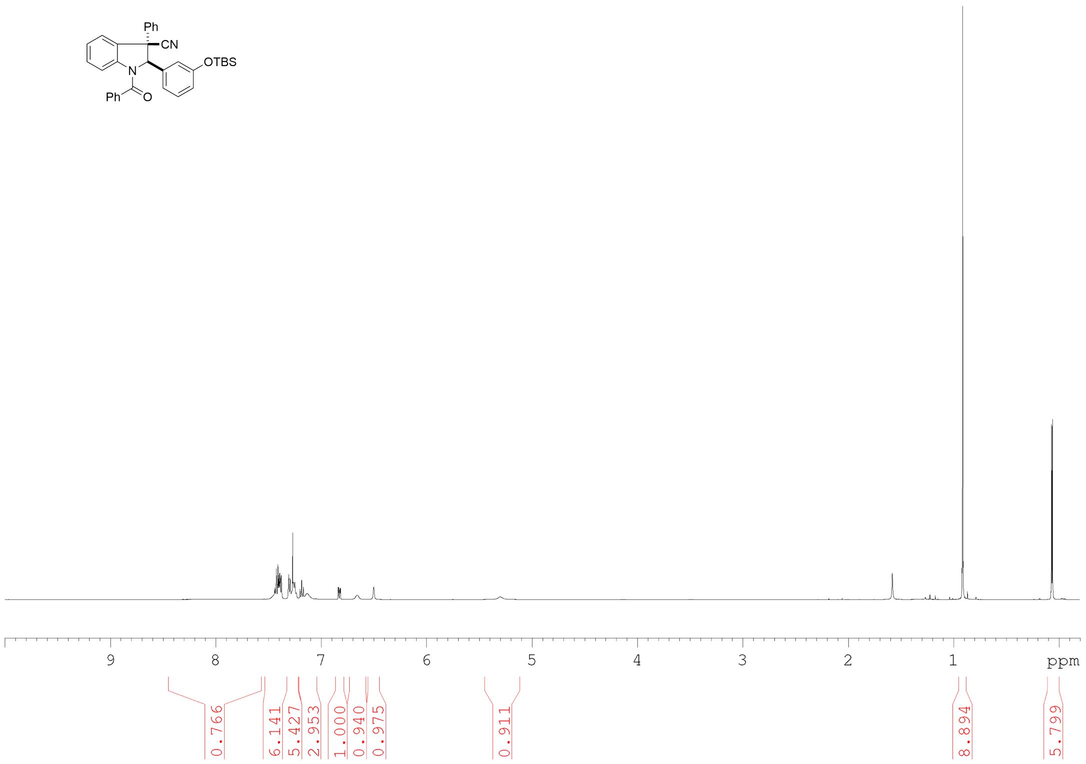
Ph
CN
N
OTBS
Ph
O
9
8
7
6
5
4
3
2
1
ppm
0.766
6.141
5.427
2.953
1.000
0.940
0.975
0.911
8.894
5.799

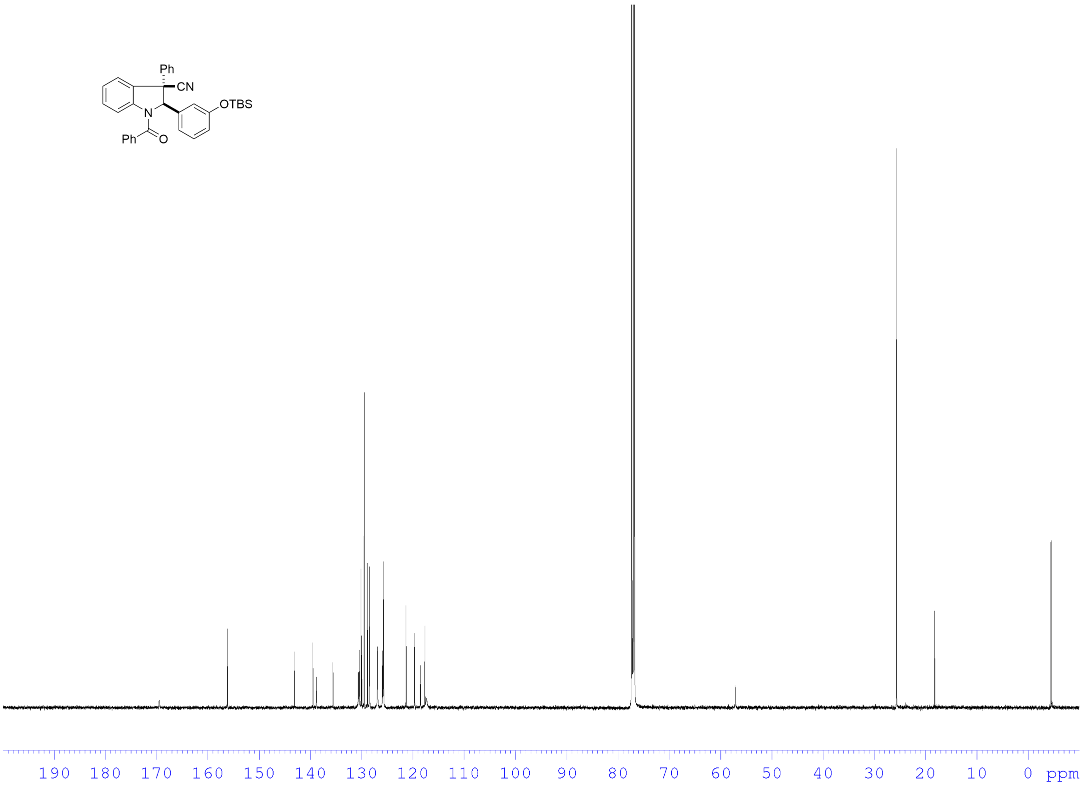
Ph
CN
N
OTBS
Ph
O
190
180
170
160
150
140
130
120
110
100
90
80
70
60
50
40
30
20
10
0
ppm

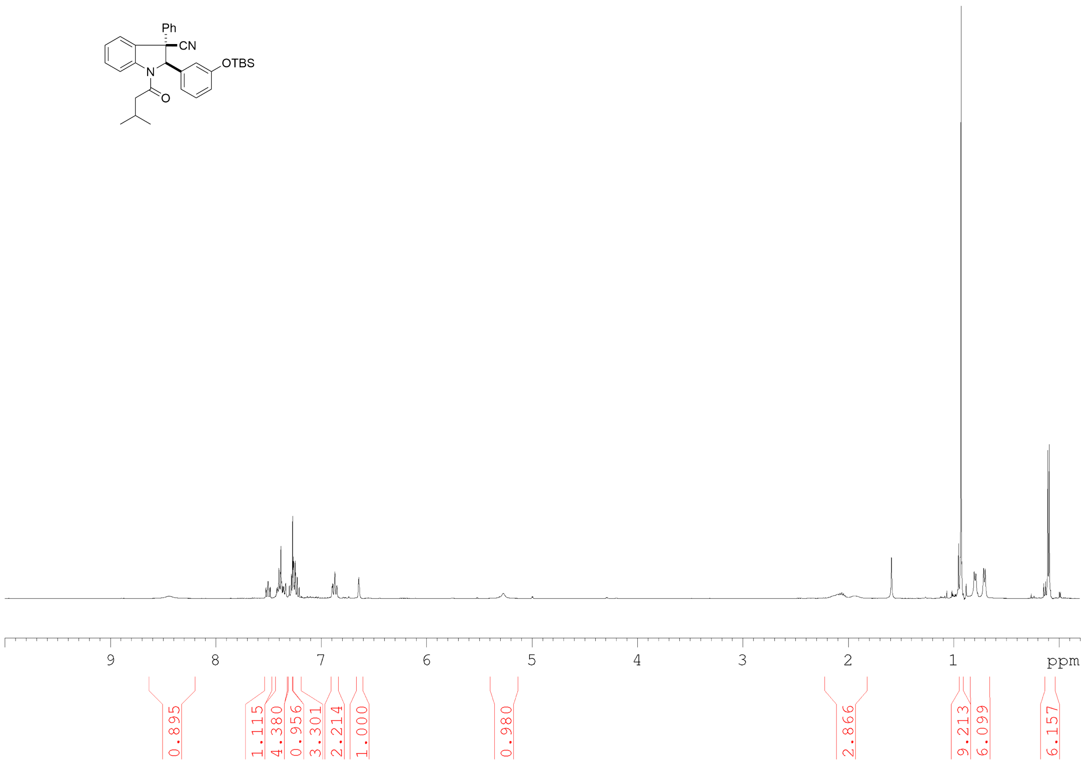
Ph
CN
OTBS
N
O
9 8 7 6 5 4 3 2 1 ppm
0.895
1.115
4.380
0.956
3.301
2.214
1.000
0.980
2.866
9.213
6.099
6.157

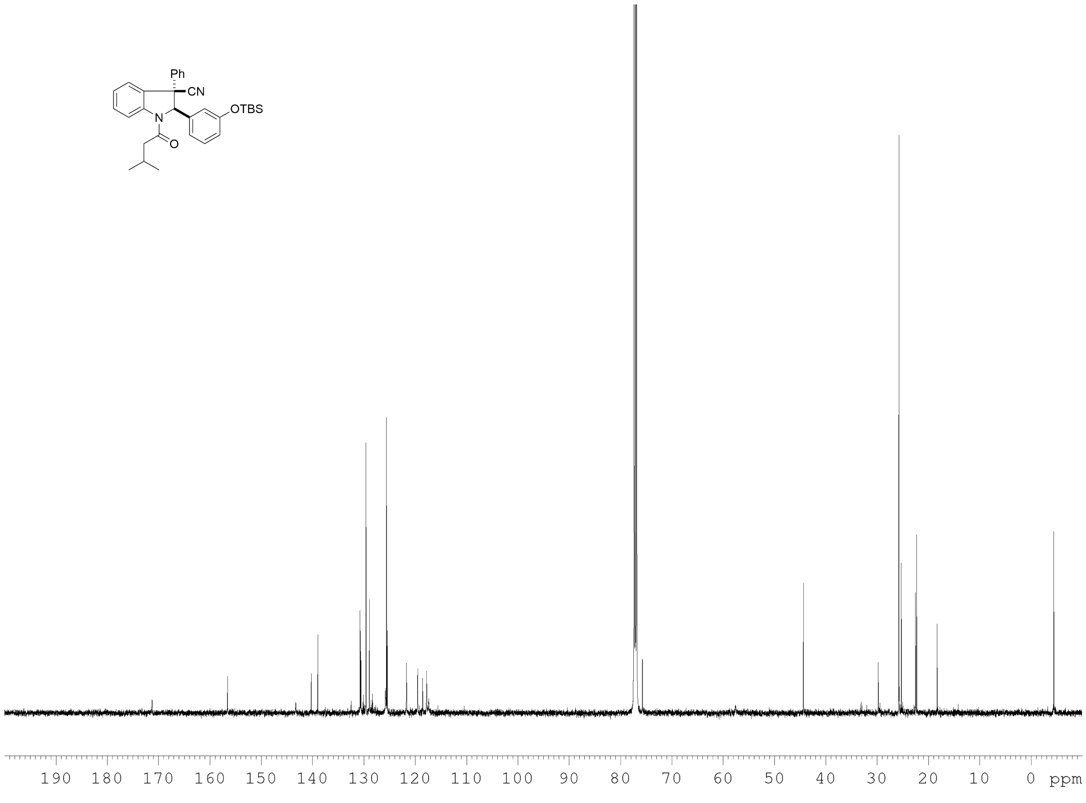
Ph
CN
OTBS
N
O
190 180 170 160 150 140 130 120 110 100 90 80 70 60 50 40 30 20 10 0 ppm

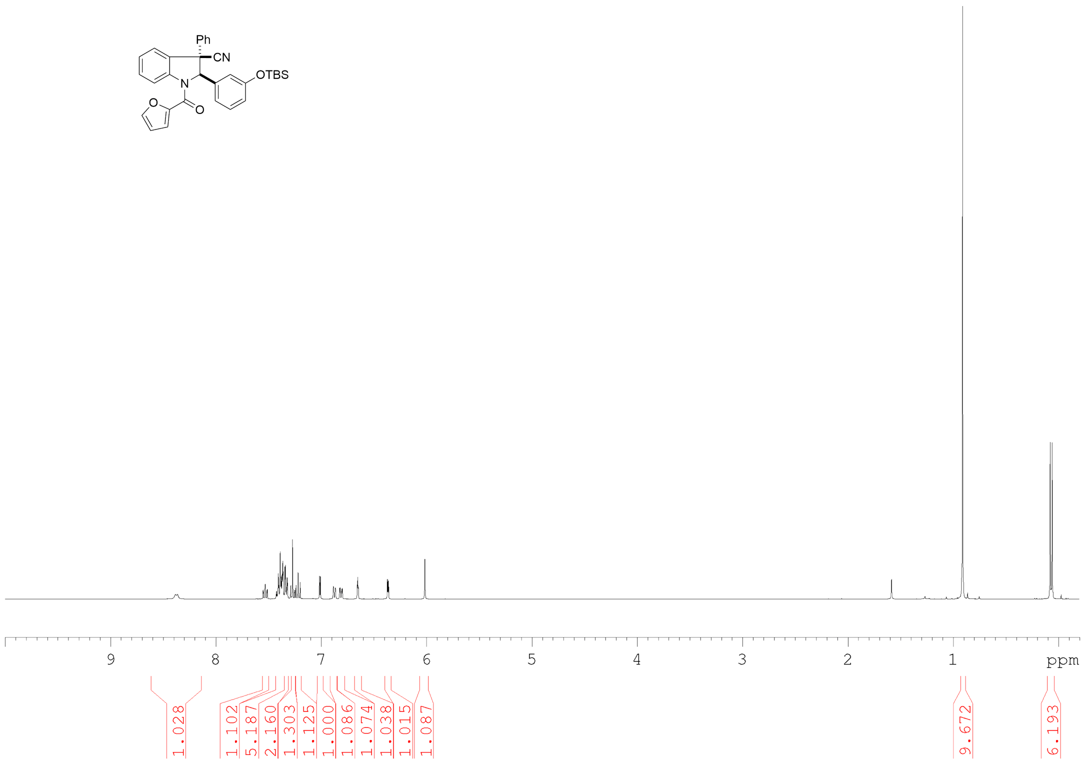
Ph
CN
N
OTBS
O
O
9
8
7
6
5
4
3
2
1
ppm
1.028
1.102
5.187
2.160
1.303
1.125
1.000
1.086
1.074
1.038
1.015
1.087
9.672
6.193

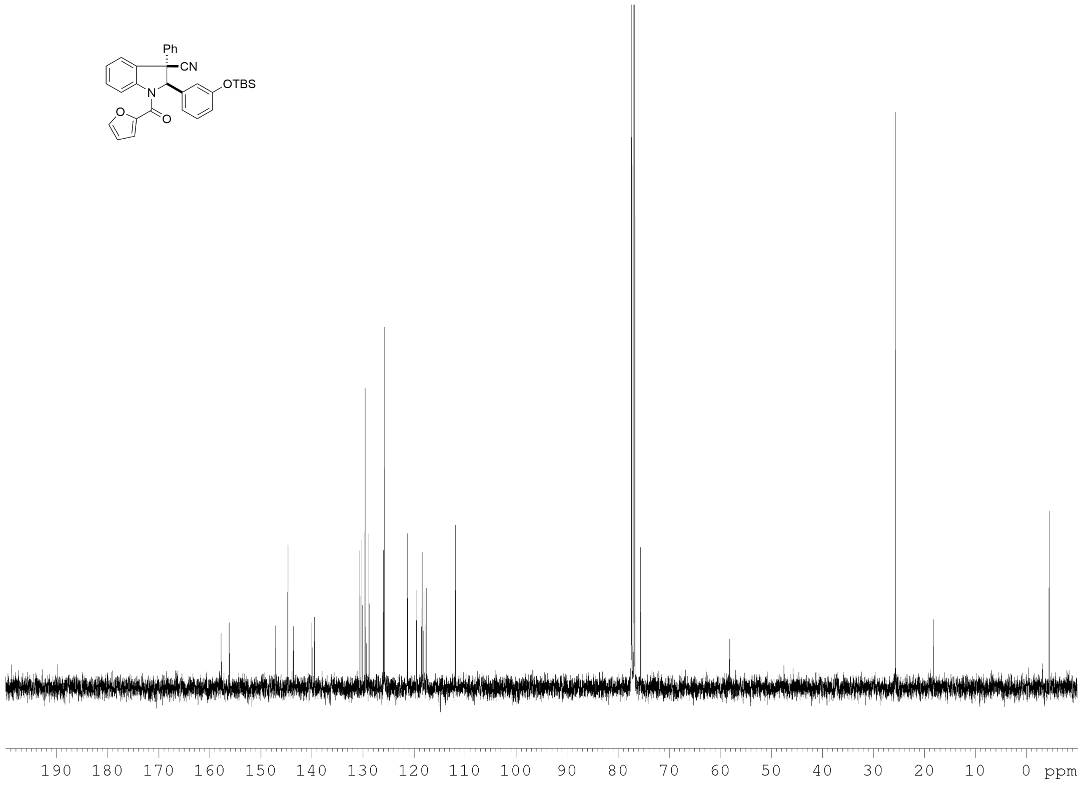
Ph
CN
N
OTBS
O
O
190
180
170
160
150
140
130
120
110
100
90
80
70
60
50
40
30
20
10
0
ppm

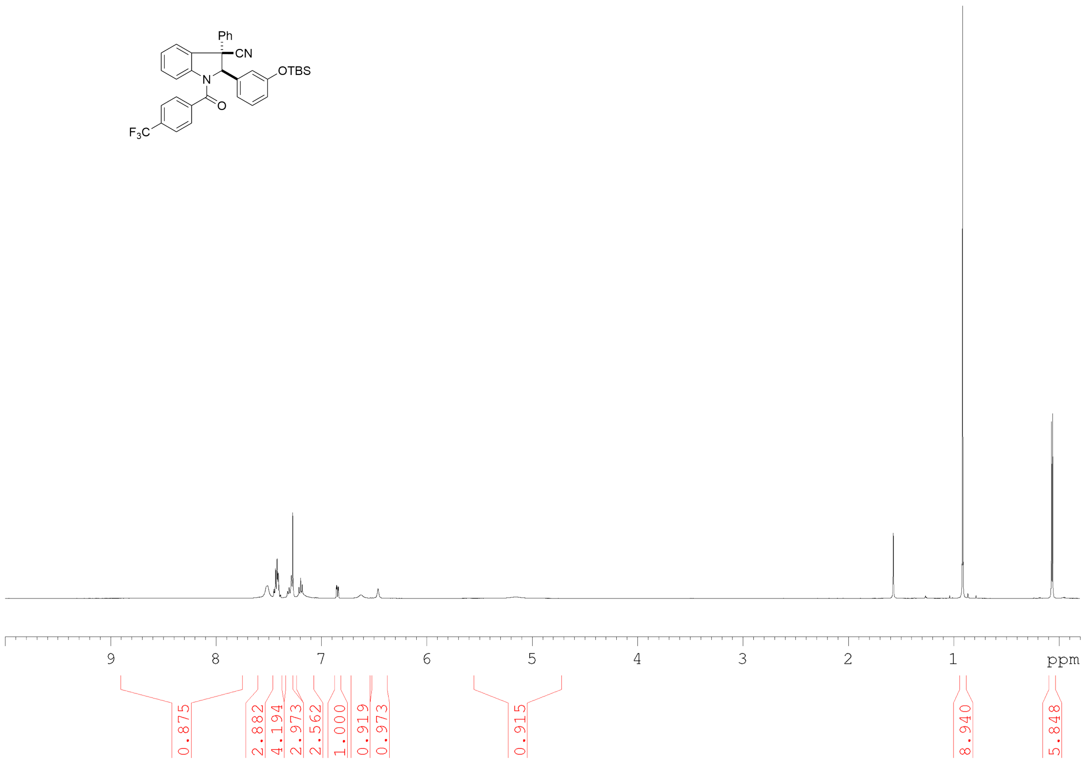
Ph
CN
OTBS
N
O
F3C
9 8 7 6 5 4 3 2 1 ppm
0.875
2.882
4.194
2.973
2.562
1.000
0.919
0.973
0.915
8.940
5.848

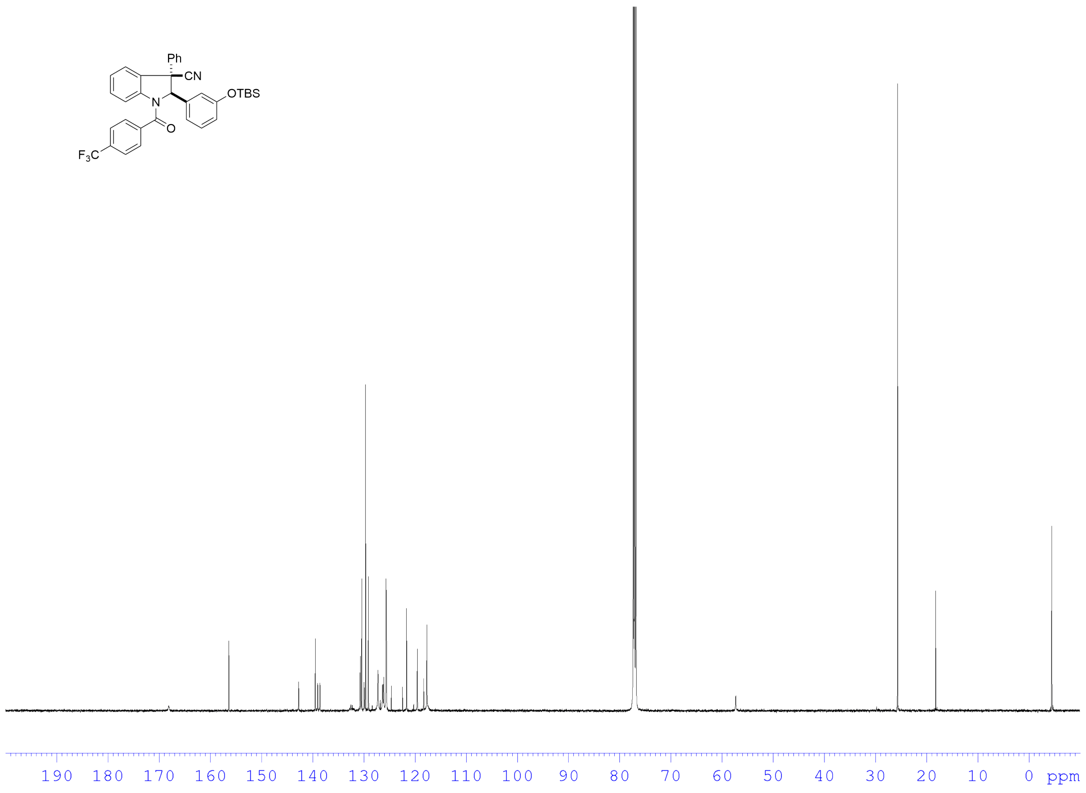
Ph
CN
OTBS
N
O
F3C
190 180 170 160 150 140 130 120 110 100 90 80 70 60 50 40 30 20 10 0 ppm

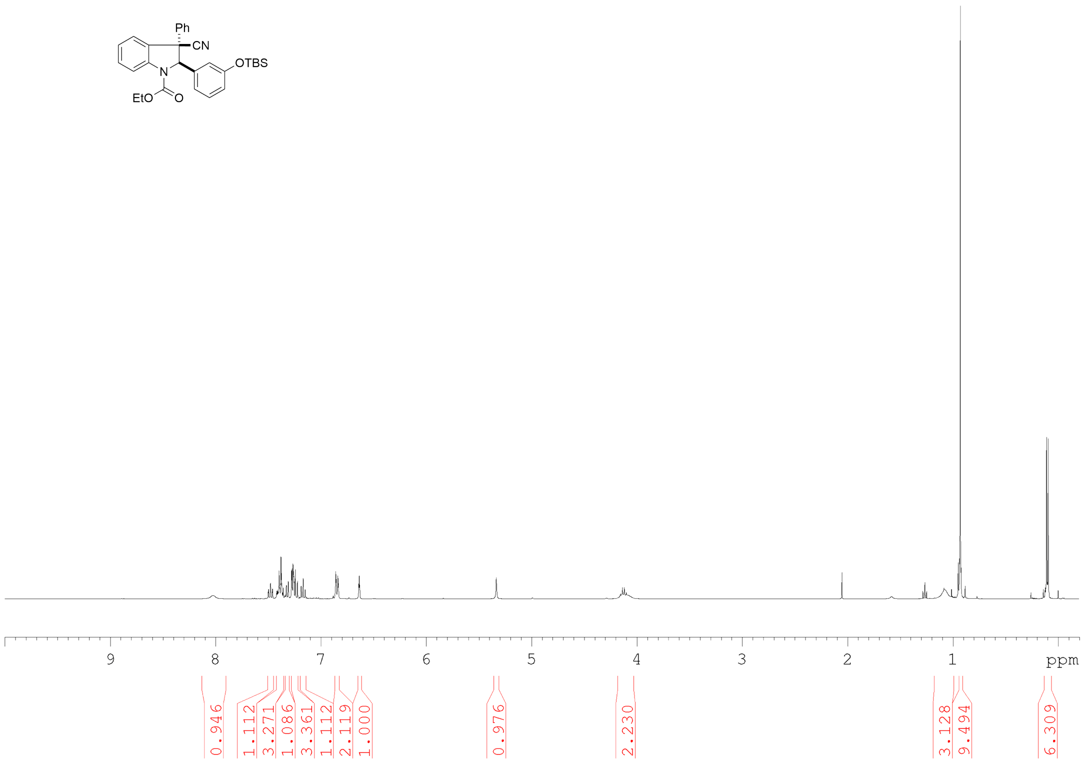

Ph
CN
N
OTBS
EtO
O
9
8
7
6
5
4
3
2
1
ppm
0.946
1.112
3.271
1.086
3.361
1.112
2.119
1.000
0.976
2.230
3.128
9.494
6.309

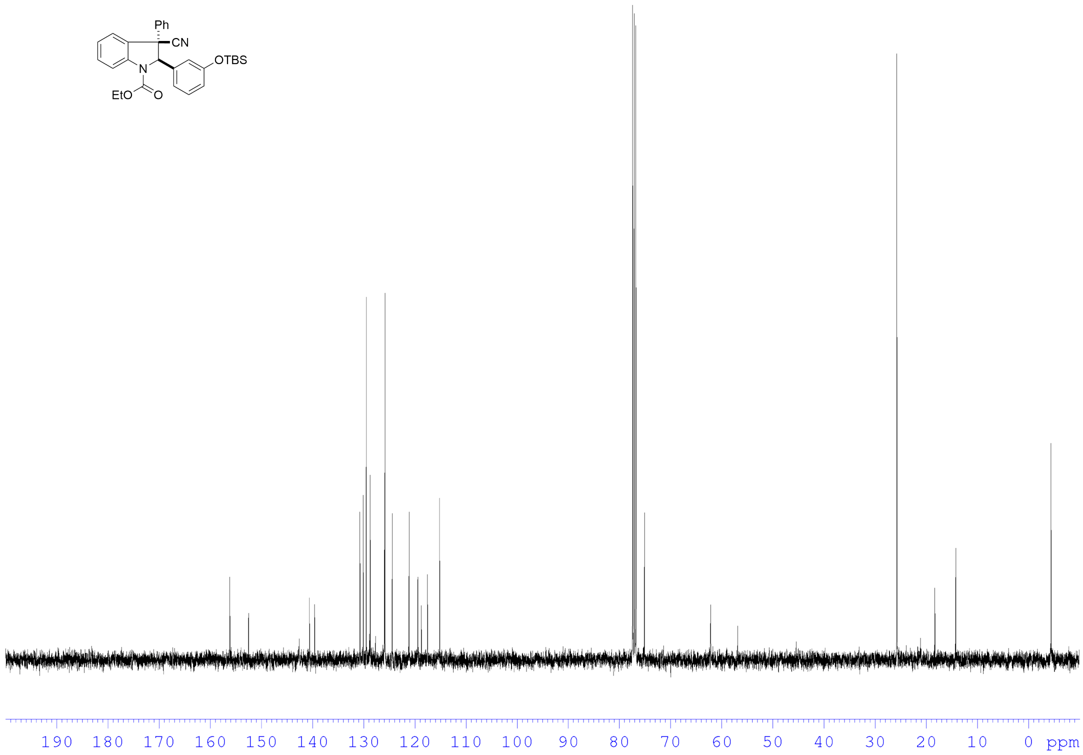

Ph
CN
N
OTBS
EtO
O
190
180
170
160
150
140
130
120
110
100
90
80
70
60
50
40
30
20
10
0
ppm

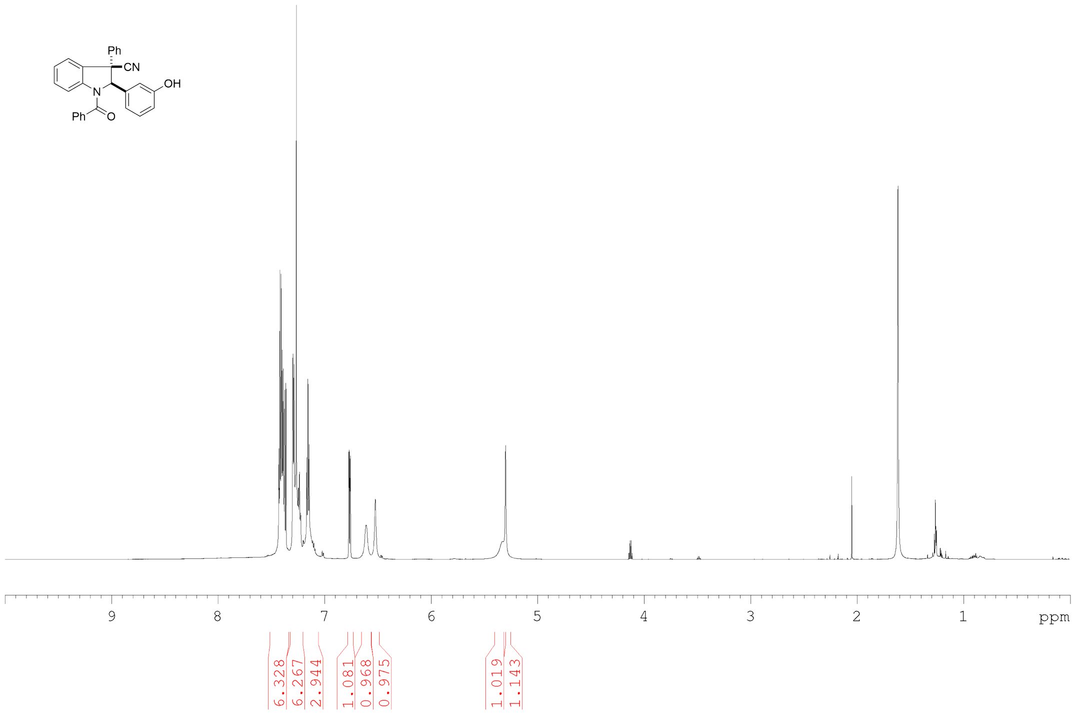
Ph
CN
N
OH
Ph
O
9
8
7
6
5
4
3
2
1
ppm
6.328
6.267
2.944
1.081
0.968
0.975
1.019
1.143

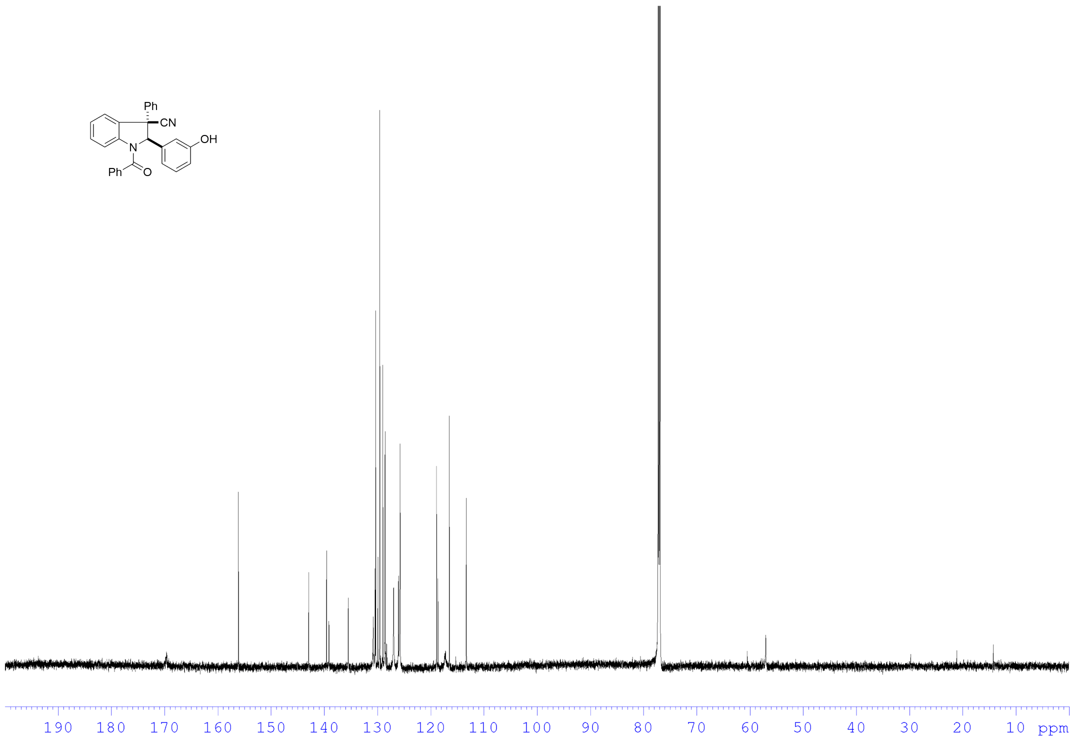
Ph
CN
N
OH
Ph
O
190
180
170
160
150
140
130
120
110
100
90
80
70
60
50
40
30
20
10
ppm

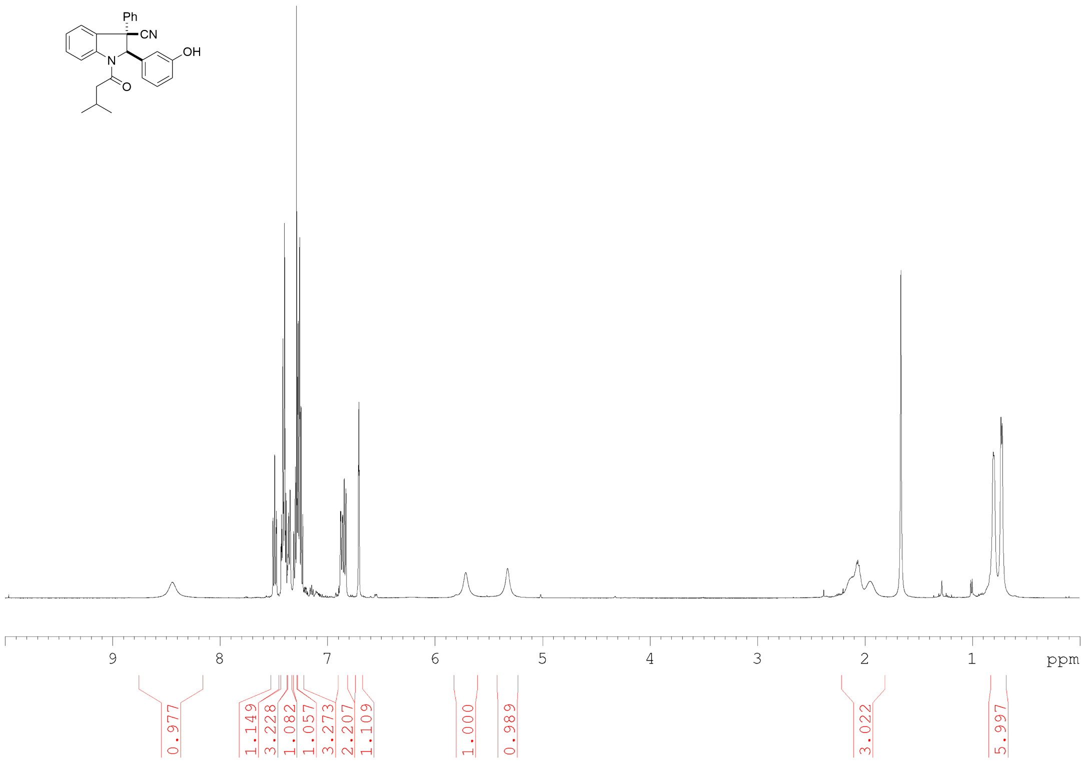
Ph
CN
OH
N
O
9 8 7 6 5 4 3 2 1 ppm
0.977
1.149
3.228
1.082
1.057
3.273
2.207
1.109
1.000
0.989
3.022
5.997

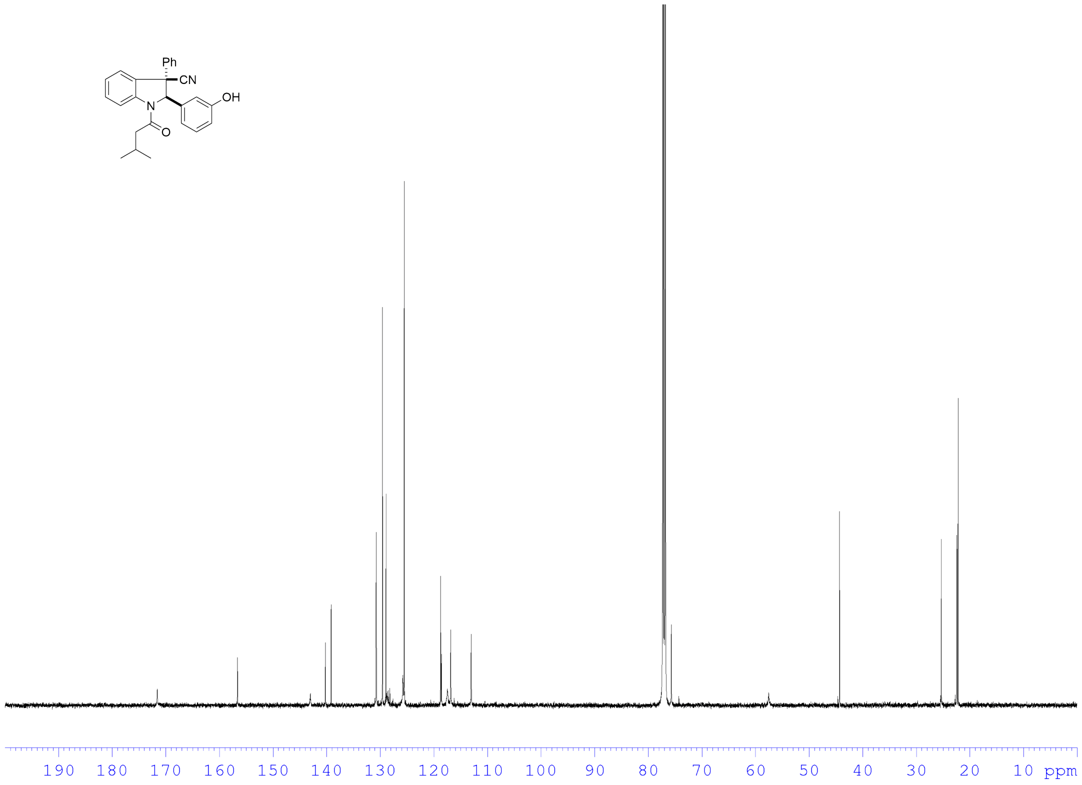
Ph
CN
OH
N
O
190 180 170 160 150 140 130 120 110 100 90 80 70 60 50 40 30 20 10 ppm

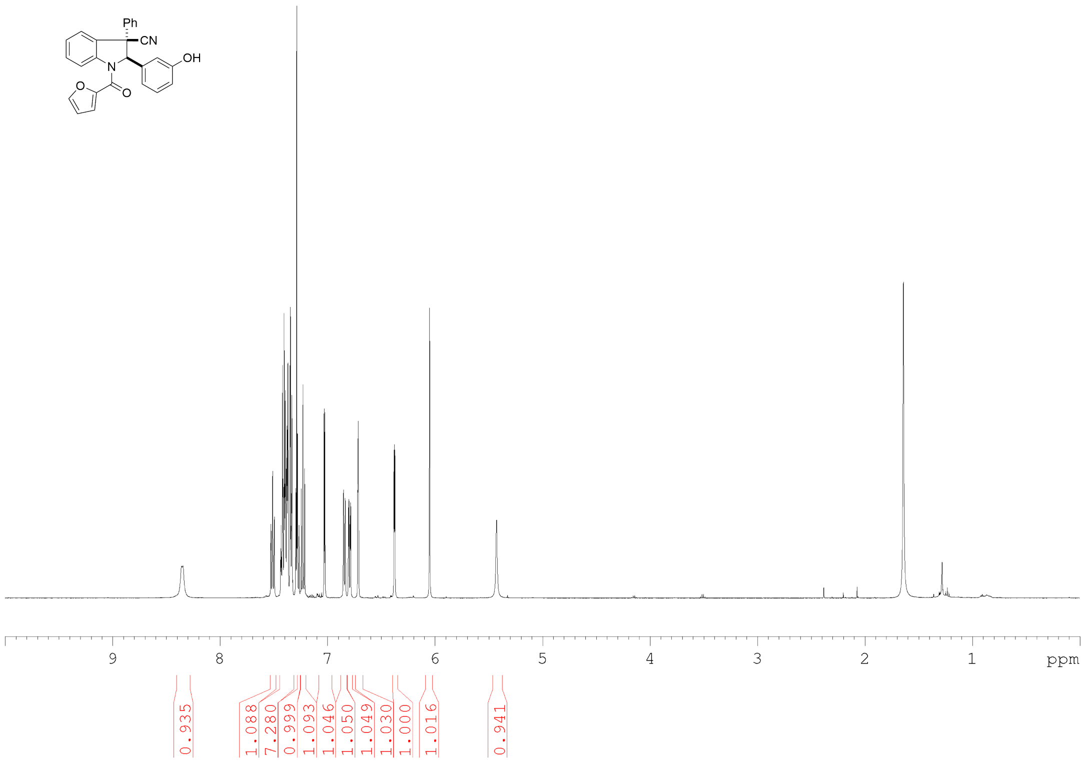
Ph
CN
OH
N
O
O
9
8
7
6
5
4
3
2
1
ppm
0.935
1.088
7.280
0.999
1.093
1.046
1.050
1.049
1.030
1.000
1.016
0.941

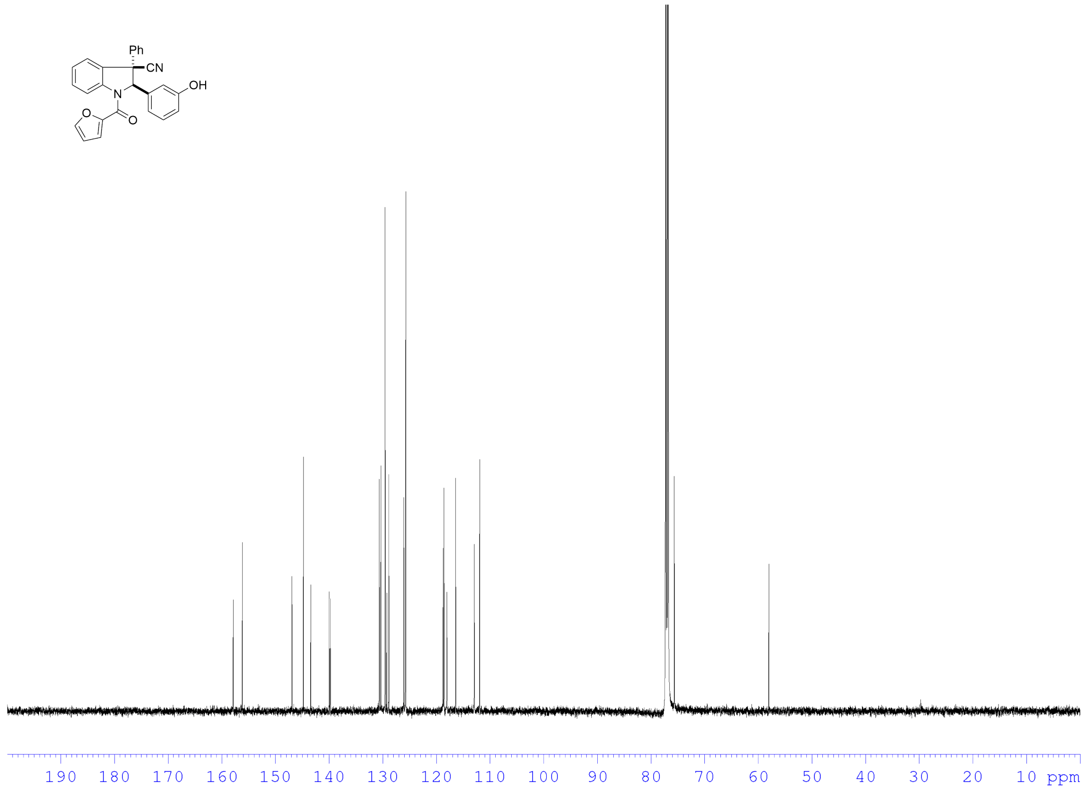
Ph
CN
OH
N
O
O
190
180
170
160
150
140
130
120
110
100
90
80
70
60
50
40
30
20
10 ppm

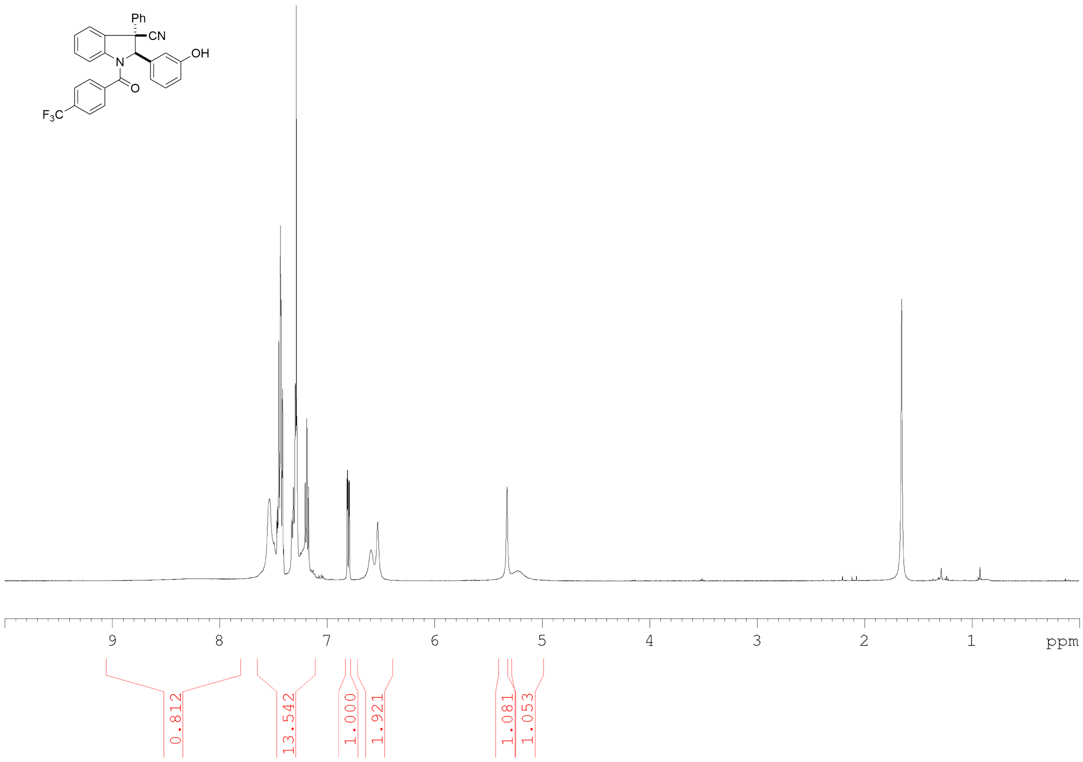
Ph
CN
N
OH
O
F3C
9
8
7
6
5
4
3
2
1
ppm
0.812
13.542
1.000
1.921
1.081
1.053

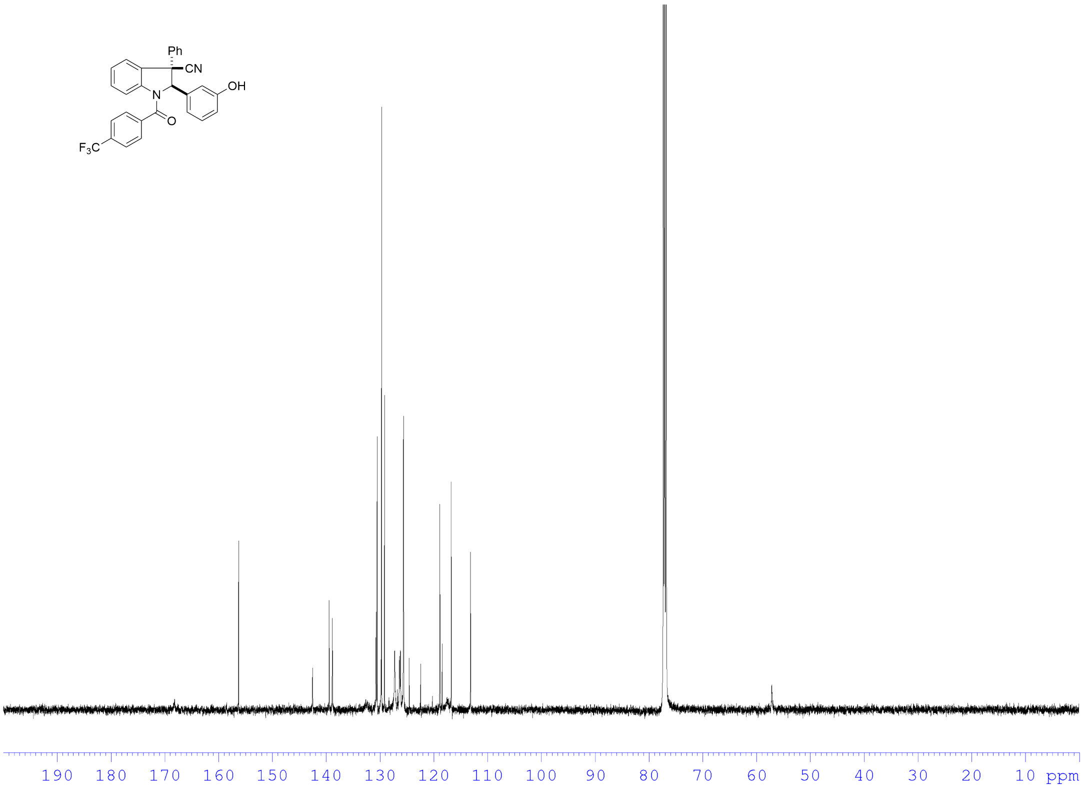
Ph
CN
N
OH
O
F3C
190
180
170
160
150
140
130
120
110
100
90
80
70
60
50
40
30
20
10
ppm

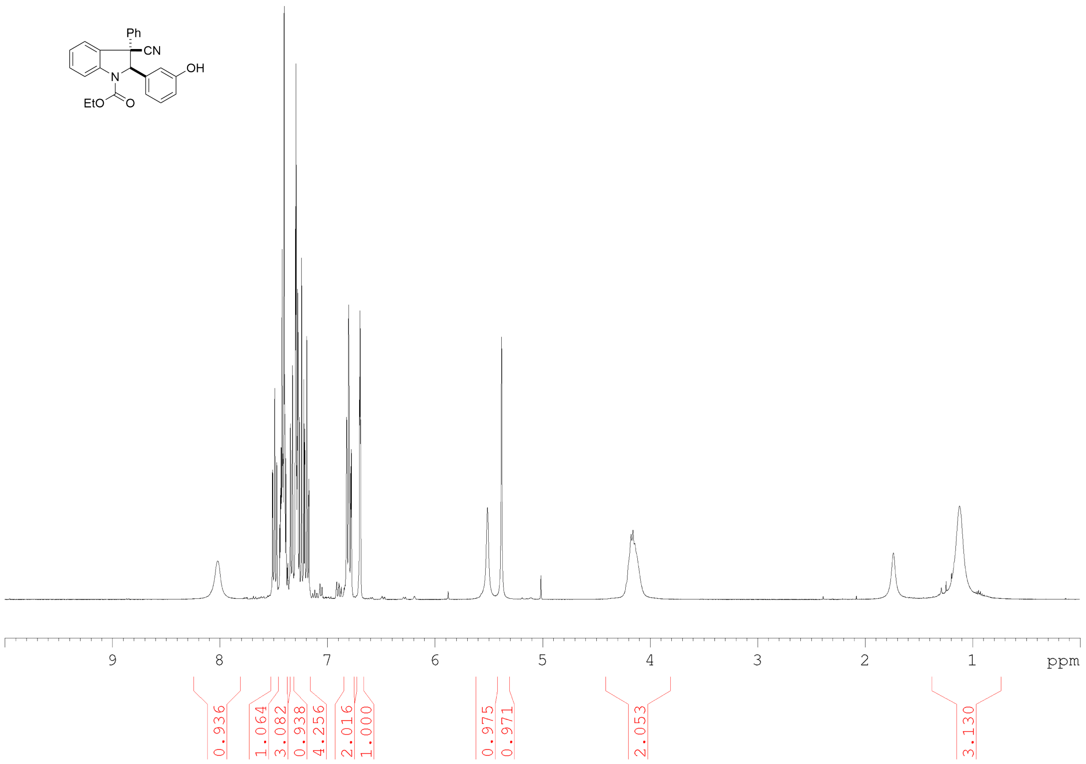

Ph
CN
N
OH
EtO
O
9
8
7
6
5
4
3
2
1
ppm
0.936
1.064
3.082
0.938
4.256
2.016
1.000
0.975
0.971
2.053
3.130

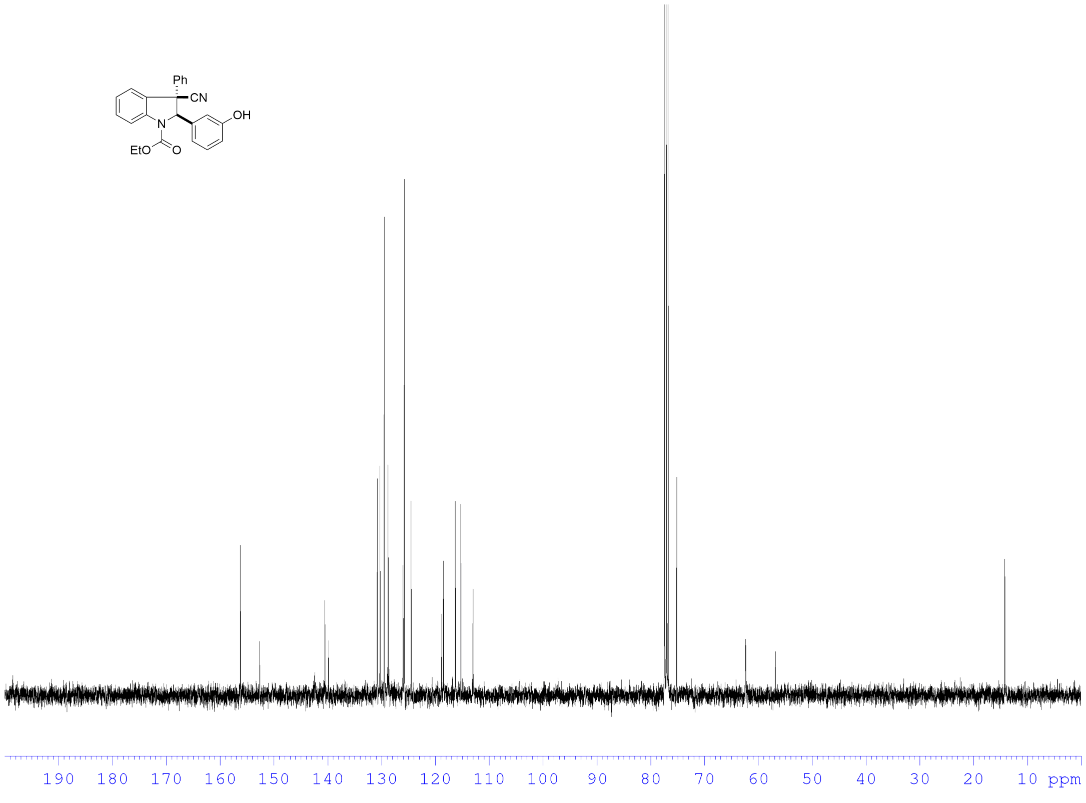

Ph
CN
N
OH
EtO
O
190
180
170
160
150
140
130
120
110
100
90
80
70
60
50
40
30
20
10
ppm

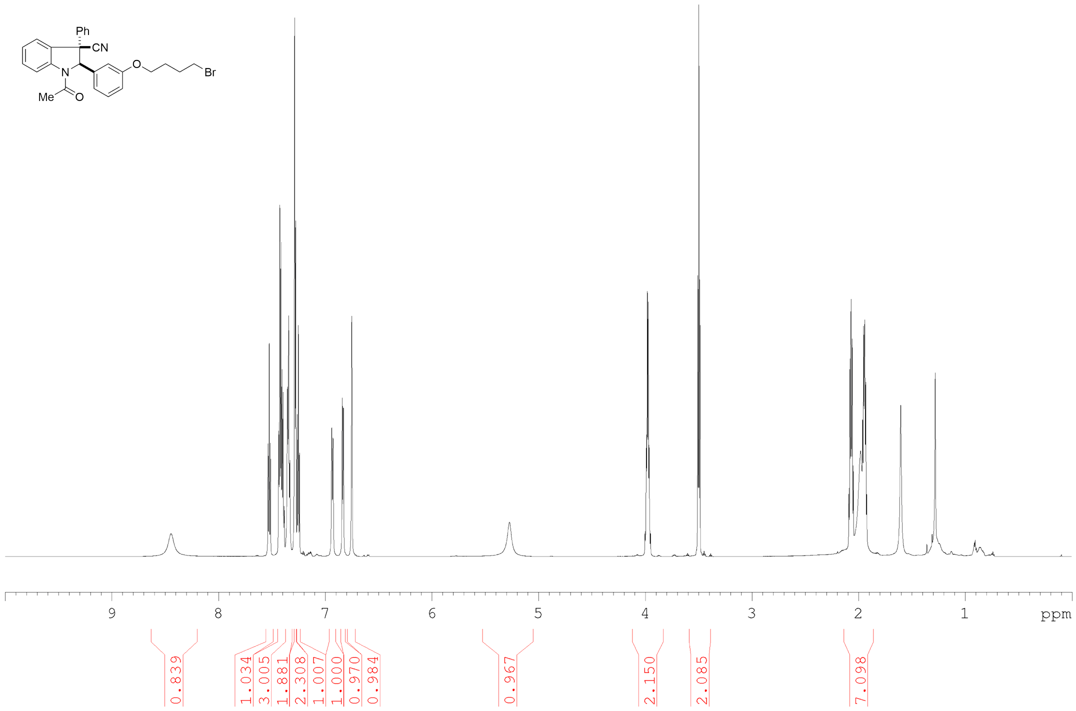
Ph
CN
N
O
Br
Me
O
9 8 7 6 5 4 3 2 1 ppm
0.839
1.034
3.005
1.881
2.308
1.007
1.000
0.970
0.984
0.967
2.150
2.085
7.098

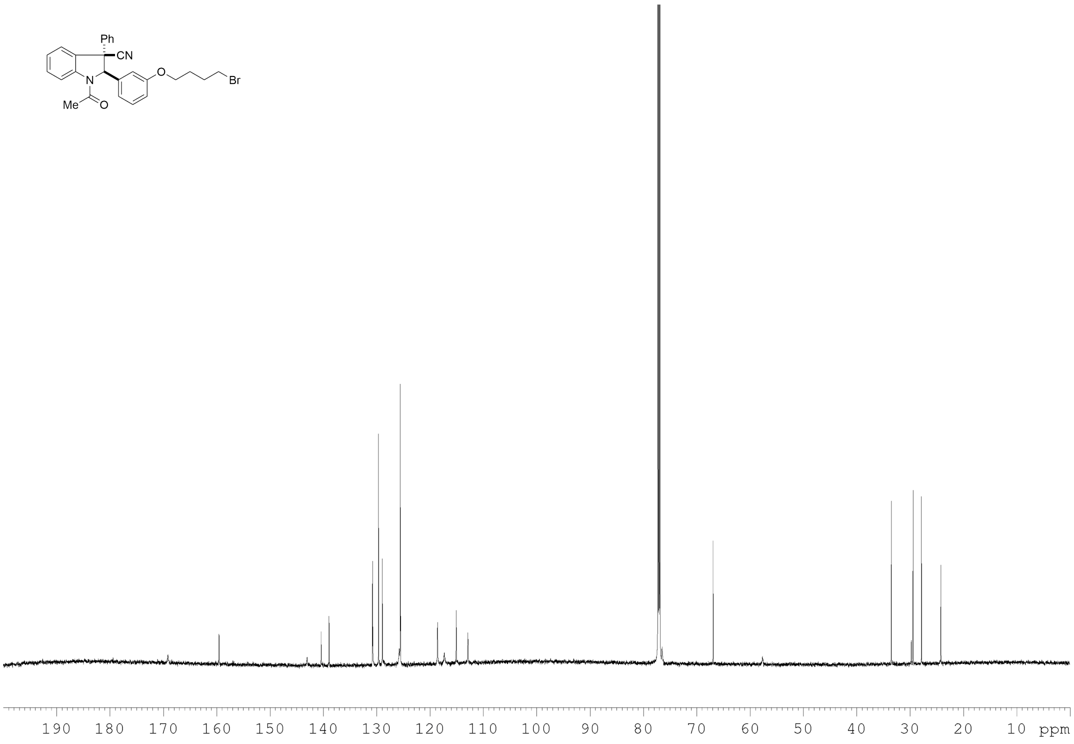
Ph
CN
N
O
Br
Me
O
190 180 170 160 150 140 130 120 110 100 90 80 70 60 50 40 30 20 10 ppm

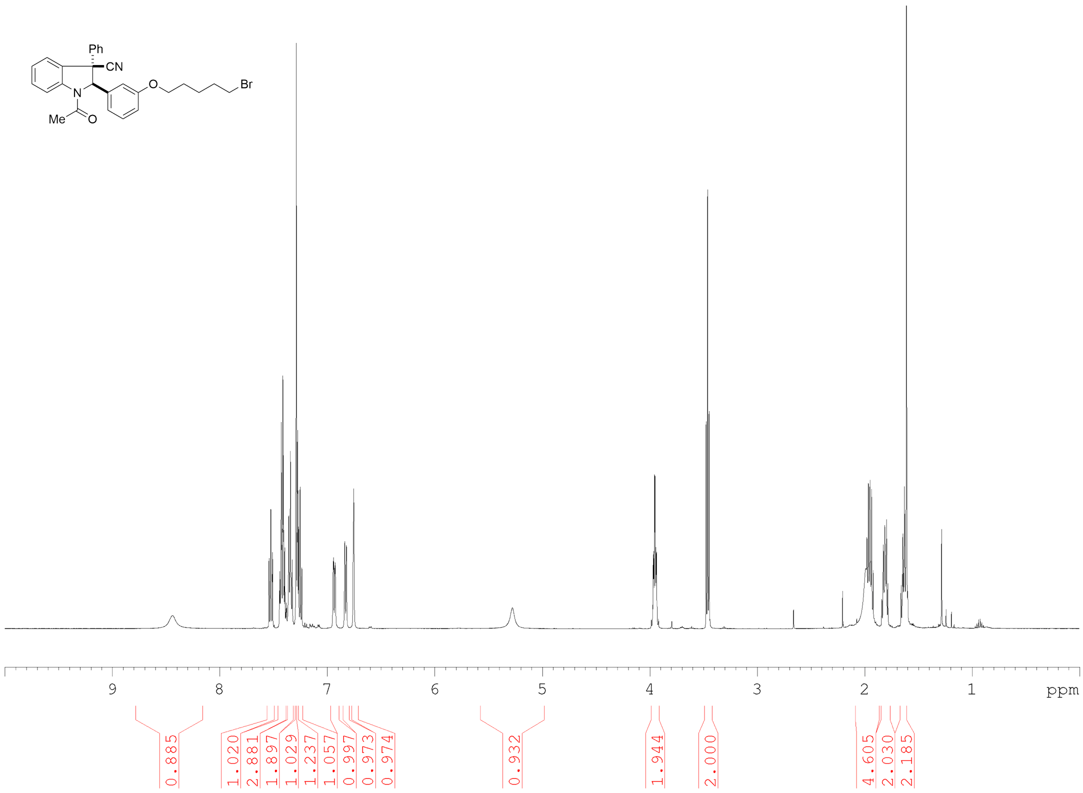
Ph
CN
N
O
Br
Me
O
9
8
7
6
5
4
3
2
1
ppm
0.885
1.020
2.881
1.897
1.029
1.237
1.057
0.997
0.973
0.974
0.932
1.944
2.000
4.605
2.030
2.185

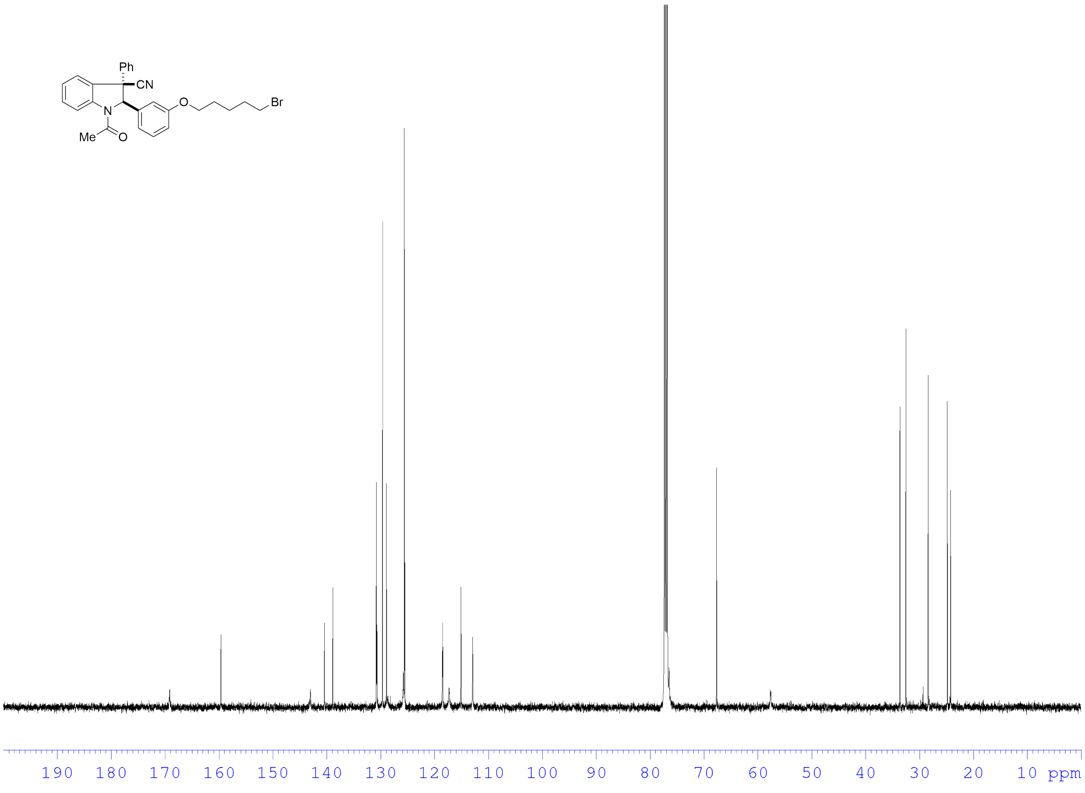
Ph
CN
N
O
Br
Me
O
190
180
170
160
150
140
130
120
110
100
90
80
70
60
50
40
30
20
10
ppm

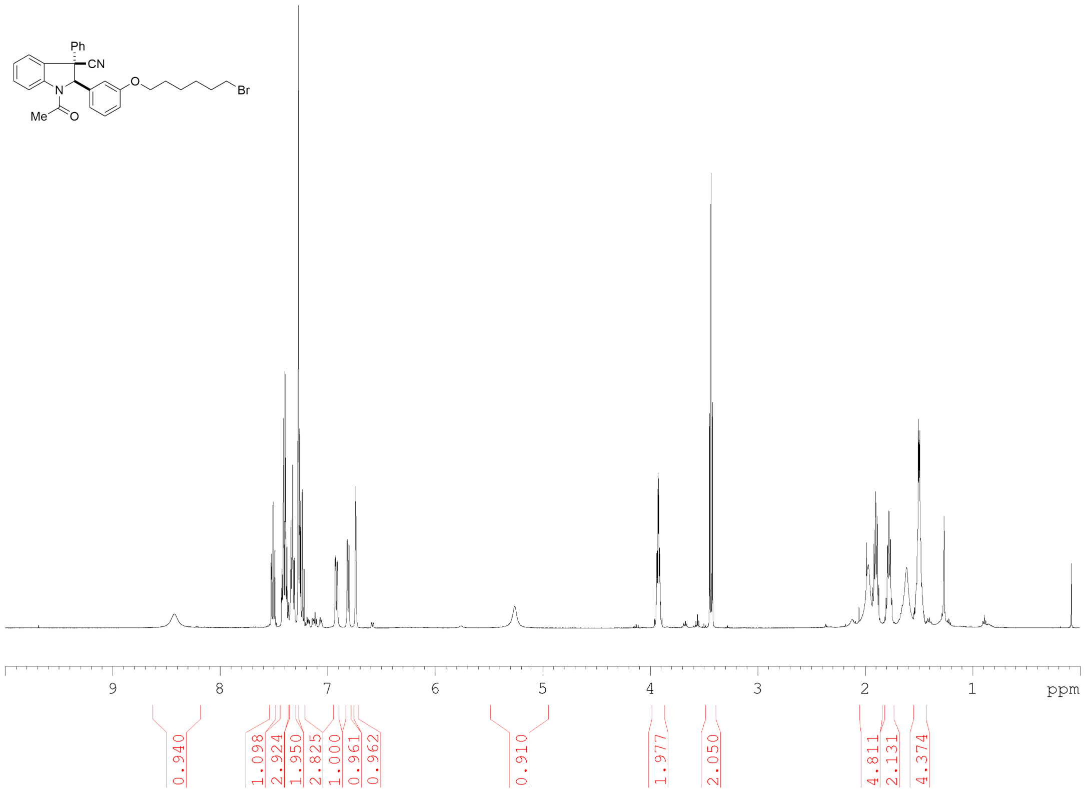
Ph
CN
N
O
Br
Me
O
9 8 7 6 5 4 3 2 1 ppm
0.940
1.098
2.924
1.950
2.825
1.000
0.961
0.962
0.910
1.977
2.050
4.811
2.131
4.374

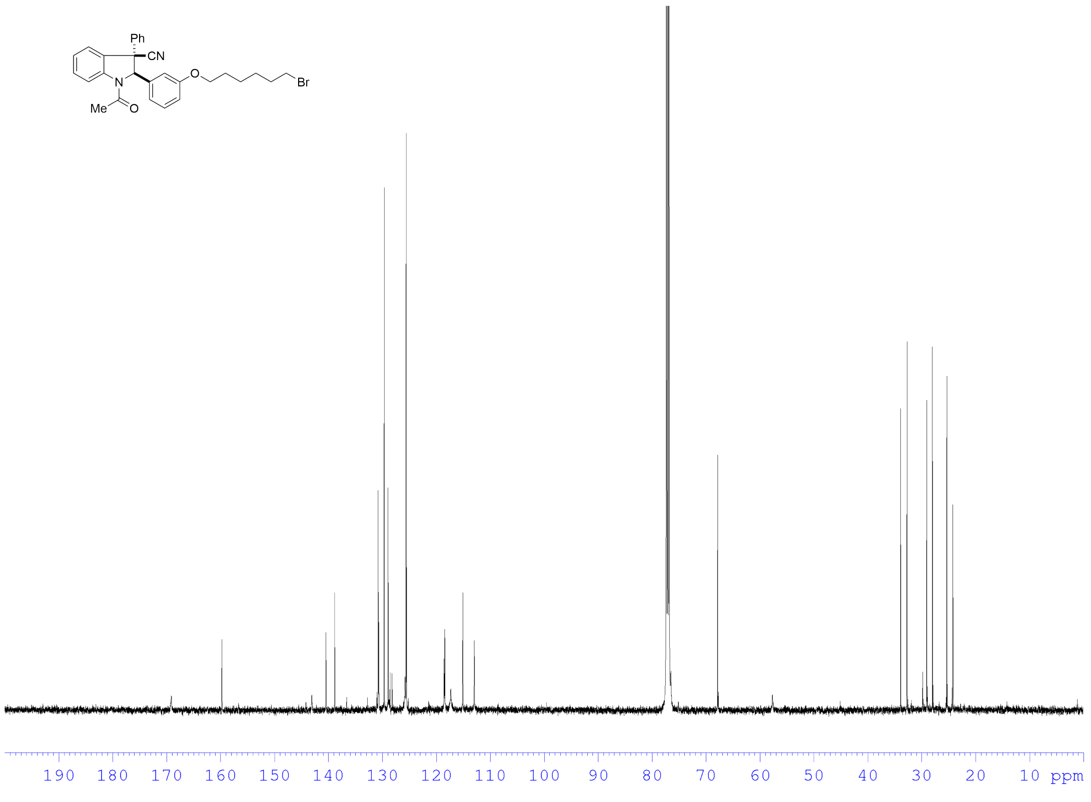
Ph
CN
N
O
Br
Me
O
190 180 170 160 150 140 130 120 110 100 90 80 70 60 50 40 30 20 10 ppm

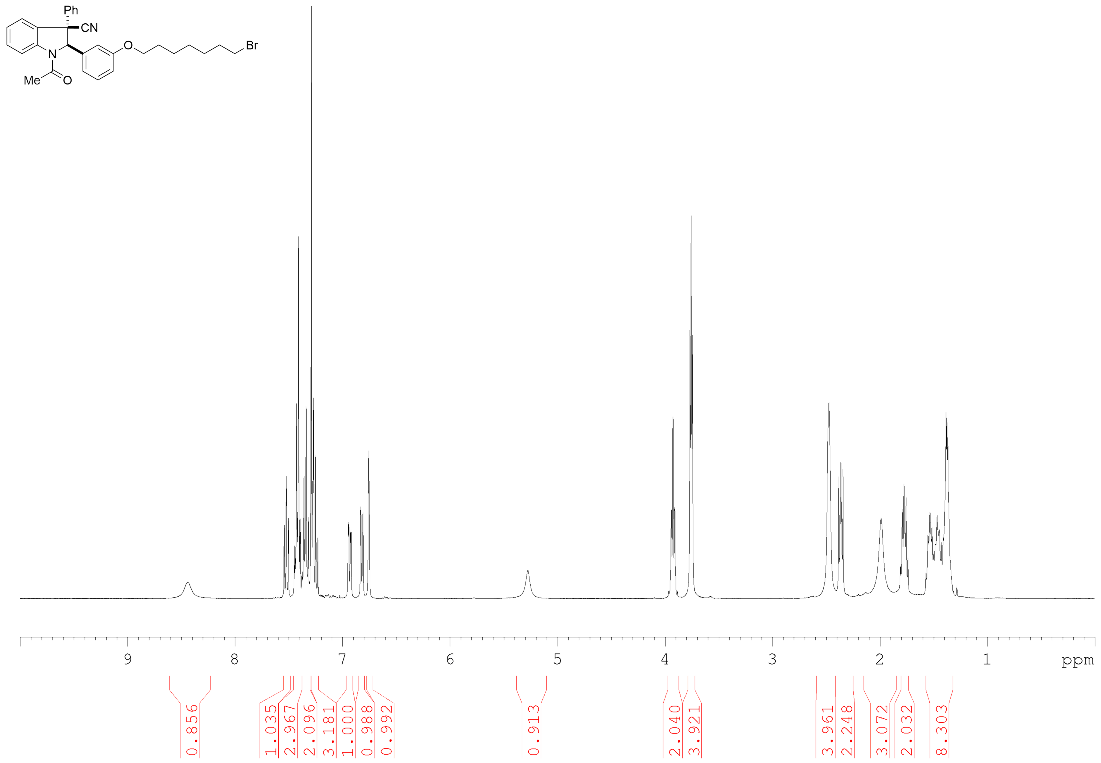
Ph
CN
N
O
Br
Me
O
9 8 7 6 5 4 3 2 1 ppm
0.856
1.035
2.967
2.096
3.181
1.000
0.988
0.992
0.913
2.040
3.921
3.961
2.248
3.072
2.032
8.303

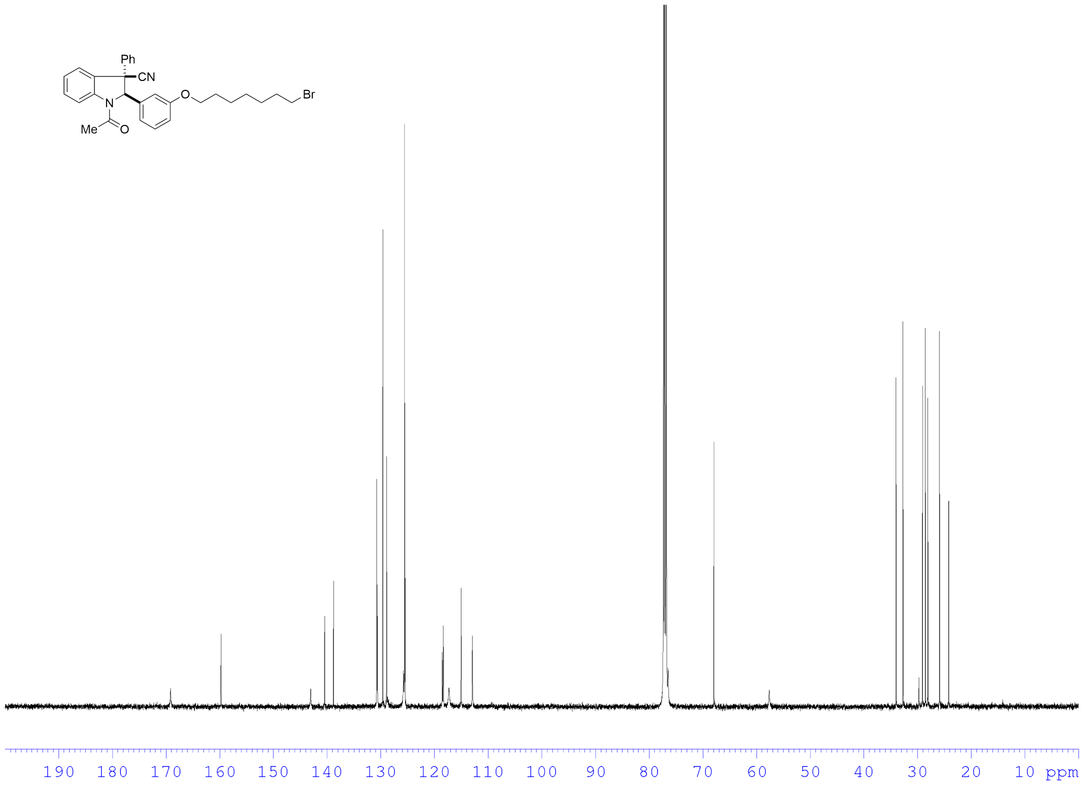
Ph
CN
N
O
Br
Me
O
190 180 170 160 150 140 130 120 110 100 90 80 70 60 50 40 30 20 10 ppm

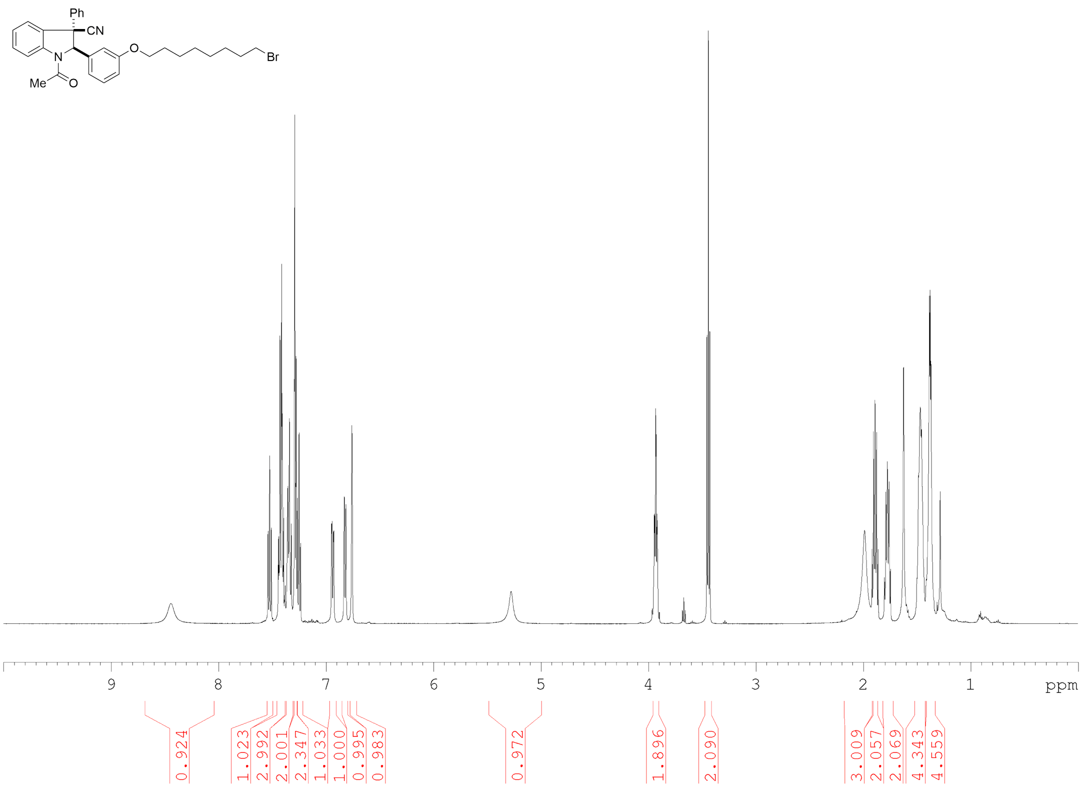

Ph
CN
N
O
Br
Me
O
9
8
7
6
5
4
3
2
1
ppm
0.924
1.023
2.992
2.001
2.347
1.033
1.000
0.995
0.983
0.972
1.896
2.090
3.009
2.057
2.069
4.343
4.559

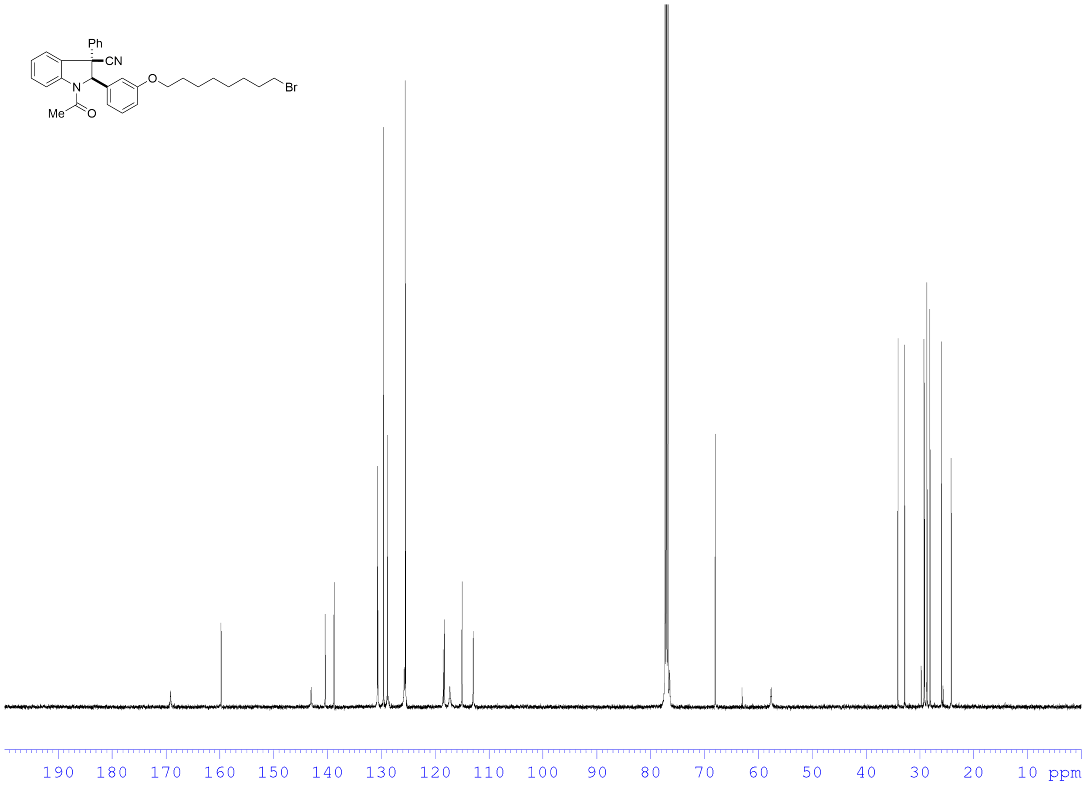

Ph
CN
N
O
Br
Me
O
190
180
170
160
150
140
130
120
110
100
90
80
70
60
50
40
30
20
10
ppm

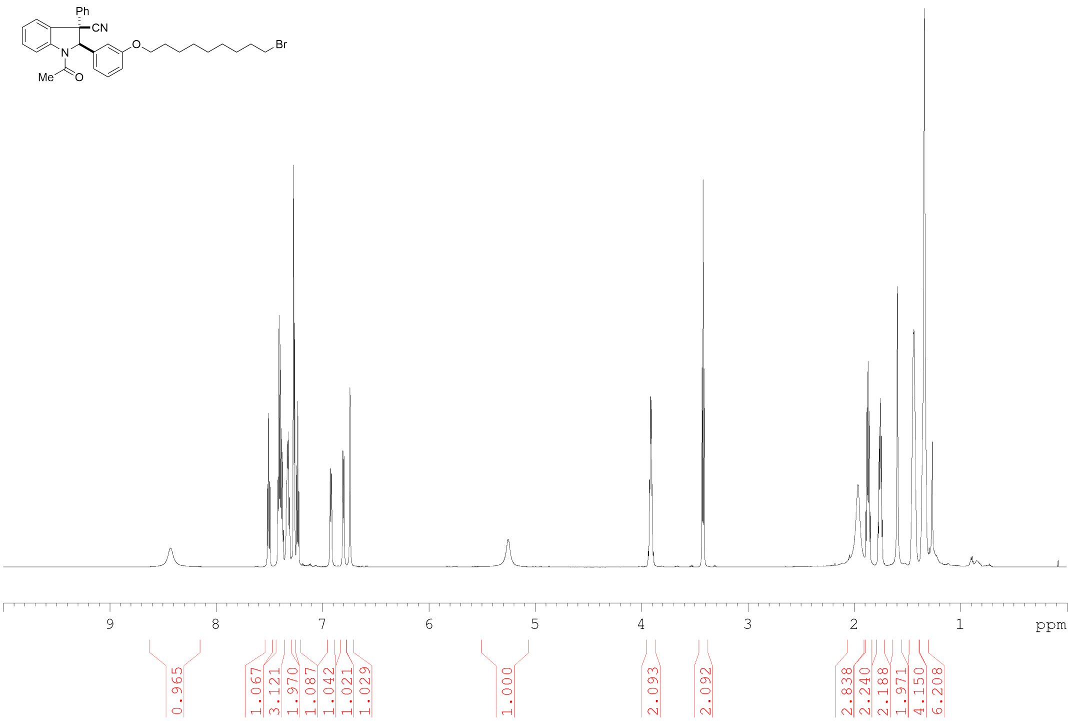
Ph
CN
N
O
Br
Me
O
9
8
7
6
5
4
3
2
1
ppm
0.965
1.067
3.121
1.970
1.087
1.042
1.021
1.029
1.000
2.093
2.092
2.838
2.240
2.188
1.971
4.150
6.208

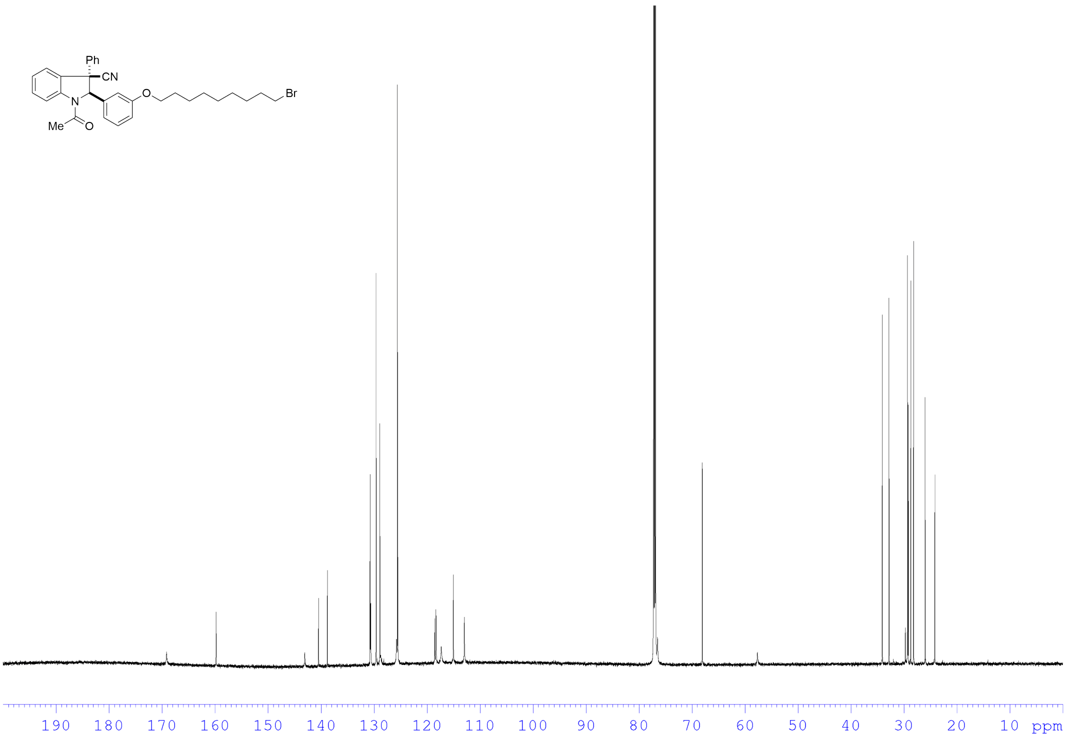
Ph
CN
N
O
Br
Me
O
190
180
170
160
150
140
130
120
110
100
90
80
70
60
50
40
30
20
10
ppm

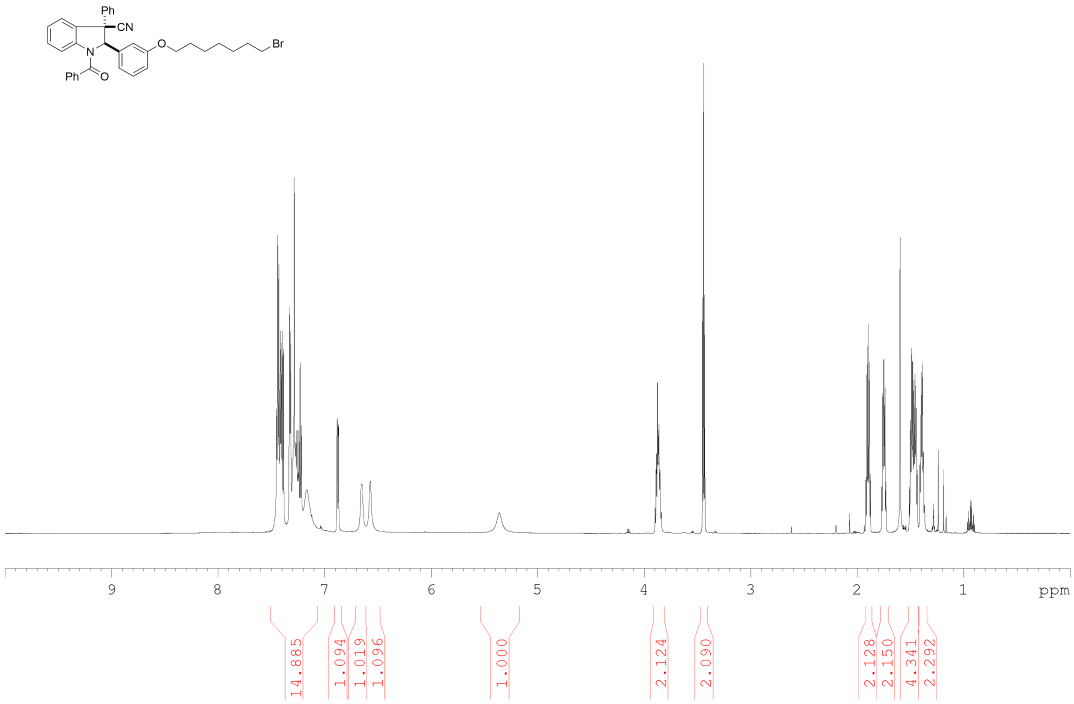

Ph
CN
N
O
Br
Ph
O
9
8
7
6
5
4
3
2
1
ppm
14.885
1.094
1.019
1.096
1.000
2.124
2.090
2.128
2.150
4.341
2.292

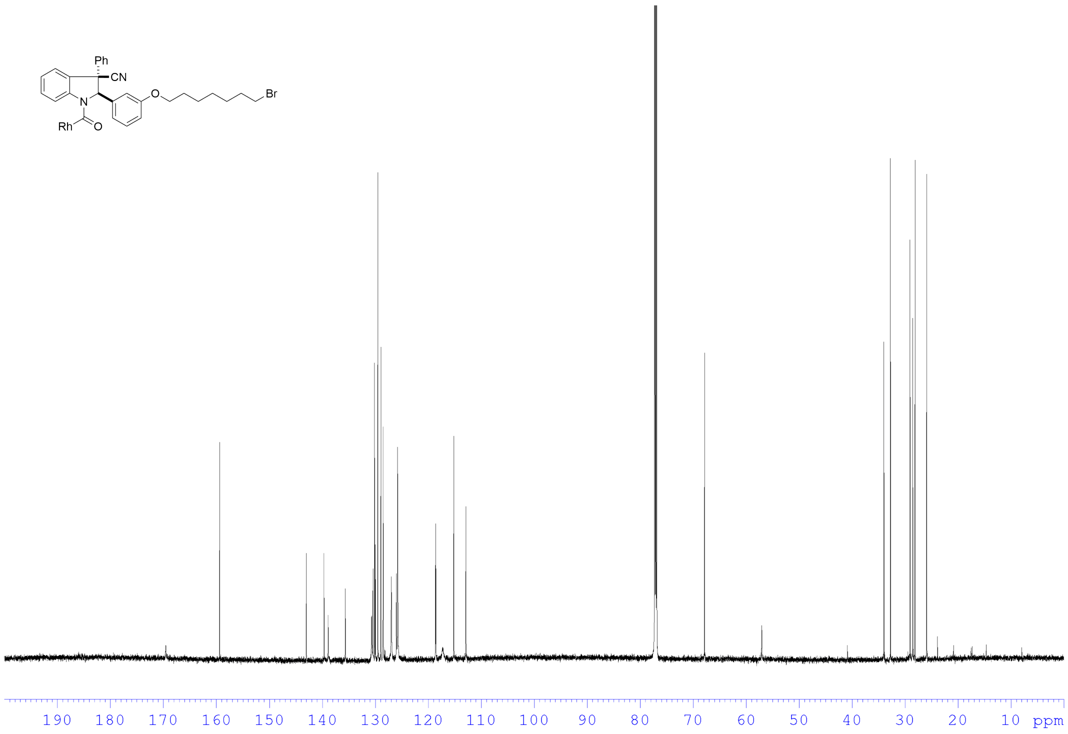

Ph
CN
N
O
Br
Rh
O
190
180
170
160
150
140
130
120
110
100
90
80
70
60
50
40
30
20
10
ppm

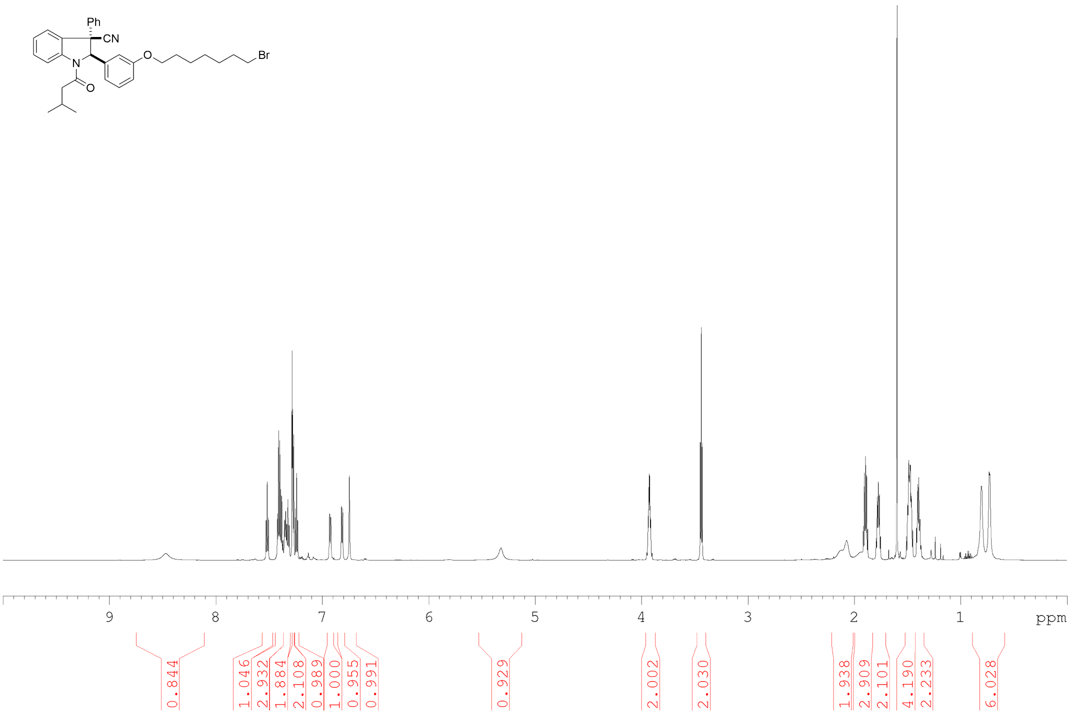

Ph
CN
N
O
O
Br
9
8
7
6
5
4
3
2
1
ppm
0.844
1.046
2.932
1.884
2.108
0.989
1.000
0.955
0.991
0.929
2.002
2.030
1.938
2.909
2.101
4.190
2.233
6.028

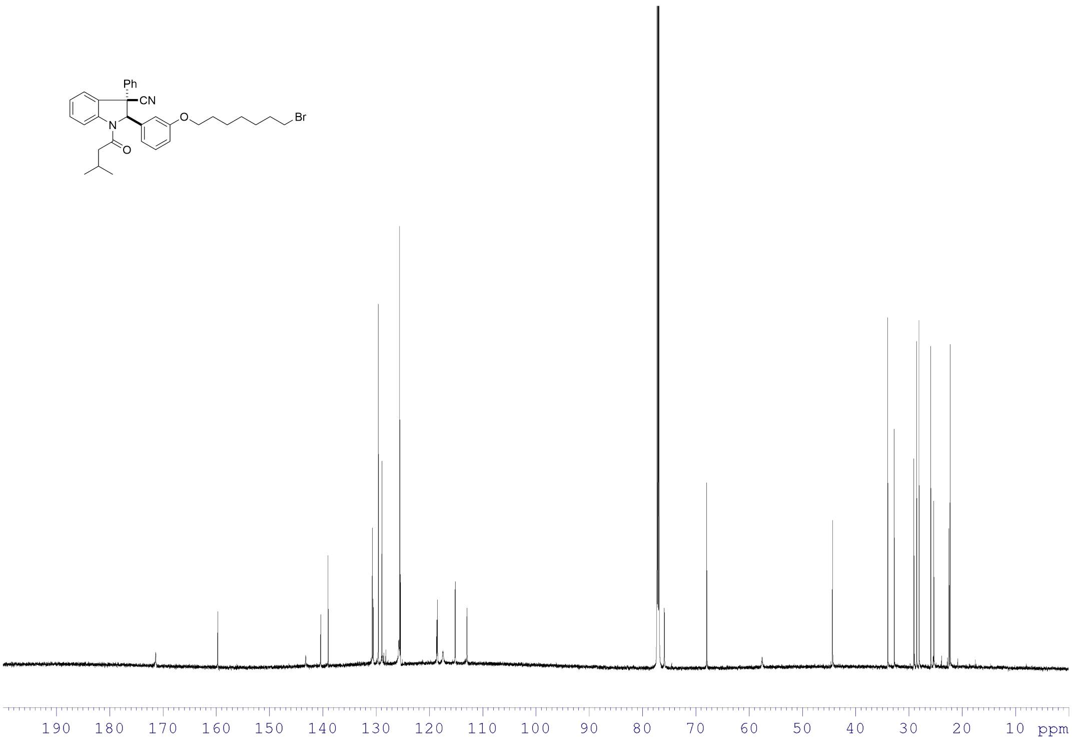

Ph
CN
N
O
O
Br
190
180
170
160
150
140
130
120
110
100
90
80
70
60
50
40
30
20
10
ppm

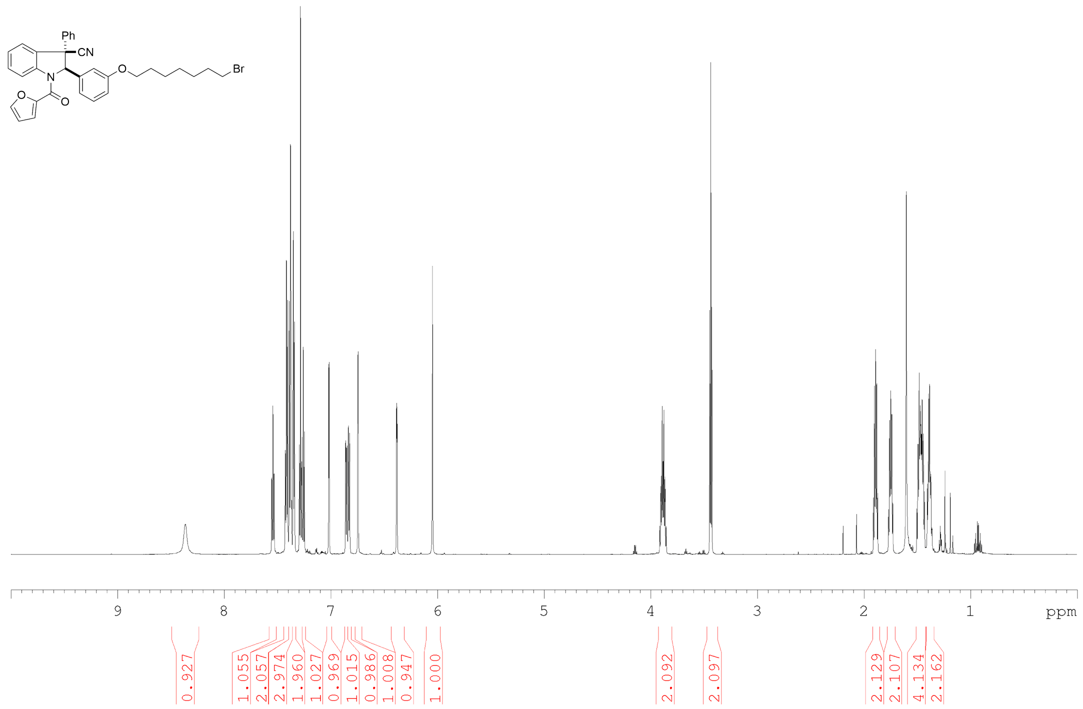
Ph
CN
N
O
O
O
Br
9
8
7
6
5
4
3
2
1
ppm
0.927
1.055
2.057
2.974
1.960
1.027
0.969
1.015
0.986
1.008
0.947
1.000
2.092
2.097
2.129
2.107
4.134
2.162

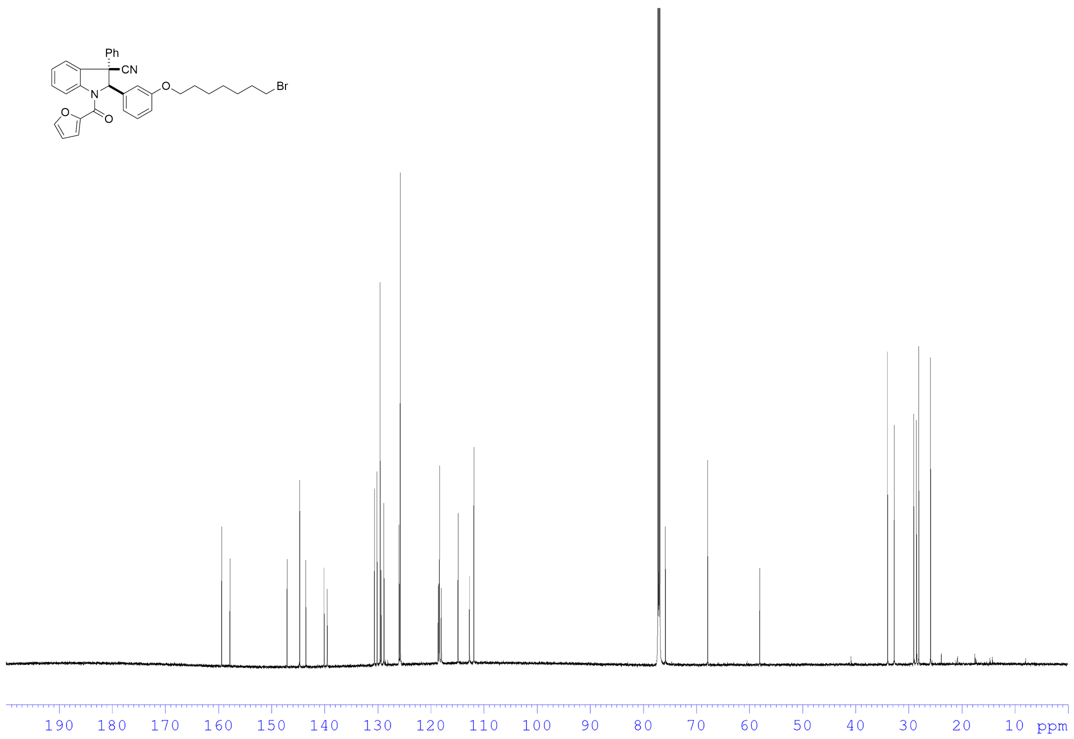
Ph
CN
N
O
O
O
Br
190
180
170
160
150
140
130
120
110
100
90
80
70
60
50
40
30
20
10
ppm

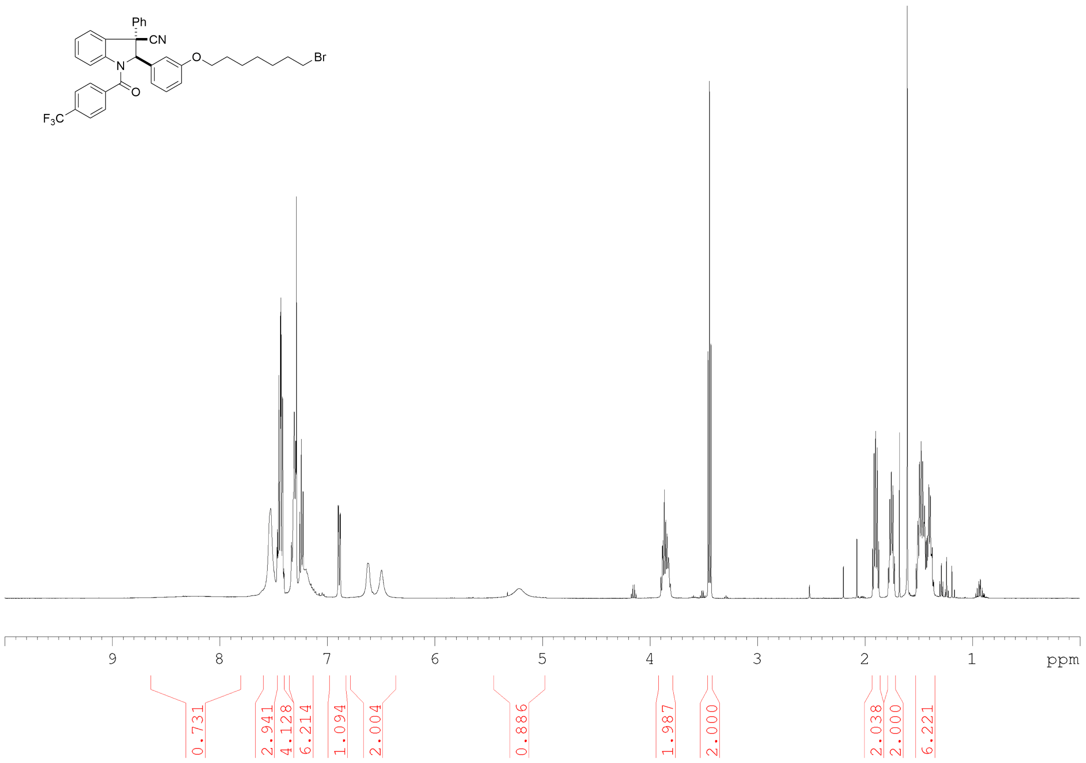
Ph
CN
O
Br
N
O
F3C
9 8 7 6 5 4 3 2 1 ppm
0.731
2.941
4.128
6.214
1.094
2.004
0.886
1.987
2.000
2.038
2.000
6.221

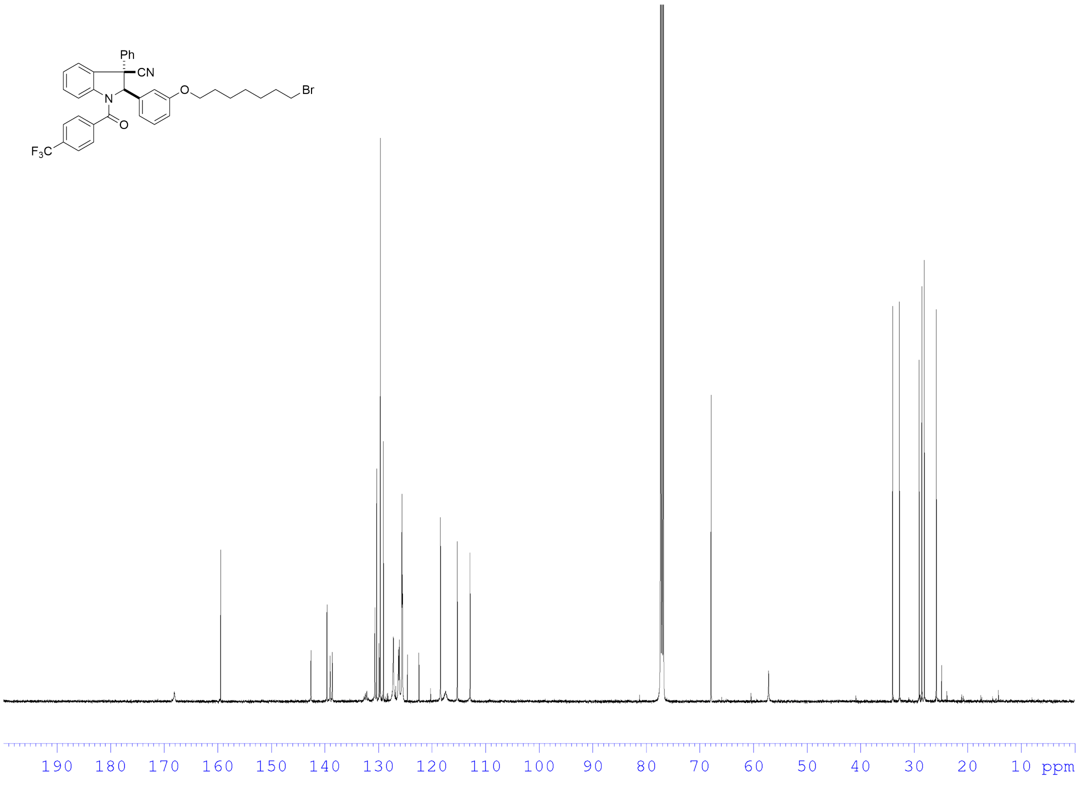
Ph
CN
O
Br
N
O
F3C
190 180 170 160 150 140 130 120 110 100 90 80 70 60 50 40 30 20 10 ppm

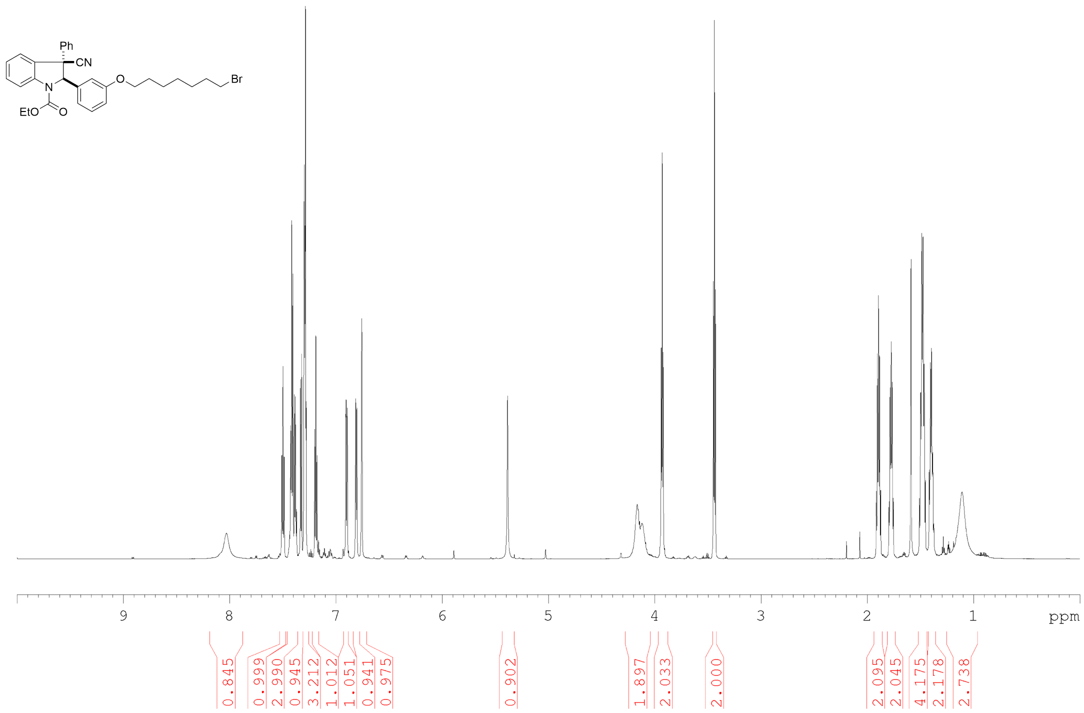
Ph
CN
N
O
Br
EtO
O
9
8
7
6
5
4
3
2
1
ppm
0.845
0.999
2.990
0.945
3.212
1.012
1.051
0.941
0.975
0.902
1.897
2.033
2.000
2.095
2.045
4.175
2.178
2.738

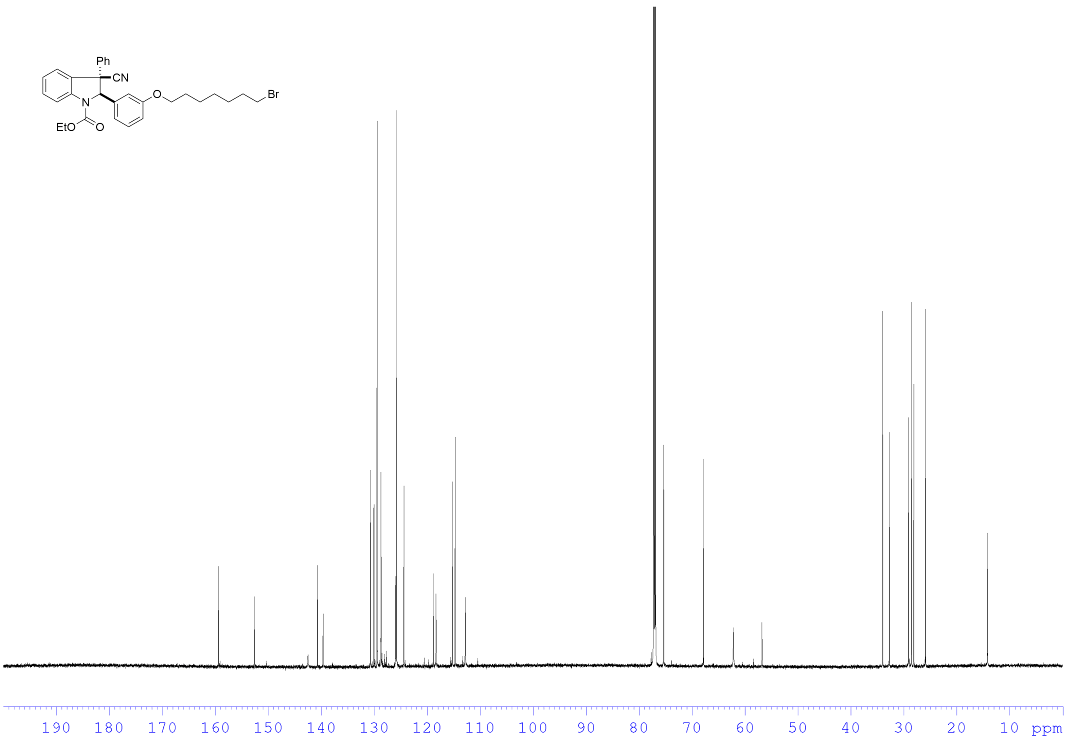
Ph
CN
N
O
Br
EtO
O
190
180
170
160
150
140
130
120
110
100
90
80
70
60
50
40
30
20
10
ppm

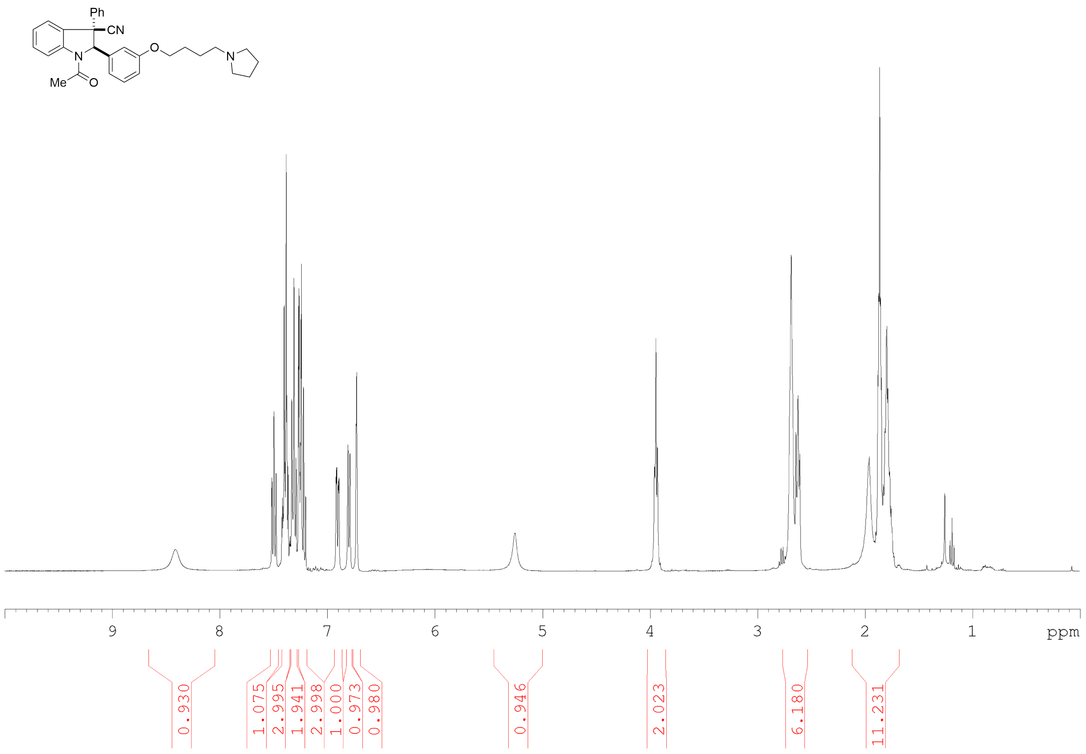
Ph
CN
N
O
N
Me
O
9 8 7 6 5 4 3 2 1 ppm
0.930
1.075
2.995
1.941
2.998
1.000
0.973
0.980
0.946
2.023
6.180
11.231

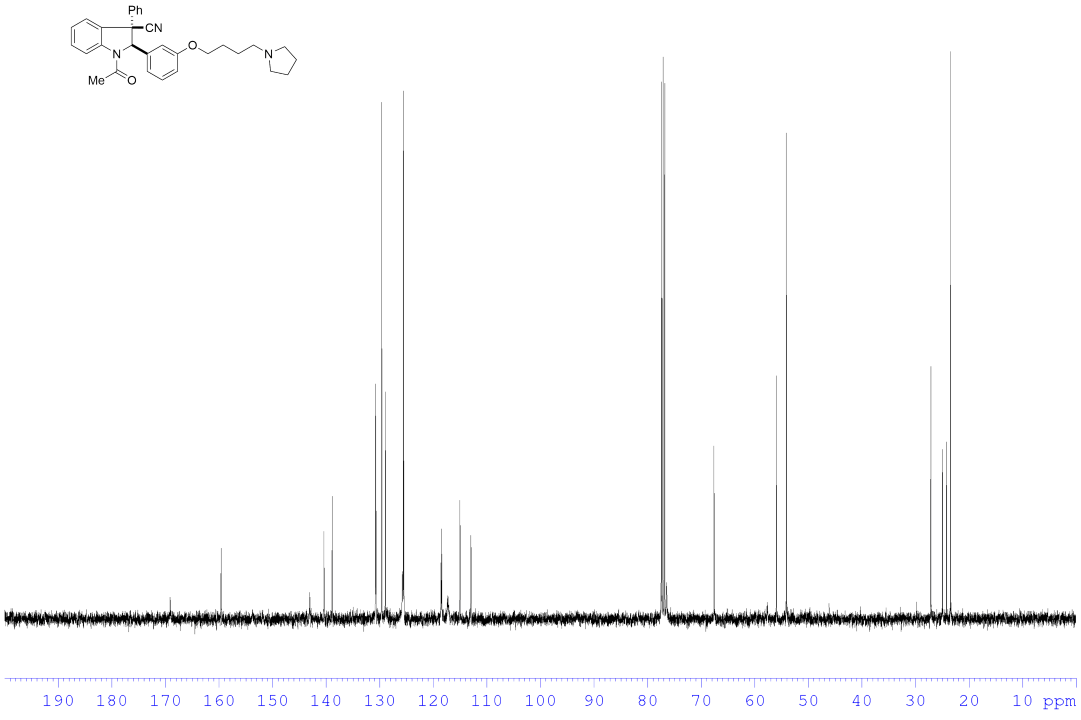
Ph
CN
N
O
N
Me
O
190 180 170 160 150 140 130 120 110 100 90 80 70 60 50 40 30 20 10 ppm

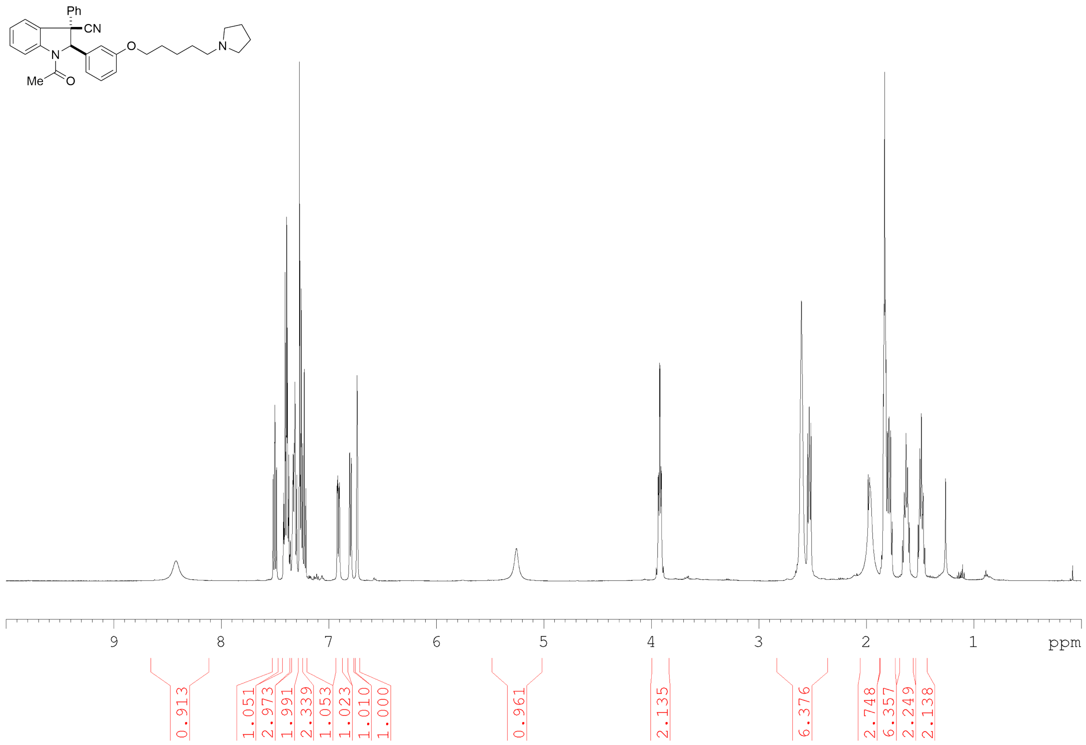

Ph
CN
N
O
N
Me
O
9
8
7
6
5
4
3
2
1
ppm
0.913
1.051
2.973
1.991
2.339
1.053
1.023
1.010
1.000
0.961
2.135
6.376
2.748
6.357
2.249
2.138

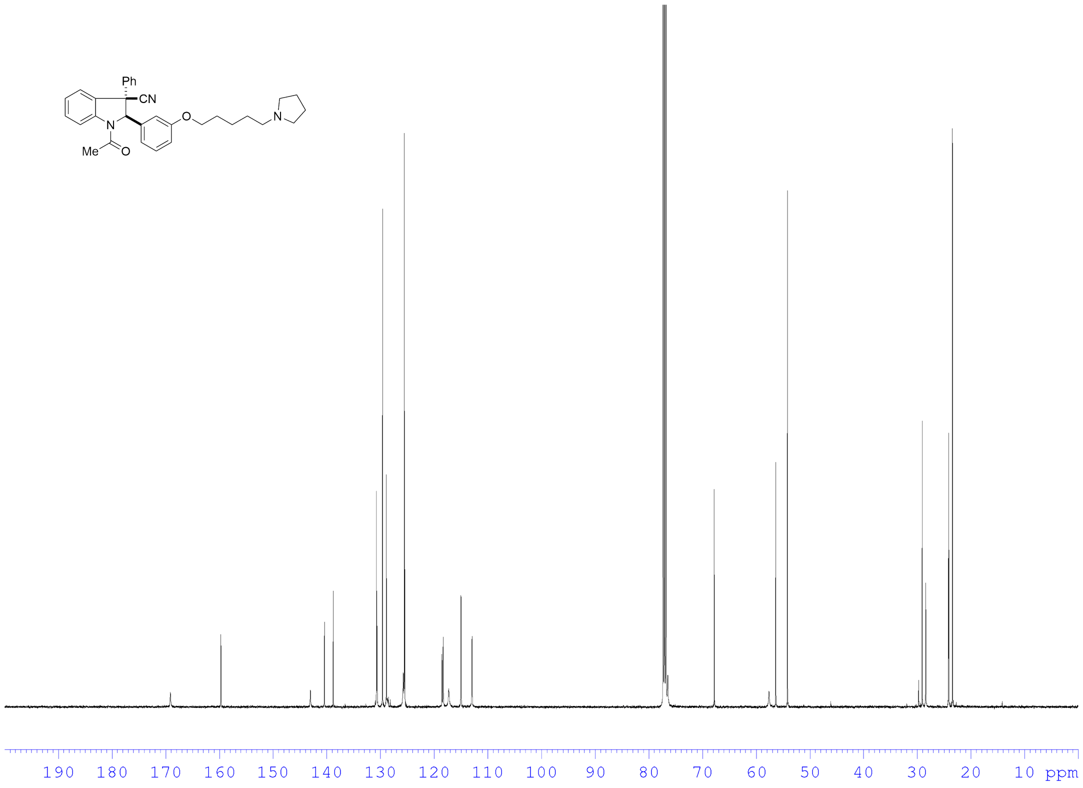

Ph
CN
N
O
N
Me
O
190
180
170
160
150
140
130
120
110
100
90
80
70
60
50
40
30
20
10
ppm

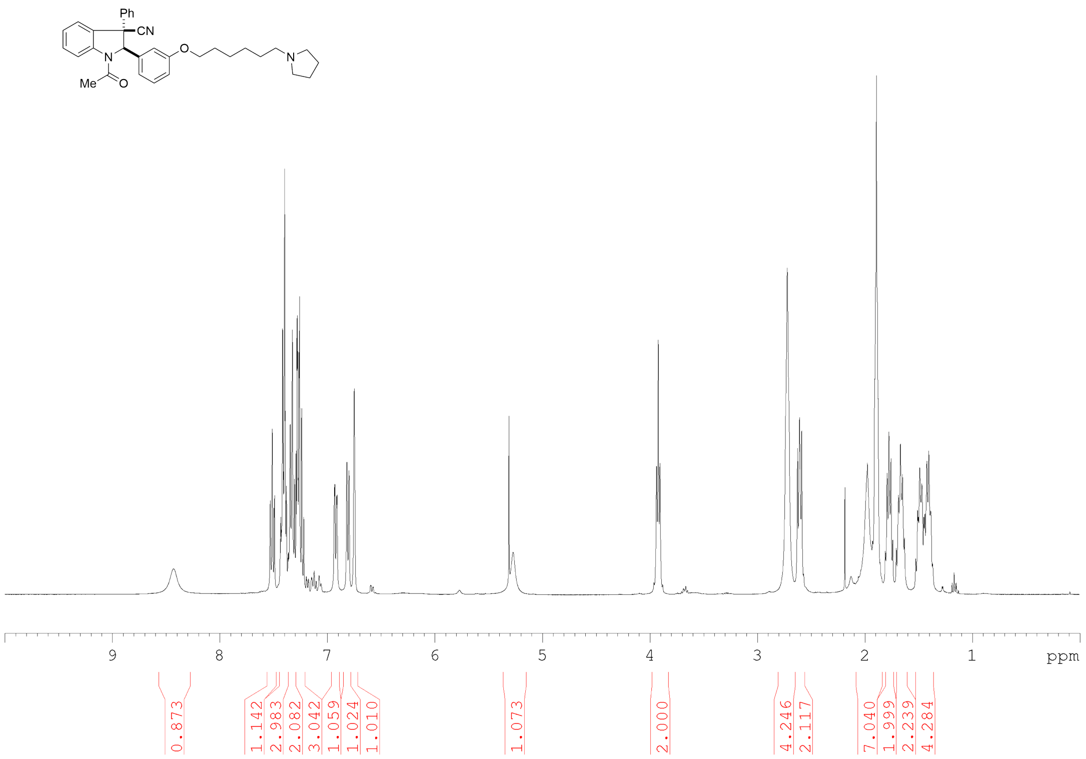
Ph
CN
N
O
Me
O
N
ppm
0.873
1.142
2.983
2.082
3.042
1.059
1.024
1.010
1.073
2.000
4.246
2.117
7.040
1.999
2.239
4.284

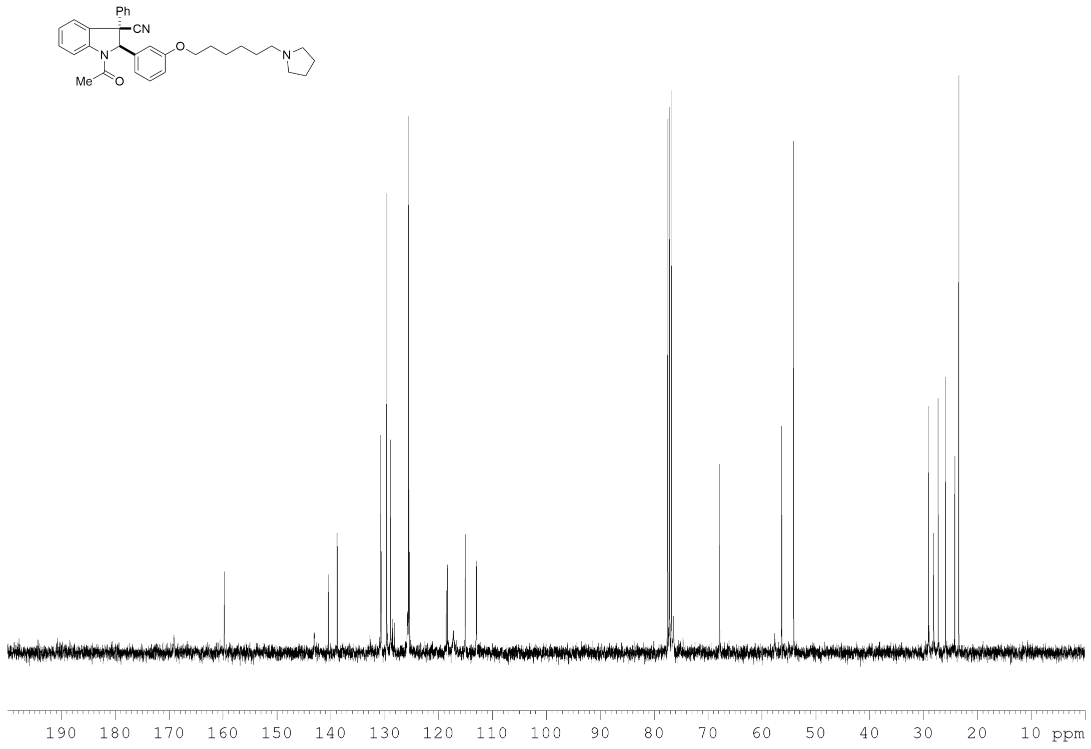
Ph
CN
N
O
Me
O
N
ppm

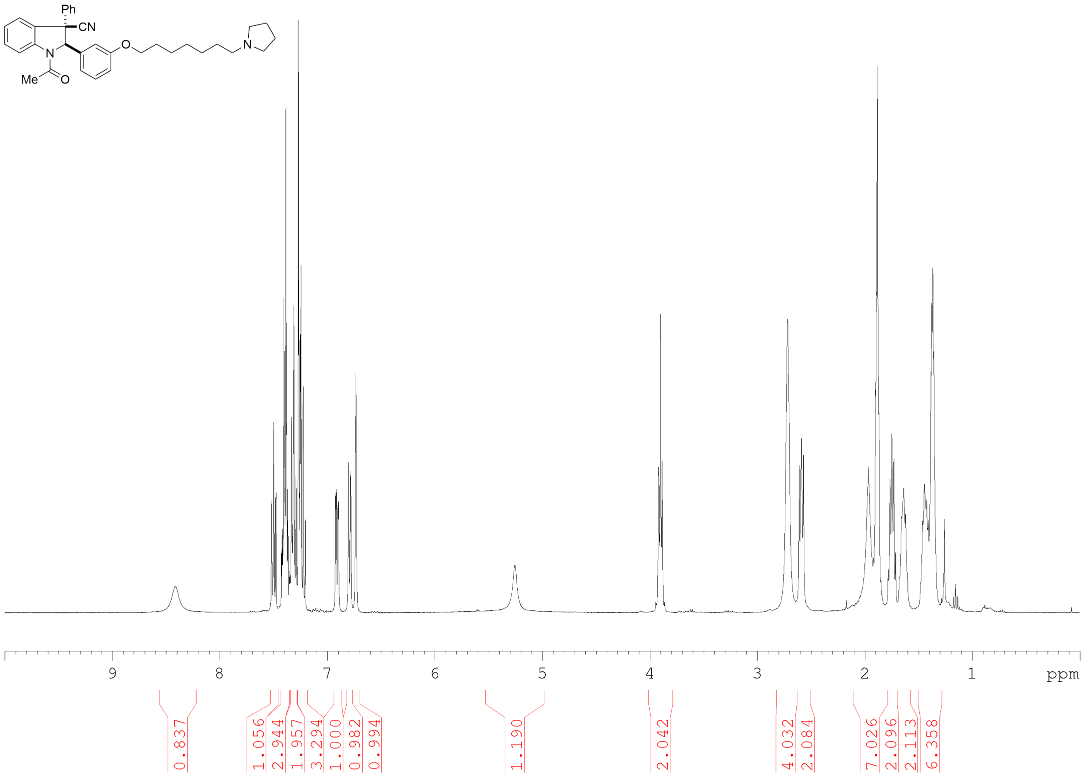
Ph
CN
N
O
N
Me
O
9
8
7
6
5
4
3
2
1
ppm
0.837
1.056
2.944
1.957
3.294
1.000
0.982
0.994
1.190
2.042
4.032
2.084
7.026
2.096
2.113
6.358

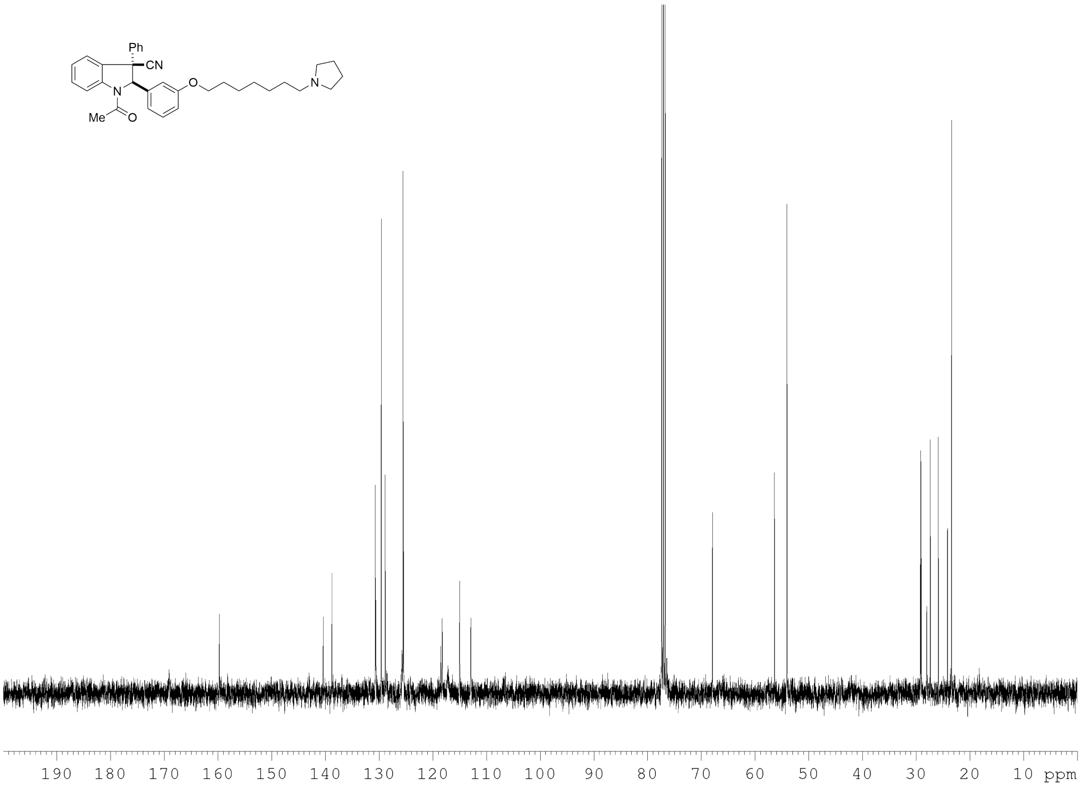
Ph
CN
N
O
N
Me
O
190
180
170
160
150
140
130
120
110
100
90
80
70
60
50
40
30
20
10
ppm

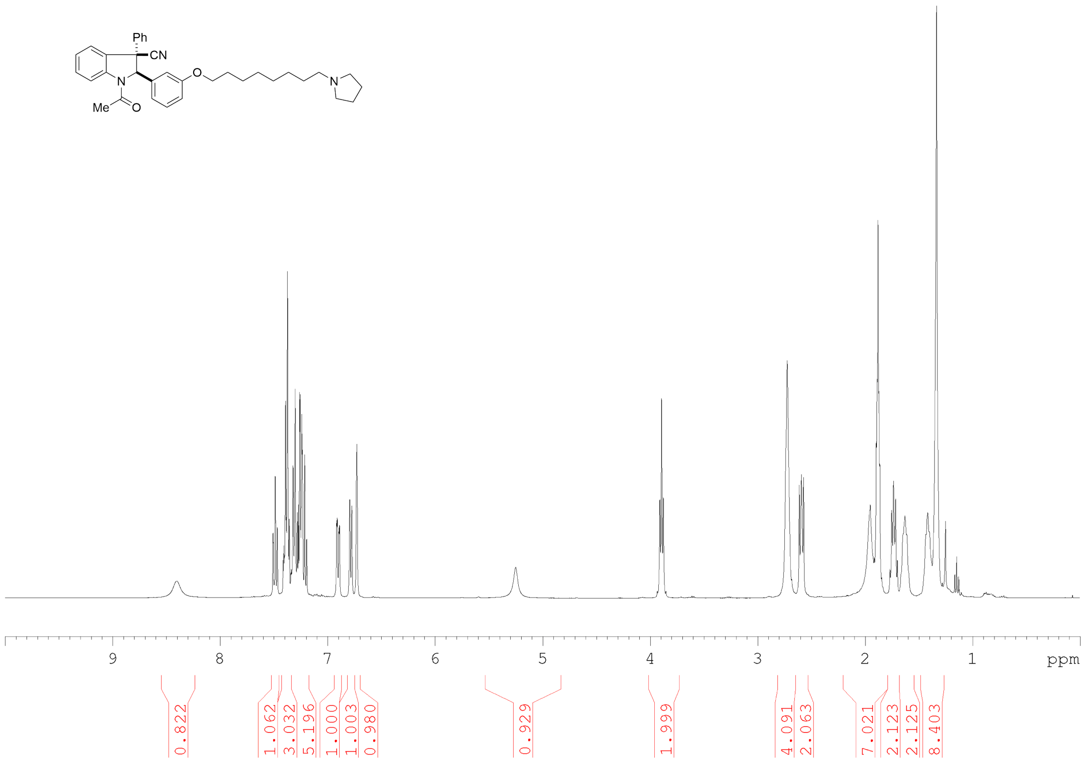
Ph
CN
N
O
Me
N
9
8
7
6
5
4
3
2
1
ppm
0.822
1.062
3.032
5.196
1.000
1.003
0.980
0.929
1.999
4.091
2.063
7.021
2.123
2.125
8.403

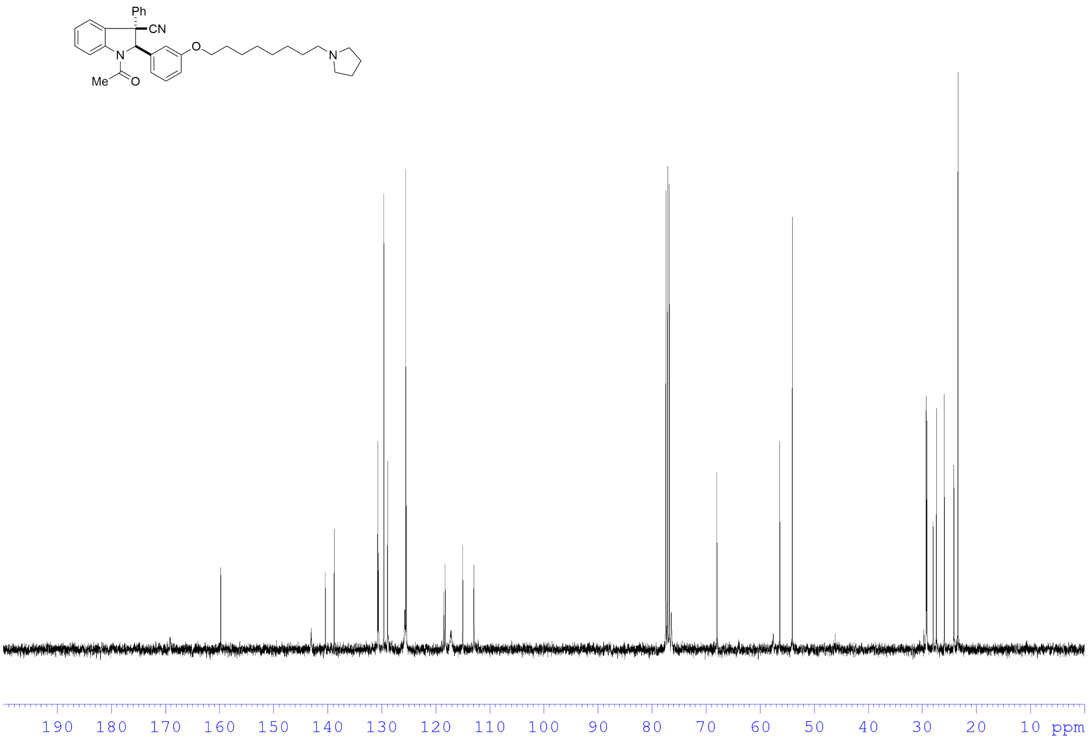
Ph
CN
N
O
Me
N
190
180
170
160
150
140
130
120
110
100
90
80
70
60
50
40
30
20
10
ppm

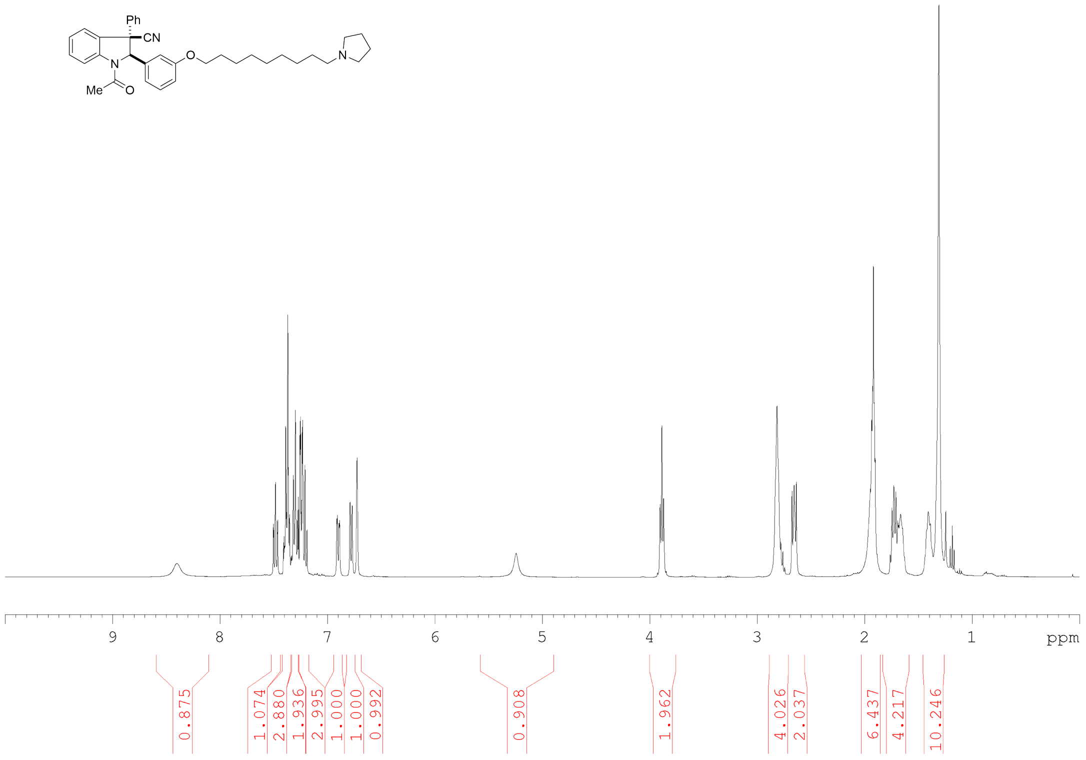
Ph
CN
O
N
N
Me
O
9 8 7 6 5 4 3 2 1 ppm
0.875
1.074
2.880
1.936
2.995
1.000
1.000
0.992
0.908
1.962
4.026
2.037
6.437
4.217
10.246

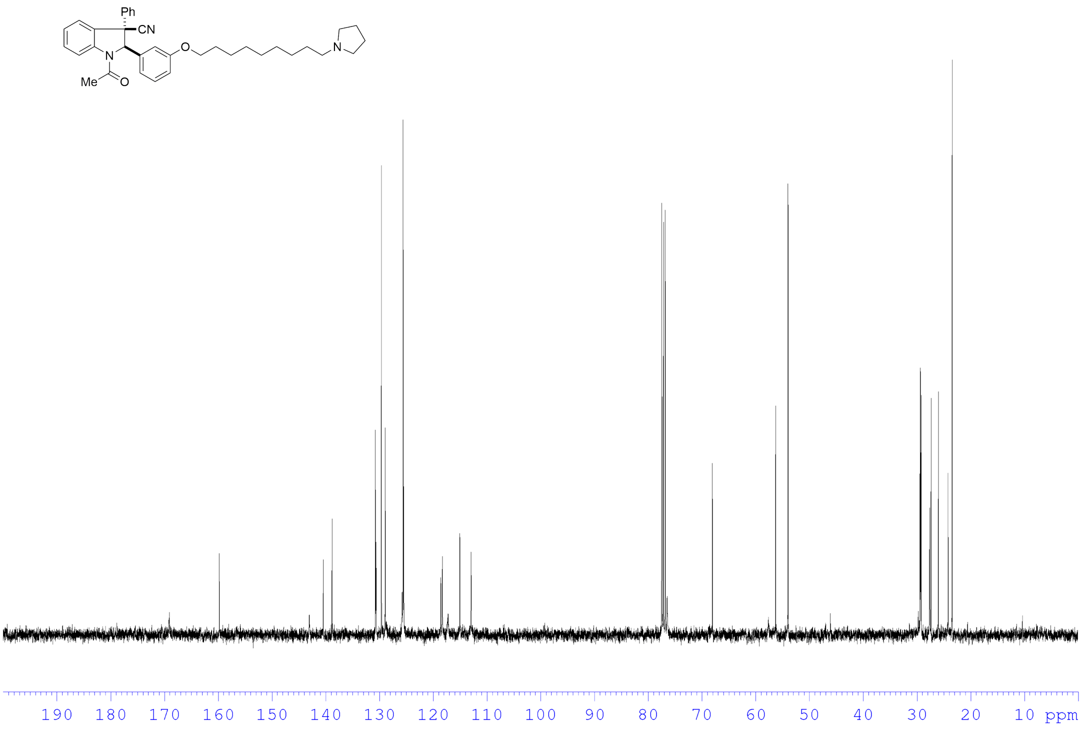
Ph
CN
O
N
N
Me
O
190 180 170 160 150 140 130 120 110 100 90 80 70 60 50 40 30 20 10 ppm

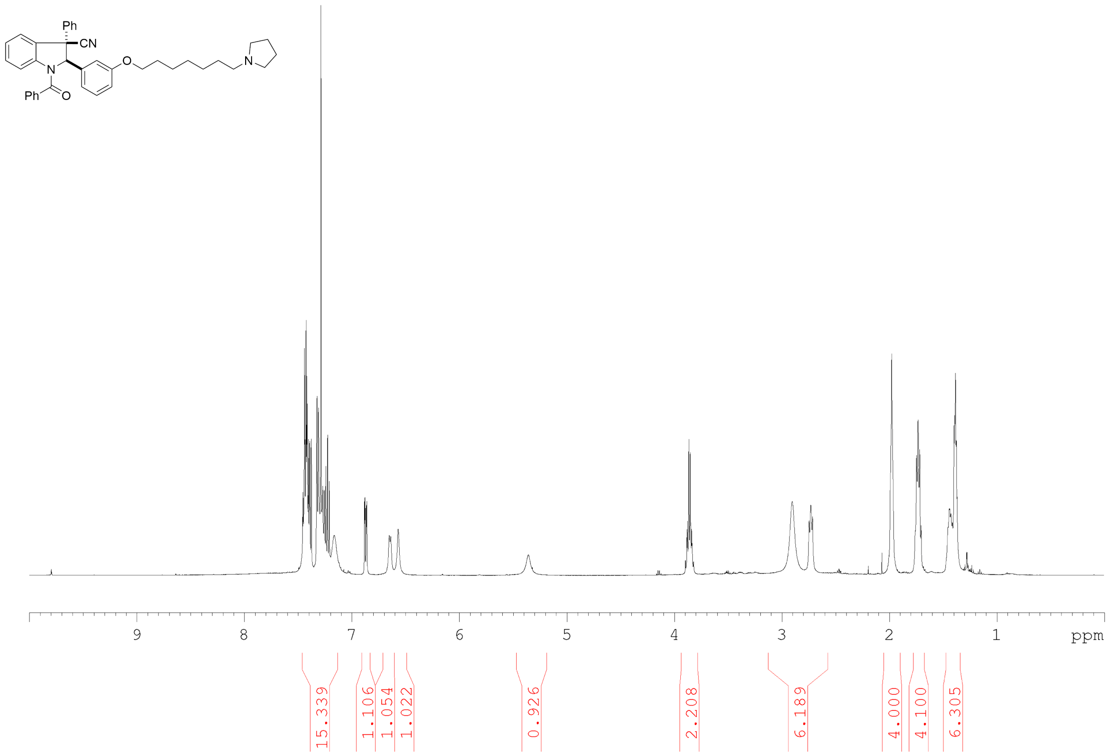
Ph
CN
N
O
Ph
O
N
9 8 7 6 5 4 3 2 1 ppm
15.339
1.106
1.054
1.022
0.926
2.208
6.189
4.000
4.100
6.305

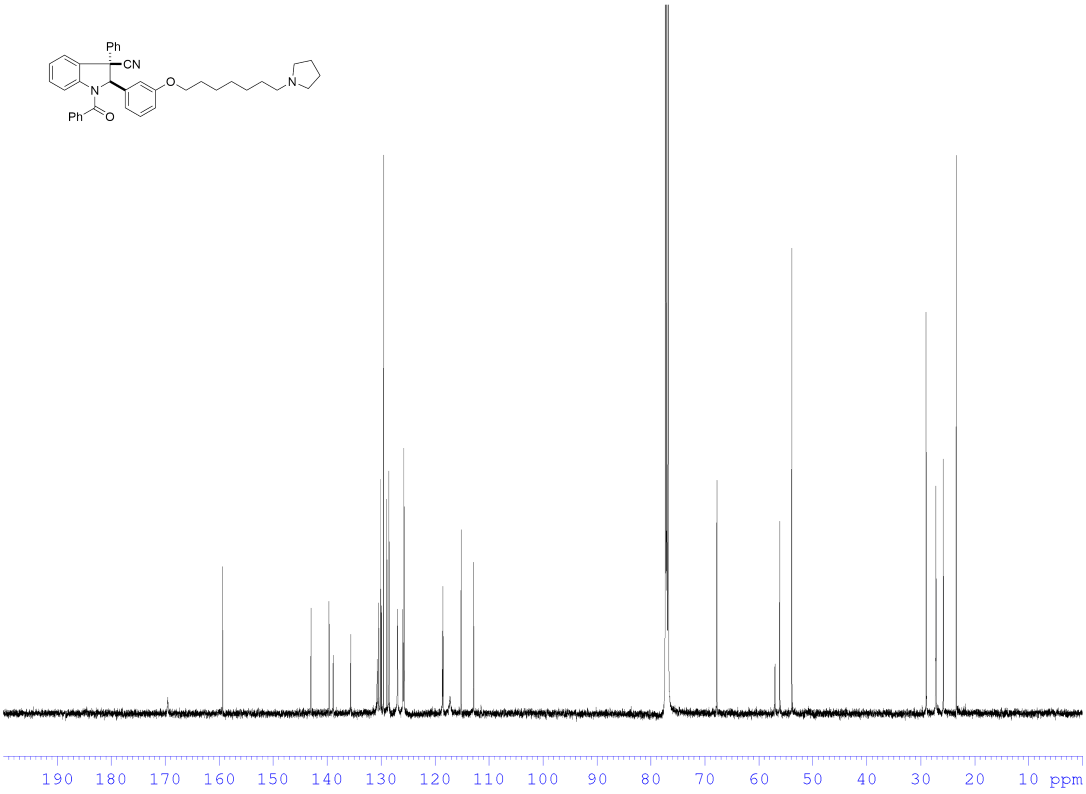
Ph
CN
N
O
Ph
O
N
190 180 170 160 150 140 130 120 110 100 90 80 70 60 50 40 30 20 10 ppm

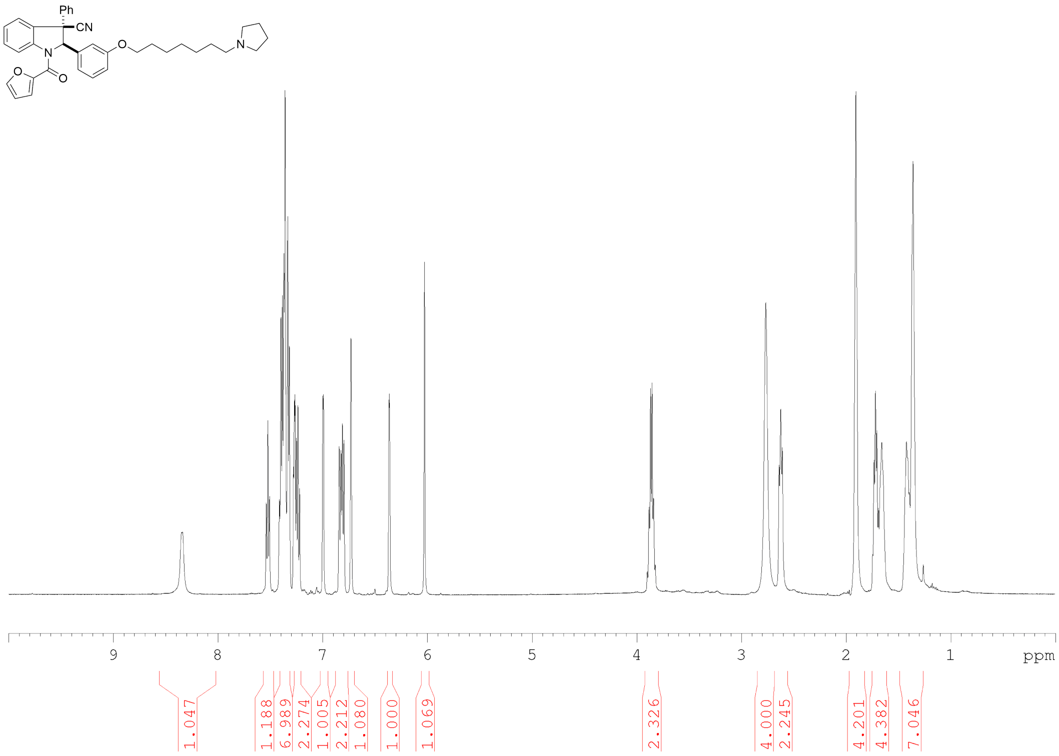
Ph
CN
N
O
O
O
N
9 8 7 6 5 4 3 2 1 ppm
1.047
1.188
6.989
2.274
1.005
2.212
1.080
1.000
1.069
2.326
4.000
2.245
4.201
4.382
7.046

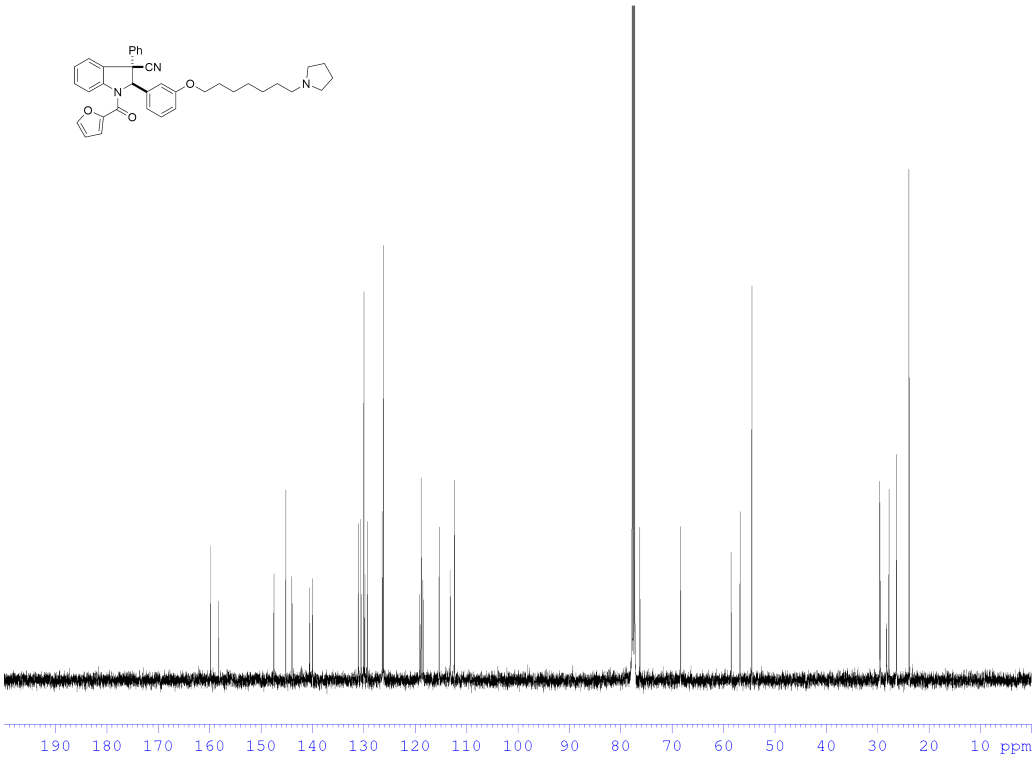
Ph
CN
N
O
O
O
N
190 180 170 160 150 140 130 120 110 100 90 80 70 60 50 40 30 20 10 ppm

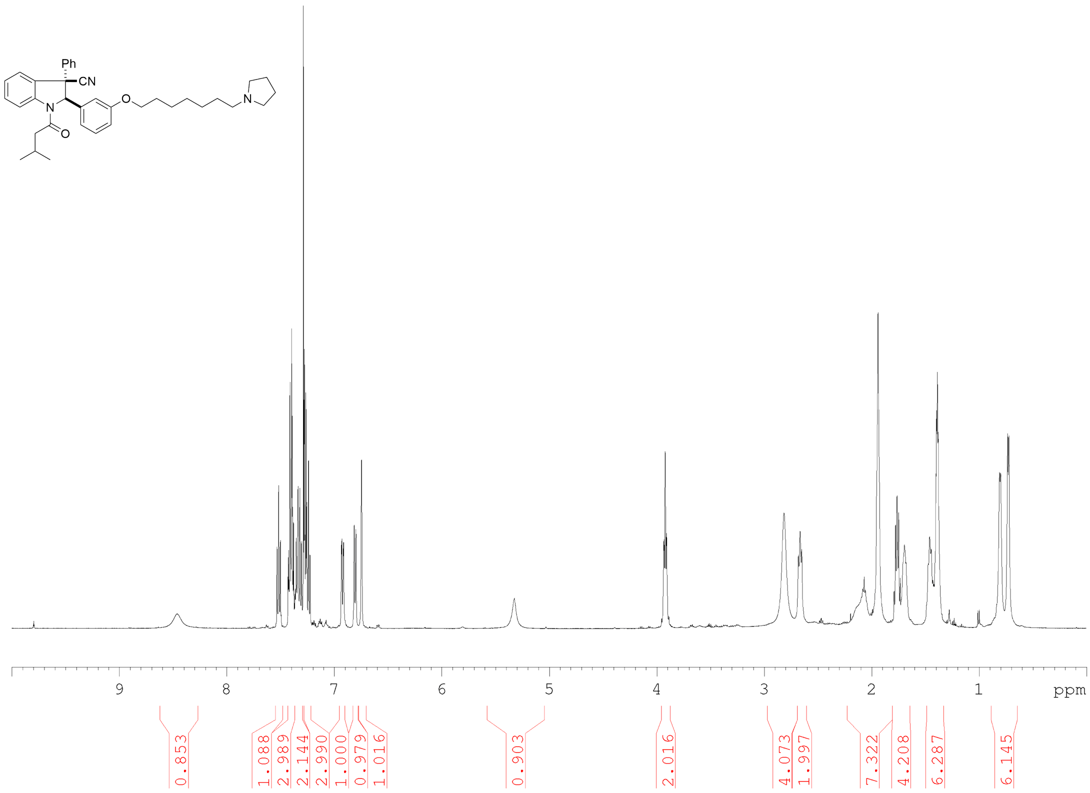
Ph
CN
N
O
O
N
9
8
7
6
5
4
3
2
1
ppm
0.853
1.088
2.989
2.144
2.990
1.000
0.979
1.016
0.903
2.016
4.073
1.997
7.322
4.208
6.287
6.145

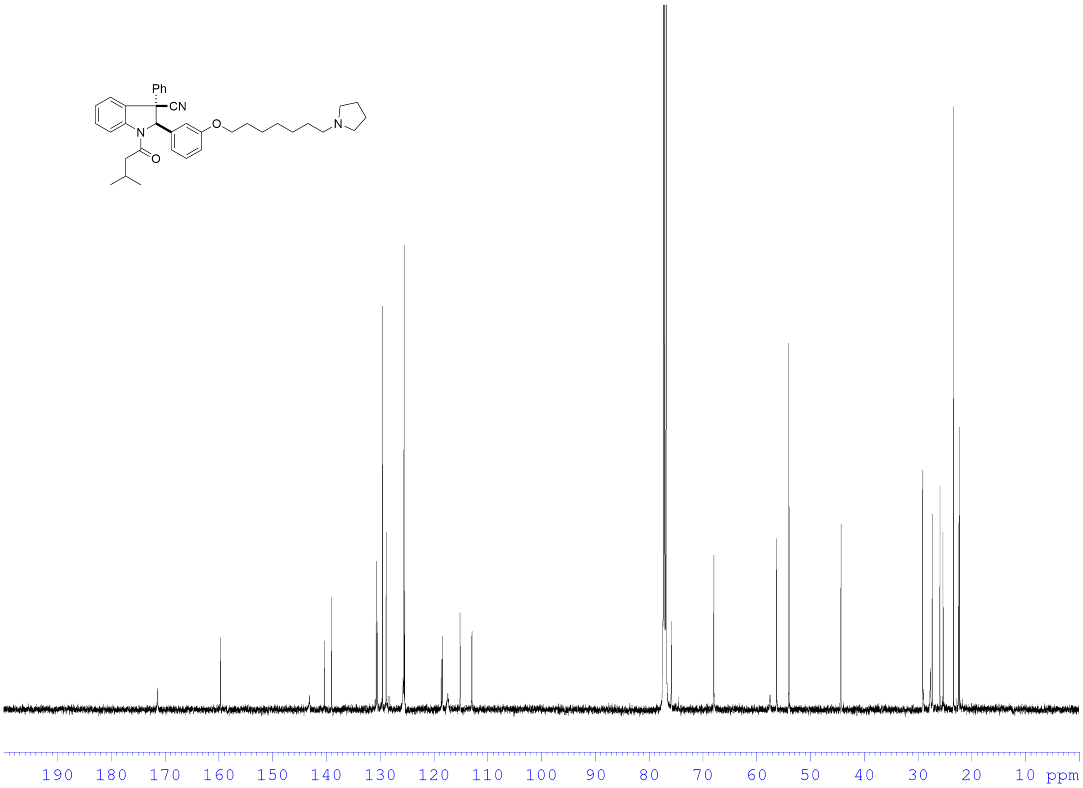
Ph
CN
N
O
O
N
190
180
170
160
150
140
130
120
110
100
90
80
70
60
50
40
30
20
10
ppm

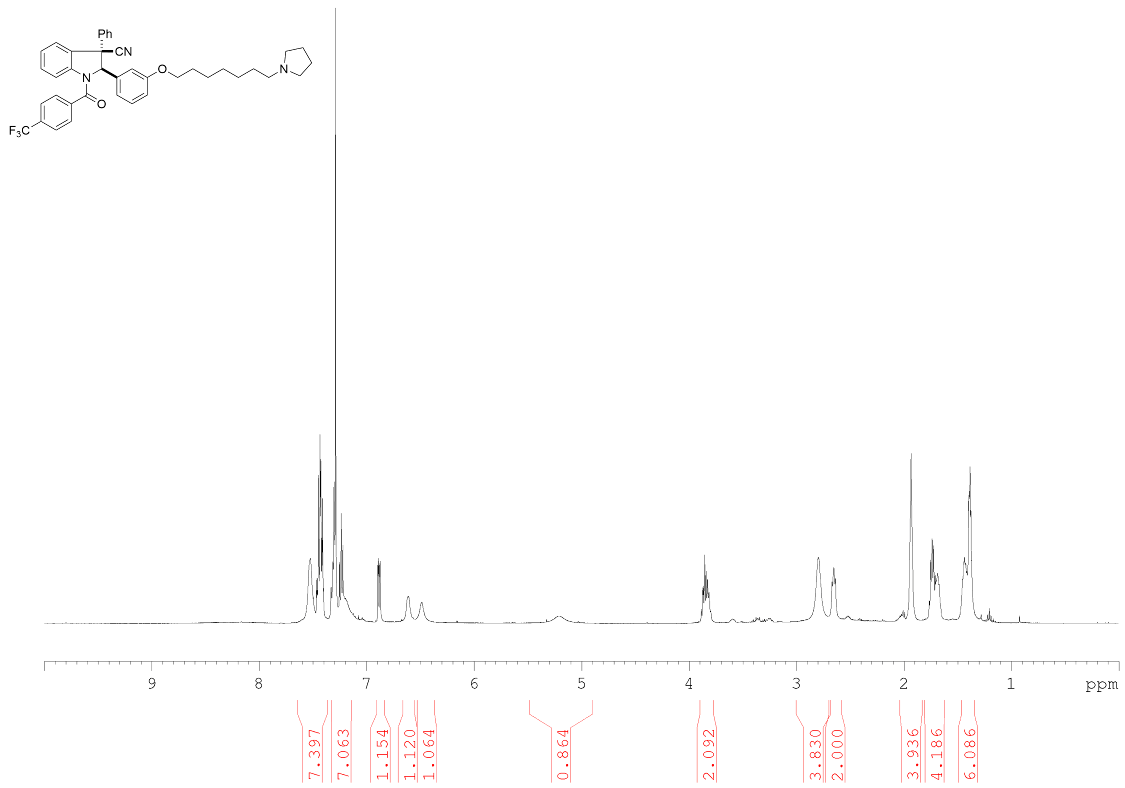

Ph
CN
O
N
N
O
F3C
9
8
7
6
5
4
3
2
1
ppm
7.397
7.063
1.154
1.120
1.064
0.864
2.092
3.830
2.000
3.936
4.186
6.086

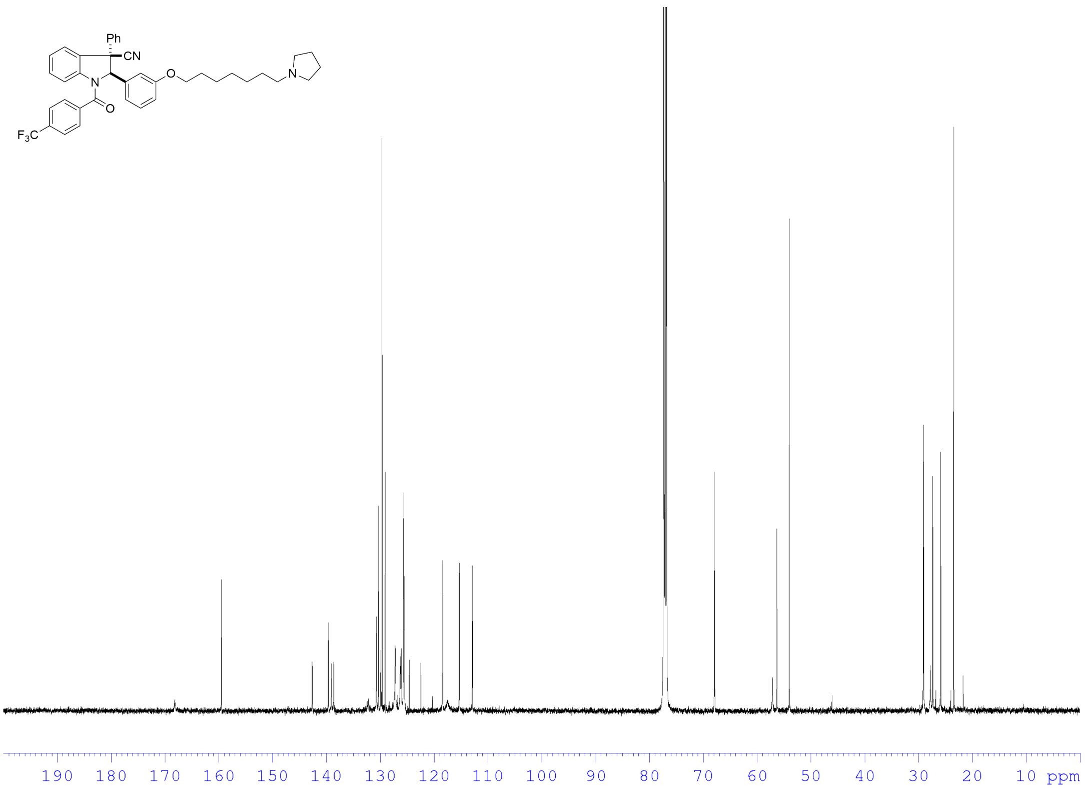

Ph
CN
O
N
N
O
F3C
190
180
170
160
150
140
130
120
110
100
90
80
70
60
50
40
30
20
10
ppm

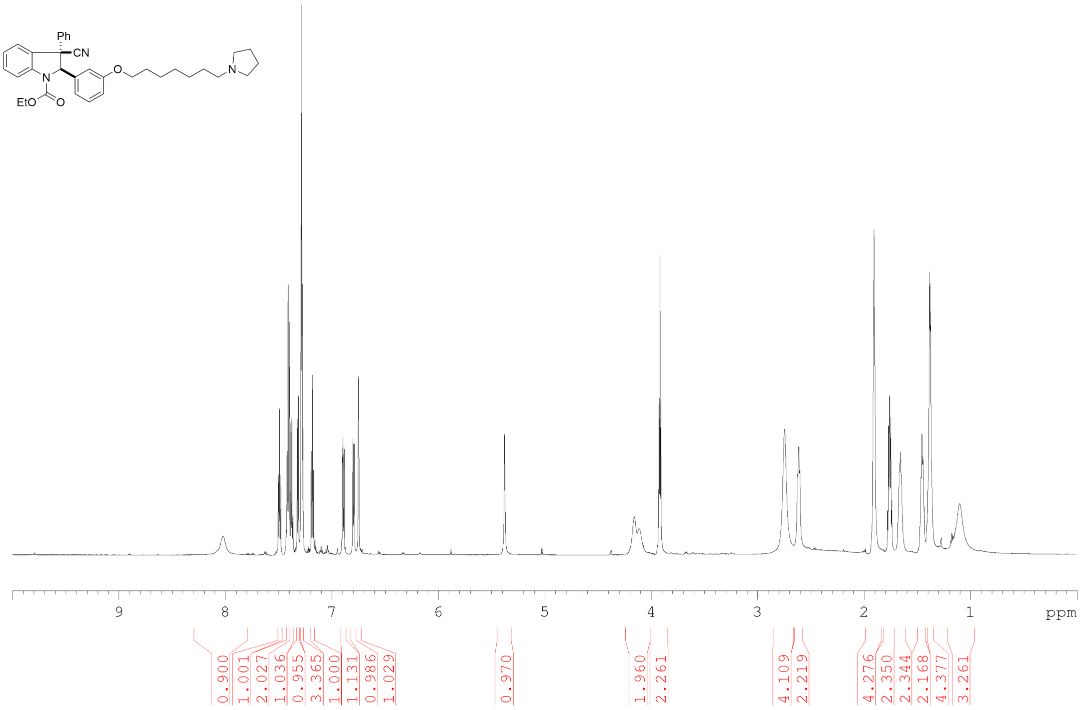
Ph
CN
O
N
EtO
O
9
8
7
6
5
4
3
2
1
ppm
0.900
1.001
2.027
1.036
0.955
3.365
1.000
1.131
0.986
1.029
0.970
1.960
2.261
4.109
2.219
4.276
2.350
2.344
2.168
4.377
3.261

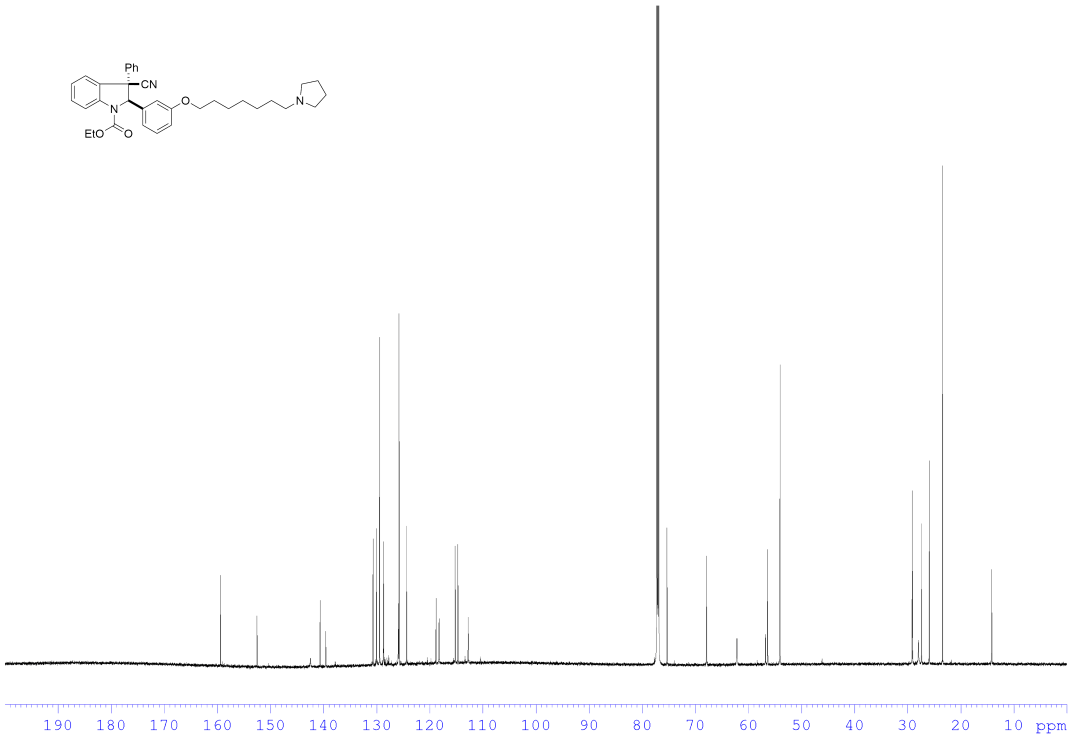
Ph
CN
O
N
EtO
O
190
180
170
160
150
140
130
120
110
100
90
80
70
60
50
40
30
20
10
ppm

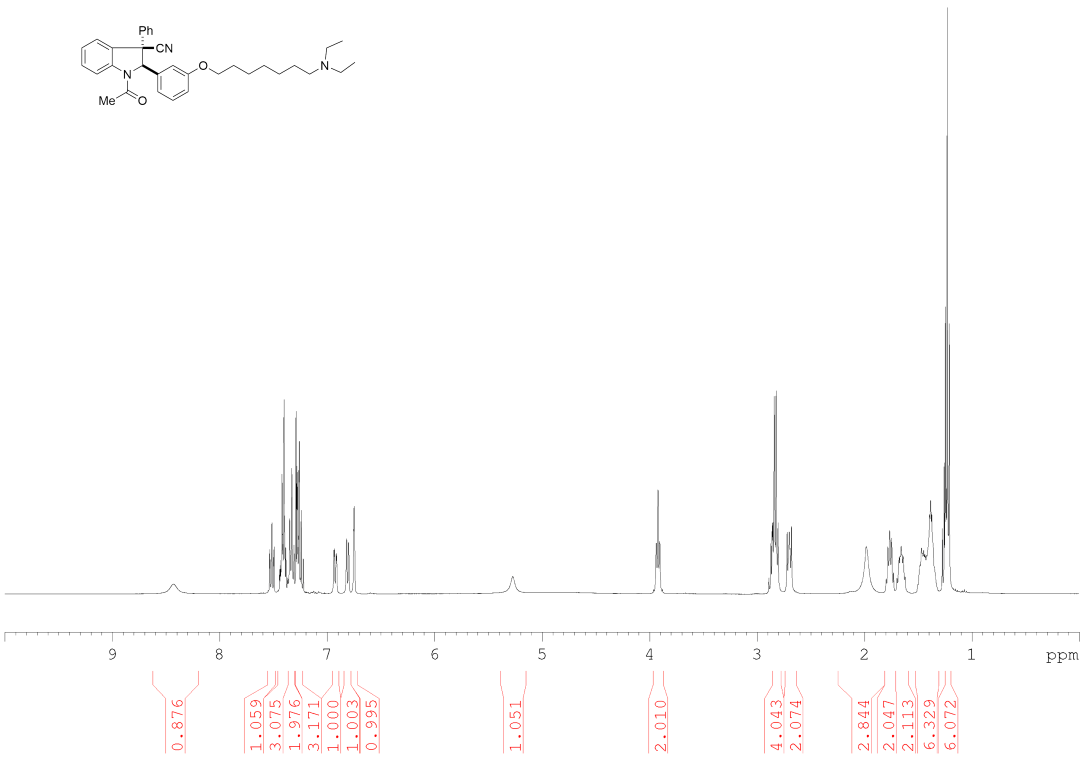
Ph
CN
N
O
N
Me
O
9
8
7
6
5
4
3
2
1
ppm
0.876
1.059
3.075
1.976
3.171
1.000
1.003
0.995
1.051
2.010
4.043
2.074
2.844
2.047
2.113
6.329
6.072

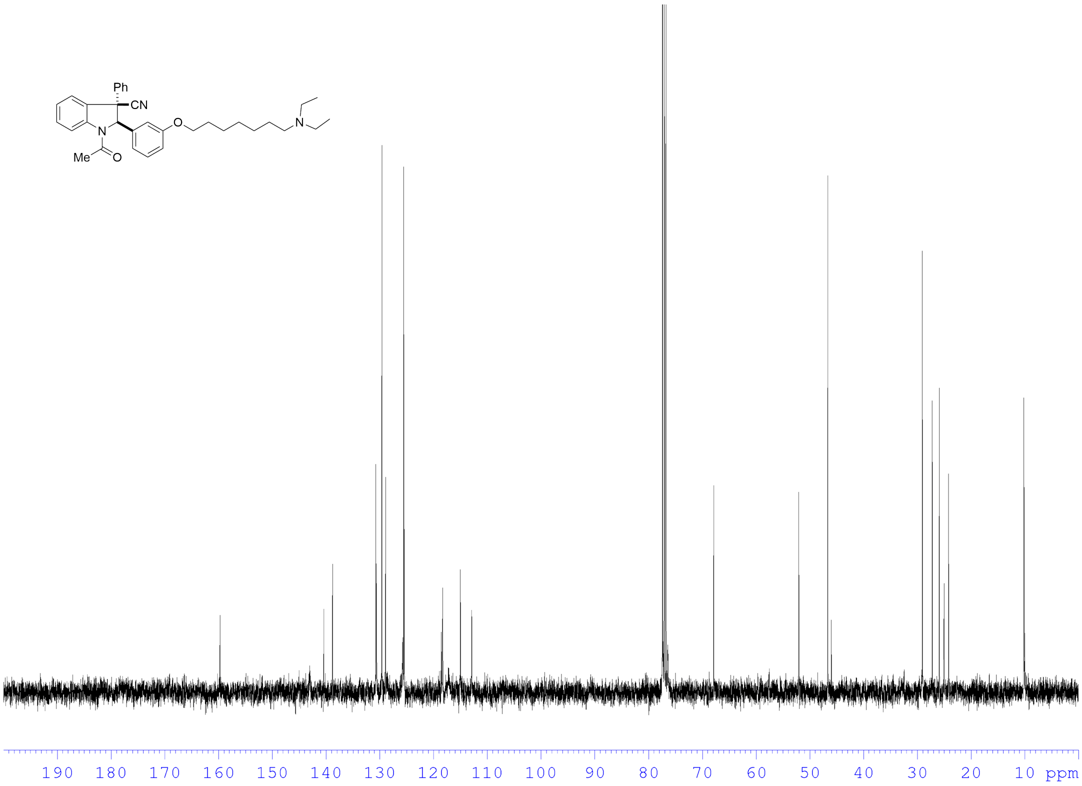
Ph
CN
N
O
N
Me
O
190
180
170
160
150
140
130
120
110
100
90
80
70
60
50
40
30
20
10
ppm

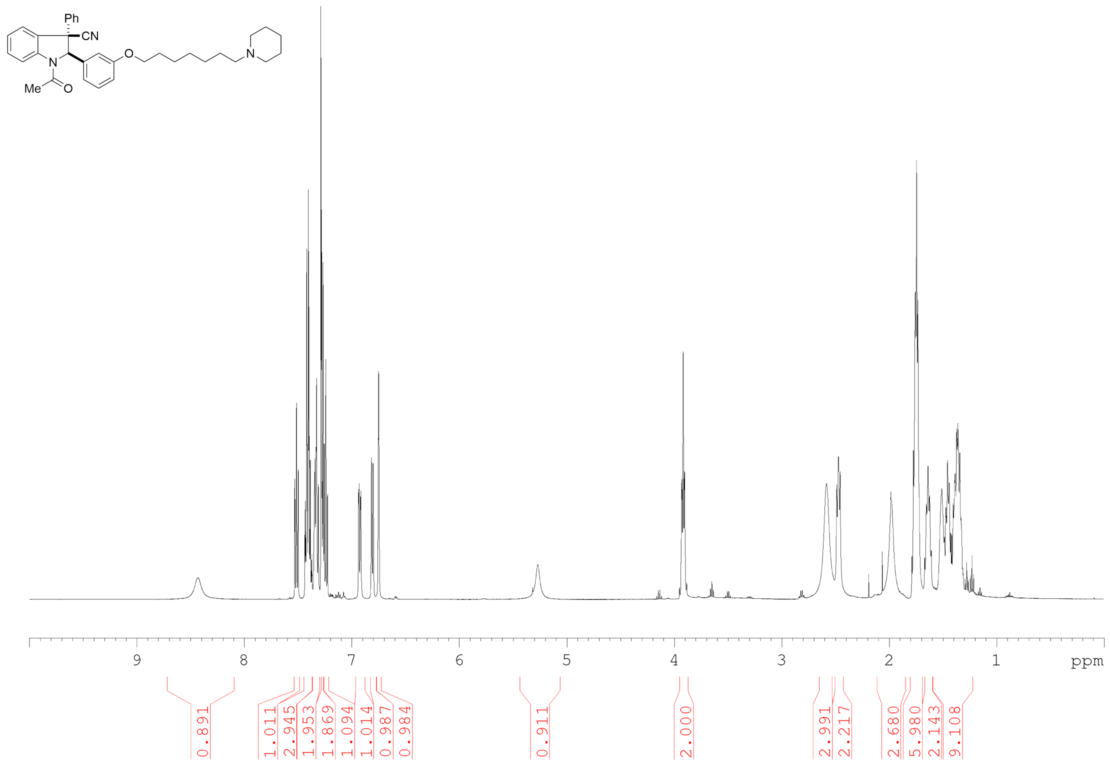
Ph
CN
N
O
N
Me
O
9
8
7
6
5
4
3
2
1
ppm
0.891
1.011
2.945
1.953
1.869
1.094
1.014
0.987
0.984
0.911
2.000
2.991
2.217
2.680
5.980
2.143
9.108

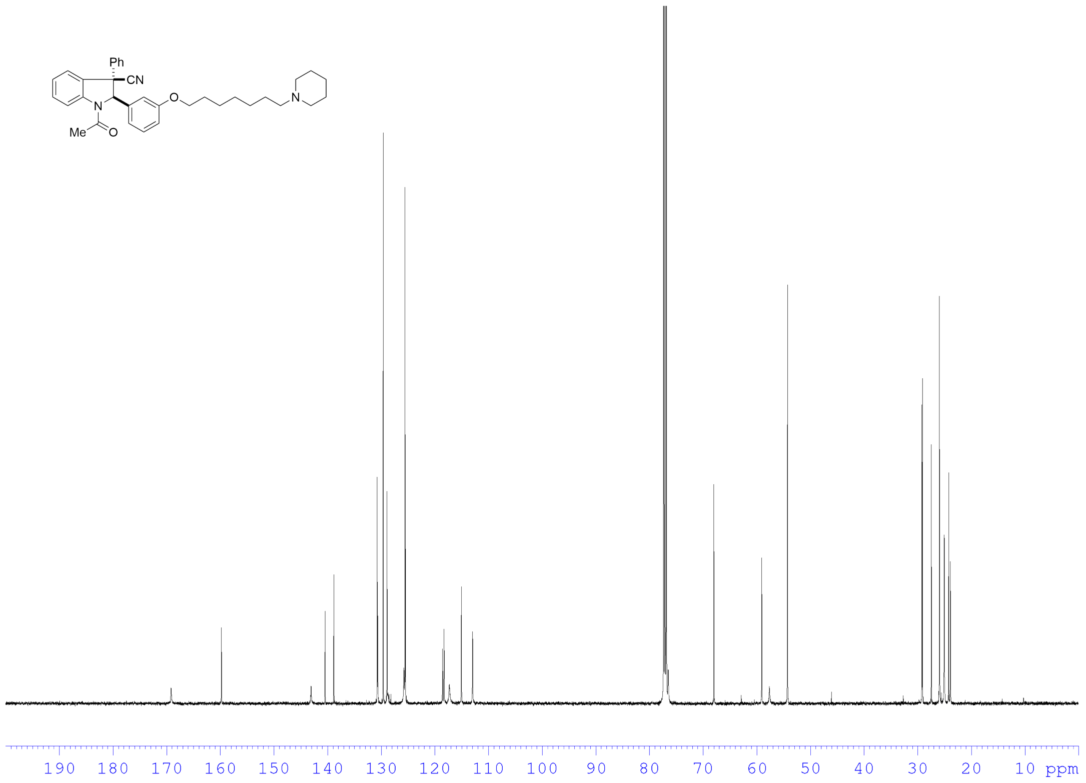
Ph
CN
N
O
N
Me
O
190
180
170
160
150
140
130
120
110
100
90
80
70
60
50
40
30
20
10
ppm

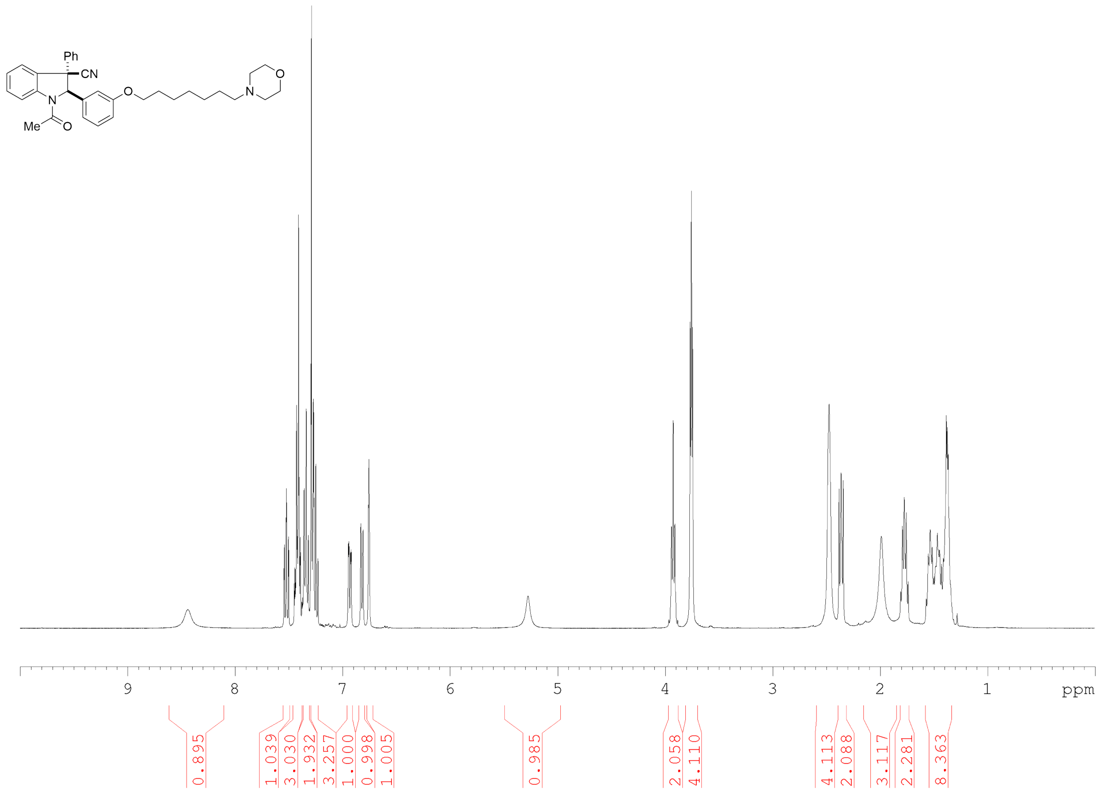
Ph
CN
N
O
Me
9 8 7 6 5 4 3 2 1 ppm
0.895
1.039
3.030
1.932
3.257
1.000
0.998
1.005
0.985
2.058
4.110
4.113
2.088
3.117
2.281
8.363

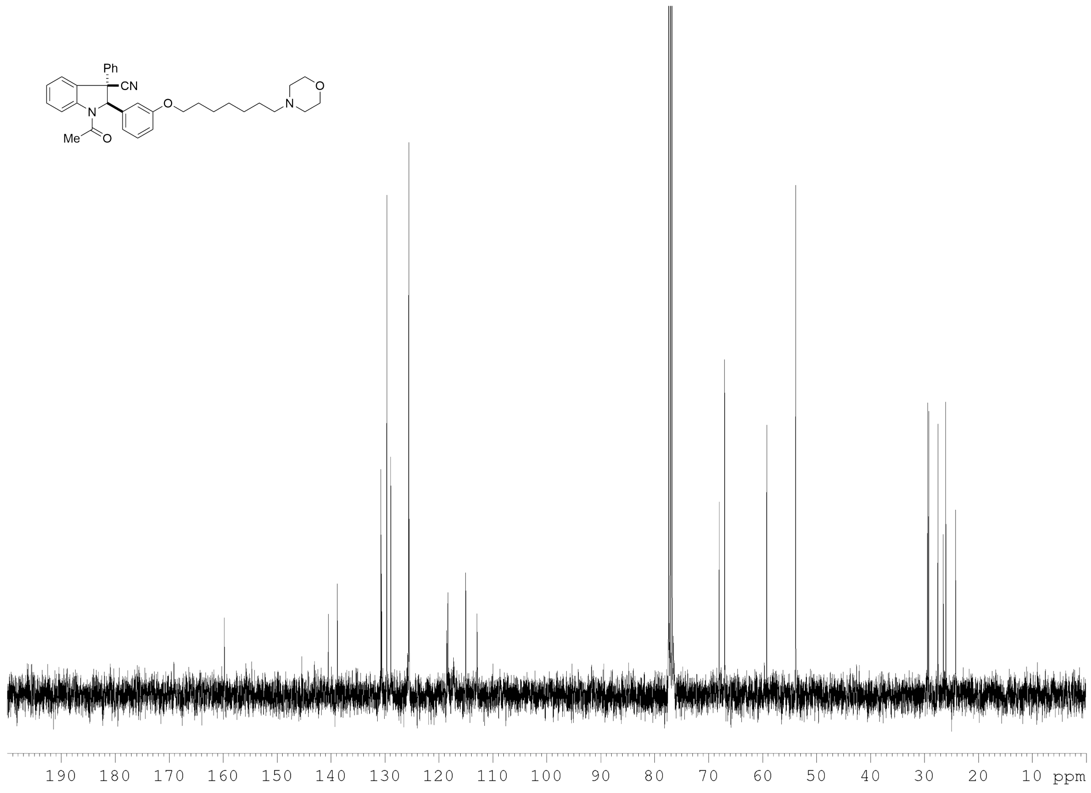
Ph
CN
N
O
Me
190 180 170 160 150 140 130 120 110 100 90 80 70 60 50 40 30 20 10 ppm

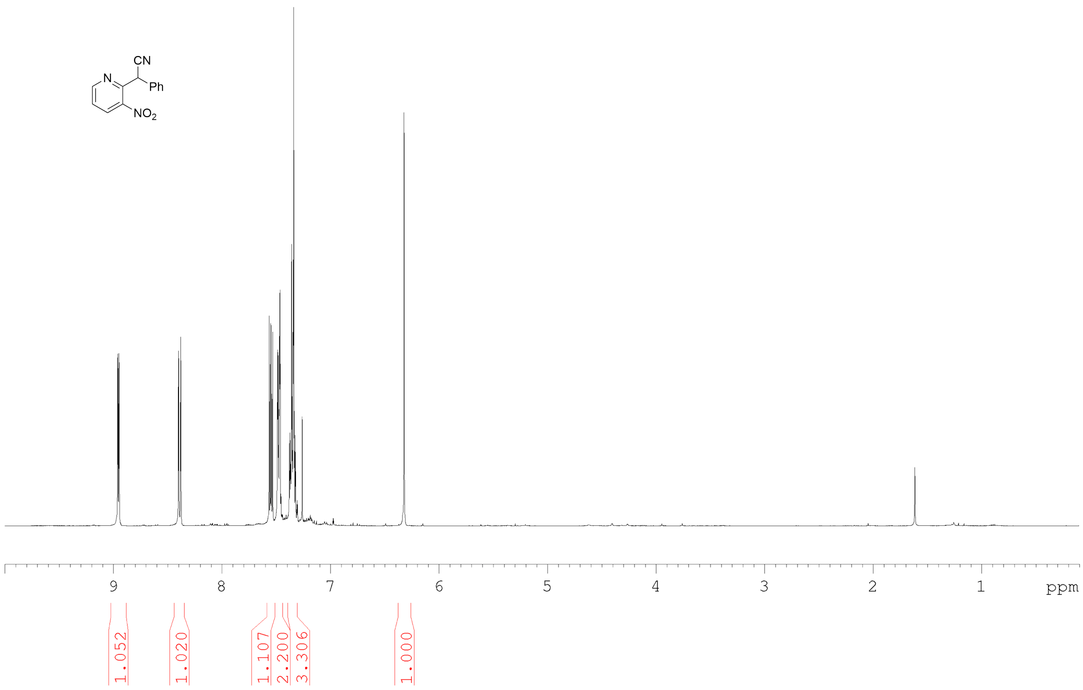
CN
N
Ph
NO2
9
8
7
6
5
4
3
2
1
ppm
1.052
1.020
1.107
2.200
3.306
1.000

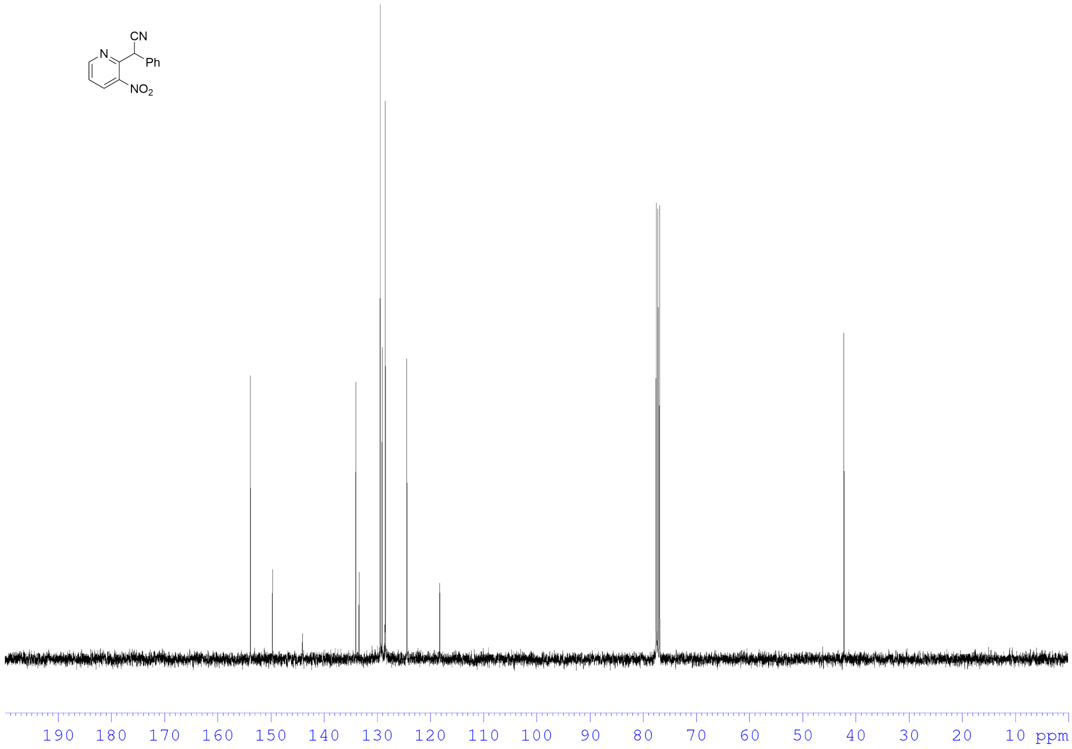
CN
N
Ph
NO2
190
180
170
160
150
140
130
120
110
100
90
80
70
60
50
40
30
20
10
ppm

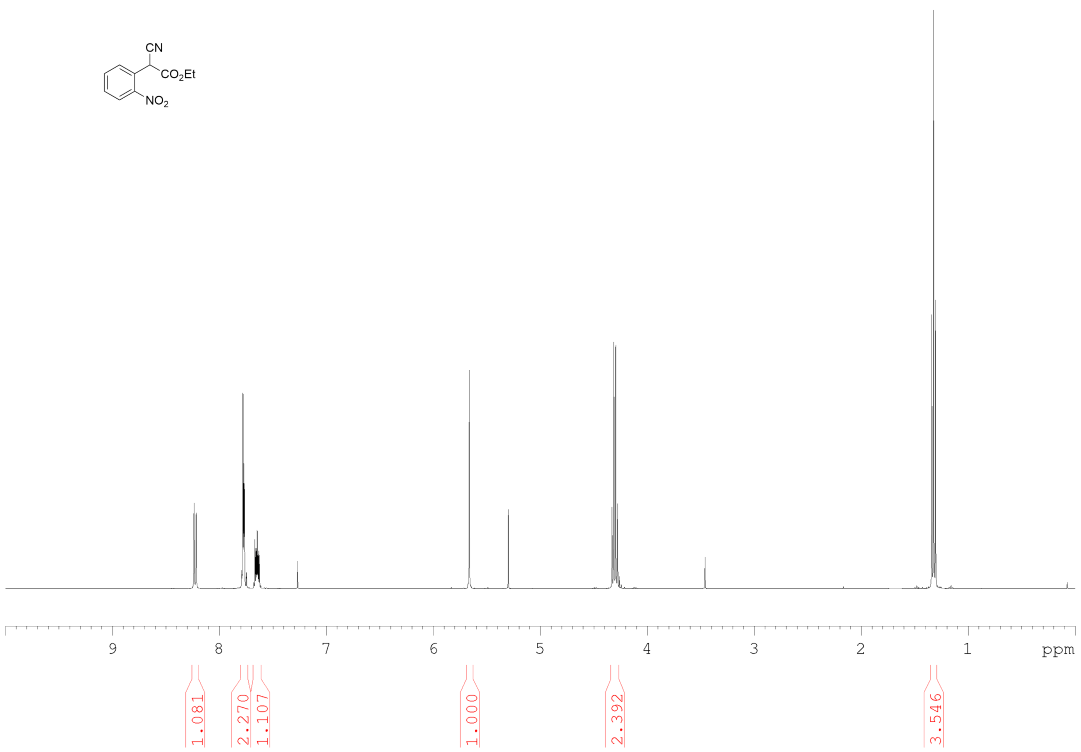
CN
CO2Et
NO2
9
8
7
6
5
4
3
2
1
ppm
1.081
2.270
1.107
1.000
2.392
3.546

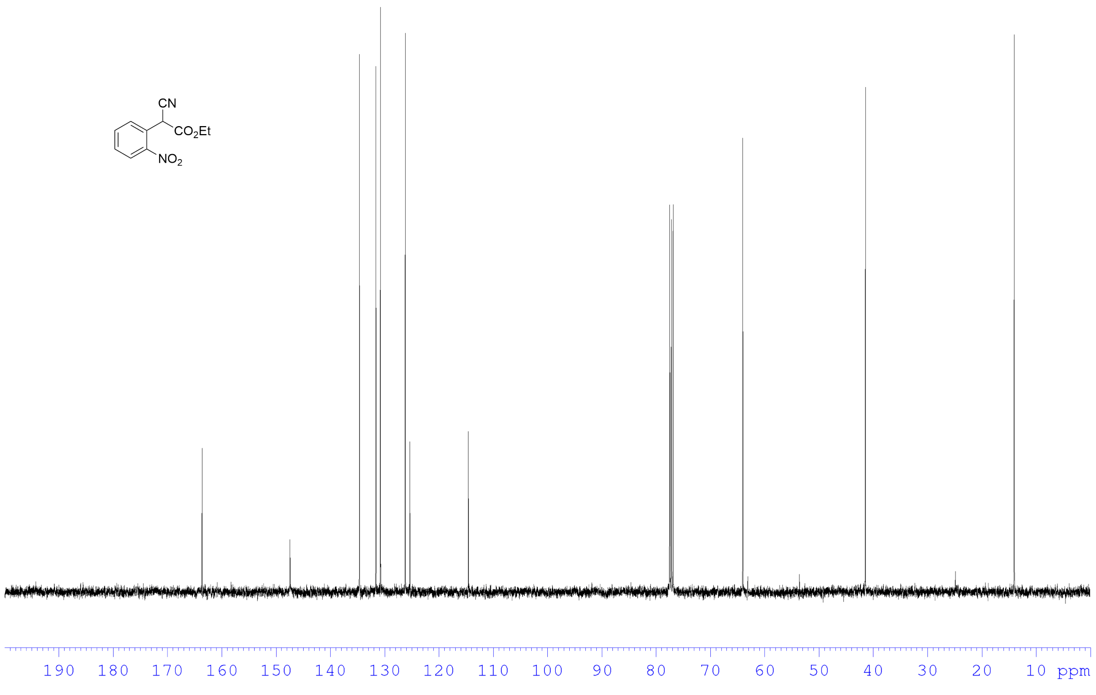
CN
CO2Et
NO2
190
180
170
160
150
140
130
120
110
100
90
80
70
60
50
40
30
20
10 ppm

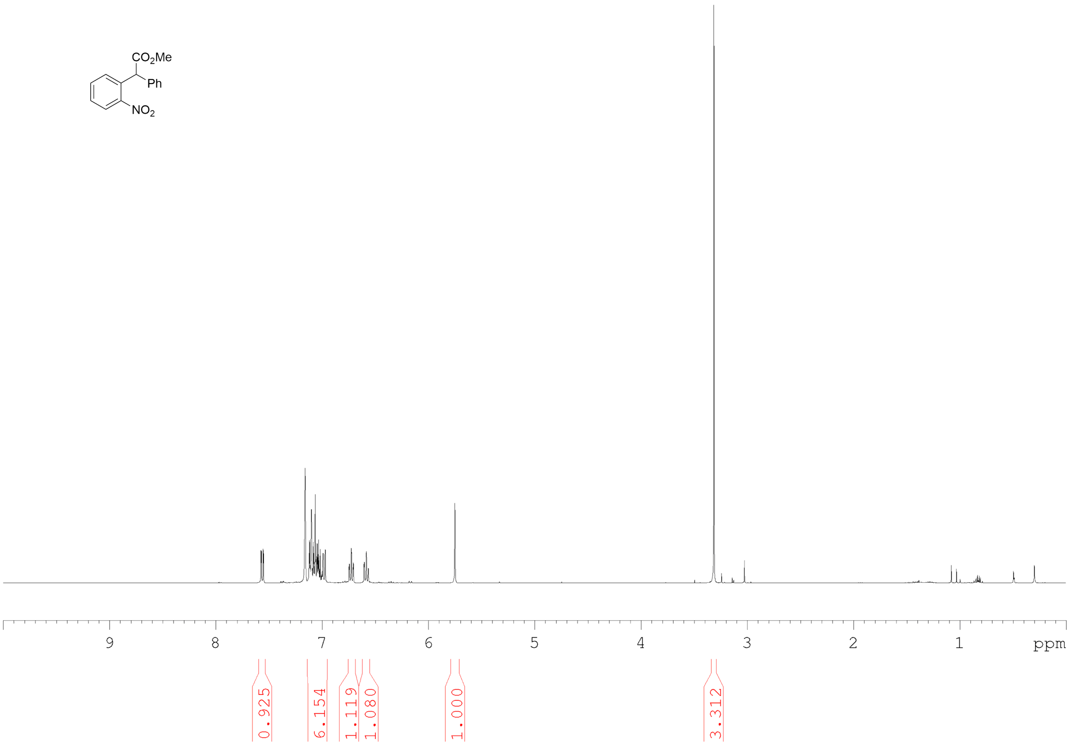
CO2Me
Ph
NO2
9
8
7
6
5
4
3
2
1
ppm
0.925
6.154
1.119
1.080
1.000
3.312

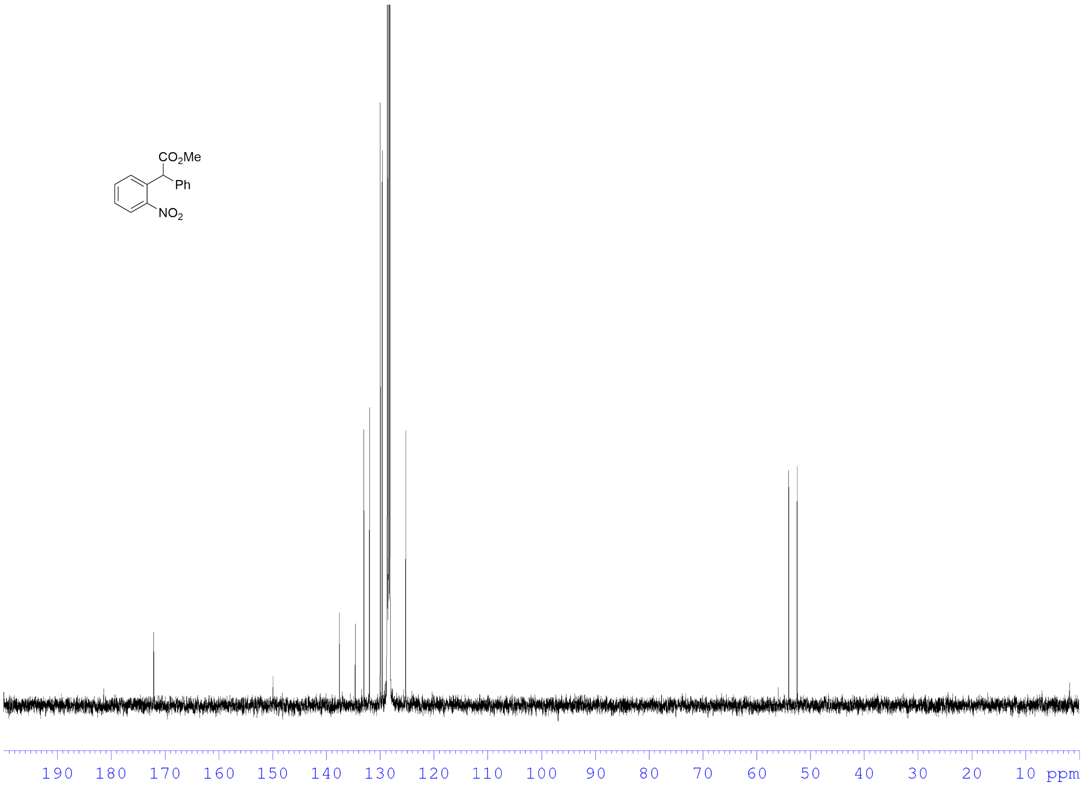
CO2Me
Ph
NO2
190
180
170
160
150
140
130
120
110
100
90
80
70
60
50
40
30
20
10
ppm

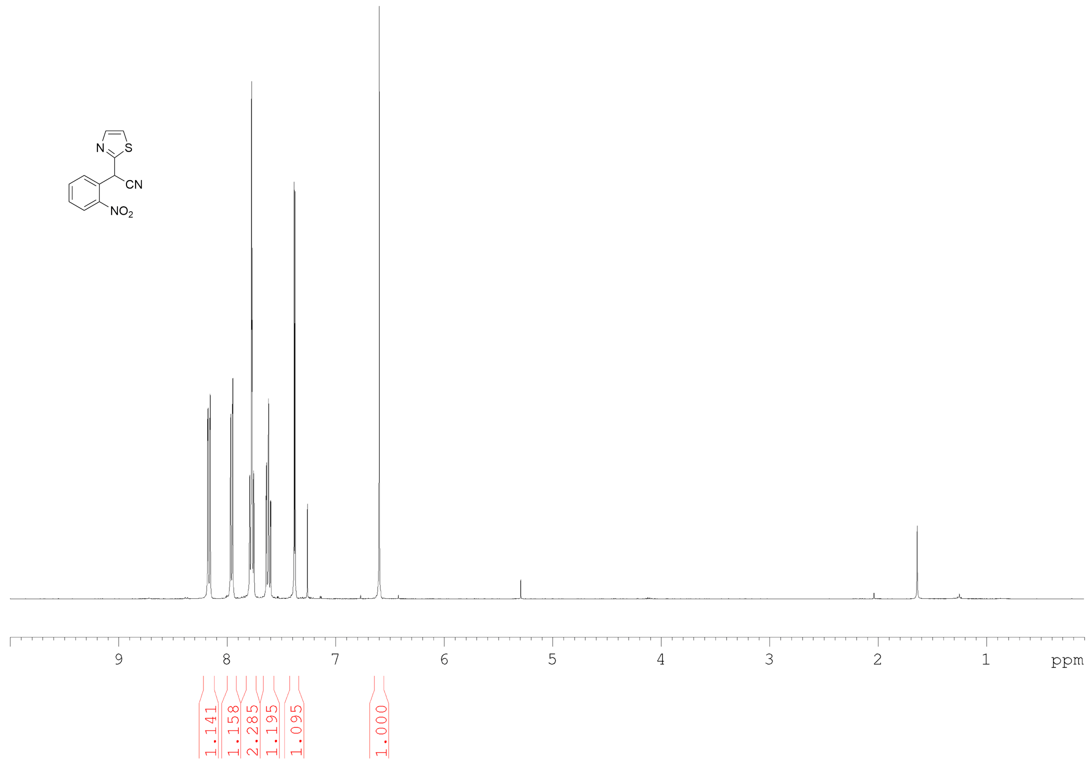
N
S
CN
NO2
9
8
7
6
5
4
3
2
1
ppm
1.141
1.158
2.285
1.195
1.095
1.000

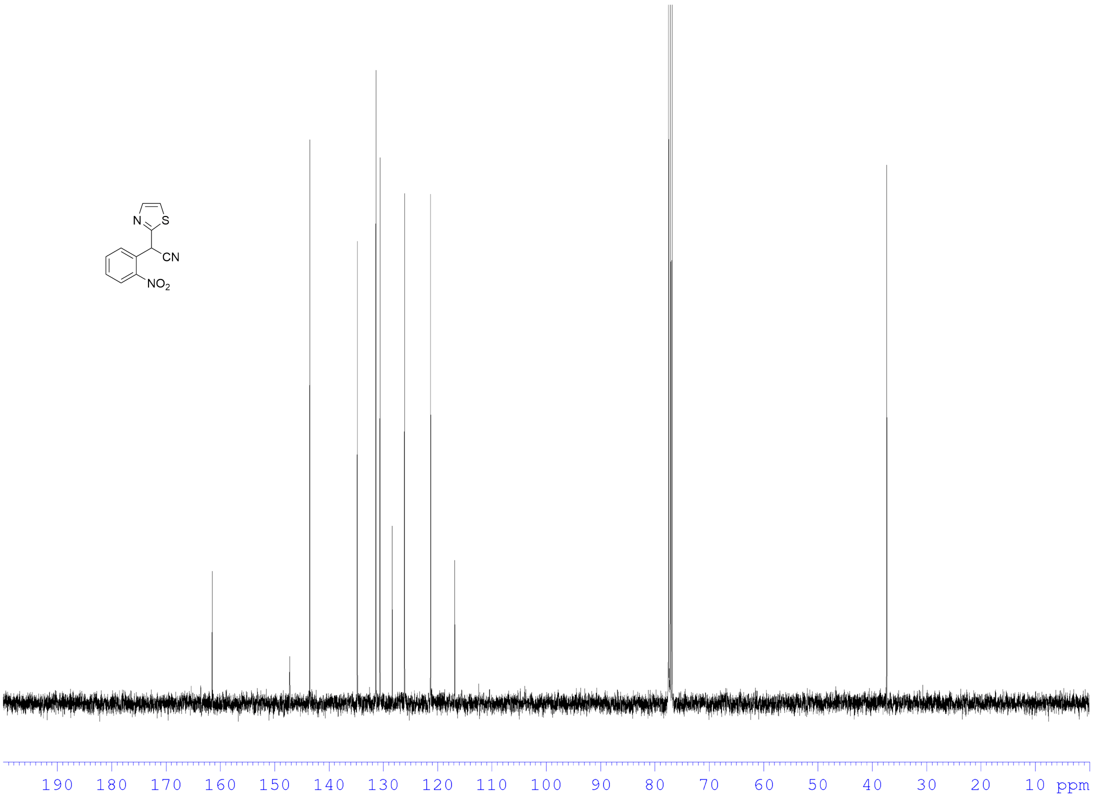
N
S
CN
NO2
190
180
170
160
150
140
130
120
110
100
90
80
70
60
50
40
30
20
10 ppm

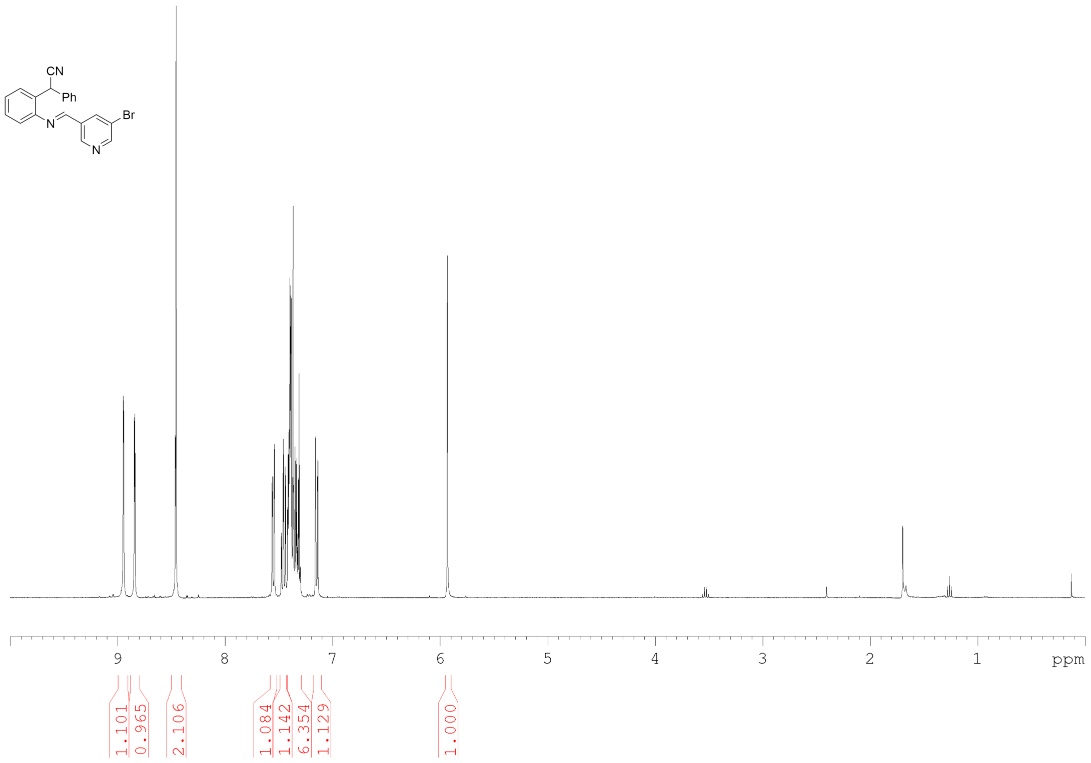
CN
Ph
N
Br
N
9
8
7
6
5
4
3
2
1
ppm
1.101
0.965
2.106
1.084
1.142
6.354
1.129
1.000

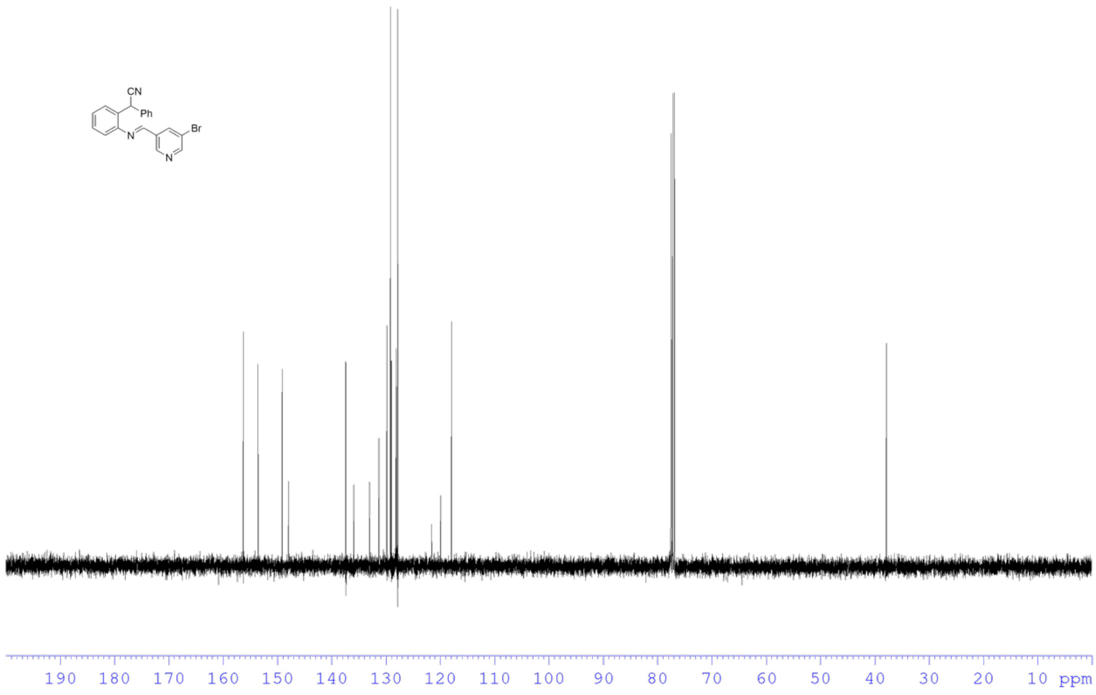
CN
Ph
N
Br
N
190
180
170
160
150
140
130
120
110
100
90
80
70
60
50
40
30
20
10
ppm

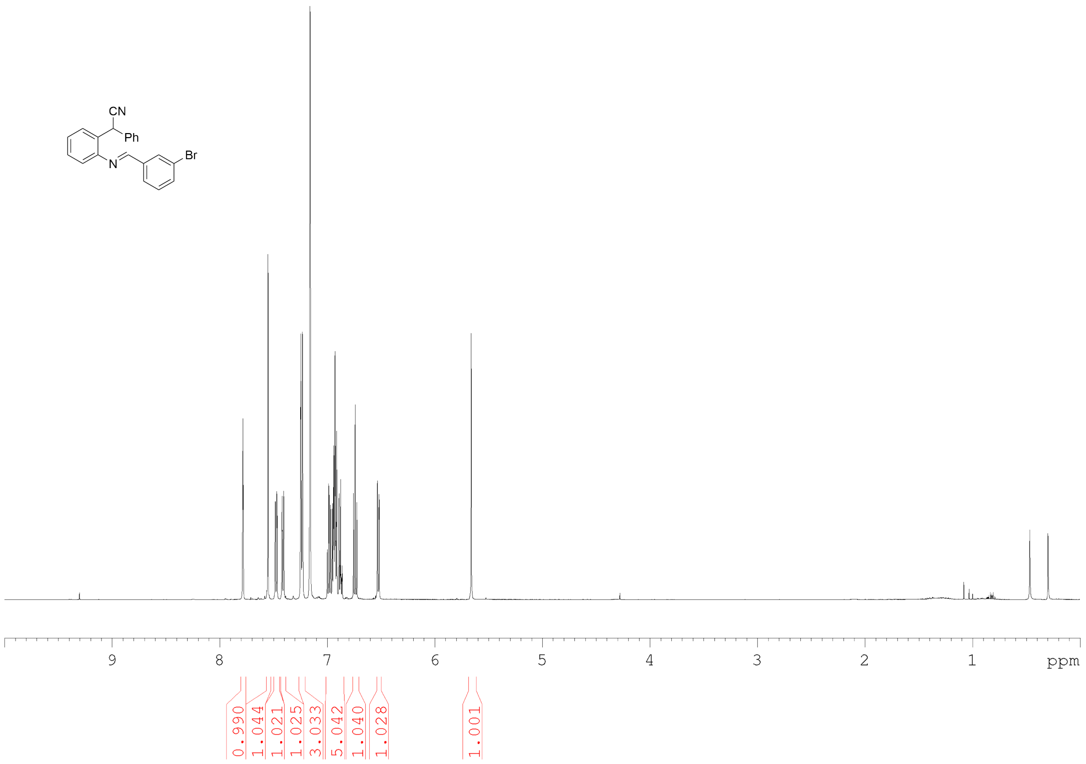
CN
Ph
N
Br
9
8
7
6
5
4
3
2
1
ppm
0.990
1.044
1.021
1.025
3.033
5.042
1.040
1.028
1.001

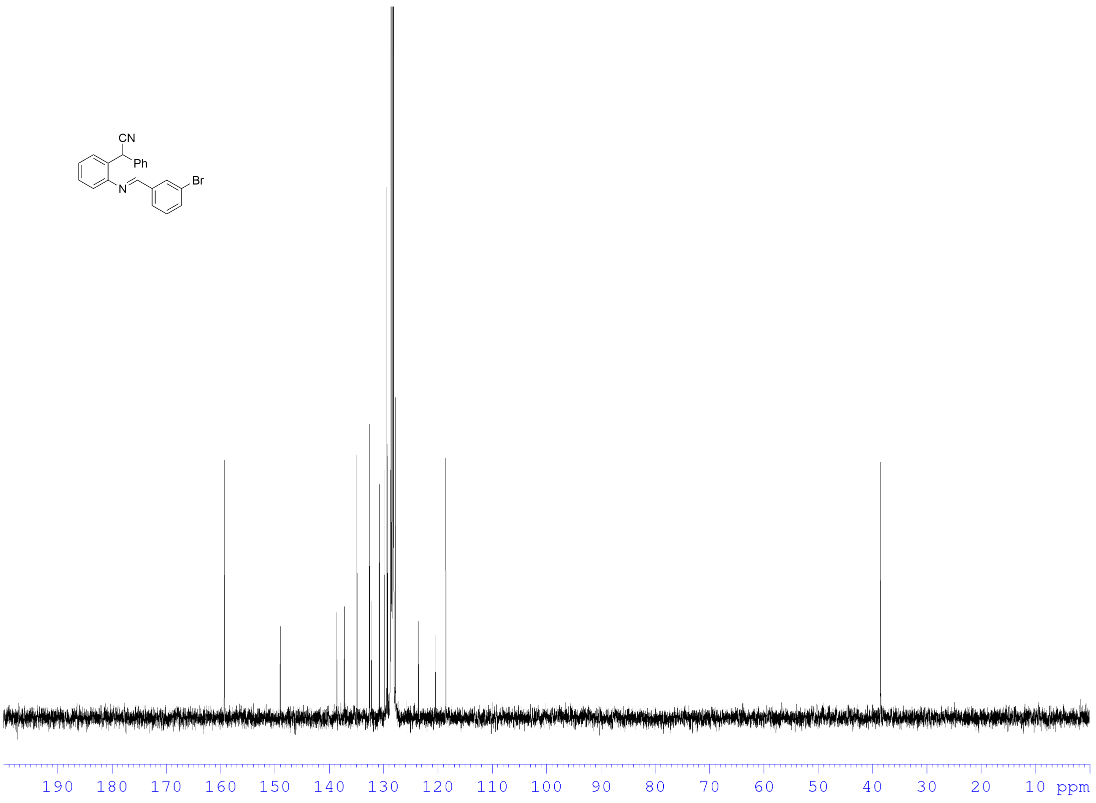
CN
Ph
N
Br
190
180
170
160
150
140
130
120
110
100
90
80
70
60
50
40
30
20
10
ppm

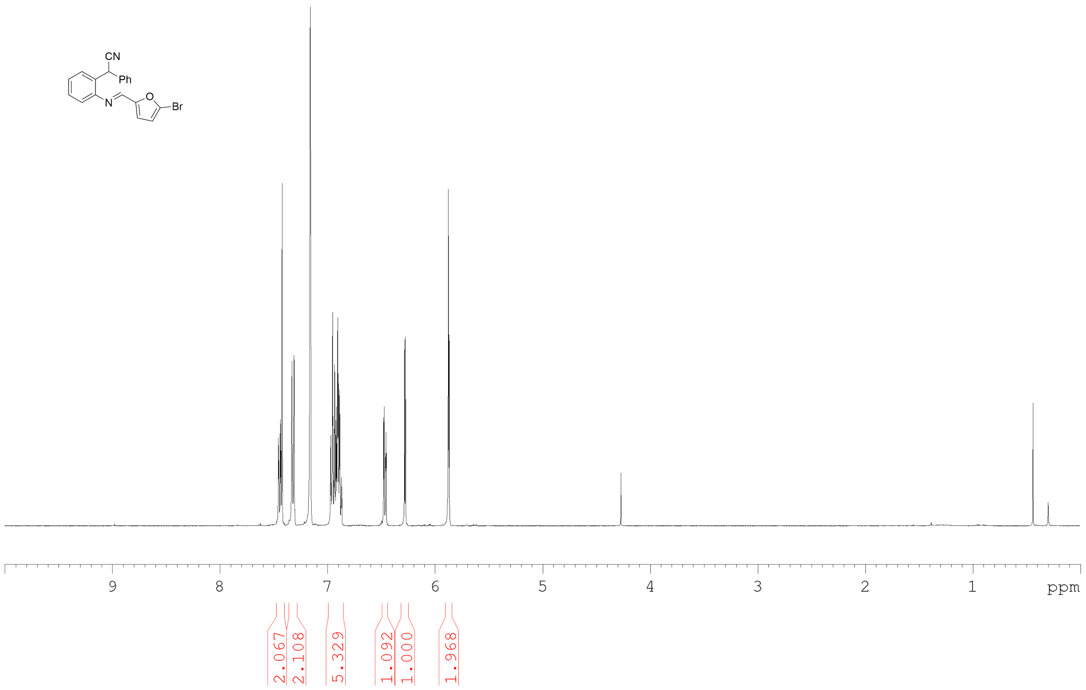
CN
Ph
N
O
Br
9 8 7 6 5 4 3 2 1 ppm
2.067
2.108
5.329
1.092
1.000
1.968

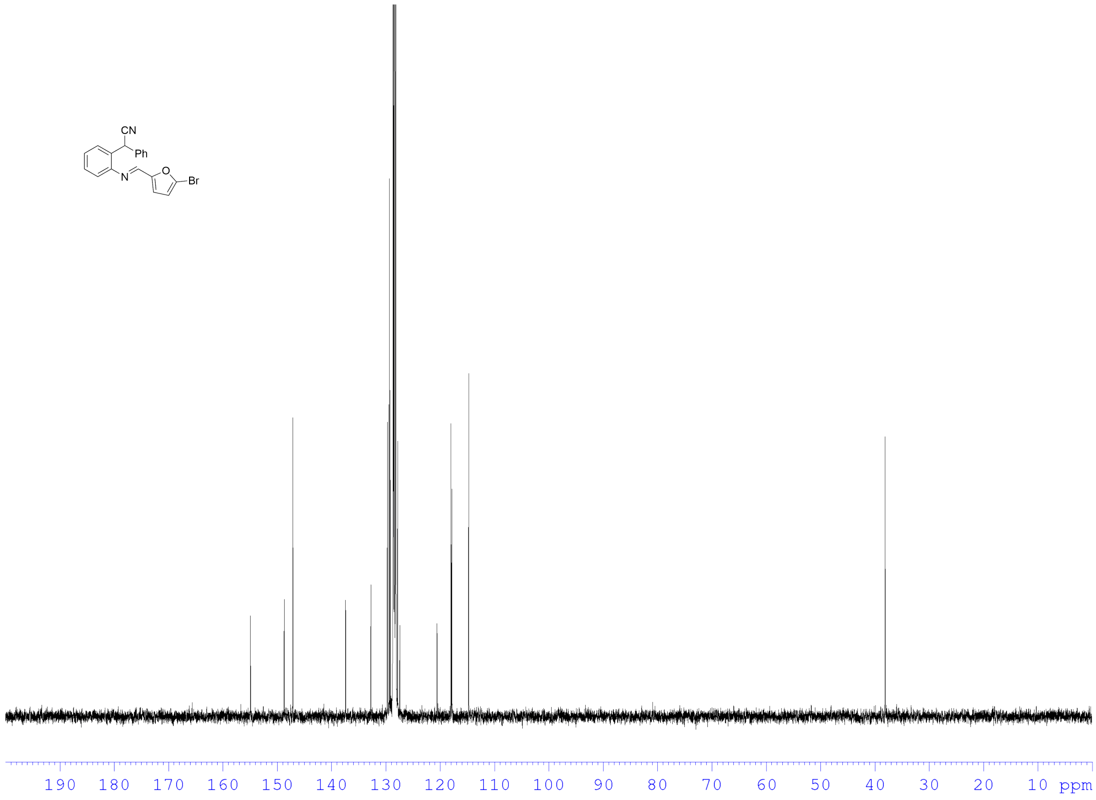
CN
Ph
N
O
Br
190 180 170 160 150 140 130 120 110 100 90 80 70 60 50 40 30 20 10 ppm

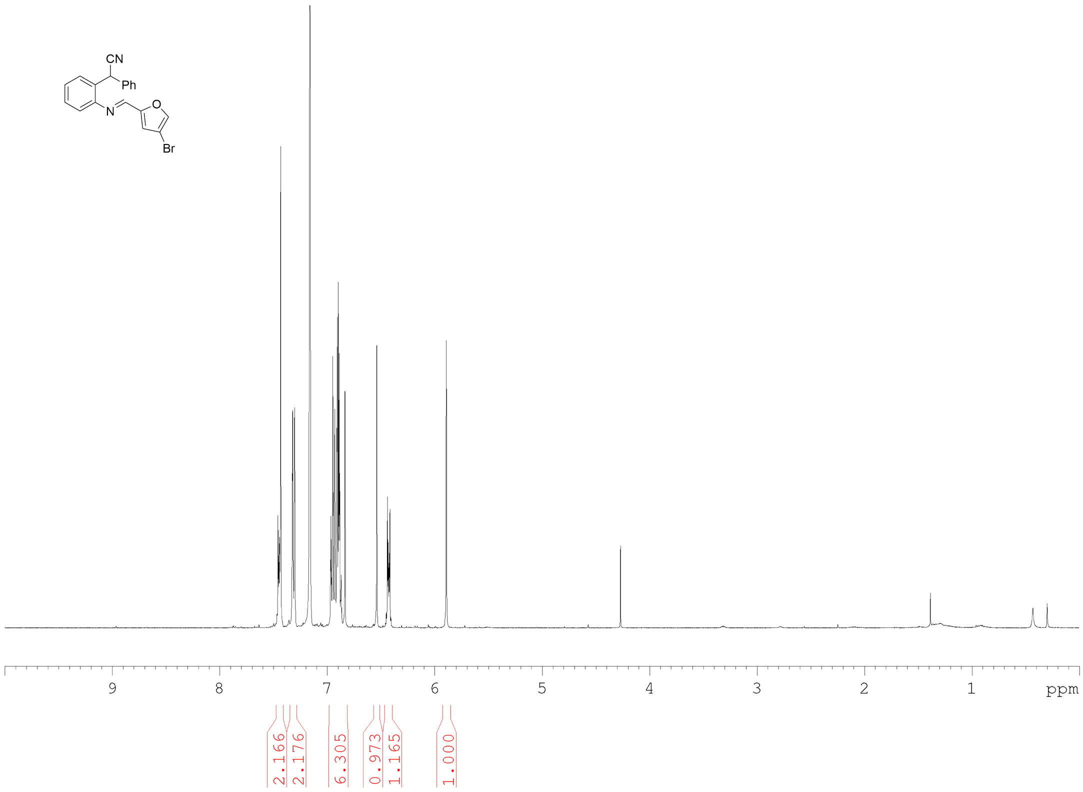
CN
Ph
N
O
Br
9
8
7
6
5
4
3
2
1
ppm
2.166
2.176
6.305
0.973
1.165
1.000

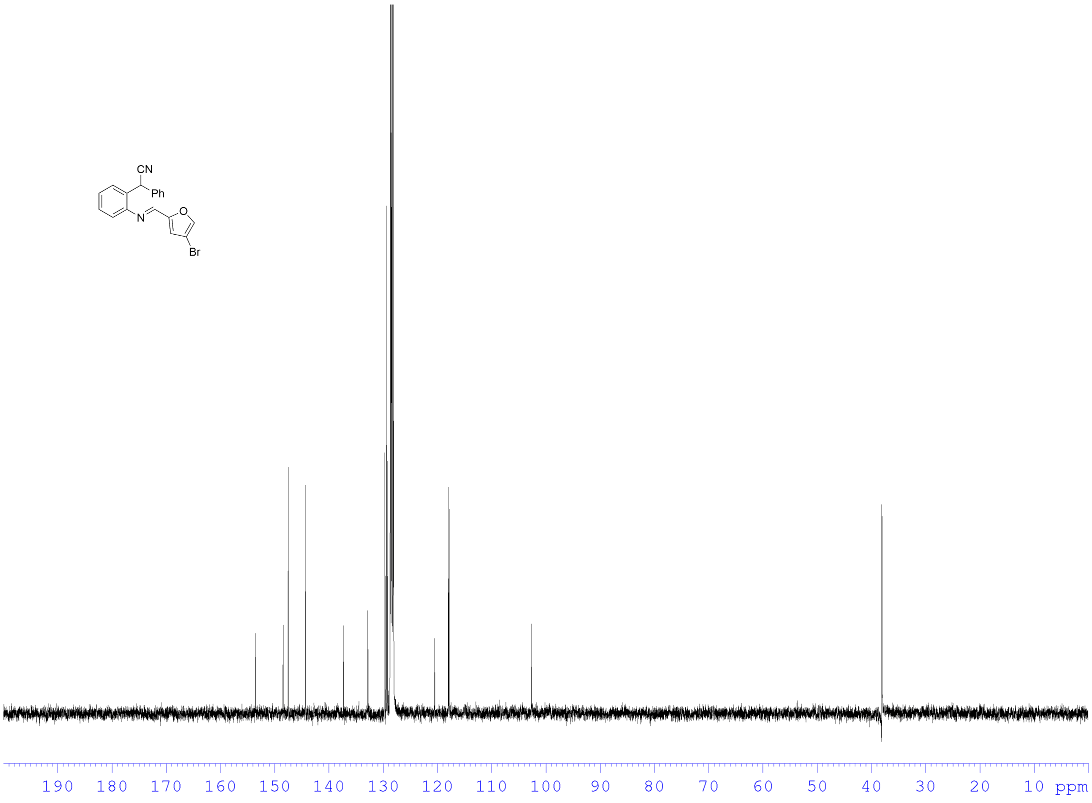
CN
Ph
N
O
Br
190
180
170
160
150
140
130
120
110
100
90
80
70
60
50
40
30
20
10
ppm

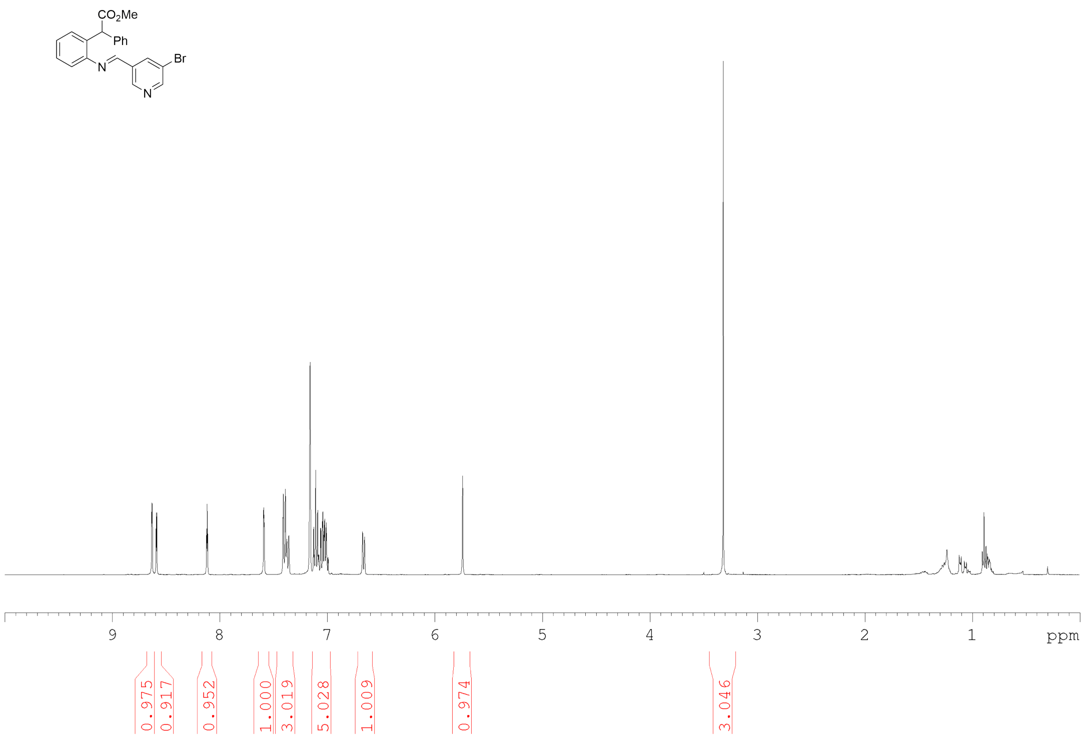
CO2Me
Ph
N
Br
N
9
8
7
6
5
4
3
2
1
ppm
0.975
0.917
0.952
1.000
3.019
5.028
1.009
0.974
3.046

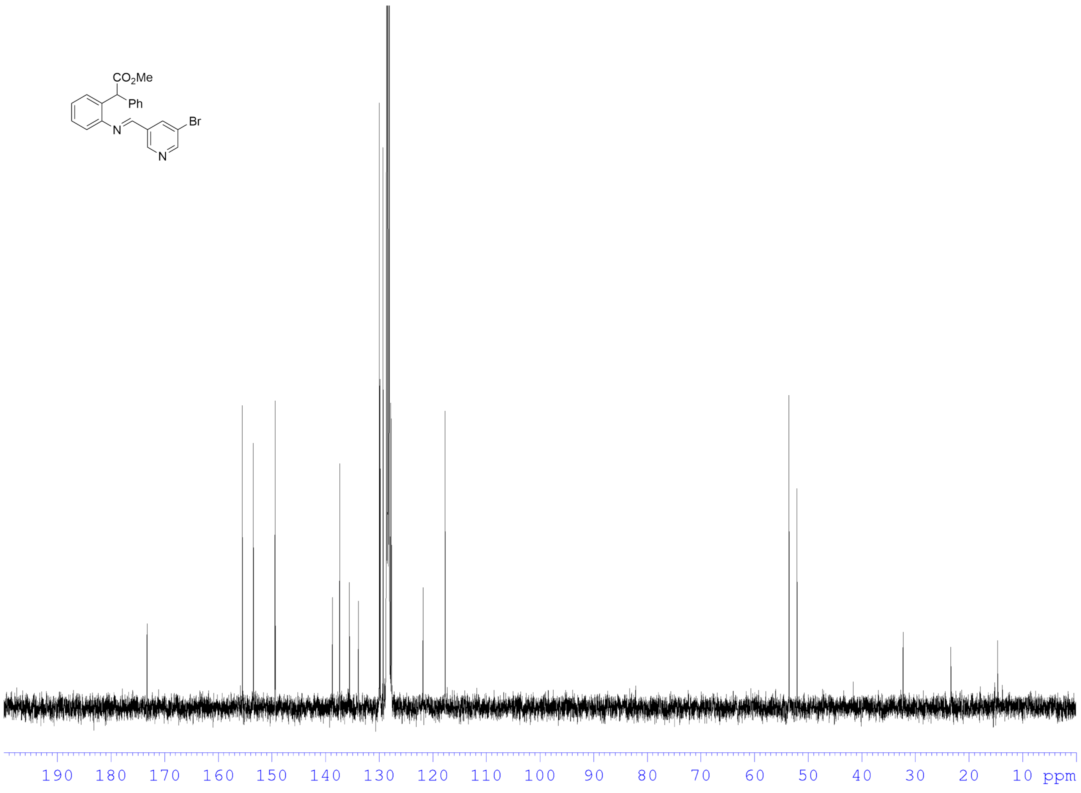
CO2Me
Ph
N
Br
N
190
180
170
160
150
140
130
120
110
100
90
80
70
60
50
40
30
20
10
ppm

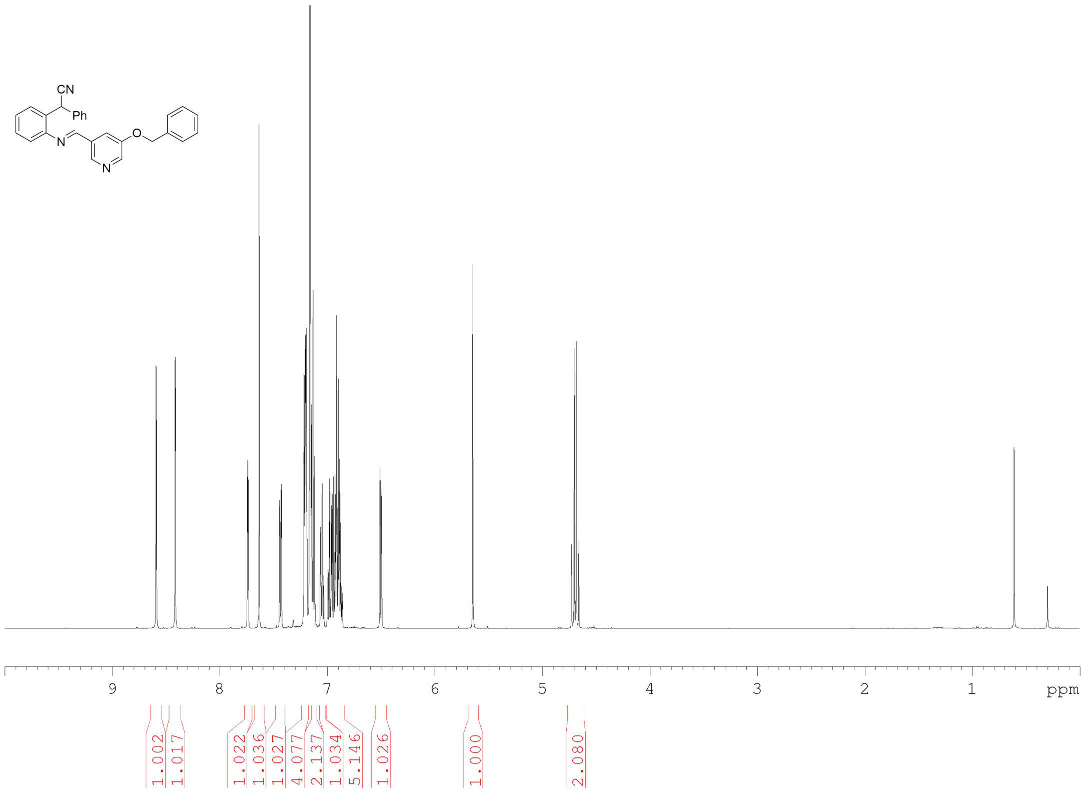
CN
Ph
N
O
N
9 8 7 6 5 4 3 2 1 ppm
1.002
1.017
1.022
1.036
1.027
4.077
2.137
1.034
5.146
1.026
1.000
2.080

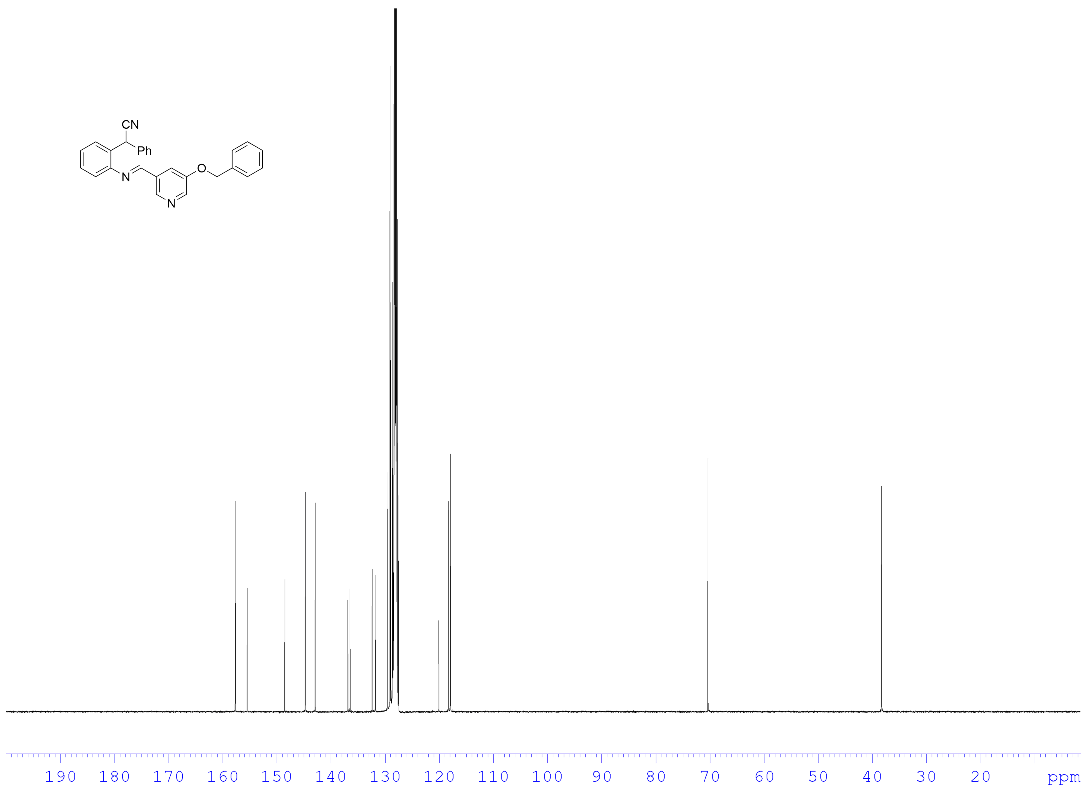
CN
Ph
N
O
N
190 180 170 160 150 140 130 120 110 100 90 80 70 60 50 40 30 20 ppm

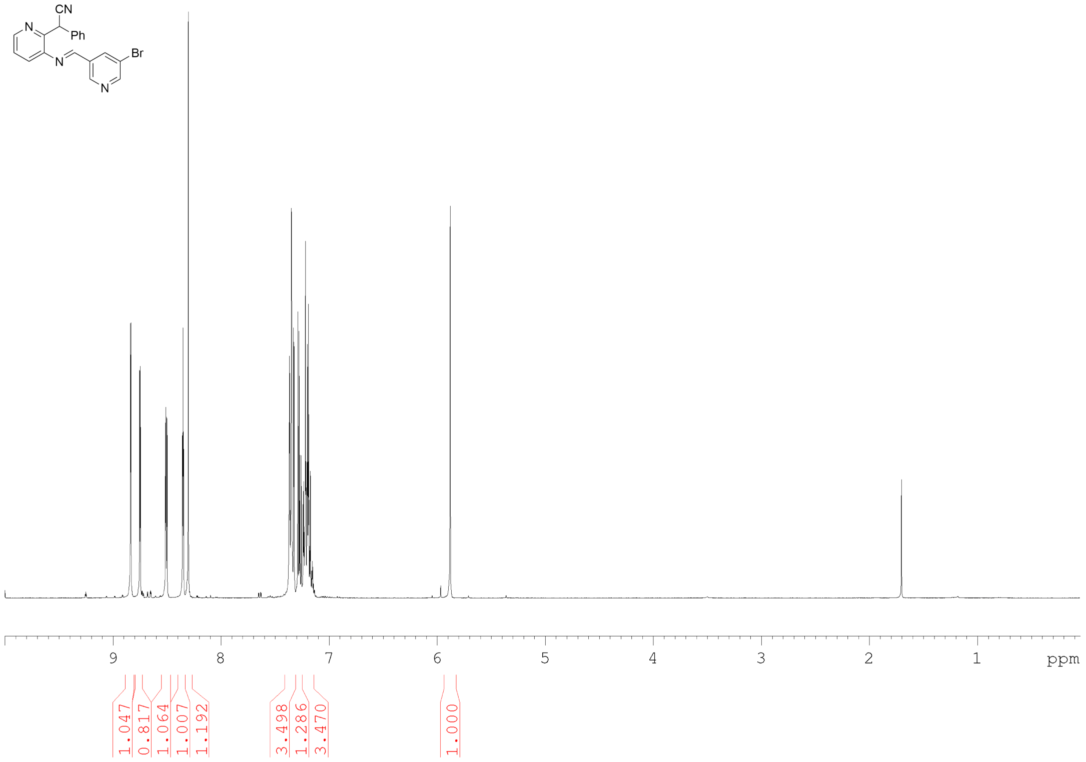
CN
Ph
N
N
Br
N
9 8 7 6 5 4 3 2 1 ppm
1.047
0.817
1.064
1.007
1.192
3.498
1.286
3.470
1.000

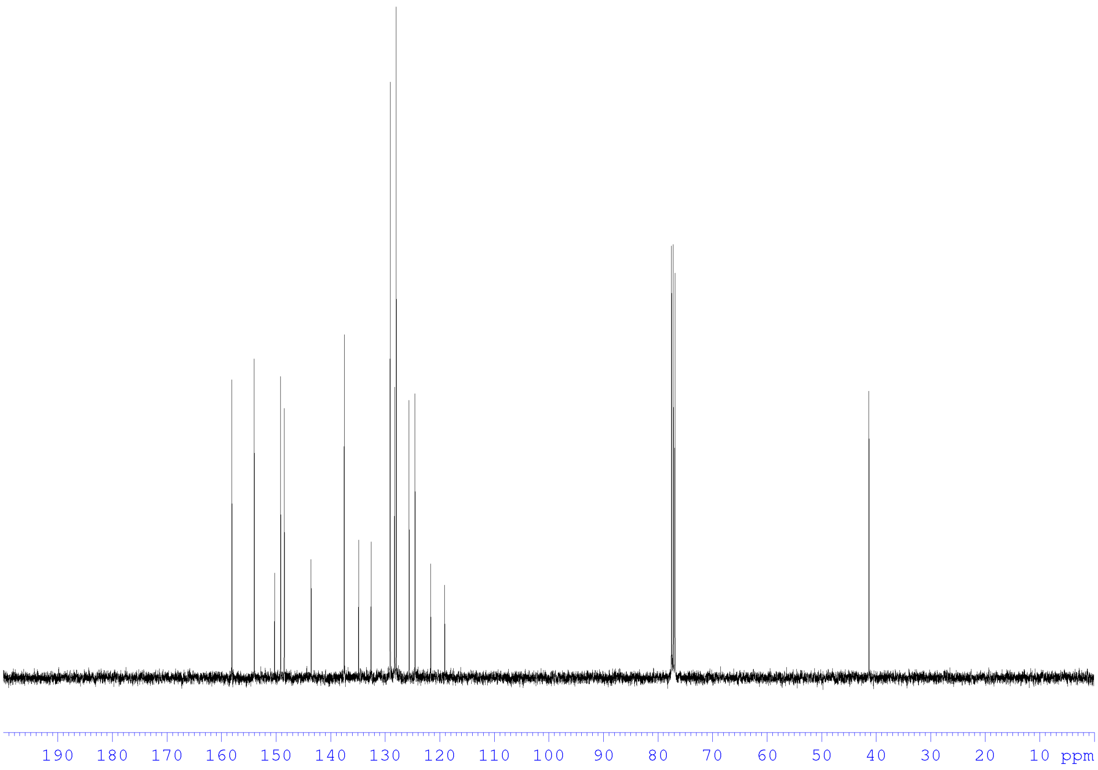
190 180 170 160 150 140 130 120 110 100 90 80 70 60 50 40 30 20 10 ppm

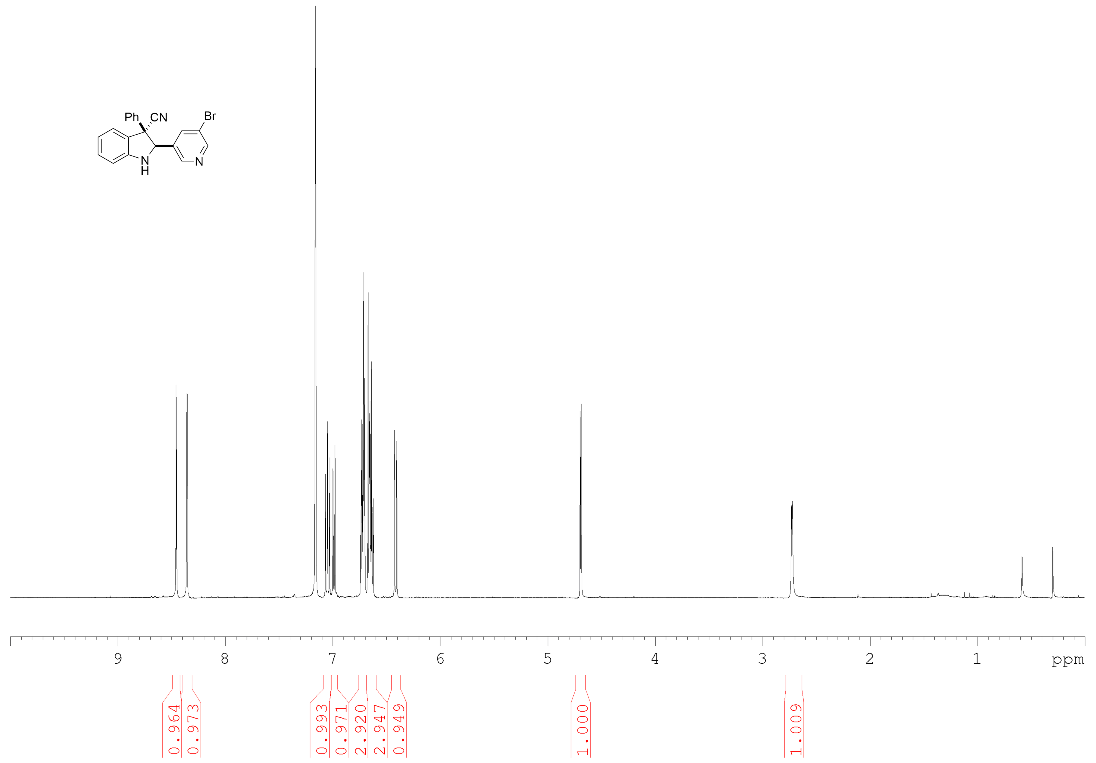
Ph
CN
Br
N
H
N
9
8
7
6
5
4
3
2
1
ppm
0.964
0.973
0.993
0.971
2.920
2.947
0.949
1.000
1.009

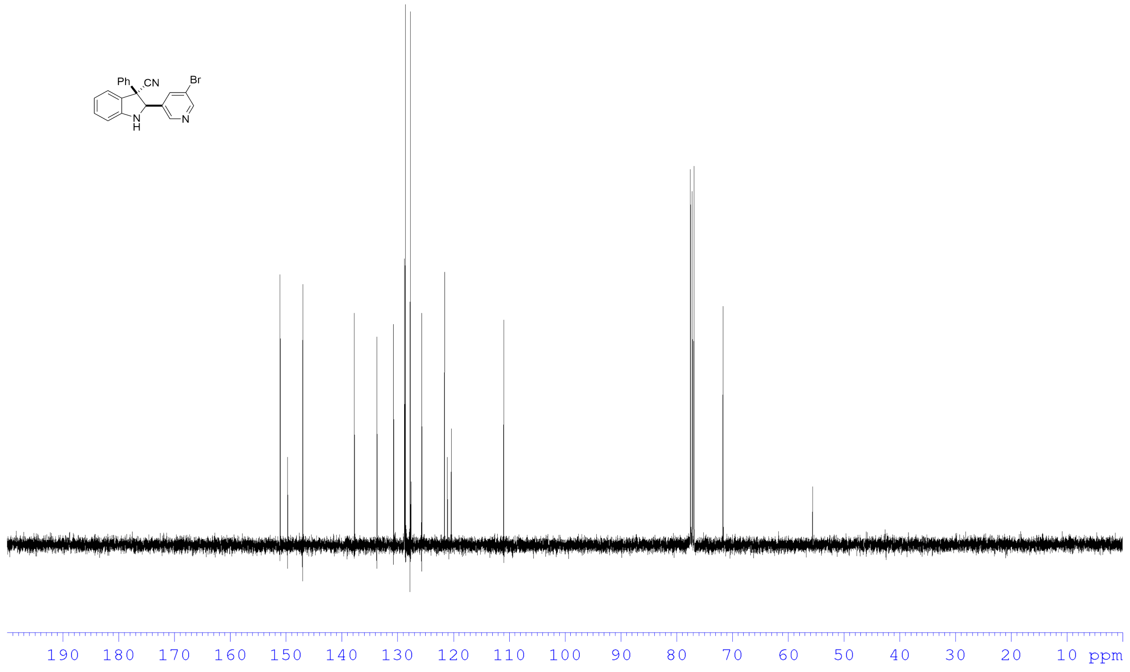
Ph
CN
Br
N
H
N
190
180
170
160
150
140
130
120
110
100
90
80
70
60
50
40
30
20
10
ppm

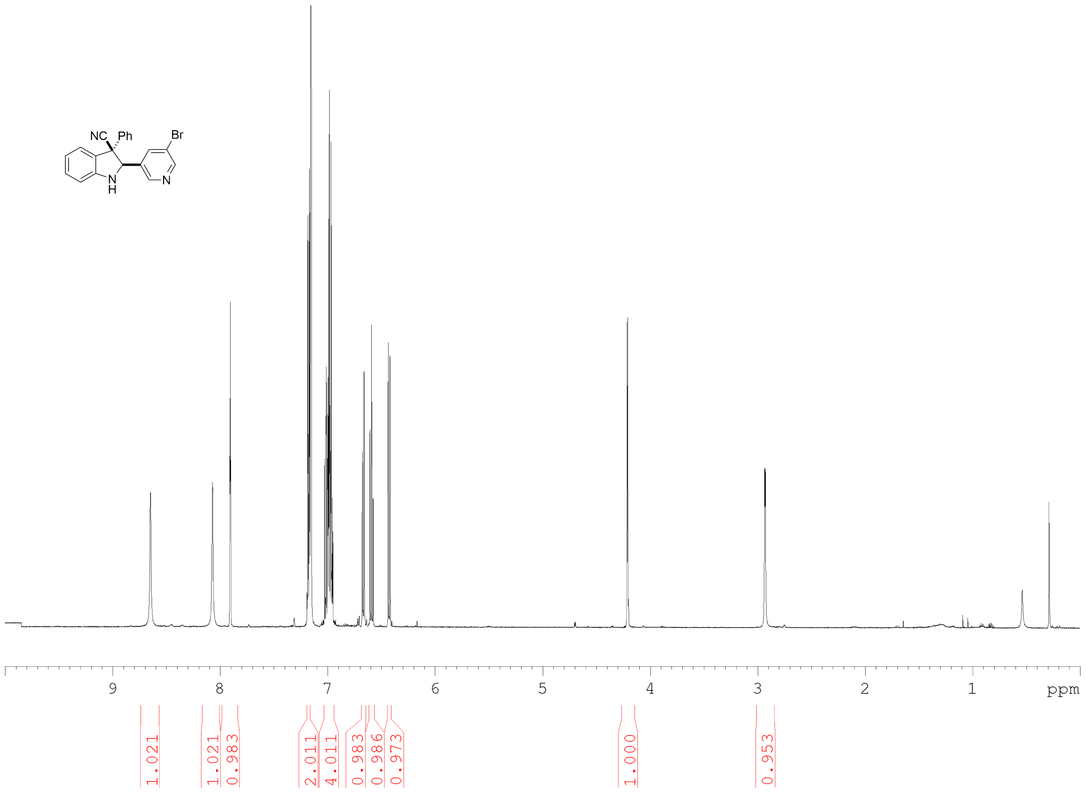

NC
Ph
Br
N
H
N
9
8
7
6
5
4
3
2
1
ppm
1.021
1.021
0.983
2.011
4.011
0.983
0.986
0.973
1.000
0.953

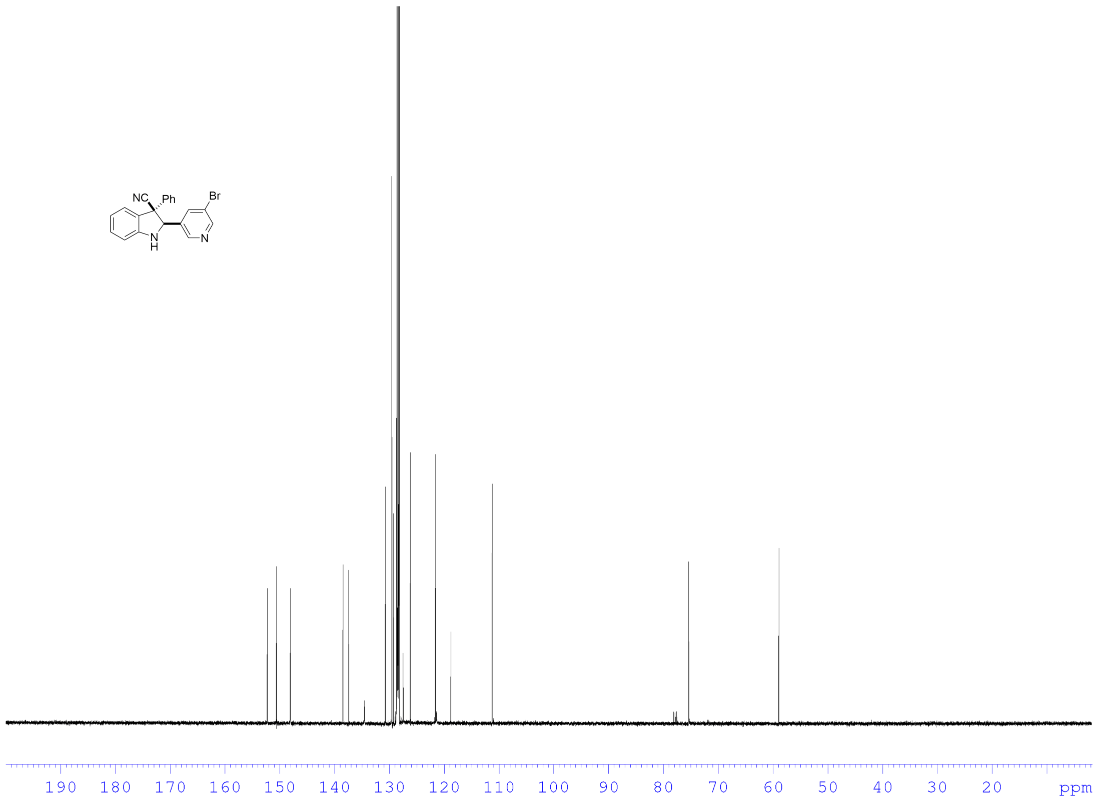

NC
Ph
Br
N
H
N
190
180
170
160
150
140
130
120
110
100
90
80
70
60
50
40
30
20
ppm

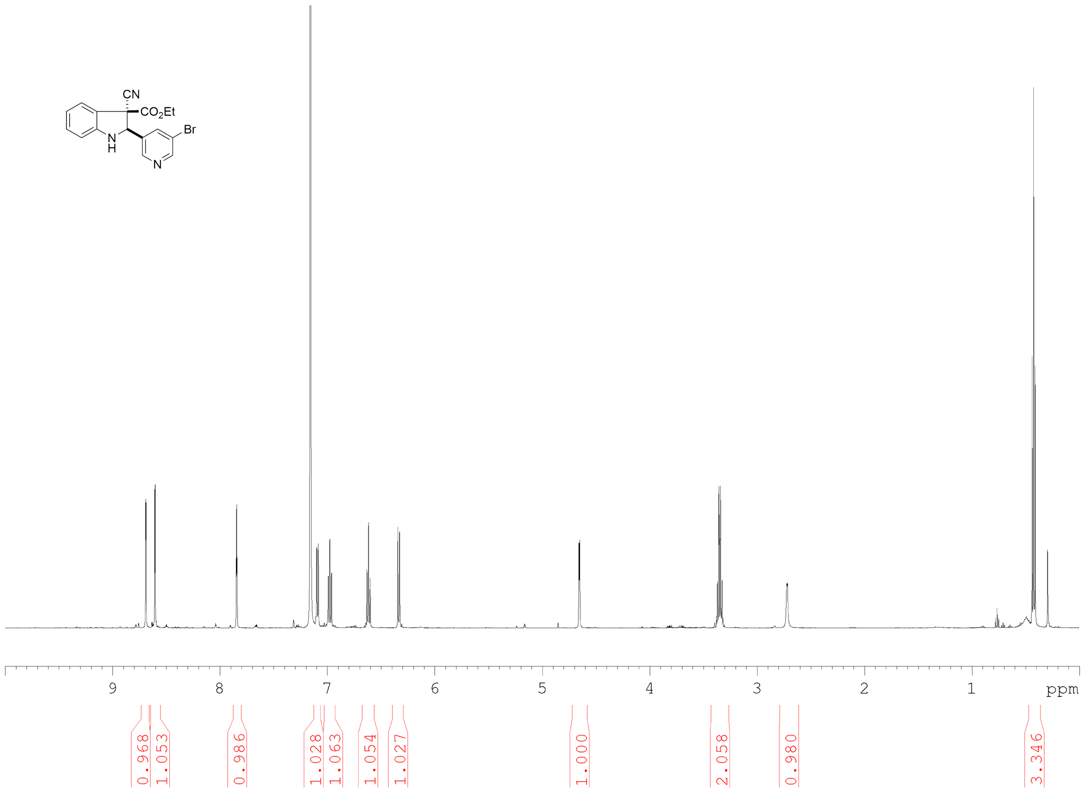
CN
CO2Et
Br
N
H
9 8 7 6 5 4 3 2 1 ppm
0.968
1.053
0.986
1.028
1.063
1.054
1.027
1.000
2.058
0.980
3.346

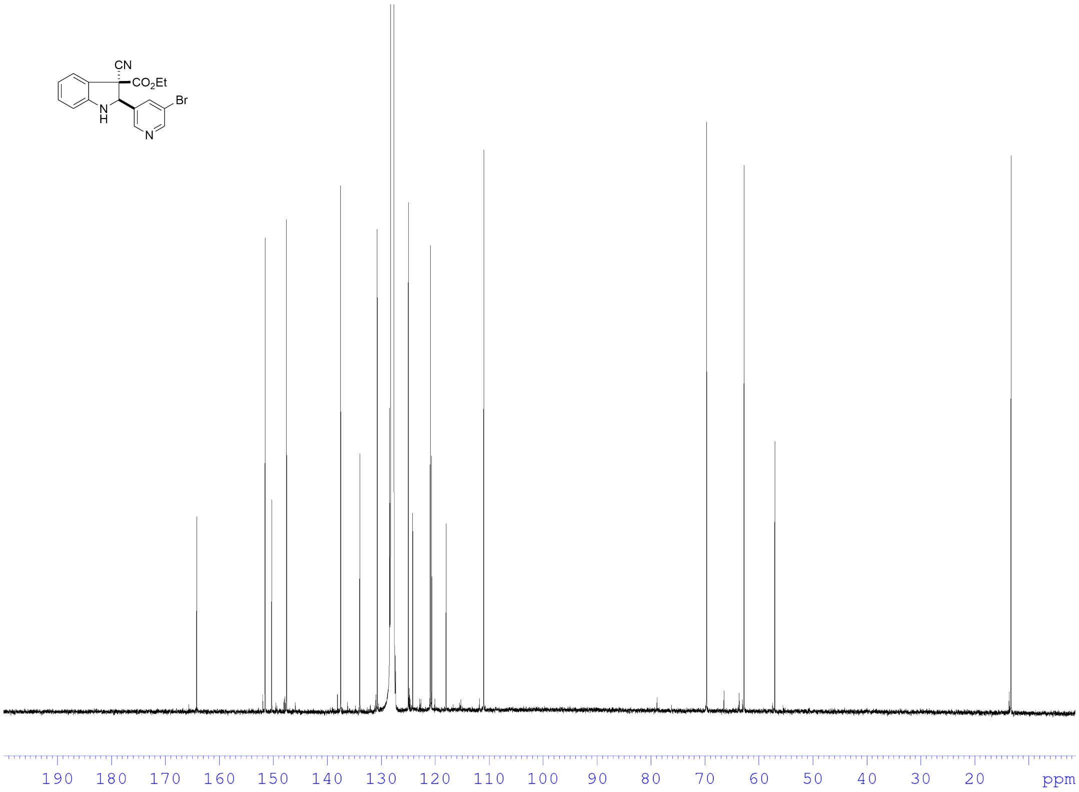
CN
CO2Et
Br
N
H
190 180 170 160 150 140 130 120 110 100 90 80 70 60 50 40 30 20 ppm

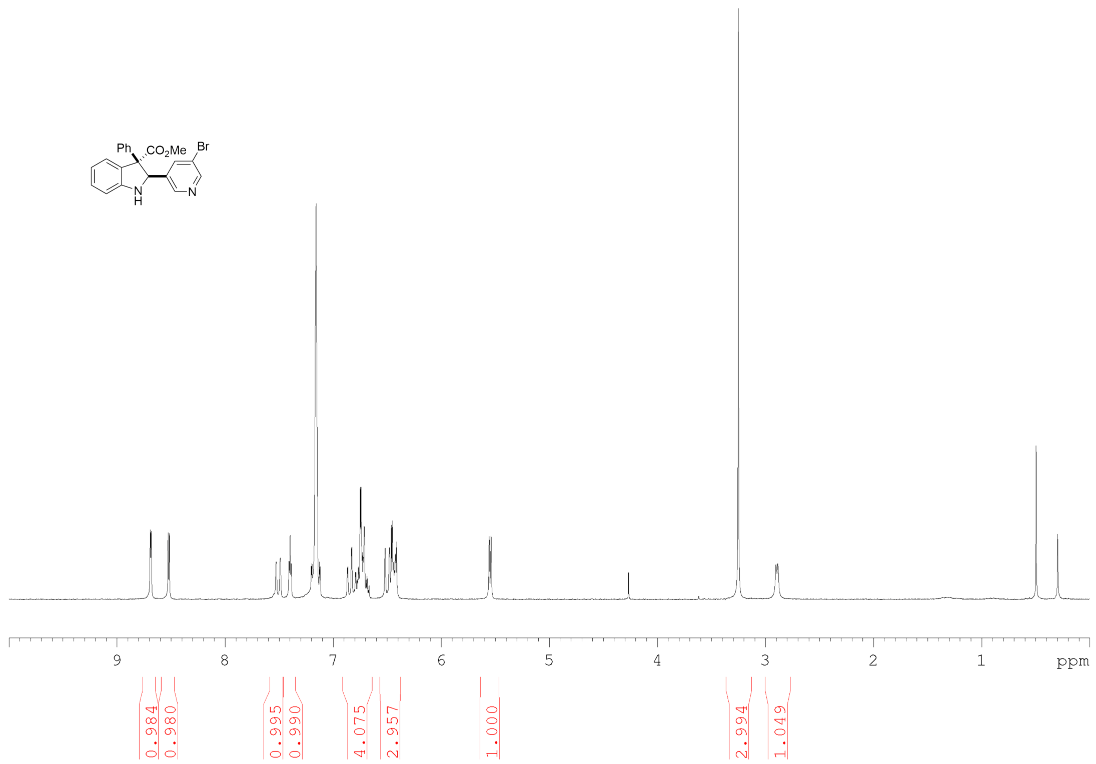

Ph
CO2Me
Br
N
H
N
9
8
7
6
5
4
3
2
1
ppm
0.984
0.980
0.995
0.990
4.075
2.957
1.000
2.994
1.049

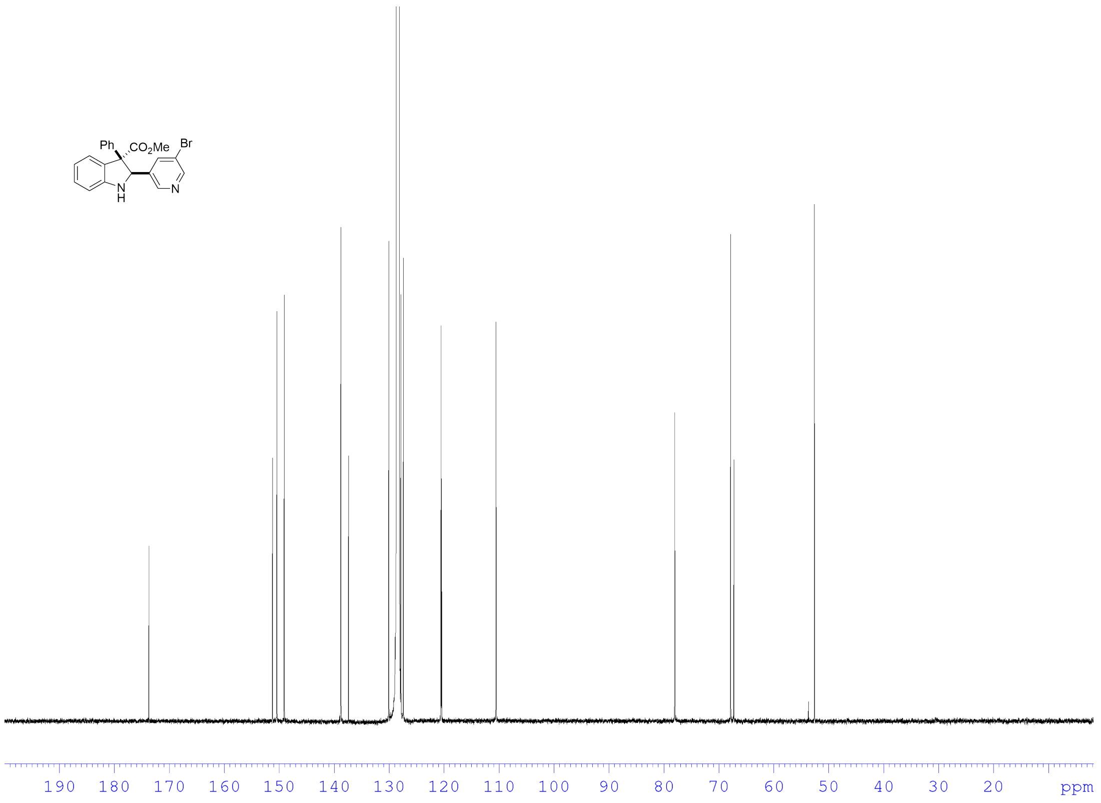

Ph
CO2Me
Br
N
H
N
190
180
170
160
150
140
130
120
110
100
90
80
70
60
50
40
30
20
ppm

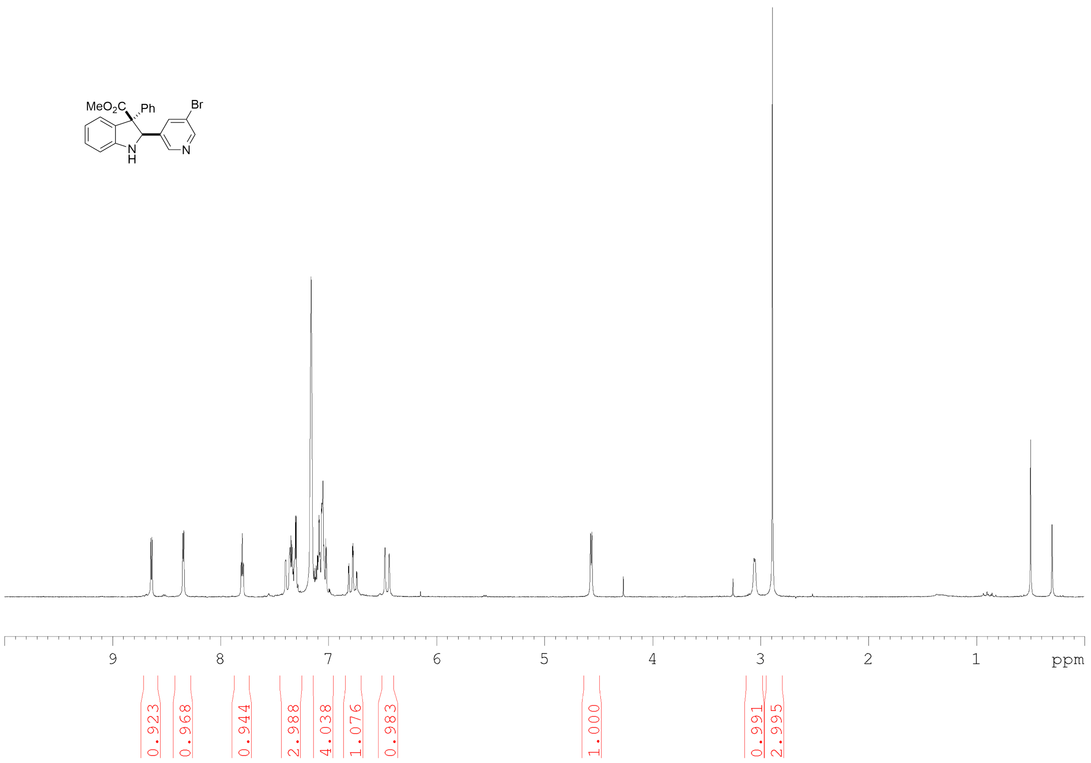

MeO2C
Ph
Br
N
H
N
9
8
7
6
5
4
3
2
1
ppm
0.923
0.968
0.944
2.988
4.038
1.076
0.983
1.000
0.991
2.995

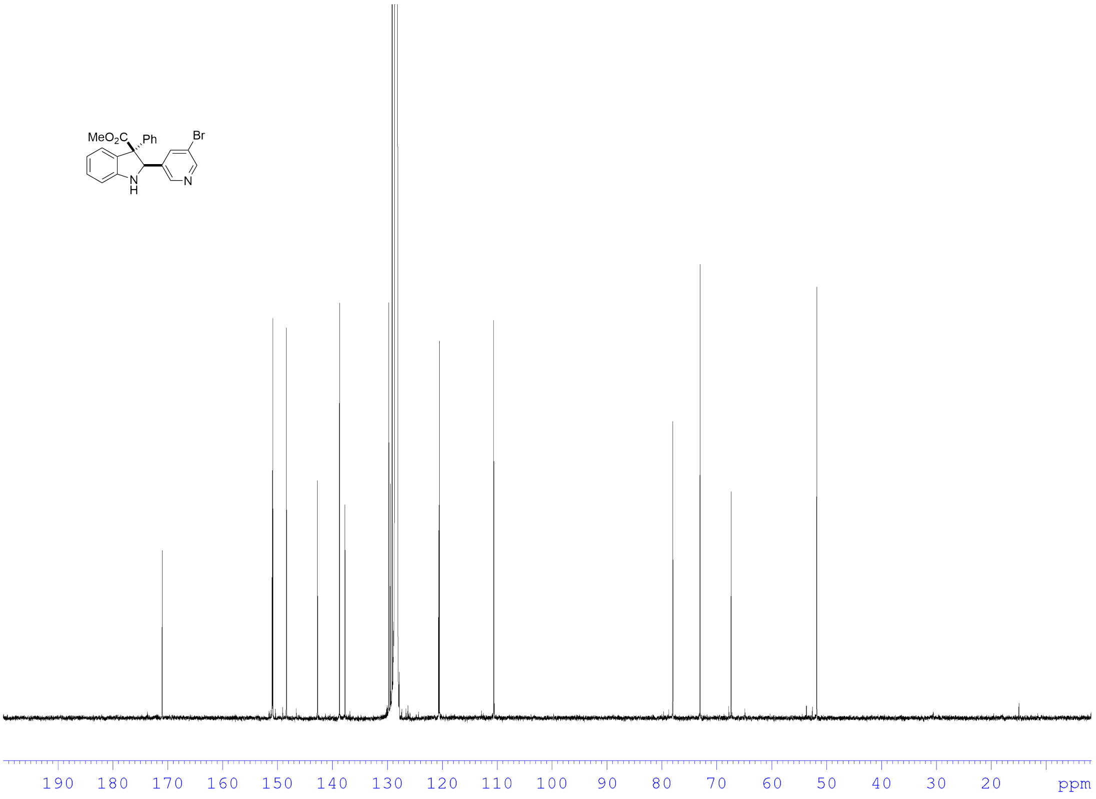

MeO2C
Ph
Br
N
H
N
190
180
170
160
150
140
130
120
110
100
90
80
70
60
50
40
30
20
ppm

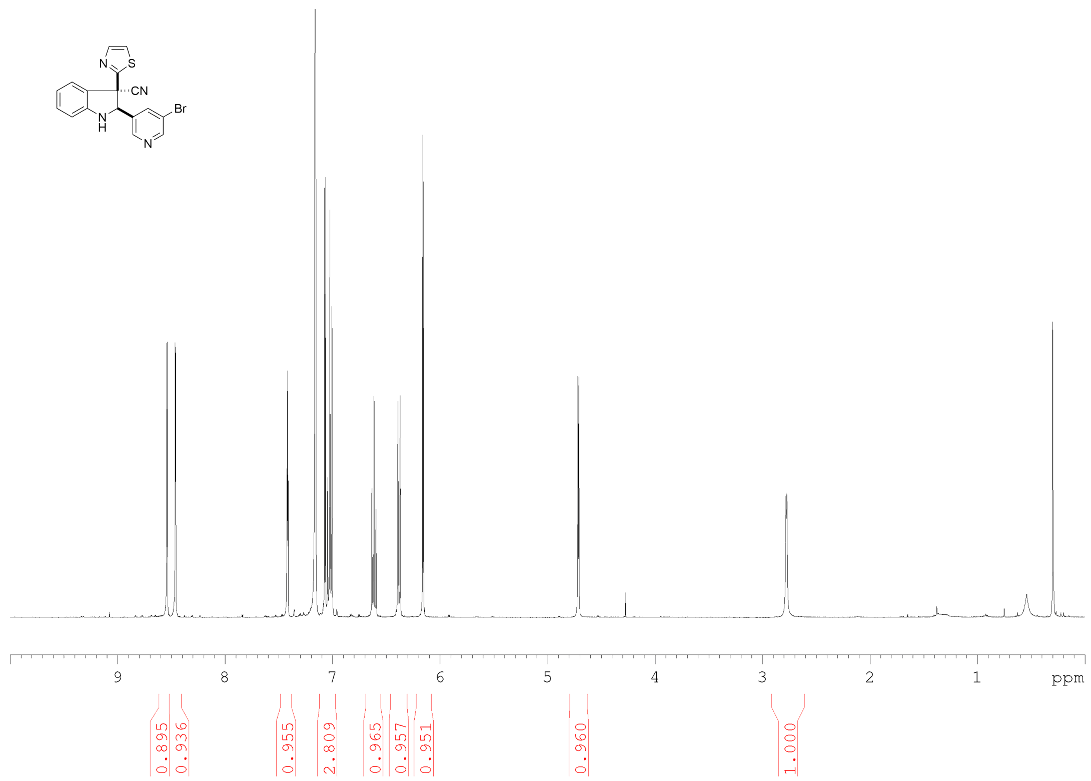
CN
Br
N
H
N
S
9
8
7
6
5
4
3
2
1
ppm
0.895
0.936
0.955
2.809
0.965
0.957
0.951
0.960
1.000

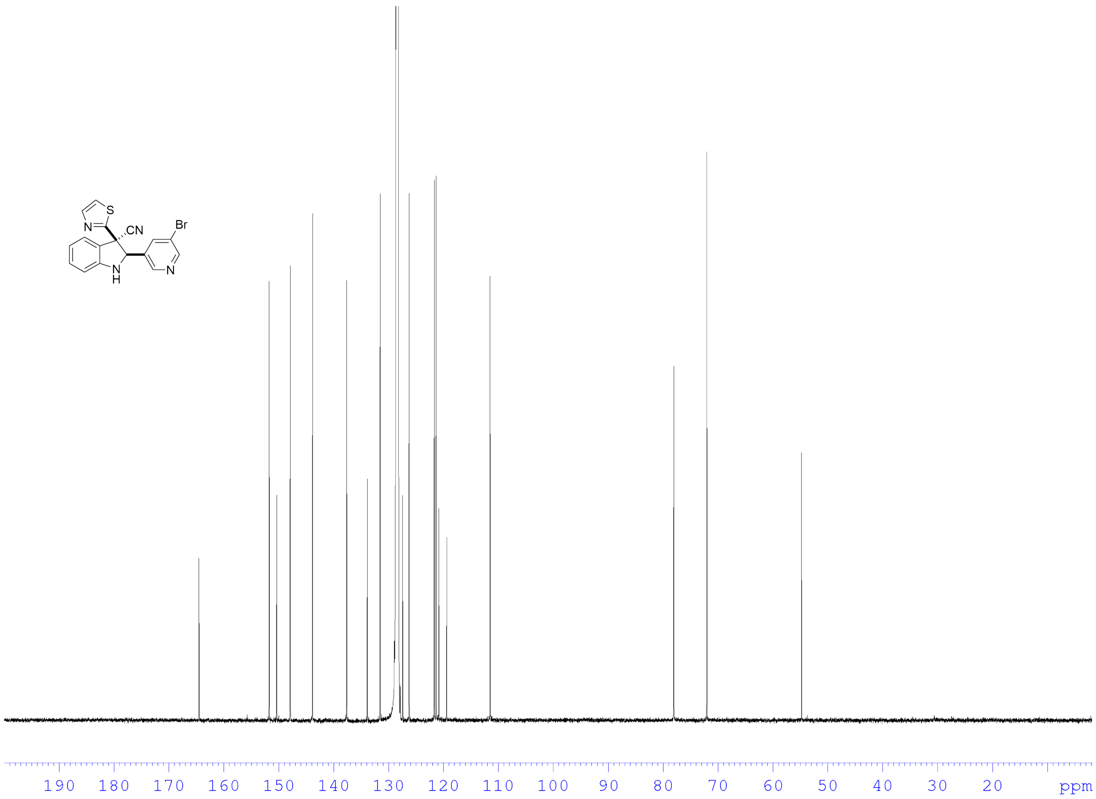
CN
Br
N
H
N
S
190
180
170
160
150
140
130
120
110
100
90
80
70
60
50
40
30
20
ppm

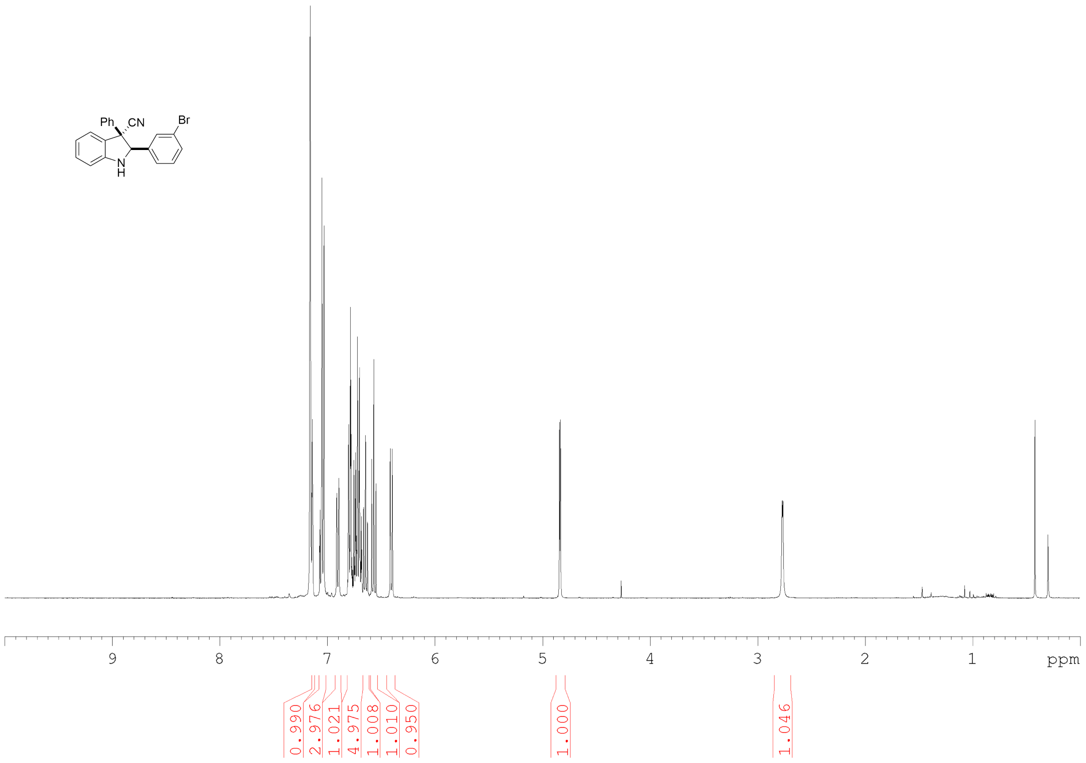
Ph
CN
Br
N
H
9
8
7
6
5
4
3
2
1
ppm
0.990
2.976
1.021
4.975
1.008
1.010
0.950
1.000
1.046

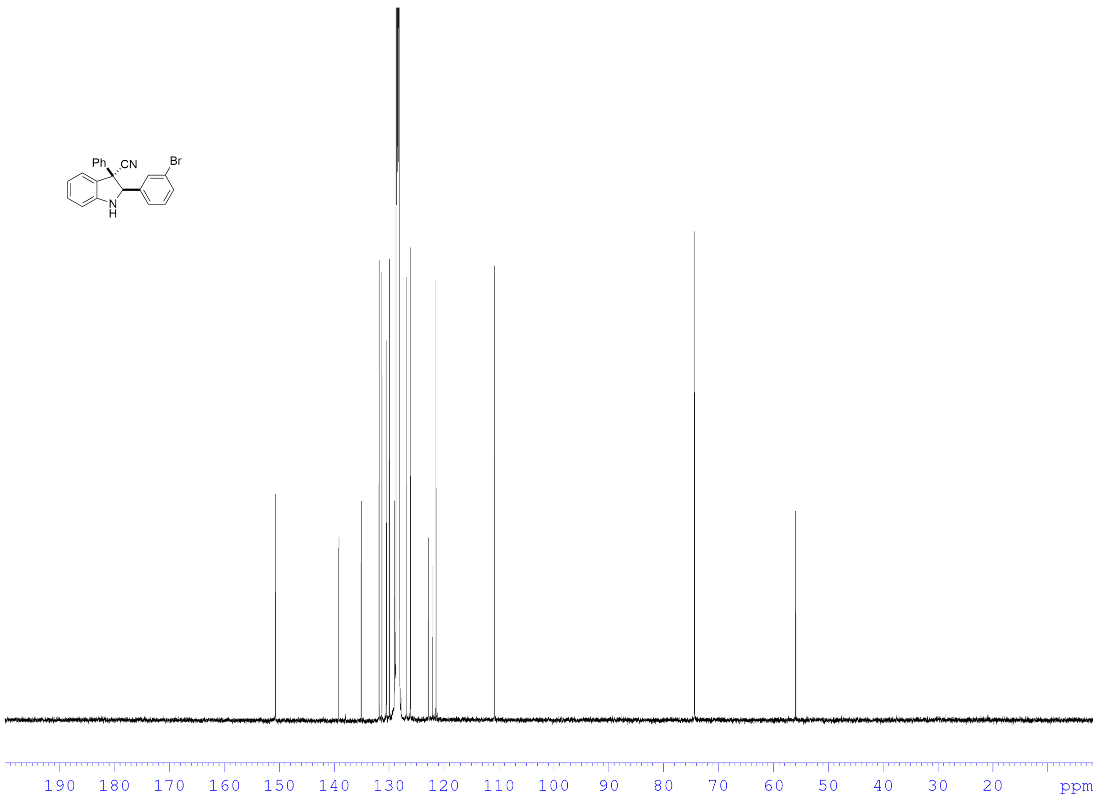
Ph
CN
Br
N
H
190
180
170
160
150
140
130
120
110
100
90
80
70
60
50
40
30
20
ppm

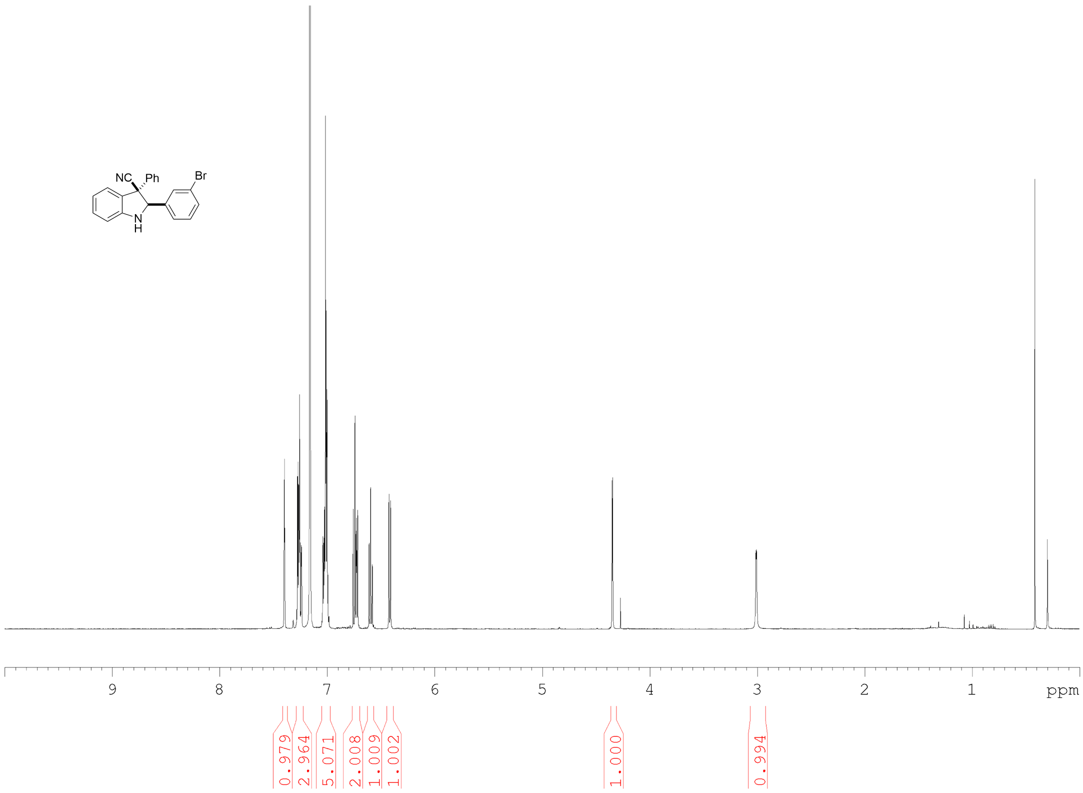
NC
Ph
Br
N
H
9 8 7 6 5 4 3 2 1 ppm
0.979 2.964 5.071 2.008 1.009 1.002 1.000 0.994

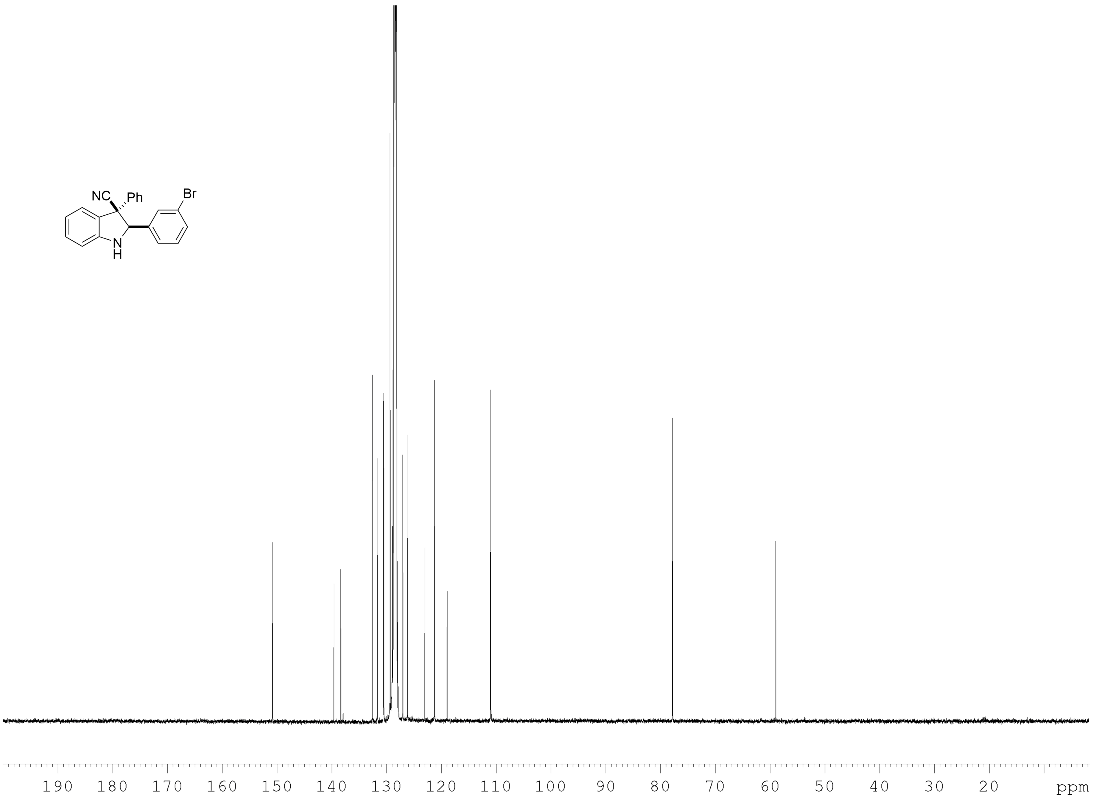
NC
Ph
Br
N
H
190 180 170 160 150 140 130 120 110 100 90 80 70 60 50 40 30 20 ppm

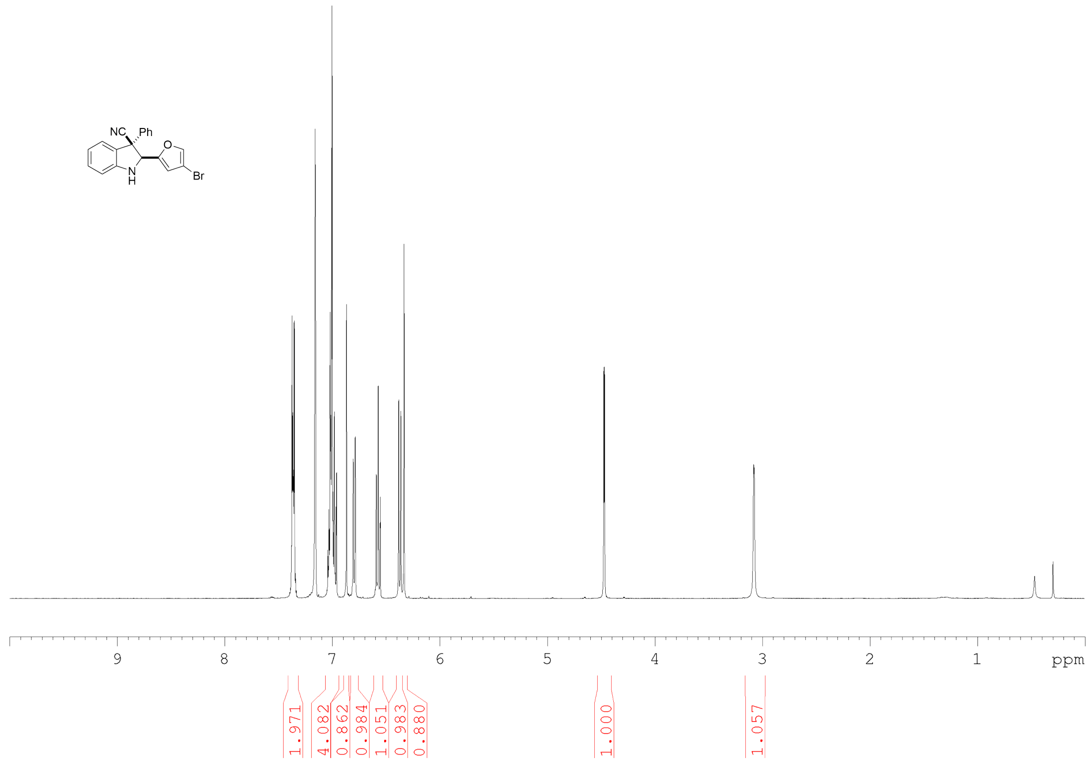
NC
Ph
O
N
H
Br
9 8 7 6 5 4 3 2 1 ppm
1.971
4.082
0.862
0.984
1.051
0.983
0.880
1.000
1.057

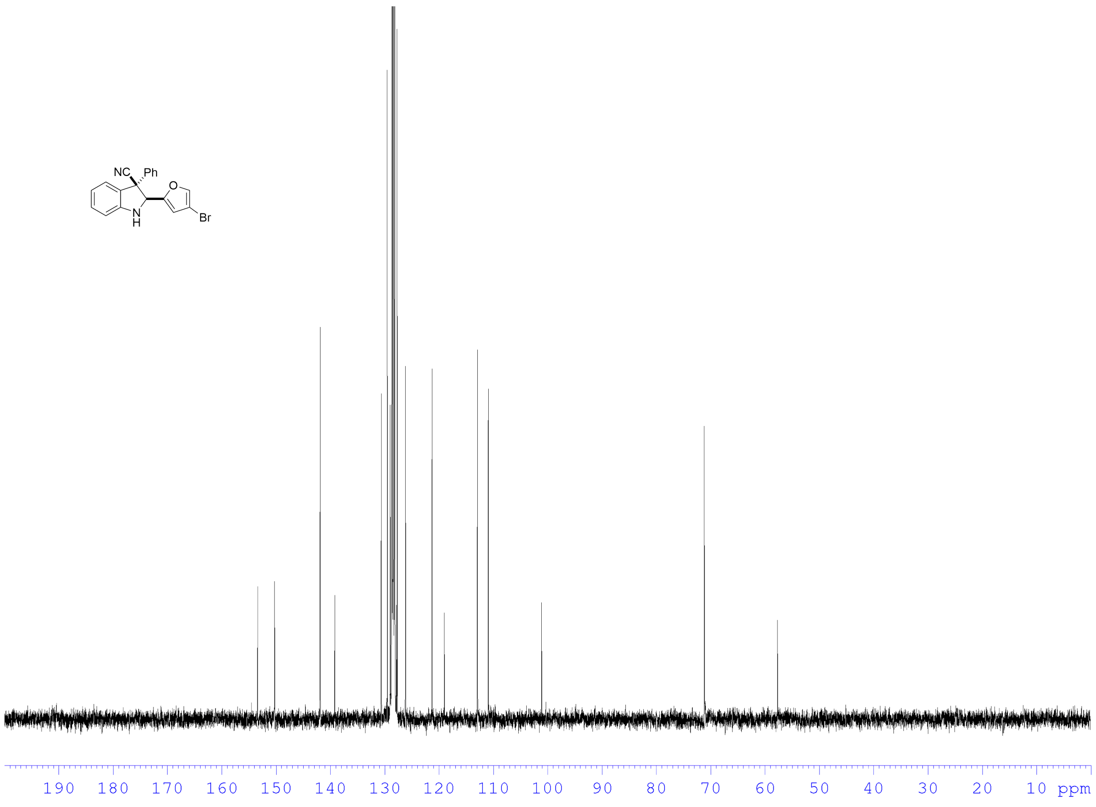
NC
Ph
O
N
H
Br
190 180 170 160 150 140 130 120 110 100 90 80 70 60 50 40 30 20 10 ppm

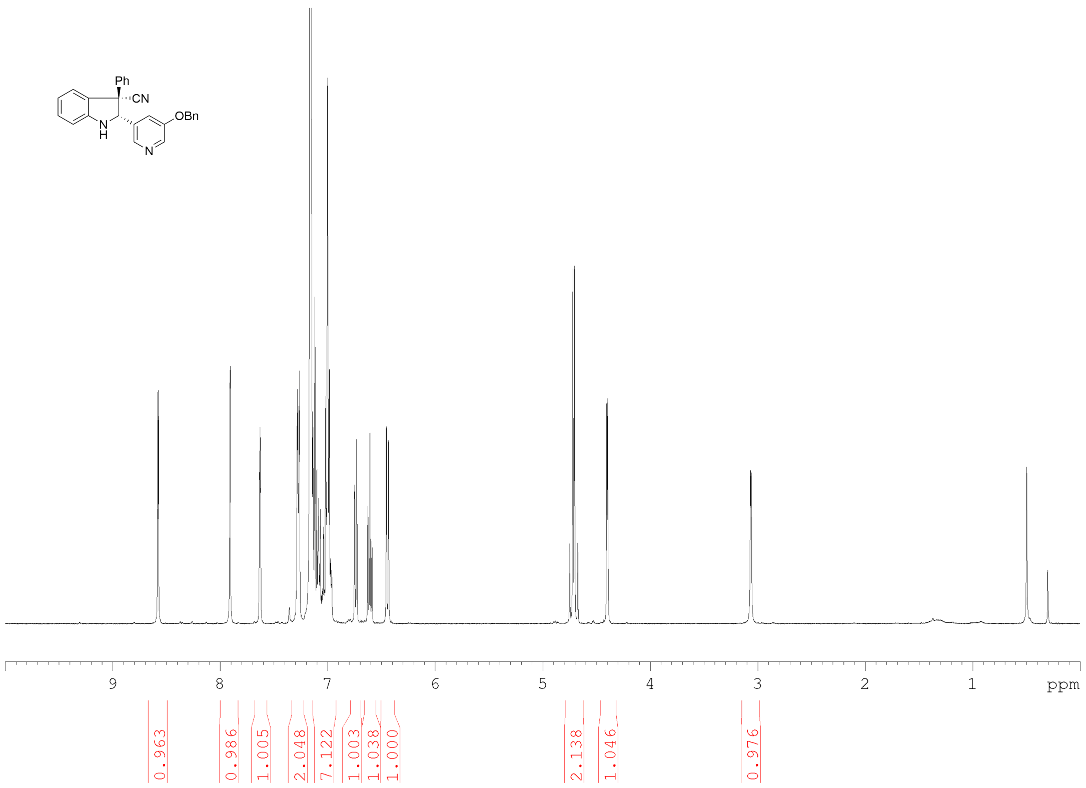
Ph
CN
N
H
N
OBn
9
8
7
6
5
4
3
2
1
ppm
0.963
0.986
1.005
2.048
7.122
1.003
1.038
1.000
2.138
1.046
0.976

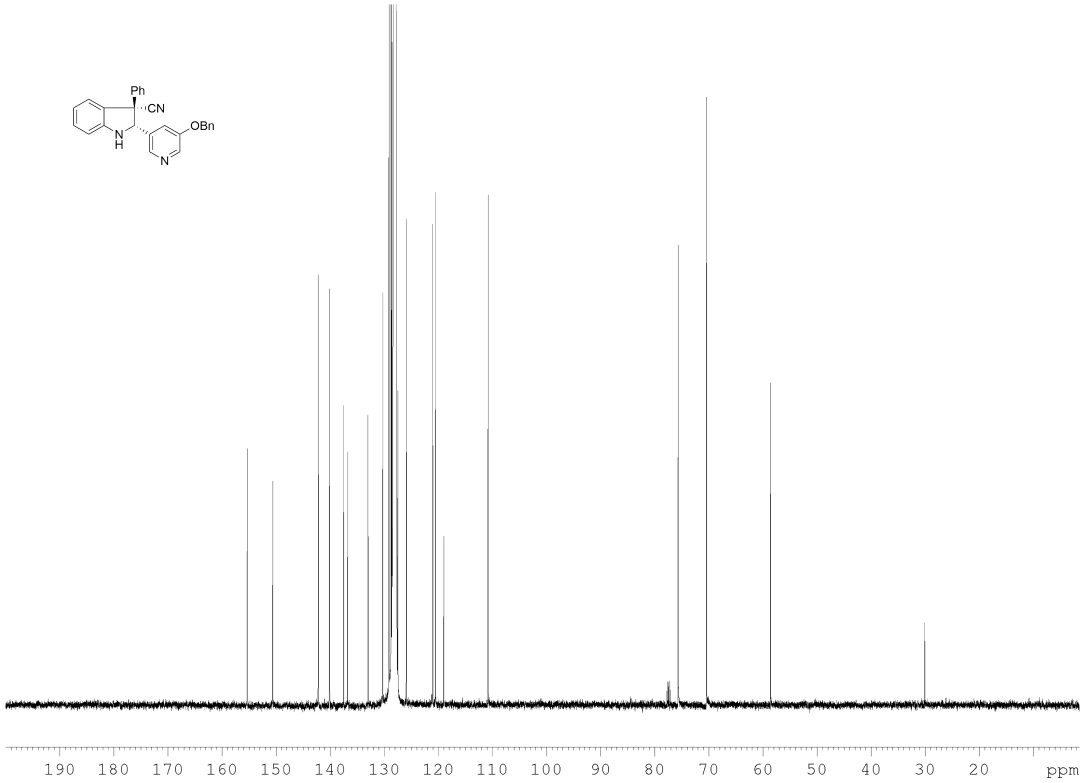
Ph
CN
N
H
N
OBn
190
180
170
160
150
140
130
120
110
100
90
80
70
60
50
40
30
20
ppm

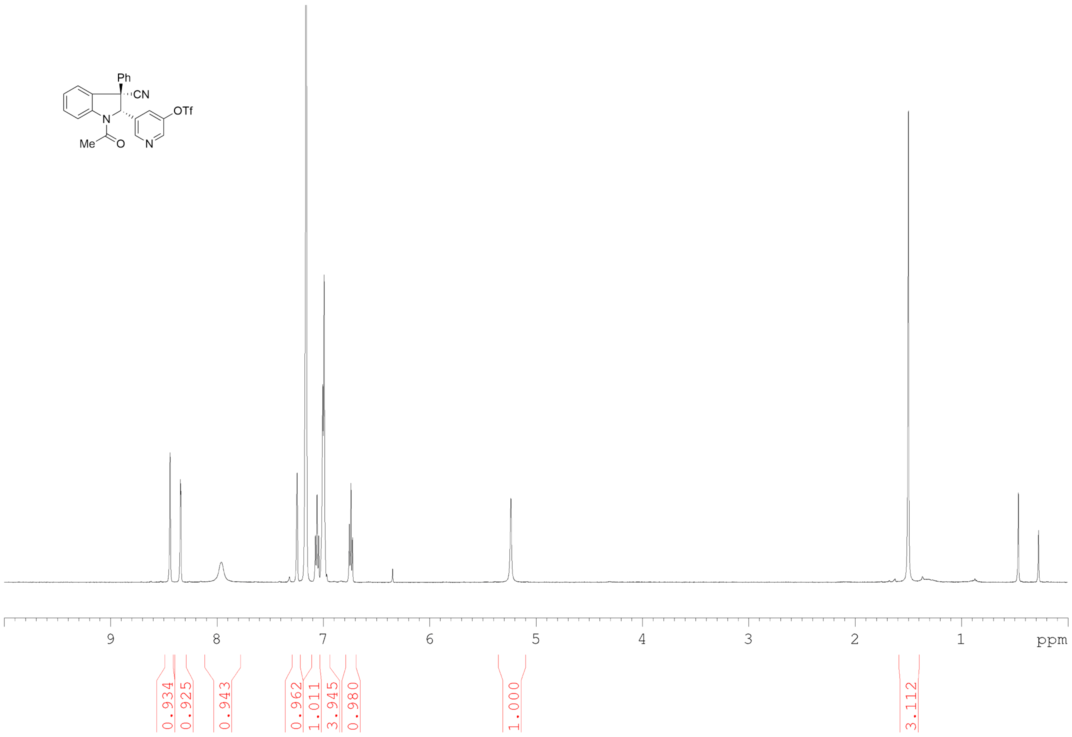
Ph
CN
OTf
N
Me
O
9
8
7
6
5
4
3
2
1
ppm
0.934
0.925
0.943
0.962
1.011
3.945
0.980
1.000
3.112

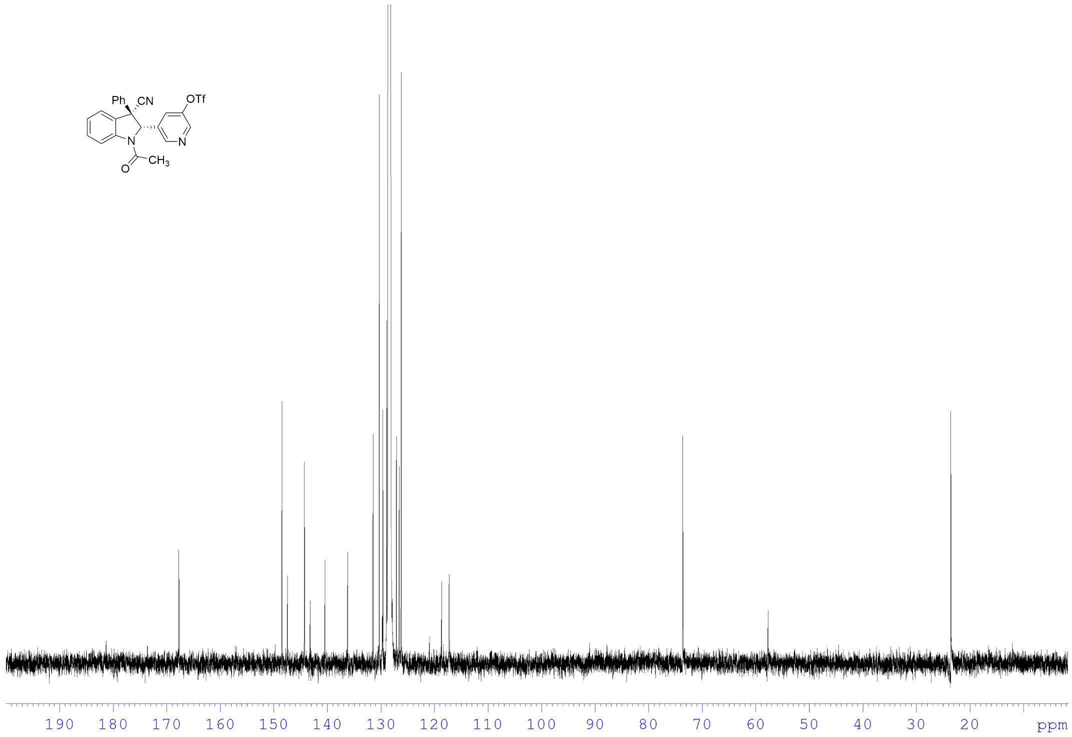
Ph
CN
OTf
N
O
CH3
190
180
170
160
150
140
130
120
110
100
90
80
70
60
50
40
30
20
ppm

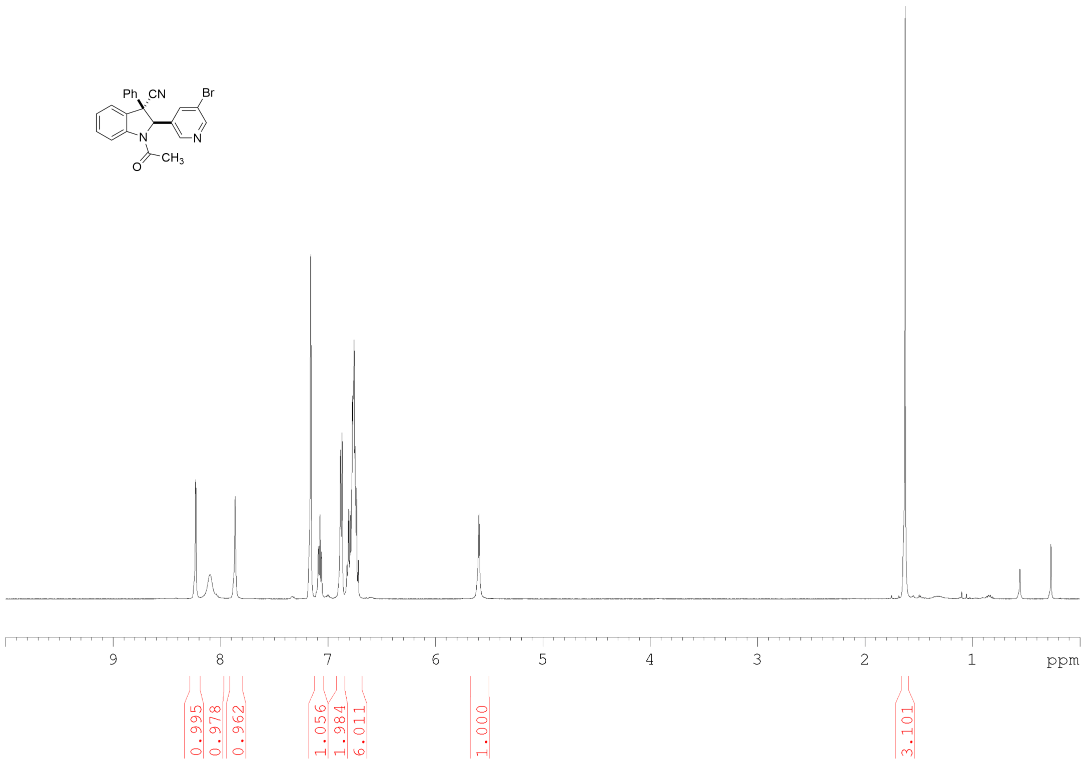
Ph
CN
Br
N
N
O
CH3
9
8
7
6
5
4
3
2
1
ppm
0.995
0.978
0.962
1.056
1.984
6.011
1.000
3.101

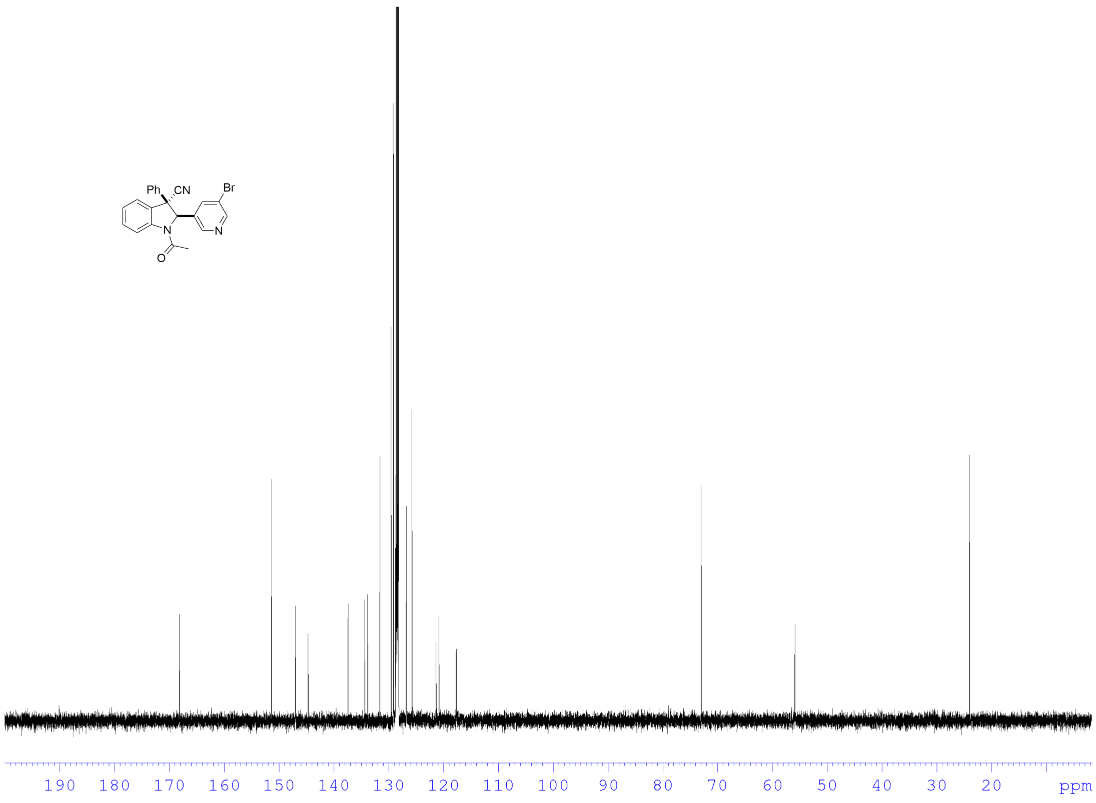
Ph
CN
Br
N
N
O
190
180
170
160
150
140
130
120
110
100
90
80
70
60
50
40
30
20
ppm

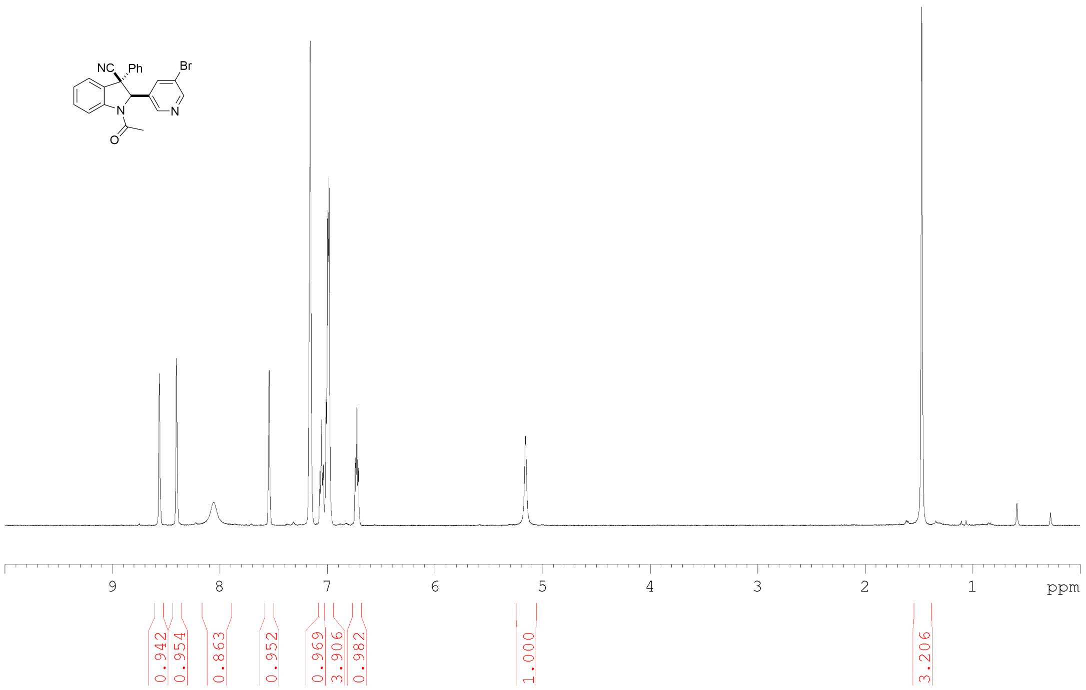
NC
Ph
Br
N
N
O
9
8
7
6
5
4
3
2
1
ppm
0.942
0.954
0.863
0.952
0.969
3.906
0.982
1.000
3.206

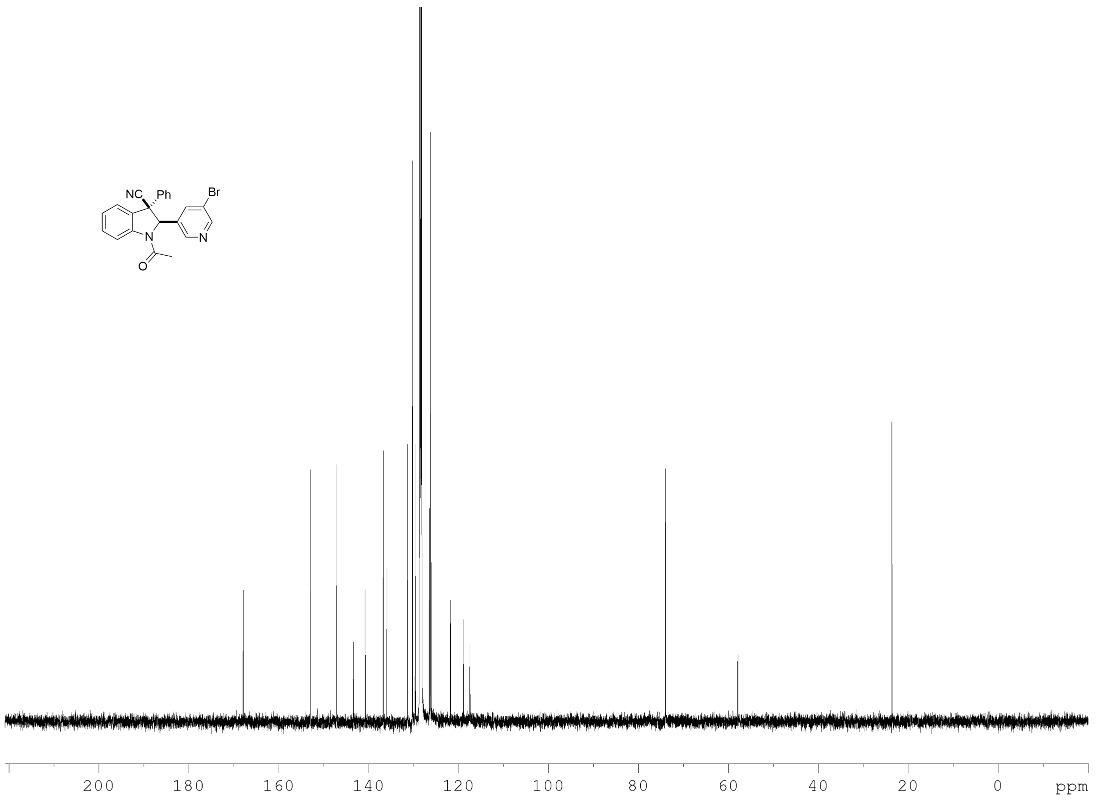
NC
Ph
Br
N
N
O
200
180
160
140
120
100
80
60
40
20
0
ppm

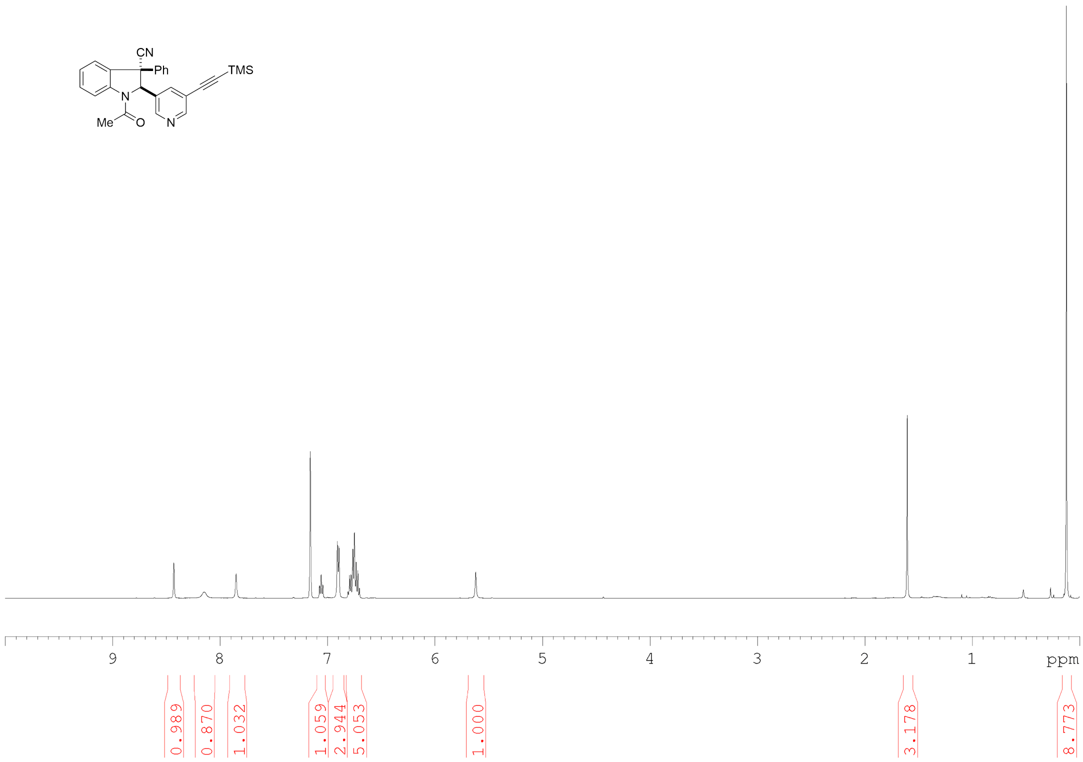
CN
Ph
TMS
N
Me
O
N
9
8
7
6
5
4
3
2
1
ppm
0.989
0.870
1.032
1.059
2.944
5.053
1.000
3.178
8.773

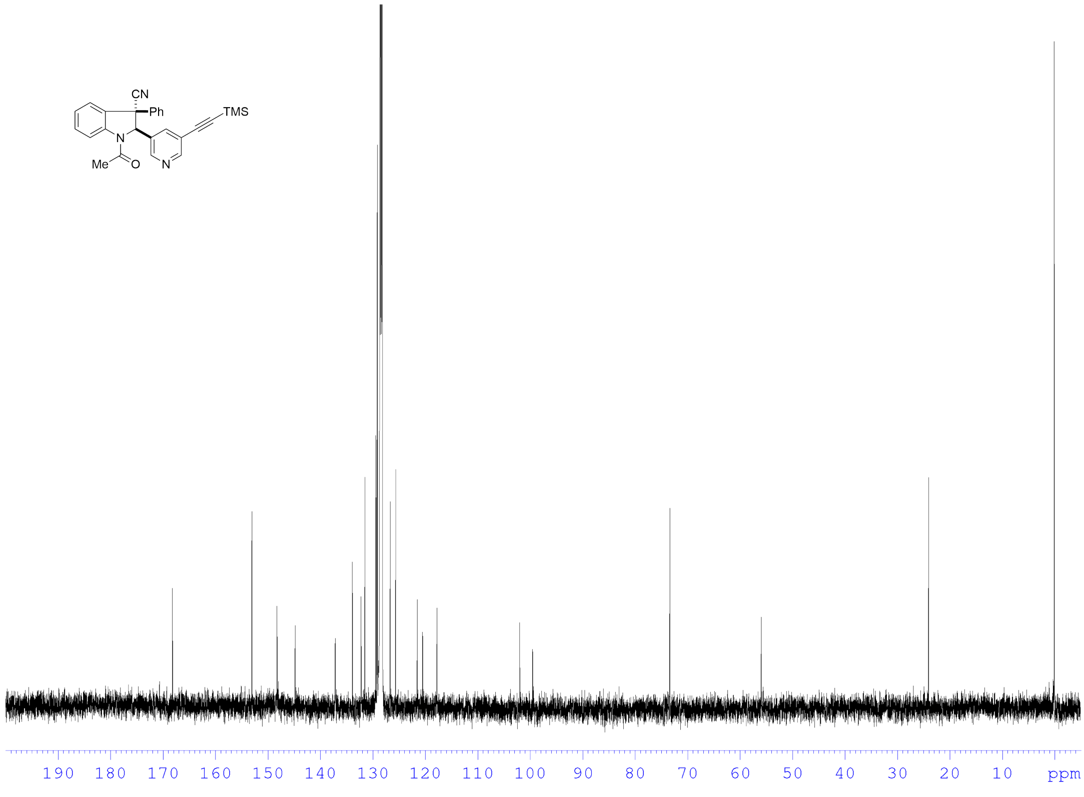
CN
Ph
TMS
N
Me
O
N
190
180
170
160
150
140
130
120
110
100
90
80
70
60
50
40
30
20
10
ppm

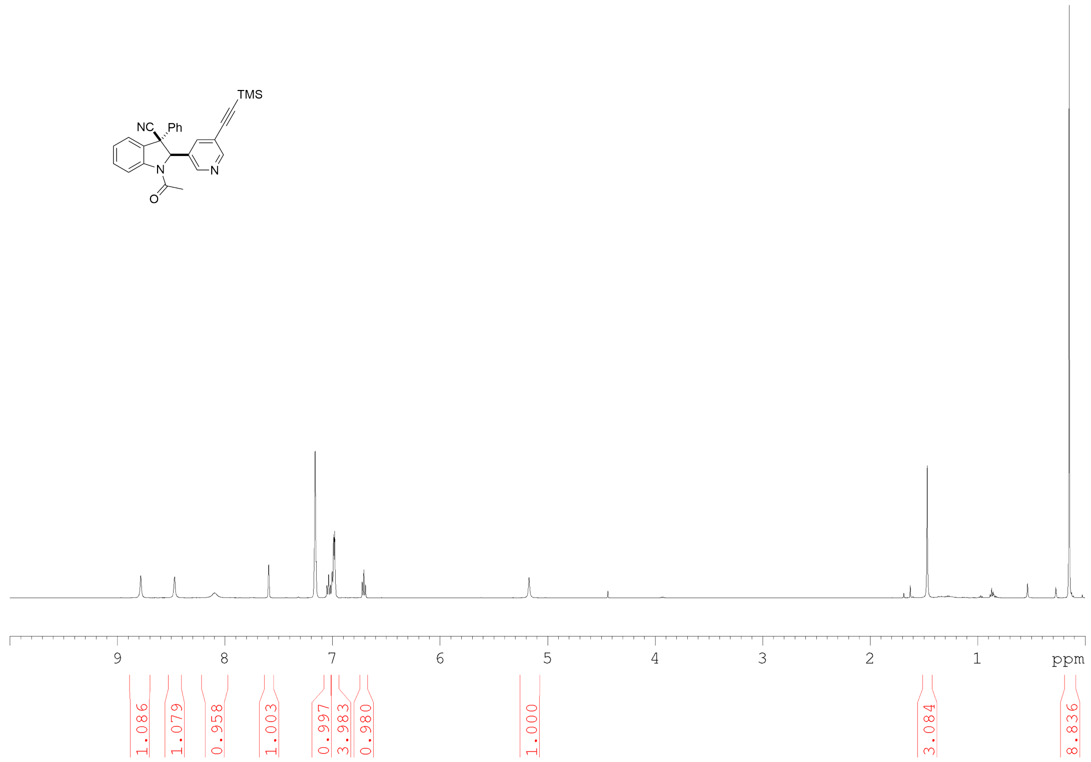
TMS
NC
Ph
N
N
O
9
8
7
6
5
4
3
2
1
ppm
1.086
1.079
0.958
1.003
0.997
3.983
0.980
1.000
3.084
8.836

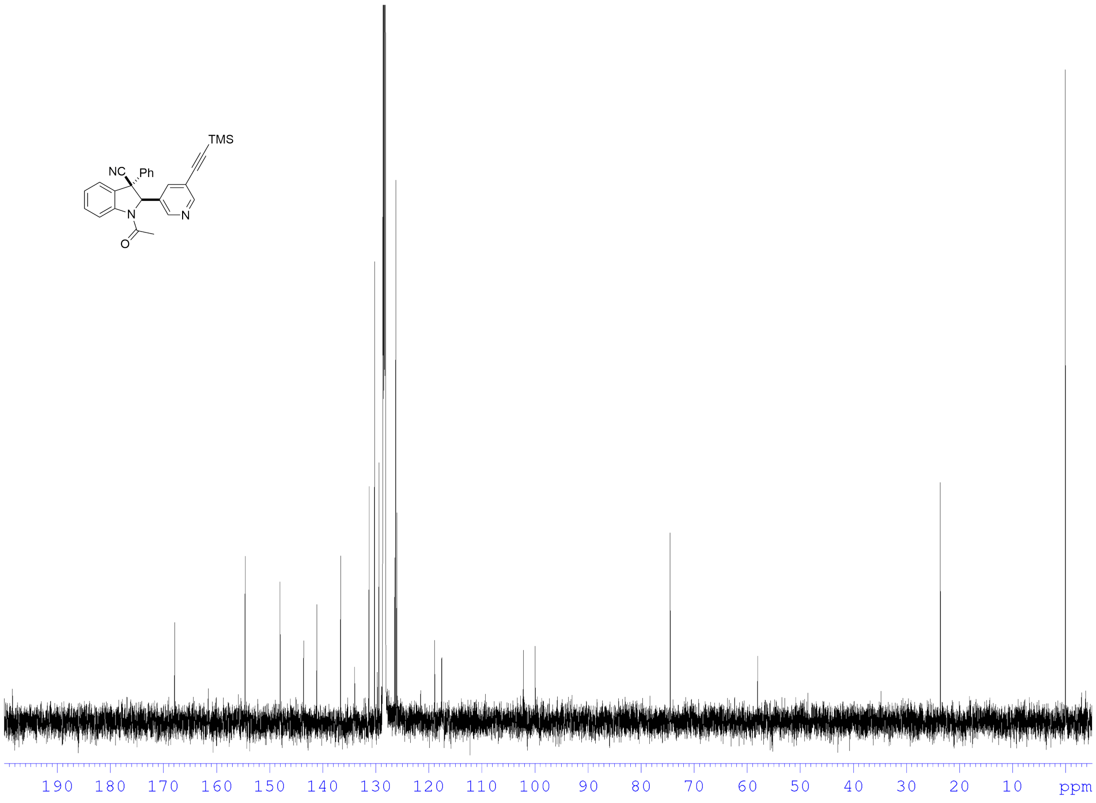
TMS
NC
Ph
N
N
O
190
180
170
160
150
140
130
120
110
100
90
80
70
60
50
40
30
20
10
ppm

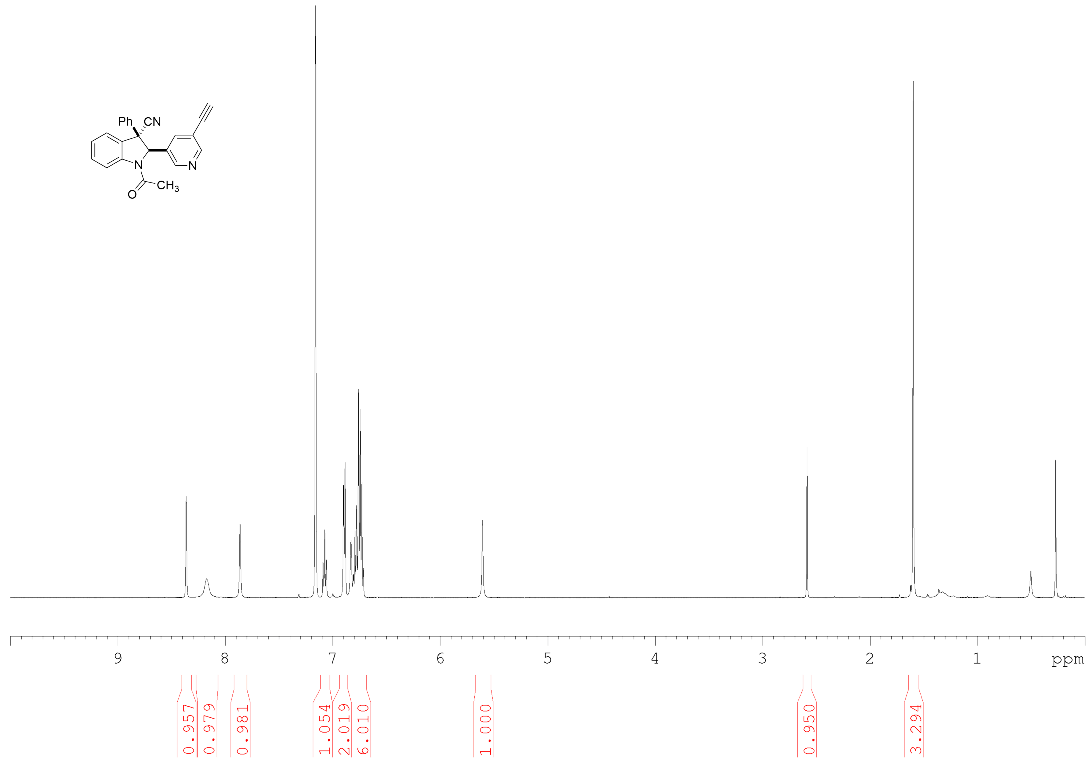
Ph
CN
N
N
O
CH3
9
8
7
6
5
4
3
2
1
ppm
0.957
0.979
0.981
1.054
2.019
6.010
1.000
0.950
3.294

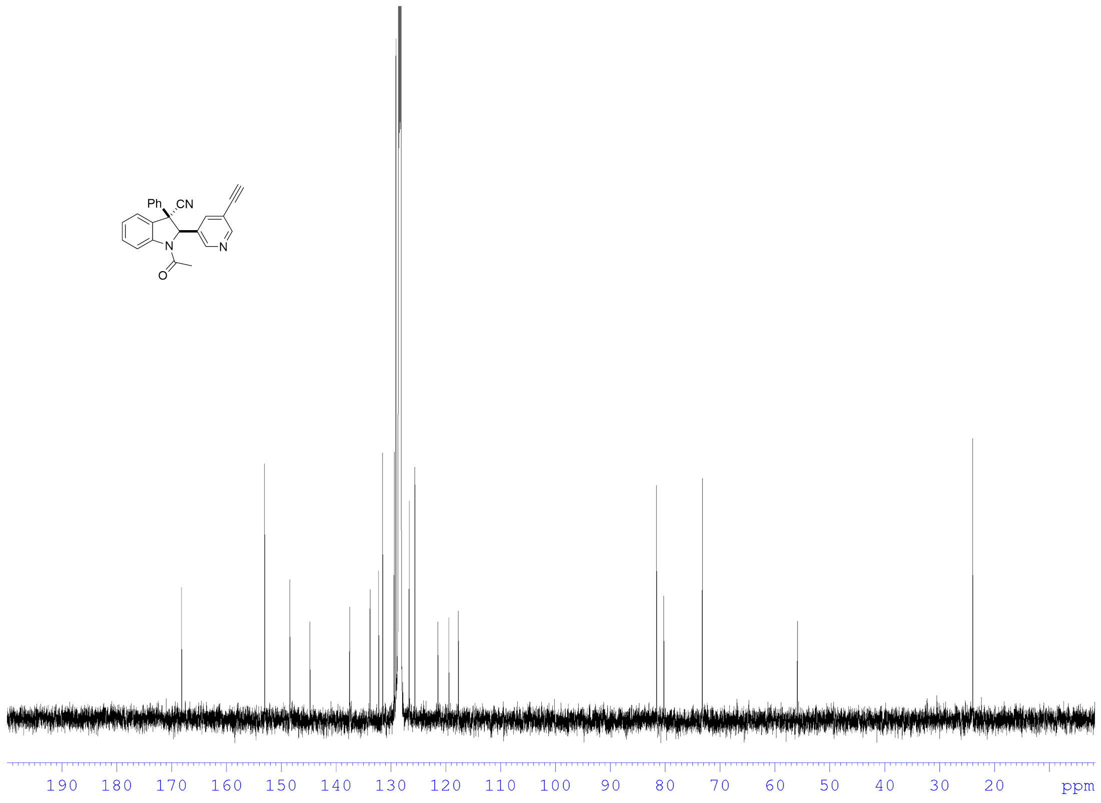
Ph
CN
N
N
O
190
180
170
160
150
140
130
120
110
100
90
80
70
60
50
40
30
20
ppm

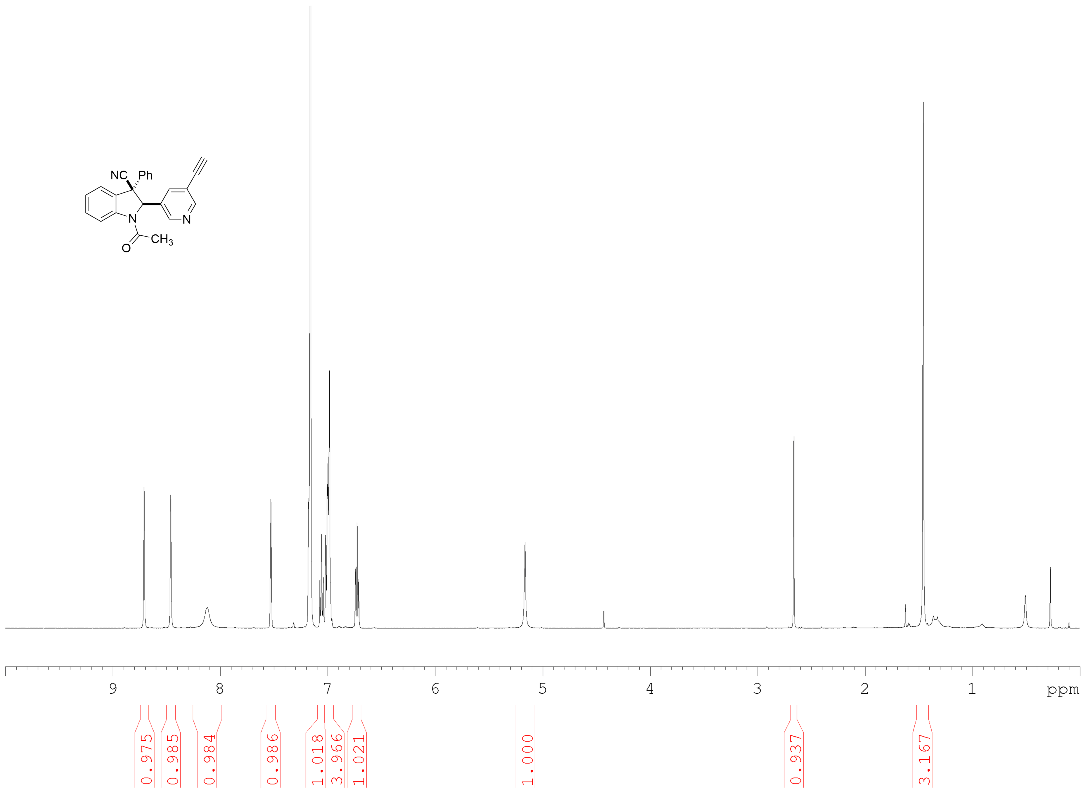
NC
Ph
N
N
O
CH3
9
8
7
6
5
4
3
2
1
ppm
0.975
0.985
0.984
0.986
1.018
3.966
1.021
1.000
0.937
3.167

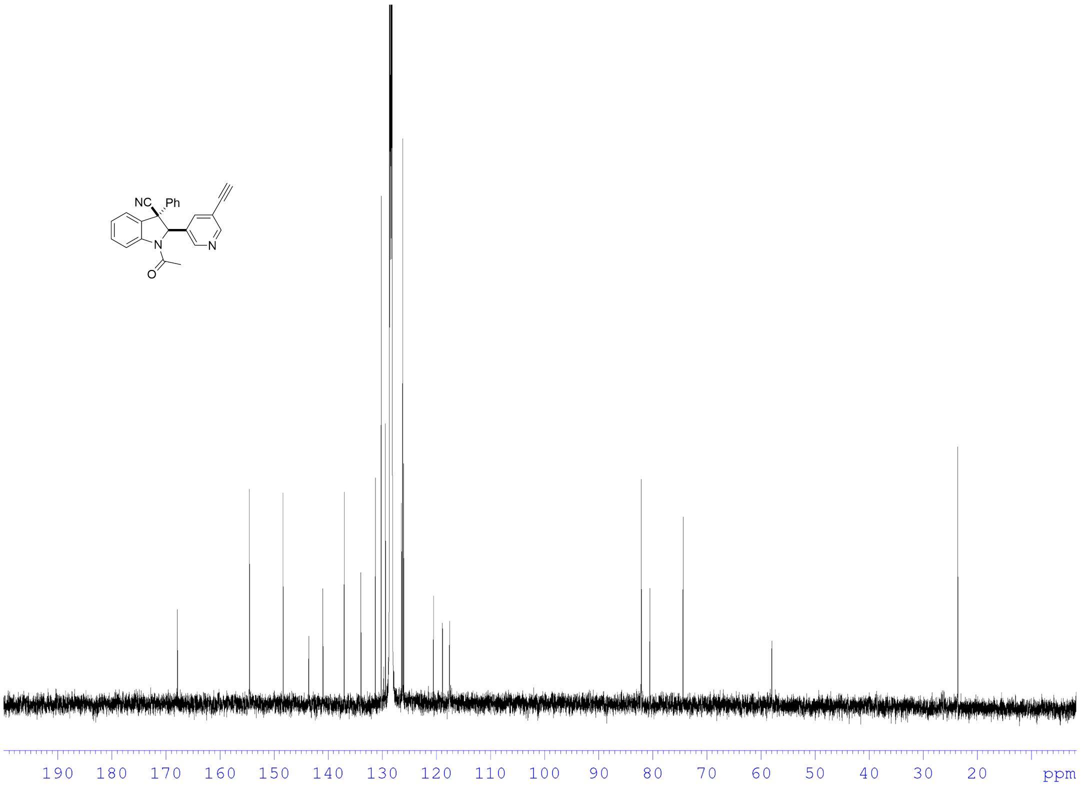
NC
Ph
N
N
O
190
180
170
160
150
140
130
120
110
100
90
80
70
60
50
40
30
20
ppm

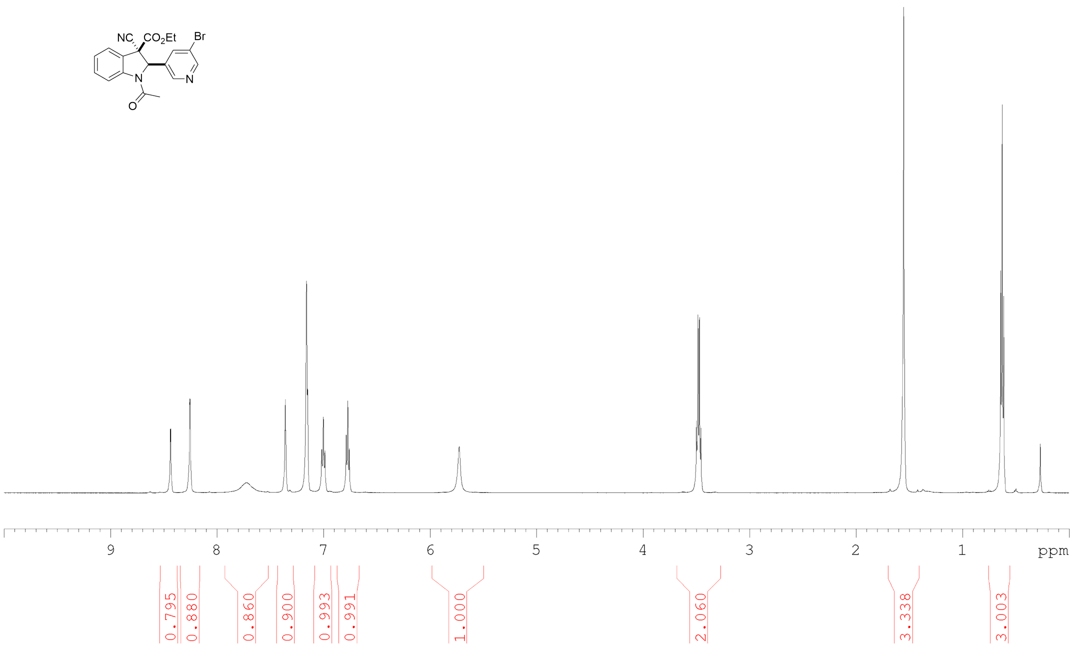
NC
CO2Et
Br
N
N
O
9
8
7
6
5
4
3
2
1
ppm
0.795
0.880
0.860
0.900
0.993
0.991
1.000
2.060
3.338
3.003

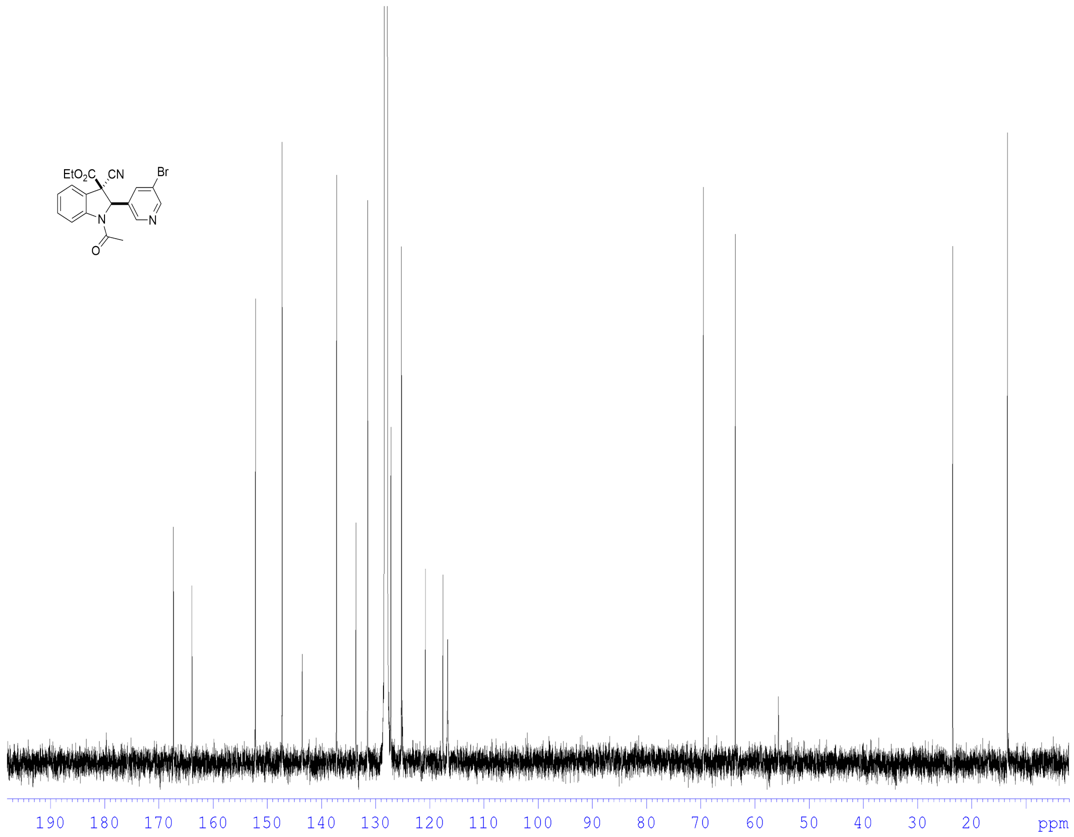
EtO2C
CN
Br
N
N
O
190
180
170
160
150
140
130
120
110
100
90
80
70
60
50
40
30
20
ppm

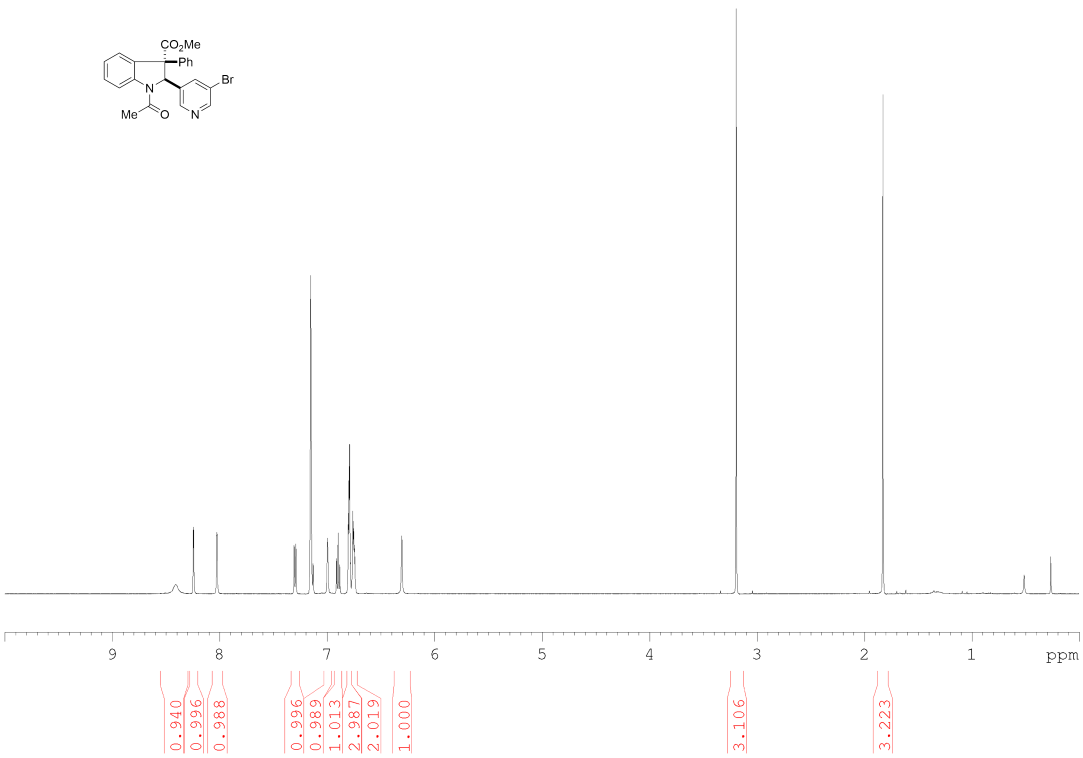
CO2Me
Ph
Br
N
Me
O
N
9
8
7
6
5
4
3
2
1
ppm
0.940
0.996
0.988
0.996
0.989
1.013
2.987
2.019
1.000
3.106
3.223

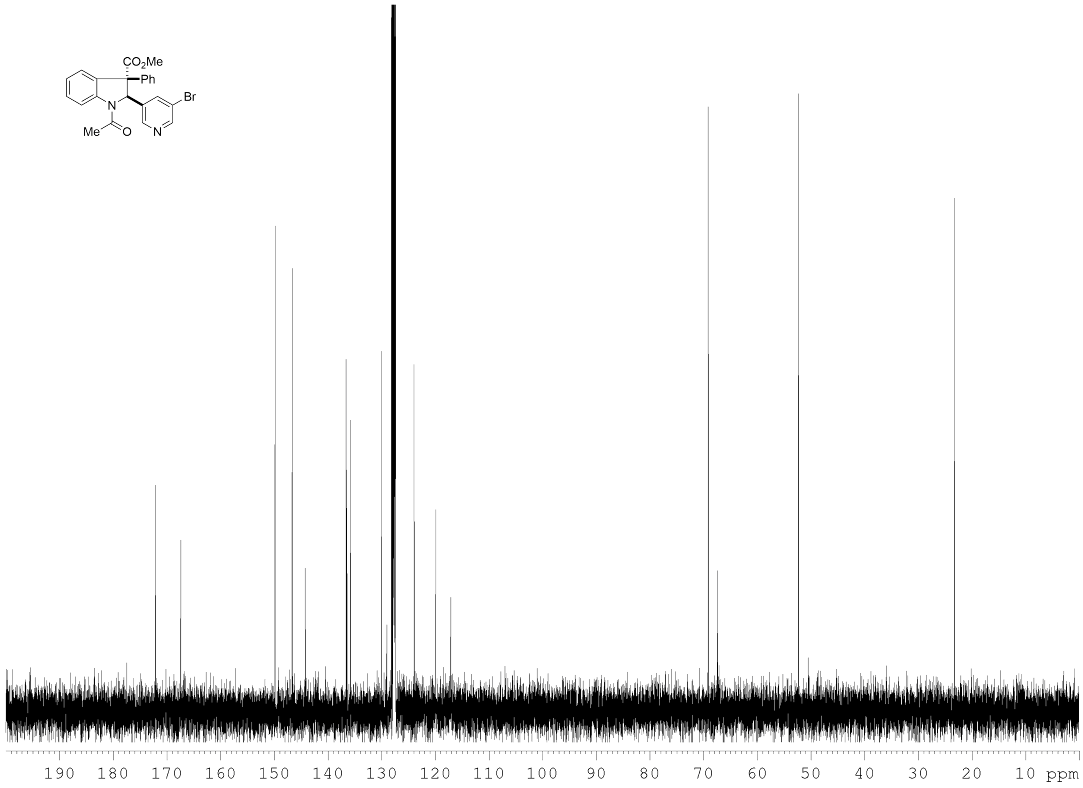
CO2Me
Ph
Br
N
Me
O
N
190
180
170
160
150
140
130
120
110
100
90
80
70
60
50
40
30
20
10
ppm

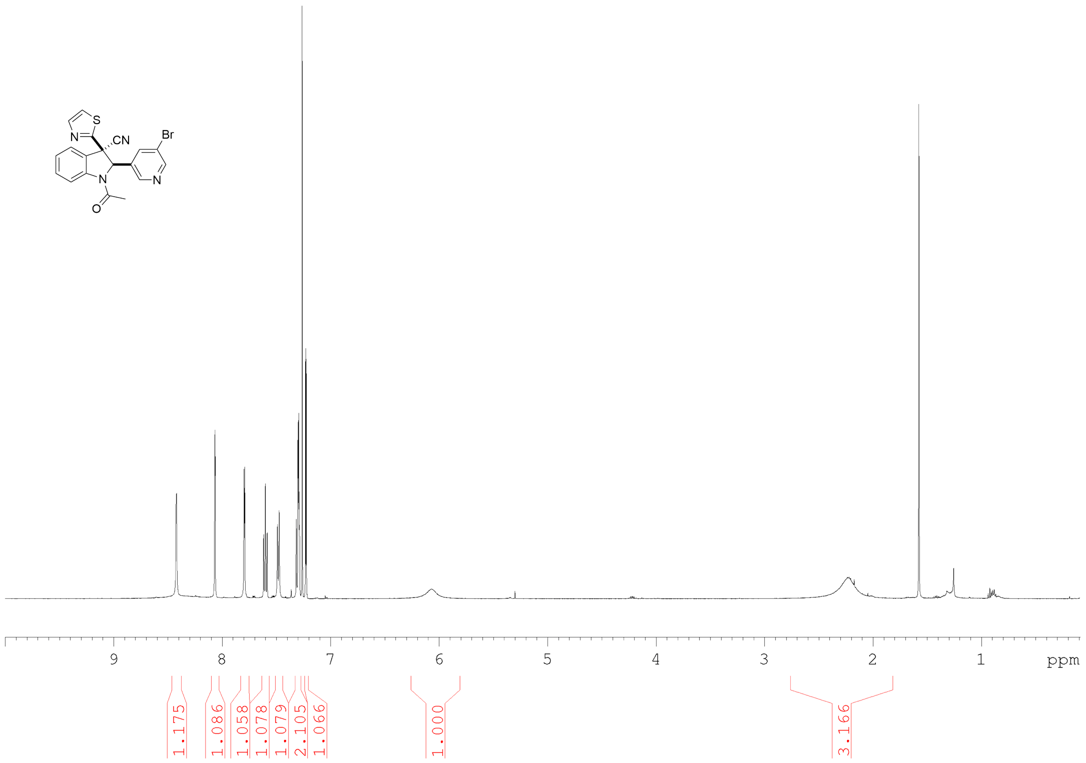

Br
CN
N
S
N
O
9 8 7 6 5 4 3 2 1 ppm
1.175
1.086
1.058
1.078
1.079
2.105
1.066
1.000
3.166

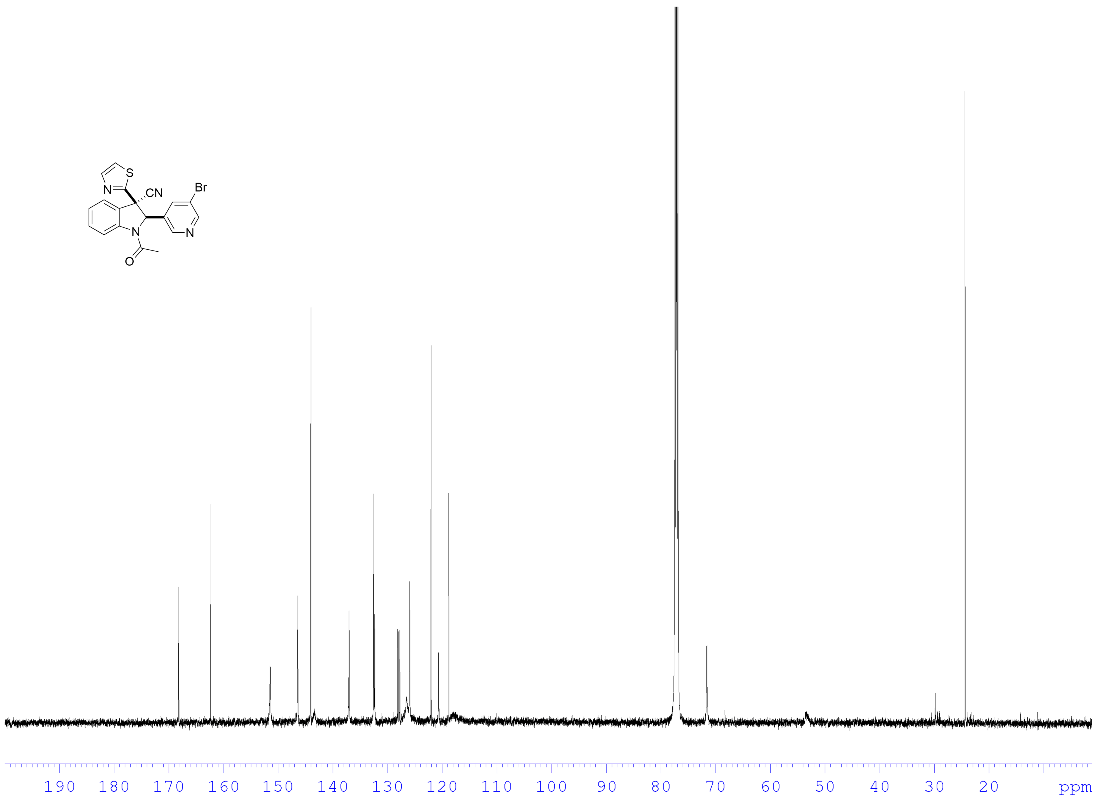

Br
CN
N
S
N
O
190 180 170 160 150 140 130 120 110 100 90 80 70 60 50 40 30 20 ppm

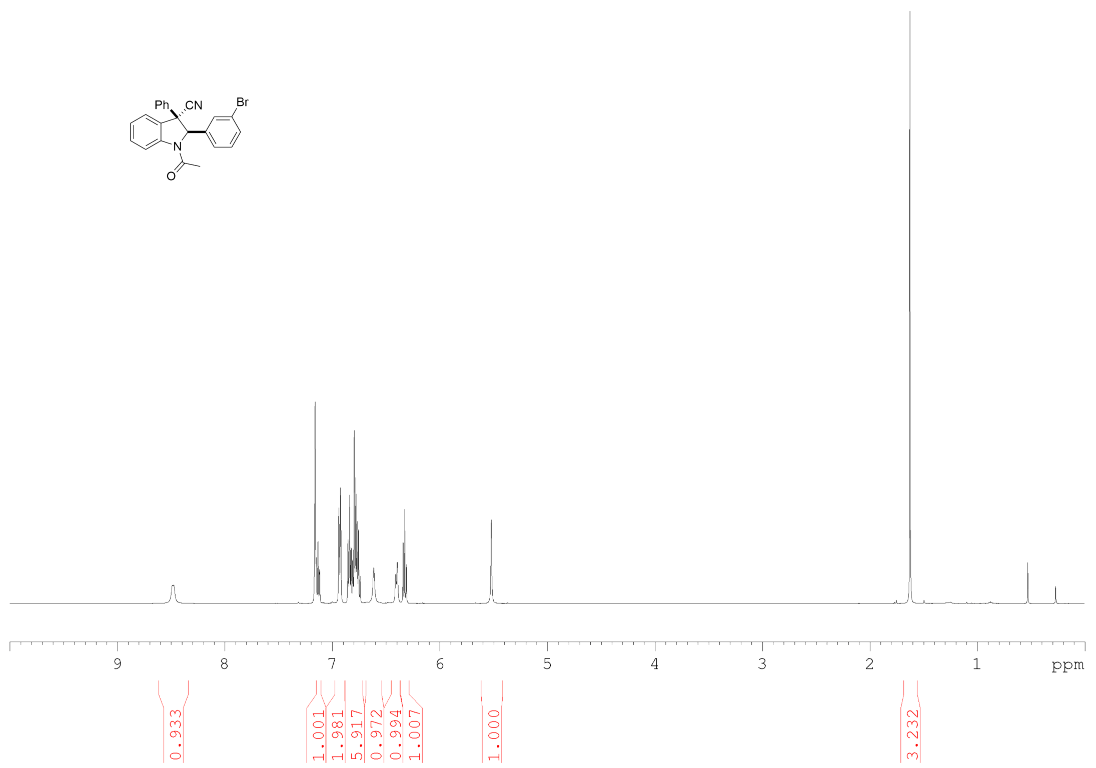
Ph
CN
Br
N
O
9
8
7
6
5
4
3
2
1
ppm
0.933
1.001
1.981
5.917
0.972
0.994
1.007
1.000
3.232

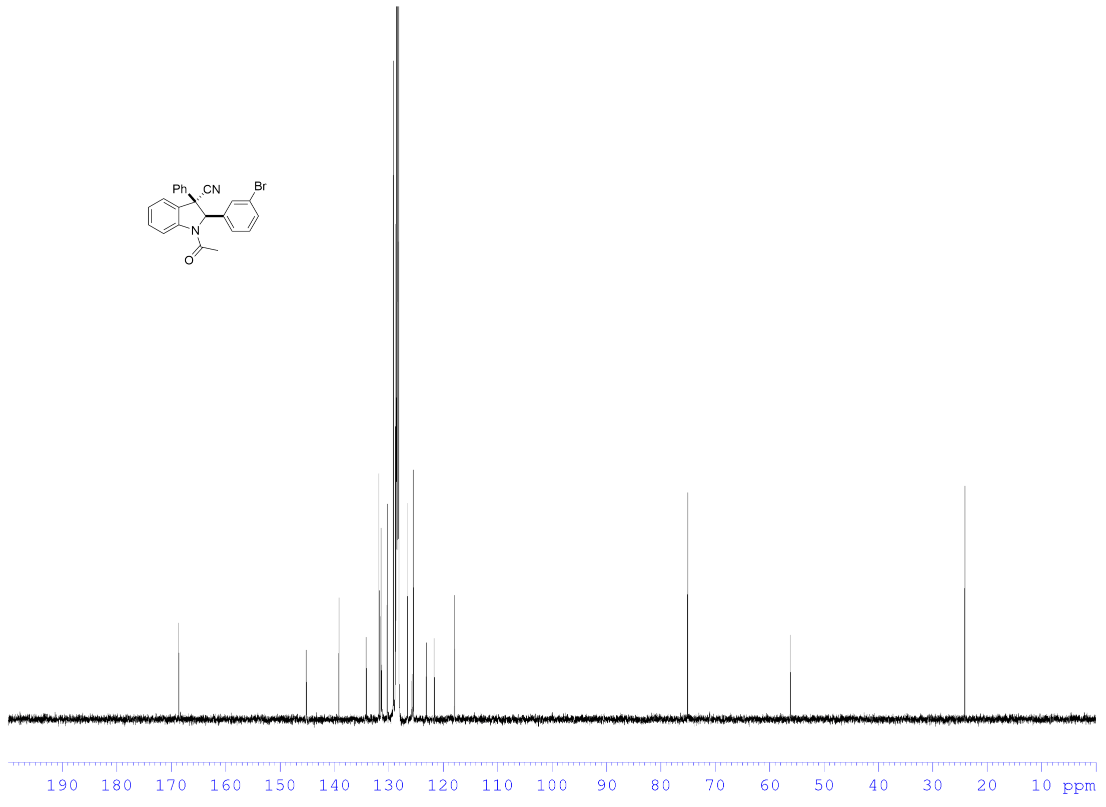
Ph
CN
Br
N
O
190
180
170
160
150
140
130
120
110
100
90
80
70
60
50
40
30
20
10
ppm

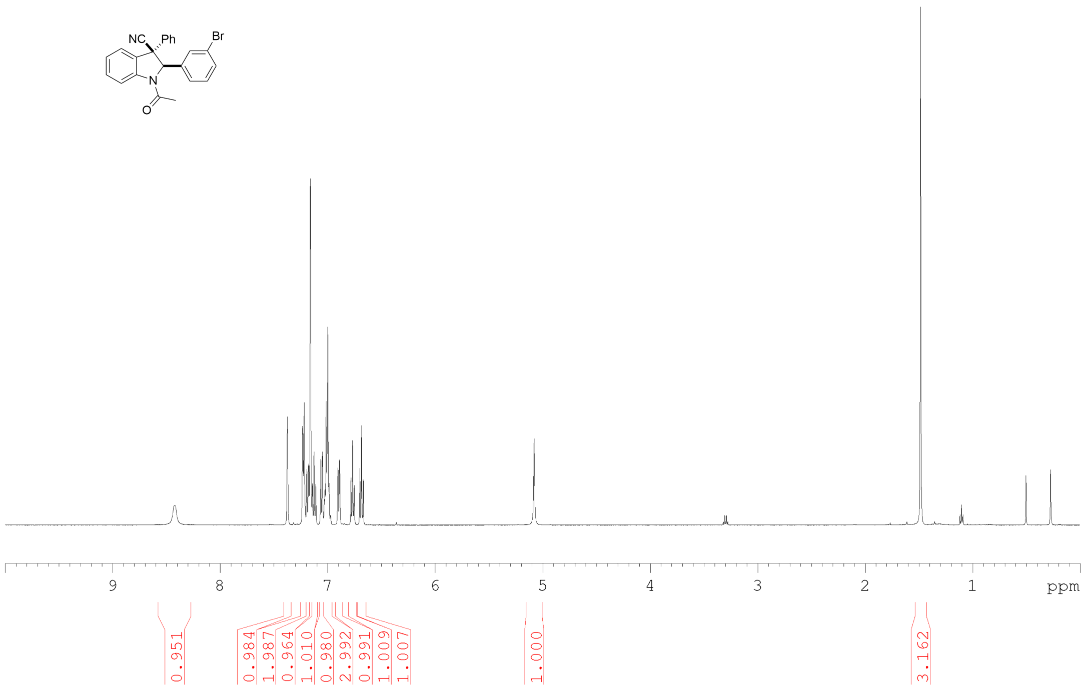
NC
Ph
Br
N
O
ppm
0.951
0.984
1.987
0.964
1.010
0.980
2.992
0.991
1.009
1.007
1.000
3.162

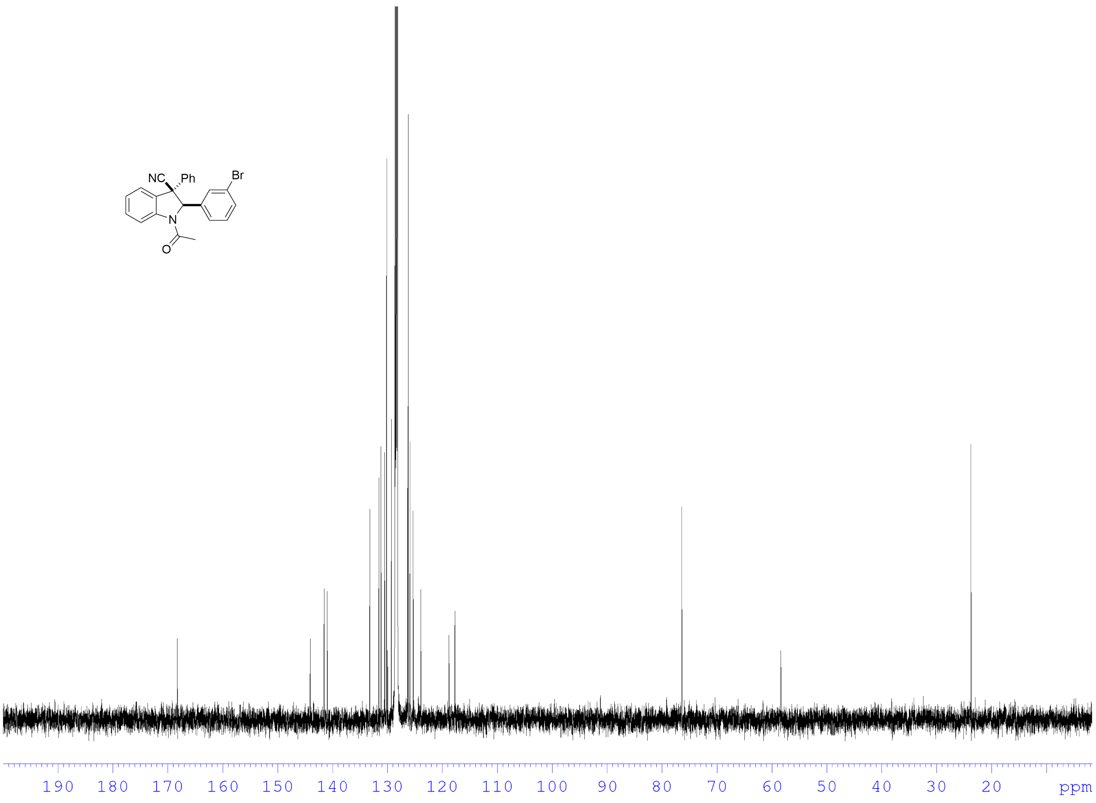
NC
Ph
Br
N
O
ppm

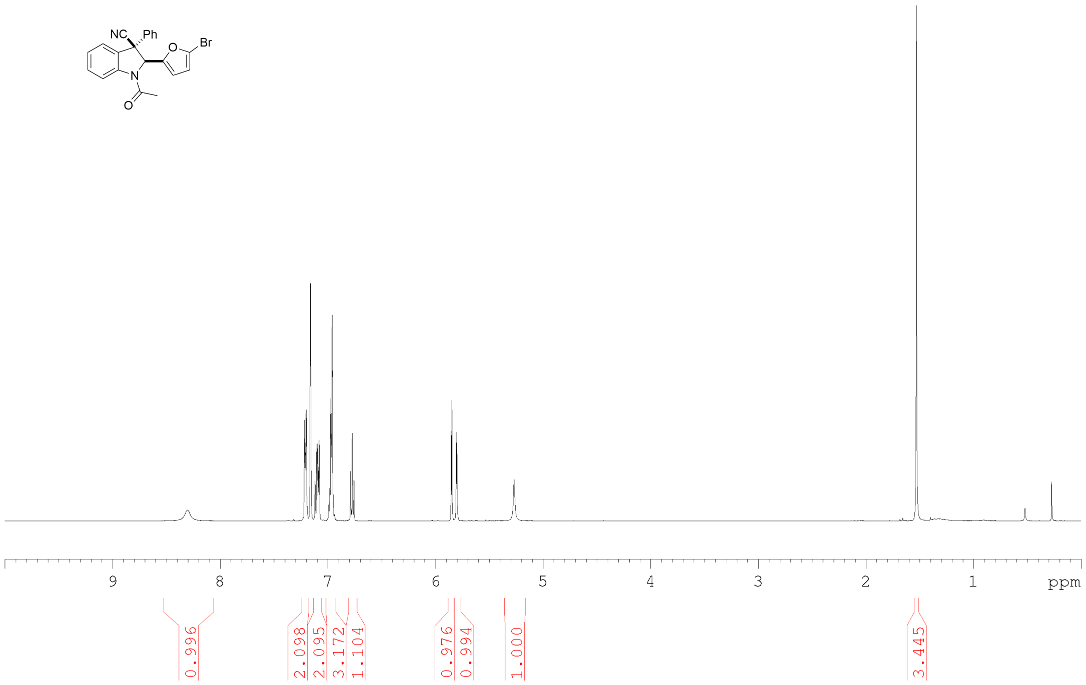
NC
Ph
O
Br
N
O
9
8
7
6
5
4
3
2
1
ppm
0.996
2.098
2.095
3.172
1.104
0.976
0.994
1.000
3.445

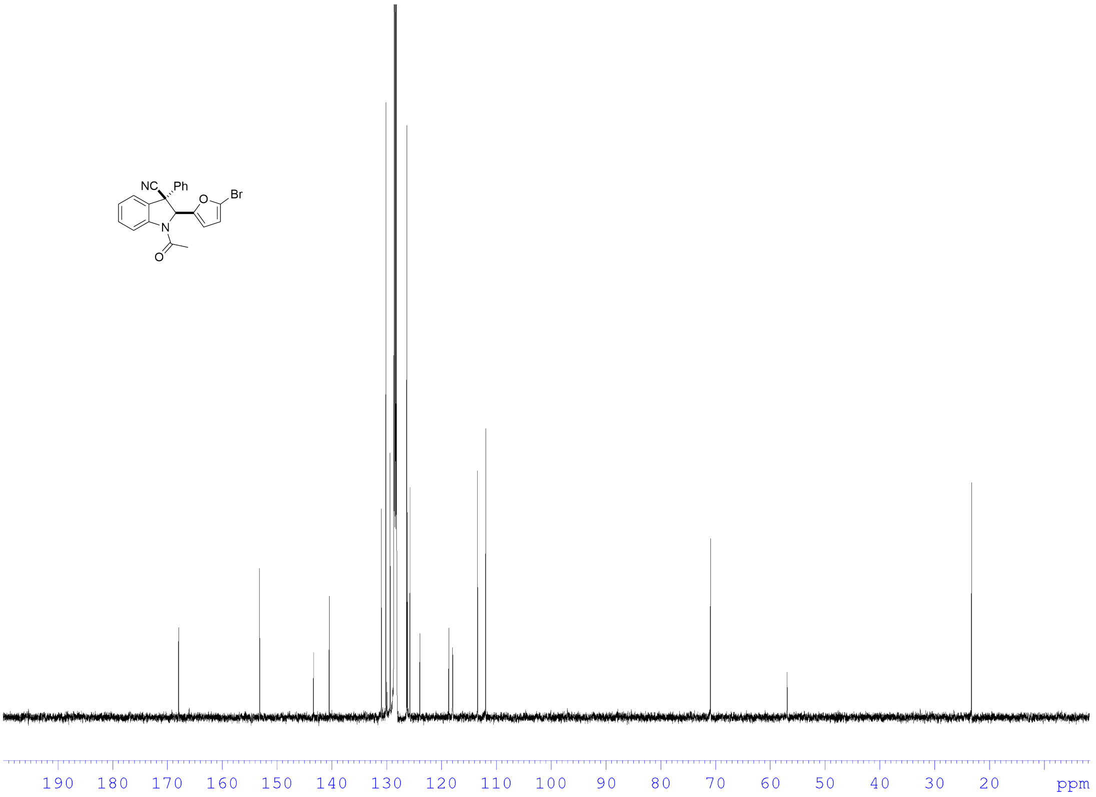
NC
Ph
O
Br
N
O
190
180
170
160
150
140
130
120
110
100
90
80
70
60
50
40
30
20
ppm

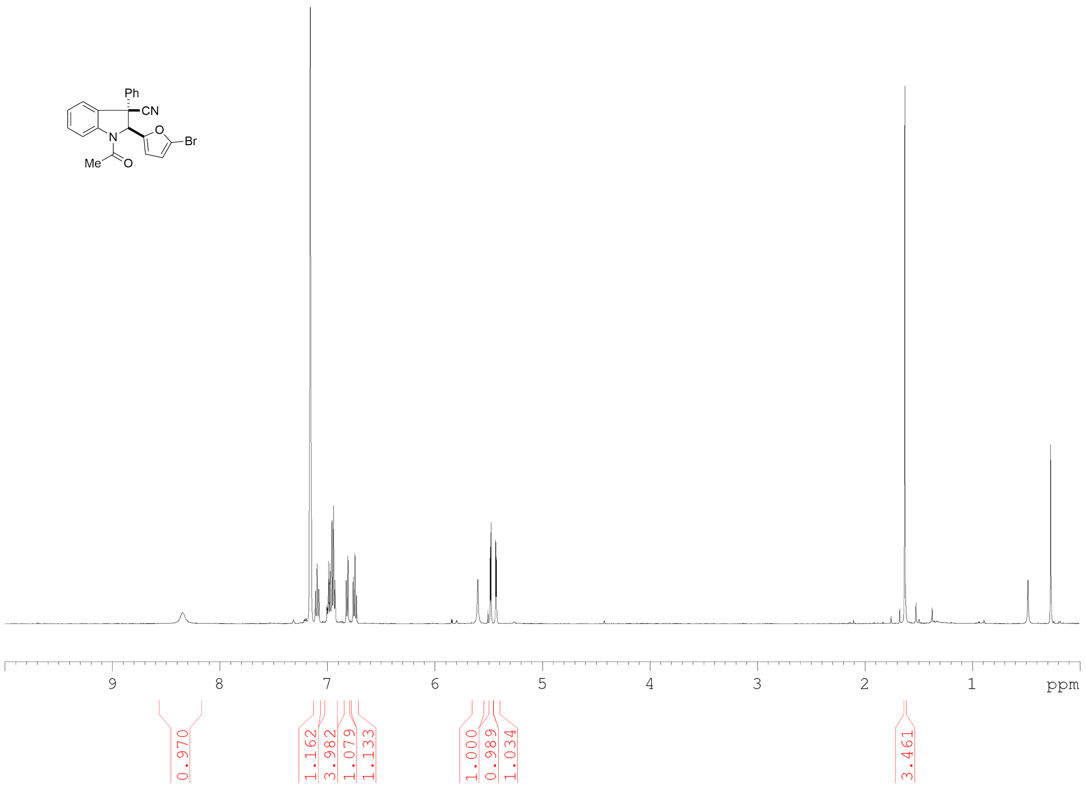
Ph
CN
N
O
Br
Me
O
9
8
7
6
5
4
3
2
1
ppm
0.970
1.162
3.982
1.079
1.133
1.000
0.989
1.034
3.461

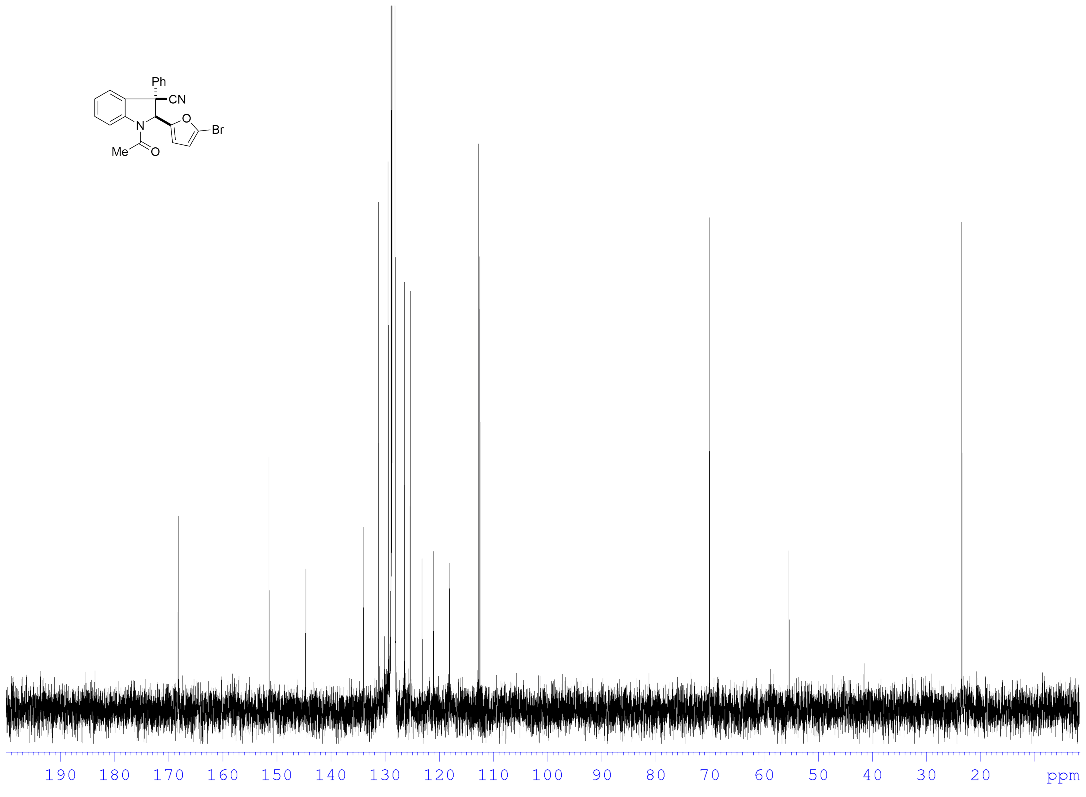
Ph
CN
N
O
Br
Me
O
190
180
170
160
150
140
130
120
110
100
90
80
70
60
50
40
30
20
ppm

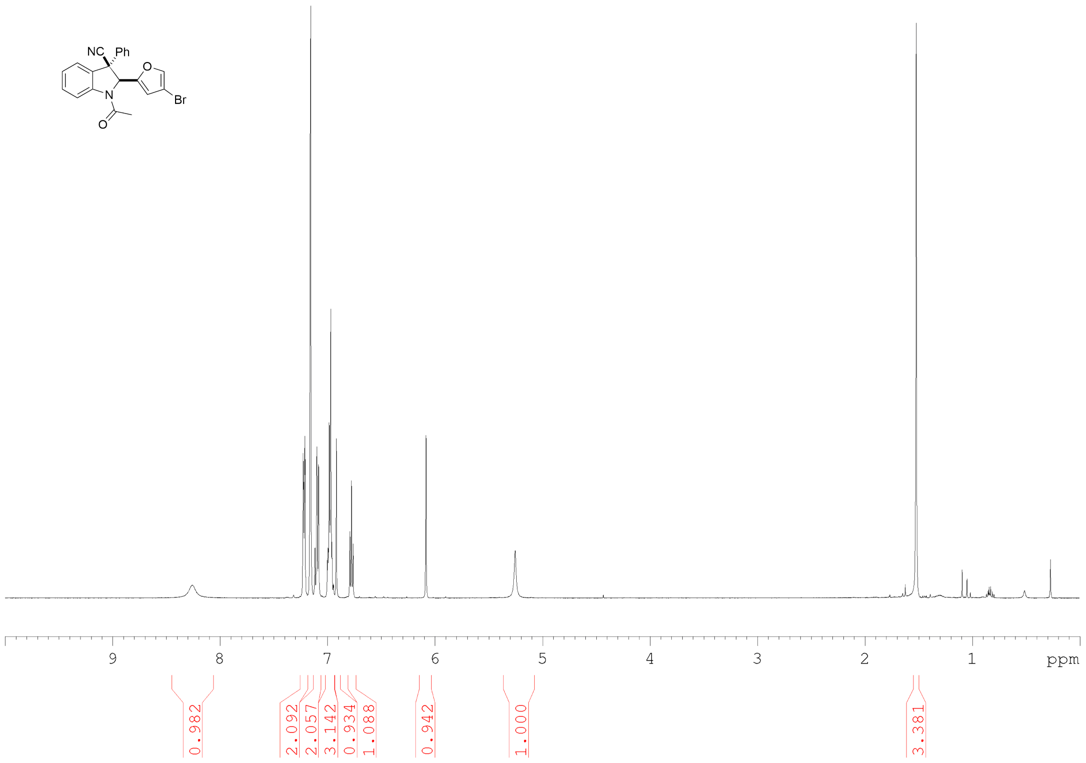
NC
Ph
O
Br
N
O
9 8 7 6 5 4 3 2 1 ppm
0.982
2.092
2.057
3.142
0.934
1.088
0.942
1.000
3.381

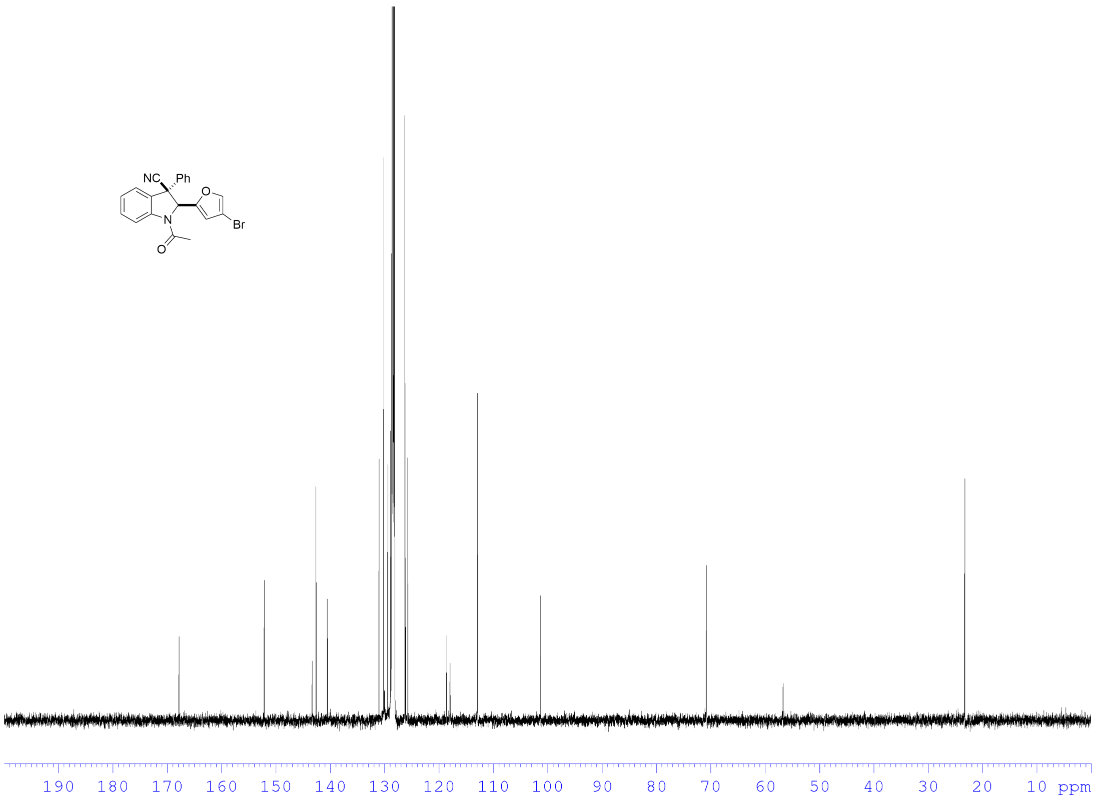
NC
Ph
O
Br
N
O
190 180 170 160 150 140 130 120 110 100 90 80 70 60 50 40 30 20 10 ppm

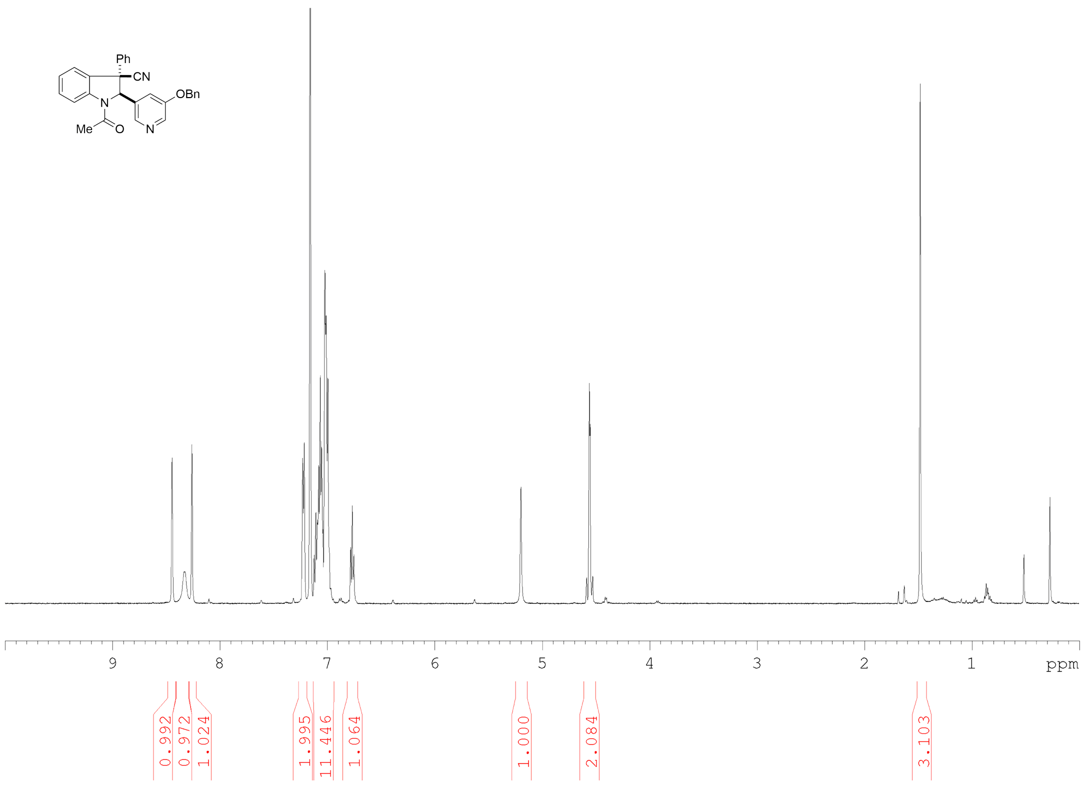

Ph
CN
OBn
N
Me
O
N
9 8 7 6 5 4 3 2 1 ppm
0.992
0.972
1.024
1.995
11.446
1.064
1.000
2.084
3.103

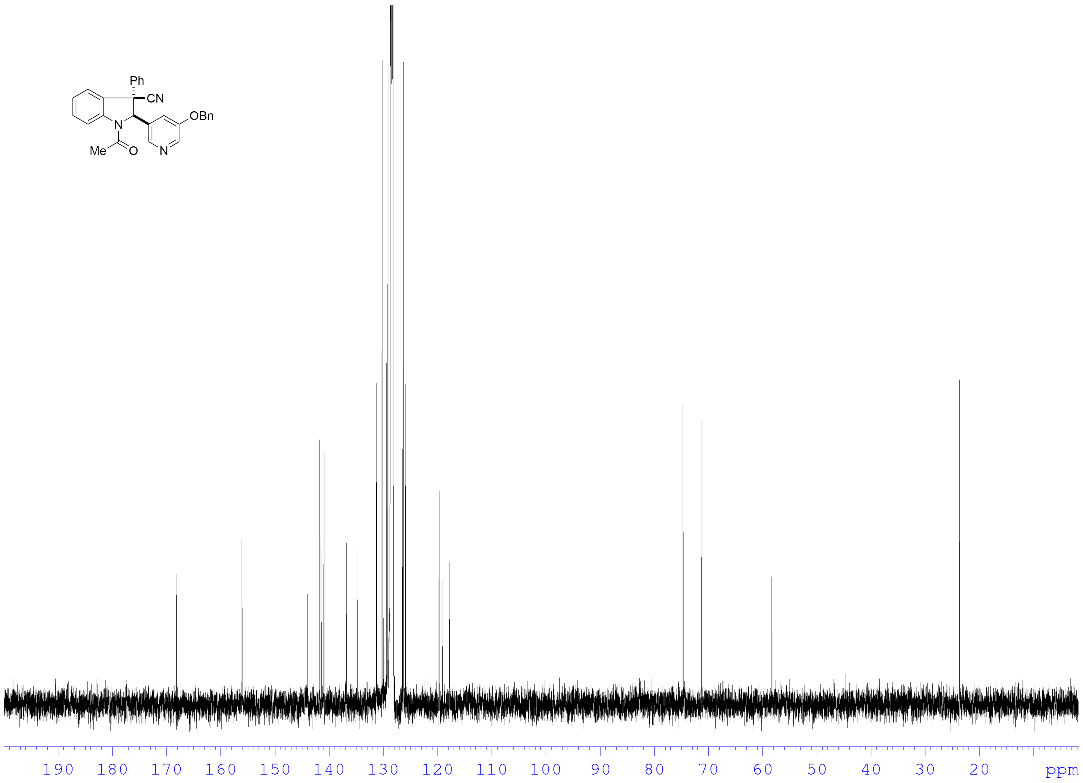

Ph
CN
OBn
N
Me
O
N
190 180 170 160 150 140 130 120 110 100 90 80 70 60 50 40 30 20 ppm

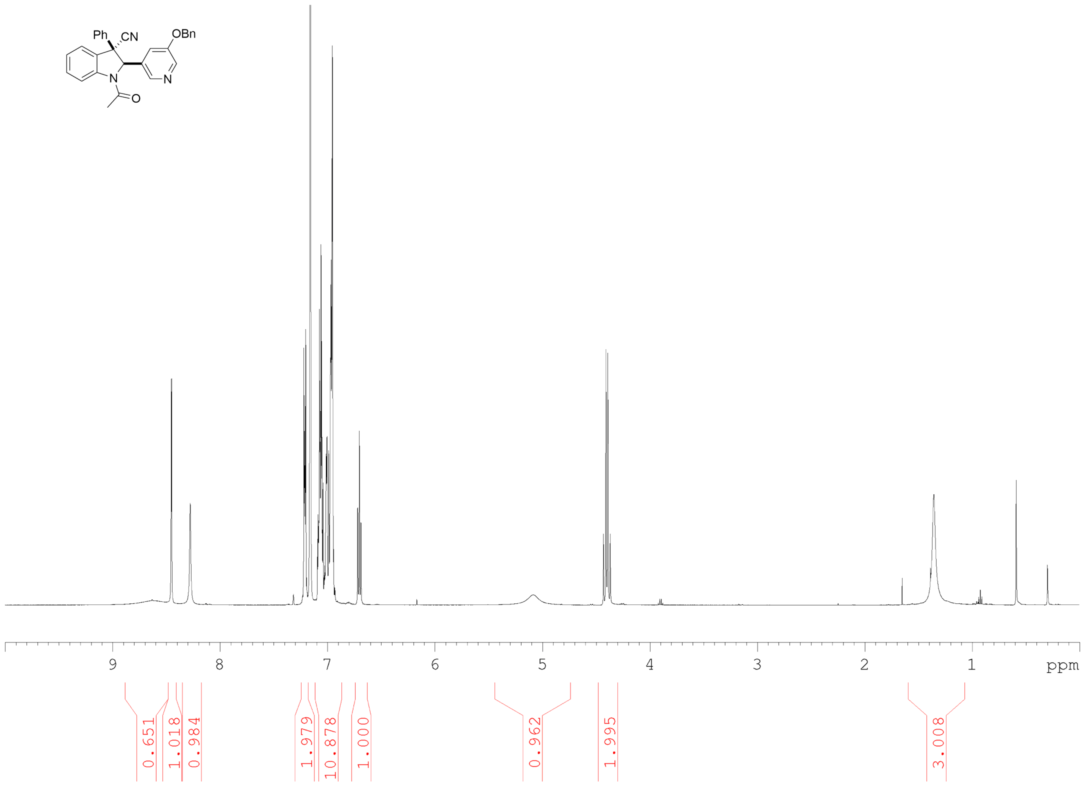
Ph
CN
OBn
N
N
O
9
8
7
6
5
4
3
2
1
ppm
0.651
1.018
0.984
1.979
10.878
1.000
0.962
1.995
3.008

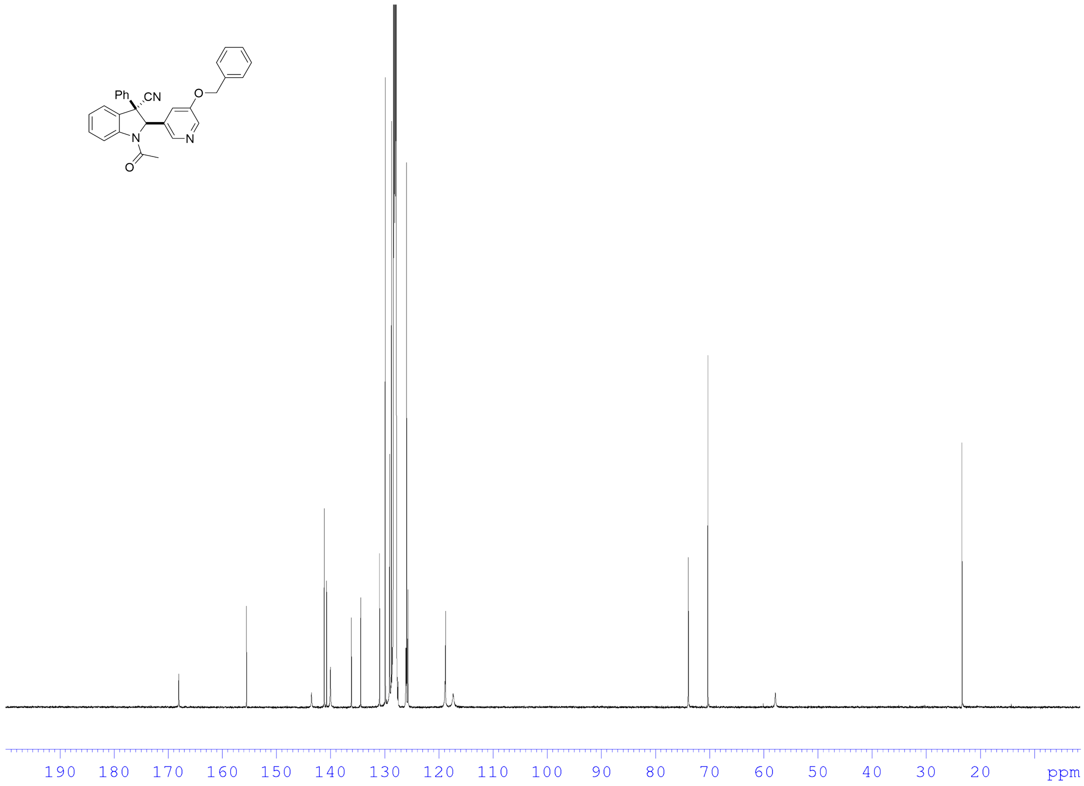
Ph
CN
O
N
N
O
190
180
170
160
150
140
130
120
110
100
90
80
70
60
50
40
30
20
ppm

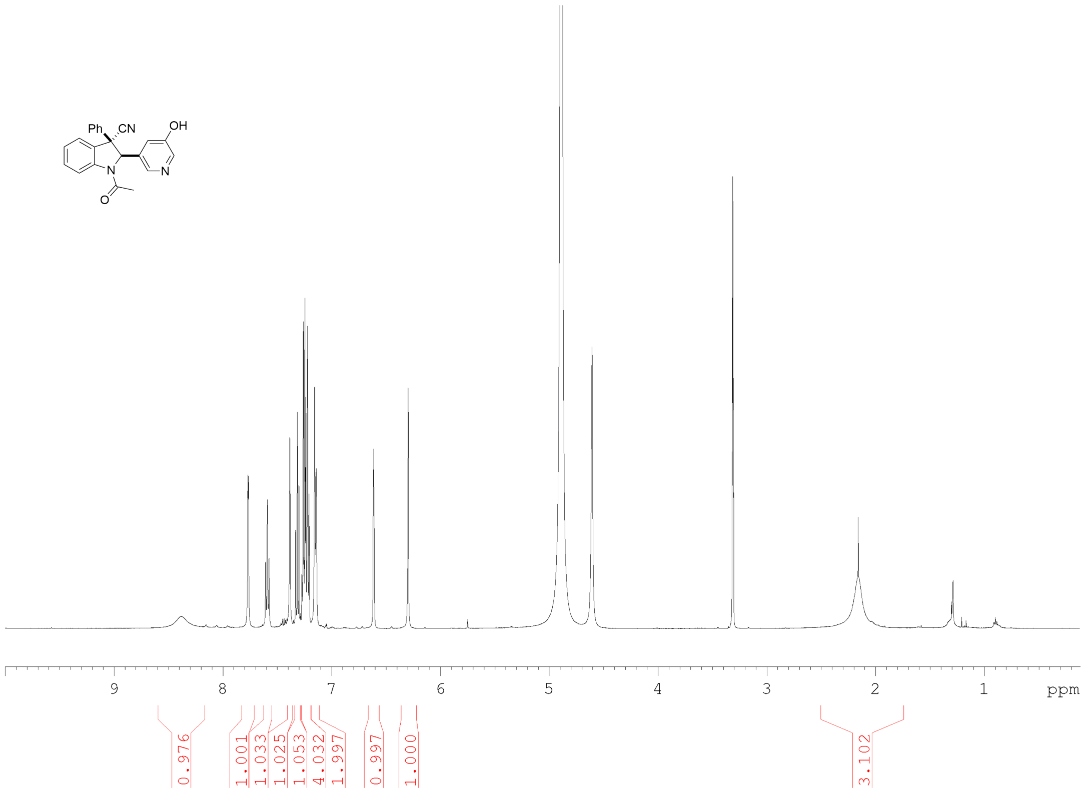
Ph
CN
OH
N
N
O
9
8
7
6
5
4
3
2
1
ppm
0.976
1.001
1.033
1.025
1.053
4.032
1.997
0.997
1.000
3.102

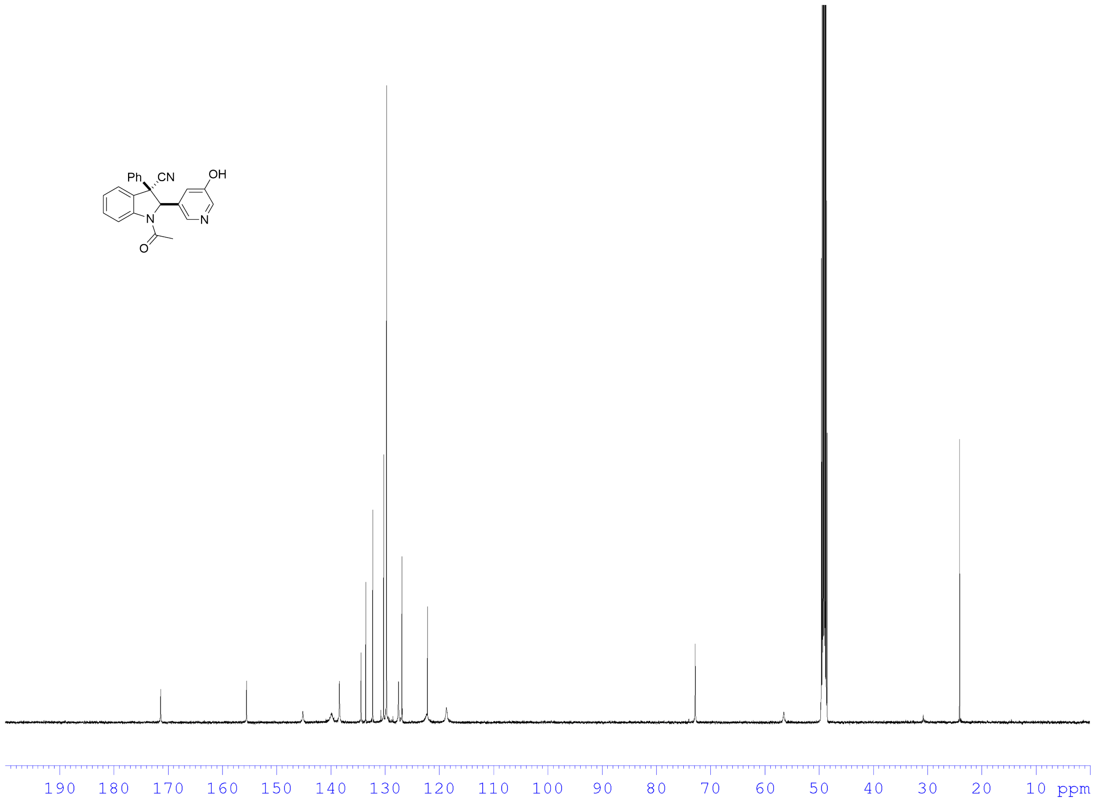
Ph
CN
OH
N
N
O
190
180
170
160
150
140
130
120
110
100
90
80
70
60
50
40
30
20
10
ppm

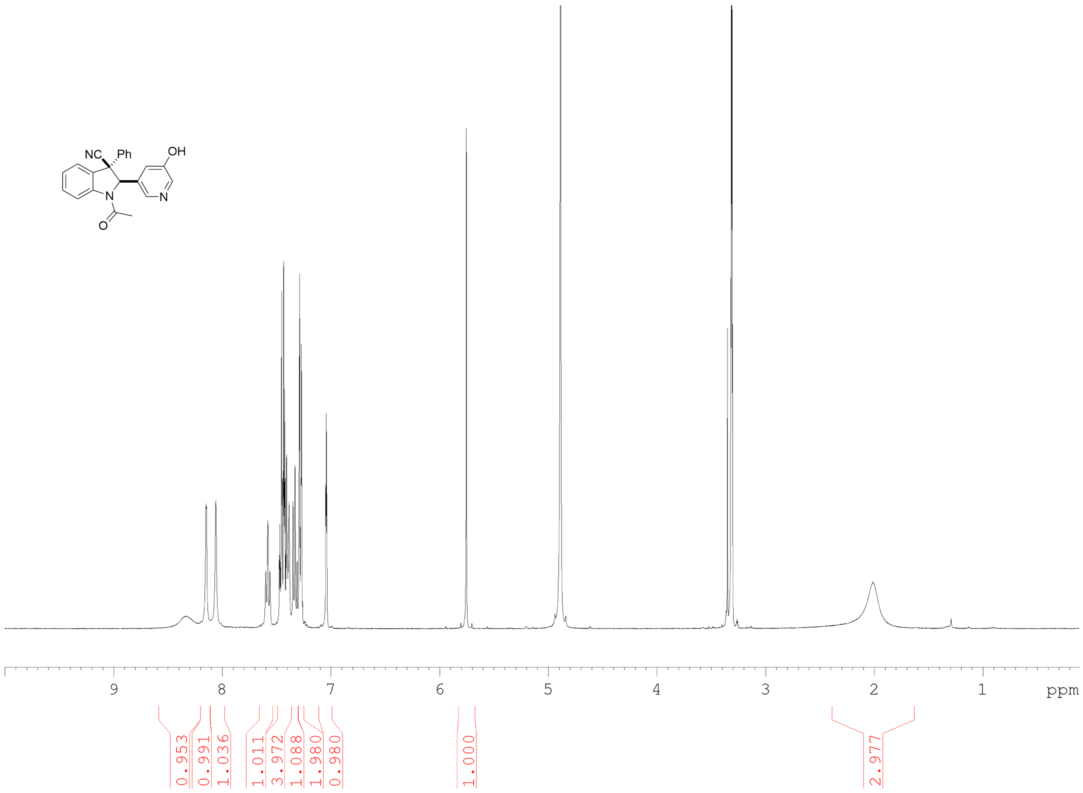
NC
Ph
OH
N
N
O
9
8
7
6
5
4
3
2
1
ppm
0.953
0.991
1.036
1.011
3.972
1.088
1.980
0.980
1.000
2.977

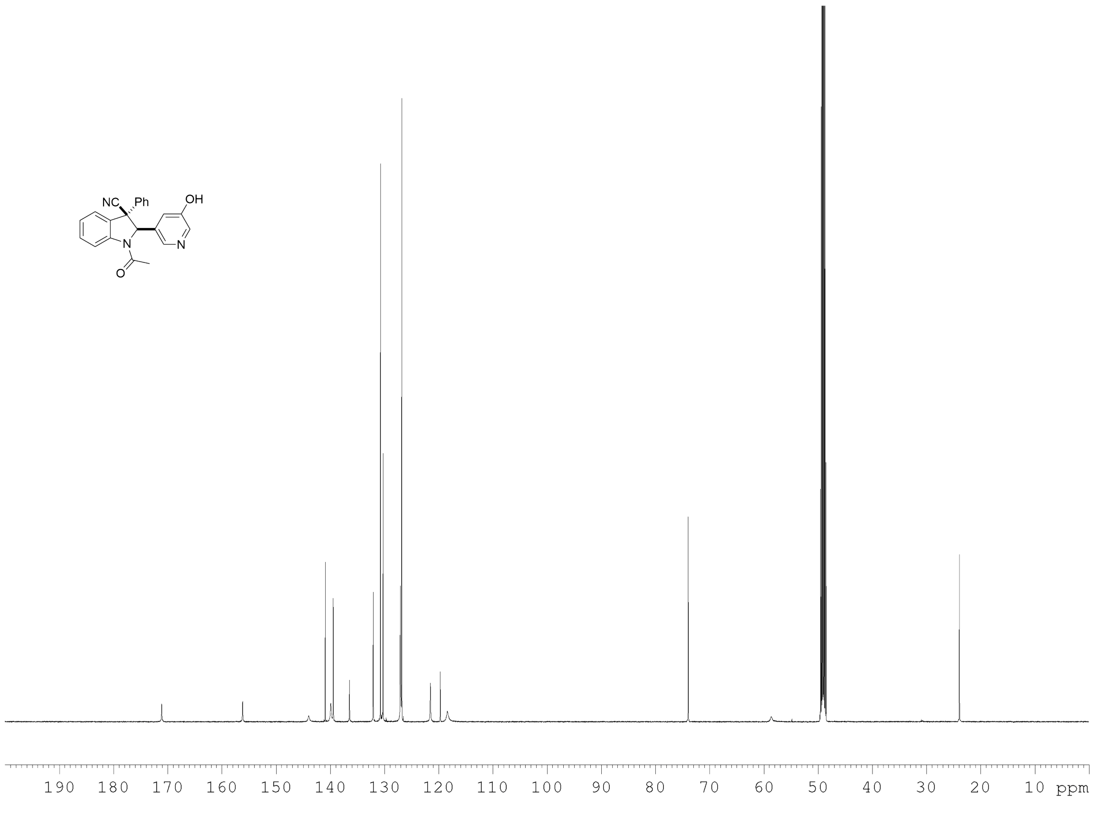
NC
Ph
OH
N
N
O
190
180
170
160
150
140
130
120
110
100
90
80
70
60
50
40
30
20
10
ppm

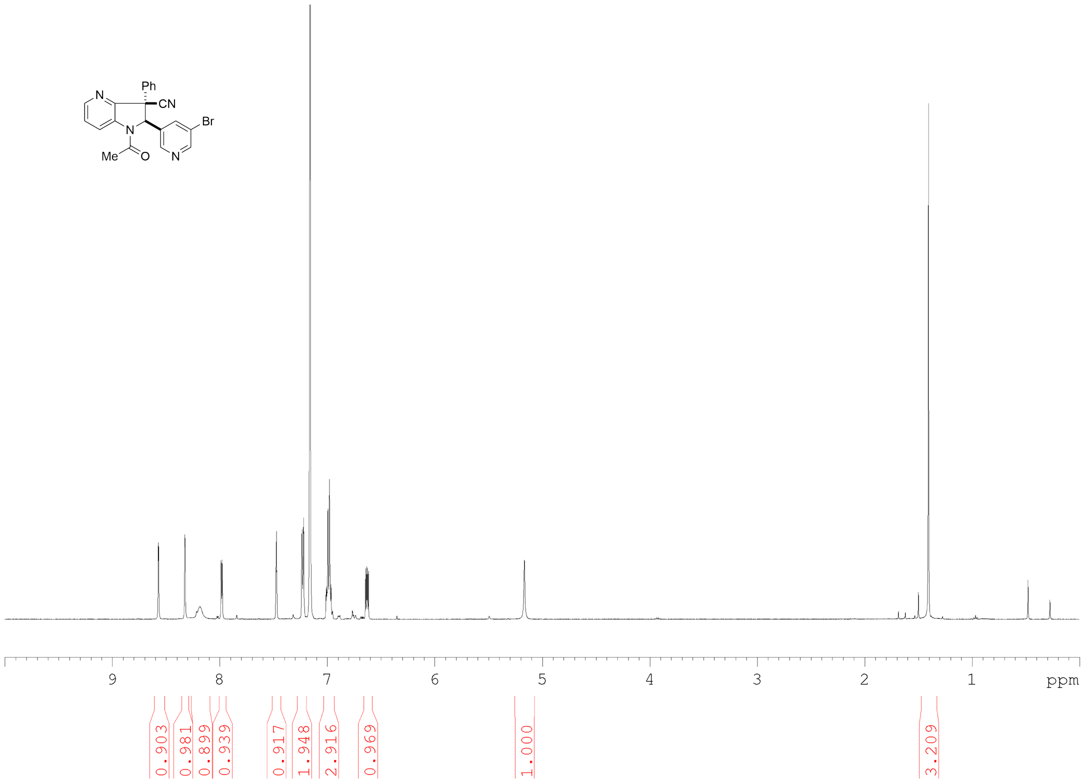
Ph
CN
N
N
Br
Me
O
N
9
8
7
6
5
4
3
2
1
ppm
0.903
0.981
0.899
0.939
0.917
1.948
2.916
0.969
1.000
3.209

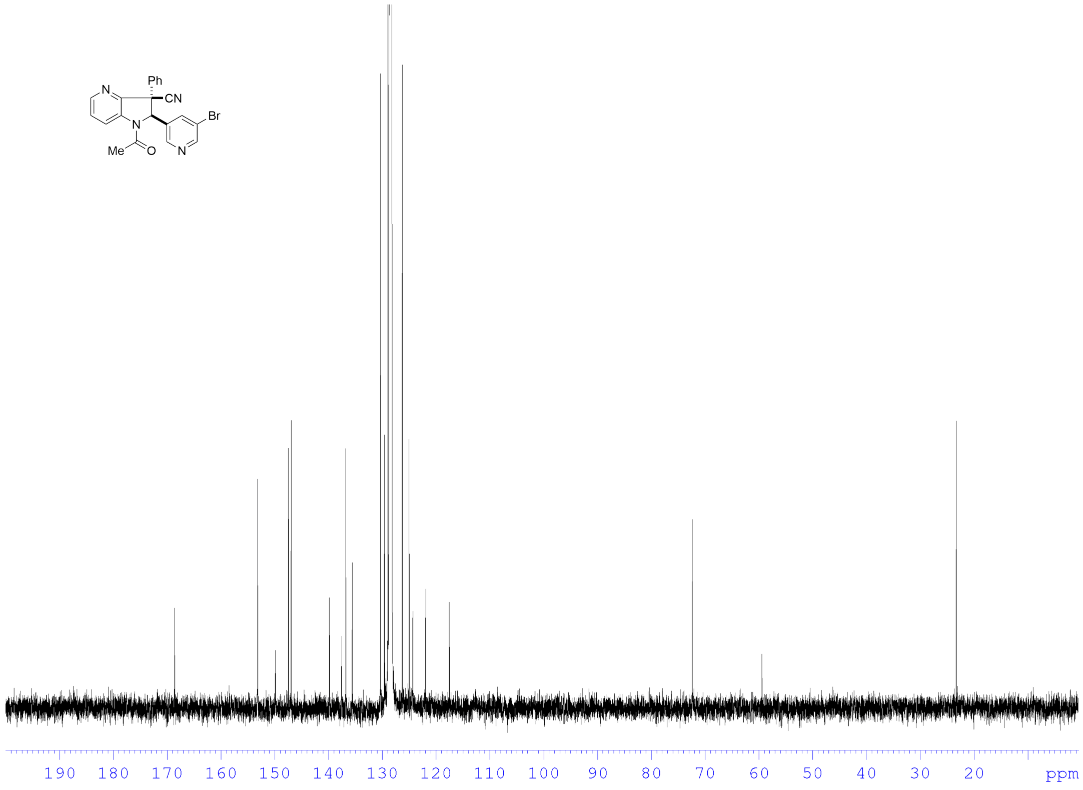
Ph
CN
N
N
Br
Me
O
N
190
180
170
160
150
140
130
120
110
100
90
80
70
60
50
40
30
20
ppm

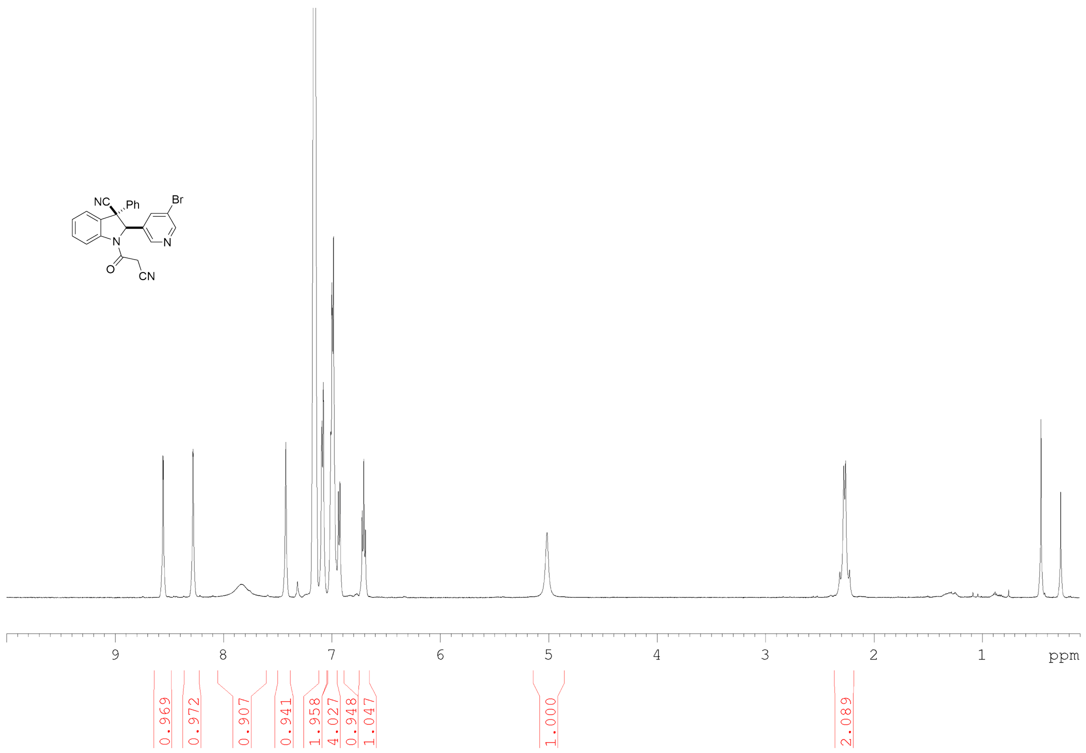
NC
Ph
Br
N
N
O
CN
9
8
7
6
5
4
3
2
1
ppm
0.969
0.972
0.907
0.941
1.958
4.027
0.948
1.047
1.000
2.089

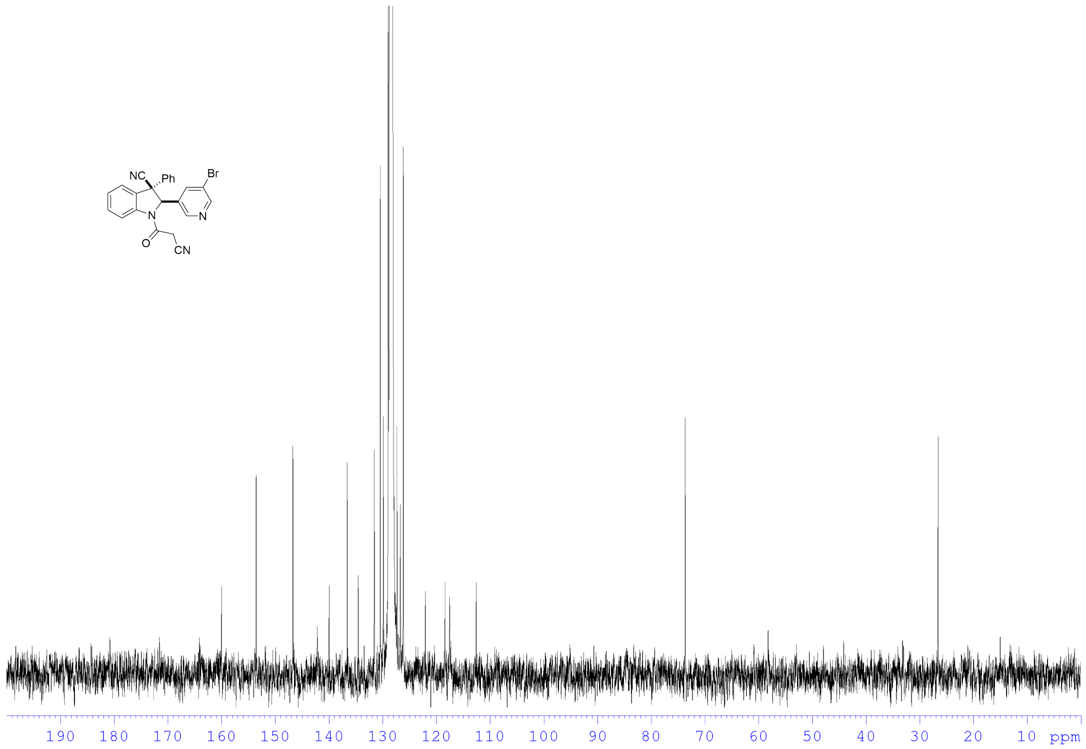
NC
Ph
Br
N
N
O
CN
190
180
170
160
150
140
130
120
110
100
90
80
70
60
50
40
30
20
10
ppm

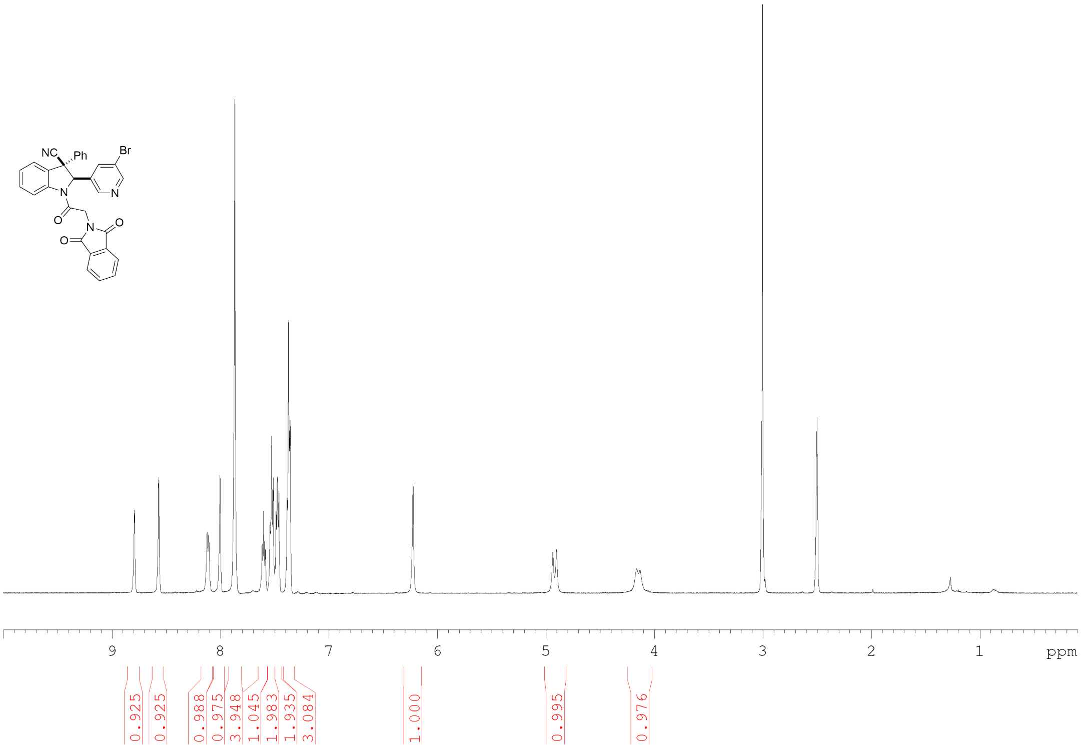
NC
Ph
Br
N
O
9
8
7
6
5
4
3
2
1
ppm
0.925
0.925
0.988
0.975
3.948
1.045
1.983
1.935
3.084
1.000
0.995
0.976

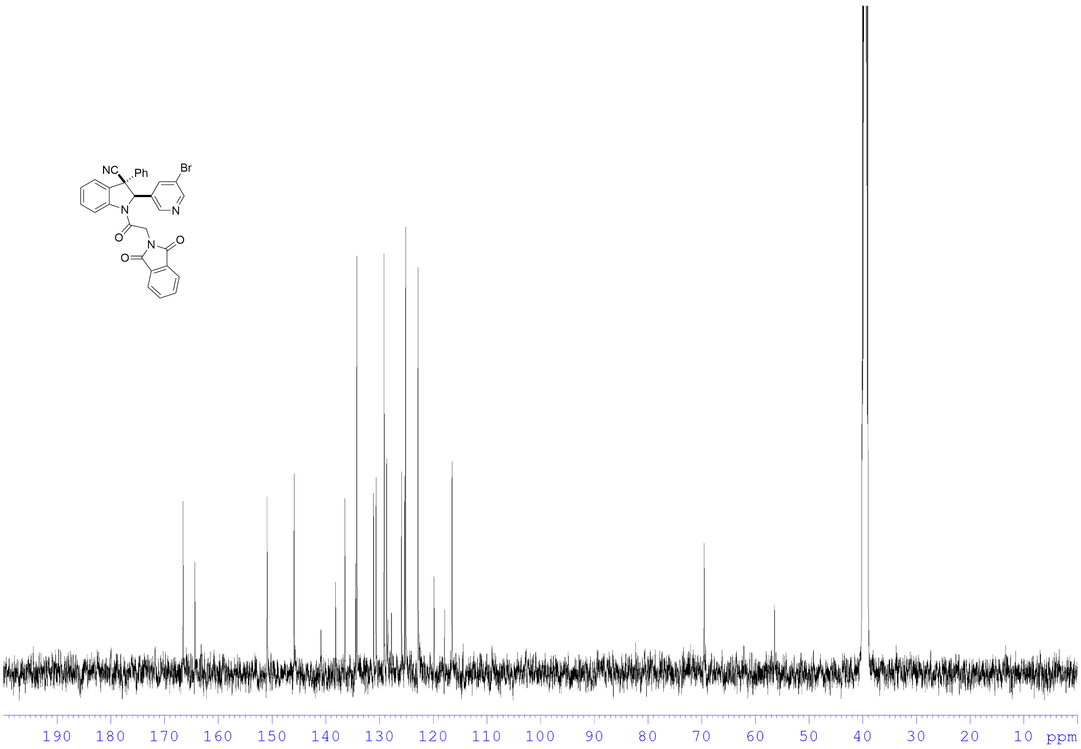
NC
Ph
Br
N
O
190
180
170
160
150
140
130
120
110
100
90
80
70
60
50
40
30
20
10
ppm

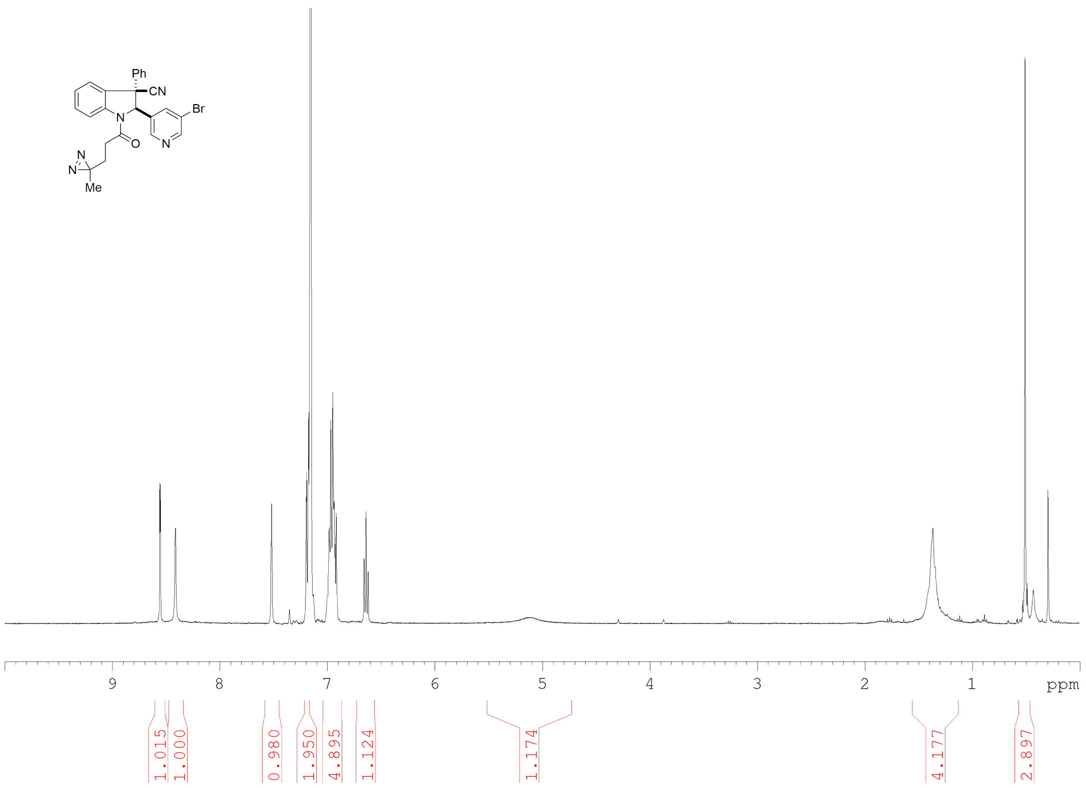
Ph
CN
N
Br
N
O
N
N
Me
9 8 7 6 5 4 3 2 1 ppm
1.015
1.000
0.980
1.950
4.895
1.124
1.174
4.177
2.897

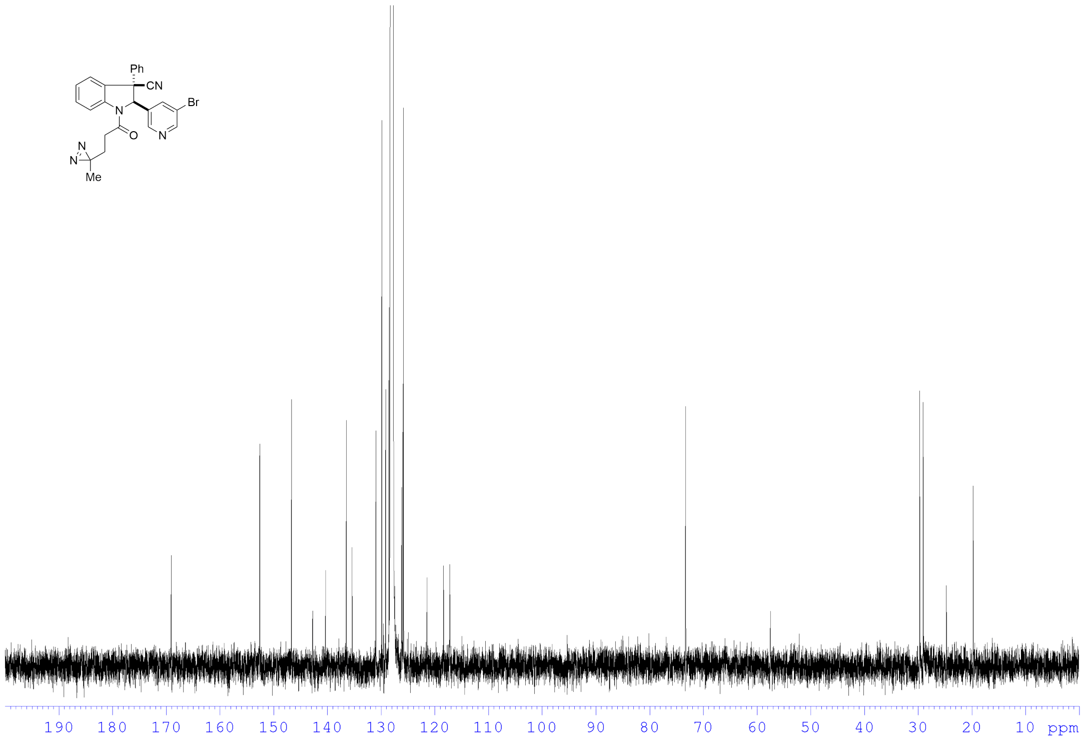
Ph
CN
N
Br
N
O
N
N
Me
190 180 170 160 150 140 130 120 110 100 90 80 70 60 50 40 30 20 10 ppm

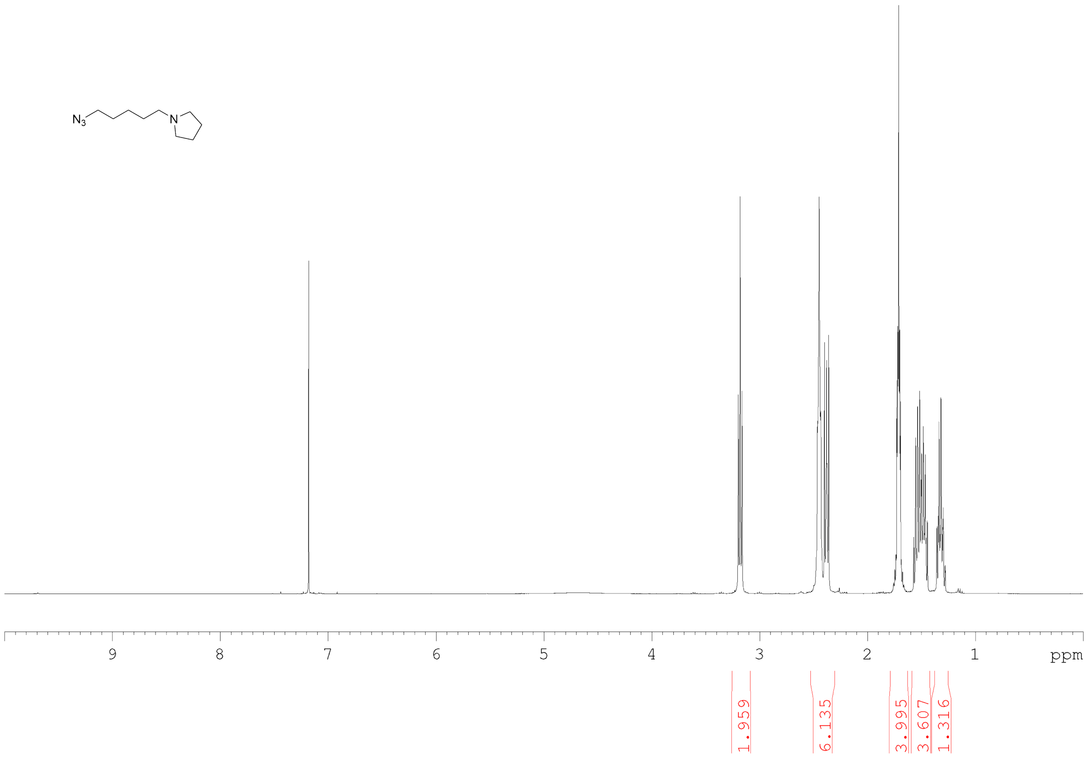
N3
N
9
8
7
6
5
4
3
2
1
ppm
1.959
6.135
3.995
3.607
1.316

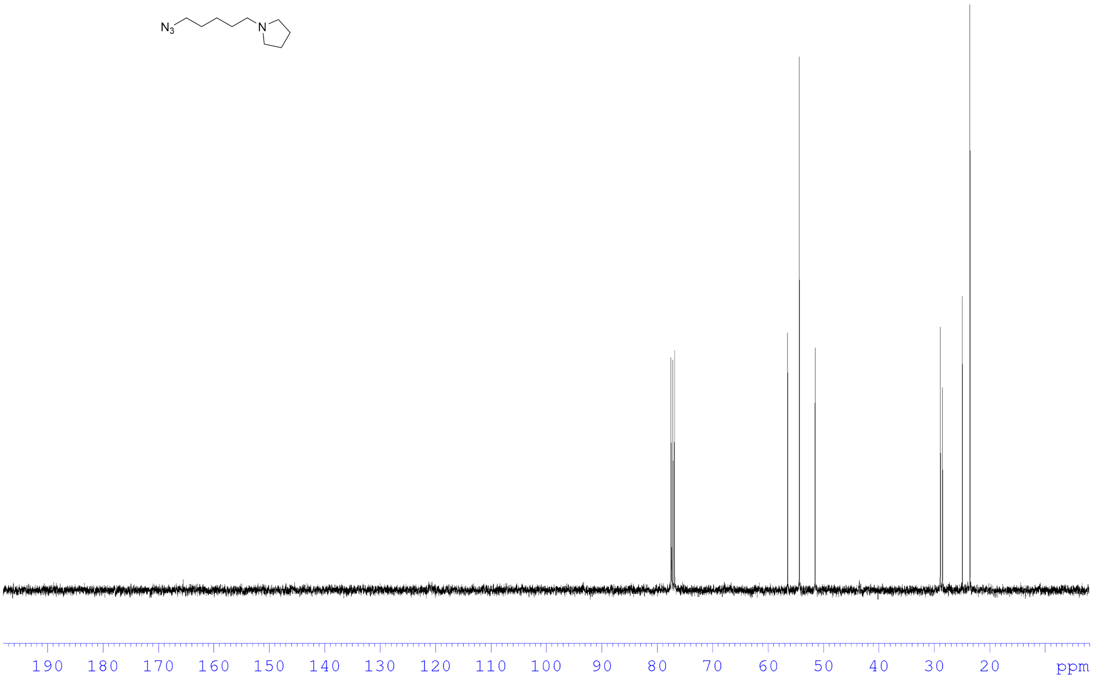
N3
N
190
180
170
160
150
140
130
120
110
100
90
80
70
60
50
40
30
20
ppm

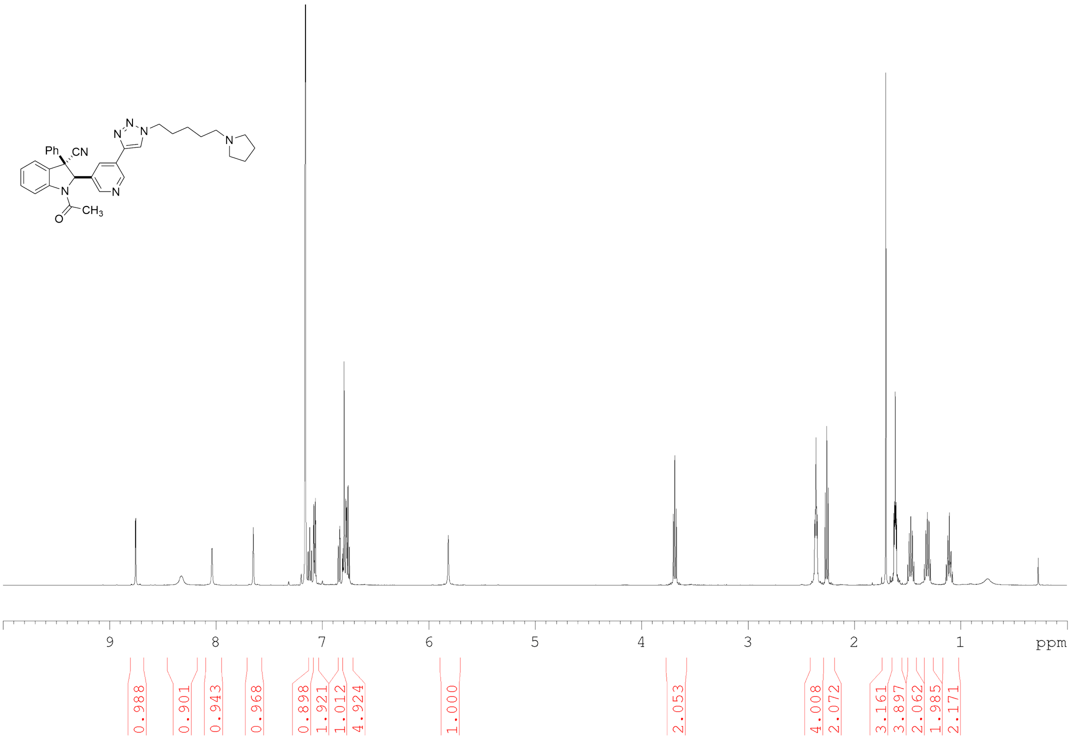
Ph
CN
N
O
CH3
9 8 7 6 5 4 3 2 1 ppm
0.988 0.901 0.943 0.968 0.898 1.921 1.012 4.924 1.000 2.053 4.008 2.072 3.161 3.897 2.062 1.985 2.171

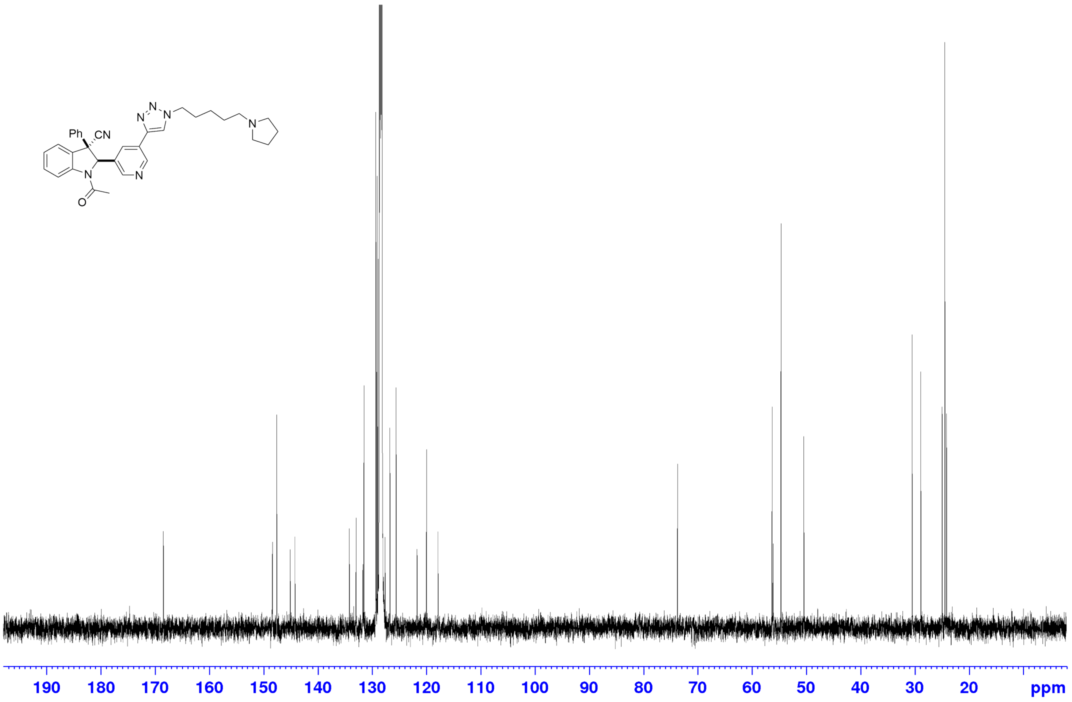
Ph
CN
N
O
190 180 170 160 150 140 130 120 110 100 90 80 70 60 50 40 30 20 ppm

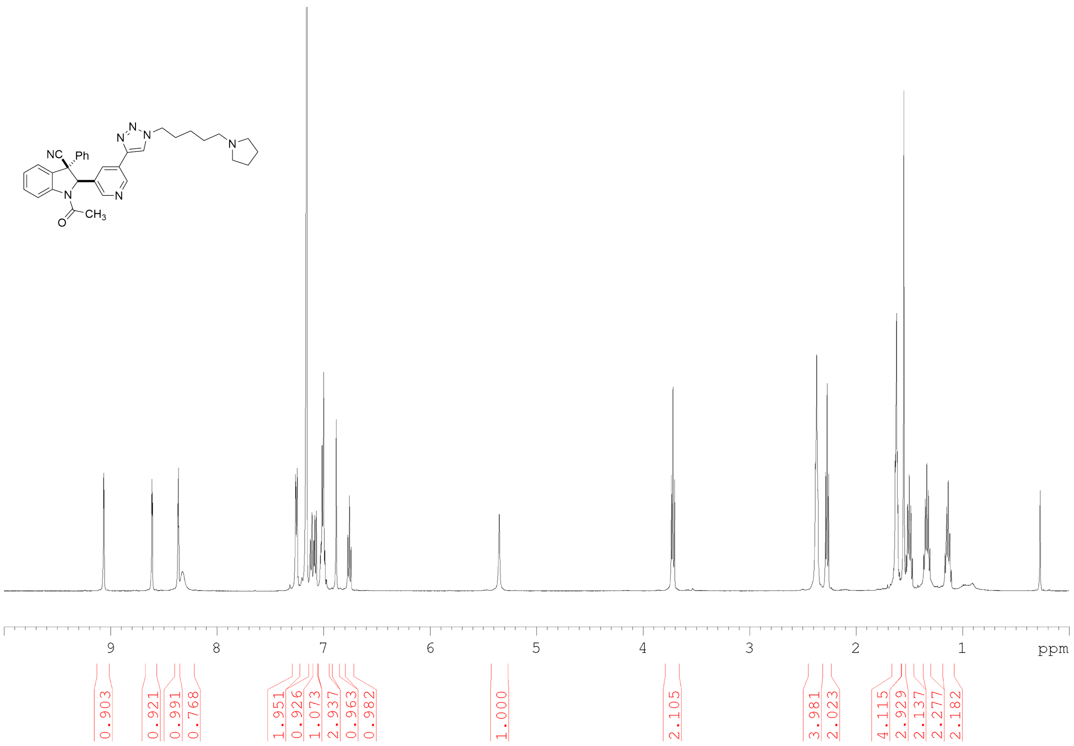
NC
Ph
N
N
N
N
N
N
O
CH3
9
8
7
6
5
4
3
2
1
ppm
0.903
0.921
0.991
0.768
1.951
0.926
1.073
2.937
0.963
0.982
1.000
2.105
3.981
2.023
4.115
2.929
2.137
2.277
2.182

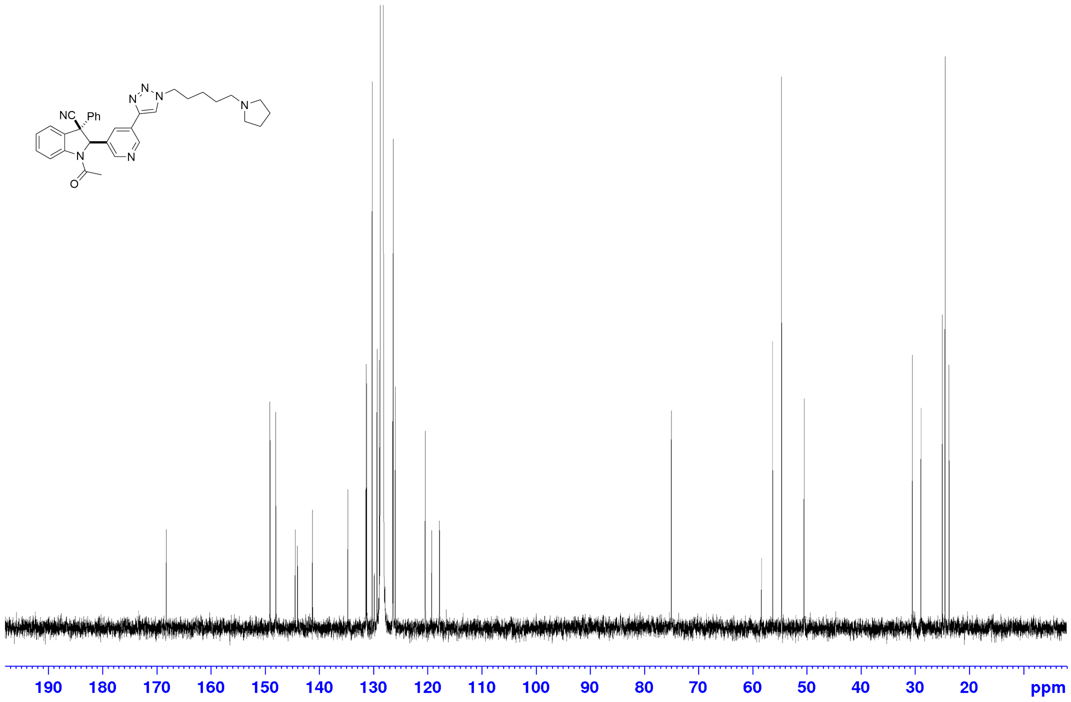
NC
Ph
N
N
N
N
N
N
O
190
180
170
160
150
140
130
120
110
100
90
80
70
60
50
40
30
20
ppm

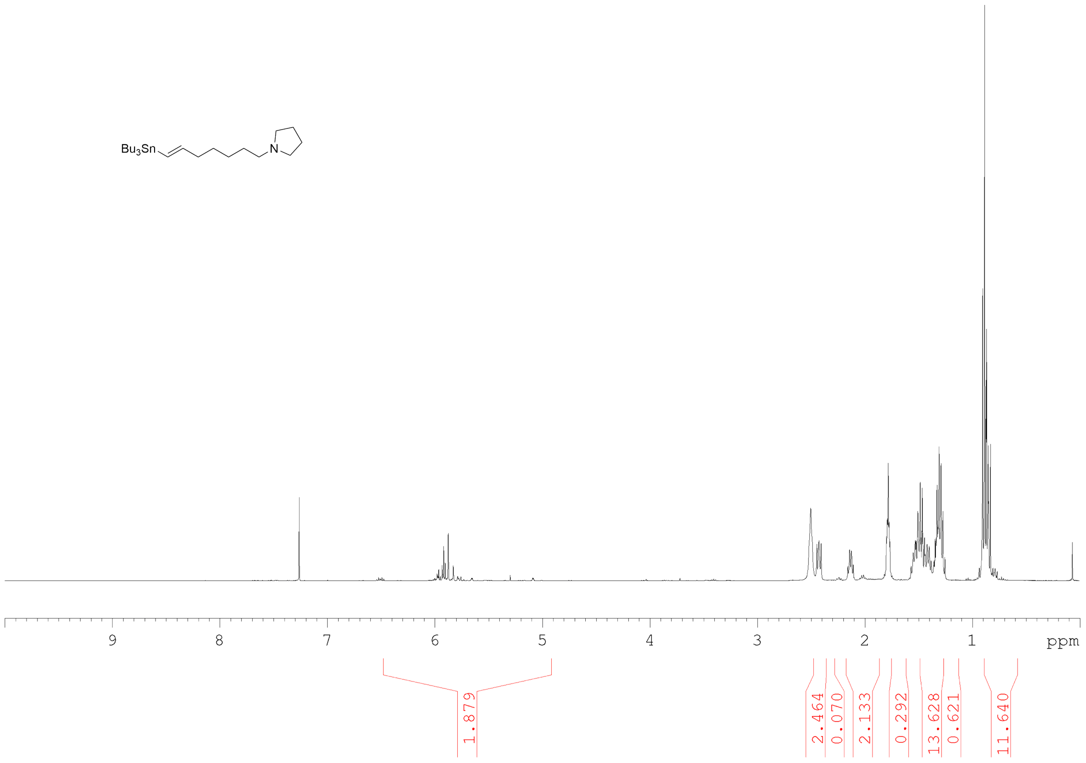
Bu3Sn
N
9
8
7
6
5
4
3
2
1
ppm
1.879
2.464
0.070
2.133
0.292
13.628
0.621
11.640

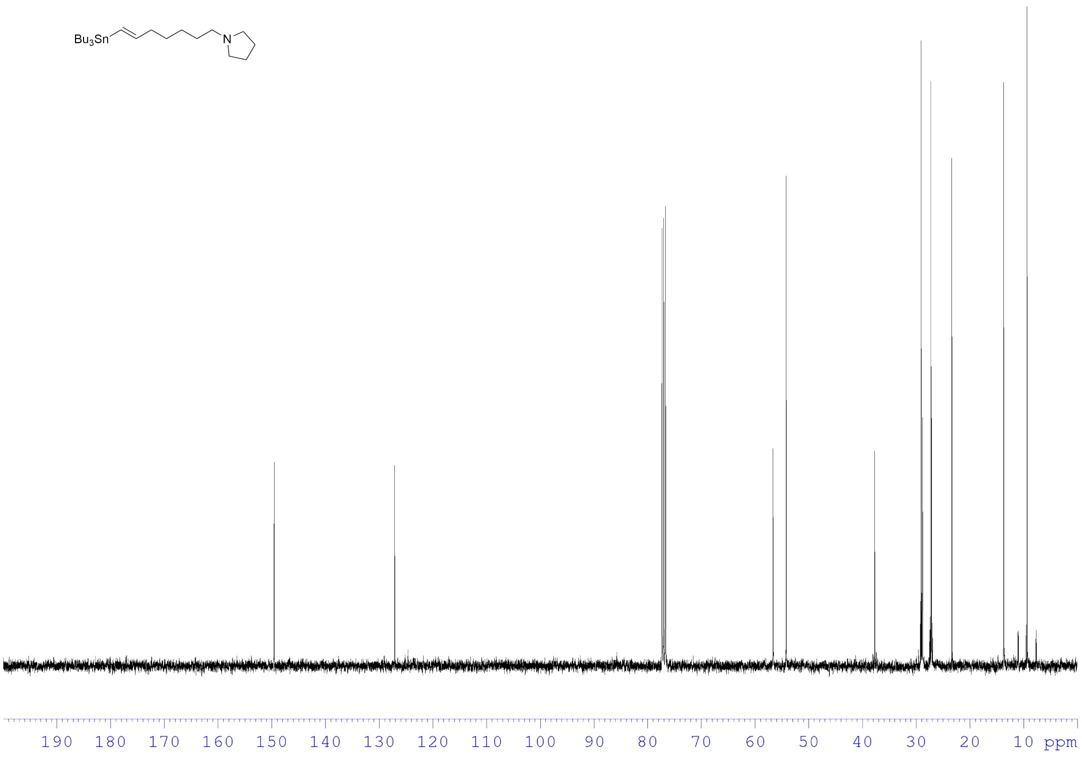
Bu3Sn
N
190
180
170
160
150
140
130
120
110
100
90
80
70
60
50
40
30
20
10
ppm

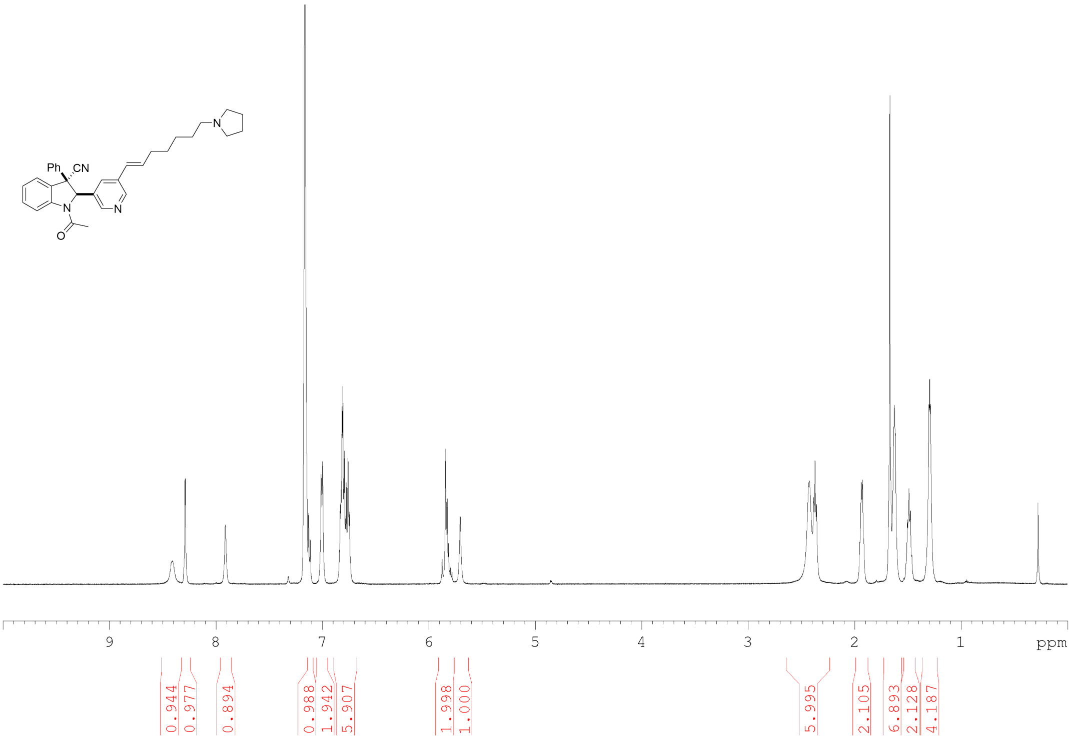
Ph
CN
N
N
O
N
9
8
7
6
5
4
3
2
1
ppm
0.944
0.977
0.894
0.988
1.942
5.907
1.998
1.000
5.995
2.105
6.893
2.128
4.187

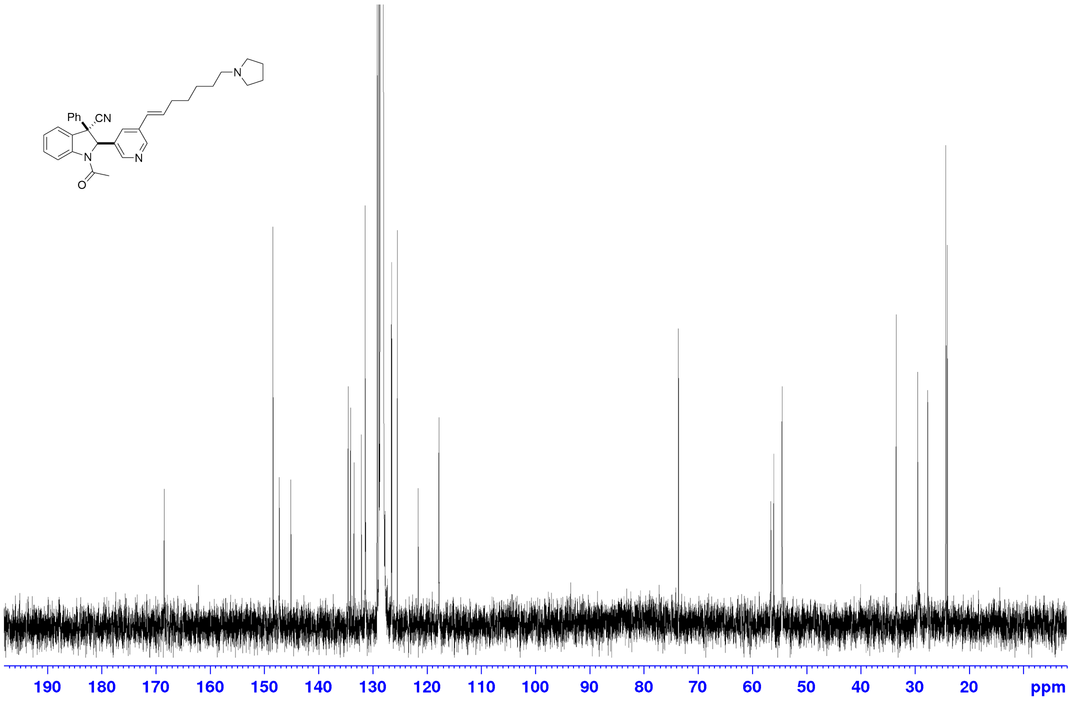
Ph
CN
N
N
O
N
190
180
170
160
150
140
130
120
110
100
90
80
70
60
50
40
30
20
ppm

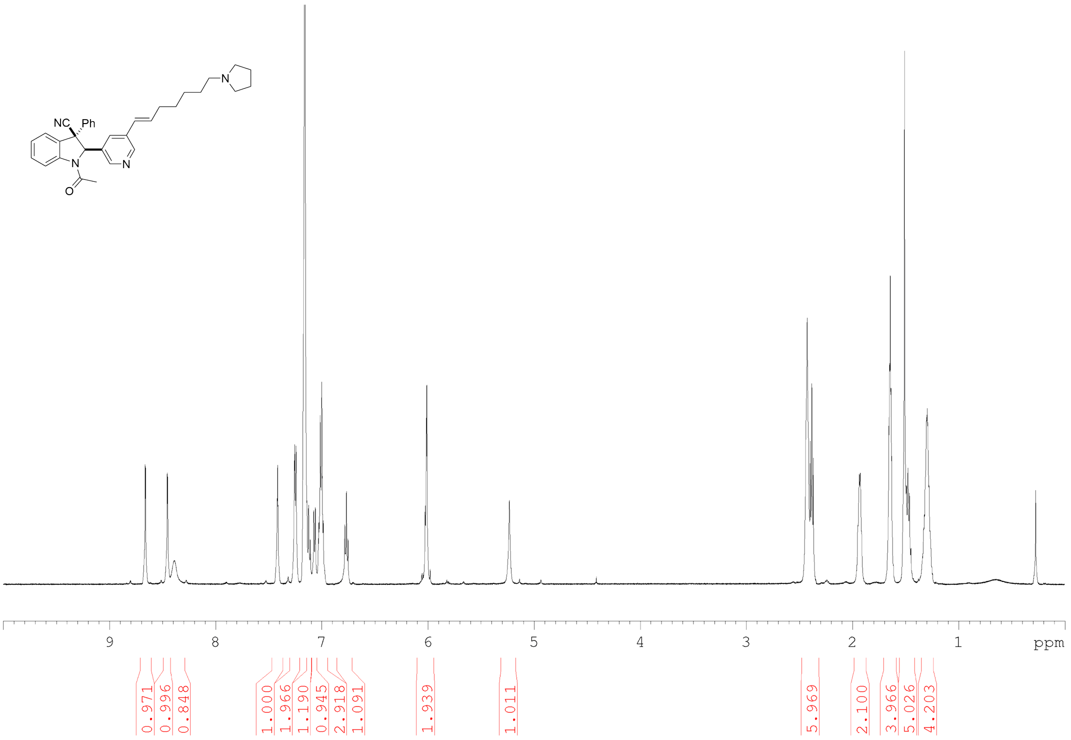

NC
Ph
N
O
N
N
9
8
7
6
5
4
3
2
1
ppm
0.971
0.996
0.848
1.000
1.966
1.190
0.945
2.918
1.091
1.939
1.011
5.969
2.100
3.966
5.026
4.203

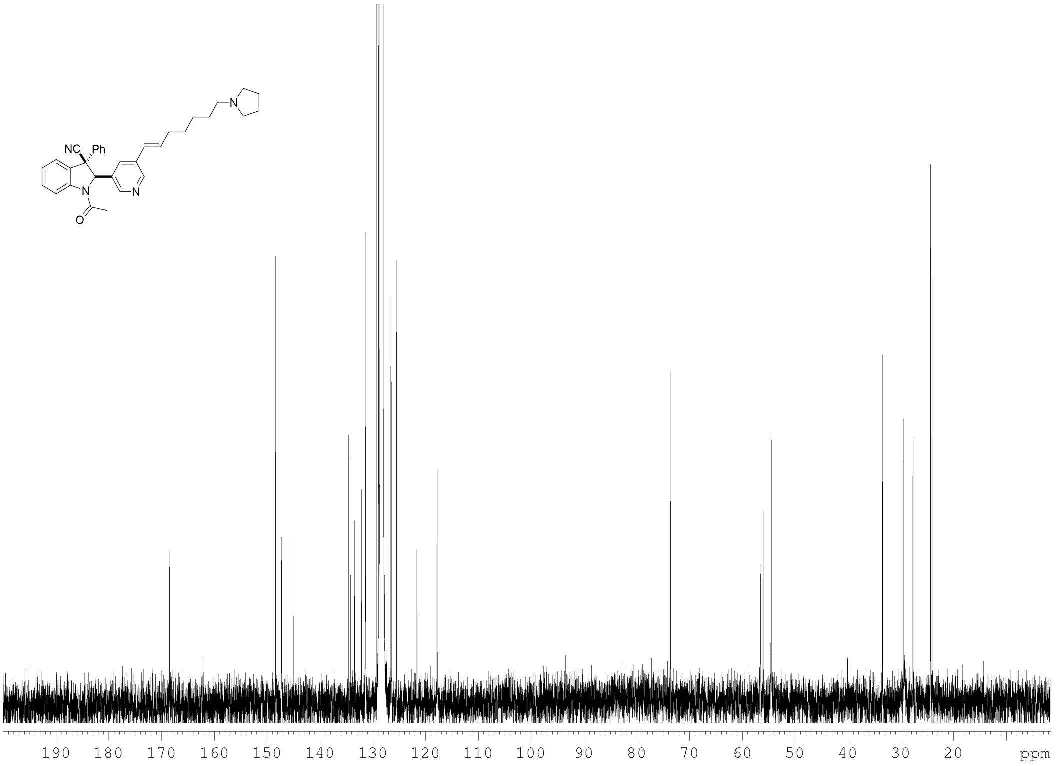

NC
Ph
N
O
N
N
190
180
170
160
150
140
130
120
110
100
90
80
70
60
50
40
30
20
ppm

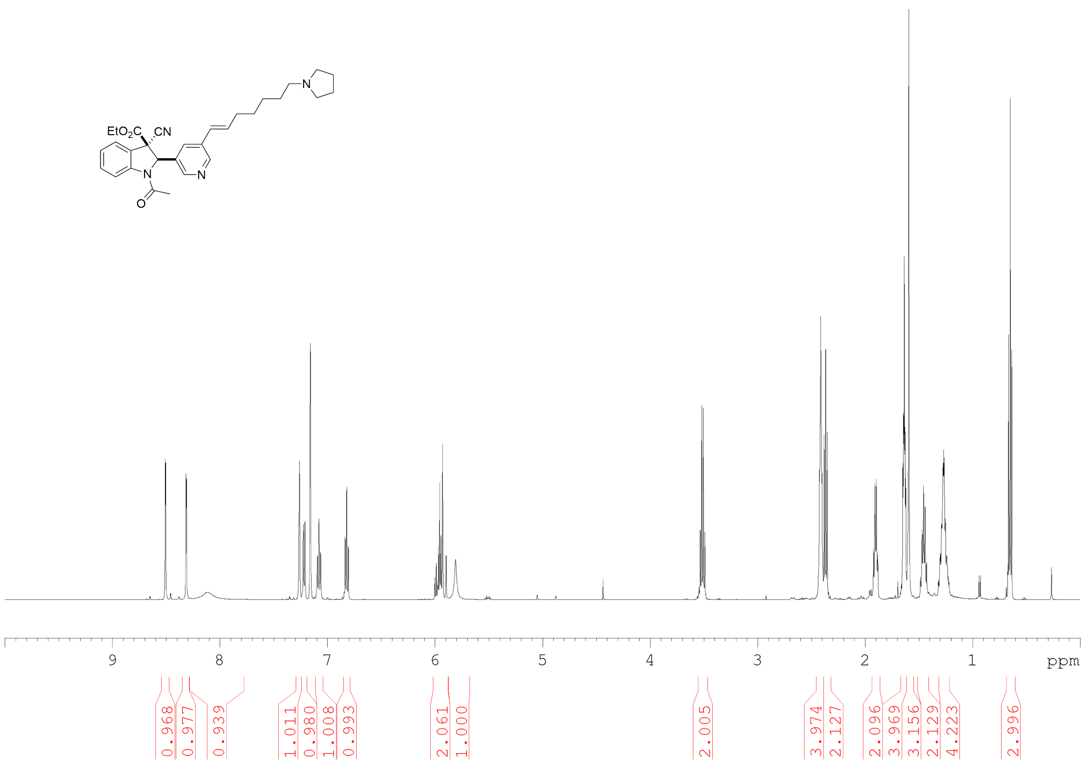
EtO2C
CN
N
O
N
N
9
8
7
6
5
4
3
2
1
ppm
0.968
0.977
0.939
1.011
0.980
1.008
0.993
2.061
1.000
2.005
3.974
2.127
2.096
3.969
3.156
2.129
4.223
2.996

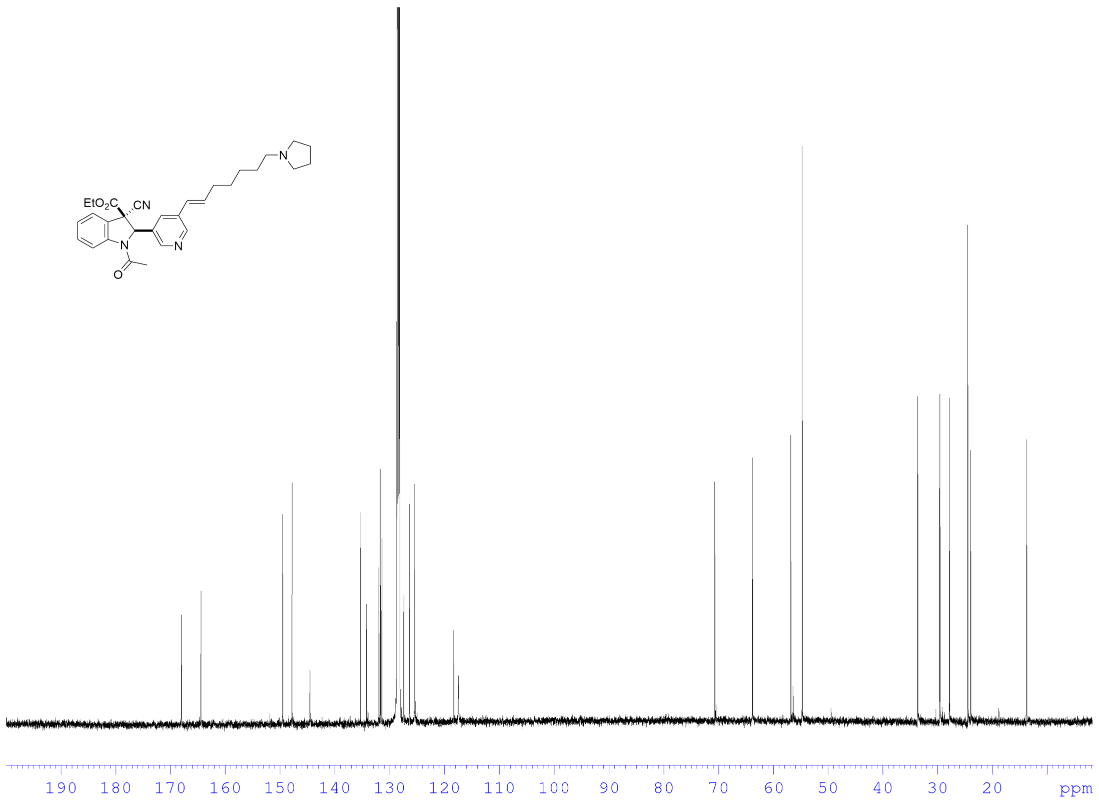
EtO2C
CN
N
O
N
N
190
180
170
160
150
140
130
120
110
100
90
80
70
60
50
40
30
20
ppm

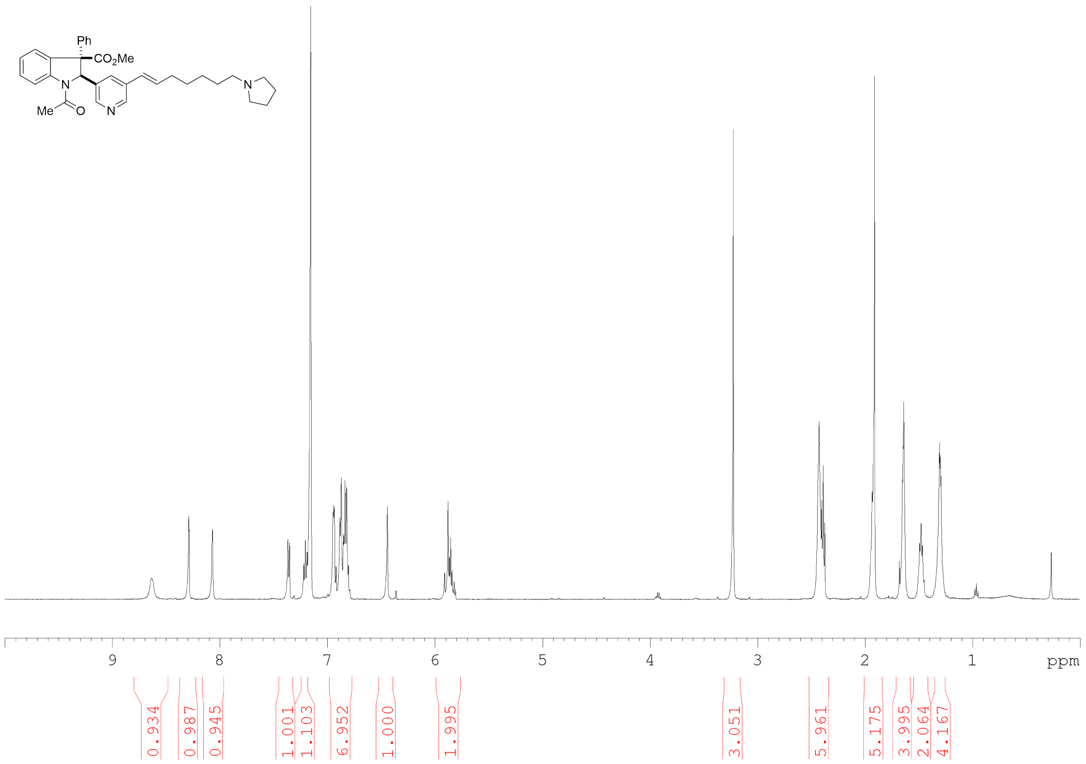
Ph
CO2Me
N
Me
O
N
N
9
8
7
6
5
4
3
2
1
ppm
0.934
0.987
0.945
1.001
1.103
6.952
1.000
1.995
3.051
5.961
5.175
3.995
2.064
4.167

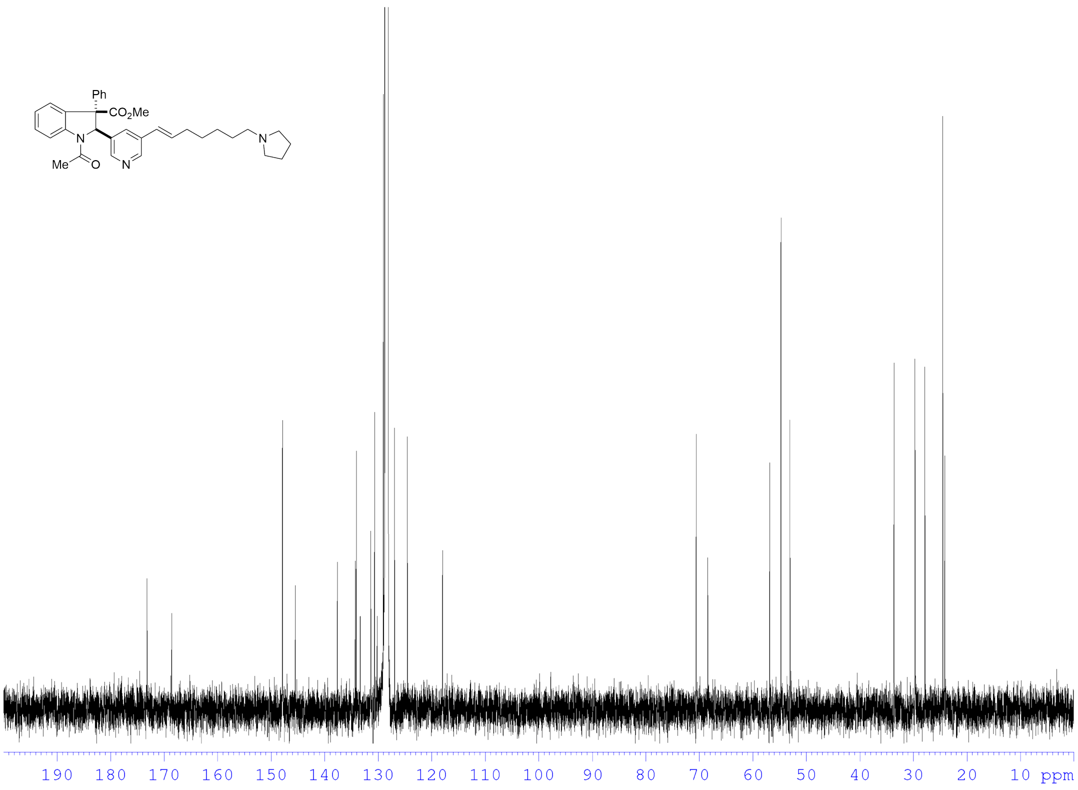
Ph
CO2Me
N
Me
O
N
N
190
180
170
160
150
140
130
120
110
100
90
80
70
60
50
40
30
20
10
ppm

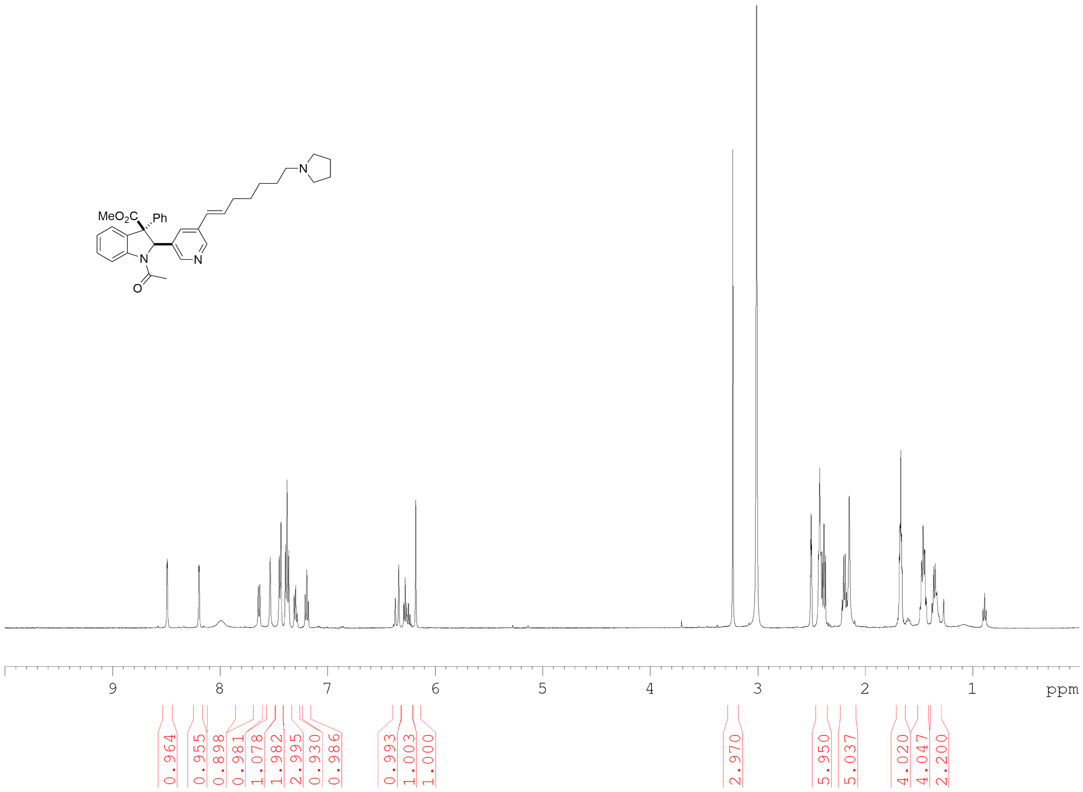
MeO2C
Ph
N
N
O
9
8
7
6
5
4
3
2
1
ppm
0.964
0.955
0.898
0.981
1.078
1.982
2.995
0.930
0.986
0.993
1.003
1.000
2.970
5.950
5.037
4.020
4.047
2.200

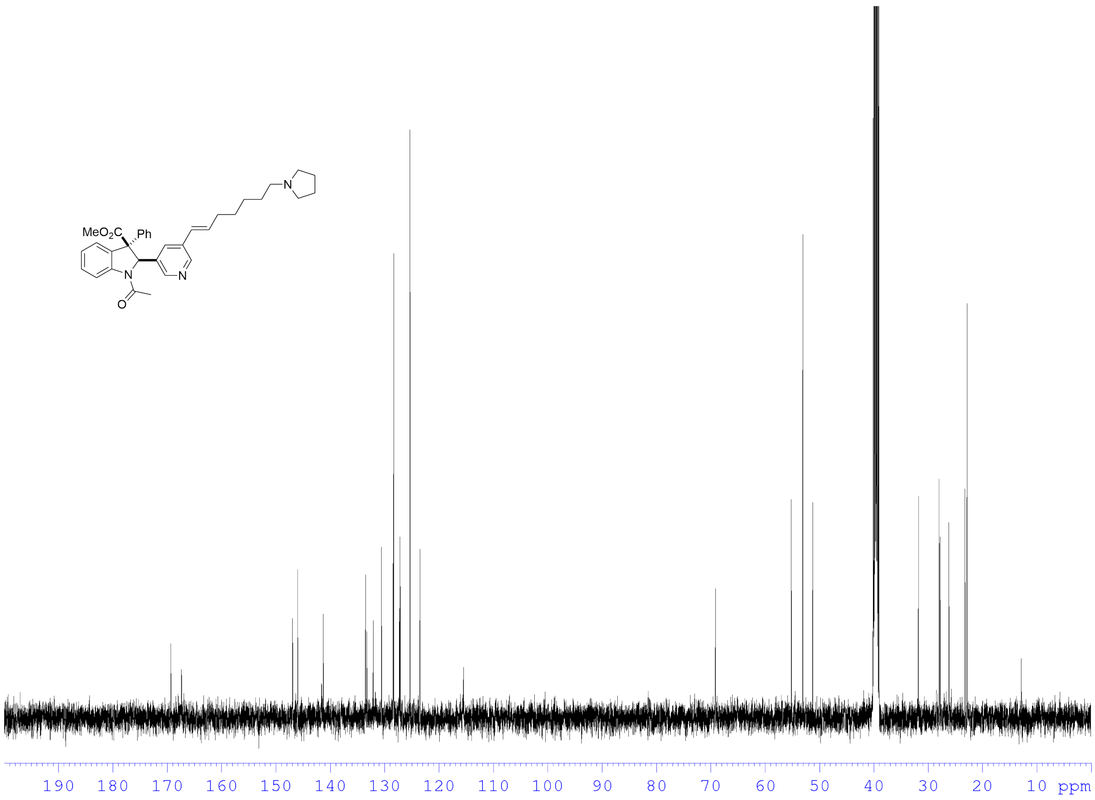
MeO2C
Ph
N
N
O
190
180
170
160
150
140
130
120
110
100
90
80
70
60
50
40
30
20
10
ppm

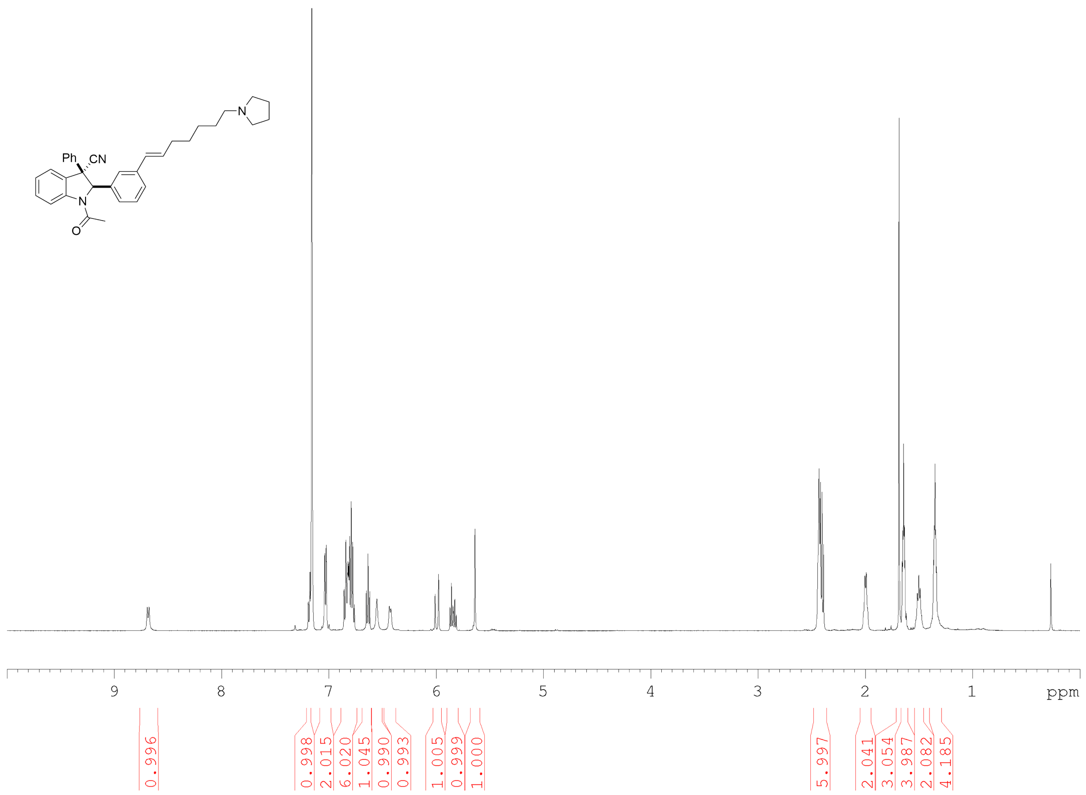
Ph
CN
N
O
9 8 7 6 5 4 3 2 1 ppm
0.996
0.998 2.015 6.020 1.045 0.990 0.993
1.005 0.999 1.000
5.997
2.041 3.054 3.987 2.082 4.185

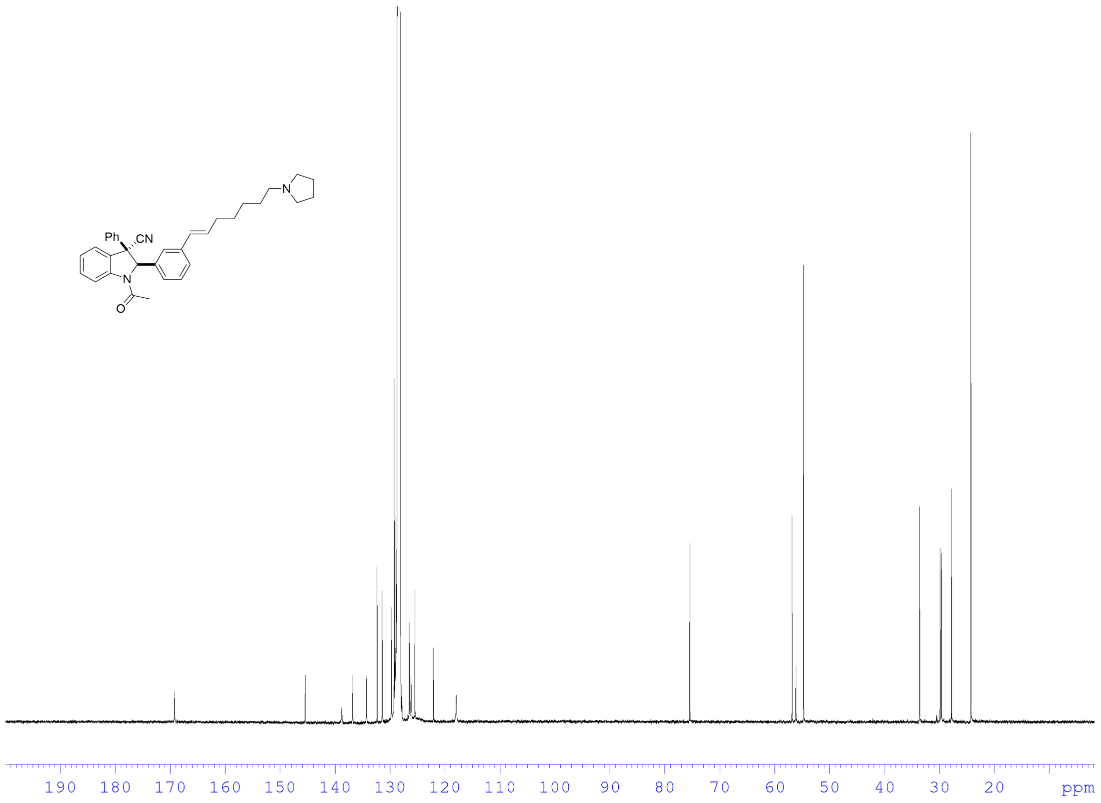
Ph
CN
N
O
190 180 170 160 150 140 130 120 110 100 90 80 70 60 50 40 30 20 ppm

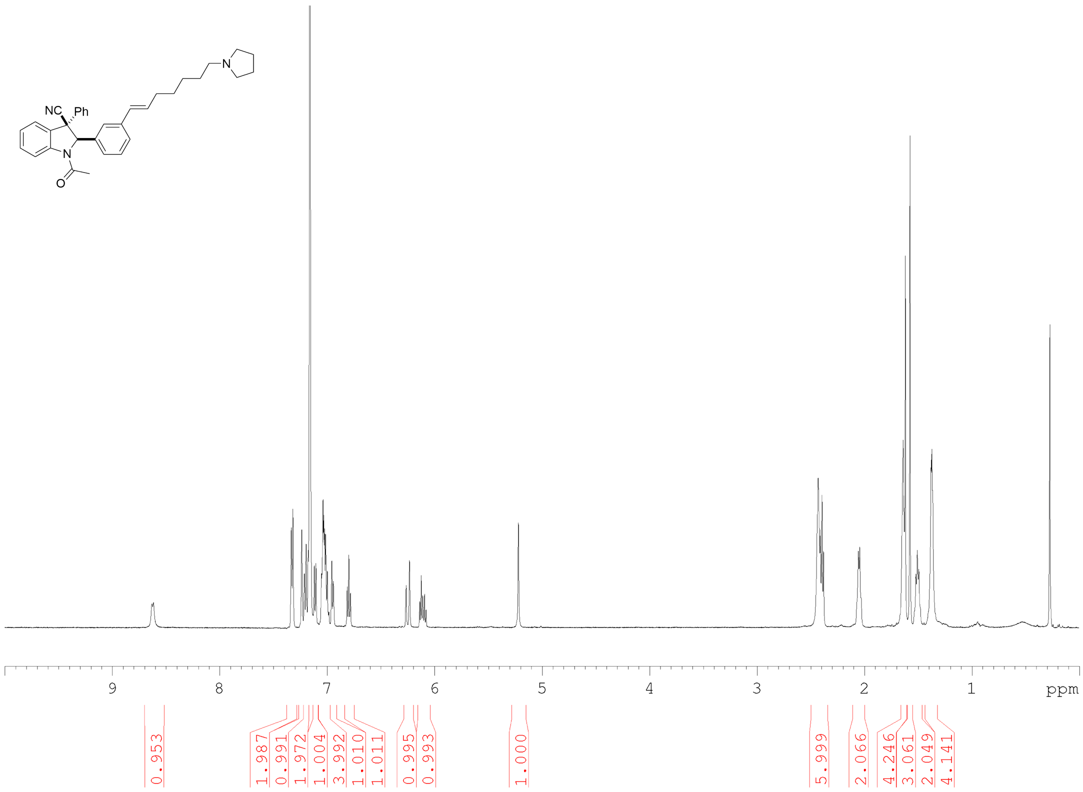
NC
Ph
N
O
N
9
8
7
6
5
4
3
2
1
ppm
0.953
1.987
0.991
1.972
1.004
3.992
1.010
1.011
0.995
0.993
1.000
5.999
2.066
4.246
3.061
2.049
4.141

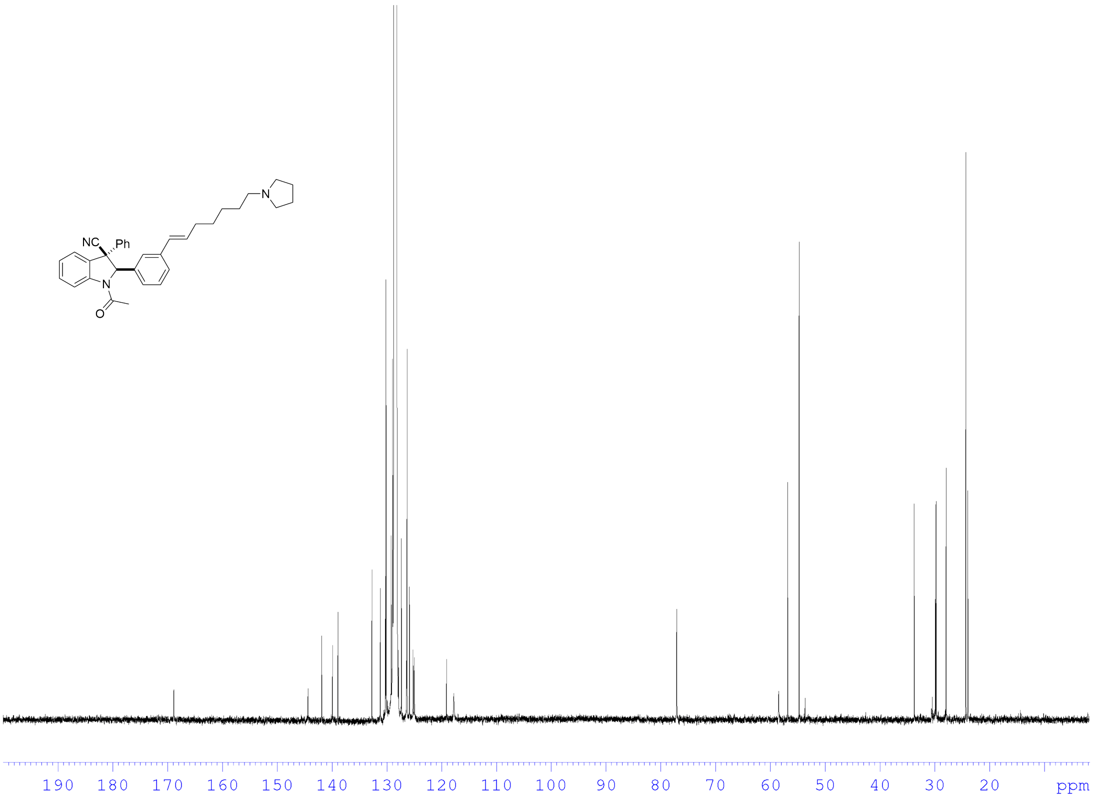
NC
Ph
N
O
N
190
180
170
160
150
140
130
120
110
100
90
80
70
60
50
40
30
20
ppm

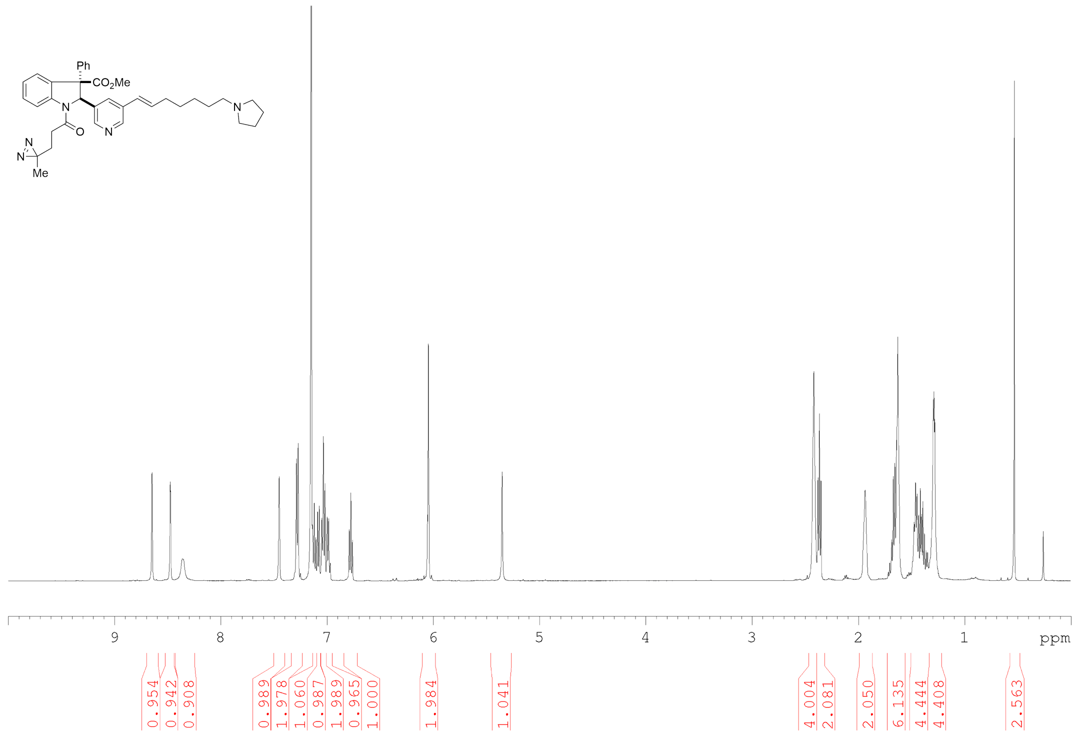
Ph
CO2Me
N
O
N
N
N
Me
N
9
8
7
6
5
4
3
2
1
ppm
0.954
0.942
0.908
0.989
1.978
1.060
0.987
1.989
0.965
1.000
1.984
1.041
4.004
2.081
2.050
6.135
4.444
4.408
2.563

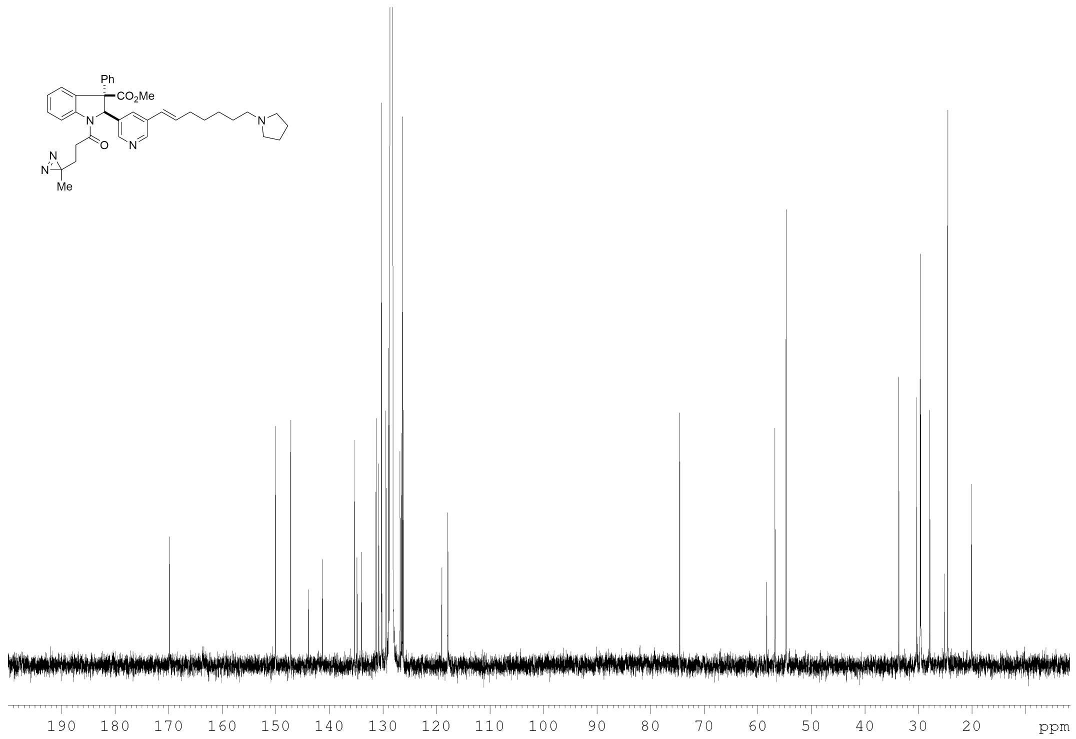
Ph
CO2Me
N
O
N
N
N
Me
N
190
180
170
160
150
140
130
120
110
100
90
80
70
60
50
40
30
20
ppm

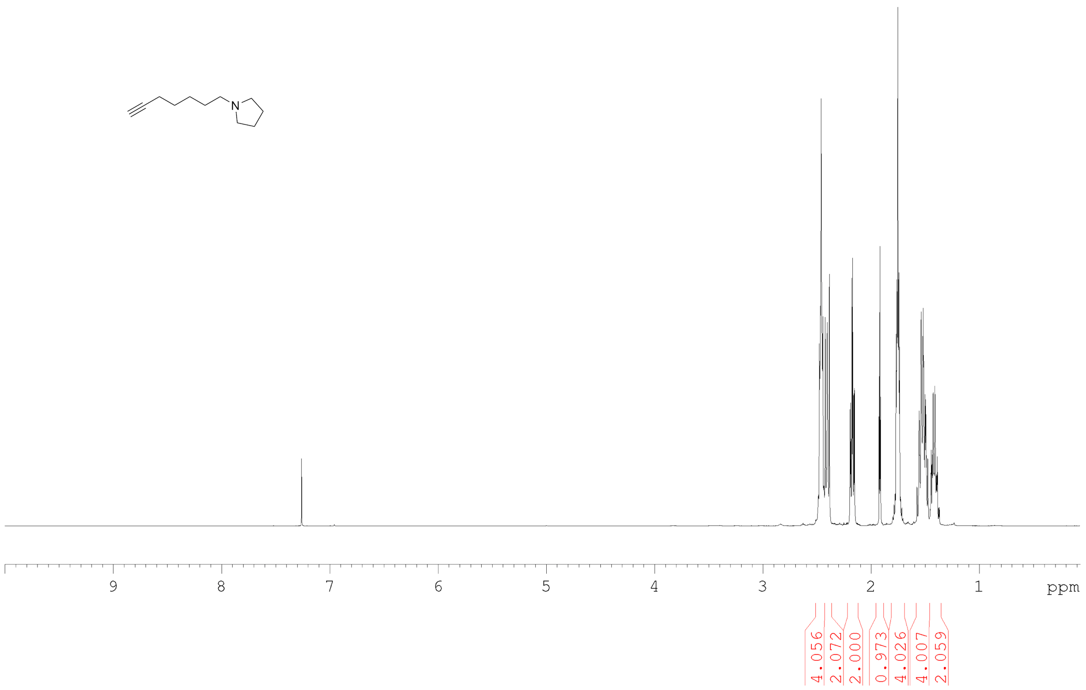

N
9
8
7
6
5
4
3
2
1
ppm
4.056
2.072
2.000
0.973
4.026
4.007
2.059

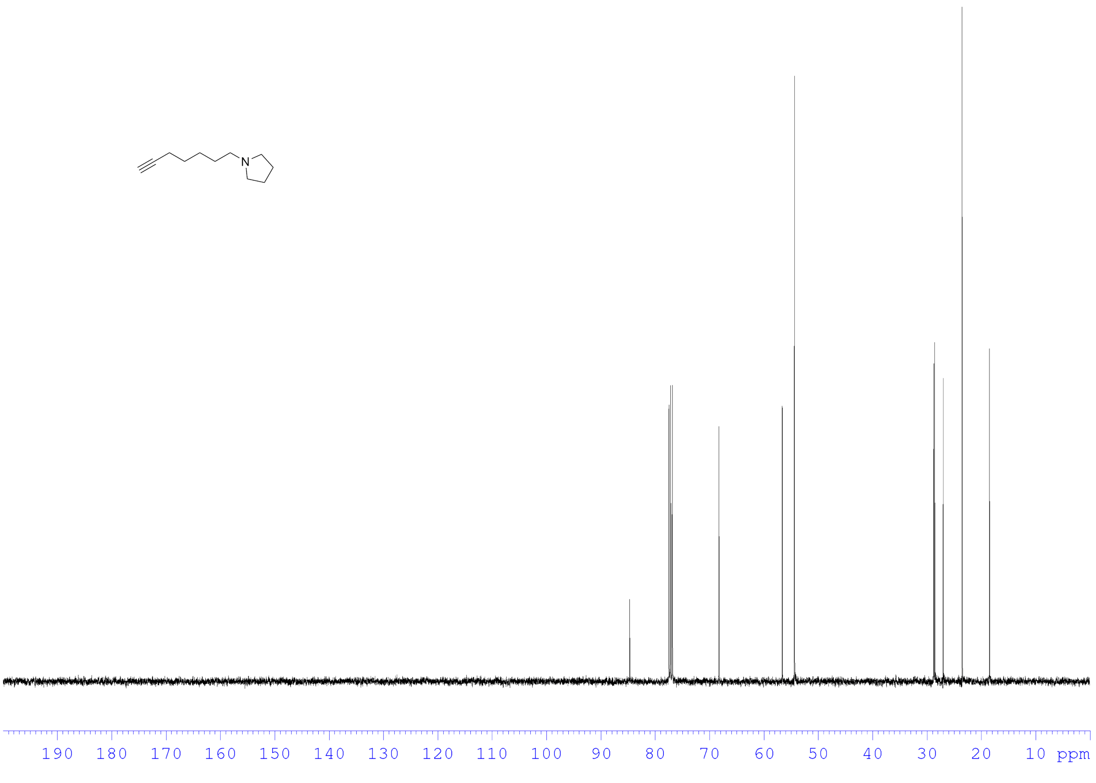

N
190
180
170
160
150
140
130
120
110
100
90
80
70
60
50
40
30
20
10
ppm

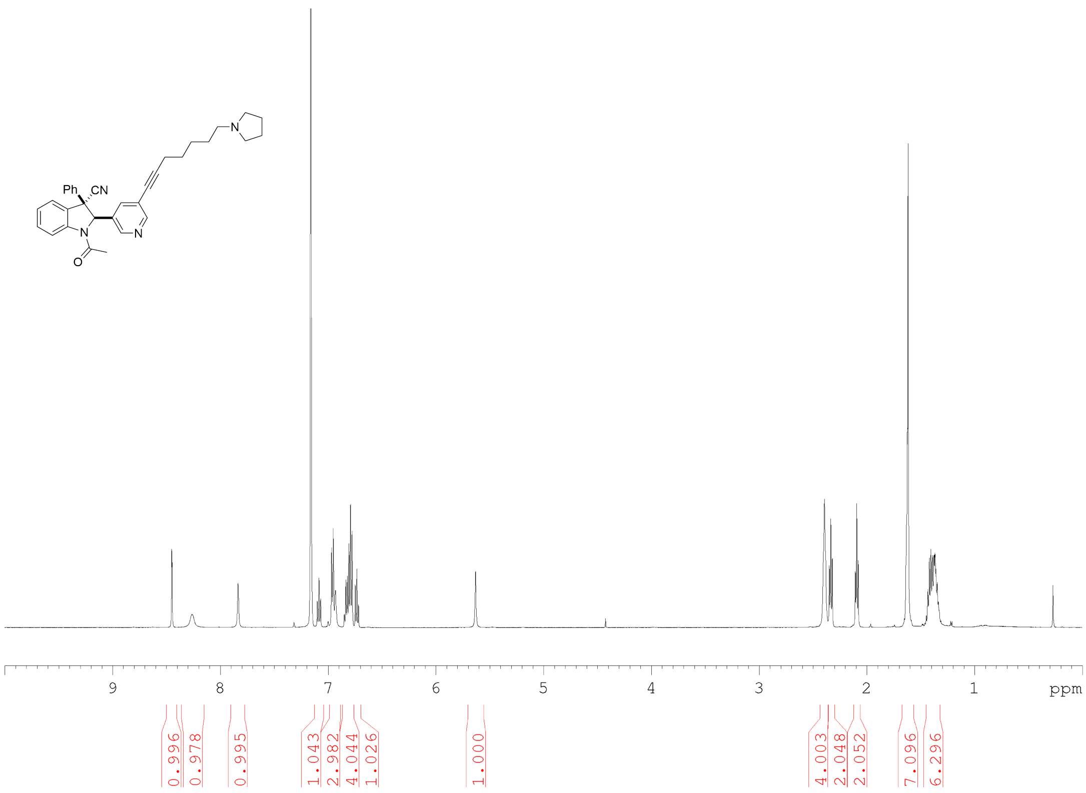
Ph
CN
N
O
9
8
7
6
5
4
3
2
1
ppm
0.996
0.978
0.995
1.043
2.982
4.044
1.026
1.000
4.003
2.048
2.052
7.096
6.296

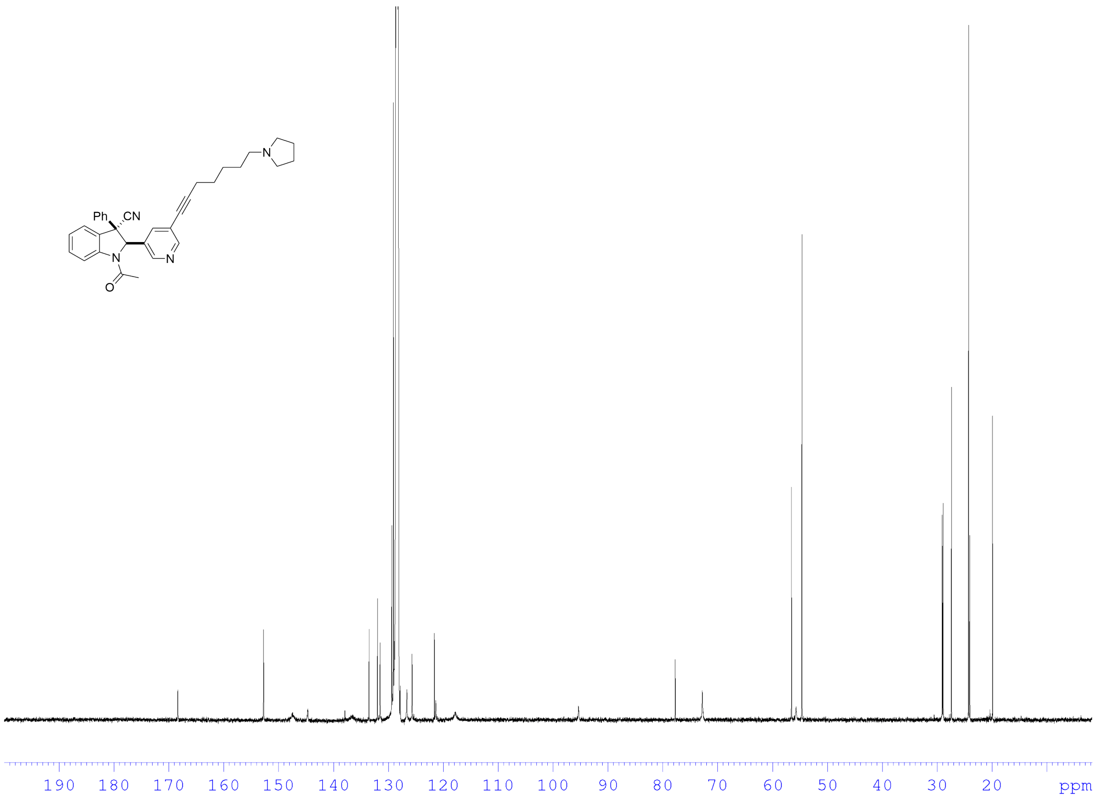
Ph
CN
N
O
190
180
170
160
150
140
130
120
110
100
90
80
70
60
50
40
30
20
ppm

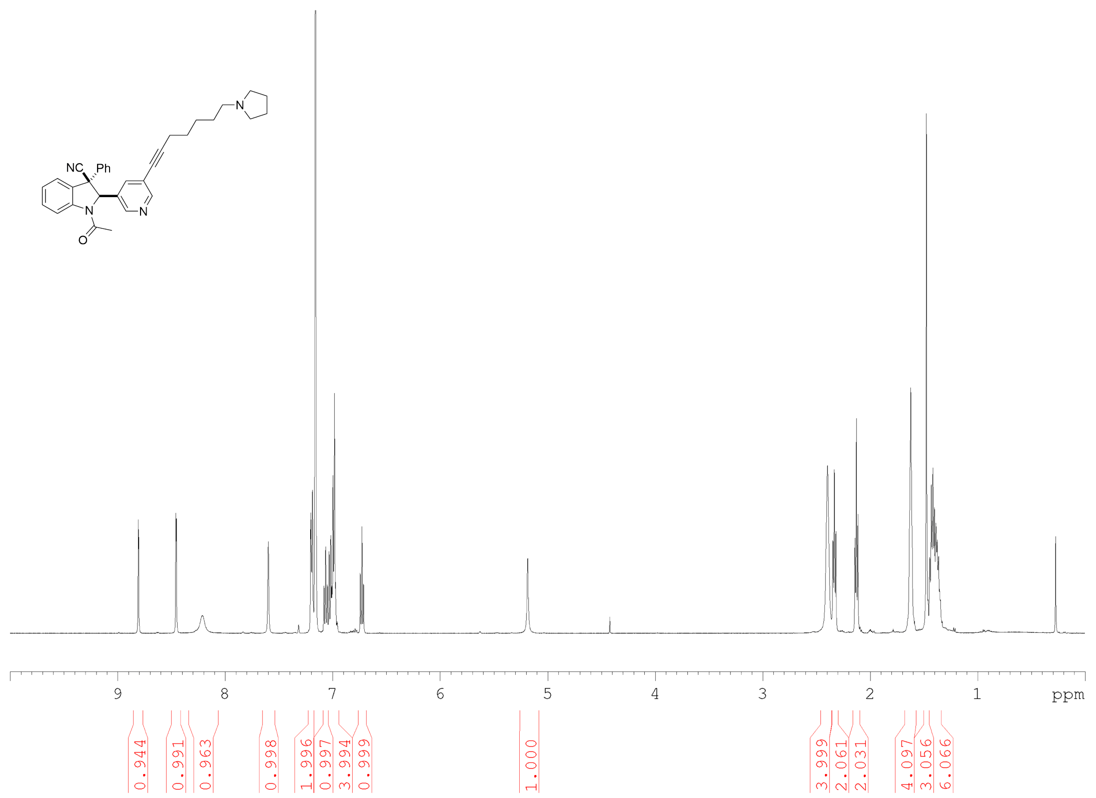
NC
Ph
N
N
O
9 8 7 6 5 4 3 2 1 ppm
0.944
0.991
0.963
0.998
1.996
0.997
3.994
0.999
1.000
3.999
2.061
2.031
4.097
3.056
6.066

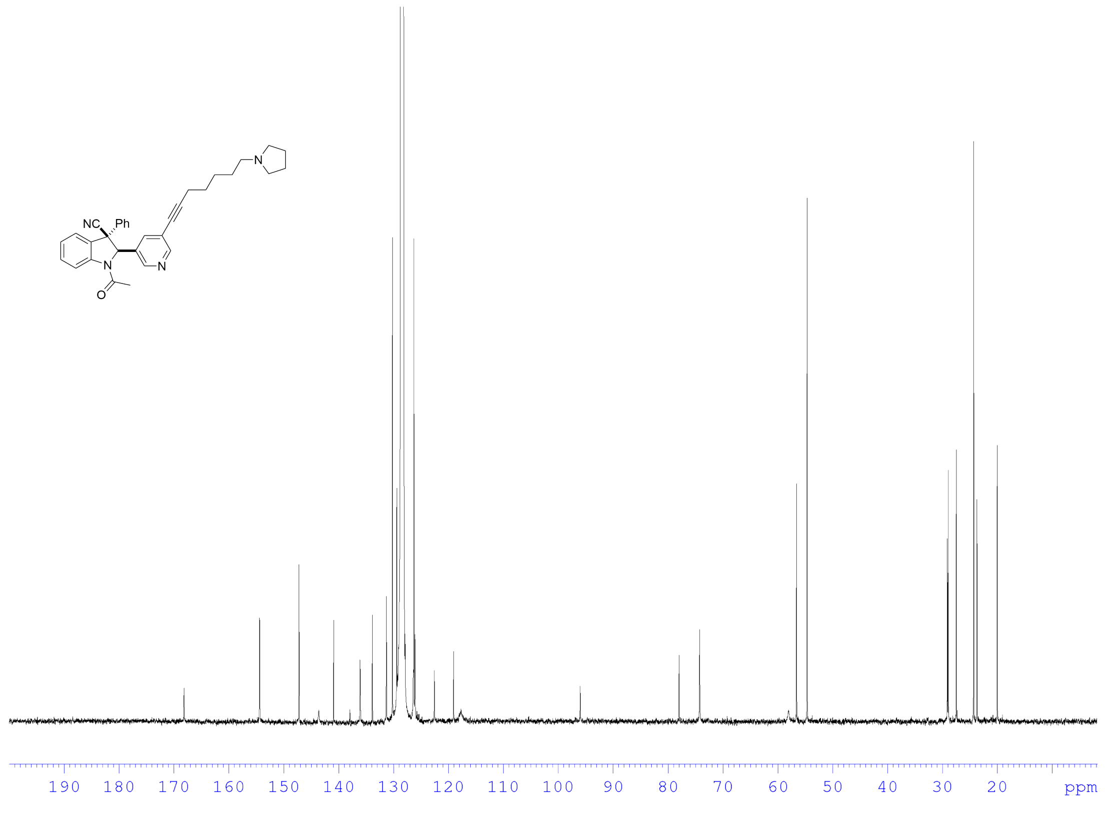
NC
Ph
N
N
O
190 180 170 160 150 140 130 120 110 100 90 80 70 60 50 40 30 20 ppm

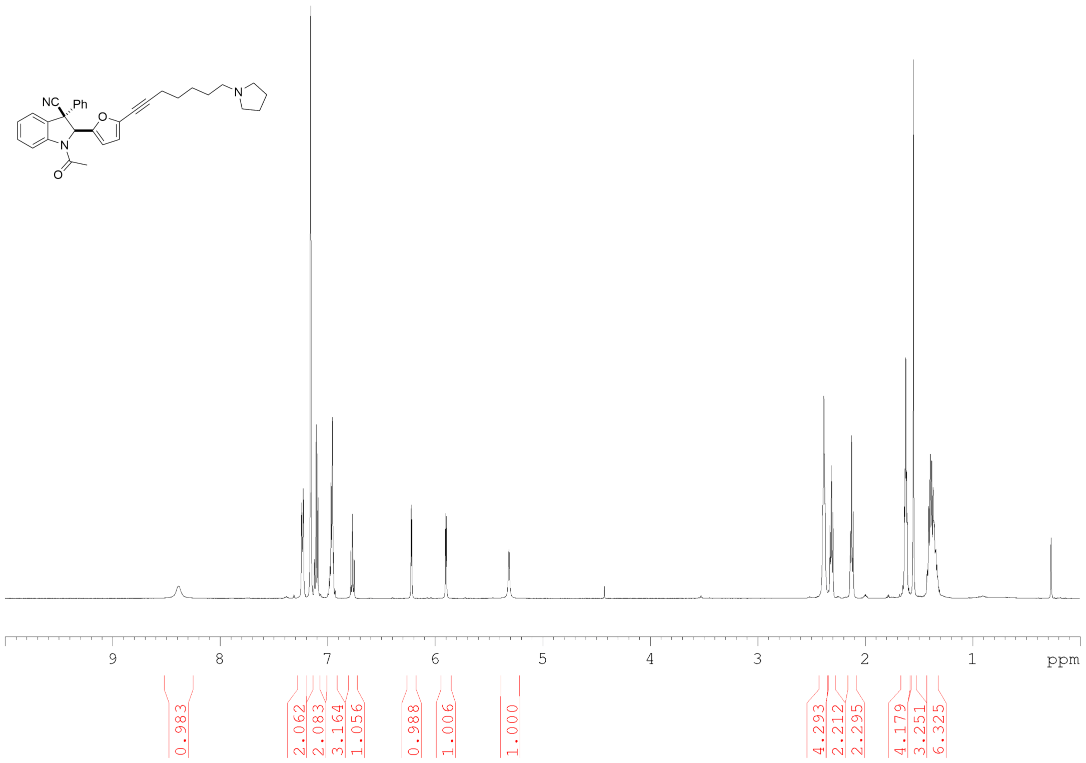
NC
Ph
O
N
N
O
0.983
2.062
2.083
3.164
1.056
0.988
1.006
1.000
4.293
2.212
2.295
4.179
3.251
6.325
9
8
7
6
5
4
3
2
1
ppm

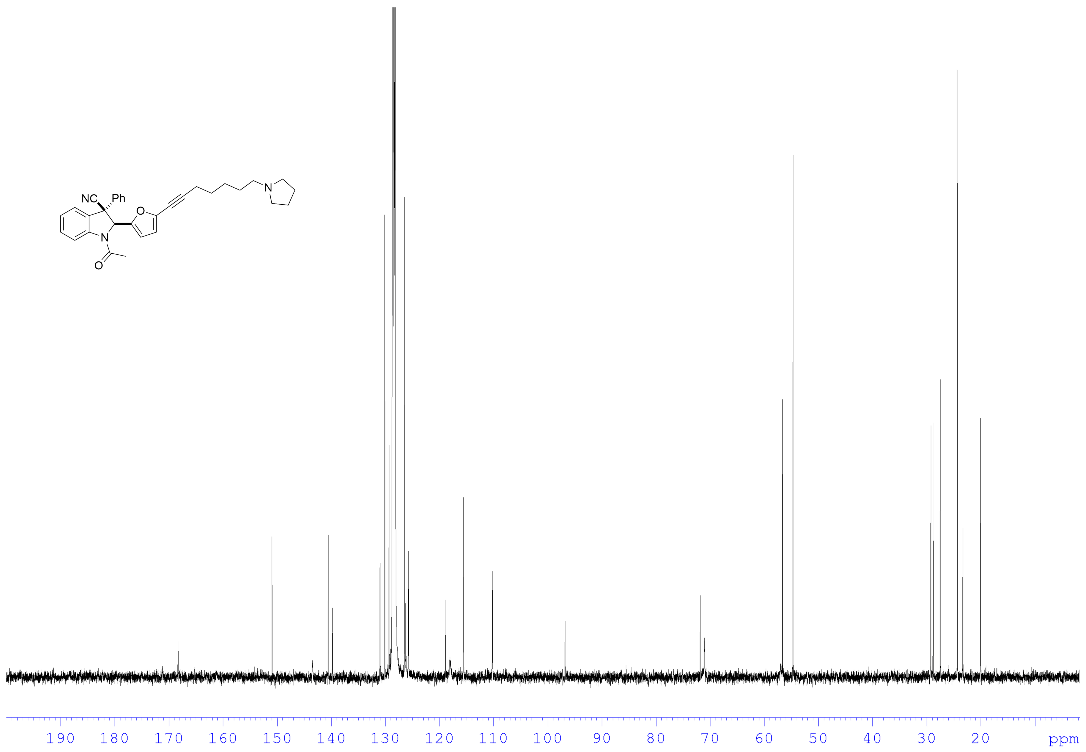
NC
Ph
O
N
N
O
190
180
170
160
150
140
130
120
110
100
90
80
70
60
50
40
30
20
ppm

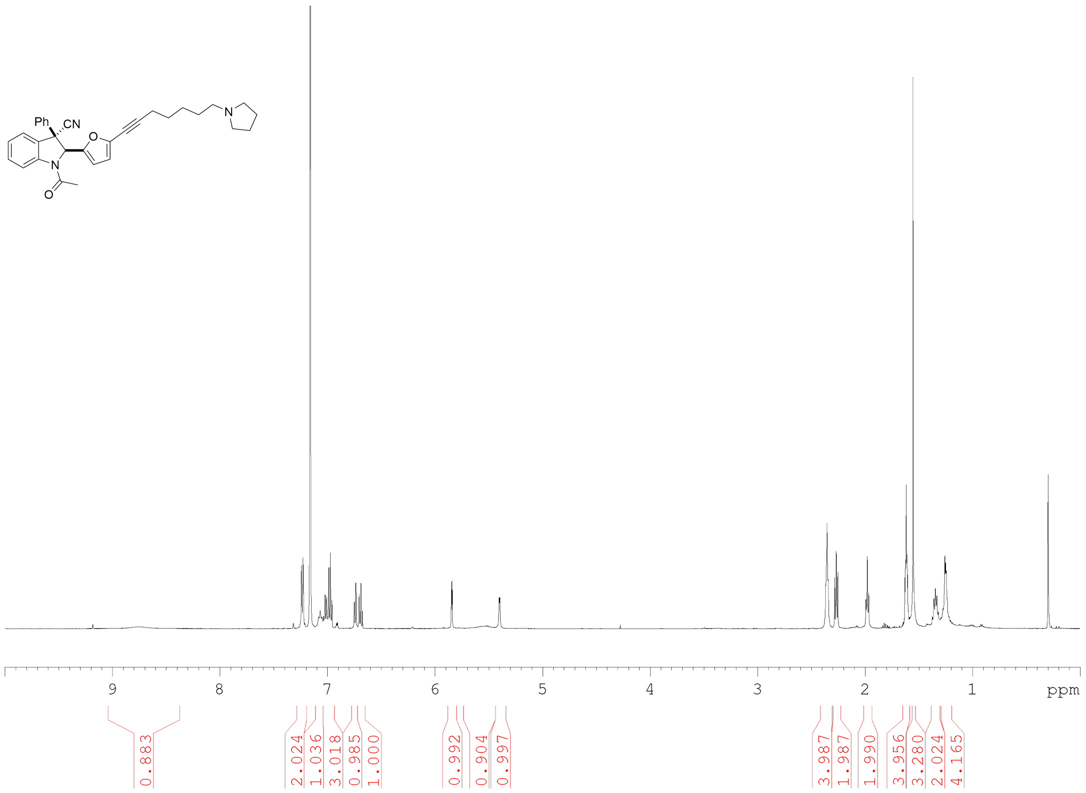
Ph
CN
N
O
0.883
2.024
1.036
3.018
0.985
1.000
0.992
0.904
0.997
3.987
1.987
1.990
3.956
3.280
2.024
4.165
ppm

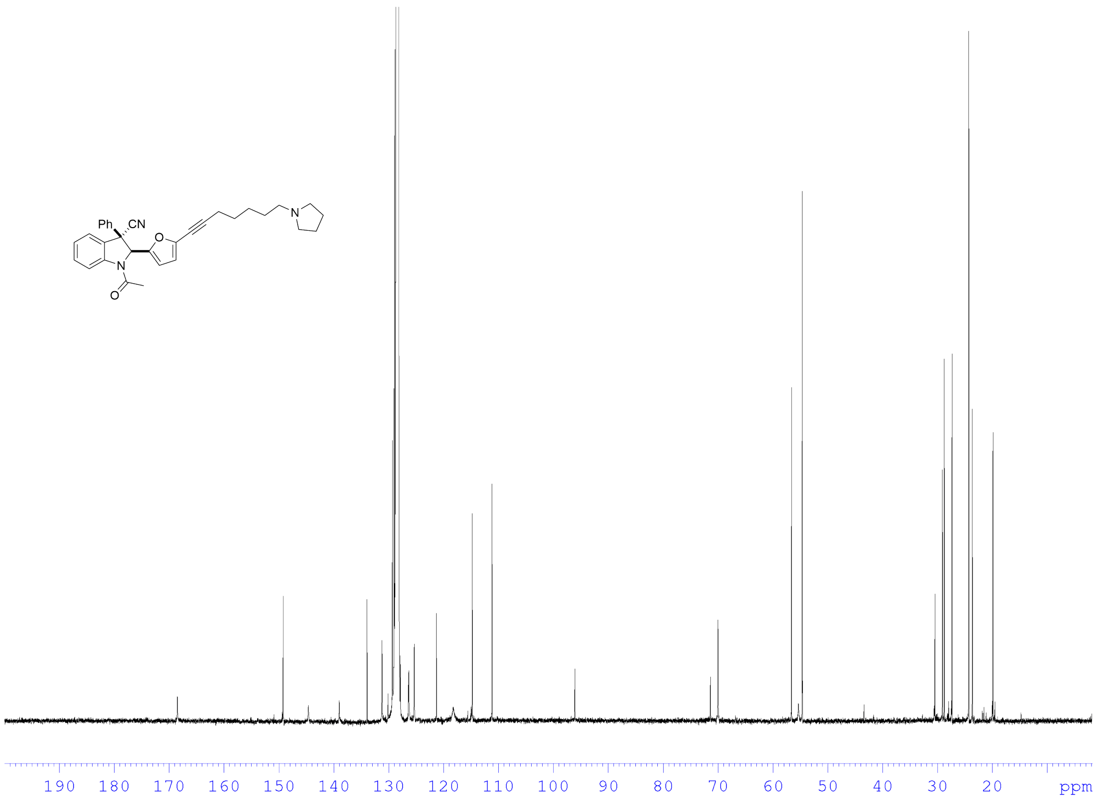
Ph
CN
N
O
ppm

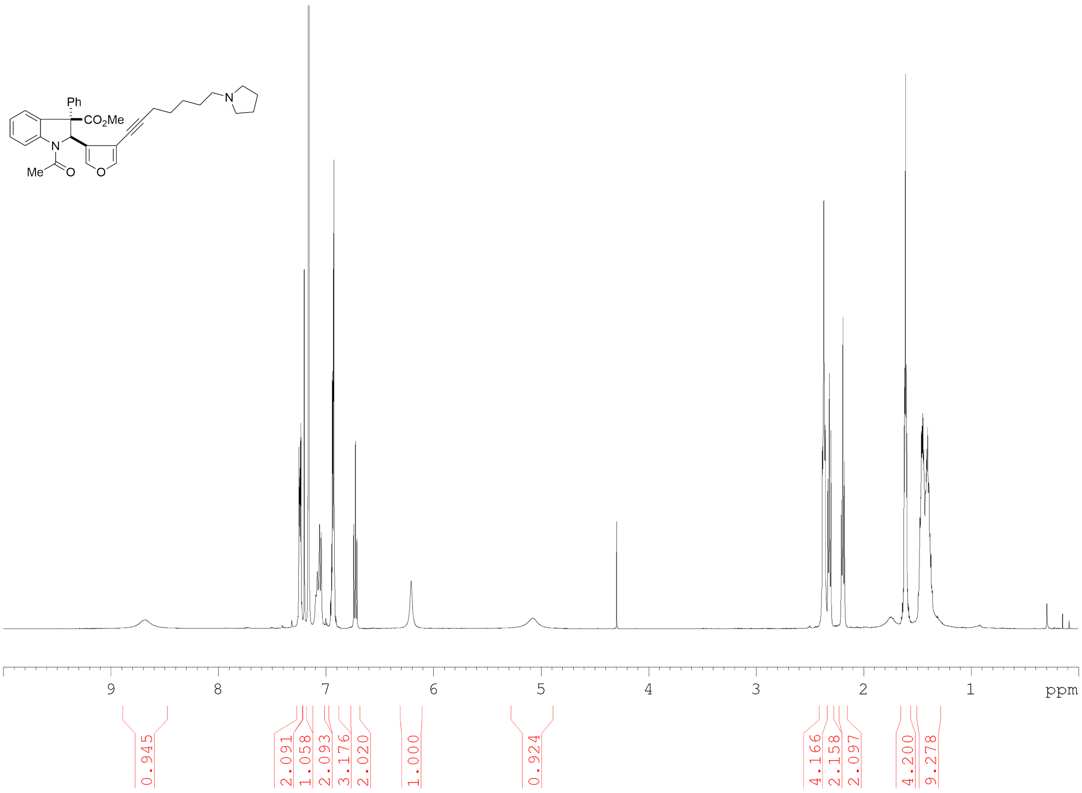
Ph
CO2Me
N
Me
O
O
N
9
8
7
6
5
4
3
2
1
ppm
0.945
2.091
1.058
2.093
3.176
2.020
1.000
0.924
4.166
2.158
2.097
4.200
9.278

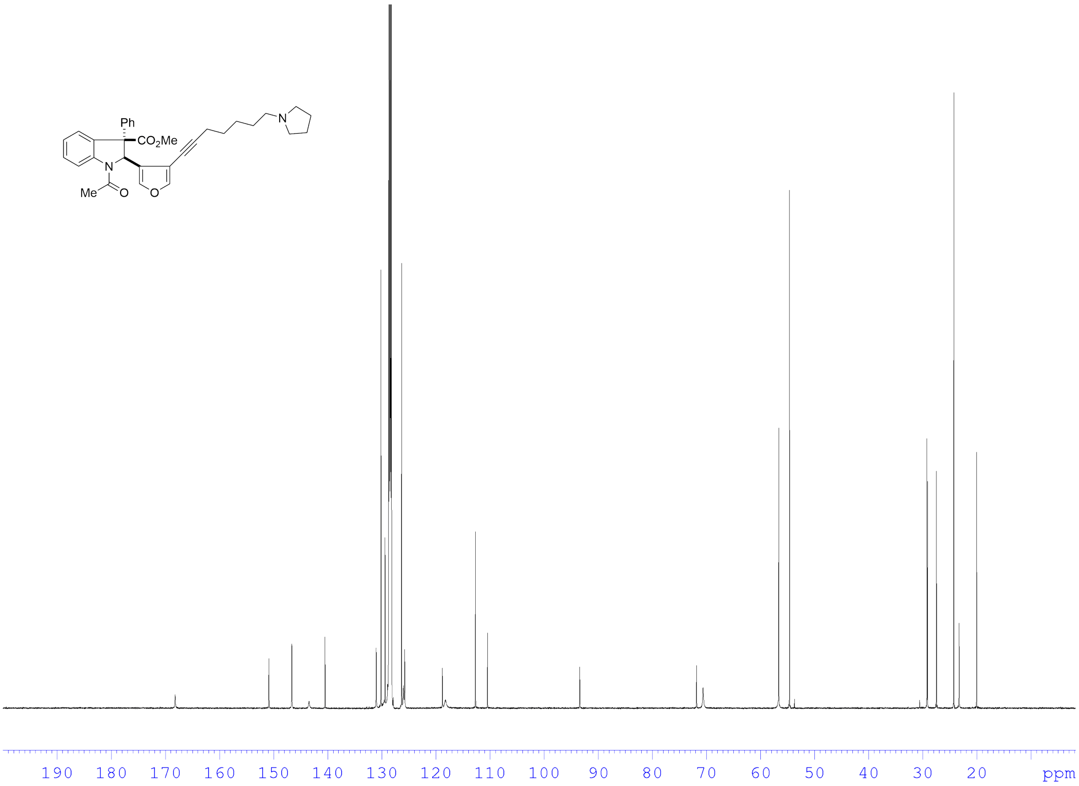
Ph
CO2Me
N
Me
O
O
N
190
180
170
160
150
140
130
120
110
100
90
80
70
60
50
40
30
20
ppm

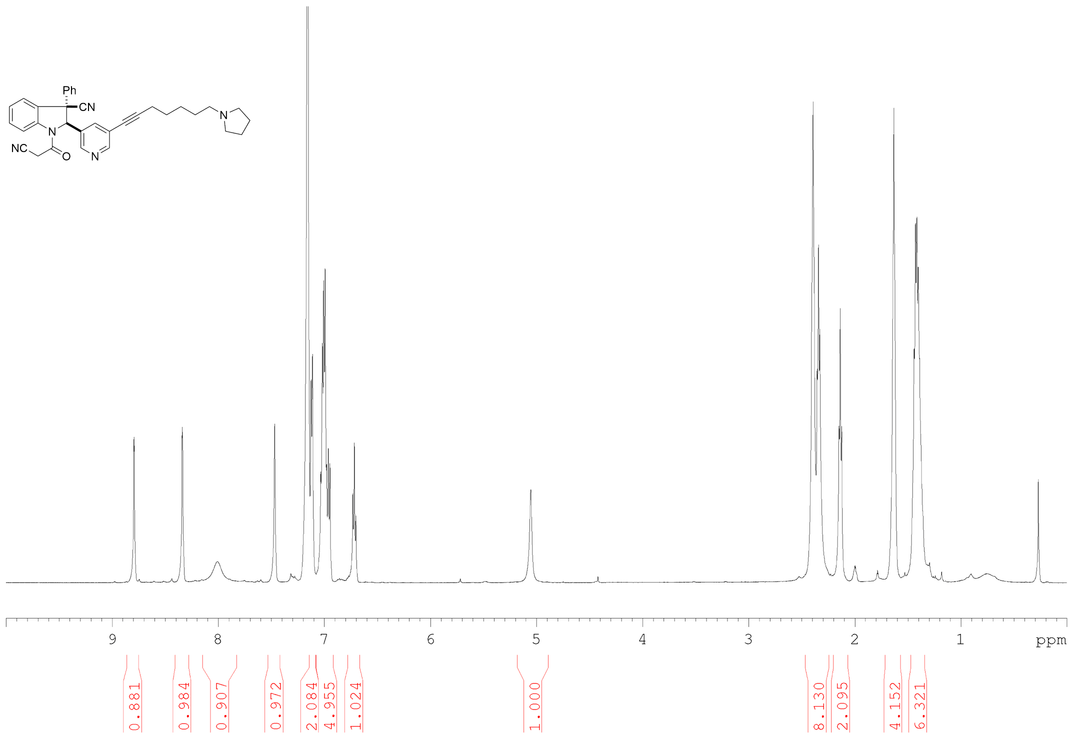
Ph
CN
N
NC
O
N
N
9
8
7
6
5
4
3
2
1
ppm
0.881
0.984
0.907
0.972
2.084
4.955
1.024
1.000
8.130
2.095
4.152
6.321

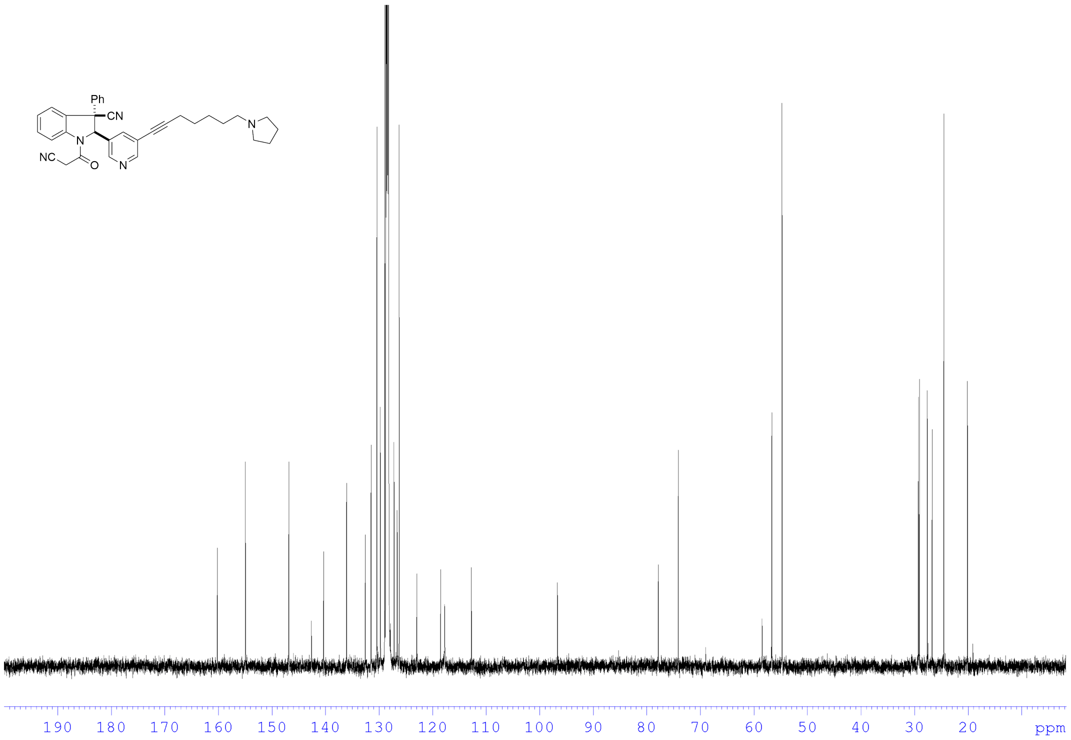
Ph
CN
N
NC
O
N
N
190
180
170
160
150
140
130
120
110
100
90
80
70
60
50
40
30
20
ppm

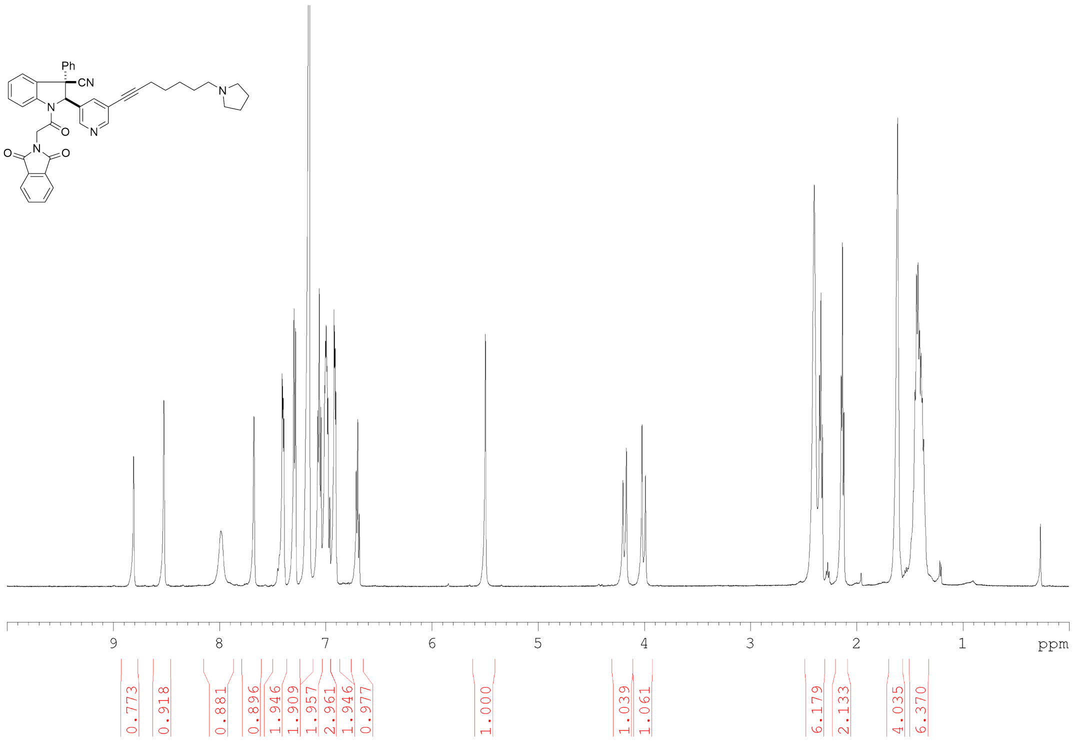

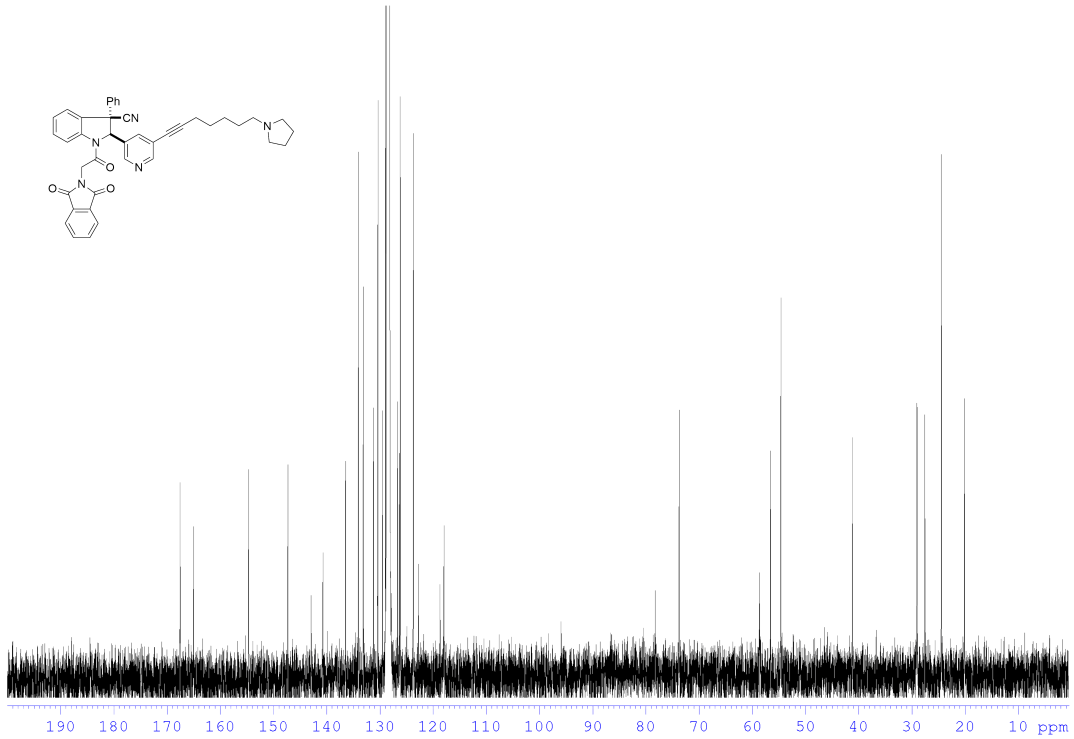

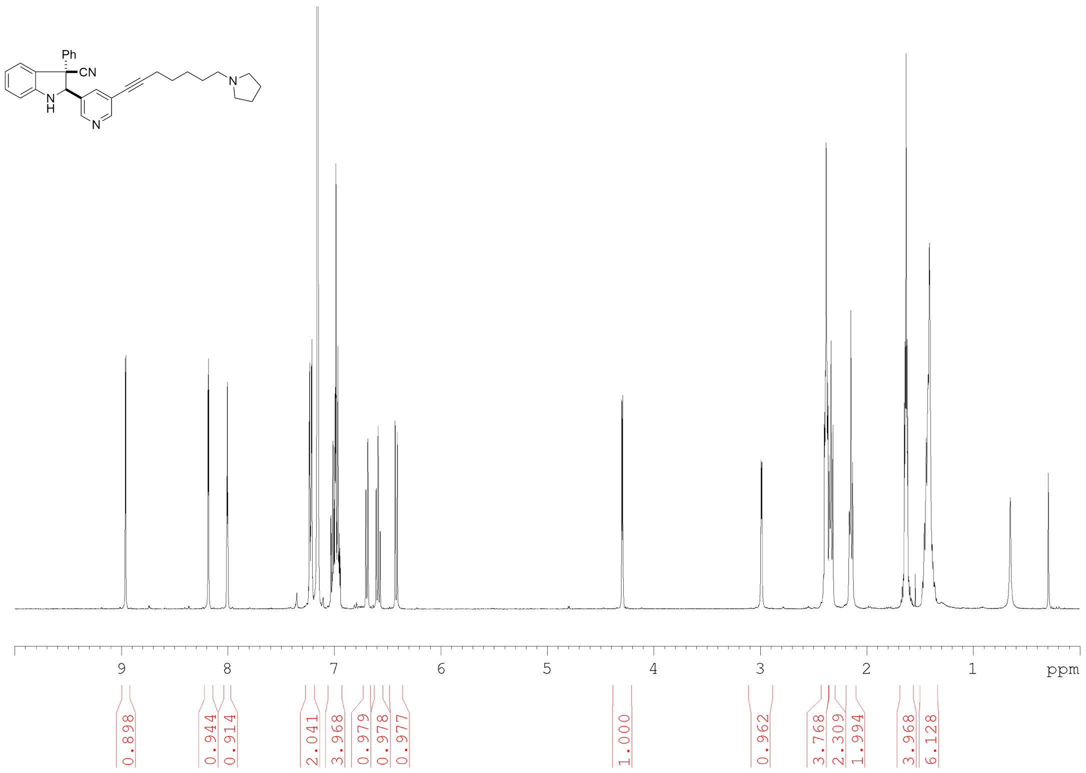
Ph
CN
N
H
N
N
9
8
7
6
5
4
3
2
1
ppm
0.898
0.944
0.914
2.041
3.968
0.979
0.978
0.977
1.000
0.962
3.768
2.309
1.994
3.968
6.128

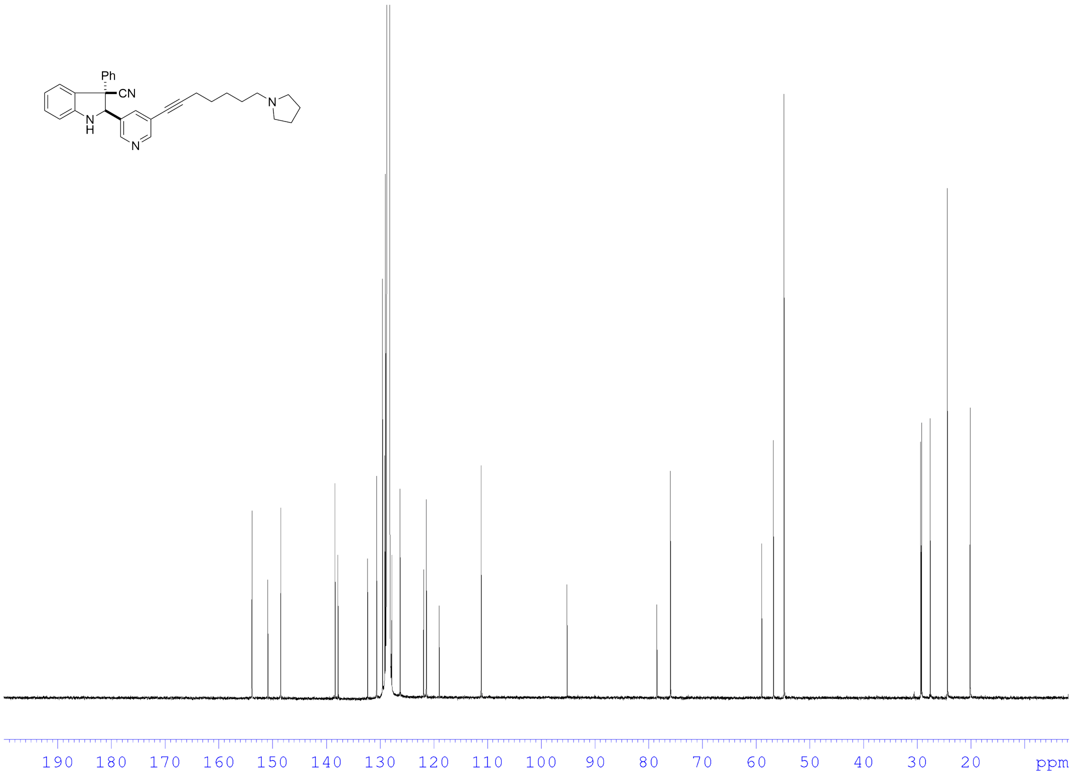
Ph
CN
N
H
N
N
190
180
170
160
150
140
130
120
110
100
90
80
70
60
50
40
30
20
ppm

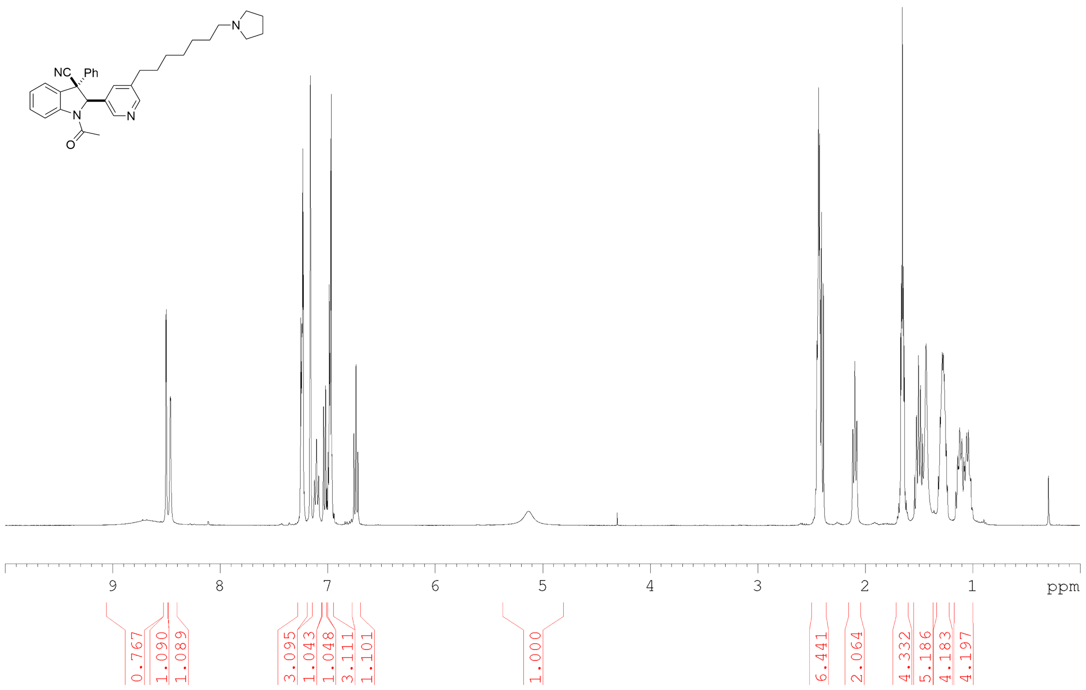
NC
Ph
N
N
O
N
9 8 7 6 5 4 3 2 1 ppm
0.767 1.090 1.089 3.095 1.043 1.048 3.111 1.101 1.000 6.441 2.064 4.332 5.186 4.183 4.197

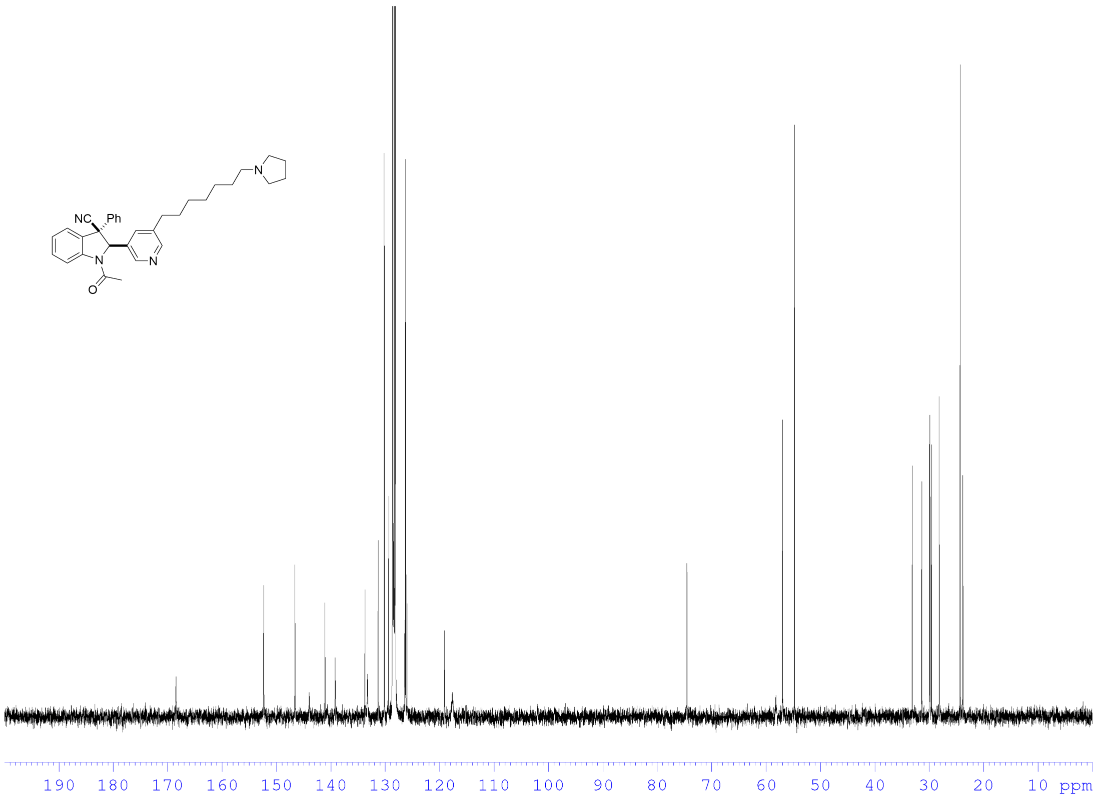
NC
Ph
N
N
O
N
190 180 170 160 150 140 130 120 110 100 90 80 70 60 50 40 30 20 10 ppm

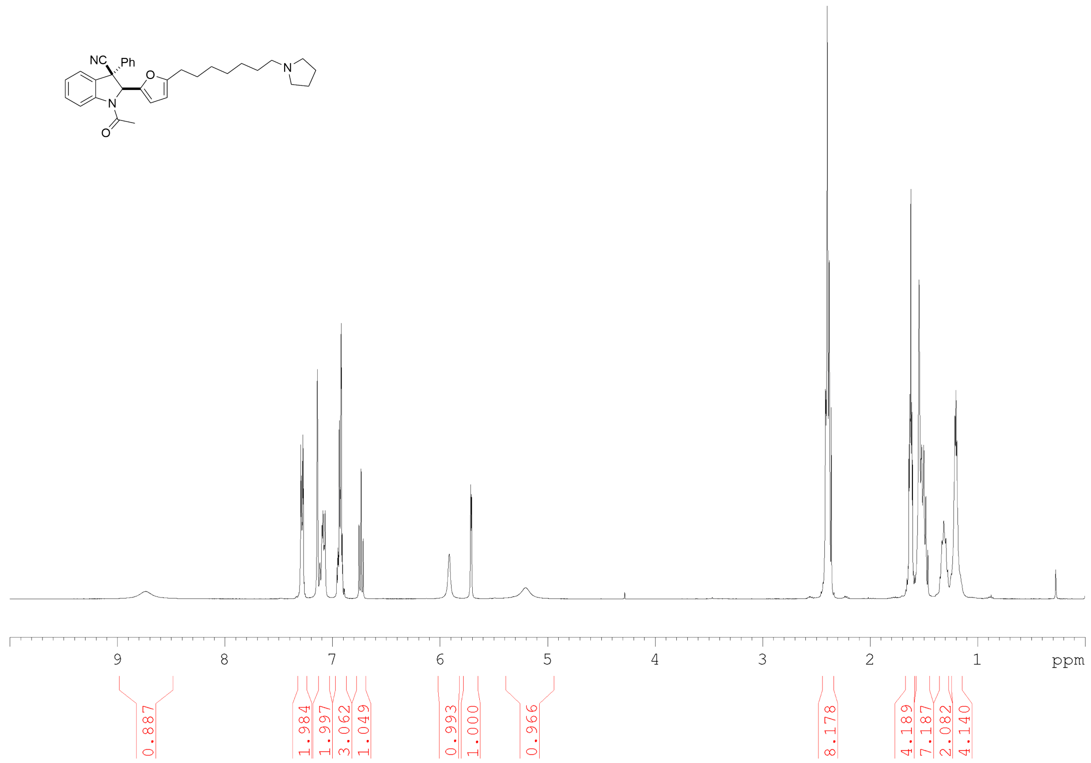
NC
Ph
O
N
N
O
9 8 7 6 5 4 3 2 1 ppm
0.887
1.984
1.997
3.062
1.049
0.993
1.000
0.966
8.178
4.189
7.187
2.082
4.140

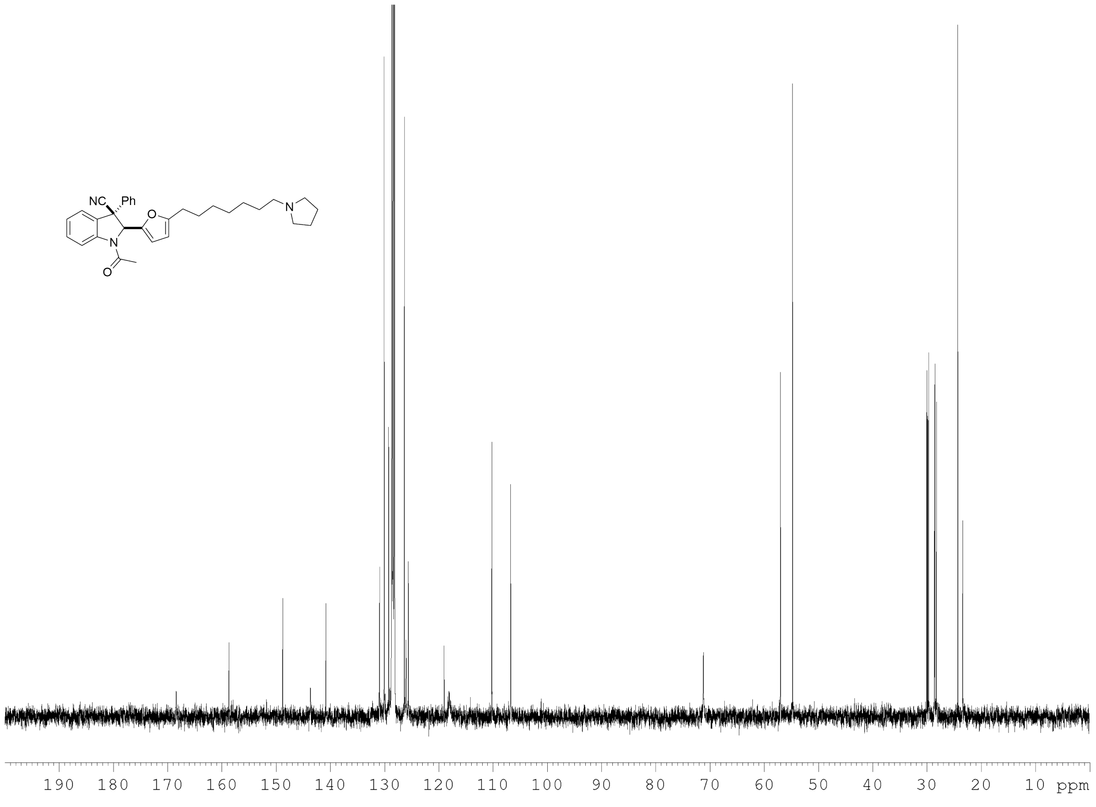
NC
Ph
O
N
N
O
190 180 170 160 150 140 130 120 110 100 90 80 70 60 50 40 30 20 10 ppm

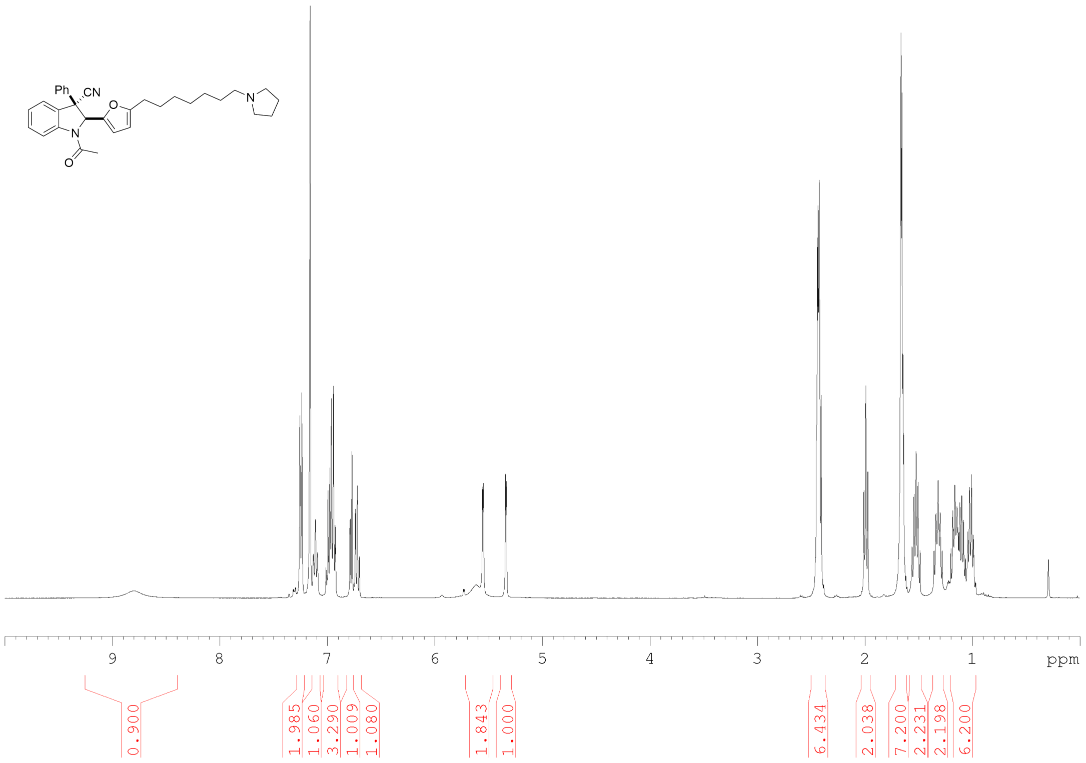

Ph
CN
O
N
N
O
9
8
7
6
5
4
3
2
1
ppm
0.900
1.985
1.060
3.290
1.009
1.080
1.843
1.000
6.434
2.038
7.200
2.231
2.198
6.200

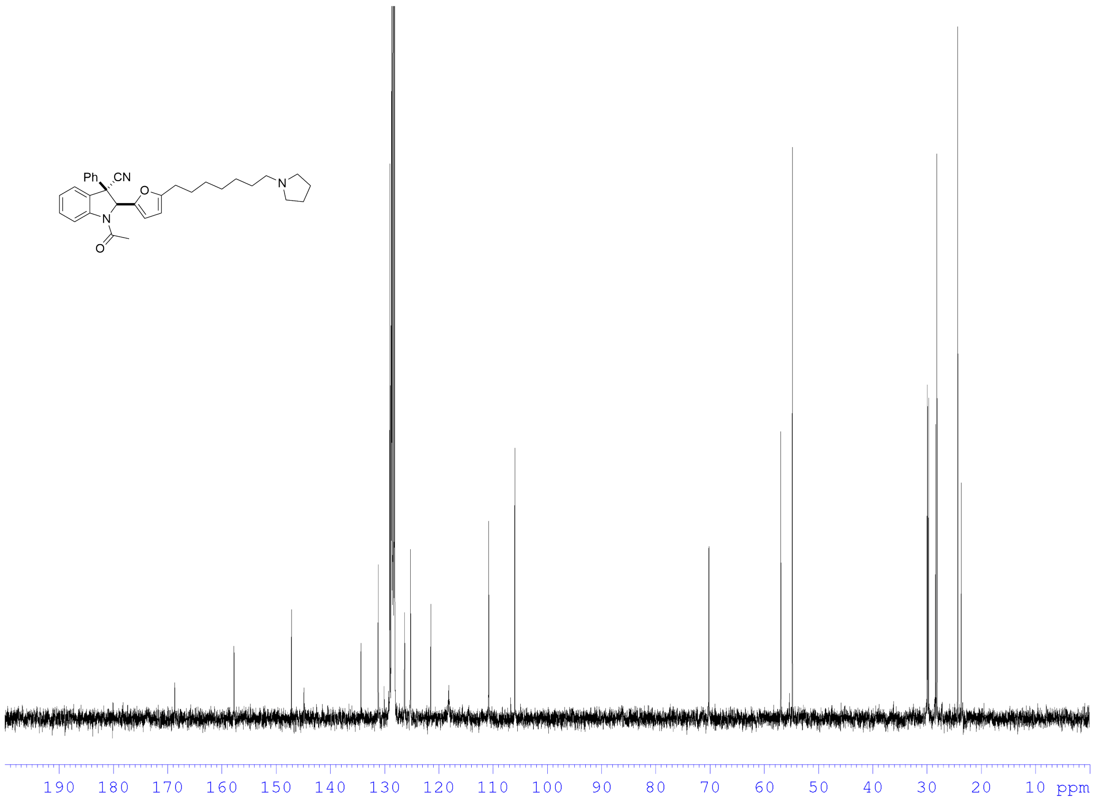

Ph
CN
O
N
N
O
190
180
170
160
150
140
130
120
110
100
90
80
70
60
50
40
30
20
10
ppm

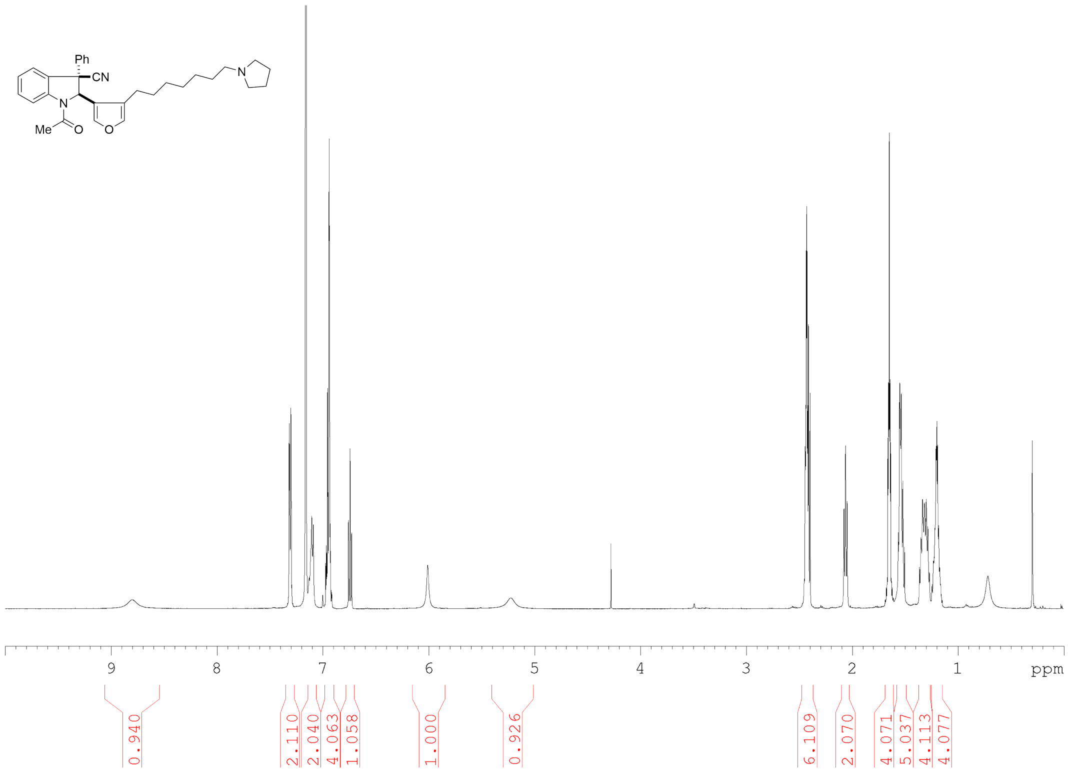

Ph
CN
N
Me
O
O
N
9 8 7 6 5 4 3 2 1 ppm
0.940
2.110
2.040
4.063
1.058
1.000
0.926
6.109
2.070
4.071
5.037
4.113
4.077

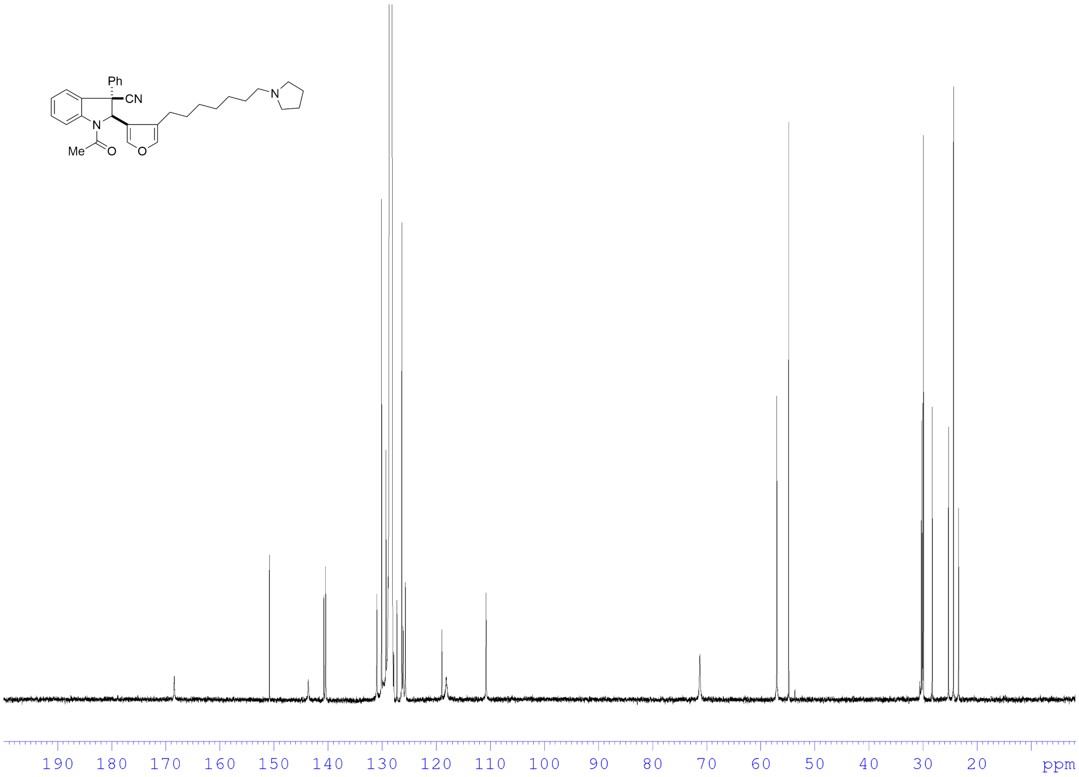

Ph
CN
N
Me
O
O
N
190 180 170 160 150 140 130 120 110 100 90 80 70 60 50 40 30 20 ppm

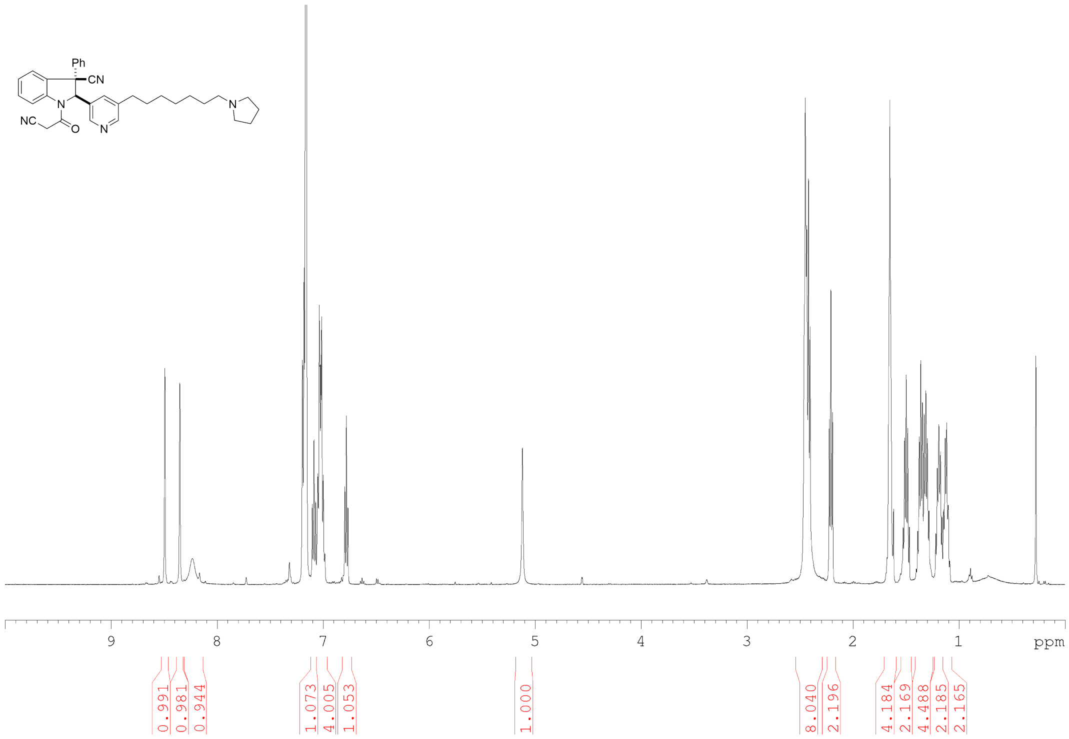
Ph
CN
N
NC
O
N
N
9 8 7 6 5 4 3 2 1 ppm
0.991 0.981 0.944 1.073 4.005 1.053 1.000 8.040 2.196 4.184 2.169 4.488 2.185 2.165

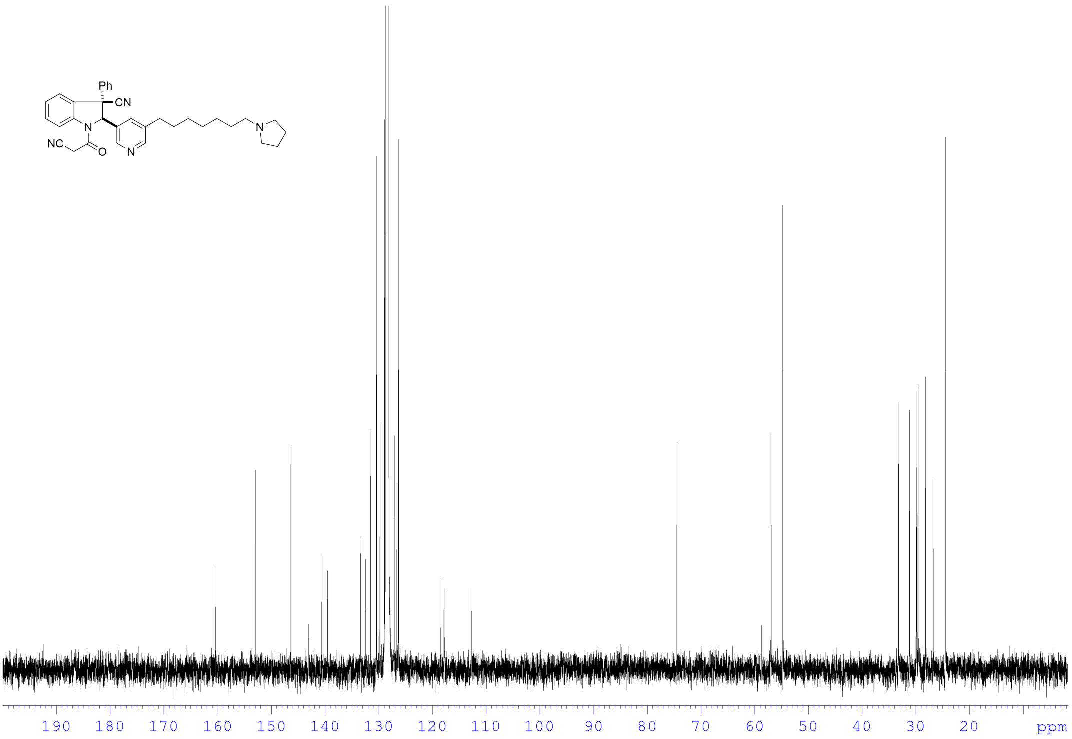
Ph
CN
N
NC
O
N
N
190 180 170 160 150 140 130 120 110 100 90 80 70 60 50 40 30 20 ppm

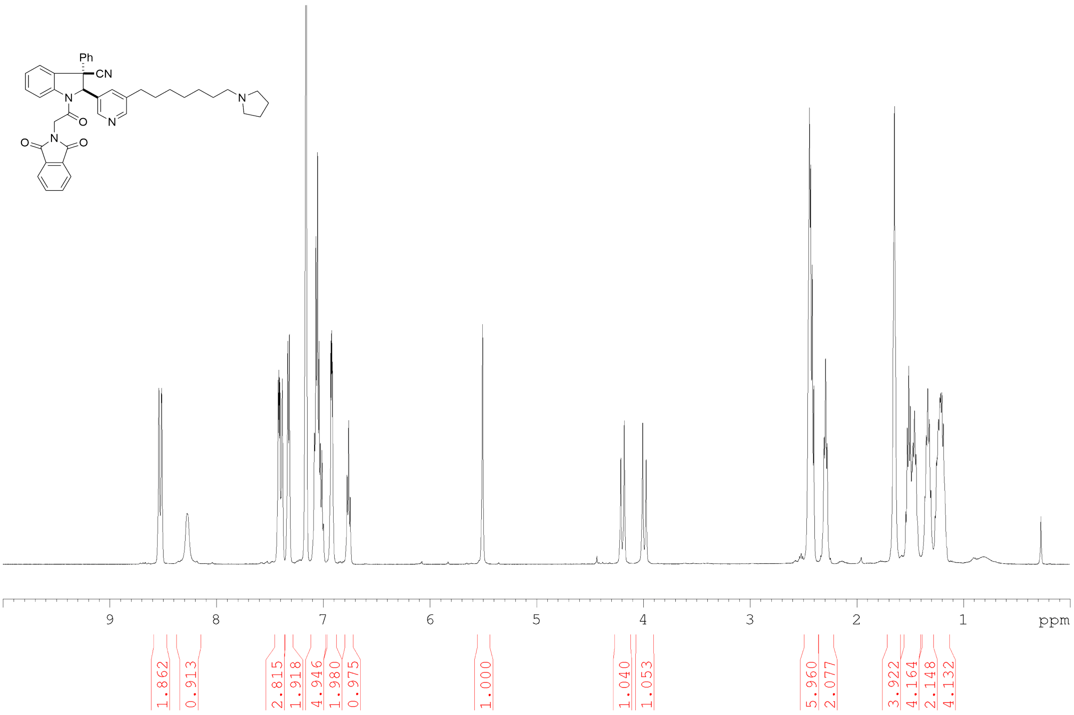
Ph
CN
N
O
N
O
O
N
N
9
8
7
6
5
4
3
2
1
ppm
1.862
0.913
2.815
1.918
4.946
1.980
0.975
1.000
1.040
1.053
5.960
2.077
3.922
4.164
2.148
4.132

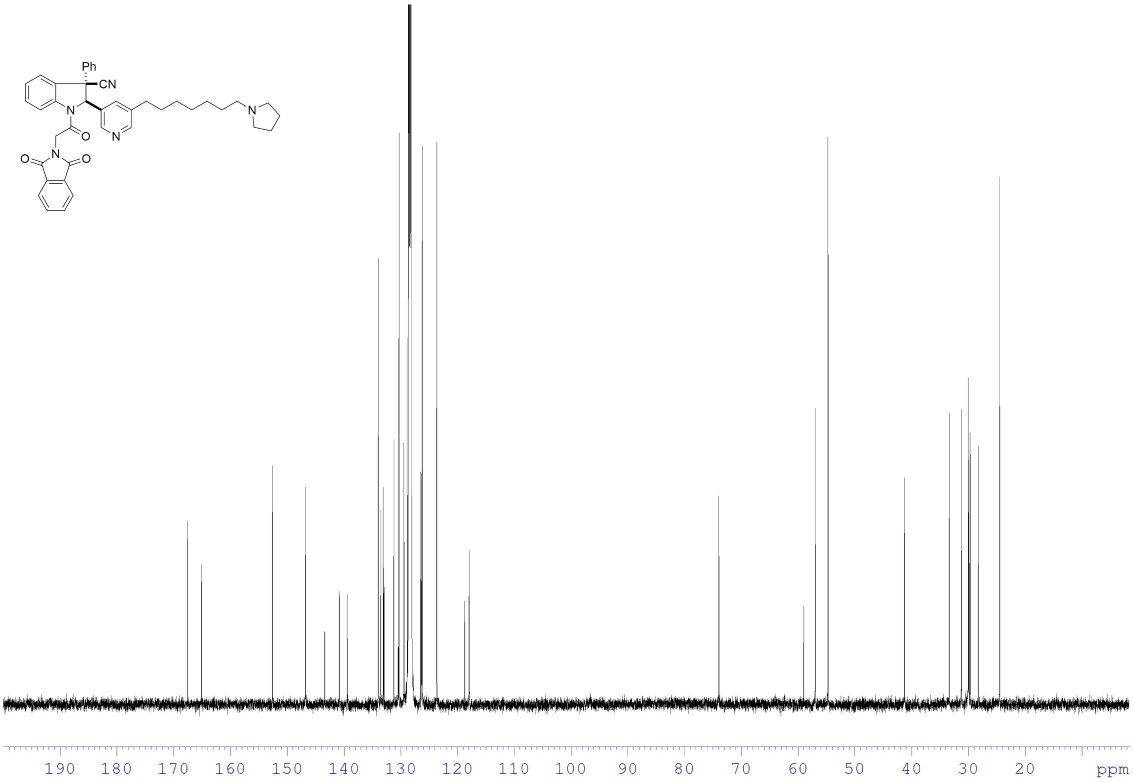
Ph
CN
N
O
N
O
O
N
N
190
180
170
160
150
140
130
120
110
100
90
80
70
60
50
40
30
20
ppm

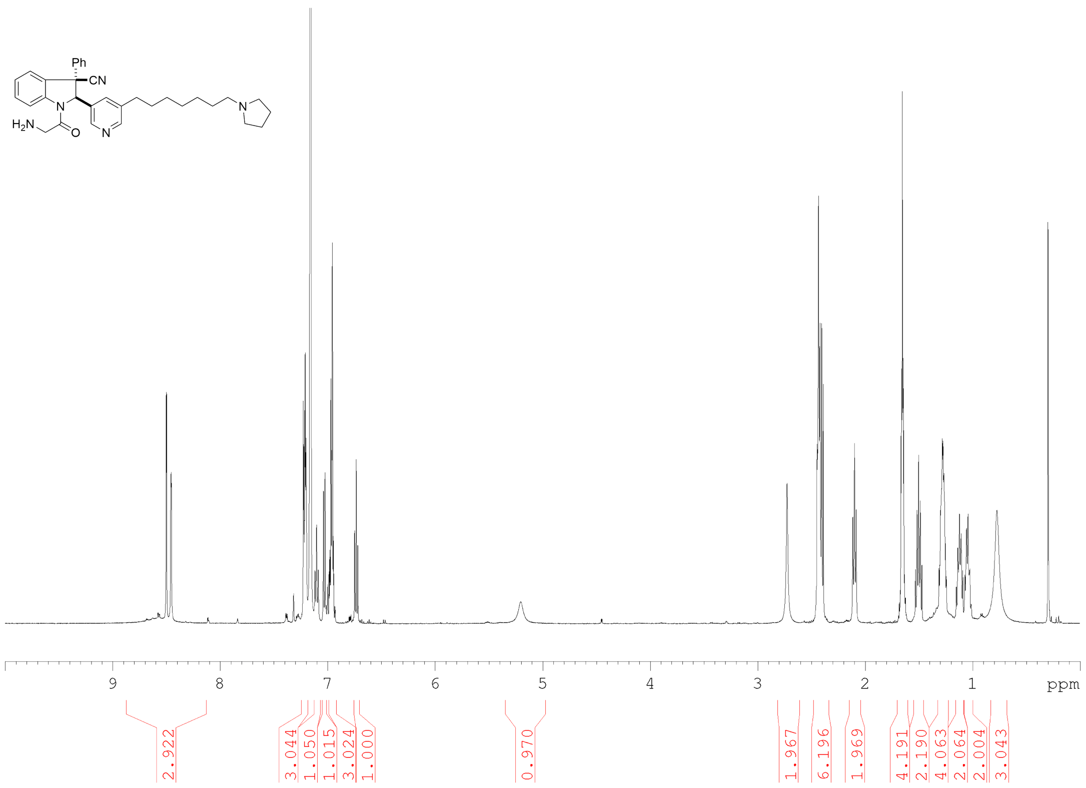
Ph
CN
N
N
N
H2N
O
9 8 7 6 5 4 3 2 1 ppm
2.922
3.044 1.050 1.015 3.024 1.000
0.970
1.967 6.196 1.969
4.191 2.190 4.063 2.064 2.004 3.043

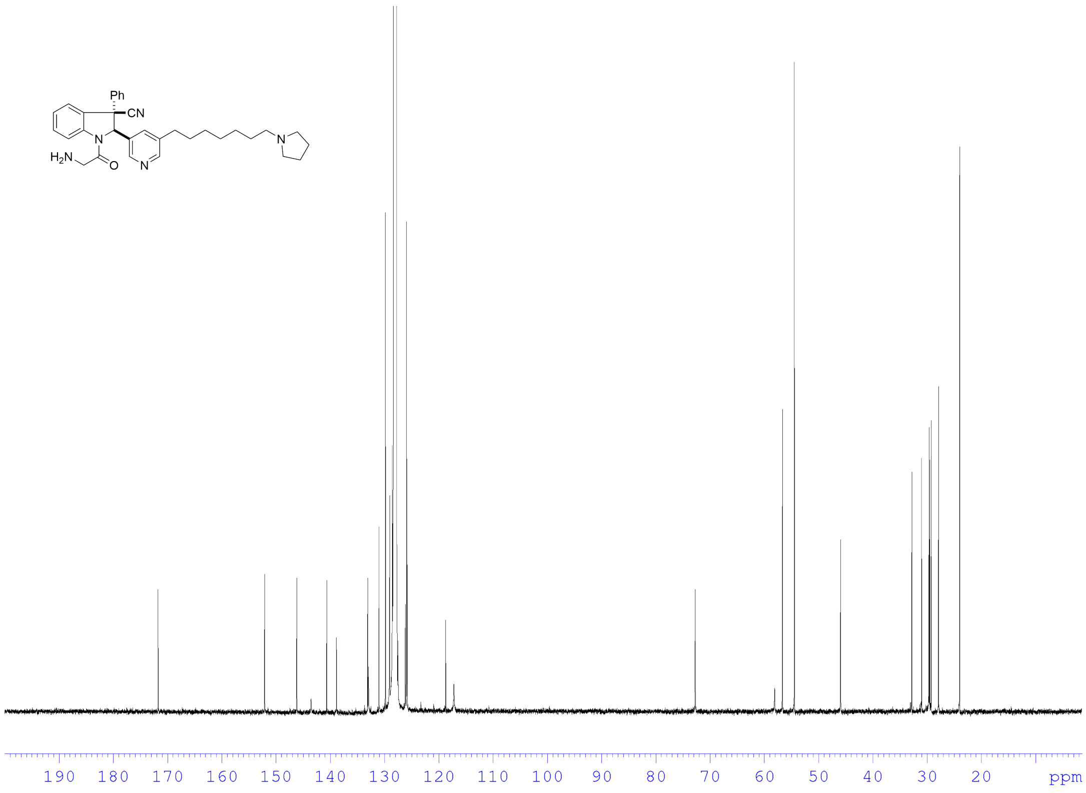
Ph
CN
N
N
N
H2N
O
190 180 170 160 150 140 130 120 110 100 90 80 70 60 50 40 30 20 ppm

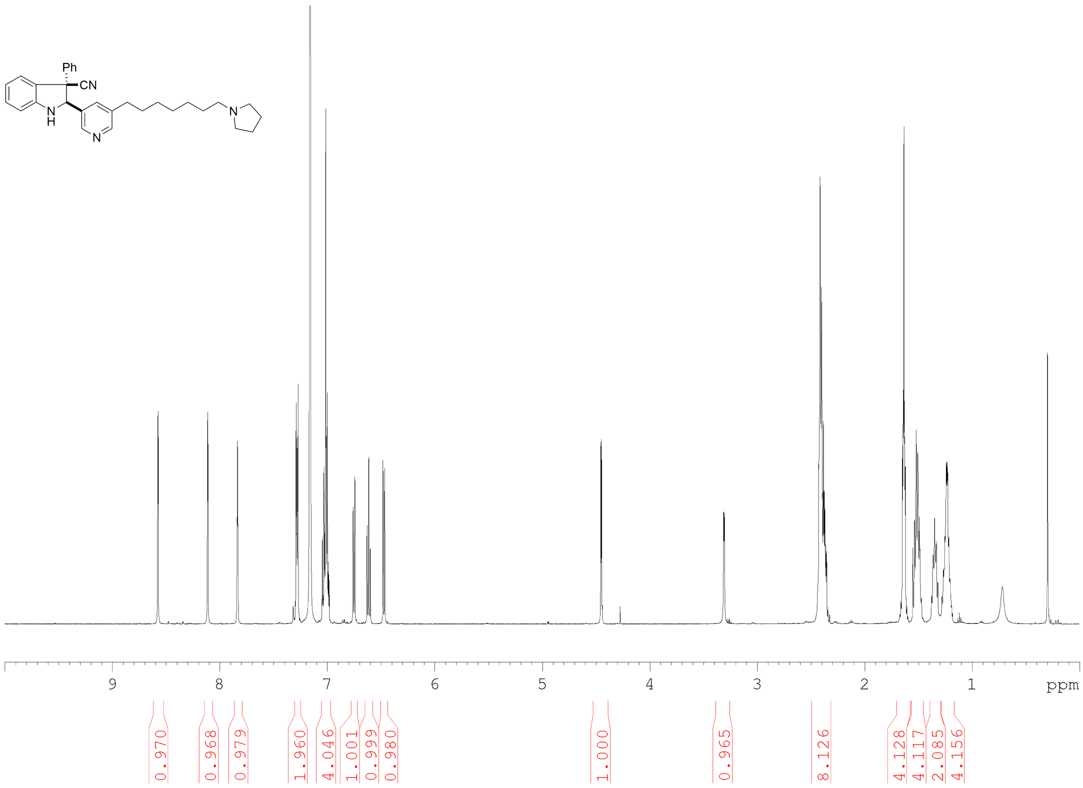
Ph
CN
N
H
N
N
9
8
7
6
5
4
3
2
1
ppm
0.970
0.968
0.979
1.960
4.046
1.001
0.999
0.980
1.000
0.965
8.126
4.128
4.117
2.085
4.156

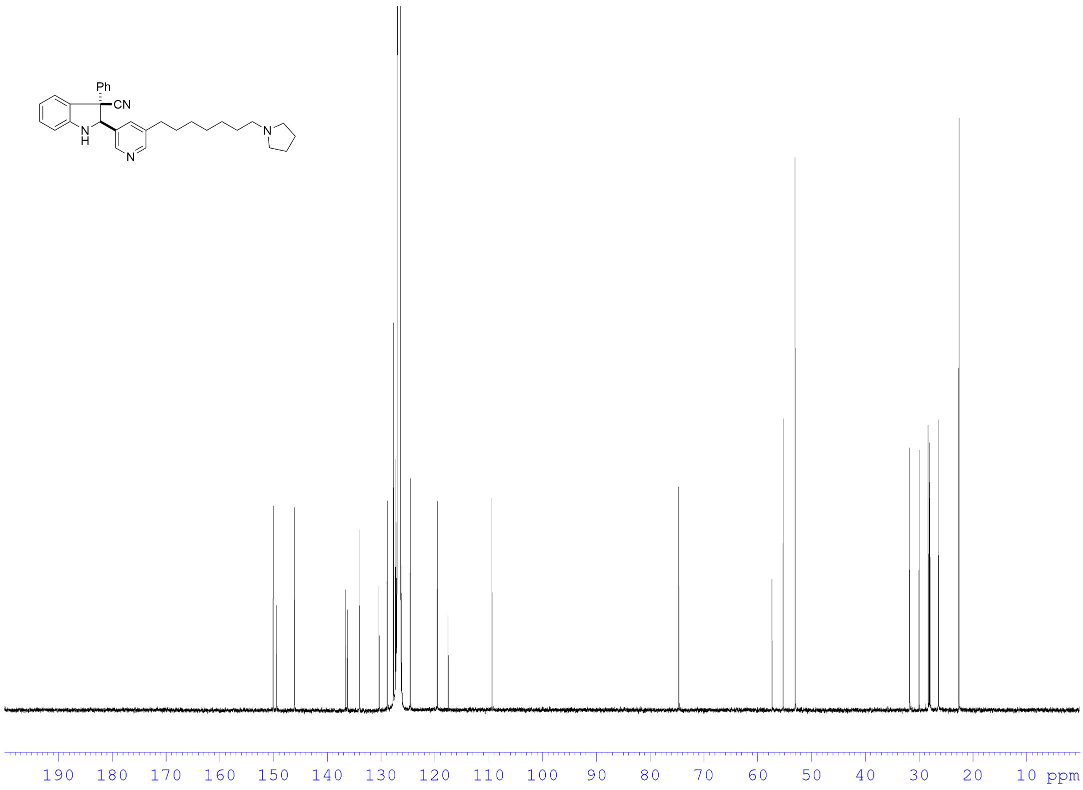
Ph
CN
N
H
N
N
190
180
170
160
150
140
130
120
110
100
90
80
70
60
50
40
30
20
10
ppm

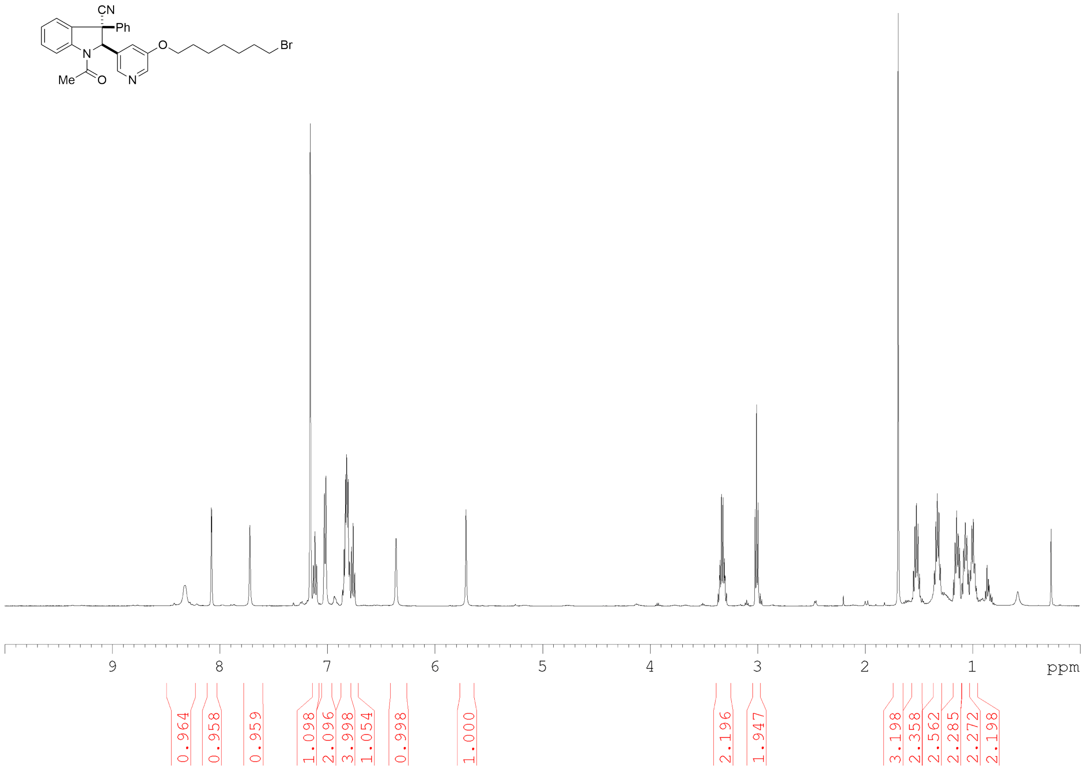
CN
Ph
O
Br
N
Me
O
N
9
8
7
6
5
4
3
2
1
ppm
0.964
0.958
0.959
1.098
2.096
3.998
1.054
0.998
1.000
2.196
1.947
3.198
2.358
2.562
2.285
2.272
2.198

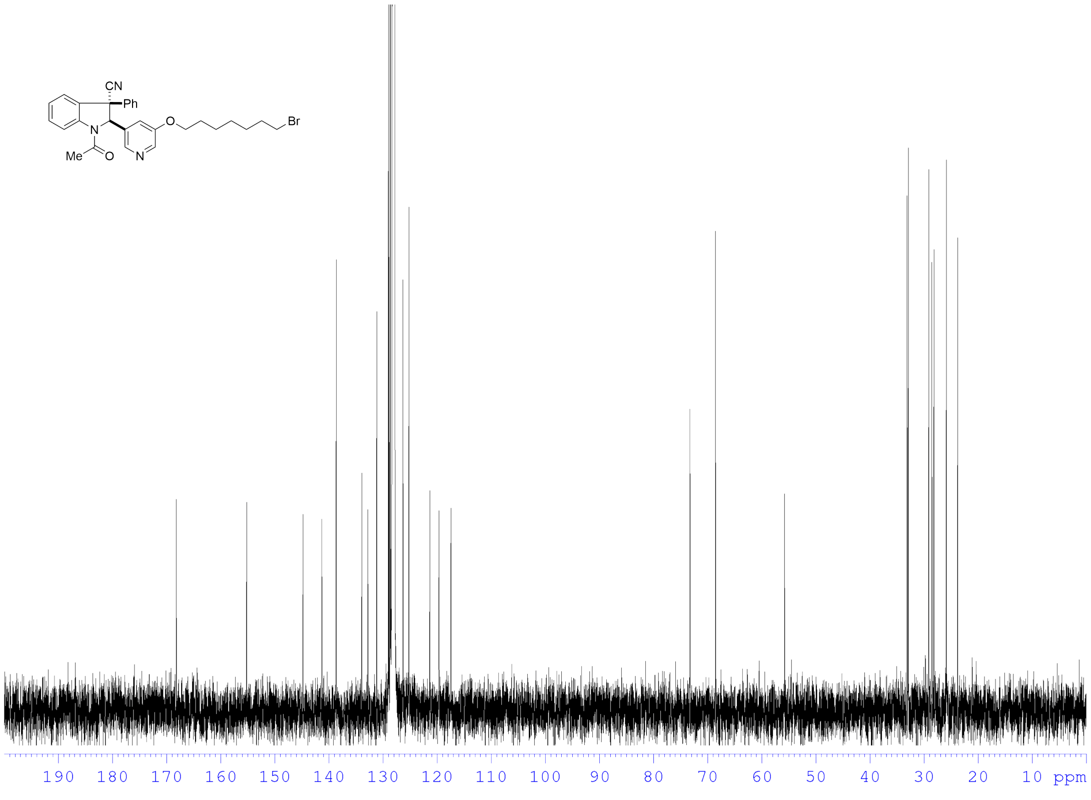
CN
Ph
O
Br
N
Me
O
N
190
180
170
160
150
140
130
120
110
100
90
80
70
60
50
40
30
20
10
ppm

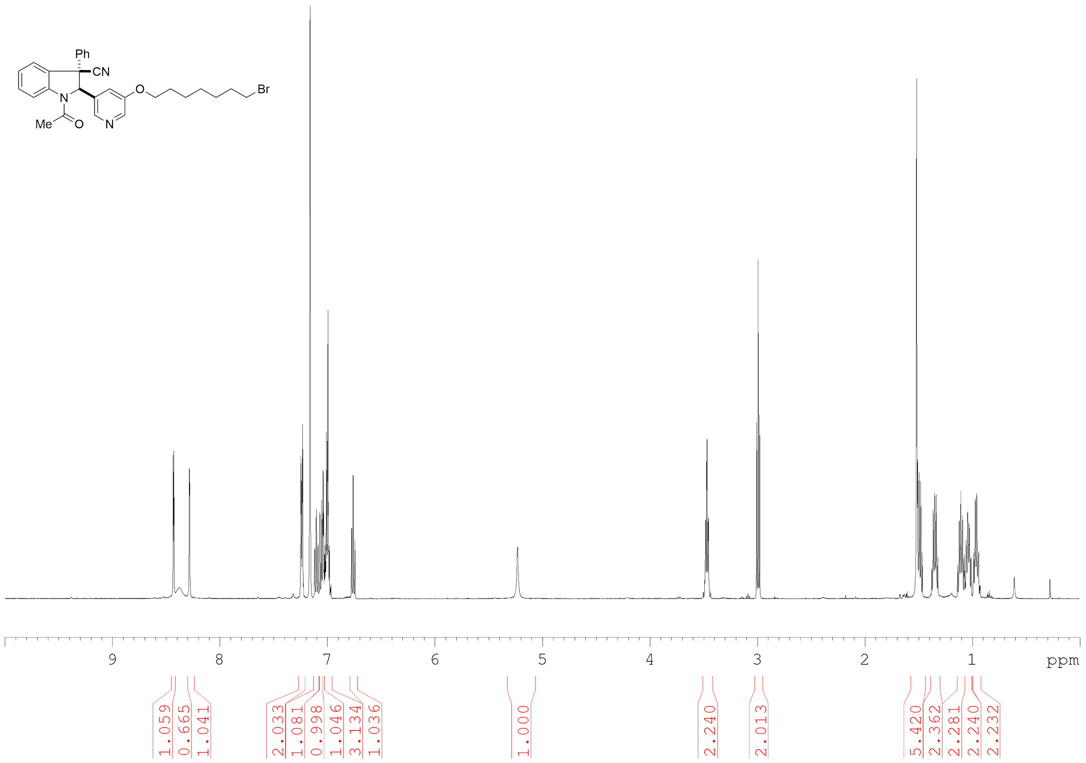
Ph
CN
O
Br
N
Me
O
N
9 8 7 6 5 4 3 2 1 ppm
1.059
0.665
1.041
2.033
1.081
0.998
1.046
3.134
1.036
1.000
2.240
2.013
5.420
2.362
2.281
2.240
2.232

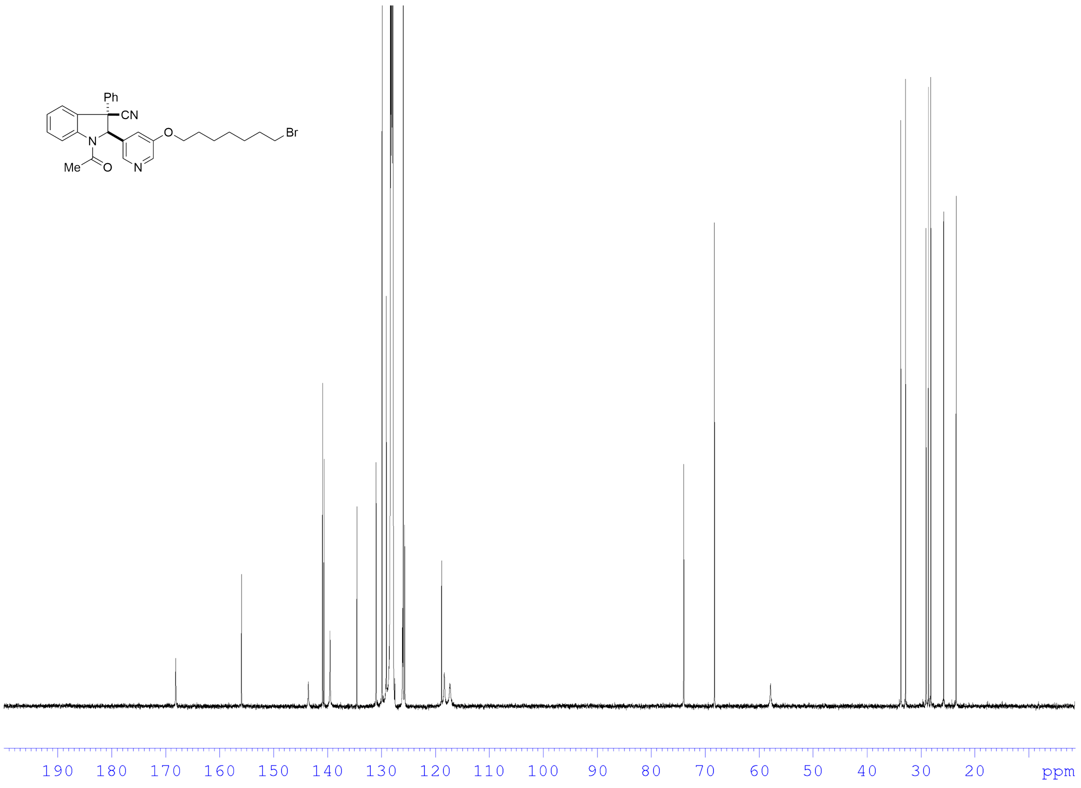
Ph
CN
O
Br
N
Me
O
N
190 180 170 160 150 140 130 120 110 100 90 80 70 60 50 40 30 20 ppm

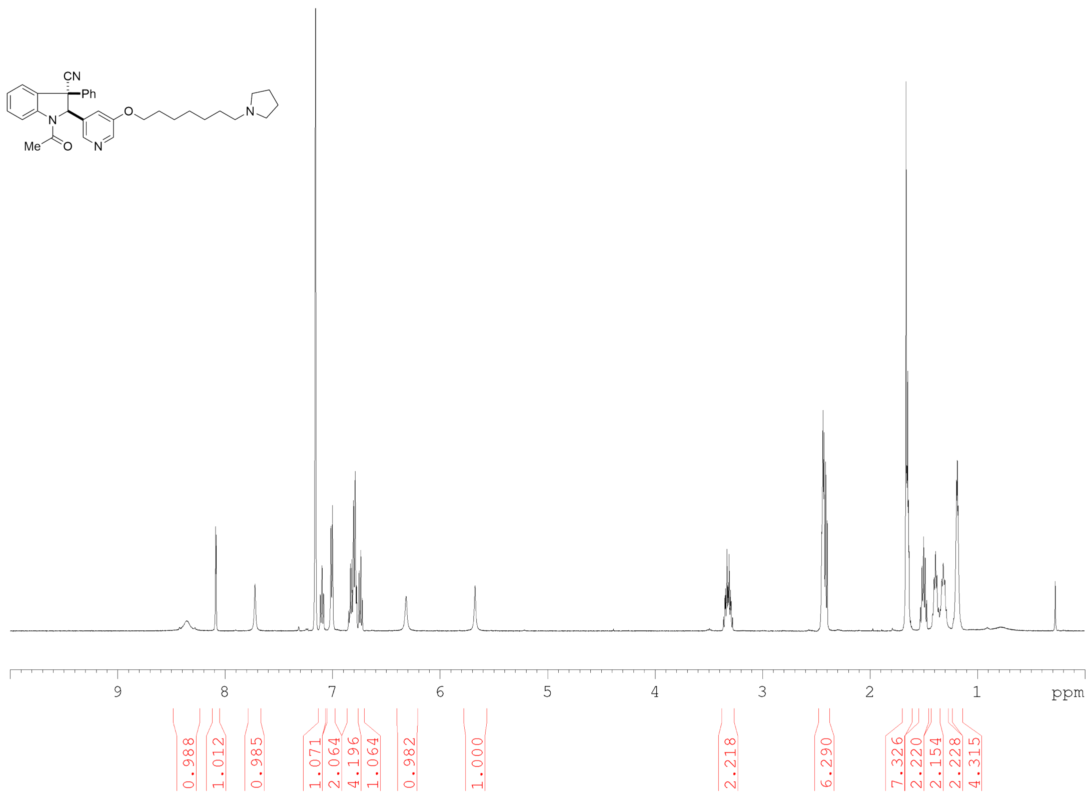
CN
Ph
N
O
N
N
Me
O
9
8
7
6
5
4
3
2
1
ppm
0.988
1.012
0.985
1.071
2.064
4.196
1.064
0.982
1.000
2.218
6.290
7.326
2.220
2.154
2.228
4.315

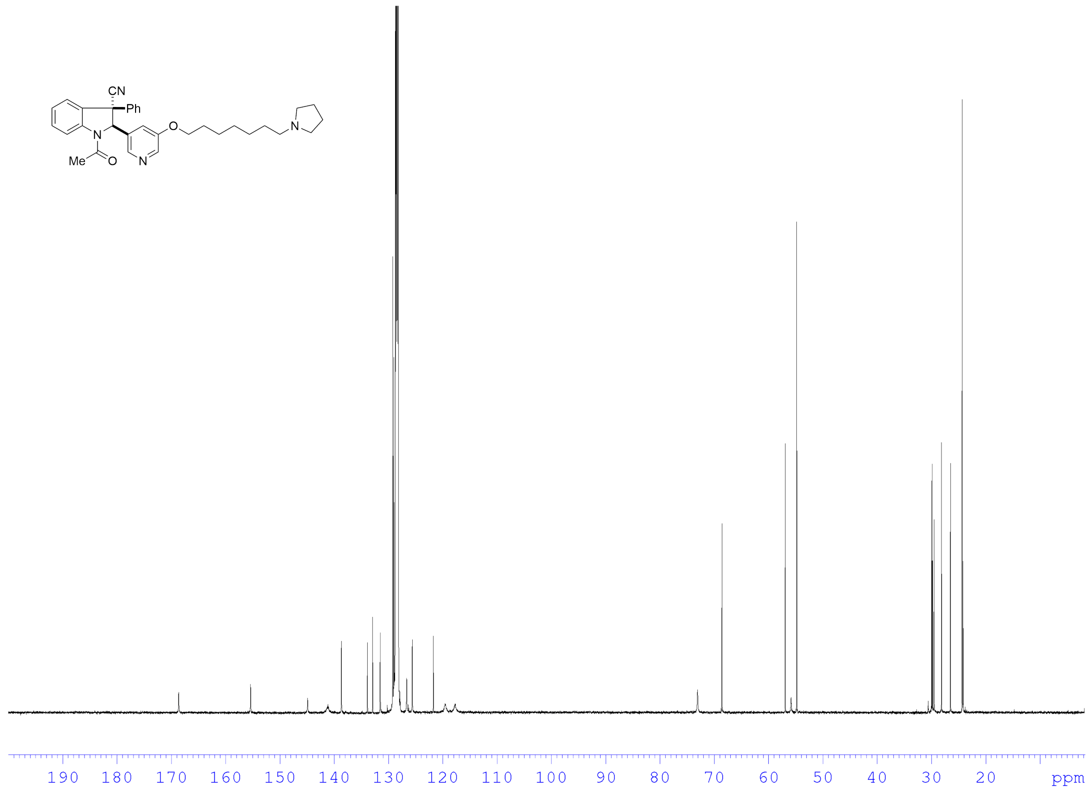
CN
Ph
N
O
N
N
Me
O
190
180
170
160
150
140
130
120
110
100
90
80
70
60
50
40
30
20
ppm

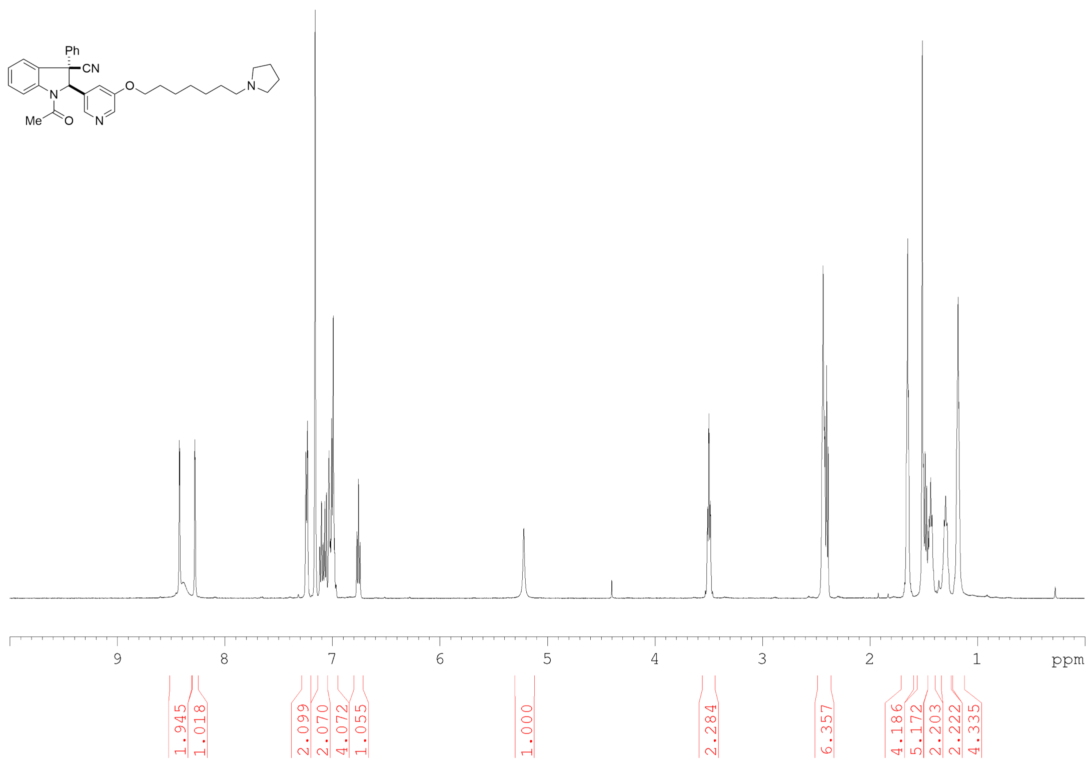
Ph
CN
N
Me
O
O
N
N
9
8
7
6
5
4
3
2
1
ppm
1.945
1.018
2.099
2.070
4.072
1.055
1.000
2.284
6.357
4.186
5.172
2.203
2.222
4.335

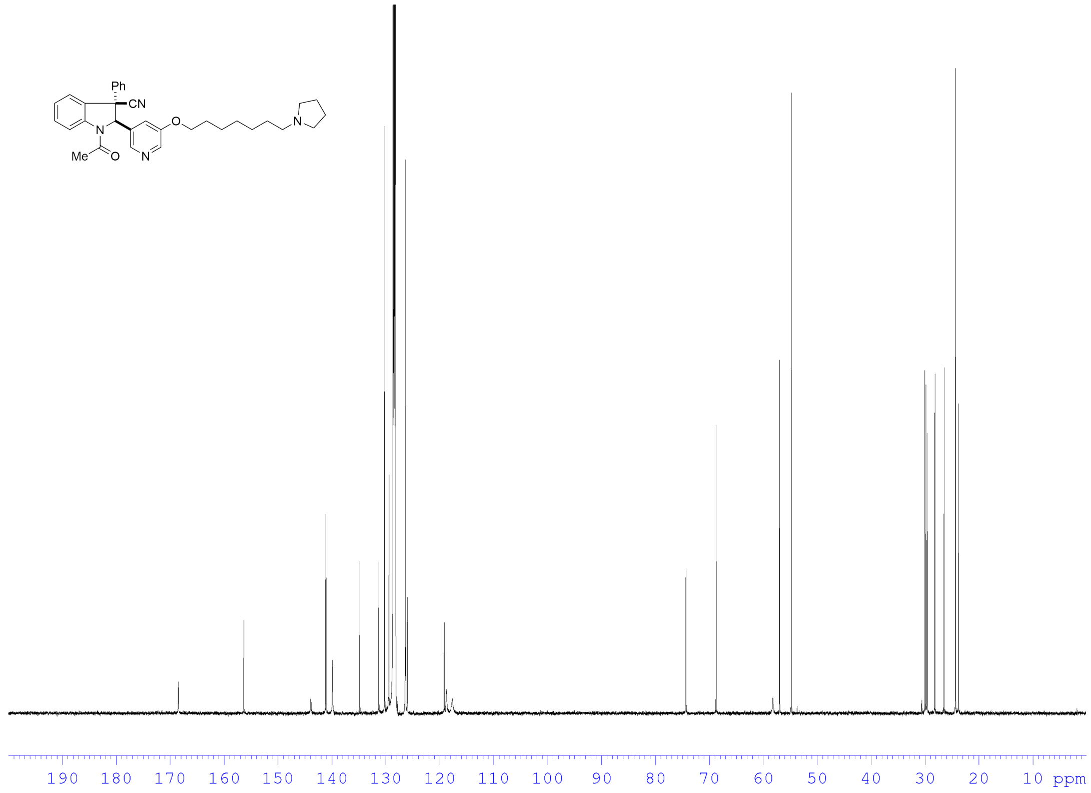
Ph
CN
N
Me
O
O
N
N
190
180
170
160
150
140
130
120
110
100
90
80
70
60
50
40
30
20
10
ppm

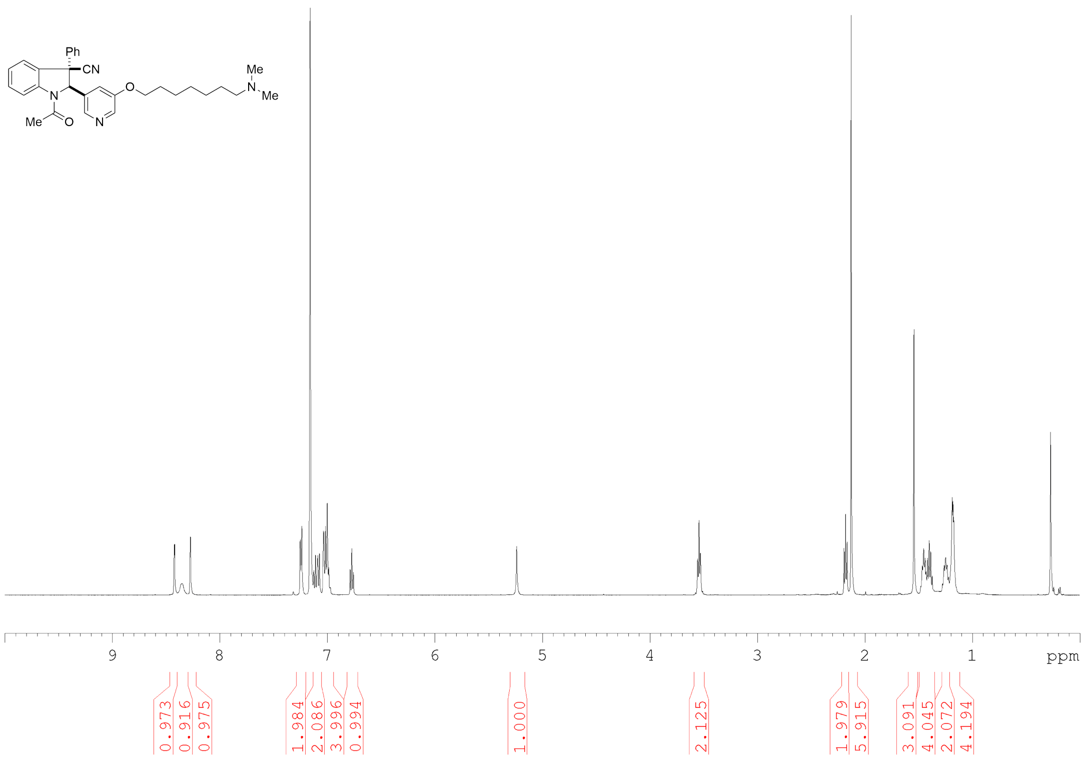
Ph
CN
O
N
Me
Me
N
Me
O
N
9
8
7
6
5
4
3
2
1
ppm
0.973
0.916
0.975
1.984
2.086
3.996
0.994
1.000
2.125
1.979
5.915
3.091
4.045
2.072
4.194

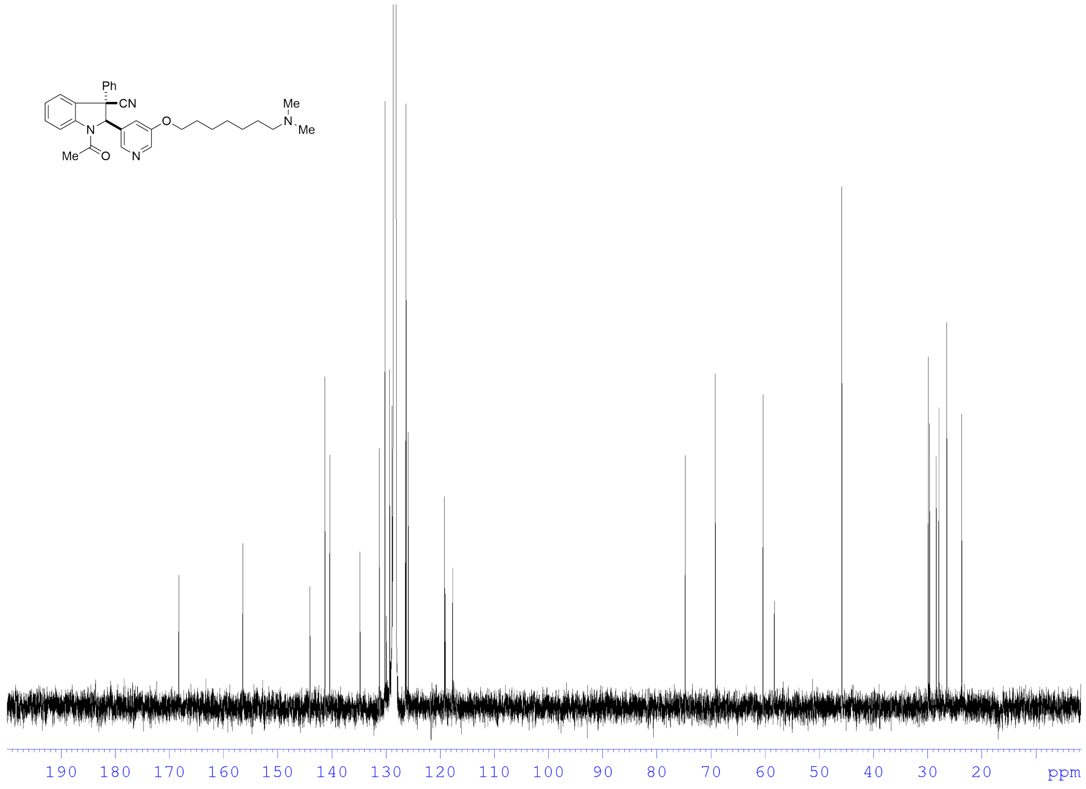
Ph
CN
O
N
Me
Me
N
Me
O
N
190
180
170
160
150
140
130
120
110
100
90
80
70
60
50
40
30
20
ppm

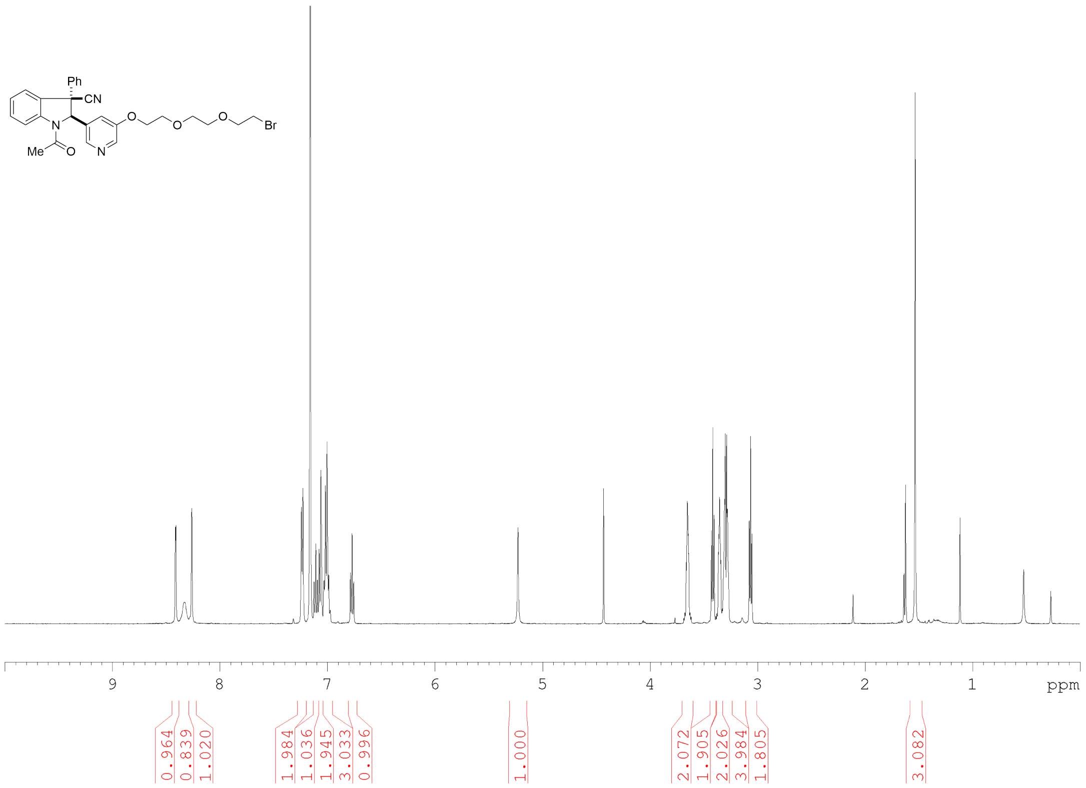
Ph
CN
N
Me
O
O
O
O
Br
N
9
8
7
6
5
4
3
2
1
ppm
0.964
0.839
1.020
1.984
1.036
1.945
3.033
0.996
1.000
2.072
1.905
2.026
3.984
1.805
3.082

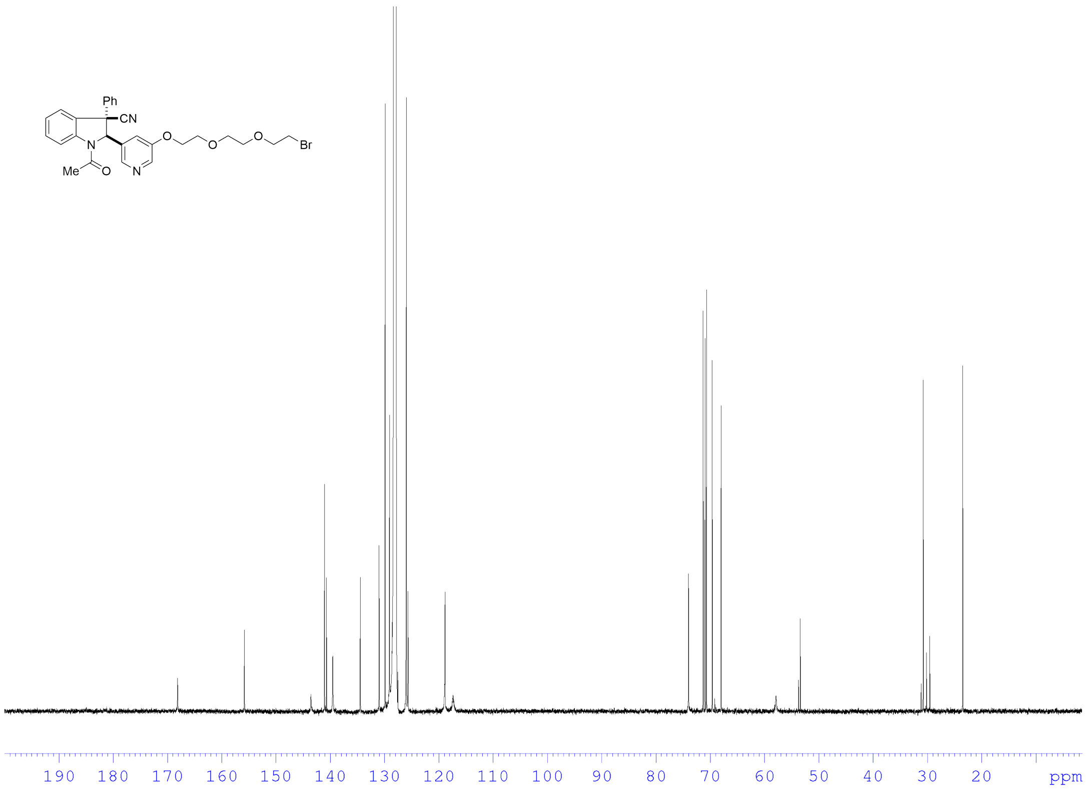
Ph
CN
N
Me
O
O
O
O
Br
N
190
180
170
160
150
140
130
120
110
100
90
80
70
60
50
40
30
20
ppm

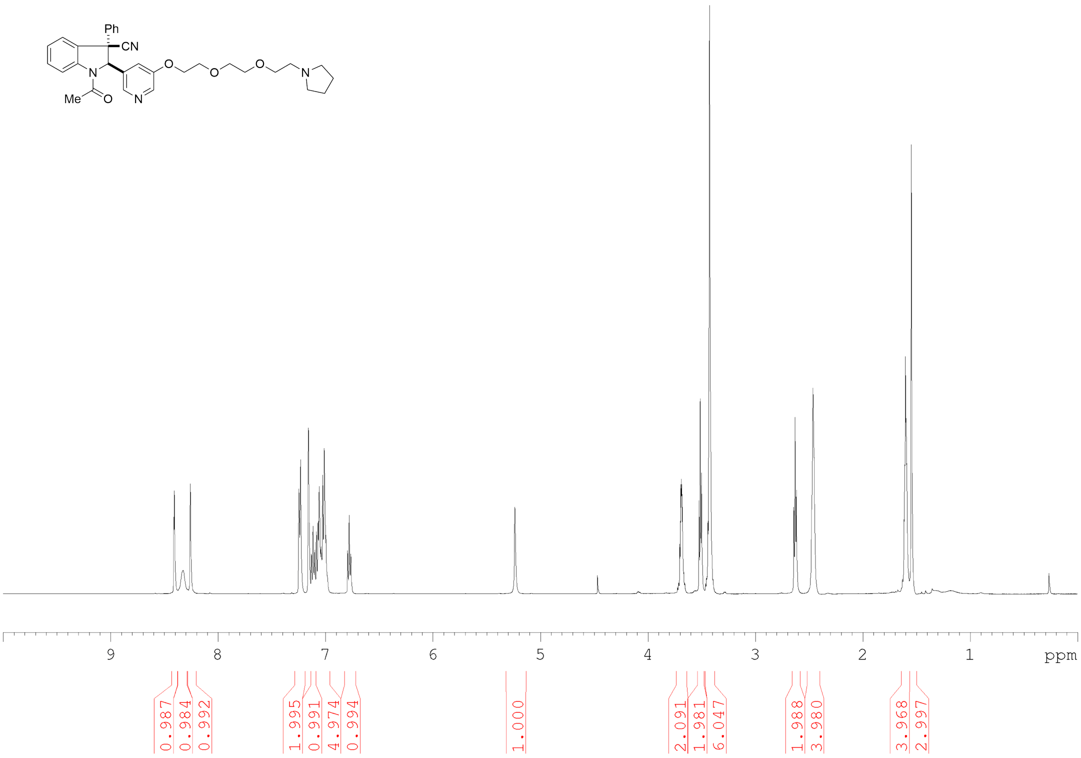
Ph
CN
O
O
O
N
N
N
Me
O
9 8 7 6 5 4 3 2 1 ppm
0.987 0.984 0.992 1.995 0.991 4.974 0.994 1.000 2.091 1.981 6.047 1.988 3.980 3.968 2.997

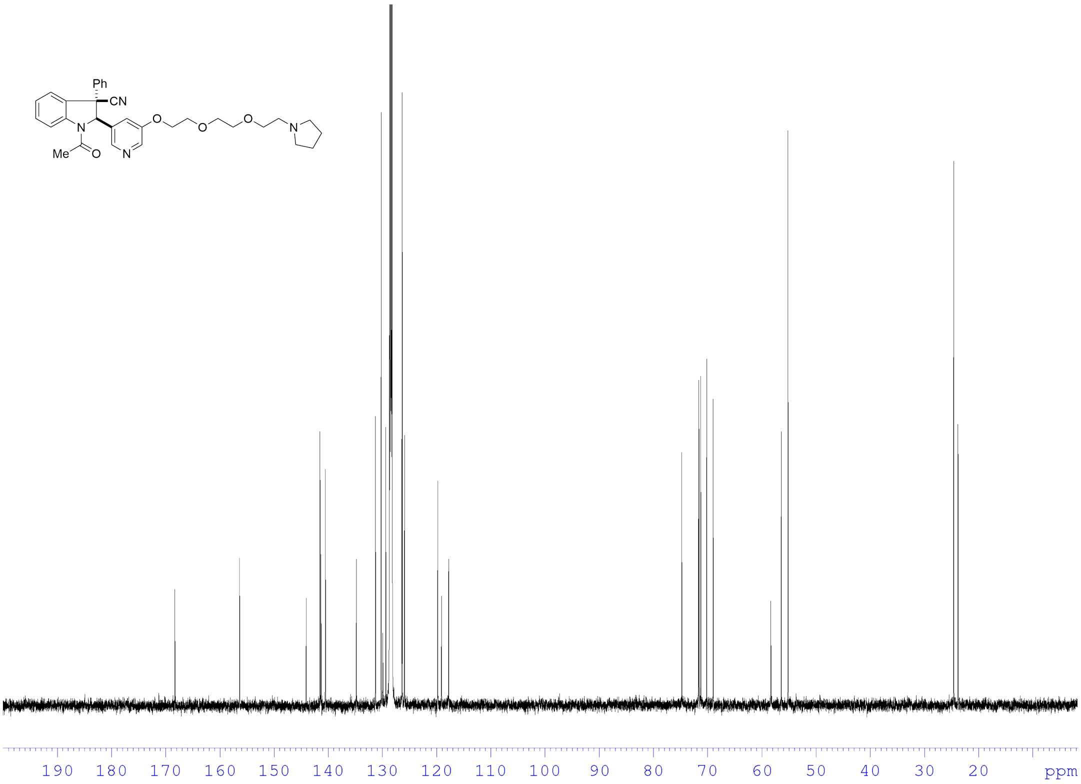
Ph
CN
O
O
O
N
N
N
Me
O
190 180 170 160 150 140 130 120 110 100 90 80 70 60 50 40 30 20 ppm

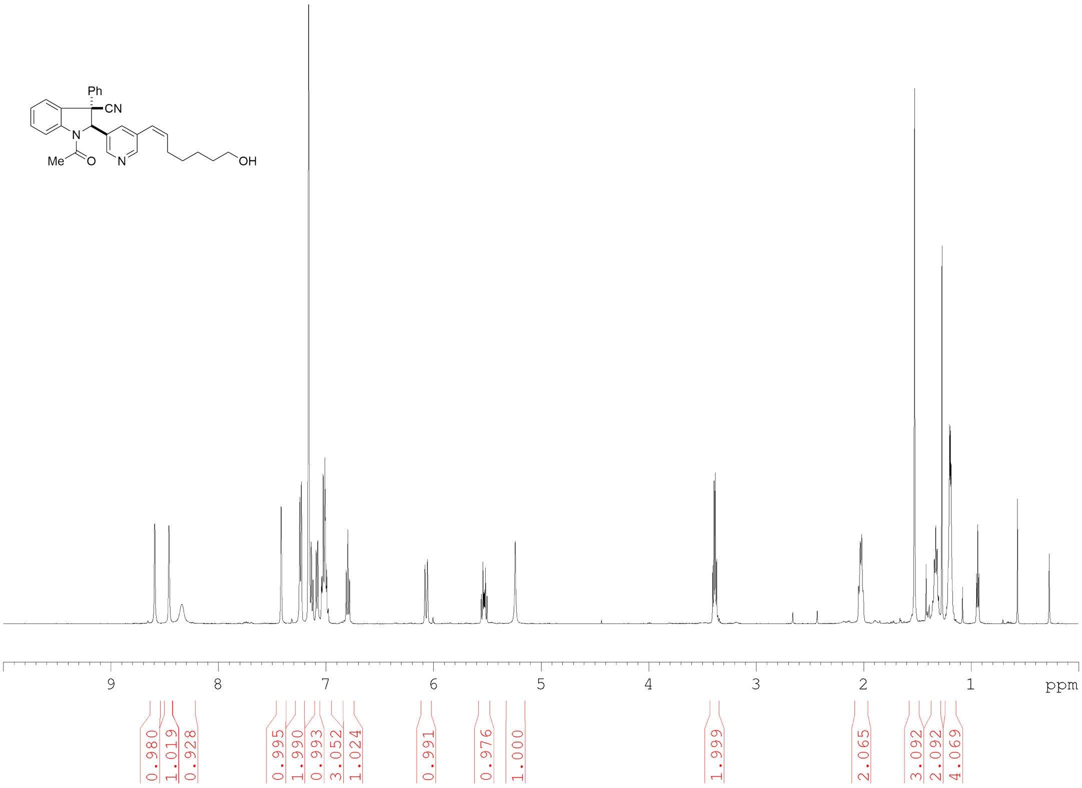

Ph
CN
N
Me
O
N
OH
9
8
7
6
5
4
3
2
1
ppm
0.980
1.019
0.928
0.995
1.990
0.993
3.052
1.024
0.991
0.976
1.000
1.999
2.065
3.092
2.092
4.069

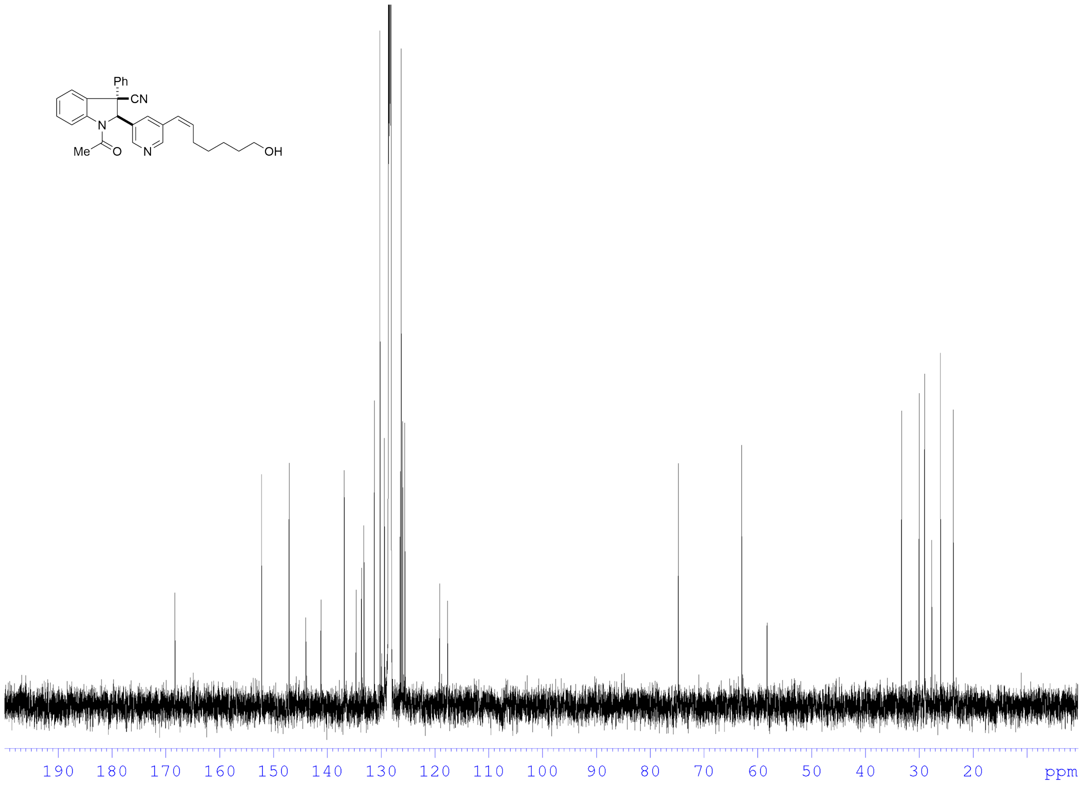

Ph
CN
N
Me
O
N
OH
190
180
170
160
150
140
130
120
110
100
90
80
70
60
50
40
30
20
ppm

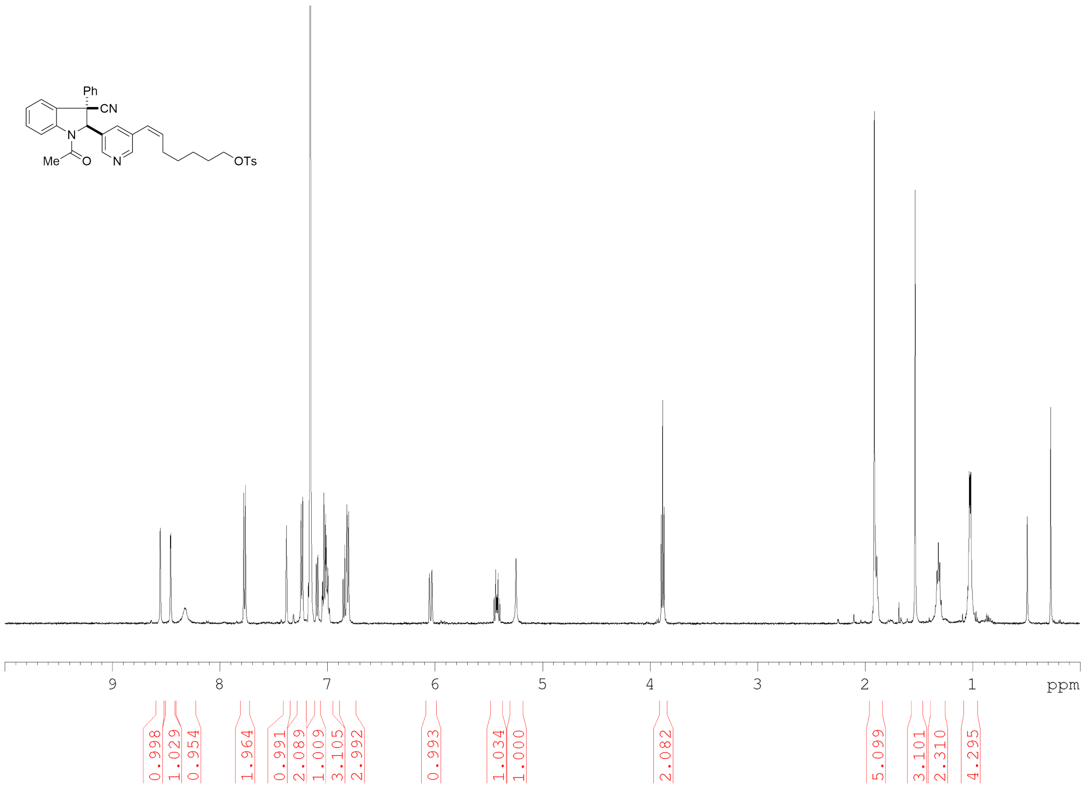
Ph
CN
N
Me
O
N
OTs
9
8
7
6
5
4
3
2
1
ppm
0.998
1.029
0.954
1.964
0.991
2.089
1.009
3.105
2.992
0.993
1.034
1.000
2.082
5.099
3.101
2.310
4.295

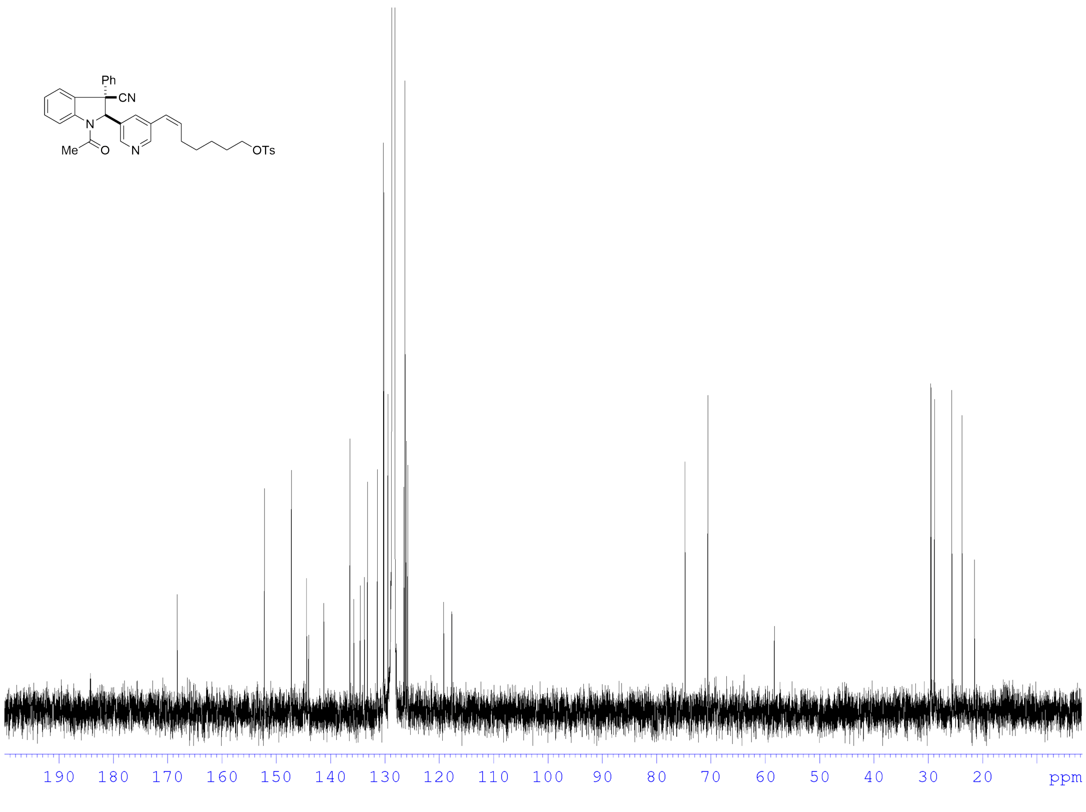
Ph
CN
N
Me
O
N
OTs
190
180
170
160
150
140
130
120
110
100
90
80
70
60
50
40
30
20
ppm

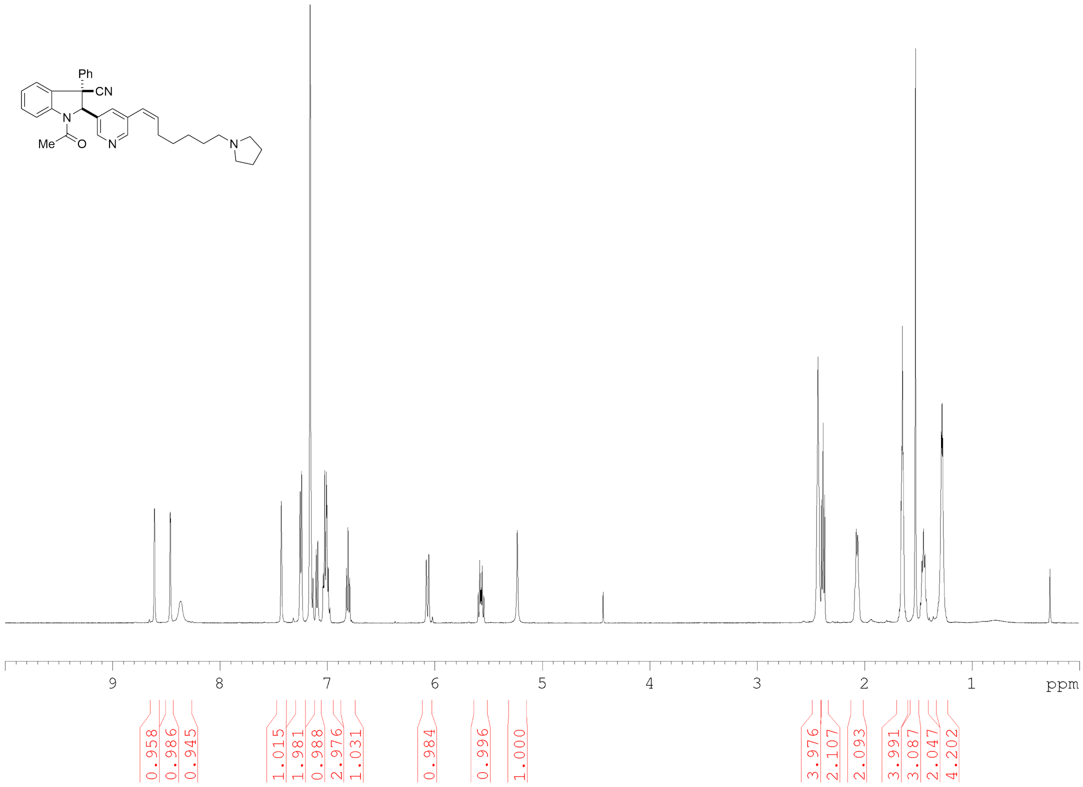
Ph
CN
N
Me
O
N
N
9
8
7
6
5
4
3
2
1
ppm
0.958
0.986
0.945
1.015
1.981
0.988
2.976
1.031
0.984
0.996
1.000
3.976
2.107
2.093
3.991
3.087
2.047
4.202

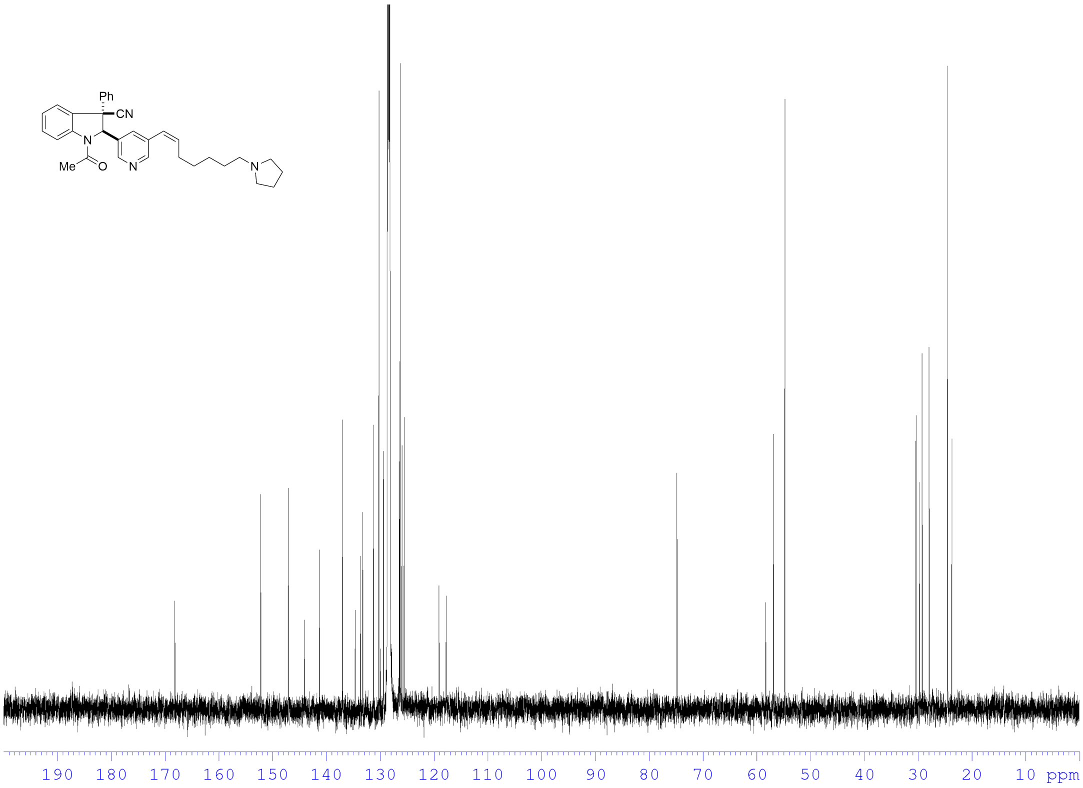
Ph
CN
N
Me
O
N
N
190
180
170
160
150
140
130
120
110
100
90
80
70
60
50
40
30
20
10
ppm

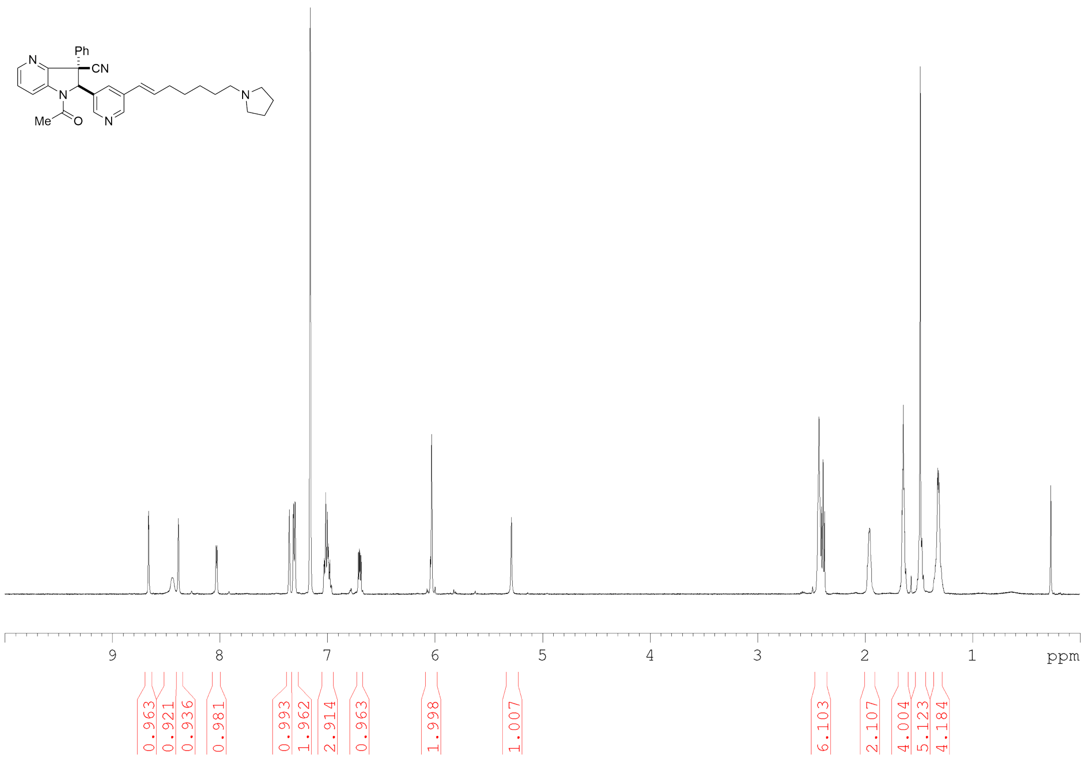
Ph
CN
N
N
N
N
Me
O
9
8
7
6
5
4
3
2
1
ppm
0.963
0.921
0.936
0.981
0.993
1.962
2.914
0.963
1.998
1.007
6.103
2.107
4.004
5.123
4.184

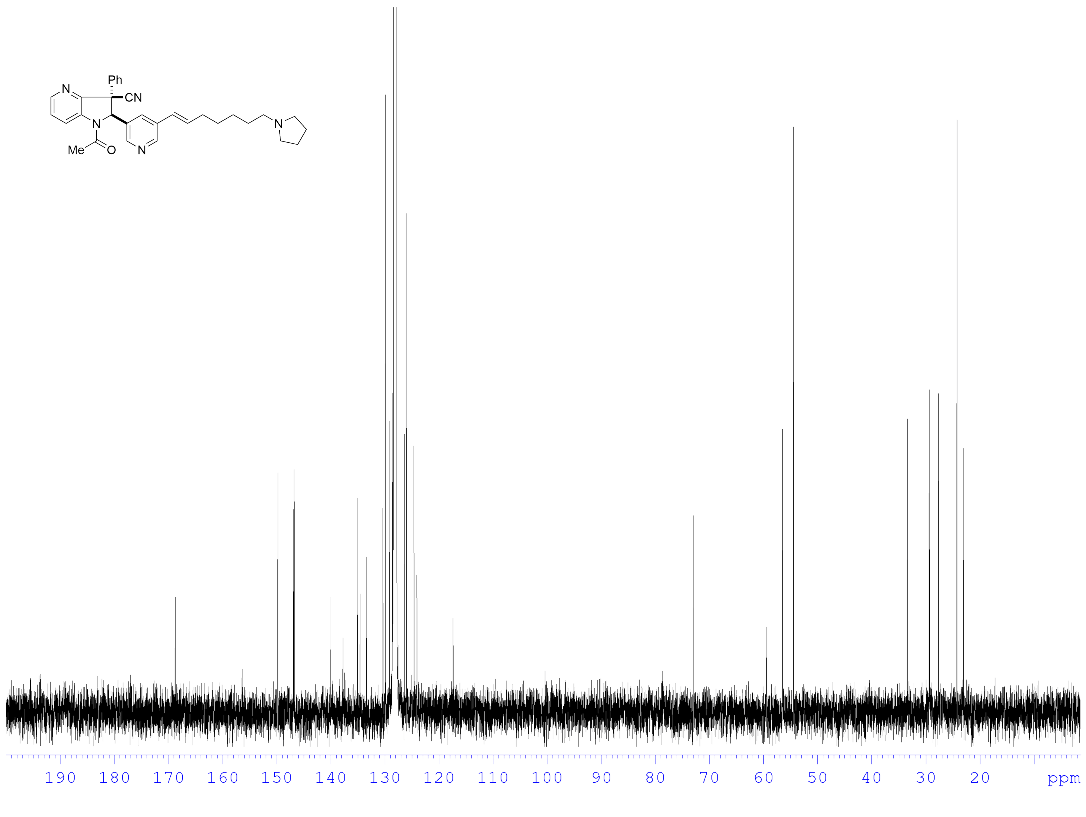
Ph
CN
N
N
N
N
Me
O
190
180
170
160
150
140
130
120
110
100
90
80
70
60
50
40
30
20
ppm

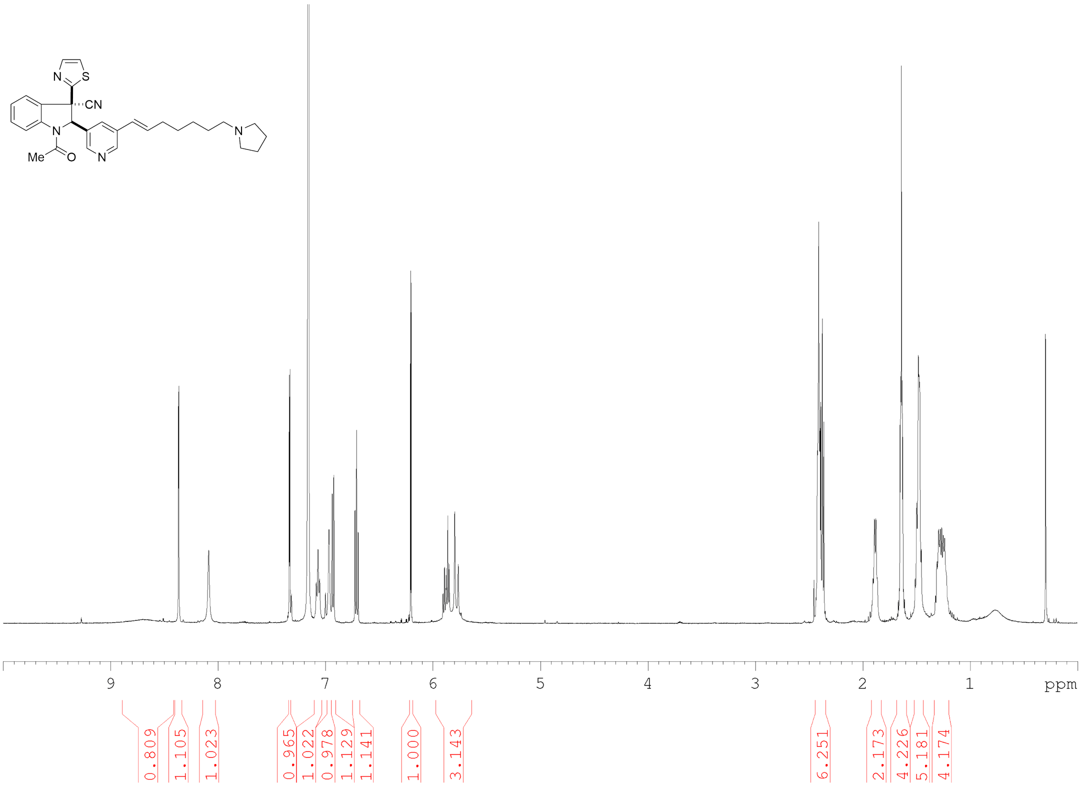
N
S
CN
N
Me
O
N
N
9
8
7
6
5
4
3
2
1
ppm
0.809
1.105
1.023
0.965
1.022
0.978
1.129
1.141
1.000
3.143
6.251
2.173
4.226
5.181
4.174

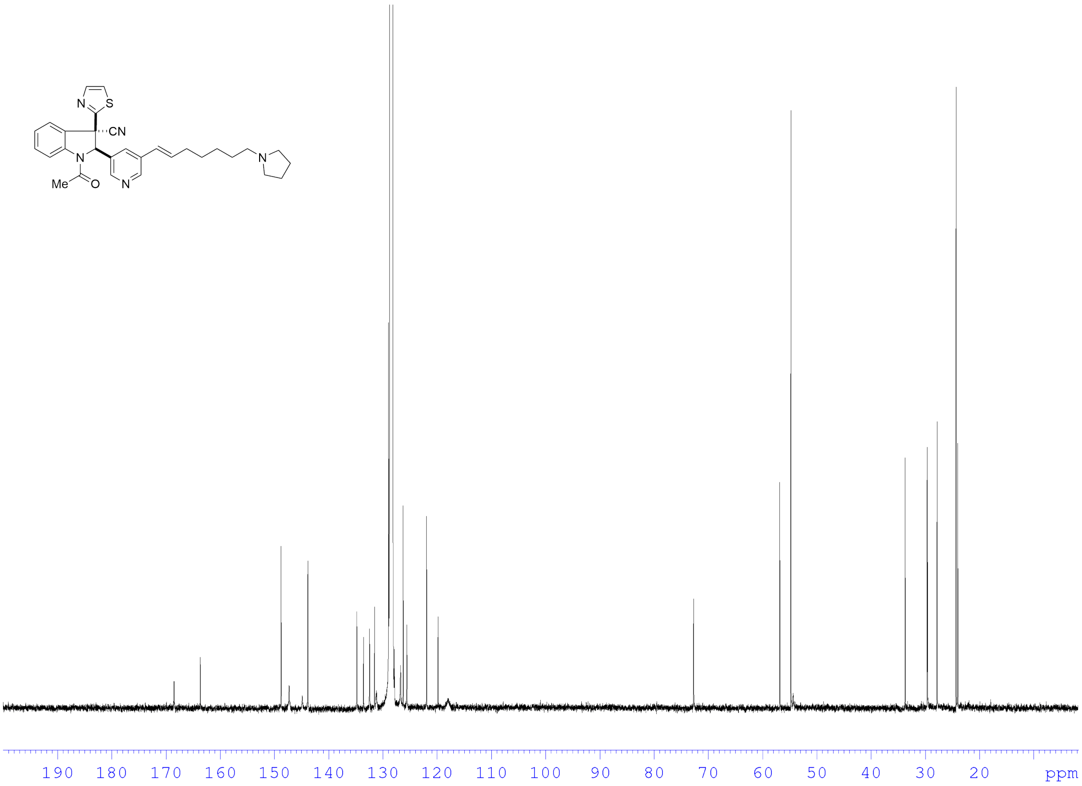
N
S
CN
N
Me
O
N
N
190
180
170
160
150
140
130
120
110
100
90
80
70
60
50
40
30
20
ppm

X) HPLC Traces

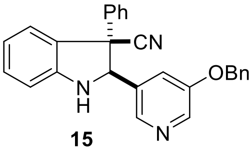

15

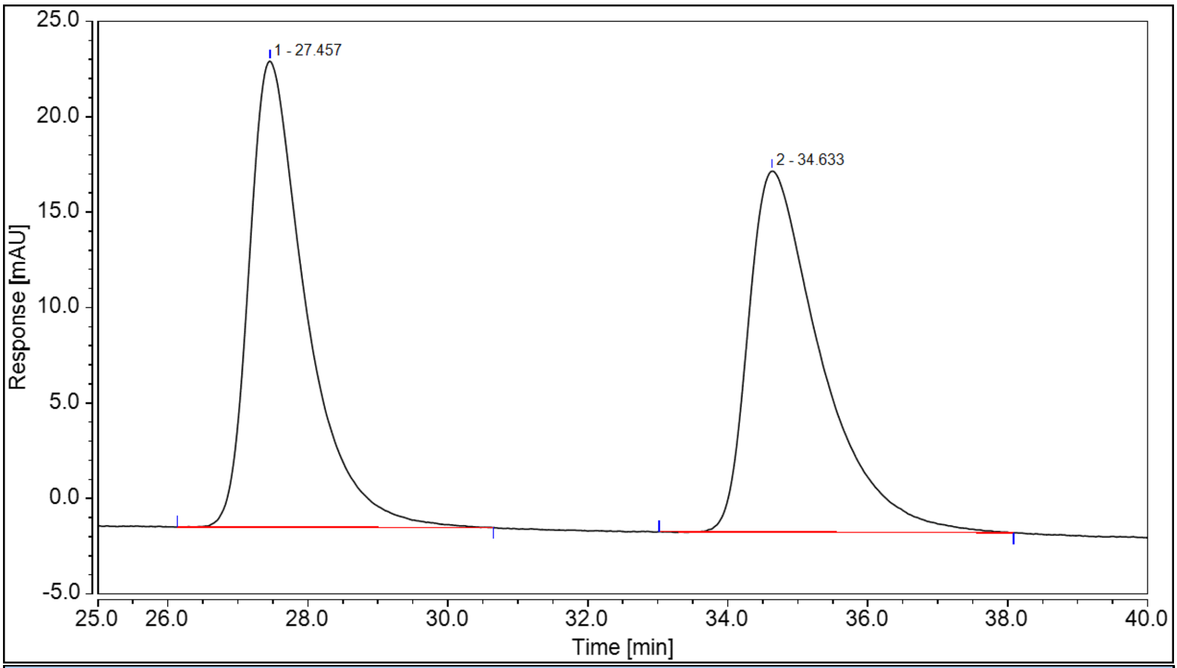

Integration Results

No.	Peak Name	Retention Time min	Area mAU*min	Height mAU	Relative Area %	Relative Height %	Amount
n.a.	Peak 1	n.a.	n.a.	n.a.	n.a.	n.a.	n.a.
1		27.457	23.060	24.426	50.37	56.36	n.a.
2		34.633	22.723	18.910	49.63	43.64	n.a.
**Total:**			**45.783**	**43.336**	**100.00**	**100.00**	

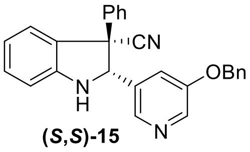

(*S*,*S*)-15

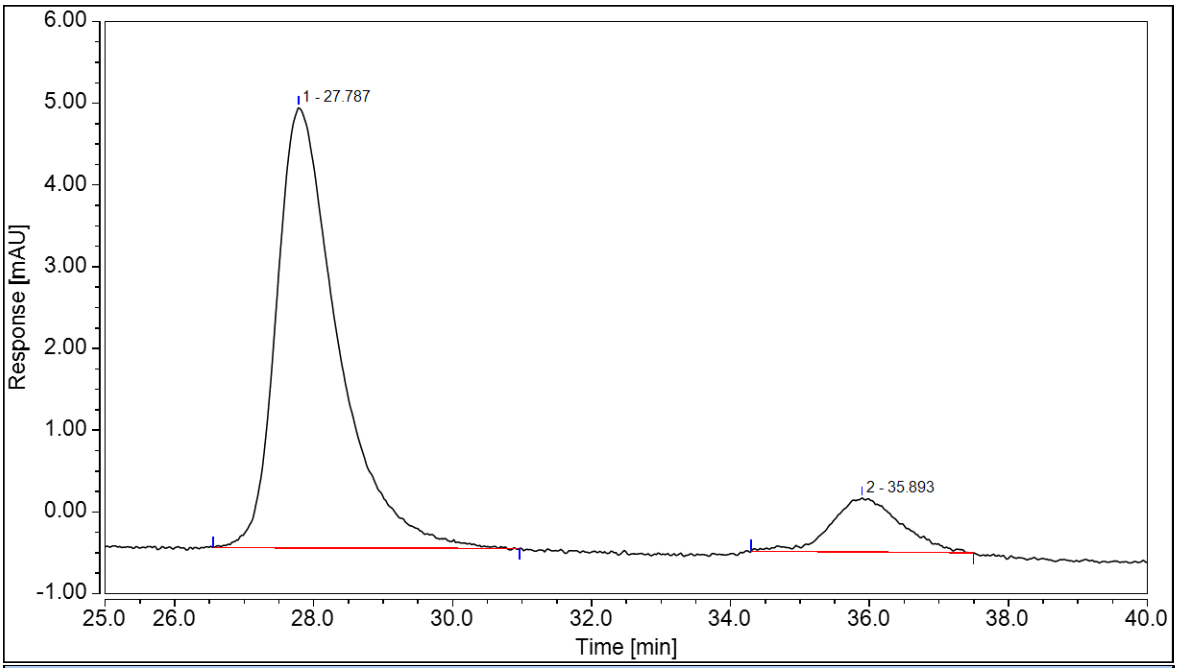

Integration Results							
No.	Peak Name	Retention Time min	Area mAU*min	Height mAU	Relative Area %	Relative Height %	Amount
n.a.	Peak 1	n.a.	n.a.	n.a.	n.a.	n.a.	n.a.
1		27.787	5.507	5.390	87.68	89.06	n.a.
2		35.893	0.774	0.662	12.32	10.94	n.a.
**Total:**			**6.281**	**6.053**	**100.00**	**100.00**	

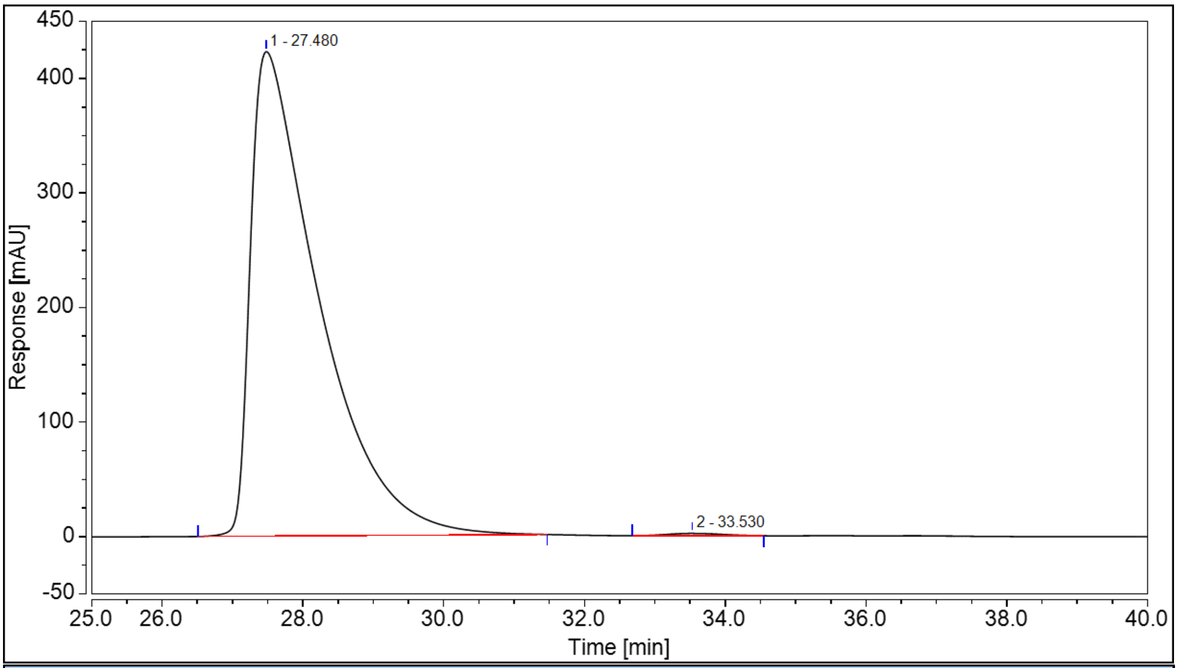

Integration Results							
No.	Peak Name	Retention Time min	Area mAU*min	Height mAU	Relative Area %	Relative Height %	Amount
n.a.	Peak 1	n.a.	n.a.	n.a.	n.a.	n.a.	n.a.
1		27.480	474.103	423.118	99.61	99.51	n.a.
2		33.530	1.877	2.069	0.39	0.49	n.a.
**Total:**			**475.979**	**425.187**	**100.00**	**100.00**	

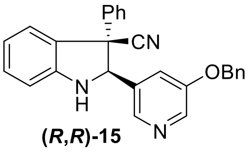

(*R*,*R*)-15

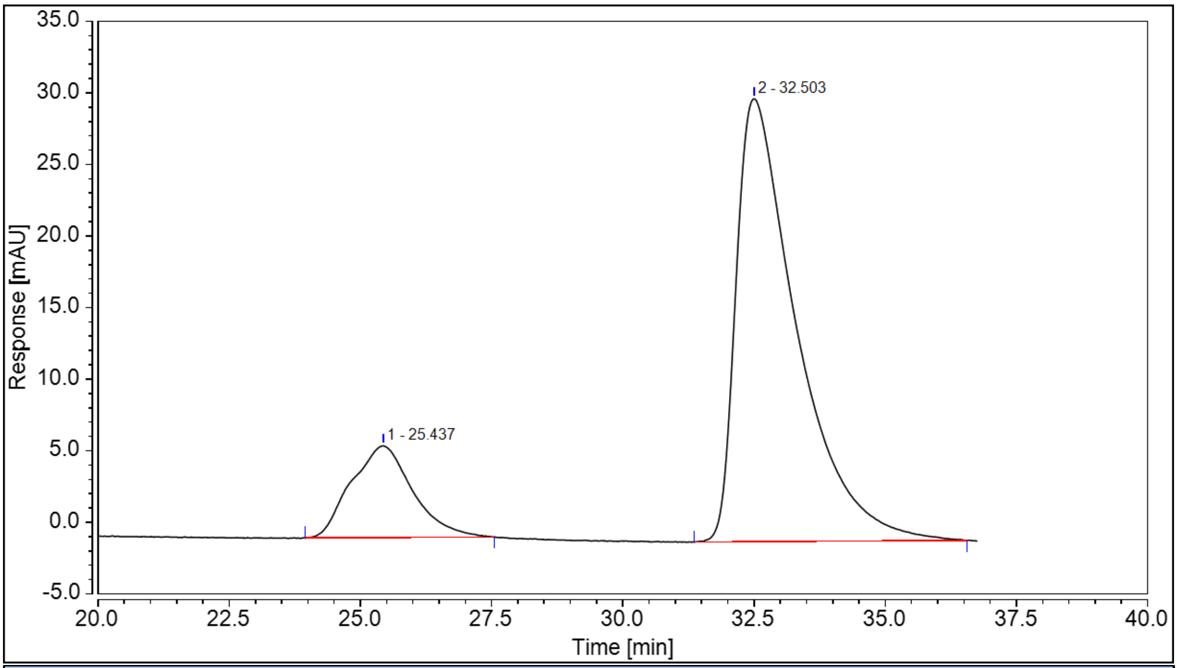

Integration Results							
No.	Peak Name	Retention Time min	Area mAU*min	Height mAU	Relative Area %	Relative Height %	Amount
n.a.	Peak 1	n.a.	n.a.	n.a.	n.a.	n.a.	n.a.
1		25.437	8.708	6.418	17.41	17.18	n.a.
2		32.503	41.297	30.945	82.59	82.82	n.a.
**Total:**			**50.006**	**37.363**	**100.00**	**100.00**	

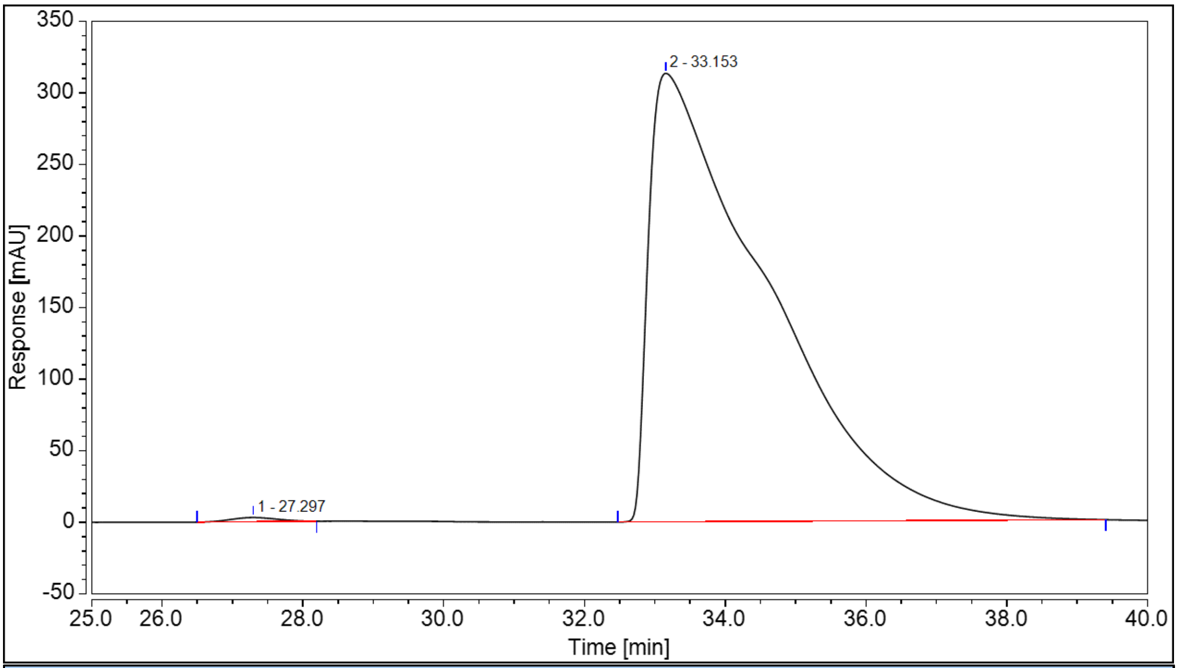

Integration Results							
No.	Peak Name	Retention Time min	Area mAU*min	Height mAU	Relative Area %	Relative Height %	Amount
n.a.	Peak 1	n.a.	n.a.	n.a.	n.a.	n.a.	n.a.
1		27.297	2.224	2.924	0.36	0.92	n.a.
2		33.153	608.014	313.355	99.64	99.08	n.a.
**Total:**			**610.238**	**316.279**	**100.00**	**100.00**	

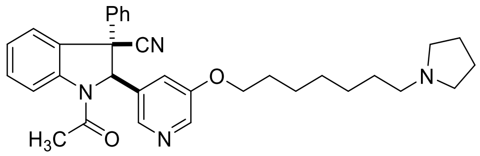

3

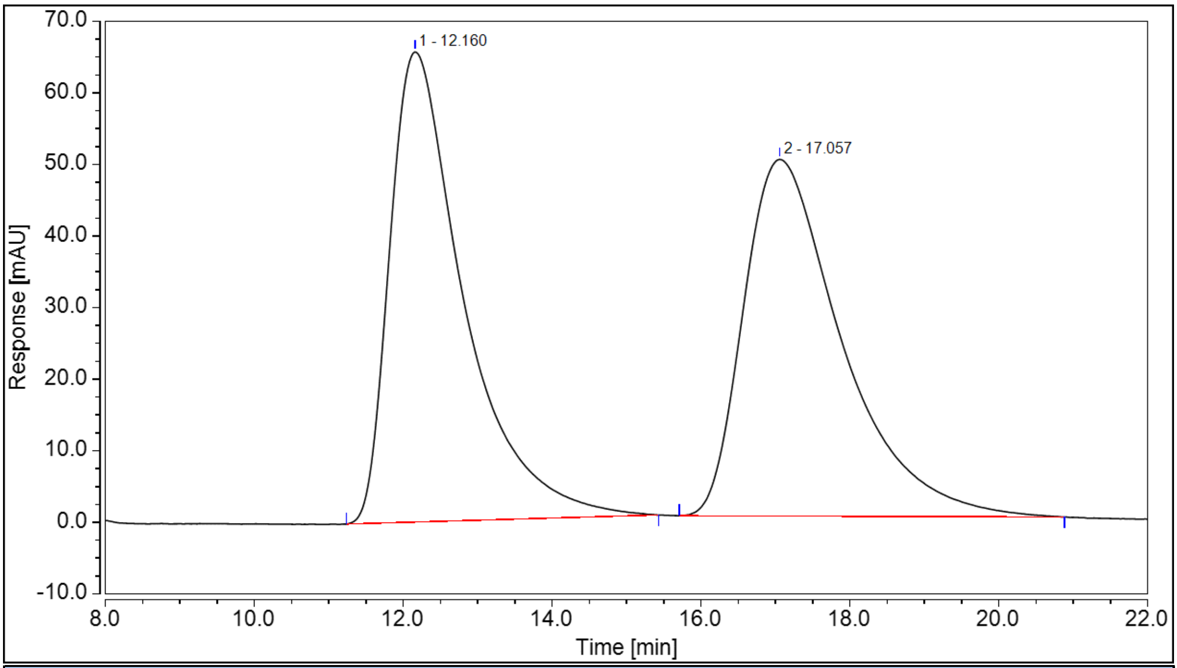

Integration Results

No.	Peak Name	Retention Time min	Area mAU*min	Height mAU	Relative Area %	Relative Height %	Amount
n.a.	Peak 1	n.a.	n.a.	n.a.	n.a.	n.a.	n.a.
1		12.160	75.775	65.695	49.85	56.86	n.a.
2		17.057	76.239	49.842	50.15	43.14	n.a.
**Total:**			**152.013**	**115.537**	**100.00**	**100.00**	

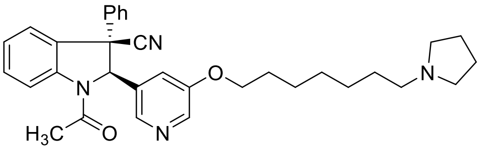

(*R*,*R*)-3

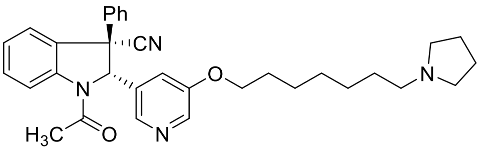

(*S*,*S*)-3

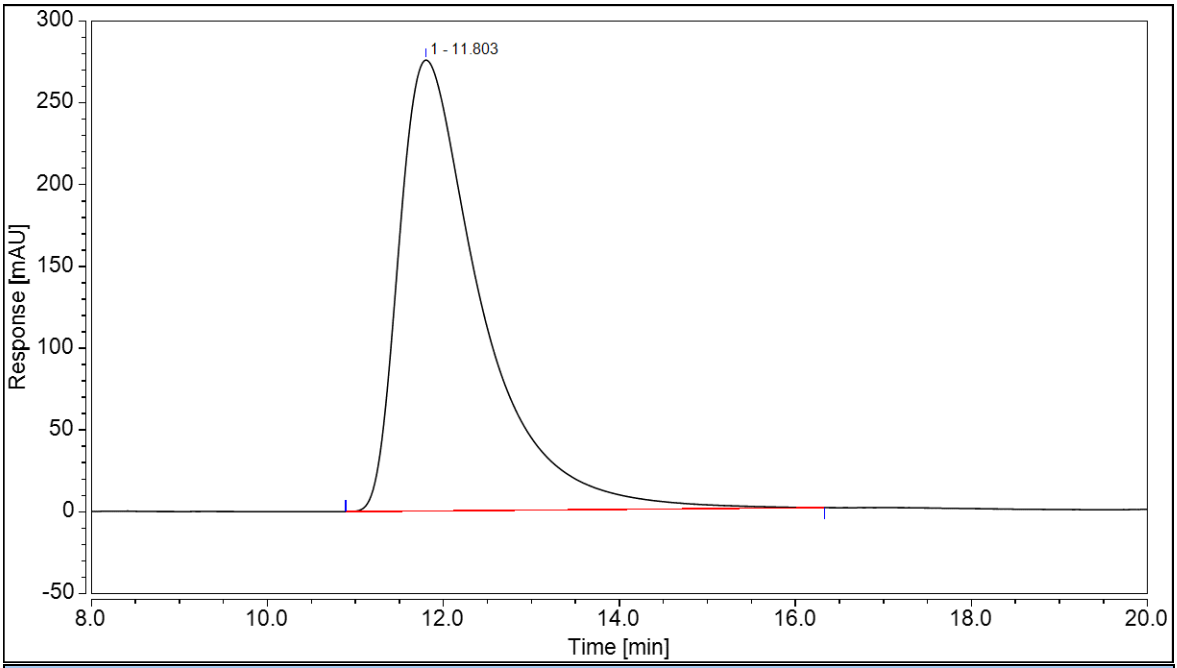

Integration Results

No.	Peak Name	Retention Time<br>min	Area<br>mAU*min	Height<br>mAU	Relative Area<br>%	Relative Height<br>%	Amount
n.a.	Peak 1	n.a.	n.a.	n.a.	n.a.	n.a.	n.a.
1		11.803	299.556	275.728	100.00	100.00	n.a.
**Total:**			**299.556**	**275.728**	**100.00**	**100.00**	

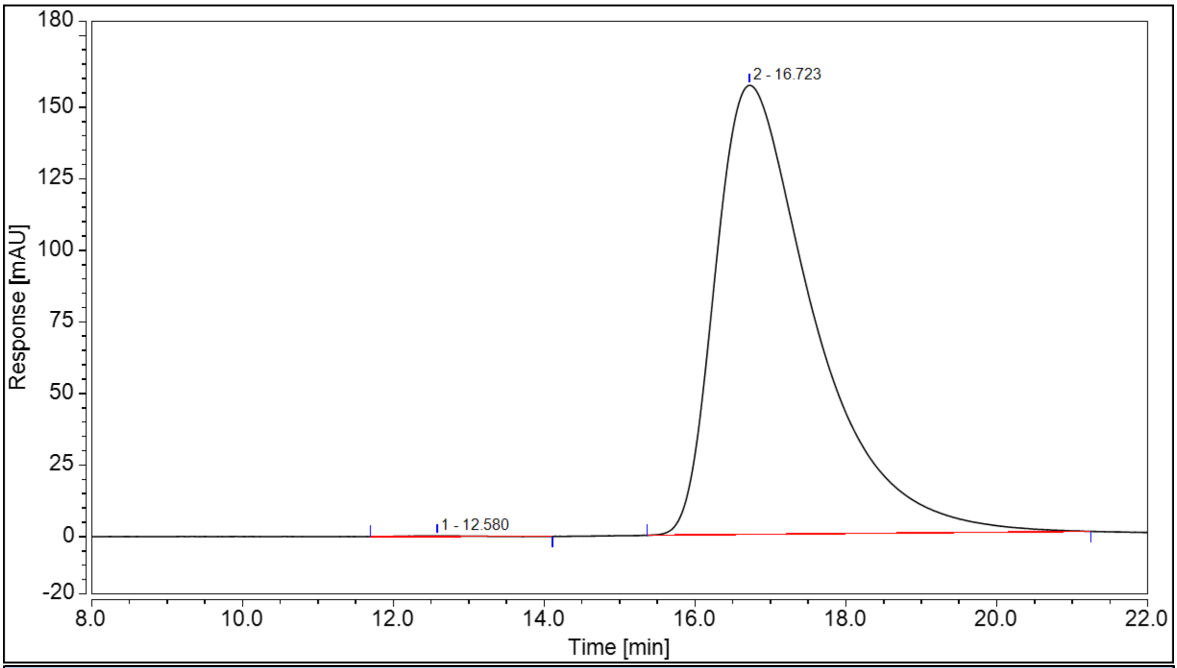

Integration Results

No.	Peak Name	Retention Time<br>min	Area<br>mAU*min	Height<br>mAU	Relative Area<br>%	Relative Height<br>%	Amount
n.a.	Peak 1	n.a.	n.a.	n.a.	n.a.	n.a.	n.a.
1		12.580	0.207	0.245	0.09	0.16	n.a.
2		16.723	237.678	156.873	99.91	99.84	n.a.
**Total:**			**237.886**	**157.119**	**100.00**	**100.00**	

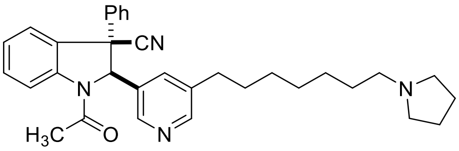

6

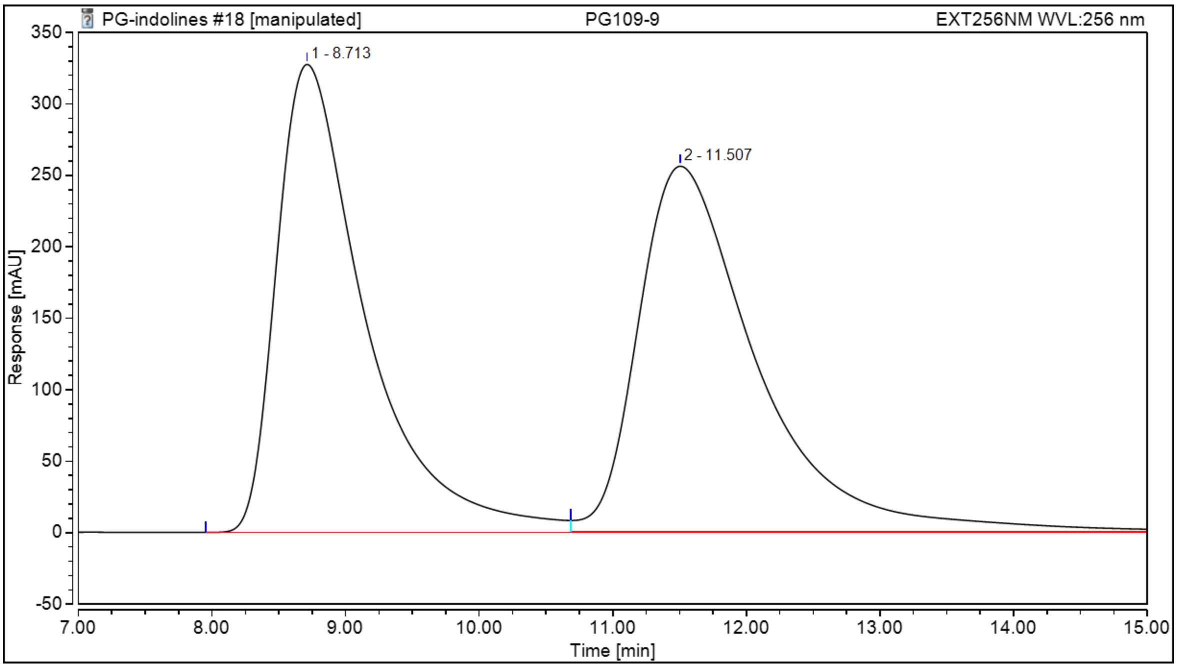

Integration Results							
No.	Peak Name	Retention Time min	Area mAU*min	Height mAU	Relative Area %	Relative Height %	Amount
n.a.	Peak 1	n.a.	n.a.	n.a.	n.a.	n.a.	n.a.
1		8.713	252.388	327.471	48.69	56.12	n.a.
2		11.507	266.010	256.089	51.31	43.88	n.a.
**Total:**			**518.397**	**583.560**	**100.00**	**100.00**	

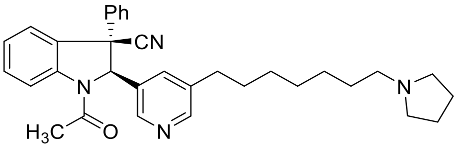

(*R*,*R*)-**6**

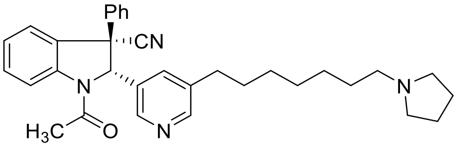

(*S*,*S*)-**6**

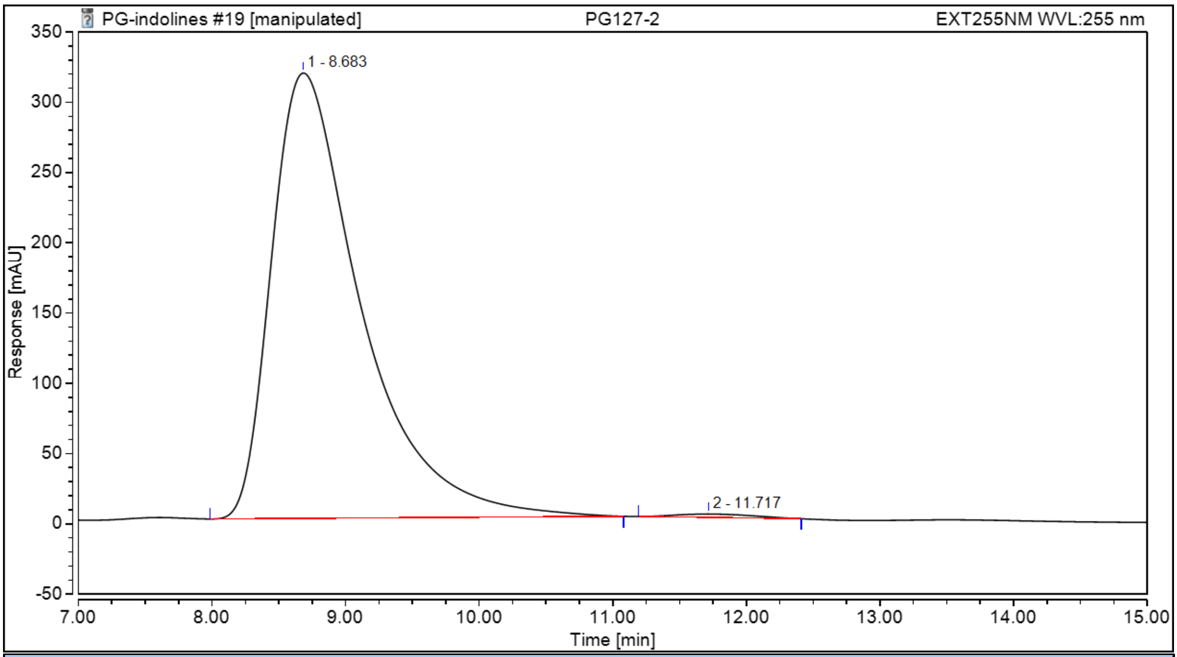

Integration Results

No.	Peak Name	Retention Time min	Area mAU*min	Height mAU	Relative Area %	Relative Height %	Amount
n.a.	Peak 1	n.a.	n.a.	n.a.	n.a.	n.a.	n.a.
1		8.683	246.252	316.982	99.33	99.24	n.a.
2		11.717	1.659	2.436	0.67	0.76	n.a.
**Total:**			**247.911**	**319.418**	**100.00**	**100.00**	

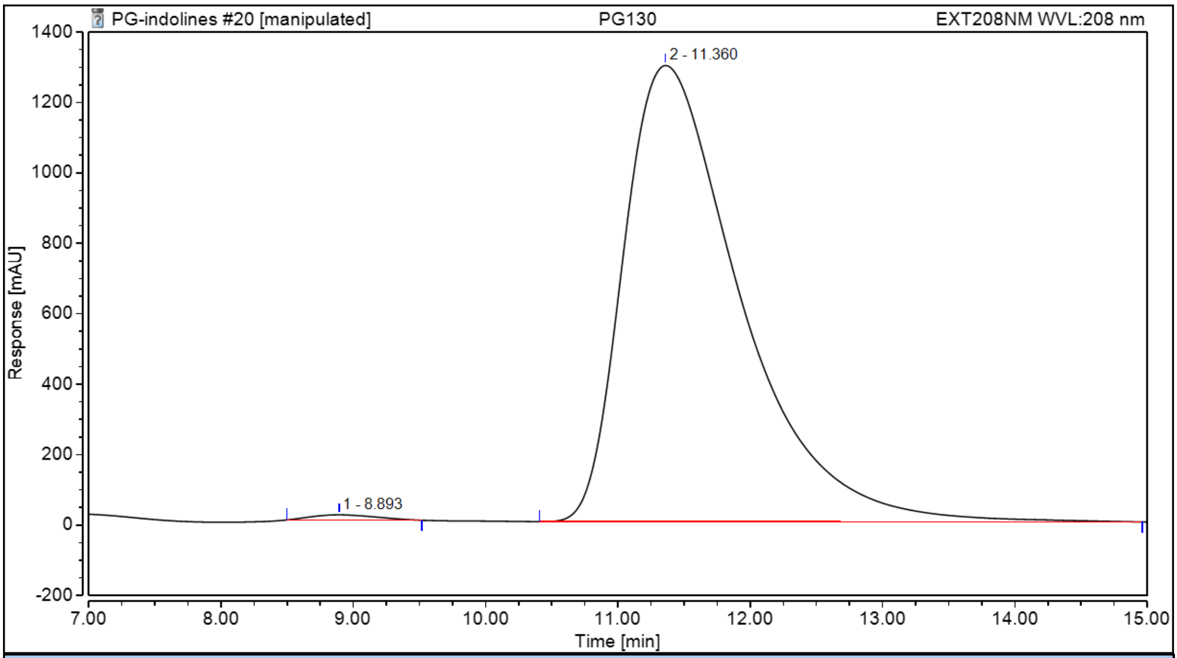

Integration Results

No.	Peak Name	Retention Time min	Area mAU*min	Height mAU	Relative Area %	Relative Height %	Amount
n.a.	Peak 1	n.a.	n.a.	n.a.	n.a.	n.a.	n.a.
1		8.893	8.271	14.658	0.62	1.12	n.a.
2		11.360	1328.356	1295.627	99.38	98.88	n.a.
**Total:**			**1336.627**	**1310.284**	**100.00**	**100.00**	

XI) X-ray Crystallography Data

<u>X-ray Crystallographic Data and Structure Refinement for 77, 007JDJ14</u>

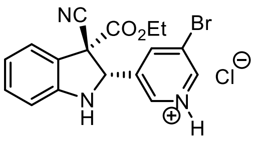

85

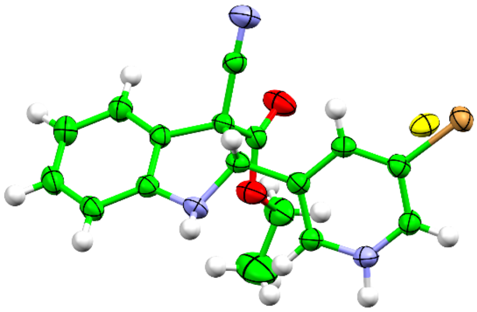

Identification code	007JDJ14	
Empirical formula	$C_{17}H_{15}BrClN_3O_2$	
Formula weight	408.68	
Temperature	150 K	
Wavelength	0.71073 Å	
Crystal system	Triclinic	
Space group	C 2/c	
Unit cell dimensions	a = 41.1184 Å	α = 90°
	b = 8.77720 Å	β = 113.8427°
	c = 22.7766 Å	γ = 90 °
Volume	7518.66 Å^3	
Z,Z’	Z: 16 Z': 0	
Density (calculated)	1.44 Mg m^{-3}	
Absorption coefficient	2.342 mm^{-1}	
F(000)	3296.0	
Crystal size	0.04 x 0.18 x 0.40 mm ^{3}	
Theta range for data collection	5.122° to 27.496°	
Reflections collected	15307	
Independent reflections	8581	
Absorption correction	Multi-scan	
Refinement method	Full-matrix least squares on F^2	
Goodness-of-fit on F^2	0.974	
Final R indices [I>2σ(I)]	R^1 = 0.0656, wR^2 = 0.1703	
R indices (all data)	R^1 = 0.0888, wR^2 = 0.1852	

X-ray Crystallographic Data and Structure Refinement for 100, 030JDJ15

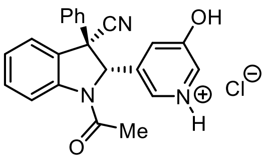

(*S*,*S*)-17

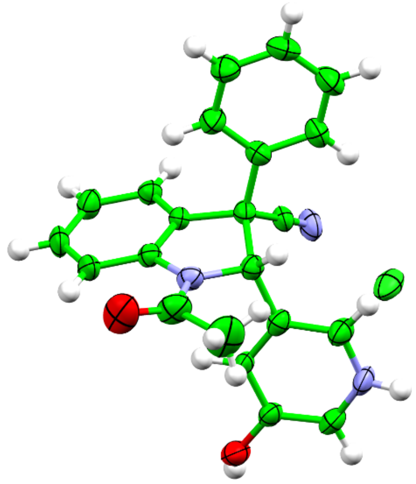

Identification code	030JDJ15	
Empirical formula	$C_{23}H_{21}ClN_3O_{2.5}$	
Formula weight	414.89	
Temperature	150 K	
Wavelength	1.54180 Å	
Crystal system	Triclinic	
Space group	P 1	
Unit cell dimensions	a = 8.6005 Å	α = 103.198°
	b = 8.7390 Å	β = 92.916°
	c = 15.6102 Å	γ = 98.395°
Volume	1125.70 $Å^3$	
Z,Z'	Z: 2 Z': 0	
Density (calculated)	1.22 Mg m^{-3}	
Absorption coefficient	1.706 mm^{-1}	
F(000)	434.0	
Crystal size	0.01 x 0.03 x 0.20 mm^3	
Theta range for data collection	5.218° to 75.980°	
Reflections collected	22875	
Independent reflections	8722	
Absorption correction	Multi-scan	
Refinement	Full-matrix least squares	

method	on F^2
Goodness-of-fit on F^2	0.996
Final R indices [I>2σ(I)]	R^1 = 0.0753, wR^2 = 0.2132
R indices (all data)	R^1 = 0.0800, wR^2 = 0.2208

XII) References

1. Pangborn, A. B.; Giardello, M. A.; Grubbs, R. H.; Rosen, R. K.; Timmers, F. J., Safe and Convenient Procedure for Solvent Purification. *Organometallics* **1996,** *15* (5), 1518-1520.
2. Armarego, W. L. F.; Perrin, D. D., *Purification of Laboratory Chemicals*. 4 ed.; Butterworth Heinemann: Oxford, 1996.
3. Makosza, M.; Tomashewskij, A. A., Does Nitroarylation of Phenylacetonitrile Proceed as a Phase-Transfer Catalyzed Process? *J. Org. Chem.* **1995,** *60* (17), 5425-9.
4. MacMillan, K. S.; Nguyen, T.; Nguyen, T.; Hwang, I.; Boger, D. L., Total synthesis and evaluation of iso-duocarmycin SA and iso-yatakemycin. *J Am Chem Soc* **2009,** *131* (3), 1187-94.
5. Yamazaki, Y.; Toma, T.; Nishikawa, M.; Ozawa, H.; Okuda, A.; Araki, T.; Abe, K.; Oda, S. Preparation of azole compounds as PPARα agonists. WO2005023777A1, 2005.
6. Sharma, K.; Wolstenhulme, J. R.; Painter, P. P.; Yeo, D.; Grande-Carmona, F.; Johnston, C. P.; Tantillo, D. J.; Smith, M. D., Cation-Controlled Enantioselective and Diastereoselective Synthesis of Indolines: An Autoinductive Phase-Transfer Initiated 5-endo-trig Process. *J. Am. Chem. Soc.* **2015,** *137* (41), 13414-13424.
7. Choi, S. E.; Pflum, M. K. H., The structural requirements of histone deacetylase inhibitors: Suberoylanilide hydroxamic acid analogs modified at the C6 position. *Bioorganic & Medicinal Chemistry Letters* **2012,** *22* (23), 7084-7086.
8. Rosillo, M.; Arnáiz, E.; Abdi, D.; Blanco-Urgoiti, J.; Domínguez, G.; Pérez-Castells, J., Combination of RCM and the Pauson–Khand Reaction: One-Step Synthesis of Tricyclic Structures. *European Journal of Organic Chemistry* **2008,** *2008* (23), 3917-3927.
9. Mallia, C. J.; Englert, L.; Walter, G. C.; Baxendale, I. R., Thiazole formation through a modified Gewald reaction. *Beilstein J. Org. Chem.* **2015,** *11*, 875-883.
10. Morales, S.; Guijarro, F. G.; Garcia Ruano, J. L.; Cid, M. B., A General Aminocatalytic Method for the Synthesis of Aldimines. *J. Am. Chem. Soc.* **2014,** *136* (3), 1082-1089.
11. Jackson, R. F. W.; Wishart, N.; Wood, A.; James, K.; Wythes, M. J., Preparation of enantiomerically pure protected 4-oxo α-amino acids and 3-aryl α-amino acids from serine. *J. Org. Chem.* **1992,** *57* (12), 3397-404.
12. Ahad, A. M.; Jensen, S. M.; Jewett, J. C., A Traceless Staudinger Reagent To Deliver Diazirines. *Org. Lett.* **2013,** *15* (19), 5060-5063.
13. Caldarelli, S. A.; El Fangour, S.; Wein, S.; Tran van Ba, C.; Perigaud, C.; Pellet, A.; Vial, H. J.; Peyrottes, S., New Bis-thiazolium Analogues as Potential Antimalarial Agents: Design, Synthesis, and Biological Evaluation. *J. Med. Chem.* **2013,** *56* (2), 496-509.

14. Wei, Z.-L.; Petukhov, P. A.; Bizik, F.; Teixeira, J. C.; Mercola, M.; Volpe, E. A.; Glazer, R. I.; Willson, T. M.; Kozikowski, A. P., Isoxazolyl-serine-based agonists of peroxisome proliferator-activated receptor: design, synthesis, and effects on cardiomyocyte differentiation. *J. Am. Chem. Soc.* **2004,** *126* (51), 16714-16715.

15. Altendorfer, M.; Raja, A.; Sasse, F.; Irschik, H.; Menche, D., Modular synthesis of polyene side chain analogues of the potent macrolide antibiotic etnangien by a flexible coupling strategy based on hetero-bis-metallated alkenes. *Org. Biomol. Chem.* **2013,** *11* (13), 2116-2139.

16. Coombs, J. R.; Zhang, L.; Morken, J. P., Enantiomerically Enriched Tris(boronates): Readily Accessible Conjunctive Reagents for Asymmetric Synthesis. *J. Am. Chem. Soc.* **2014,** *136* (46), 16140-16143.

17. Takami, K.; Yorimitsu, H.; Oshima, K., Trans-Hydrometalation of Alkynes by a Combination of InCl3 and DIBAL-H: One-Pot Access to Functionalized (Z)-Alkenes. *Org. Lett.* **2002,** *4* (17), 2993-2995.

18. Kondrat, F.D., Struwe, W.B., & Benesch, J.L.P., Native mass spectrometry: towards high-throughput structural proteomics, *Methods Mol. Biol.* (2014), 1261:349-71.

19. Lamb, A. D., Asymmetric synthesis of heterocycles via cation-directed cyclizations and rearrangements. University of Oxford, Oxford, 2014.

20. Trott, O.; Olson, A. J., AutoDock Vina: Improving the speed and accuracy of docking with a new scoring function, efficient optimization, and multithreading. *J. Comput. Chem.* **2010**, *31* (2), 455-461.

21. Cheng, Z.; Cheung, P.; Kuo, A. J.; Yukl, E. T.; Wilmot, C. M.; Gozani, O.; Patel, D. J.; A molecular threading mechanism underlies Jumonji lysine demethylase KDM2A regulation of methylated H3K36. *Genes Dev.* **2014,** *28* (16), 1758-1771.

22. Rotili, D., Altun, M., Kawamura, A., Wolf, A., Fischer, R., Leung, I.K.H., Mackeen, M.M., Tian, Y., Ratcliffe, P.J., Mai, A., Kessler, B.M., and Schofield, C.J. (2011) Article A Photoreactive Small-Molecule Probe for 2-Oxoglutarate Oxygenases. *Chem. Biol.*, **18** (5), 642–654.

23. Hopkinson, R.J., Tumber, A., Yapp, C., Chowdhury, R., Aik, W., Che, K.H., Li, X.S., Kristensen, J.B.L., King, O.N.F., Chan, M.C., Yeoh, K.K., Choi, H., Walport, L.J., Thinnes, C.C., Bush, J.T., Lejeune, C., Rydzik, A.M., Rose, N.R., Bagg, E. a., McDonough, M. a., Krojer, T.J., Yue, W.W., Ng, S.S., Olsen, L., Brennan, P.E., Oppermann, U., Müller, S., Klose, R.J., Ratcliffe, P.J., Schofield, C.J., and Kawamura, A. (2013) 5-Carboxy-8-hydroxyquinoline is a broad spectrum 2-oxoglutarate oxygenase inhibitor which causes iron translocation. *Chem. Sci.*, 3110–3117.

24. Johansson, C., Velupillai, S., Tumber, A., Szykowska, A., Hookway, E.S., Nowak, R.P., Strain-Damerell, C., Gileadi, C., Philpott, M., Burgess-Brown, N., Wu, N., Kopec, J., Nuzzi, A., Steuber, H., Egner, U., Badock, V., Munro, S., LaThangue, N.B., Westaway, S., Brown, J., Athanasou, N., Prinjha, R., Brennan, P.E., and Oppermann, U. (2016) Structural analysis of human KDM5B guides histone demethylase inhibitor development. *Nat. Chem. Biol.*, (may), 1–10.

25. Xu, W., Podoll, J.D., Dong, X., Tumber, A., Oppermann, U., and Wang, X. (2013) Quantitative analysis of histone demethylase probes using fluorescence polarization. *J Med Chem*, **56**, 5198–5202.

26. Allali-Hassani, A., Wasney, G. a, Siarheyeva, A., Hajian, T., Arrowsmith, C.H., and Vedadi, M. (2012) Fluorescence-based methods for screening writers and readers of histone methyl marks. *J. Biomol. Screen.*, **17** (1), 71–84.

27. Hatch, S.B., Yapp, C., Montenegro, R.C., Savitsky, P., Gamble, V., Tumber, A., Ruda, G.F., Bavetsias, V., Fedorov, O., Atrash, B., Raynaud, F., Lanigan, R., Carmichael, L., Tomlin, K., Burke, R., Westaway, S.M., Brown, J.A., Prinjha, R.K., Martinez, E.D., Oppermann, U., Schofield, C.J., Bountra, C., Kawamura, A., Blagg, J., and Brennan, P.E. (2017) Assessing histone demethylase inhibitors in cells : lessons learned. *Epigenetics Chromatin*, 1–17.

28. Wu, T.D., and Nacu, S. (2010) Fast and SNP-tolerant detection of complex variants and splicing in short reads. *Bioinformatics*, **26** (7), 873–81.

29. Gapp, B. V, Konopka, T., Penz, T., Dalal, V., Bürckstümmer, T., Bock, C., and Nijman, S.M.B. (2016) Parallel reverse genetic screening in mutant human cells using transcriptomics. *Mol. Syst. Biol.*, **12** (8), 879.

30. Totrov, M.; Abagyan, R., Flexible protein-ligand docking by global energy optimization in internal coordinates. *Proteins*, **1997**, *Suppl 1*, 215–220.

31. Wright ,T. H., Bower, B. J., Chalker, J. M., Bernardes G. J. L., Wiewiora R., Ng, W.-L., Raj, R., Faulkner, S., Vallée M. R. J., Phanumartwiwath A., Coleman, O. D., Thézénas M.-L., Khan, M., Galan S. R. G., Lercher L., Schombs M. W., Gerstberger S., Palm-Espling M. E., Baldwin A. J., Kessler B. M., Claridge T. D. W., Mohammed S., Davis B. G., Posttranslational mutagenesis: A chemical strategy for exploring protein side-chain diversity. *Science*, **2016**, *354* (6312), aag1465-5.

32. Cheng, Z.; Cheung, P.; Kuo, A. J.; Yukl, E. T.; Wilmot, C. M.; Gozani, O.; Patel, D. J., A molecular threading mechanism underlies Jumonji lysine demethylase KDM2A regulation of methylated H3K36. *Genes Dev.*, **2014**, *28* (16), 1758–1771.
